# The addition of *Psathyrostachys Huashanica* Keng 6Ns large segment chromosomes has positive impact on stripe rust resistance and plant spikelet number of common wheat

**DOI:** 10.1186/s12870-024-05395-9

**Published:** 2024-07-18

**Authors:** Jiaojiao Li, Jiachuang Li, Xueni Cheng, Zujun Yang, Yuhui Pang, Chunping Wang, Jun Wu, Wanquan Ji, Xinhong Chen, Jixin Zhao

**Affiliations:** 1 College of Agronomy, Henan University of Science and Technology, Luoyang, Henan, 471023 China; 2 College of Agronomy, Northwest A&F University, Yangling, Shaanxi 712100 China; 3 College of Life Science, Northwest A&F University, Yangling, Shaanxi 712100 China; 4 School of Life Sciences and Technology, University of Electronic Science and Technology of China, Chengdu, Sichuan 610000 China

**Keywords:** Distant hybridization, *Psathyrostachys Huashanica*, Addition line, Large segment chromosome, Wheat

## Abstract

**Background:**

Developing novel germplasm by using wheat wild related species is an effective way to rebuild the wheat resource bank. The *Psathyrostachys huashanica* Keng (*P. huashanica*, 2*n* = 2*x* = 14, NsNs) is regarded as a superior species to improve wheat breeding because of its multi-resistance, early maturation and numerous tiller traits. Introducing genetic components of *P. huashanica* into the common wheat background is the most important step in achieving the effective use. Therefore, the cytogenetic characterization and influence of the introgressed *P. huashanica* large segment chromosomes in the wheat background is necessary to be explored.

**Results:**

In this study, we characterized a novel derived line, named D88-2a, a progeny of the former characterized wheat-*P. huashanica* partial amphiploid line H8911 (2*n* = 7*x* = 49, AABBDDNs). Cytological identification showed that the chromosomal composition of D88-2a was 2*n* = 44 = 22II, indicating the addition of exogenous chromosomes. Genomic in situ hybridization demonstrated that the supernumerary chromosomes were a pair of homologues from the *P. huashanica* and could be stably inherited in the common wheat background. Molecular markers and 15 K SNP array indicated that the additional chromosomes were derived from the sixth homoeologous group (i.e., 6Ns) of *P. huashanica*. Based on the distribution of the heterozygous single-nucleotide polymorphism sites and fluorescence in situ hybridization karyotype of each chromosome, this pair of additional chromosomes was confirmed as *P. huashanica* 6Ns large segment chromosomes, which contained the entire short arm and the proximal centromere portion of the long arm. In terms of the agronomic traits, the addition line D88-2a exhibited enhanced stripe rust resistance, improved spike characteristics and increased protein content than its wheat parent line 7182.

**Conclusions:**

The new wheat germplasm D88-2a is a novel cytogenetically stable wheat-*P. huashanica* 6Ns large segment addition line, and the introgressed large segment alien chromosome has positive impact on plant spikelet number and stripe rust resistance. Thus, this germplasm can be used for genetic improvement of cultivated wheat and the study of functional alien chromosome segment.

**Supplementary Information:**

The online version contains supplementary material available at 10.1186/s12870-024-05395-9.

## Background

Because wheat is required for basic human nutritional needs, further improvements in wheat disease resistance, yield and processing quality are top priorities for wheat breeding [1]. Stripe rust caused by *Puccinia striiformis* f. sp. *tritici.* (*Pst*) is a severe wheat disease that affects more than 80% wheat-growing areas around the world. Its pathogenic spores can be transmitted over long distance via high-altitude airflow, which mainly harm the wheat leaves and sheaths by destroying the synthesis of chlorophyll. This destruction causes the decrease of photosynthetic capacity, and hinders the grain filling, leading to the probabilistic estimated damage of 5.47 million tons of grain each year [2, 3]. Moreover, the rapid evolution and spread of new pathogenic stripe rust races often make the newly made wheat varieties lose their expected resistance [4, 5]. Improving wheat flour quality and protein content of grain can increase human health. Wheat flour properties are mainly influenced by the composition of protein subunits; however, due to the limited number of high-quality subunits available for breeding, this has led to a high level of homogeneity of protein subunits in the current cultivated varieties, which seriously restricts the quality improvement of wheat [6, 7]. Since 1960s, the green revolution genes *Rht1* and *Rht8* have improved the lodging resistance and harvest index of wheat by reducing plant height, thus the increase of grain yield [8]. While the wheat plant height is now obviously dwarfed, the spike as a product organ has not been significantly improved in morphological type when comparison to the past. The limited capacity of the reproductive organs to absorb nutrients (i.e., small sink) is also a major reason for the bottleneck in wheat yield growth [9, 10]. Therefore, there are many wheat breeding problems that need to be solved, and the genetic background of cultivated wheat under the modern agricultural system is increasingly narrow, making it extremely difficult to achieve breeding breakthroughs solely through limited crossing between conventional wheat varieties [11].

Fortunately, wild relatives of wheat are normally living under complex and changeable natural conditions, and their rich genetic diversity gives them many excellent and desirable traits. Considering the five main diseases of wheat as an example, 17 stripe rust resistance genes, 35 leaf rust resistance genes, 30 stem rust resistance genes, 41 powdery mildew resistance genes and 3 fusarium head blight resistance genes from related species have been officially named by the International Committee on Nominating New Genes of Wheat, accounting for 21%, 44%, 50%, 63% and 43% of the total named genes, respectively [12, 13]. In terms of field breeding, the introgression of exogenous material from *Aegilops* spp. improved the processing quality of dough, especially for bread making [14]. The addition of genetic components from *Thinopyrum* and *Dasypyrum* increased the yield of common wheat [15, 16]. A well-known example of the use of related species to improve wheat is the development of the wheat-rye 1B/1R translocation line, which significantly improved the wheat yield and disease resistance. The rye 1RS chromosome is still favoured by breeders today, as it is present in more than 1,000 wheat varieties around the world [17, 18]. Therefore, further exploration of superior genes in related species by distant hybridization and chromosome engineering holds inestimable application prospects.

*Psathyrostachys huashanica* Keng (2*n* = 2*x* = 14) from the *Gramineae* family, is a diploid perennial species that grows only on high-altitude rocky slopes of the Huashan Mountains (Shaanxi Province, China) and possesses numerous excellent traits, including abiotic stress tolerance, multiple tillers, outstanding kernel quality and early maturity [7, 19, 20]. *P. huashanica* is self-incompatibility meaning that the progeny can only be obtained through cross-pollination. Moreover, *P. huashanica* was listed in the National Rare and Endangered Plants and Red List of Biodiversity in China (Endangered grade: critically endangered) due to its small populations and single distribution area, and it is commonly regard as the ‘giant panda’ in *Poaceae* plants in China. To break the restriction of reproductive isolation, distant hybridization between *P. huashanica* and common wheat was realised by Chen et al. [21] via embryo rescue, and several wheat-*P. huashanica* derived lines were gradually selected via multigenerational self-cross and backcross. These alien introgression lines showed various traits because of the different genetic material obtained from *P. huashanica*. For example, the 1Ns chromosome made the recipient wheat awnless, improved kernel quality and resistant to leaf rust [7]; the 2Ns chromosome made the recipient wheat express long spike and middle resistant to wheat take-all disease [22]; the 3Ns chromosome made recipient wheat resistant to powdery mildew [23]; the 4Ns and 5Ns chromosome made recipient wheat have superior stripe rust resistance [24, 25]; the 6Ns chromosome made recipient wheat have more spikelet and early maturation trait [26, 27]; and the 7Ns chromosome caused wheat to mature early [28]. These desirable exogenous traits can be effectively introgressed into the main wheat varieties through chromosome engineering to achieve yield improvement [29, 30]. Therefore, as far as breeding improvement of common wheat and effective utilization of *P. huashanica* are concerned, it is important to continuously develop wheat-*P. huashanica* derived lines with excellent characteristics.

In the present study, a novel wheat-alien derived line with outstanding traits was developed from BC_1_F_8_ progenies of common wheat and *P. huashanica*, named D88-2a. The main objectives of this research were (1) to examine the inheritance and pairing of introgressed alien chromosomes through cytogenetic methods; (2) to determine the chromosomal composition by using molecular marker analysis, in situ hybridization, and single-nucleotide polymorphism (SNP) array genotyping; and (3) to investigate agronomic and morphologic traits of this new alien introgression line.

## Materials and methods

### Plant materials

**Fig. 1:**
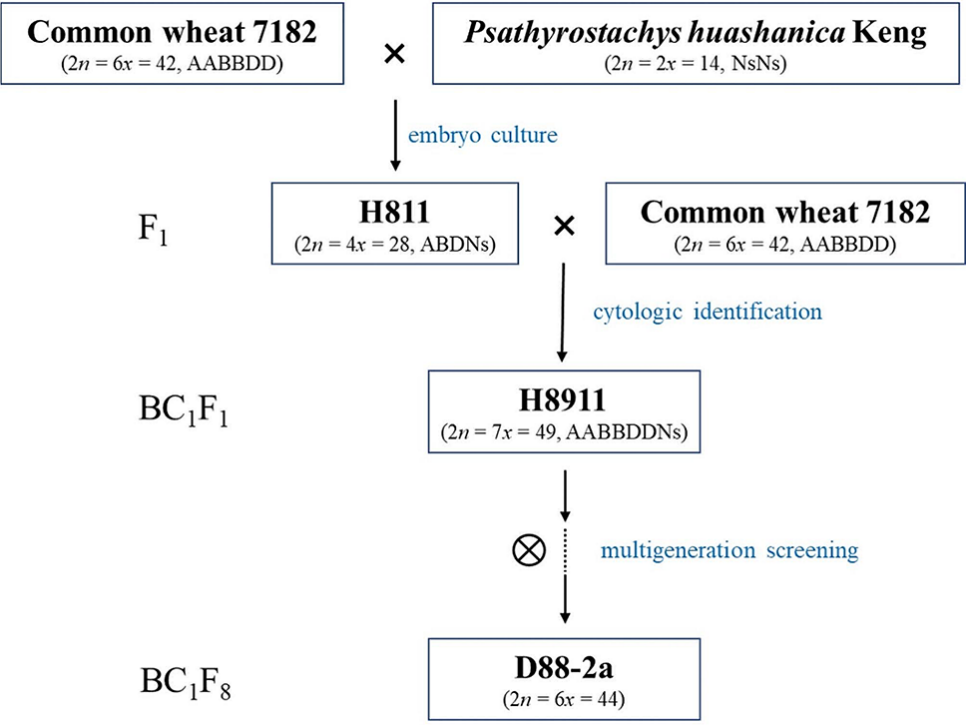
Scheme of the development of wheat-*P. huashanica* addition line D88-2a

The materials used in this research comprised the common wheat line 7182 and common wheat cultivated variety Huixianhong (HXH, 2*n* = 42, AABBDD), durum wheat line Trs-372 (2*n* = 28, AABB), *Psathyrostachys huashanica* Keng (2*n* = 14, NsNs) and wheat–*P. huashanica* addition line D88-2a. D88-2a was developed from the selfing of the partial amphiploid line H8911 (2*n* = 49, AABBDDNs), which was the BC_1_F_1_ progeny of 7182 × *P. huashanica* (Fig. 1); 7182 was the recurrent parent. About 18 plants were screened per generation based on cytogenetic observation (chromosome counting, see behind paragraph) and field morphology record, from which found D88-2a had stable chromosome number and morphological traits for three consecutive years. Durum wheat Trs-372 was used as an AB genome control in marker analysis, and the HXH was the susceptible control in the stripe rust resistance investigation because of its high susceptible trait to powdery mildew and stripe rust. Genomic DNA extraction used the modified cetyltrimethylammonium bromide (CTAB) method from leaf tissues [31].

### Cytological observation

The young spikes and root tips of all the genotypes were sampled at a particular period for cytological analysis, i.e., at meiotic metaphase I of pollen mother cells (PMCs) and mitosis metaphase of root tip cells (RTCs). During this step, the roots, spikes and grown plants were numbered to ensure one-to-one correspondence. For metaphase cell accumulation, the roots were pretreated in 20 µM amiprofos-methyl for 2 h. All samples were treated with Carnoy’s fixative solution (ethanol: ethanoic acid = 3:1, v/v) for 8 h, transferred to 70% ethyl alcohol and stored at 4℃. For the roots, the apical meristems were rinsed with 0.075 M potassium chloride (KCl) for 5 min and then digested in enzyme solution (1% pectinase and 2% cellulase in 2×SSC, pH = 4.5) in a 37℃-water bath for 55–65 min. Precooled 70% ethanol was used to wash off the solution before cleaving the root tip into single cells. After drying root tip cells, 18 $\:\mu\:$L ethanoic acid was added to each root as a suspending agent. For the spikes, the PMCs taken from each anther were screened until meiotic metaphase I stages, and the anther size indicated its developmental progress. The RTCs and anthers were stained using 1% acetocarmine that could only dye the genetic material. Then, the number of chromosomes and bivalent in intact cells was counted. Microscopic observations were performed using a BX60 positive microscope (OLYMPUS, Japan) at 400× magnification. The good division phases were crosslinked on slides for the following experiments through UV crosslinking (UVP, USA) at 1,250 mj/cm^2^ for 2 min. Ten plants were randomly selected annually in three consecutive years during cytological screening.

### In situ hybridization

Genomic in situ hybridization (GISH) was performed according to Wang et al. [32]. Briefly, *P. huashanica* genomic DNA was labelled with DIG-11-dUTP using a Dig-nick Translation Mix Kit (Roche, Germany) for 90 min, and the alien chromosomes were visualized by combining anti-digoxigenin-fluorescein. All chromosomes were counterstained by Vectashield H-1300 with propidium iodide (PI) (VECTOR, USA). In the nondenaturing fluorescence in situ hybridization (ND-FISH) experiment, fluorescent probes HS-TZ3, HS-TZ4, pSc119.2, pTa535-1 and Oligo-D [33, 34] were synthesized by Invitrogen (Thermo Fisher Scientific, China Branch). For non-denaturing fluorescence in situ hybridization, each glass slide with a split phase was hybridized with the specific probe solution (30 ng probe in 10 µL 2×SSC) in a 42℃ incubator for 4 h; the procedures are detailed by An et al. [17]. Chromosomal fluorescent karyotypes of wheat and *P. huashanica* were provided by Wang et al. [32] and Li et al. [33]. Then, the slides with excellent fluorescent signals in ND-FISH experiments were kept for sequential GISH analysis. To eliminate the original signals, the samples were washed with 70% ethanol in a 42℃-water bath for 30 min and exposed under light for 24 h. The fluorescent signals on chromosomes were observed and captured through a positive fluorescence microscope Imager M2 (ZEISS, Germany) with Photometrics SenSys cameras ICc5 and 503 (ZEISS, Germany).

Genomic in situ hybridization (GISH) was performed according to Wang et al. [32]. Briefly, *P. huashanica* genomic DNA was labelled with DIG-11-dUTP using a Dig-nick Translation Mix Kit (Roche, Germany) for 90 min, and the alien chromosomes were visualized by combining anti-digoxigenin-fluorescein. All chromosomes were counterstained by Vectashield H-1300 with propidium iodide (PI) (VECTOR, USA). In the nondenaturing fluorescence in situ hybridization (ND-FISH) experiment, fluorescent probes HS-TZ3, HS-TZ4, pSc119.2, pTa535-1 and Oligo-D [33, 34] were synthesized by Invitrogen (Thermo Fisher Scientific, China Branch). For non-denaturing fluorescence in situ hybridization, each glass slide with a split phase was hybridized with the specific probe solution (30 ng probe in 10 µL 2×SSC) in a 42℃ incubator for 4 h; the procedures are detailed by An et al. [17]. Chromosomal fluorescent karyotypes of wheat and *P. huashanica* were provided by Wang et al. [32] and Li et al. [33]. Then, the slides with excellent fluorescent signals in ND-FISH experiments were kept for sequential GISH analysis. To eliminate the original signals, the samples were washed with 70% ethanol in a 42℃-water bath for 30 min and exposed under light for 24 h. The fluorescent signals on chromosomes were observed and captured through a positive fluorescence microscope Imager M2 (ZEISS, Germany) with Photometrics SenSys cameras ICc5 and 503 (ZEISS, Germany).

### Molecular marker analysis

Seventeen pairs of specific sequence characterized amplified region (SCAR) markers [35, 36] were screened to confirm the supernumerary chromosomes in line D88-2a belonging to the *P. huashanica* Ns genome. Polymerase chain reaction (PCR) was conducted using 2× Taq plus PCR Master Mix with Dye (Biosharp, China), according to the manufacturer’s directions. The products were electrophoresed on 1% agarose gels. In addition, a total of 124 pairs of sequence tag site (STS) markers with good collinearity relationships were selected to distinguish the homoeologous group of introgressed chromosomes. The detailed information of SCAR and STS markers could be seen in supplementary material (Table S1. Detail information of molecular markers). The products were visible using 8% polyacrylamide electrophoresis and the alkaline silver dyeing method [37]. Table 1 showed some representative markers.

**Table 1:** Representative ND-FISH probes, wheat STS markers and *P. Huashanica* SCAR markers used in this study. The 6-FAM and TAMRA were fluorescent adaptor of probes

Markers	Type	Primer (5′ − 3′)	Tm (°C)	location
TC249514	STS	**F**: GCCAGGTCAAGGAGGGGAAAG **R**: CGCCGAATCATACCGAATCATC	55	6DL
MWG652	STS	**F**: GAGCTGCTCGTTCTCGTTGA **R**: CACACCTTCTTCTTCCTCTT	60	6AS 6BS
CD452568	STS	**F**: TTTGCATTTTCGTCTGCAAG **R**: TCGACACGAGCAAGATTCAC	60	6AL 6BL 6DL
RHS23	SCAR	**F**: ACGCAGGCACGTTCTGATGACTACT **R**: ACGCAGGCACCAAATAACAATTATT	70	1Ns–7Ns
pSc119.2	Oligo	**6-FAM-** CCGTTTTGTGGACTATTACTCACCGCTTTGGGGTCCCATAGCTAT	42	B-genome
pTa535-1	Oligo	**TAMRA-** AAAAACTTGACGCACGTCACGTACAAATTGGACAAACTCTTTCGGAGTA TCAGGGTTTC	42	A, D-genome
Oligo-D	Oligo	**TAMRA-** TACGGGTGCCAAACGAGTGTCTGAAAGACTCCTCGAGAGGAAAATGCGAA	42	D-genome
HS-TZ3	Oligo	**6-FAM-** AGGCTCACACTAGAGAAGAAACGGTGGAAAAATAGAAGAAAAGAATC	42	Ns-genome
HS-TZ4	Oligo	**TAMRA-** CAATCGGAGCTGGTTCTCATCCGCGTTGATAGTCCCTATCAA	42	Ns-genome

### Wheat 15 K SNP array analysis

RNase purified genomic DNA (gDNA) of D88-2a and its parents were hybridized with a wheat 15 K solid SNP array for genotype comparison at the China Golden Marker Biotechnology Company (Beijing, China) [38]. After filtration, there were 10,782 valid SNP loci distributed on 21 wheat chromosomes. The heterozygous rate of each chromosome was equal to the number of heterozygous genotypes divided by the total valid SNP marker number. In addition, a comparison of each SNP locus between D88-2a and 7182 was analysed in Wheat Gmap online tools [39], and IWGSC RefSeq assembly v2.0 was used as the reference genome. The number of mutated loci in materials was counted for drawing.

#### Gliadin subunit and kernel quality comparison

Acid-polyacrylamide gel electrophoresis (A-PAGE) was employed to detect the subunit composition of gliadin. With reference to Liu et al. [40] and Li et al. [41], the extract was added to the powdered endosperm, and after 12 h of extraction in the dark, the supernatant containing gliadin was obtained by centrifugation at 14,000 rpm for 10 min. Electrophoresis was performed at a constant voltage of 60 V until the strip ran out of the glass plate. Subunit bands were stained using Coomassie brilliant blue G250 and decolourised with water. The kernel quality-related parameters including crude protein content, gluten protein content, starch content, and flour yield were measured using a DA 7250 NIR analyzer (Pertern Instrument, Sweden) and single grain analyzer SKCS-4100 (Perten Instrument, Sweden) with kernels. Each sample was tested in triplicate and ultimately used the average. So, the procedure of biscuit making referred to the Standardization Administration of the People’s Republic of China SB/T10141-93 ‘wheat flour for fermenting biscuits’. Referring to Rakshit and Srivastav [42], the colour saturation (ΔC*) value of biscuits were measured using a CM-5 spectrophotometer (Konica Minolta, Shanghai, China). Each sample was tested in triplicate and ultimately used the average.

### Morphological traits and wheat stripe rust resistance evaluation

In the field, each genotype was planted in three rows (row space 30 cm and row length 1.2 m, six plants per row and 15 cm between each plant) at the farm of Northwest A&F University, Yangling, China (108°08′E, 34°27′N) and harvested in June. The agronomic traits of the materials were evaluated, comprising the following eight traits: plant height, tiller number, spike length, spikelet number, spikelet type (i.e., normal, paired spikelet and ear-branched spikelet), kernels per spike, thousand-kernel weight and grain yield per plant. Six samples collected annually in three successive years were investigated to ensure that the obtained values were accurate. The significant differences were analysed using ANOVA (LSD test) at *p* = 0.05 level through PASW Statistics 18 software (IBM Corp., USA).

The *Pst* mixed races (CYR33 and CYR34) were used for artificial inoculation at the jointing stage to evaluate adult plant resistance to wheat stripe rust. In March of 2020 and 2021, temperatures around 20℃, the rust spores were smeared onto the flag leaf of materials in the form of a slight scratch after a spring drizzle to obtain better infection. Huixianhong was the susceptible control, and the infection types (ITs) of each material were graded based on the standards mentioned in Ma et al. [43] and An et al. [17]. In detail, IT ranged from 0 to 4, in which 0 indicated immune (no visible symptom), 0; indicated nearly immune (no congregate urediospore and hypersensitive flecks), 1 indicated high resistance (few small urediospores visible embedded in well-defined necrotic areas), 2 indicated moderate resistance (few small to medium-sized urediospores surrounded by necrotic areas), 3 indicated moderately susceptible (many medium-sized urediospores with chlorosis on leaves) and 4 indicated highly susceptible (a large number of large-sized urediospores with substantial necrosis on leaves); each grade was appended with “+” or “−” to emphasize heavier or lighter.

## Results

### Chromosome configuration observation and GISH analysis of D88-2a

First, we counted the root tip cell (RTC) chromosomes of D88-2a, the BC_1_F_8_ progeny of wheat and *P. huashanica*. Each individual cell had a chromosomal number of 44 in mitosis metaphase. Subsequently, the pairing and division process of chromosomes at the meiosis stage were observed (Table 2). These results showed that D88-2a has two extra chromosomes than that of common wheat.

**Table 2:** The chromosome numbers and pairing status in the meiotic phases of D88-2a

Material	Number of cells	2n	Chromosome configuration					
			Univalent	Bivalent			Trivalent	Quadrivalent
				Rod	Ring	Total		
D88-2a	165	44	0.11 (0–2)	1.05 (1–2)	20.78 (20–22)	21.83 (21–22)	0	0

**Fig. 2:**
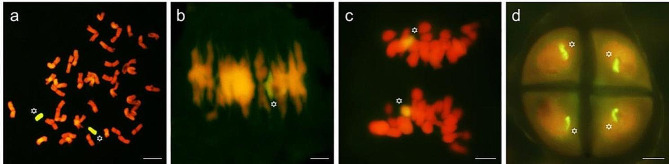
GISH detection of the wheat-*P. huashanica* derived line D88-2a genotype. The total gDNA of *P. huashanica* labelled with digoxigenin has given yellow fluorescent signals on the alien chromosomes, marked with asterisks. **(a)** GISH at mitotic metaphase. Two chromosomes with fluorescent signals were identified as alien chromosomes. **(b)** GISH at meiosis metaphase I, a yellow‒green colour ring bivalent was observed when *P. huashanica* chromosomes paired. **(c)** GISH at meiosis anaphase I. Two chromosomes with fluorescent signals moved to the poles with wheat chromosomes at meiosis anaphase (I) **(d)** GISH at meiosis telophase (II) Four progeny cells contained fluorescent signals that were alien chromosomes at the tetrad stage. The probe labelling used gDNA of *P. huashanica.* Propidium iodide (PI) made the chromosomes appear red or orange red, and asterisks refer to alien chromosomes. Scale bar = 10 μm

GISH experiments were conducted by using digoxigenin-labelled gDNA from *P. huashanica* as the probe to confirm the relationship between the additional chromosomes and the parental *P. huashanica* Ns genome. Two chromosomes in the mitotic RTCs at metaphase of D88-2a exhibited strong yellow-green fluorescent signals (Fig. 2a). At the metaphase I stage of PMCs, the two alien chromosomes formed a ring bivalent with hybridization signals on the cell equatorial plate (Fig. 2b, asterisk). Then, at meiosis anaphase I, the ring bivalent separated into two parts that each carried fluorescent signals and moved to the cell pole together with wheat chromosomes (Fig. 2c). At telophase II stage of meiosis, each cell of the tetrad possessed a chromosome from *P. huashanica* (Fig. 2d). Therefore, GISH analysis in mitosis and meiosis demonstrated that D88-2a was a wheat-*P. huashanica* disomic addition line and that the additional chromosome can be transmitted to the offspring normally, suggesting D88-2a is a cytologically stable line.

### Molecular marker analysis for alien chromosomes

**Fig. 3:**
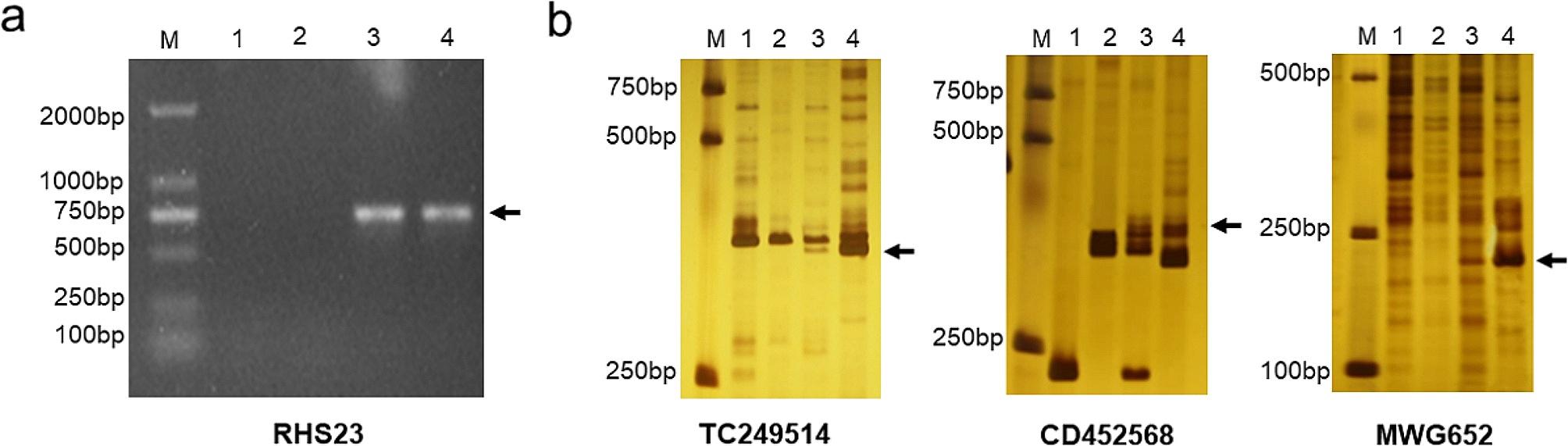
Analysis of homoeologous attribution of additional chromosomes in addition line D88-2a by markers. **(a)** Example of SCAR marker analysis. Marker RHS23 amplified unique and clear products (about 750 bp, arrow) in materials that contained Ns chromosomes. **(b)** Example of STS markers analysis. Three markers from homoeologous Group 6 amplified distinctive bands in D88-2a and *P. huashanica*. The arrows indicated Ns chromosome special bands amplified by marker TC249514 (about 350 bp of target product), CD452568 (about 370 bp of target product) and MGW652 (about 230 bp of target product). Lane M = DL2000 marker, lane 1 = common wheat parent 7182, lane 2 = durum wheat Trs-372, lane 3 = D88-2a addition line, and lane 4 = alien donor *P. huashanica*. Arrows refer to Ns genome–specific bands

STS and SCAR markers were used for preliminary identification of the homoeology of alien chromosomes in D88-2a. Among the 17 pairs of SCAR markers, the Ns genome-specific marker RHS23 amplified unique products in D88-2a and *P. huashanica* (Fig. 3a, arrows), indicating that the added alien chromosomes in line D88-2a were from *P. huashanica*. In addition, three pairs of STS markers (i.e., TC249514, MWG652 and CD452568) distributed in homoeologous Group 6 amplified Ns chromosome-specific bands in D88-2a and *P. huashanica*, but were absent in durum wheat Trs-372 and common wheat parent 7182 (Fig. 3b, arrows), suggesting that the alien chromosomes in D88-2a belongs to the sixth homoeologous group chromosomes from *P. huashanica*. Consequently, D88-2a is likely a wheat-*P. huashanica* 6Ns disomic addition line.

### Wheat 15 K SNP array typing for D88-2a

**Fig. 4:**
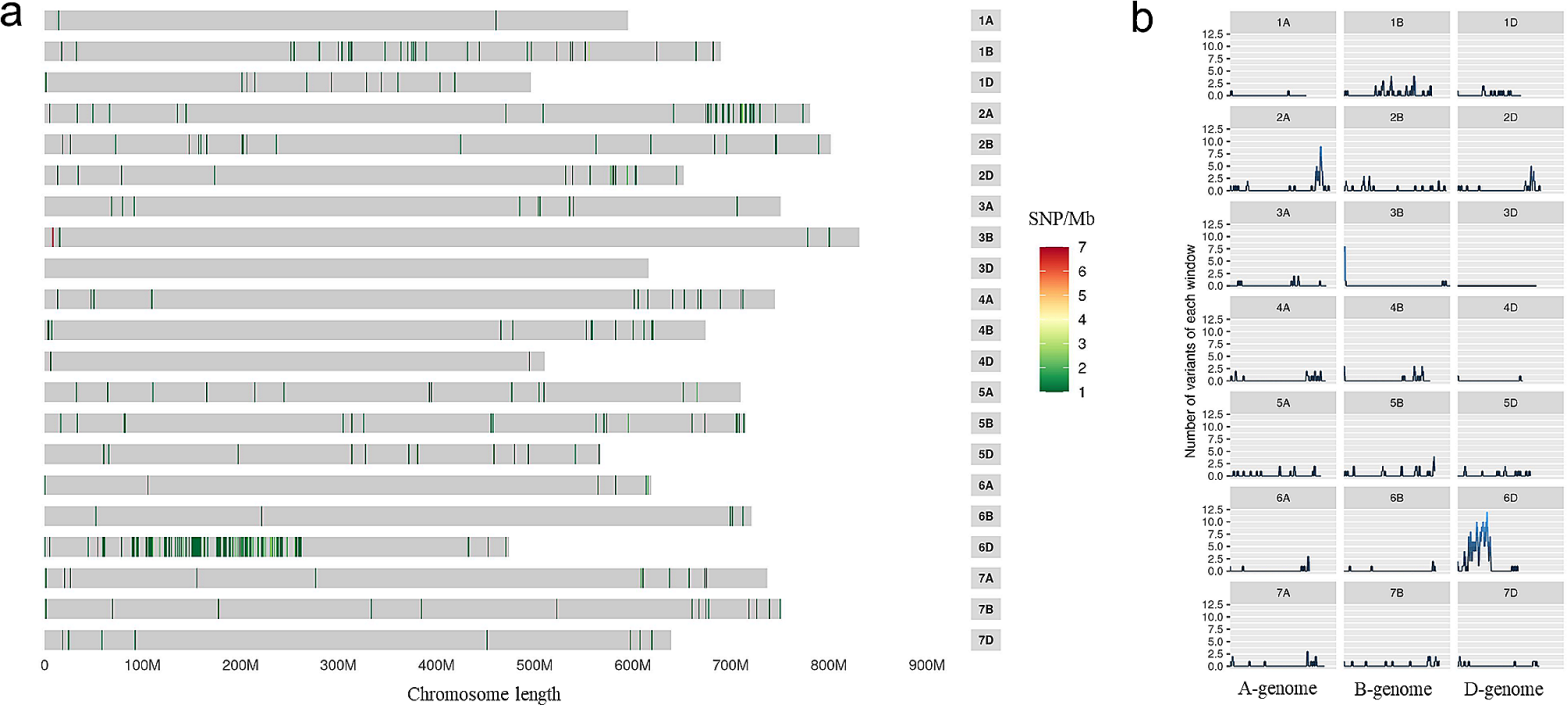
Differential SNP loci analysis between addition line D88-2a and parental common wheat 7182. **(a)** Distribution map of differential SNPs on each chromosome arranged by physical location. Only on the 6D chromosome, a concentration of differential SNP loci appeared between D88-2a and its wheat parent 7182. **(b)** The number of variation sites within a unit interval on each chromosome of D88-2a compared with common wheat 7182 and Chinese Spring. D88-2a expressed large variation sites on its 1B, 2 A, 3D and 6D, among which the sites on 6D showed more variation

The 15 K solid SNP array was adopted to clarify the chromosomal recombination state in D88-2a (Table S2). Based on the statistical data of the heterozygous rate (Table 3), the alien parent *P. huashanica* commonly had approximately 80% heterozygous SNPs on each chromosome, whereas the wheat parent 7182 had only approximately 5% heterozygous SNPs on its chromosomes. The SNP values of the derived line D88-2a were similar to those of 7182, except on the 6D chromosome, where the heterozygous genotype of SNPs accounted for 40% of the total. To make recombination zones easier to observe, each valid SNP was compared between D88-2a and its parent 7182 (Fig. 4a and b). It was obvious that the different SNPs mainly focused on the wheat 6D chromosome, embodied by more variations on the short arm. Those data suggest that the chromosome composition of the sixth homoeologous group was different between addition line D88-2a and 7182, and the SNPs in the 6D chromosome was mainly affected.

**Table 3:** Comparison of genotype data between D88-2a and its parents using a 15 K SNP array

Chromosome	Number of valid markers	Number (%) of heterozygous genotypes in *P*. huashanica	Number (%) of heterozygous genotypes in 7182	Number (%) of heterozygous genotypes in D88-2a
1 A	502	399 (79.5%)	22 (4.4%)	16 (3.2%)
1B	549	425 (77.4%)	32 (5.8%)	57 (10.4%)
1D	288	230 (79.9%)	11 (3.8%)	22 (7.6%)
2 A	762	592 (77.7%)	32 (4.2%)	51 (6.7%)
2B	579	413 (71.3%)	37 (6.4%)	35 (6.0%)
2D	512	398 (77.7%)	35 (6.8%)	41 (8.0%)
3 A	489	381 (77.9%)	14 (2.9%)	24 (4.9%)
3B	782	600 (76.7%)	38 (4.9%)	40 (5.1%)
3D	431	349 (81.0%)	24 (5.6%)	22 (5.1%)
4 A	652	535 (82.1%)	11 (1.7%)	19 (2.9%)
4B	464	340 (73.3%)	31 (6.7%)	33 (7.1%)
4D	213	163 (76.5%)	12 (5.6%)	12 (5.6%)
5 A	524	399 (76.1%)	17 (3.2%)	28 (5.3%)
5B	597	453 (75.9%)	41 (6.9%)	43 (7.2%)
5D	444	353 (79.5%)	27 (6.1%)	35 (7.9%)
6 A	354	260 (73.4%)	12 (3.4%)	21 (5.9%)
6B	587	430 (73.3%)	24 (4.1%)	20 (3.4%)
6D	329	263 (79.9%)	26 (7.9%)	137 (41.6%)
7 A	615	483 (78.5%)	26 (4.2%)	33 (5.4%)
7B	527	391 (74.2%)	27 (5.1%)	33 (6.3%)
7D	582	452 (77.7%)	24 (4.1%)	26 (4.5%)
A genome	3898	3049 (78.2%)	134 (3.4%)	192 (4.9%)
B genome	4085	3052 (74.7%)	230 (5.6%)	261 (6.4%)
D genome	2799	2208 (78.9%)	159 (5.7%)	295 (10.5%)
Total	10,782	8309 (77.1%)	523 (4.9%)	748 (6.9%)

### Chromosomal completeness analysis of 6D and 6Ns in D88-2a

**Fig. 5:**
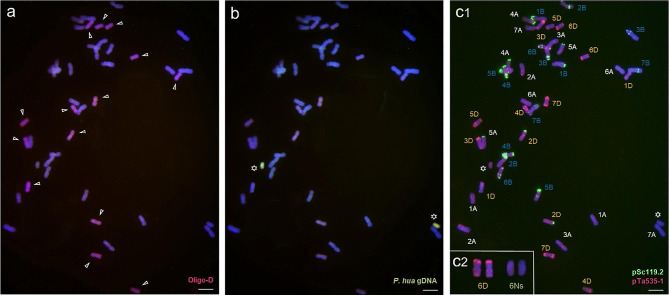
Cytogenetic analysis by FISH and sequential GISH for D88-2a and its parents. **(a)** ND-FISH of D88-2a. FISH probe Oligo-D (red) normally detected the 14 wheat D genome chromosomes, as indicated by arrows. **(b)** Sequential GISH of D88-2a. *P. huashanica* gDNA detected the alien chromosomes in yellow-green colour. c1. Mc-FISH used a probe set of pSc119.2 (green) and pTa535 (red). Examining the recombination sites of wheat chromosomes in D88-2a. c2. Comparison of hybridization signals between the wheat 6D and *P. huashanica* 6Ns in D88-2a. The 6D had normal FISH signals and the 6Ns had no hybridization signals. Chromosomes were counterstained with DAPI (blue) and PI (red). The asterisks indicate Ns chromosomes. Scale bar = 10 μm

The repetitive oligonucleotide probe Oligo-D was labelled with a red signal and was applied to determine the completeness of the 6D chromosomes. The results showed that D88-2a had the correct number of D genome chromosomes (Fig. 5a). GISH experiment using digoxigenin-labelled *P. huashanica* gDNA as a probe provided evidence that there were two alien chromosomes from *P. huashanica* (Fig. 5b). On the same slide, probes pSc119.2 and pTa535 were used to distinguish the structural variation of wheat chromosomes in D88-2a by comparing the FISH karyotype with the standard idiogram (Fig. 5c1). The standard FISH idiogram of common wheat was referenced to Tang et al. [34] and Du et al. [44]. The pattern of probes showed that the 6D chromosomes in D88-2a had normal FISH signals as it in the parent 7182, and the additional *P. huashanica* 6Ns chromosomes had no hybridization signals as it in *P. huashanica* by using the probe set of pSc119.2 and pTa535-1 (Fig. 5c2). Thus, these results suggest that although D88-2a possesses *P. huashanica* 6Ns chromosomes, the alien chromosomes do not influence the structure and composition of wheat 6D chromosomes, i.e. the additional 6Ns chromosomes were not structurally recombined with wheat 6D chromosome or induced the structural variation of 6D.

**Fig. 6:**
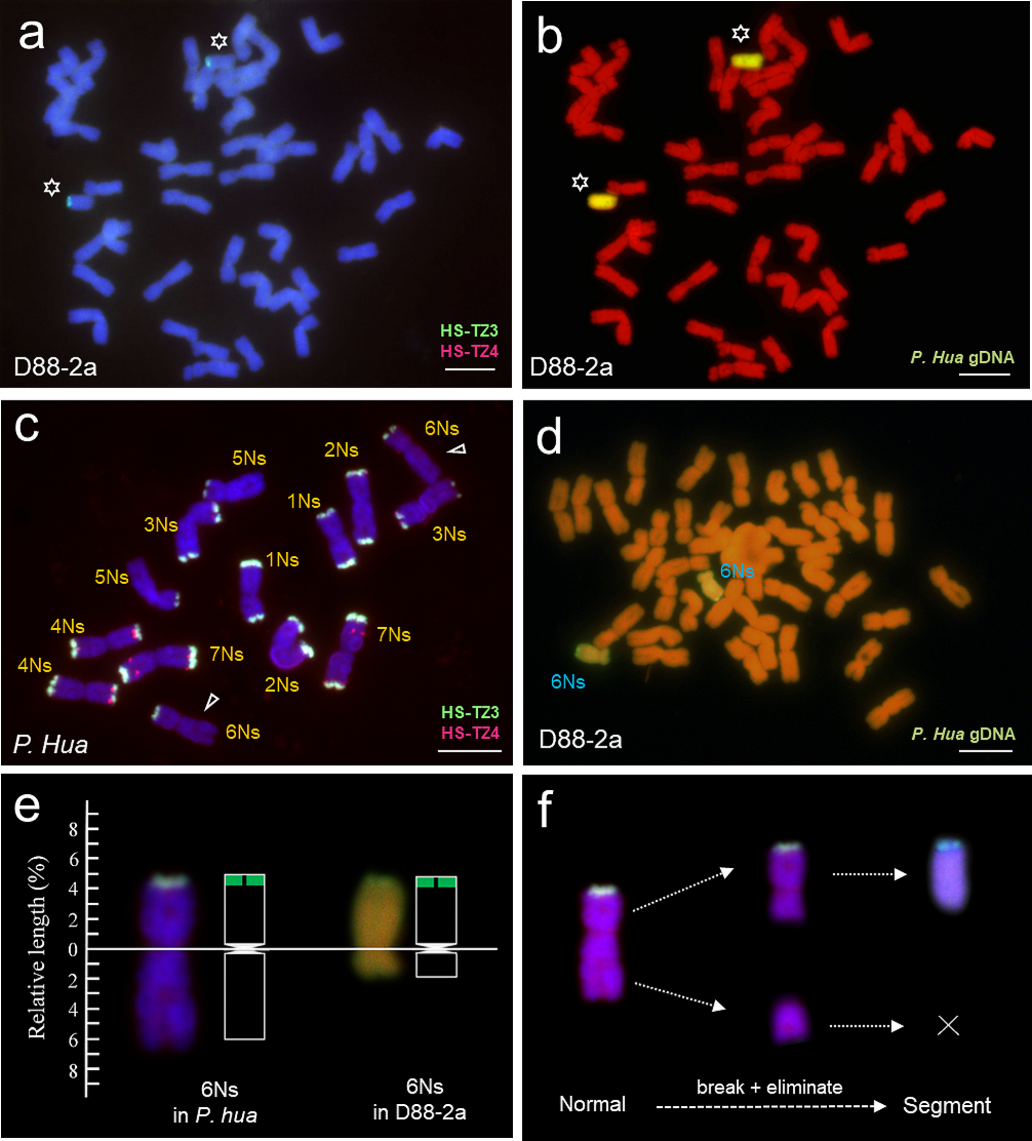
FISH karyotype and structural variation diagram of 6Ns chromosomes. **(a)** ND-FISH analysis of D88-2a using Ns genome-specific probe HS-TZ3 and HS-TZ4. The result showed that two chromosomes had fluorescent signals at their terminal parts. **(b)** Sequential GISH experiment behind FISH. The two chromosomes with fluorescent signals were *P. huashanica* chromosomes, and wheat chromosomes appear red or orange-red. **(c)** ND-FISH result of *P. huashanica* using probe set of HS-TZ3 and HS-TZ4. 6Ns chromosome had fluorescent signals only on its telomere region of the short arm. **(d)** GISH result to show the morphology of 6Ns chromosomes in D88-2a. The alien chromosomes carry yellow-green colour and the wheat chromosomes appear red or orange-red. **(e)** Comparison of 6Ns chromosomes in *P. huashanica* and in D88-2a. **(f)** The process of presumed structural variation. The normal 6Ns in *P. huashanica* went through breaking and eliminating to 6Ns segment in D88-2a. *P. hua* indicated *P. huashanica.* Chromosomes were counterstained with DAPI (blue) and PI (red). The asterisks indicate Ns chromosomes. Scale bar = 10 μm

The *P. huashanica* chromosomes were identified by using the Ns genome-specific probe set HS-TZ3 and HS-TZ4. D88-2a had two chromosomes expressing green terminal fluorescent, and sequential GISH showed that they were from the *P. huashanica* Ns genome (Fig. 6a and b). For probe HS-TZ3 and HS-TZ4, normal 6Ns had fluorescent signals only at the end of short arms (Fig. 6c). GISH experiment showed that the alien chromosome possessed its short arm and centromere in D88-2a (Fig. 6d). Based on the chromosome length of wheat 6D (493 Mb) [1], *P. huashanica* 6Ns (903 Mb, unpublished data) and the length comparison between alien chromosomes and 6D in Fig. 5c2 and 6d, we made the karyotype of 6Ns chromosomes in *P. huashanica* and D88-2a (Fig. 6e). In addition, we made a diagram of formation process to facilitate easier understand (Fig. 6f). Therefore, the result demonstrated that the pair of additional 6Ns chromosomes in D88-2a were large segment chromosomes which contained entire short arm and proximal centromere portion of the long arm.

### Effect of alien chromosomes on kernel quality

**Fig. 7:**
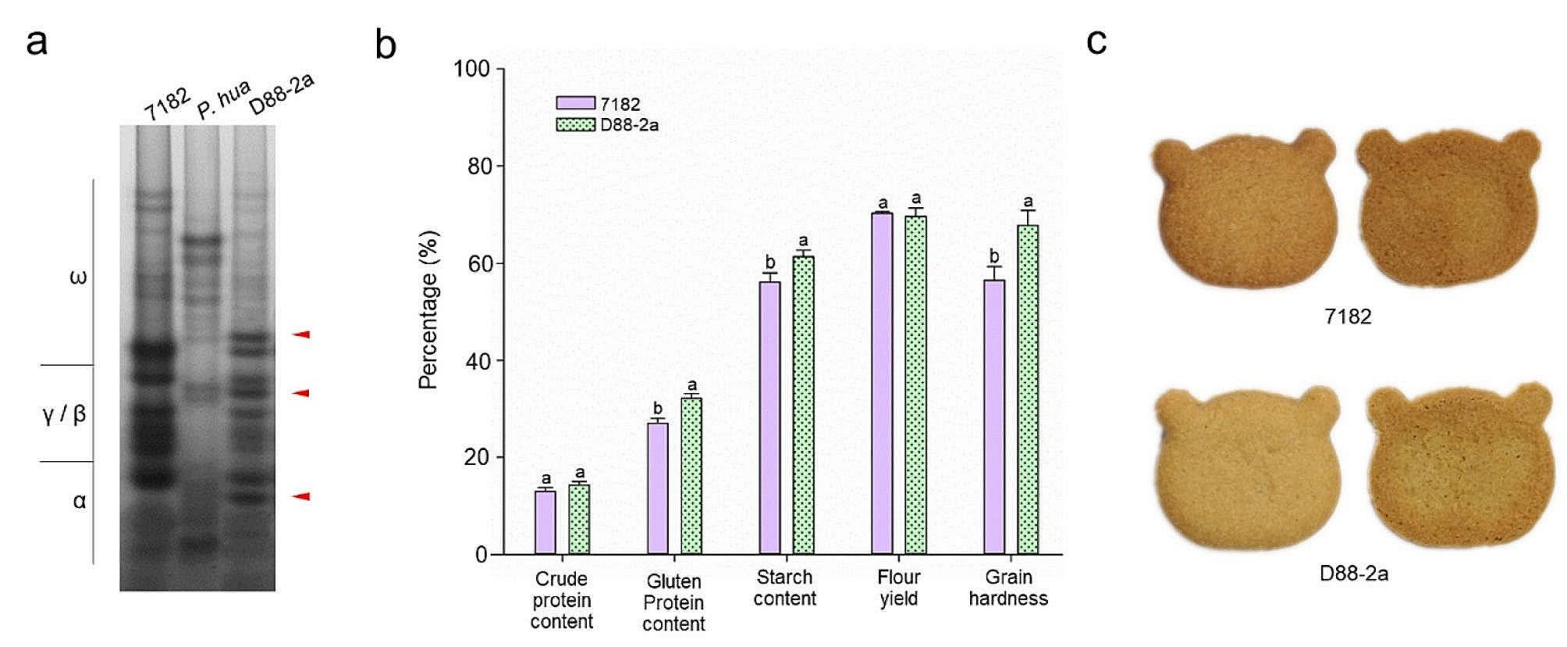
Kernel quality comparison of D88-2a and its parents. **(a)** Gliadins analysis through A-PAGE experiment. D88-2a inherited similar gliadin patterns with wheat parent 7182, but it also obtained three gliadins (ω-, γ/β- and α-gliadin) from *P. huashanica.* Red arrows indicate alien gliadins in D88-2a. **(b)** Kernel quality analysis of the D288-2a and parental 7182 line. D88-2a had higher gluten protein and starch content and grain hardness than its parent 7182. **(c)** Comparison of cookies made from D88-2a and 7182 flour. The front and back of cookies made with flour of 7182 and D88-2a, in which cookie from D88-2a expressed brighter-whiter colour

The gliadin band patterns separation of D88-2a and its parents using A-PAGE is shown in Fig. 7a. The gliadins of *P. huashanica* were mainly ω- and α-gliadin bands, in contrast to the bands of 7182 and D88-2a. Clearly, D88-2a not only inherited the same gliadin band pattern from 7182 but also possessed specific bands from the alien parent *P. huashanica*, as indicated by the arrows. According to the measured data (Fig. 7b), the gluten protein and starch contents in the kernels of D88-2a (32.14% and 61.36%, respectively) were significantly higher than those in the kernels of 7182 (26.96% and 56.09%, respectively) at *p* = 0.05. And the grain hardness of D88-2a was also higher 7182. The biscuits made with flours of D88-2a and 7182 showed significant differences of appearance under the same conditions. During biscuit baking, the Maillard reaction is an important factor affecting colour, and the greater the colour saturation (ΔC*) value, the brighter the biscuit colour [45]. Specifically, the ΔC^*^ value of D88-2a and 7182 were 2.28 ± 0.43 and 1.98 ± 0.37 respectively, demonstrated by the biscuits made from D88-2a expressed a brighter-white colour than those from 7182 (Fig. 7c). Therefore, the addition of the *P. huashanica* 6Ns chromosome has positive effects on the storage protein content of recipient wheat.

### Performance of stripe rust resistance and agronomic traits

**Fig. 8:**
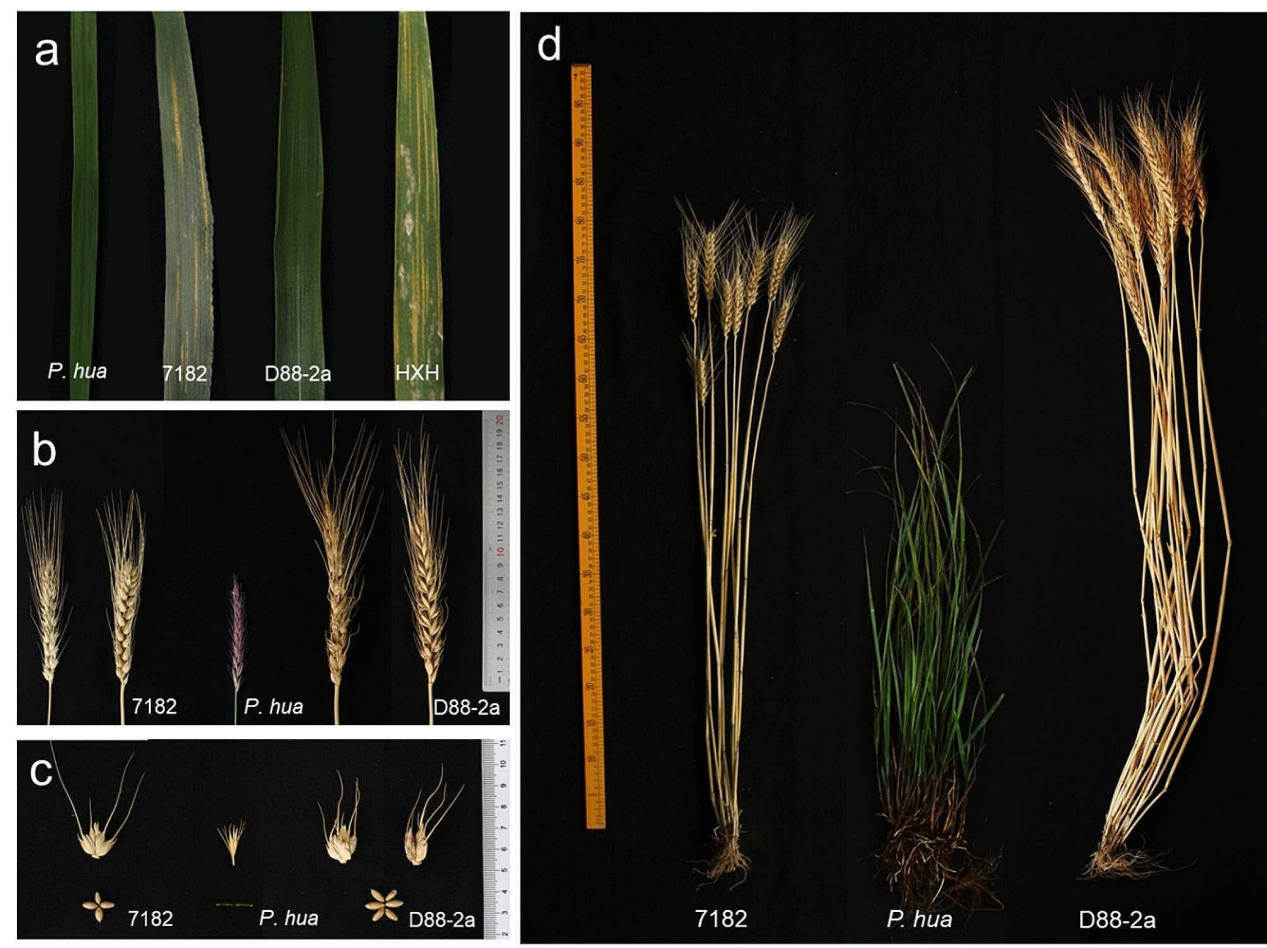
Stripe rust reaction and plant, spike and spikelet morphology of D88-2a, common wheat parent 7182 and alien donor *P. huashanica*. **(a)** Reaction to wheat stripe rust of the second leaf. Common wheat cultivar HXH was control. **(b)** Comparison of spikes. **(c)** Spikelets, D88-2a had paired spikelets. (d) Adult plants. *P. hua* indicated *P. huashanica*; HXH indicated Huixianhong

The resistance of adult plants to mixed stripe rust races was tested in the field. When the susceptible control wheat HXH exhibited sufficient disease symptom, and the wheat parent 7182 showed symptoms of infection, whereas line D88-2a was almost immune (Fig. 8a). The resistance of the materials were ranked by ITs: *P. huashanica*, IT = 0; D88-2a, IT = 0;; 7182, IT = 3-; HXH, IT = 4. Therefore, the excellent resistance of D88-2a to stripe rust could be attributed to the resistance genes from the *P. huashanica* 6Ns chromosome.

The morphological traits of D88-2a and its two parents are shown in Fig. 8. The distinct difference was the spike trait that D88-2a had long spikes and paired spikelets (Fig. 8b and c). D88-2a had more spikelet number, kernels per spike and yield per plant than the common wheat parent 7182 at *p* = 0.05 level (Table 4). Moreover, D88-2a were taller and had more tillers at *p* = 0.05 level than wheat parent 7182 (Fig. 8d; Table 4). The thousand-kernel weight were similar between D88-2a and 7182. These results indicate that *P. huashanica* 6Ns may has positive effect on spikelet number and tiller under our controlled growth condition.

**Table 4:** Agronomic traits of line D88-2a and its parents

Year	Materials	Plant height (cm)	Tiller number	Spike length (cm)	Spikelet number	Kernels per spike	Thousand kernel weight (g)	Grain yield /plant (g)
2020	*P. hua*	52.4	clustered	8.23	15	23	3.16	—
	7182	77.5	7	8.7	20	52	38.76	6.82
	D88-2a	95.3	13	13.23	33	78	38.25	12.55
2021	*P. hua*	49.5	clustered	9.07	18	30	3.3	
	7182	82.3	7	9.35	20	51	37.94	7.96
	D88-2a	89.5	16	12.5	31	72	39.46	17.19
2022	*P. hua*	57.7	clustered	8.73	16	26	3.42	—
	7182	74.8	9	8.55	18	48	39.56	9.08
	D88-2a	91.5	15	11.92	27	66	40.38	16.47
AVG	*P. hua*	53.6 ± 4.1^c^	clustered	8.65 ± 0.42^b^	16 ± 2^b^	26 ± 4^c^	3.29 ± 0.13^b^	—
	7182	77.1 ± 2.3^b^	8 ± 1^b^	8.95 ± 0.4^b^	18 ± 2^b^	50 ± 2^b^	38.75 ± 0.81^a^	8.45 ± 1.63^b^
	D88-2a	92.4 ± 2.9^a^	14 ± 2^a^	12.58 ± 0.66^a^	30 ± 3^a^	68 ± 6^a^	39.32 ± 1.06^a^	14.87 ± 2.32^a^

The AVG mean average. The lowercase letters indicate significant differences of each column at *p* = 0.05 level, statistic using the LSD test

## Discussion

A complex and changeable cultivation environment requires the accumulation of resist variation in wheat, among which the naturally existing superior genes are more stable and easier to obtain than the artificially modified ones. Since Backhouse [46] first crossed wheat and rye to produce hybridized offspring with beneficial traits, the scientific community was inspired by the fact that more than 320 wheat relatives in the *Triticeae* family could be genetic donors for common wheat. At present, alien genes conferring specific traits on plants have been added into wheat through extensive crosses, and one of the effective methods is through the generation of alien addition lines [17, 47]. For example, according to the difficulty rate of alien chromosome introgression, the earliest wheat-*Aegilops comosa* progeny was the 2 M addition line [48], and the earliest wheat-*Haynaldia villosa* progeny were 2 V, 4 V, 5 V and 6 V addition lines [49]. Based on addition lines, other types of alien introgression lines (e.g., substitution lines, translocation and introgression lines) can be further created through mutagenesis, cell engineering and tissue culture [28, 29]. In the study, we developed a novel wheat-alien derived line via distant hybridization that exhibited several superior traits than its parental lines because of the addition of 6Ns chromosome from *P. huashanica*.

A derived line aroused the interest of researchers when it showed significant outstanding traits than its parents over the years. To understand the causes of these changes, determining the genetic background of this line was the first step. Cytogenetic observation of RTCs and PMCs in particular stages is a classical way to thoroughly understand the chromosomal composition [20, 50]. GISH technology can clearly visualize the number and behaviour of alien chromosomes in the plant genetic background. Therefore, we employed these two approaches to clarify that the two extra chromosomes originated from the *P. huashanica* genome, and found that they were homoeologues chromosomes. A wheat-alien derived line can only be used in field breeding programs only when the introgressed alien component can be stably inherited by the offspring, otherwise the loss of the alien components will result in the loss of superior traits [51]. Our GISH experiment in meiosis stages of PMCs showed that the alien chromosomes were genetically stable in D88-2a.

The underlying reasons of phenotypic change in the early breeding process were unclear, and the regulations could only be discovered through multi-round hybridization and long-term screening [52]. Therefore, modern wheat breeding can further promote the research and utilization of derived lines by identifying the homoeology of introgressed alien chromosomes and their recombination with wheat chromosomes [53]. Genome-specific molecular markers are simple and accurate tools for foreign genetic material analysis. For example, Ren et al. [54] confirmed that the 6RS chromosome was introgressed in wheat-rye line 117-6 using SCAR markers, and Zhang et al. [55] found that *Thinopyrum intermedium* 6StS.6JsL chromosomes existed in line CH51 through SNP array. Similarly, we determined that D88-2a contained *P. huashanica* homologous Group 6 chromosomes by comprehensively using multi-type molecular markers (SCAR, STS and SNP array).

Previously, a wheat SNP array was used to analyse wheat-alien substitution and translocation lines because the lost wheat chromosomes caused a large number of deletions in SNP loci [44]. This study found that the 15 K SNP array could also accurately identify the homoeologous groups of alien chromosomes in addition lines, which might be because the genotype of SNPs on the target homologous group chromosomes were affected by alien chromosomes. Specifically, because of the collinearity of genes on the chromosomes of *Triticeae*, the alien genetic components (heterozygous genotype) and wheat genetic components (homozygous genotype) competitively bind with given loci in the array, which leads to great changes in the heterozygosity rate on corresponding chromosomes. However, considering the homozygosity of genomic loci, the 15 K SNP array is only recommended for application to the addition lines developed from wheat and its self-incompatibility related species. It is worth noting that the addition of 6Ns chromosomes only caused a large number of different SNPs on the wheat 6D chromosome, but not 6 A and 6B. According to Anamthawat-Jónsson [56], the sequences on the same homoeologous groups of different species have high collinearity. Also, considering almost all the interaction and recombination happened between wheat and *P. huashanica* chromosomes were in D genome and Ns genome [16, 22, 57–59], we suspect that 6Ns may have better collinearity or higher genetic similarity with 6D rather than 6 A or 6B.

There are approximately 83–92% repeated sequences in the genome of *Triticeae* species [60, 61], and these sequences have a strong linear correspondence on the chromosomes of different species within the family, which allows oligo-probes designed from repeated sequences to be used jointly between related species [62, 63]. Therefore, oligo-pSc119.2 and pTa535 used in the identification of chromosome recombination in D88-2a may also be successfully hybridized on the chromosomes of rye (RR), *Leymus mollis* (NsNsXmXm), *Agropyron Gaertn* (PP) and *Thinopyrum elongatum* (EE) [34, 64]. However, the distribution of fluorescence signals on chromosomes of different species is different, which provides a basis for the determination of homoeologous group attribution and variation of chromosomes [65, 66]. The wheat 6D chromosome normally existed in D88-2a, which showed that different SNPs on wheat 6D reflected by SNP array were not because of the structure changes on short arm of the chromosome. It supported our speculation that the different SNPs on 6D chromosome were due to the nonspecific competitive binding between 6Ns and 6D to 15 K SNP array (see ahead paragraph). In addition, Because of the above collinear relationship, the different SNPs in nearly half of wheat 6D chromosomes were likely to be affected by sequences on half of the 6Ns chromosomes, which was verified the additional 6Ns chromosomes were incomplete.

Natural distant hybridization resulted in the aggregation of three sets of different genomes, which endowed the wheat genome with a strong buffer capacity, enabling it to accept exogenous genetic material [41, 67]. Although wild related species contain many excellent genes, breeding experience tells us that not all alien components are beneficial to recipient genome [68]. The addition of the *Aegilops comosa* 5 M chromosome significantly reduced the tillers of wheat, and the rye 1R chromosome improved wheat powdery mildew and rust resistance, yet sacrificed wheat flour processing quality [69, 70]. The ultimate purpose of germplasm innovation is to improve breeding rather than simply to obtain new materials, so we must pay attention to the agronomic traits of exogenously introgressed lines. In the previous study, Du et al. [26] reported a wheat-*P. huashanica* 6Ns addition line 59 − 11 which expressed special spike traits with top awn and multi-kernel clusters; Wang et al. [27] found a 6Ns addition line 25-10-3 with early-maturing trait; and Li et al. [71] proved that the 6Ns chromosome carries genes that improve the quality of wheat processing in the aspect of food chemistry. Although the materials were all 6Ns addition lines, they were developed from independent crossing making them have different characteristics. Therefore, the novel addition line D88-2a has great potential for improving cultivated wheat. In addition, we noted that 6Ns introgression lines generally exhibit paired spikelet characteristic. Paired spikelet forms a wheat inflorescence with more elaborate arrangement and increased number of grain producing spikelets, which therefore could be a strategy to improve yield potential of wheat [72]. As how the genes located in the additional 6Ns chromosome control these traits require further studies in the future.

## Conclusion

In this research, cell cytogenetics, multi-type molecular markers, and agronomic trait investigations were employed to analyse the chromosomal composition, homoeologous group of alien chromosomes and agronomic performance of line D88-2a. Our study showed that D88-2a is a novel wheat-*P. huashanica* 6Ns addition line with more tillers, better spike characteristics, higher stripe rust resistance and an increased kernel protein content. Therefore, line D88-2a can be an excellent germplasm for wheat yield-improvement and disease-resistance breeding programs.

### Electronic supplementary material

Below is the link to the electronic supplementary material.

Supplementary Material 1

Supplementary Material 2

Supplementary Material 3

## Data Availability

All related plant materials are available and comply Wild Plants Protection Regulation of China. The datasets supporting the conclusions of this article are included within the article and its supplementary files.

## Abbreviations

P. huashanica: Psathyrostachys huashanica Keng
GISH: Genomic in situ hybridization
FISH: Fluorescence in situ hybridization
PI: Propidium iodide
SNP: Single-nucleotide polymorphism
HXH: Huixianhong
CTAB: Cetyltrimethylammonium bromide
PMC: Pollen mother cell
RTC: Root tip cell
ND-FISH: Nondenaturing fluorescence in situ hybridization
SCAR: Specific sequence characterized amplified region
PCR: Polymerase chain reaction
STS: Sequence tag site
gDNA: Genomic DNA
A-PAGE: Acid-polyacrylamide gel electrophoresis
Pst: Puccinia striiformis f. sp. tritici
ΔC*: Colour saturation
ITs: Infection types
AVG: Average
