# Supplementary material for: The addition of Psathyrostachys Huashanica Keng 6Ns large segment chromosomes has positive impact on stripe rust resistance and plant spikelet number of common wheat
Source: BMC Plant Biol. 2024 Jul 18;24:685. doi: 10.1186/s12870-024-05395-9 (PMC11256485; doi:10.1186/s12870-024-05395-9)
Supplement: Supplementary file 1 — Supplementary Material 1 [file 12870_2024_5395_MOESM1_ESM.pdf]

Table S1 Detailed information of molecular markers used in this research.

Marker	Type	Chromosome	Left Primer	Right Primer
BE591325	STS	1AL 1BL	CTATCCAAGAGCCTTGCTGG	GCCAGTGTACAAATGCAACG
BF473108	STS	1AL 1BL 1DL	CGTTCACTCCCATTTTCTCC	AAGGCGTAGTGCTGCTTCAT
BE498745	STS	1AL 1BL 1DL	TCCCTTCCTGCAAGTTCTTC	CCCTCCATATCCATTGGTTG
BE498984	STS	1AL 1BL 1DL	CTGCTGGTGACCGGAAGTAT	AGGAGCGAATATGGCAGTTG
BE442562	STS	1AL 1BL 1DL	TGAACCAGCGTGTACCCATA	CCCTATAATACCGCGAGCAG
BF478395	STS	1AL 1BL 1DL	AGCAGGGGAAAGCCAGTTAT	CAATAACCGAGCCAGTCCAT
BE490064	STS	1BL 1DL	GCAGGAGCAAGGAGATCAAG	GCTTCCAACACACGGTTTTT
BE518358	STS	1BL 1DL	TCTTGCATCAAAGTGCTTGC	TGCACTTGCTGAGTCCATTC
BG262560	STS	1BL 2BS	AGGGGTTTGAGAAATGGACC	GTTTATGAGCTGGAGTCGCC
BF201480	STS	1BS 1A 1DS	CGTTTGCATCCCTTCTTGAT	AGCAAGTCCTTCAAGCCAAA
BE637715	STS	1BS 1DS	CAGGATTCAGCTACTTCGCC	GAAGAAGGTGATGCGGTAGG
BF475149	STS	1DL	GAGCAGCAAGAACCAGGAAC	TGTGAGAGCTCCAAAACCAA
BG604540	STS	1DL 1A	ATCACAAGACAGGTGGAGGC	GCTGGAGGTGTAGCCATGAT
BE406516	STS	1DL 1AS 1BS	ATGCTGGCACTGGCTTTACT	TTGAAGGGAAACGAGTCACC
BG607523	STS	1DL 2B	AAGGAAATTGAAACCCGCTT	ACCTCGTGTAAATTGGTGCC

BF484270	STS	2A 2BL 2DL	AGCAGCTGGTGCTTTACCTC	GGGTGAAAGTGCAGGGTAGA
BE604760	STS	2A 2DS	GGTTCTTGGCACAAGAGAGG	AAGGTGCCAATTTGGTTGAG
BE426620	STS	2AL	TGTGGTGGGGAAGACTAACC	CGGACAATATGGTTGCAGTG
BE403352	STS	2AL	ATGCAAAGCTGGCTGCTAAT	GCTCAAGTTGCTTCGGATTC
BF200628	STS	2AL	TGACCTACCTAGCCCCACTG	CGCGTAGTAGATCCACAGCA
BG605368	STS	2AL 1AL 2BL 2DL	GGGTGAAAGACCTCACCGT	CTGTGCTGATATGGGTGTCG
BE497955	STS	2AL 2BL	ATGCCCATAATTTTCGTTGC	CTATCGTCATAACCCGGTGC
BF482335	STS	2AL 2BL	TGCTCTCCTGCTTGTTTTCC	TGGTAAAAGAGCTCATGCTGG
BG274911	STS	2AL 2BL	ATGCCCACAAGTGCCTAAAC	AGAATCGGCTCATTTGATGG
BG274118	STS	2AL 2BL	AGGTGGGCTGGTACGTCCT	CCTTCTTTGTGACCCCAGAA
BE423738	STS	2AL 2BL 2DL	TGGAATGGAGCGATAGAAGG	TCAGCATGCAACACTTTGGT
BE425566	STS	2AL 2BL 2DL	ACGGCTTCACCGTCACTATC	CGCGAGGAGGTAGTTGAGAC
BE497569	STS	2AL 2BL 2DL	TGGGATTGGTTAGCACTTGA	TGGGCAAGTCTTTCTTGCAT
BE497814	STS	2AL 2BL 2DL	GTGGACATGCCTCTCAACAG	CTGCGTGGATAAGTAAGGGC
BG605024	STS	2BS 2DS	TCTGAGAACTCGCAAACTGC	TTAGTTCCTCGGGGCCAT
BE585687	STS	2D	ACCAAAGGGGAGAGCACTTT	TGGGAATCCGAATGATTTTC
BG262836	STS	2D	ATGCCTCTTTGGTAACCACG	CATGAGAATGTGTTCCACGG
BE500817	STS	2DL	CCACCAGGCATTGTCCTC	CGTTGAGCACCACGTTCAT
BE591243	STS	2DL	TTCTTTGGGCCTGCTTAGAA	TGTTGCACTTCTCTCATCCG

BE489611	STS	2DS	CATGATCCGCGAGATACTCA	GCATGACCATATGCTTGCAC
BG604453	STS	3AL 3BL 3DL	AAGCAGTTTGATACTGTTGGTGA	CGTCATGGTAAGGAGTGCCT
BE497979	STS	3AL 3BL 3DL	CAGCCAACCATACTCTCAAGC	GCATGATAACCCTCTTGACCA
BE591575	STS	3AL 3BL 3DL	CGAATTGCCAAACTCCATCT	CCTTCTCCCGACATTTGTGT
BF145691	STS	3AL 3BL 3DL	AACGTCTACGCGCTCAAGAT	TGGGAGGACTTTGATTGGAG
BF473348	STS	3AS 3BS 3DS	TGCTCATCTCCTTCCTCTGC	AGAGGGACGATGTTCCTGTG
BE498786	STS	3AS 3BS 3DS	TCATGGTGGTGGGATAGTCA	CGACATGGGTAACGAGGACT
BE405496	STS	3AS 3BS 3DS	CTTCCCATGGTCAGCAGAAT	ATTAGGGCCTTCAACTGGGT
BF293133	STS	3AS 3BS 3DS	AAGGTGAAGCTGTTCATGGG	GTAGAGCGTGATCACCGTGG
BG314551	STS	3AS 3D	GAAAAACACCGGGGTCCTAT	TATCCGAGCTGTGTGGAACA
BG274146	STS	3AS 3DS	GACCTCCAAGTGGACAATCC	TGCCATCTTACAGGAAAGCAA
BE496144	STS	3BL 3DL	AGGGGCTCAAGGAGATCGT	CCTTTTTCTTATCGCGCTTG
BE495175	STS	3BL 3DL	GTGATGCGCTTGAGAAGTCA	TTGGCTATCTGTCCCTGGTC
BF478559	STS	3BL 3DL	CAGCTTCTCCCCTTCCTCC	GAGGGTGTTGAGGGTCTTGA
BF474270	STS	3BL 5DL	TTTGCGATATTGTGGATGGA	AGAGTTGCTGTACGCTGGGT
BE494921	STS	3BS	CCTTCCTTGATCACATGGCT	TTTCCCAGCAAAGATGAACC
BF202635	STS	3BS	GGCGTGATCTTATTGGCATT	TCCGCAAATTTGTGTGATGT
BF291720	STS	3BS 3DS	AAGCCTTAATGCGTGCAAAG	GTGACAACACGCGTCTCACT
BF484268	STS	3BS 3DS	CGCAGGTCATAACAATGGTG	TTGCTCTCACCATCACCAAG

BG314507	STS	3D	ACCTGGAGATCCTCAGGGAG	CAACGCTGTATTCCCTTGGT
BE490085	STS	3DL	GTCATCTGCAACCTCGTCAC	ATTGCATCAGCATCATCAGC
BM138210	STS	3DL	CAGTCGCTCCCAAGAATGAT	AAGTGTGGCGTTCCTTTGAG
BE404377	STS	4AL	TGCTCAAGATCTGTCTGGGA	CTCCCCTCGGACTAGATGAA
BE517802	STS	4AL	ATAAGCAAGGAGACGGTGGA	GGGATTGGGCTTTTTGACTT
BE591563	STS	4AL	ACCCGTCCGAGTCACACTAC	CTTGATCTCCTCCAAGTCCG
BE404977	STS	4AL 4BS 4DS	AAGCAGCTGAGAAGCGTAGG	TTGCGTACAATCAGAAACGC
BF483640	STS	4AL 4BS 4DS	AGTGAGGAGATGCACAACCC	TAGCCAAAACCAGCTCCAGT
BF291753	STS	4AL 4DS	TCTACCCGGACCACAAGTTC	AAAAGCACCTTTTCCACGAA
BG605577	STS	4AL 4DS	GCCTGCAGATCTTCTCCATC	CCTGAAGCAAGATAGCGTCC
BF200966	STS	4AS	TCGATCCTGCCCTGTTATTC	GAGCCAAAAAGGCTTTACCC
BF202511	STS	4AS	TTCTGGTGTTGTGAAGGCAG	CTTGAATCGAAGGGTCTTGG
BQ281133	STS	4DL	ACATGCTAGGCAGGAGGCTA	GTGACGTTAGCTGCTTGCTG
BF292484	STS	4DL 4AS	GGACTCAGTTCAGGACAGCC	TGAGCTCAAAACCATTGCTG
BE495011	STS	4DL 5AL	GAATTTCATCTCACTCGCGG	TAAGCTGCTGAACCGTTCCT
BE490079	STS	5AL	CTCTCGGAGCGAGACTTCC	TACCAAGACCAATCTTGCCC
BG262917	STS	5AS 5BS	GTCATTTGCTTTGAGGCTGA	GCAAGGGCATTTGAGGAGTA
BE495282	STS	5AS 5BS	CAGACACCACACAAAGTGGG	CTGCAGTTGCATTCTCAGGA
BE591461	STS	5AS 5BS 5DS	GGCCGAGTAAAGAAGAGGCT	ATACCAGCGCCAGTTTCATC

BF202632	STS	5AS 5BS 5DS	TCAGCACATTGCCAGAAGTC	GTGATGACAGGTCACGTTGG
BQ294783	STS	5AS 5BS 5DS	GCAATTACCACAGCAAGGGT	CAGAATGATGACATCGGCAC
BF473352	STS	5BL	ATCCAACAGGGATGTGAAGG	GATAATGCGACCATCCCTTG
BE442810	STS	5BL	GGATGGTATGCAAATCCTGG	ATGAGCTCCCGGGTAAAAAT
BF202353	STS	5BL	AAGCGACAAGCAACTACGCT	AGCTTTCGAAACTGGGGAAG
BE495479	STS	5BL 5DL 5AL	ACTGGATGGCACCTGAAGTC	CCTCCTGATACTGCTGGCTC
BE591512	STS	5BL 5DL 5AL	AGGAAGCATAGCCCATTTGA	ATGCTCATCCCCAAGAACAC
BF145701	STS	5BL 5DL 5AL	ATCAGCTTCCTCCTCAGCAA	ATCCCCATCTTCCTGAGCTT
BF291755	STS	5BL 5DL 5AL	TTGGGCTTGAGGATGCTACT	ATGGCAACCGTCTTCAGAAC
BF146177	STS	5BS	TTTATTTGTGCCACCGTTGA	TCCGACTCTGATGCGTAGTG
BG263504	STS	5D 5B 5AS	GCACATGCTGTTCTACGGTC	TCACCACATTAATTCCCCGT
BE494817	STS	5DL	AAGGCCTCCATCACCGTC	AGGCACCTTACCTTCTGGGT
BF202667	STS	5DL	TGCATTGCTTCGGTCTACTG	AACTTTTTCGAGCCGCTGTA
BF473224	STS	5DL 5AL	GGGTACATTTCGGAAAGCAA	AGGTCACGGTGGATGATAGC
BE500894	STS	5DL 5AL	TACGTACAACCCGGAGGAAG	TTGAGCTGCTCTGTGCTGAT
BE591380	STS	5DL 5AL	GATGCACGGGCTGTTCTAAT	CATGGACTTCATCCATTGTCA
BQ282249	STS	5DL 5AS	GCCGCATGACCAAGAGTATT	CGGTTCACCATTTACCAAGG
BE604721	STS	6A 6BL	ACAGCAACTCAAGCAGCTCA	CAAGATGACCACCCTCTGGT
BE591367	STS	6AL 6BL	CTTCTATTGGATTTGGGGCA	ATTCTTTGGCTCGGTGAGAA

BF483267	STS	6AL 6BL	TGTTGCATCAGCTTCCTTTG	CTTCGCTTTGCAACTTTCCT
BE497701	STS	6AL 6BL	CCAGTTGAGTTTGGGTCGTT	CAGGTTTCATTCCCATTGCT
BG312637	STS	6AS 6BL	AATGATGCTTTGTTCCCCAA	CTTGGTATATCAGGATCACAGAA
BE426362	STS	6AS 6BS	TCGGACAAGATGAAGAAGCC	CGCGATGTATTTCTCCACG
BG274266	STS	6AS 6BS	ATCTTGGATGTCGGTTGGAA	GCTAGCCGCTCATCAGAGTT
MWG652	STS	6AS 6BS	GAGCTGCTCGTTCTCGTTGA	CACACCTTCTTCTTCCTCTT
BM138368	STS	6D	CATGGATGACGACGGTGC	ACTTGGCCTTCTCGACTTTG
BE497290	STS	6DL	GGGGATGATTGACGGTTCTA	AGATTTGCTTTGATGGGTGC
BF473299	STS	6DL	GGAAACTGTGAGACCGGAAA	TGGAAATAAGCAGACTGCCC
TC249514	STS	6DL	GCCAGGTCAAGGAGGGGAAAG	CGCCGAATCATACCGAATCATC
BG263549	STS	6DL	CTCTTCTTCATGCTCCTGCC	GCATGAATCCAACAAGGTCC
BF478945	STS	6DL 6AL 6BL	CTCGTGGGGTATAATGTGGC	TGGGGATTTGATCTTTCAGG
BG313802	STS	6DL 6AL 6BL	TTCATTTGCATCAAAGCAGC	GCGTAAGGCCCTGTGTGTAT
BF482566	STS	6DL 6AL 6BL	GACCCTGGGAGTGACAAGAA	TTTCAAAAGAAGAGGGCACC
BF482982	STS	6DL 6AL 6BL	TGACCAGCAGAAACAAGTGC	CGAGTTAAAGGTCGCGTAGC
CD452568	STS	6DL 6AL 6BL	TTTGCATTTTCGTCTGCAAG	TCGACACGAGCAAGATTCAC
BM134597	STS	6DL 6BL	AAGGAGATGGCTCAGGTTCA	AAAAGTATGTCCTGCGCACC
BF483397	STS	7A 7BS	AACTCATGAAATTTTGGGCG	TCTGGTTTGGGTTTACCTCG
BG606853	STS	7A 7BS	AGCAGAGTATGCCATTTCAGTG	GCGTTCATTCTCGTGAGCTT

BF293371	STS	7A 7BS 7DS	AATGCATTTAAGCCAGCACC	GATTCCGCCAGTTTATCGAA
BF482692	STS	7A 7BS 7DS	AGATGGCAGTGGTGTGTCTG	TCTCTATCTCTGCCGTTCCC
BF428868	STS	7AL	CTGCGGGCTTCTTCATCTAC	TGGTTGGTTGTTGCTGTGAT
BF293596	STS	7AL	CCGTCTGCTACAAGACCTTCA	CTTCCGGTACTGCGACCC
BF200911	STS	7AS	TTTCATCAACTTAAGCTGTATCCG	ACCTGCTGACGAGCAGCC
BE493868	STS	7AS 7BS 7DS	AGGTCGCCAGTACATGGAAG	TGCACAAAGGATCTGCTCAC
BE446380	STS	7AS 7BS 7DS	GTTTGCATCCTTTTCAGGGA	AACATCCAGCCAAGTATCCG
BF201628	STS	7AS 7BS 7DS	GTTTTGCTGCTGTTGCTCTG	CCAGCTGCTTTCACTTTGGT
BE403192	STS	7BS	ACTCCACGAAGCCCTCCTAC	TTGTTGTTGACGGTGTTGTG
BM134479	STS	7BS	CATCCAGGGTTGTGAGGAAT	TGAATAGGCACATCCACCAA
BE494123	STS	7BS 7DS	GAGCACCGAGTTCTCCTTCA	GTGCCCCACGAGGAAGTT
BE518447	STS	7DL	GACGTTGCCGTTGATCACTA	ATACTTGGGAAGATGCGTGC
BF201318	STS	7DL 7AL	GGATTGGTCTGAGGGGAAAT	TGGACTCTTTGATCCGTTCC
BE490023	STS	7DL 7AL 7BL	AACGCCACACTTATTTTGCC	CCATTTCTTCAATGCCAGGT
RHS141	SCAR	Ns	CTCGGCACCATAAACTAT	CTCGGCACTAGAGGAAAC
RHS23	SCAR	Ns	ACGCAGGCACGTTCTGATGACTACT	ACGCAGGCACCAAATAACAATTATT
RHS153	SCAR	1Ns	ACTGCCCGACGTAGACCAACTCTGC	ACTGCCCGACCCCACACACATATTG
SHS10	SCAR	1Ns	ATCGAGGACGACATGAAGGTGAT	TTAAGTTGCTGCCAATGTTCCAA
S131	SCAR	2Ns	CAACCTGCCTAACTATGTCA	CGACTTGGGGAGAAGGCTG

s3-125	SCAR	3Ns	GGTGACGAGGGTGTTGGATG	AGTGAACCGCATGGGTCTTT
s3-113	SCAR	3Ns	CGAATTGGATTGGCAGAGGGA	ACGATCTCCCTACGAATTGCA
RHS7	SCAR	5Ns	TGAGCGGACAGTTAACGGGC	TGAGCGGACATGTGGTGCAT
RHS14	SCAR	5Ns	ACTGGGACTCCAATGGGAAT	ACTGGGACTCGGGGTGTATT
RHS103	SCAR	5Ns	TCCGCATACCCGATAAT	TCCGCATACCACCTGTT
5NSR1	SCAR	5Ns	TGAGATTCGAGCATAGTTATC	CTTGGTGTGAACTATGCTCTTG
5NSR4	SCAR	5Ns	CAATATTTGTACTGTGAAGCC	CTTGGTGTGAACTATGCTCTTG
5NSR5	SCAR	5Ns	AGGTTGAATTTAGTGGTTGTG	CTTGGTGTGAACTATGCTCTTG
5NSR7	SCAR	5Ns	TGAGATTCGAGCATAGTTATC	GAGAGTGGTAAGCTCATGAGT
5NSR11	SCAR	5Ns	AGGTTGAATTTAGTGGTTGTG	GAGAGTGGTAAGCTCATGAGT
5NSR12	SCAR	5Ns	CTGATGCTCTATTGTGTGCTC	GAGAGTGGTAAGCTCATGAGT
5NSR14	SCAR	5Ns	ACTGTCTCCTGGCTTCACTC	AGAATACGGATTGCATTTCTG
