# Supplementary material for: The addition of Psathyrostachys Huashanica Keng 6Ns large segment chromosomes has positive impact on stripe rust resistance and plant spikelet number of common wheat
Source: BMC Plant Biol. 2024 Jul 18;24:685. doi: 10.1186/s12870-024-05395-9 (PMC11256485; doi:10.1186/s12870-024-05395-9)
Supplement: Supplementary file 2 — Supplementary Material 2 [file 12870_2024_5395_MOESM2_ESM.pdf]

Table S2 The genotype of 15K SNP array of D88-2a and its parents

probeset id	D88-2a	*P.huashanica*	7182	Affy SNP ID	Chr id	Start
AX-108730532	C/C	C/G	C/C	Affx-109255894	1A	547884554
AX-108732062	C/C	C/G	C/C	Affx-111872338	1A	104240003
AX-108733288	G/G	A/G	G/G	Affx-109775754	1A	31891661
AX-108756719	G/G	A/G	G/G	Affx-111944442	1A	480155150
AX-108761163	G/G	A/G	G/G	Affx-111929640	1A	139210422
AX-108762805	T/T	T/C	T/T	Affx-110555724	1A	424801366
AX-108766125	T/T	T/C	C/C	Affx-111132656	1A	502860219
AX-108766638	T/T	T/C	T/T	Affx-111286378	1A	377003739
AX-108773096	C/C	T/C	C/C	Affx-110296680	1A	257567903
AX-108775719	A/A	A/C	A/A	Affx-110052856	1A	223879216
AX-108777399	T/T	T/G	G/G	Affx-109015361	1A	10579460
AX-108779844	G/G	A/G	A/A	Affx-110953449	1A	9980666
AX-108782177	C/C	C/C	C/C	Affx-110317348	1A	240948016
AX-108789891	G/G	A/G	G/G	Affx-109998815	1A	155021453
AX-108791013	A/A	A/G	A/A	Affx-108953270	1A	308390322
AX-108795741	C/C	T/C	C/C	Affx-110037975	1A	388525167
AX-108807975	C/C	T/C	C/C	Affx-109679446	1A	565398182
AX-108808989	C/C	A/C	C/C	Affx-109918121	1A	551165346
AX-108814201	A/G	A/A	A/G	Affx-110659594	1A	305404584
AX-108815180	T/T	C/C	T/T	Affx-111423031	1A	40554914
AX-108818720	C/C	C/G	C/C	Affx-111085776	1A	570863125
AX-108820427	A/A	A/G	G/G	Affx-111190494	1A	28664400
AX-108821529	T/T	C/C	C/C	Affx-109287049	1A	31182614
AX-108828312	T/T	C/C	C/C	Affx-111186464	1A	492611518
AX-108830701	A/A	A/C	A/A	Affx-109594363	1A	322955974
AX-108835010	G/G	A/G	G/G	Affx-111893369	1A	49964748
AX-108841071	G/G	A/G	G/G	Affx-108987018	1A	327272366
AX-108844691	C/C	T/C	C/C	Affx-109106295	1A	343660821
AX-108844886	C/C	T/C	C/C	Affx-110634045	1A	520122965
AX-108852569	T/T	T/C	T/T	Affx-109362504	1A	339100657
AX-108858579	G/G	A/G	G/G	Affx-111059727	1A	340701630
AX-108859495	C/C	T/C	C/C	Affx-109573089	1A	318191337
AX-108865536	C/C	T/C	C/C	Affx-111270997	1A	388941546
AX-108866416	A/A	A/G	A/G	Affx-109087067	1A	20917708
AX-108877432	C/C	T/C	T/T	Affx-111569647	1A	30571380
AX-108881024	T/T	T/C	C/C	Affx-111714345	1A	510758911
AX-108881072	A/A	A/G	A/A	Affx-109726209	1A	344948322
AX-108882591	C/C	C/C	C/C	Affx-108903701	1A	440495263
AX-108888974	C/C	C/C	T/T	Affx-111007803	1A	41076253
AX-108894116	G/G	A/G	G/G	Affx-108924421	1A	191031702
AX-108896257	C/C	T/C	C/C	Affx-109528462	1A	28201863
AX-108907198	C/C	T/C	C/C	Affx-110560711	1A	168704407
AX-108914803	G/G	A/G	G/G	Affx-88639660	1A	574481294
AX-108916488	C/C	A/C	C/C	Affx-109315850	1A	235779078
AX-108917420	G/G	A/G	G/G	Affx-111090900	1A	485173993
AX-108921683	T/T	T/C	T/T	Affx-111458004	1A	349351057
AX-108929659	G/G	A/G	G/G	Affx-110023080	1A	277976628
AX-108931576	C/C	T/C	C/C	Affx-109297978	1A	522152681
AX-108935155	C/C	T/C	C/C	Affx-111756885	1A	549673052
AX-108943724	T/T	T/C	T/T	Affx-109661940	1A	367051284
AX-108948975	T/T	T/C	T/T	Affx-88560787	1A	259062806

AX-108964531	T/T	T/T	T/T	Affx-111640078	1A	309000814
AX-108966198	G/G	G/G	T/T	Affx-111372686	1A	16038997
AX-108973273	A/A	A/G	A/A	Affx-108930614	1A	201449191
AX-108974862	C/C	T/C	C/C	Affx-110611898	1A	145703714
AX-108988313	G/G	A/G	A/A	Affx-111604063	1A	47806425
AX-108999030	T/T	T/C	T/T	Affx-110983513	1A	147683716
AX-109004702	G/G	A/G	G/G	Affx-110537729	1A	531059828
AX-109041305	A/A	A/C	A/A	Affx-88758619	1A	151413441
AX-109043743	T/T	T/T	T/T	Affx-88610734	1A	143291868
AX-109044183	G/G	A/G	G/G	Affx-88493019	1A	474300128
AX-109044271	A/A	A/G	A/A	Affx-111245228	1A	164405062
AX-109044272	G/G	A/A	G/G	Affx-111365378	1A	285475581
AX-109048694	T/T	T/T	C/C	Affx-109602321	1A	514336708
AX-109052393	G/G	C/G	G/G	Affx-111061573	1A	207300487
AX-109054331	G/G	A/G	G/G	Affx-109239281	1A	302218996
AX-109083206	A/A	A/G	A/A	Affx-111574758	1A	134458307
AX-109102857	G/G	G/G	G/G	Affx-110079851	1A	225207125
AX-109277966	G/G	C/G	G/G	Affx-109250414	1A	439411878
AX-109289579	G/G	A/G	G/G	Affx-111653638	1A	155530744
AX-109291490	C/C	A/C	A/A	Affx-111317422	1A	504991472
AX-109296950	C/C	C/G	C/C	Affx-111930728	1A	320431994
AX-109299553	T/T	T/C	T/T	Affx-111080500	1A	571928598
AX-109301570	C/C	C/C	C/C	Affx-110297928	1A	202102183
AX-109302537	T/T	T/T	C/C	Affx-111347076	1A	99867205
AX-109304452	G/G	A/G	G/G	Affx-109162189	1A	343384533
AX-109311143	G/G	A/G	A/A	Affx-110974716	1A	32744633
AX-109314921	C/C	T/C	C/C	Affx-110862388	1A	11563697
AX-109325332	C/C	T/C	C/C	Affx-110187197	1A	381272347
AX-109326208	G/G	A/G	G/G	Affx-109704563	1A	101107864
AX-109326813	C/C	T/C	T/T	Affx-110427548	1A	513859613
AX-109328513	G/G	A/G	G/G	Affx-109107868	1A	371984854
AX-109329254	G/G	G/G	G/G	Affx-88497906	1A	461047793
AX-109333149	C/C	T/C	T/T	Affx-111865548	1A	462796333
AX-109335959	C/C	T/C	C/C	Affx-111930537	1A	295353416
AX-109340279	T/T	T/C	T/T	Affx-109752117	1A	40280825
AX-109340495	T/T	T/C	T/T	Affx-110240823	1A	584583724
AX-109341225	C/C	T/C	C/C	Affx-111668762	1A	567371662
AX-109344712	T/T	T/C	T/T	Affx-109631271	1A	14627346
AX-109351766	A/A	A/C	A/A	Affx-110550762	1A	331519295
AX-109353936	G/G	C/G	G/G	Affx-109903778	1A	220533715
AX-109356080	T/T	T/G	T/T	Affx-111669336	1A	568417998
AX-109356451	C/C	A/C	C/C	Affx-111836318	1A	301204340
AX-109356616	T/T	C/C	T/T	Affx-111435526	1A	151877811
AX-109360059	G/G	G/G	G/G	Affx-109826857	1A	277079262
AX-109363171	A/A	C/C	A/A	Affx-111065160	1A	423724359
AX-109363703	G/G	A/G	G/G	Affx-109610397	1A	158927428
AX-109368678	T/T	T/C	T/T	Affx-110903838	1A	375012155
AX-109373066	A/A	A/G	G/G	Affx-108972626	1A	32295651
AX-109374083	T/T	T/C	T/T	Affx-111724194	1A	166611635
AX-109374169	G/G	A/G	G/G	Affx-111208892	1A	391247594
AX-109374200	G/G	A/G	G/G	Affx-111221163	1A	143777932
AX-109375456	T/T	T/C	C/C	Affx-108858982	1A	496145066
AX-109382288	T/T	T/C	T/T	Affx-109690119	1A	341591187
AX-109391306	C/C	A/C	C/C	Affx-111092862	1A	170576953

AX-109396457	G/G	A/G	G/G	Affx-111585612	1A	220546917
AX-109397393	C/C	C/C	C/C	Affx-109980714	1A	237508877
AX-109403945	G/G	T/G	G/G	Affx-109472701	1A	144799711
AX-109404355	G/G	A/G	G/G	Affx-109122086	1A	373201260
AX-109408386	T/T	T/C	T/T	Affx-110599323	1A	532065433
AX-109412526	A/A	A/G	A/A	Affx-109941175	1A	194931207
AX-109415183	G/G	A/G	G/G	Affx-111533015	1A	562322390
AX-109421148	A/A	A/G	A/A	Affx-111858014	1A	291236722
AX-109423270	T/T	T/C	T/T	Affx-111132570	1A	358154622
AX-109423347	T/T	T/C	T/T	Affx-88526834	1A	557487909
AX-109425297	C/C	T/T	C/C	Affx-110945598	1A	523030643
AX-109428041	T/T	T/C	T/T	Affx-109345091	1A	22483490
AX-109441476	A/A	A/G	A/A	Affx-110352732	1A	257716824
AX-109442898	A/A	A/G	A/A	Affx-110727311	1A	359028019
AX-109447158	T/T	T/C	T/T	Affx-111357409	1A	488648697
AX-109454533	G/G	C/G	G/G	Affx-111796651	1A	519263551
AX-109456415	A/A	A/G	A/A	Affx-109649308	1A	518444344
AX-109456940	T/T	C/C	C/C	Affx-110375395	1A	576905100
AX-109465131	G/G	A/A	A/A	Affx-111978690	1A	512367109
AX-109469627	G/G	A/G	G/G	Affx-110419629	1A	488409378
AX-109471276	T/T	T/C	T/T	Affx-108883810	1A	581874194
AX-109473499	G/G	A/G	A/A	Affx-109053188	1A	33403197
AX-109482870	C/C	T/C	C/C	Affx-88555379	1A	384821889
AX-109483444	T/T	T/C	T/T	Affx-109884448	1A	557056493
AX-109483727	G/G	A/G	G/G	Affx-111923010	1A	39640209
AX-109487391	T/T	T/C	T/T	Affx-109544578	1A	185016615
AX-109488577	C/C	C/C	C/C	Affx-110494097	1A	361450560
AX-109494616	A/A	A/G	A/A	Affx-111433059	1A	296216227
AX-109517045	A/A	A/G	A/A	Affx-111904825	1A	309423337
AX-109528407	G/G	G/G	G/G	Affx-109599127	1A	497194286
AX-109562522	G/G	A/G	G/G	Affx-109127468	1A	360009501
AX-109585289	C/C	T/C	C/C	Affx-110499981	1A	173056779
AX-109597085	G/G	T/G	G/G	Affx-111237700	1A	569977069
AX-109606033	A/A	A/C	A/A	Affx-110212173	1A	352873176
AX-109619286	C/C	C/G	C/C	Affx-110938930	1A	390708666
AX-109621044	T/T	T/G	T/T	Affx-110301330	1A	589697812
AX-109623840	T/T	T/C	T/T	Affx-110665650	1A	56486064
AX-109624394	A/A	A/G	A/A	Affx-110455815	1A	262176611
AX-109627395	C/C	C/C	C/C	Affx-110951952	1A	474795403
AX-109628778	G/G	A/G	G/G	Affx-110019292	1A	338609216
AX-109630836	A/A	A/G	A/A	Affx-110483871	1A	188833953
AX-109634424	G/G	A/G	G/G	Affx-111441608	1A	550789727
AX-109635515	C/C	T/C	C/C	Affx-109589641	1A	296409028
AX-109816635	T/T	T/T	T/T	Affx-110227920	1A	580439377
AX-109819145	C/C	A/C	C/C	Affx-110088321	1A	298067643
AX-109820251	C/C	T/C	C/C	Affx-111114795	1A	7363621
AX-109822252	A/G	A/G	A/G	Affx-109606321	1A	588691268
AX-109824940	T/T	T/C	T/T	Affx-109111748	1A	262939765
AX-109831764	C/C	C/G	C/C	Affx-109690153	1A	487725293
AX-109841934	C/C	T/C	C/C	Affx-111126255	1A	74429616
AX-109842766	C/C	T/C	C/C	Affx-109605680	1A	477737419
AX-109848072	C/C	T/C	C/C	Affx-111336400	1A	315390754
AX-109849380	A/A	A/C	A/A	Affx-109664906	1A	224806146
AX-109849752	A/A	A/C	A/A	Affx-109490510	1A	535716169

AX-109855969	T/T	G/G	T/T	Affx-109793586	1A	575224835
AX-109859174	C/C	C/C	C/G	Affx-109373659	1A	7671749
AX-109862316	A/A	A/G	A/A	Affx-110922220	1A	54687790
AX-109863129	T/T	T/G	T/T	Affx-108957280	1A	532673880
AX-109863151	G/G	A/G	G/G	Affx-110508087	1A	382033563
AX-109863848	C/C	T/C	C/C	Affx-109153363	1A	540413542
AX-109864205	C/C	T/C	C/C	Affx-110674350	1A	30276357
AX-109864216	G/G	A/G	G/G	Affx-109398068	1A	483131050
AX-109866949	T/T	T/C	T/T	Affx-88770497	1A	553705997
AX-109867513	C/C	T/T	T/T	Affx-109388940	1A	493820290
AX-109875945	G/G	C/C	G/G	Affx-109780422	1A	324560834
AX-109886270	G/G	A/G	G/G	Affx-108936914	1A	344324226
AX-109887522	C/C	T/C	C/C	Affx-88413477	1A	365646107
AX-109902115	G/G	A/G	G/G	Affx-109757341	1A	357186179
AX-109904229	G/G	A/G	A/A	Affx-111002423	1A	516450384
AX-109905627	T/T	T/C	T/T	Affx-110233030	1A	402928251
AX-109909262	A/A	A/C	A/A	Affx-109789901	1A	351475106
AX-109911956	A/A	G/G	A/A	Affx-110974702	1A	50911068
AX-109914567	G/G	A/G	G/G	Affx-88563065	1A	539880228
AX-109916940	T/T	T/G	T/T	Affx-109522058	1A	506524807
AX-109927600	G/G	A/G	G/G	Affx-110770471	1A	273664590
AX-109930799	G/G	A/G	A/A	Affx-111130978	1A	464896790
AX-109935575	G/G	A/G	G/G	Affx-111333524	1A	531547546
AX-109937019	C/C	T/C	C/C	Affx-109333815	1A	525123787
AX-109938803	C/C	T/T	C/C	Affx-108914809	1A	529583616
AX-109954487	G/G	T/G	T/T	Affx-111284181	1A	43571899
AX-109967116	G/G	C/G	G/G	Affx-109552741	1A	334502147
AX-109968061	G/G	A/A	G/G	Affx-111079512	1A	555817271
AX-109969719	T/T	T/C	T/T	Affx-110913502	1A	9572902
AX-109971623	C/C	C/C	C/C	Affx-110779137	1A	549011020
AX-109972578	G/G	A/G	G/G	Affx-108882559	1A	394302994
AX-109974584	A/A	A/C	A/A	Affx-110265360	1A	197344946
AX-109977330	G/G	A/G	G/G	Affx-109243725	1A	337705896
AX-109983715	T/T	T/C	T/T	Affx-109396040	1A	137999776
AX-109984571	G/G	A/A	G/G	Affx-111399402	1A	190418873
AX-109984878	T/G	T/G	T/G	Affx-109700650	1A	484868422
AX-109985412	T/T	T/C	C/C	Affx-111708481	1A	513362330
AX-109987797	G/G	T/G	G/G	Affx-109622680	1A	587809580
AX-109989893	C/C	T/C	C/C	Affx-110769936	1A	127480729
AX-110005760	C/C	T/C	C/C	Affx-110279512	1A	254944329
AX-110006847	G/G	A/G	A/A	Affx-111757245	1A	48759326
AX-110012280	G/G	G/G	G/G	Affx-111019446	1A	553882841
AX-110014905	G/G	A/G	G/G	Affx-88407881	1A	560578516
AX-110018834	T/T	T/C	T/T	Affx-110583976	1A	337154946
AX-110019998	G/G	G/G	G/G	Affx-109468863	1A	186663085
AX-110023564	T/T	T/T	T/T	Affx-109430281	1A	528973058
AX-110026141	T/G	T/G	T/G	Affx-111293392	1A	524387425
AX-110026287	C/C	T/C	C/C	Affx-111413955	1A	374775771
AX-110032820	C/C	T/C	T/T	Affx-109405140	1A	471272117
AX-110035801	T/T	T/T	T/T	Affx-110125039	1A	371080282
AX-110044204	A/A	A/G	A/A	Affx-109292757	1A	177788012
AX-110049336	C/C	A/C	C/C	Affx-111403788	1A	218478571
AX-110049395	A/A	A/C	A/A	Affx-109172627	1A	424604028
AX-110062407	G/G	A/G	G/G	Affx-111752557	1A	404419268

AX-110084994	G/G	A/G	G/G	Affx-109451912	1A	539315190
AX-110089093	T/C	T/C	T/T	Affx-109871447	1A	460278088
AX-110090502	C/C	T/C	T/T	Affx-109275675	1A	16407055
AX-110094282	A/A	A/A	A/A	Affx-111956018	1A	255874119
AX-110122042	C/C	T/C	C/C	Affx-110804868	1A	528633639
AX-110173584	G/G	A/G	G/G	Affx-111126186	1A	219097774
AX-110176062	C/C	T/T	C/C	Affx-109386534	1A	181790435
AX-110181408	T/T	T/C	T/T	Affx-109834181	1A	24331546
AX-110367274	A/A	A/G	A/A	Affx-111165023	1A	241654628
AX-110368348	T/T	T/G	T/T	Affx-109542046	1A	311846987
AX-110377259	C/C	T/C	T/T	Affx-108931939	1A	563308643
AX-110377941	C/C	A/C	C/C	Affx-109065519	1A	160986441
AX-110378928	T/T	T/G	T/T	Affx-110536790	1A	544594992
AX-110384221	T/T	T/C	T/T	Affx-109593873	1A	306103781
AX-110390222	G/G	A/G	G/G	Affx-110488525	1A	560095842
AX-110394989	C/C	T/C	C/C	Affx-108946489	1A	582655804
AX-110395979	C/C	C/C	C/C	Affx-109156011	1A	518505178
AX-110396848	T/T	T/C	T/T	Affx-109509057	1A	229691779
AX-110396896	T/T	T/C	T/T	Affx-108966767	1A	5491723
AX-110397170	C/C	T/T	C/C	Affx-110159861	1A	224218968
AX-110398363	T/T	T/C	T/T	Affx-111712995	1A	356388090
AX-110402839	A/A	G/G	A/A	Affx-111707927	1A	162652894
AX-110402962	T/T	T/G	T/T	Affx-110632945	1A	162480445
AX-110408363	C/C	C/C	C/C	Affx-111454246	1A	435671058
AX-110412867	G/G	G/G	G/G	Affx-88639511	1A	208887667
AX-110421810	G/G	A/G	G/G	Affx-111891906	1A	255302725
AX-110427428	T/T	T/C	T/T	Affx-109212504	1A	486322263
AX-110433295	G/G	G/G	G/G	Affx-109678871	1A	92564383
AX-110434112	G/G	C/G	G/G	Affx-111240187	1A	313121935
AX-110441785	T/T	T/G	T/T	Affx-108915297	1A	209473521
AX-110450556	G/G	C/C	G/G	Affx-109600146	1A	319886708
AX-110455706	T/T	T/C	T/T	Affx-111073497	1A	270133145
AX-110455726	T/T	T/G	T/T	Affx-110638171	1A	259046191
AX-110463408	A/A	A/G	G/G	Affx-111552153	1A	494722975
AX-110464527	T/C	T/C	T/C	Affx-109511694	1A	538235443
AX-110464570	G/G	A/G	G/G	Affx-111177705	1A	51678756
AX-110471563	C/C	C/G	C/C	Affx-110264216	1A	317922045
AX-110471753	G/G	A/G	G/G	Affx-110879612	1A	555951314
AX-110475253	G/G	A/G	G/G	Affx-110689799	1A	331414199
AX-110480896	T/T	T/G	T/T	Affx-109268144	1A	379139661
AX-110489045	A/A	A/G	A/A	Affx-111392731	1A	365489201
AX-110493799	A/A	A/G	A/A	Affx-110311829	1A	295034616
AX-110499703	A/A	A/G	A/A	Affx-111626998	1A	312527773
AX-110506787	T/T	T/G	T/T	Affx-109569336	1A	339794635
AX-110507437	T/T	C/C	T/T	Affx-110870601	1A	435735594
AX-110511731	C/C	T/C	C/C	Affx-110877269	1A	334788278
AX-110516805	A/A	C/C	A/A	Affx-88402135	1A	352141066
AX-110523831	C/C	C/C	A/C	Affx-111255380	1A	15187341
AX-110525765	A/A	G/G	A/A	Affx-109324138	1A	256704709
AX-110540561	C/C	T/C	C/C	Affx-88404536	1A	99140580
AX-110547230	G/G	A/G	G/G	Affx-88778334	1A	355318778
AX-110548582	C/C	T/C	C/C	Affx-110529460	1A	438540535
AX-110562440	A/A	A/G	A/A	Affx-88632214	1A	537353589
AX-110562636	C/C	C/G	C/C	Affx-110437546	1A	110074030

AX-110567183	A/A	A/G	A/A	Affx-111759976	1A	336460977
AX-110574139	C/C	T/C	C/C	Affx-109946641	1A	118502958
AX-110576015	A/A	A/A	A/A	Affx-110016512	1A	157515267
AX-110592831	A/A	A/G	A/A	Affx-88409385	1A	236723044
AX-110593666	T/T	T/G	T/T	Affx-110506143	1A	339916340
AX-110605358	T/T	T/G	T/T	Affx-110381919	1A	34143926
AX-110610111	G/G	G/G	G/G	Affx-109953548	1A	12087652
AX-110611419	C/C	T/C	T/T	Affx-111116120	1A	514695443
AX-110616388	T/T	T/C	T/T	Affx-109210567	1A	479539377
AX-110616913	T/T	T/C	T/T	Affx-111201579	1A	267153822
AX-110617203	G/G	A/G	G/G	Affx-109075281	1A	368710195
AX-110628967	T/T	T/C	T/T	Affx-109380391	1A	389803509
AX-110636090	T/T	T/C	T/T	Affx-109615474	1A	579869577
AX-110642950	G/G	A/G	G/G	Affx-111699218	1A	471503530
AX-110661013	C/C	T/C	C/C	Affx-109232429	1A	147357383
AX-110670478	T/T	T/C	T/T	Affx-111603601	1A	92469528
AX-110697418	A/A	A/G	A/A	Affx-110832238	1A	290340831
AX-110712805	A/A	A/G	A/A	Affx-110516358	1A	437227053
AX-110714545	C/C	C/G	C/C	Affx-108901445	1A	35837799
AX-110732157	G/G	T/G	G/G	Affx-110771772	1A	237217385
AX-110735773	G/G	A/G	G/G	Affx-109278118	1A	378395050
AX-110906010	A/G	A/G	A/G	Affx-111992429	1A	256932365
AX-110906954	T/C	T/C	C/C	Affx-88526215	1A	14161799
AX-110917923	C/C	T/C	C/C	Affx-110483294	1A	36388893
AX-110922919	G/G	G/G	G/G	Affx-111671206	1A	317714960
AX-110926621	T/T	T/G	T/T	Affx-110591755	1A	475204083
AX-110931640	A/A	A/C	A/A	Affx-111087094	1A	311844480
AX-110937281	T/T	T/T	T/T	Affx-88743101	1A	519626949
AX-110943762	T/T	T/C	T/T	Affx-111774755	1A	297883939
AX-110944731	G/G	A/G	G/G	Affx-108980579	1A	173664017
AX-110945242	C/C	C/G	C/C	Affx-111146796	1A	53659451
AX-110947388	G/G	A/G	G/G	Affx-110035437	1A	336126282
AX-110955950	C/C	A/C	C/C	Affx-110545239	1A	371524922
AX-110960234	T/T	T/C	T/T	Affx-111631854	1A	51962196
AX-110971755	C/C	C/C	C/C	Affx-110716118	1A	49565802
AX-110977845	C/C	A/C	C/C	Affx-111969732	1A	316151009
AX-110981297	T/T	T/C	T/T	Affx-109811037	1A	527095488
AX-110987235	T/T	T/G	T/T	Affx-109987076	1A	584755997
AX-110990436	A/A	A/C	A/A	Affx-111554734	1A	349517298
AX-110991760	T/T	C/C	C/C	Affx-111225188	1A	491140000
AX-110997190	C/C	C/C	C/C	Affx-110557971	1A	227474419
AX-111001034	C/C	T/C	C/C	Affx-110436910	1A	23014071
AX-111003139	A/A	A/G	A/A	Affx-110777102	1A	314438579
AX-111014967	G/G	A/G	G/G	Affx-111310337	1A	125151717
AX-111017764	C/C	C/C	C/C	Affx-110344408	1A	18048549
AX-111019464	G/G	G/G	G/G	Affx-109588025	1A	25888886
AX-111026946	T/T	T/C	T/T	Affx-110039096	1A	377609033
AX-111026966	C/C	T/C	C/C	Affx-110018906	1A	182324857
AX-111031072	G/G	T/G	G/G	Affx-110735678	1A	486035384
AX-111032242	G/G	A/G	A/A	Affx-111777100	1A	493217690
AX-111034164	G/G	T/G	G/G	Affx-110650497	1A	316861419
AX-111039702	C/C	T/C	T/T	Affx-109454569	1A	35545627
AX-111042246	G/G	G/G	G/G	Affx-109025988	1A	584072596
AX-111043857	T/T	C/C	T/T	Affx-88564648	1A	542429785

AX-111044540	C/C	T/C	C/C	Affx-108869188	1A	149351101
AX-111052075	C/C	T/C	C/C	Affx-110023139	1A	355795442
AX-111057726	G/G	T/G	G/G	Affx-109496727	1A	486822254
AX-111069240	A/A	G/G	A/A	Affx-109696574	1A	483700527
AX-111074330	C/C	T/C	C/C	Affx-109278404	1A	152463525
AX-111084696	T/T	T/C	C/C	Affx-111425854	1A	16422919
AX-111087538	T/T	T/C	T/T	Affx-110752581	1A	556728551
AX-111087592	A/A	A/G	A/A	Affx-111919182	1A	239135392
AX-111095246	A/A	A/T	A/A	Affx-109009470	1A	155701938
AX-111099153	G/G	A/G	G/G	Affx-109558809	1A	259670557
AX-111101445	G/G	A/G	G/G	Affx-109629067	1A	152814599
AX-111107679	C/C	T/C	C/C	Affx-111196181	1A	476083843
AX-111110976	C/C	T/C	C/C	Affx-110694129	1A	249112392
AX-111118849	C/C	T/C	C/C	Affx-110237424	1A	124498173
AX-111120968	T/T	T/G	T/T	Affx-111498501	1A	138430520
AX-111125144	C/C	T/C	T/T	Affx-110588575	1A	18487694
AX-111133220	T/T	T/G	T/T	Affx-109891770	1A	304849708
AX-111135025	C/C	T/C	C/C	Affx-110675403	1A	220980745
AX-111138197	T/T	T/C	T/T	Affx-109662823	1A	359564585
AX-111140587	A/A	A/G	A/A	Affx-110211228	1A	323844310
AX-111151160	C/C	C/C	C/C	Affx-110423600	1A	507777512
AX-111156568	T/T	A/T	T/T	Affx-110355190	1A	548573715
AX-111158522	T/T	T/C	T/T	Affx-110797784	1A	477934572
AX-111163383	A/A	A/A	A/A	Affx-109254874	1A	389822414
AX-111168439	C/C	C/G	C/C	Affx-88726929	1A	261825583
AX-111176006	T/T	T/G	T/T	Affx-110640418	1A	34531442
AX-111211653	C/G	C/G	C/G	Affx-110136035	1A	593408912
AX-111213190	A/A	A/A	A/A	Affx-111740427	1A	183231144
AX-111213581	T/C	T/T	T/C	Affx-111941243	1A	282876706
AX-111214633	C/C	T/C	C/C	Affx-111847329	1A	434177041
AX-111214792	G/G	A/G	G/G	Affx-111282213	1A	174051655
AX-111217732	T/T	C/C	T/C	Affx-111827494	1A	360999748
AX-111218514	G/G	A/G	G/G	Affx-109743273	1A	314744543
AX-111221462	T/T	T/C	T/T	Affx-111868084	1A	251689066
AX-111222852	C/C	C/G	C/C	Affx-109158300	1A	472544847
AX-111223739	C/C	A/C	C/C	Affx-110421771	1A	267881911
AX-111241096	T/T	T/C	T/T	Affx-110511167	1A	261413231
AX-111242393	A/A	A/G	A/A	Affx-111460676	1A	300712672
AX-111252231	T/T	T/C	T/T	Affx-110807713	1A	273153221
AX-111260564	C/C	T/C	C/C	Affx-110201548	1A	126782346
AX-111262089	T/T	T/C	T/T	Affx-110686693	1A	8727315
AX-111263532	A/A	A/G	A/A	Affx-111905166	1A	188477525
AX-111283111	C/C	C/G	C/C	Affx-110827671	1A	310143397
AX-111450961	G/G	A/G	G/G	Affx-88777403	1A	546502829
AX-111452243	A/A	A/G	A/A	Affx-111835716	1A	193688543
AX-111452805	C/C	A/C	C/C	Affx-111666768	1A	532935325
AX-111459247	C/C	A/C	C/C	Affx-111553188	1A	303347851
AX-111460918	G/G	A/A	A/A	Affx-88494265	1A	45856341
AX-111471771	G/G	C/G	C/G	Affx-111345640	1A	586132124
AX-111472392	A/A	A/G	A/A	Affx-108903719	1A	306615654
AX-111480833	G/G	A/G	G/G	Affx-111787334	1A	266082338
AX-111481601	A/A	A/G	A/A	Affx-111102663	1A	318905179
AX-111482811	G/G	A/G	G/G	Affx-110918097	1A	280713178
AX-111485169	G/G	T/G	G/G	Affx-109859861	1A	534849354

AX-111489802	T/T	T/G	T/T	Affx-111408611	1A	570580119
AX-111493911	G/G	G/G	G/G	Affx-110401347	1A	480567254
AX-111494269	G/G	A/G	G/G	Affx-109380102	1A	432558642
AX-111498487	T/T	T/T	T/T	Affx-110652221	1A	141020492
AX-111505633	G/G	A/G	G/G	Affx-110168969	1A	2876821
AX-111509307	G/G	A/G	G/G	Affx-88757592	1A	320445166
AX-111517864	T/T	T/C	T/T	Affx-88609893	1A	562154455
AX-111525529	T/T	T/C	C/C	Affx-88682088	1A	577820374
AX-111527056	C/C	T/C	C/C	Affx-109921424	1A	146255755
AX-111533945	A/A	A/A	A/A	Affx-110467736	1A	160058772
AX-111534622	C/C	C/C	C/C	Affx-109564133	1A	272106056
AX-111535469	A/A	A/G	A/A	Affx-110104321	1A	175866156
AX-111545796	G/G	G/G	G/G	Affx-110515781	1A	484293014
AX-111546934	A/A	A/G	A/A	Affx-109261797	1A	354054598
AX-111549611	A/A	A/G	A/A	Affx-110467078	1A	124789204
AX-111554031	C/C	T/C	C/C	Affx-110630120	1A	319435790
AX-111563037	C/C	A/C	C/C	Affx-110066186	1A	481233771
AX-111570965	G/G	A/G	G/G	Affx-108857529	1A	115431406
AX-111572655	T/T	T/C	T/T	Affx-109899335	1A	324607963
AX-111575061	A/A	A/G	A/A	Affx-110341558	1A	566372095
AX-111576317	T/T	T/C	T/T	Affx-109086578	1A	479030432
AX-111578749	T/T	A/T	A/A	Affx-108871620	1A	453766229
AX-111585289	G/G	A/G	G/G	Affx-110061590	1A	399231378
AX-111586048	C/C	T/C	T/C	Affx-110246877	1A	388156180
AX-111591884	C/C	C/G	G/G	Affx-110963577	1A	501343140
AX-111592472	G/G	A/G	G/G	Affx-111115815	1A	204098555
AX-111597054	A/A	A/G	A/A	Affx-111107346	1A	395367267
AX-111604707	C/C	T/C	C/C	Affx-110412837	1A	3226874
AX-111609101	T/T	T/C	T/T	Affx-111004673	1A	310539740
AX-111620737	C/C	T/C	C/C	Affx-110969930	1A	313595383
AX-111623149	C/C	T/C	C/C	Affx-111666594	1A	202069079
AX-111625323	C/C	C/C	C/C	Affx-108959795	1A	179526726
AX-111629871	G/G	A/A	A/A	Affx-88779235	1A	439929997
AX-111635974	G/G	G/G	C/C	Affx-109482824	1A	22042494
AX-111645533	C/C	T/C	C/C	Affx-109765347	1A	246571909
AX-111647350	C/C	T/C	C/C	Affx-109012966	1A	113865421
AX-111650098	C/C	T/C	C/C	Affx-111937254	1A	252279835
AX-111650792	A/A	A/A	A/A	Affx-109846484	1A	327168664
AX-111651188	G/G	A/G	G/G	Affx-109432388	1A	1348742
AX-111651445	G/G	A/G	G/G	Affx-111331227	1A	53436220
AX-111652012	T/T	T/T	T/T	Affx-110319331	1A	333072539
AX-111652275	C/C	T/C	C/C	Affx-108928115	1A	551979324
AX-111652927	A/A	A/C	A/A	Affx-110389746	1A	207944805
AX-111654904	A/A	A/A	A/A	Affx-111659636	1A	438884607
AX-111655605	C/C	C/G	C/C	Affx-109259372	1A	158195238
AX-111655790	T/T	T/C	T/T	Affx-88700917	1A	550611274
AX-111663855	C/C	T/C	C/C	Affx-109334854	1A	157627426
AX-111681900	G/G	A/G	A/A	Affx-110832356	1A	577313956
AX-111682431	T/T	T/C	T/T	Affx-109086208	1A	395311070
AX-111686031	G/G	C/G	G/G	Affx-88559376	1A	517077768
AX-111688135	G/G	A/G	G/G	Affx-109180868	1A	543480297
AX-111689506	G/G	A/G	G/G	Affx-111926073	1A	573642575
AX-111692232	C/C	A/C	A/A	Affx-111934592	1A	46967025
AX-111696669	G/G	G/G	C/G	Affx-109950992	1A	500871013

AX-111700010	A/A	A/G	A/A	Affx-109279324	1A	242531846
AX-111700821	T/T	T/C	C/C	Affx-111230832	1A	498667313
AX-111702167	T/T	T/G	T/T	Affx-109083924	1A	547028458
AX-111719607	C/C	T/C	C/C	Affx-110831427	1A	302640298
AX-111730844	G/G	C/G	G/G	Affx-109066211	1A	582860342
AX-111736507	A/A	A/G	A/A	Affx-88614067	1A	101770717
AX-111738110	C/C	T/C	T/T	Affx-110605935	1A	34876707
AX-111753200	C/C	C/C	C/C	Affx-88571605	1A	511502177
AX-111760906	C/C	A/C	C/C	Affx-110864147	1A	117817956
AX-111762593	C/C	T/C	T/T	Affx-111299690	1A	39167273
AX-111764127	G/G	A/G	G/G	Affx-110325718	1A	544497666
AX-111764359	C/C	T/C	C/C	Affx-110170534	1A	172354229
AX-111767357	G/G	A/G	G/G	Affx-108906798	1A	495125499
AX-111769036	T/T	T/T	T/T	Affx-111840074	1A	38380669
AX-111775939	G/G	A/G	G/G	Affx-111856897	1A	304177126
AX-111802924	C/C	T/C	T/T	Affx-110496423	1A	504003811
AX-111803078	T/T	T/C	C/C	Affx-111585799	1A	492023119
AX-111806559	A/G	A/G	A/G	Affx-111247679	1A	449836382
AX-111827505	T/T	T/T	C/C	Affx-109772173	1A	507223542
AX-111827953	G/G	G/G	G/G	Affx-111731925	1A	184391658
AX-112286628	A/A	A/G	A/A	Affx-88784533	1A	588767571
AX-179388397	G/G	G/G	G/G	Affx-92903632	1A	7294614
AX-182056099	G/G	A/G	G/G	Affx-472298449	1A	508961809
AX-86164646	C/C	T/C	T/T	Affx-88788576	1A	500980417
AX-86167093	C/C	T/C	T/T	Affx-88592114	1A	498811359
AX-86170121	C/C	T/C	T/T	Affx-93016140	1A	54126886
AX-86172394	T/T	T/C	C/C	Affx-88532510	1A	513736378
AX-86177236	G/G	G/G	G/G	Affx-92625004	1A	28757527
AX-86184285	A/A	A/C	A/A	Affx-92314418	1A	428546913
AX-89424853	G/G	A/G	G/G	Affx-88457519	1A	267158347
AX-89475672	A/A	A/G	A/A	Affx-88508500	1A	347633555
AX-89488696	C/C	A/C	C/C	Affx-88521558	1A	399495179
AX-89542937	C/C	T/C	C/C	Affx-110477843	1A	23966223
AX-89650136	T/T	T/C	T/T	Affx-92155216	1A	511102083
AX-89713082	G/G	A/G	G/G	Affx-88745790	1A	554536304
AX-89714190	A/A	A/G	A/G	Affx-88746897	1A	504577414
AX-89732204	T/T	T/C	C/C	Affx-88764896	1A	498141736
AX-89738551	G/G	A/G	A/A	Affx-110839688	1A	575684543
AX-89759492	A/A	A/A	A/A	Affx-88790406	1A	580961021
AX-94399869	A/A	A/G	A/A	Affx-92740311	1A	272316170
AX-94404141	A/G	A/A	A/G	Affx-88685162	1A	354823513
AX-94416982	T/T	T/C	T/T	Affx-92159466	1A	47578129
AX-94426657	A/A	G/G	A/A	Affx-92462303	1A	521323272
AX-94438518	C/C	T/C	T/T	Affx-92326031	1A	394593229
AX-94439447	A/A	A/C	A/A	Affx-92272647	1A	292077819
AX-94503000	C/C	T/C	C/C	Affx-92240794	1A	529789404
AX-94531333	G/G	A/G	G/G	Affx-92826367	1A	380262435
AX-94583334	A/A	A/G	G/G	Affx-92893704	1A	490414377
AX-94593042	C/C	C/C	C/C	Affx-92396935	1A	86648367
AX-94615199	G/G	C/G	G/G	Affx-92837116	1A	21624343
AX-94621604	C/C	C/G	C/C	Affx-92177028	1A	375475180
AX-94633764	T/C	T/T	T/C	Affx-92230563	1A	412458142
AX-94636216	C/C	T/C	C/C	Affx-92703575	1A	382249851
AX-94673150	G/G	A/G	G/G	Affx-92771019	1A	101380403

AX-94705481	C/C	C/G	G/G	Affx-92876124	1A	269581046
AX-94727697	G/G	A/G	G/G	Affx-92373575	1A	41709840
AX-94771670	C/C	T/C	C/C	Affx-92932276	1A	300104076
AX-94774041	T/T	T/T	T/T	Affx-92715783	1A	552950675
AX-94823954	A/A	A/A	A/A	Affx-92753277	1A	326213838
AX-94874566	G/G	G/G	G/G	Affx-92248094	1A	513662178
AX-94897165	C/G	G/G	C/G	Affx-92597414	1A	149510418
AX-95117204	T/T	T/C	T/T	Affx-92113385	1A	522516048
AX-95182518	C/C	C/C	C/C	Affx-92329488	1A	279832345
AX-95226098	A/A	A/G	A/A	Affx-92238485	1A	544054340
AX-95232658	T/C	C/C	T/C	Affx-92243342	1A	161671443
AX-95235150	C/C	C/G	C/C	Affx-92533717	1A	366130370
AX-95255804	C/C	G/G	C/C	Affx-92408787	1A	572350913
AX-95630373	C/C	A/C	C/C	Affx-92697490	1A	74443548
AX-95654060	C/C	A/A	A/A	Affx-88430657	1A	46238736
AX-95658304	G/G	A/G	G/G	Affx-88539804	1A	8296948
AX-95658884	T/T	T/G	T/T	Affx-88643759	1A	353255356
AX-95660980	T/G	T/T	T/G	Affx-109553700	1A	224826935
AX-95684530	G/G	A/G	G/G	Affx-88619338	1A	264983336
AX-108725476	G/G	A/G	A/A	Affx-111421322	1B	451575800
AX-108729731	A/A	A/G	G/G	Affx-109381105	1B	558736312
AX-108733712	C/C	T/T	C/C	Affx-109383417	1B	675569717
AX-108734756	A/A	A/G	A/A	Affx-109759718	1B	465245191
AX-108737720	C/C	C/G	C/C	Affx-110695426	1B	686751506
AX-108738808	G/G	T/G	T/T	Affx-111021861	1B	411177595
AX-108740171	A/A	A/G	A/A	Affx-110081630	1B	334292409
AX-108740187	A/A	A/G	A/A	Affx-88461096	1B	688119096
AX-108741139	G/G	A/G	A/A	Affx-108869253	1B	290203821
AX-108743970	A/A	A/G	G/G	Affx-111544310	1B	597951229
AX-108746762	A/A	A/G	A/A	Affx-110926572	1B	83945482
AX-108746927	T/C	T/C	C/C	Affx-108896129	1B	443330131
AX-108747070	A/A	A/G	A/A	Affx-109444541	1B	668895773
AX-108747601	T/T	T/C	C/C	Affx-111963526	1B	395959962
AX-108747782	C/C	C/G	C/C	Affx-108969168	1B	664070972
AX-108750914	T/T	C/C	T/T	Affx-110492283	1B	49144412
AX-108756721	A/A	A/T	T/T	Affx-111599044	1B	384566103
AX-108774212	T/T	T/C	T/T	Affx-108900246	1B	42335615
AX-108780177	T/C	T/C	T/C	Affx-110822798	1B	5684596
AX-108782034	G/G	A/G	G/G	Affx-109887984	1B	665454538
AX-108786643	G/G	C/G	C/C	Affx-109591192	1B	447549679
AX-108787375	A/A	A/A	A/A	Affx-110955896	1B	670277306
AX-108788117	G/G	A/G	G/G	Affx-109296767	1B	654531498
AX-108788746	A/A	A/C	C/C	Affx-110331699	1B	264349973
AX-108790366	A/A	A/G	A/A	Affx-111168472	1B	458387930
AX-108790376	A/G	A/A	A/A	Affx-111879520	1B	345306249
AX-108791049	C/C	C/C	T/T	Affx-110918298	1B	488592723
AX-108796055	T/T	T/C	C/C	Affx-111809154	1B	450220999
AX-108798446	A/A	A/G	A/A	Affx-111882569	1B	42347523
AX-108798723	A/A	A/G	G/G	Affx-109202444	1B	322940238
AX-108800295	C/C	T/C	T/T	Affx-111571111	1B	360784101
AX-108800962	G/G	A/G	G/G	Affx-111095039	1B	659361327
AX-108804866	C/C	C/G	C/C	Affx-111877080	1B	572254394
AX-108810207	C/C	T/C	T/T	Affx-109992453	1B	555941570
AX-108814725	A/A	A/C	A/A	Affx-109241936	1B	452763309

AX-108822210	C/C	T/C	C/C	Affx-108934928	1B	249857827
AX-108822833	T/T	T/G	T/T	Affx-109344142	1B	475537354
AX-108826064	A/A	A/G	G/G	Affx-109919627	1B	582119865
AX-108828473	T/T	T/T	C/C	Affx-109518457	1B	553550464
AX-108831096	G/G	A/A	A/A	Affx-109688278	1B	420568994
AX-108831740	T/T	T/C	T/T	Affx-88659781	1B	660015693
AX-108833740	C/C	C/C	C/C	Affx-110510129	1B	688629864
AX-108838990	G/G	G/G	G/G	Affx-88655950	1B	342122438
AX-108845851	C/C	C/G	G/G	Affx-110970484	1B	371140782
AX-108848761	A/A	A/G	A/A	Affx-110290968	1B	459191489
AX-108850301	A/A	A/C	A/A	Affx-109860068	1B	323946791
AX-108852469	G/G	A/G	G/G	Affx-110718632	1B	458005172
AX-108854385	T/T	T/T	C/C	Affx-109761267	1B	613548284
AX-108855359	T/T	T/C	C/C	Affx-110467347	1B	497981060
AX-108856070	G/G	A/G	G/G	Affx-111715590	1B	472691172
AX-108859471	G/G	A/G	G/G	Affx-110670628	1B	629073531
AX-108867872	A/A	A/G	A/A	Affx-111910401	1B	621538354
AX-108872900	T/T	T/C	C/C	Affx-109822957	1B	432495179
AX-108877371	T/T	T/C	C/C	Affx-111872003	1B	402337255
AX-108879322	T/T	T/T	T/C	Affx-111184790	1B	42308622
AX-108880548	G/G	C/G	G/G	Affx-109035452	1B	620463318
AX-108884580	A/A	A/G	G/G	Affx-111449855	1B	301565568
AX-108885690	C/C	T/C	T/T	Affx-109190542	1B	448540614
AX-108888480	C/C	T/C	T/T	Affx-109705276	1B	412794523
AX-108888595	A/A	A/C	C/C	Affx-109133202	1B	443566852
AX-108892781	G/G	T/G	G/G	Affx-109511824	1B	657274040
AX-108898290	A/A	A/G	A/A	Affx-108876347	1B	619587744
AX-108900436	A/G	G/G	G/G	Affx-110617340	1B	312504174
AX-108904194	T/T	T/C	C/C	Affx-109250363	1B	577031581
AX-108905424	C/C	T/C	T/T	Affx-110065849	1B	266879283
AX-108909153	C/C	C/G	G/G	Affx-88585787	1B	356984891
AX-108912443	G/G	A/G	A/A	Affx-110626555	1B	300602293
AX-108915846	A/A	A/G	G/G	Affx-111662221	1B	508709125
AX-108920629	C/C	C/G	G/G	Affx-109277393	1B	604095613
AX-108920917	T/T	T/G	T/G	Affx-111558989	1B	511050019
AX-108921800	T/C	T/C	C/C	Affx-111103484	1B	522726318
AX-108928321	A/A	A/A	G/G	Affx-109649345	1B	540990519
AX-108948156	G/G	A/G	G/G	Affx-110600491	1B	549417777
AX-108959675	G/G	G/G	A/G	Affx-111434201	1B	398788024
AX-108960559	T/G	G/G	G/G	Affx-111122292	1B	303863408
AX-108964423	T/T	T/T	C/C	Affx-110056307	1B	450603208
AX-108966369	G/G	G/G	G/G	Affx-88445029	1B	1254928
AX-108972329	T/T	A/T	A/A	Affx-111055446	1B	298437645
AX-108973265	G/G	G/G	A/A	Affx-111582580	1B	381032751
AX-108973449	G/G	C/G	G/G	Affx-88696333	1B	643446112
AX-108977901	A/A	A/G	G/G	Affx-110613895	1B	602754305
AX-108979502	T/T	C/C	C/C	Affx-110904238	1B	305170319
AX-108987264	C/C	T/C	C/C	Affx-110878786	1B	336787649
AX-108995348	G/G	A/A	A/A	Affx-110985338	1B	357649901
AX-109007995	T/T	T/C	C/C	Affx-111111942	1B	393081496
AX-109020320	G/G	A/G	A/A	Affx-111968554	1B	341520526
AX-109032009	C/C	T/C	T/T	Affx-110501038	1B	535231000
AX-109037093	C/C	C/G	C/C	Affx-110318706	1B	602547367
AX-109037969	G/G	G/G	A/G	Affx-110289101	1B	292197988

AX-109043016	C/C	A/C	A/A	Affx-109201871	1B	538461468
AX-109057464	C/C	A/A	C/C	Affx-111845473	1B	465733224
AX-109070579	C/C	T/C	T/T	Affx-110229557	1B	379583836
AX-109076514	C/C	C/G	C/C	Affx-110036149	1B	222551307
AX-109109358	G/G	A/A	G/G	Affx-110478593	1B	655324737
AX-109271826	A/A	A/C	C/C	Affx-109075992	1B	380240446
AX-109272373	T/T	A/T	A/A	Affx-110480610	1B	404523501
AX-109273370	A/A	A/G	G/G	Affx-109523926	1B	303023060
AX-109273979	G/G	G/G	G/G	Affx-111776226	1B	482462544
AX-109276571	G/G	G/G	G/G	Affx-110064224	1B	686369997
AX-109276656	A/A	A/G	A/A	Affx-111428351	1B	618551525
AX-109281992	T/T	T/G	T/T	Affx-109670749	1B	119100832
AX-109287909	C/C	C/G	C/C	Affx-109313441	1B	644435004
AX-109292833	T/T	T/C	T/T	Affx-109353511	1B	547315290
AX-109296870	G/G	A/G	A/A	Affx-108901049	1B	592215341
AX-109297395	C/C	C/G	C/C	Affx-110380213	1B	547922552
AX-109299717	A/A	G/G	A/A	Affx-110567997	1B	670794681
AX-109301822	A/A	A/G	G/G	Affx-88789297	1B	433604952
AX-109308096	G/G	A/G	A/A	Affx-110694245	1B	289529400
AX-109310461	C/C	C/C	G/G	Affx-108894096	1B	588325146
AX-109314529	A/T	A/A	A/A	Affx-88589197	1B	299457722
AX-109322773	G/G	A/G	A/A	Affx-110931800	1B	328417586
AX-109326990	G/G	C/G	C/C	Affx-110347096	1B	466501248
AX-109334796	A/G	A/G	A/G	Affx-110612533	1B	561708663
AX-109338666	G/G	T/G	T/T	Affx-110933596	1B	363956736
AX-109340517	G/G	C/G	C/C	Affx-110116661	1B	386689382
AX-109343639	T/T	T/C	T/T	Affx-109172595	1B	629489850
AX-109353011	A/A	A/A	G/G	Affx-110697028	1B	685283930
AX-109356154	A/A	A/G	G/G	Affx-88647339	1B	310932331
AX-109363225	T/T	T/C	T/T	Affx-110255099	1B	649425062
AX-109366497	T/T	A/A	T/T	Affx-110412333	1B	658365851
AX-109368546	A/A	A/G	A/A	Affx-109316038	1B	591432791
AX-109370500	G/G	A/G	A/A	Affx-111341679	1B	304379381
AX-109372181	C/C	T/T	T/T	Affx-111854245	1B	322425833
AX-109376365	A/A	A/C	A/A	Affx-111237497	1B	666807428
AX-109383638	T/T	T/C	C/C	Affx-111600298	1B	295437309
AX-109387657	C/C	T/C	T/T	Affx-109579035	1B	372217456
AX-109390528	T/T	T/C	T/T	Affx-88368231	1B	6411551
AX-109391593	C/C	C/G	G/G	Affx-110736262	1B	387288895
AX-109395396	C/C	T/C	T/T	Affx-108901543	1B	494228418
AX-109404221	G/G	A/G	A/A	Affx-110421551	1B	258519594
AX-109404795	G/G	T/G	T/T	Affx-110736529	1B	415614587
AX-109409212	C/C	T/C	T/T	Affx-111390177	1B	680121566
AX-109412475	G/G	A/G	A/A	Affx-111492486	1B	539442158
AX-109419267	A/A	A/A	C/C	Affx-109916671	1B	384249145
AX-109421320	A/C	A/C	C/C	Affx-111860742	1B	431469851
AX-109425846	G/G	A/G	G/G	Affx-110693492	1B	473006805
AX-109435258	A/A	A/A	A/A	Affx-88365888	1B	42309866
AX-109436738	C/C	A/A	C/C	Affx-109825464	1B	645531213
AX-109437757	C/C	C/G	G/G	Affx-110580399	1B	366917565
AX-109437886	C/G	C/G	C/C	Affx-109075080	1B	536107730
AX-109439401	T/T	T/G	G/G	Affx-109358594	1B	575503316
AX-109441951	A/T	A/T	A/A	Affx-109479168	1B	6473048
AX-109444940	A/A	A/G	A/A	Affx-109834354	1B	627827969

AX-109445577	G/G	A/G	G/G	Affx-109350348	1B	477357647
AX-109447941	A/A	A/A	C/C	Affx-110687037	1B	401821047
AX-109452517	T/T	T/T	C/C	Affx-109883626	1B	483905019
AX-109456390	C/C	C/C	A/A	Affx-109979441	1B	533184345
AX-109457059	T/T	T/C	C/C	Affx-110151050	1B	511409502
AX-109463291	T/T	T/C	T/T	Affx-88545369	1B	581103597
AX-109464140	C/C	T/C	C/C	Affx-110374205	1B	222429935
AX-109474853	T/T	T/G	T/T	Affx-111063502	1B	685226111
AX-109474984	C/C	T/C	T/T	Affx-109541057	1B	362355575
AX-109486098	T/T	T/G	G/G	Affx-110324950	1B	332150785
AX-109490479	G/G	G/G	G/G	Affx-110858852	1B	603085800
AX-109494986	T/T	T/C	T/T	Affx-110876557	1B	662202621
AX-109508228	G/G	A/G	G/G	Affx-111581811	1B	338002884
AX-109512508	C/C	T/C	C/C	Affx-109675583	1B	485877903
AX-109517530	C/C	C/G	G/G	Affx-110041700	1B	616360840
AX-109523456	G/G	A/G	A/A	Affx-109835108	1B	403418894
AX-109527015	C/G	C/G	G/G	Affx-110598779	1B	492792651
AX-109539628	G/G	G/G	A/A	Affx-111061221	1B	486976002
AX-109544098	C/C	T/C	T/T	Affx-109840944	1B	377963460
AX-109545914	G/G	A/G	A/G	Affx-109537733	1B	25808018
AX-109580048	T/T	T/C	T/T	Affx-88358201	1B	455176306
AX-109580210	T/T	T/G	G/G	Affx-110513513	1B	375303546
AX-109581046	G/G	A/G	A/A	Affx-111320972	1B	370197585
AX-109583401	C/C	T/C	T/T	Affx-110663454	1B	359891254
AX-109604511	G/G	T/G	T/T	Affx-111947267	1B	588852762
AX-109617246	C/C	T/C	T/T	Affx-110210936	1B	297013252
AX-109622448	A/A	A/G	A/A	Affx-111821853	1B	605665523
AX-109652390	C/C	T/C	T/T	Affx-110140219	1B	481904145
AX-109821715	C/C	C/C	A/A	Affx-88726485	1B	574372661
AX-109824660	C/C	T/C	C/C	Affx-109429959	1B	675324456
AX-109840997	T/C	T/C	T/T	Affx-111554658	1B	347558393
AX-109842914	A/A	A/G	G/G	Affx-109029558	1B	686753896
AX-109846519	G/G	A/G	A/A	Affx-108884935	1B	269315308
AX-109847715	C/C	T/T	C/C	Affx-109098018	1B	686786607
AX-109853345	A/G	A/G	A/A	Affx-110604564	1B	378461864
AX-109857682	A/A	A/G	G/G	Affx-110129041	1B	296491871
AX-109859134	T/T	T/G	G/G	Affx-109979161	1B	536573814
AX-109859227	A/A	A/C	A/A	Affx-108914173	1B	629940450
AX-109859779	C/C	C/G	C/C	Affx-109037991	1B	42335249
AX-109862469	G/G	A/G	A/A	Affx-109648756	1B	324351688
AX-109873586	A/C	A/C	C/C	Affx-109543061	1B	389879147
AX-109878201	T/T	G/G	T/T	Affx-109967199	1B	563465954
AX-109881506	A/A	A/G	A/A	Affx-109236565	1B	277262905
AX-109882817	C/C	T/C	C/C	Affx-111036378	1B	476082407
AX-109882902	C/C	A/C	C/C	Affx-110606121	1B	549792880
AX-109885745	G/G	A/G	A/A	Affx-111161676	1B	529952411
AX-109886973	T/T	T/C	T/T	Affx-110958828	1B	429922330
AX-109887385	A/A	A/C	A/A	Affx-109052237	1B	460274388
AX-109888316	A/A	A/G	G/G	Affx-111492595	1B	413748062
AX-109892539	G/G	A/G	G/G	Affx-110461039	1B	470939843
AX-109900946	G/G	C/G	C/C	Affx-109668438	1B	309706909
AX-109903718	C/C	T/C	C/C	Affx-111931647	1B	564916918
AX-109907382	T/T	T/G	G/G	Affx-110542085	1B	344322309
AX-109910123	G/G	T/G	G/G	Affx-110588114	1B	223145363

AX-109916275	T/T	T/G	G/G	Affx-111855550	1B	575828993
AX-109920004	C/C	T/T	T/T	Affx-110367471	1B	405985965
AX-109920276	G/G	A/G	A/A	Affx-109376735	1B	491227521
AX-109924351	T/C	T/C	C/C	Affx-109739236	1B	682431511
AX-109929201	T/T	T/C	C/C	Affx-110966256	1B	527632183
AX-109931371	C/C	T/C	C/C	Affx-110339772	1B	441673027
AX-109932384	C/G	C/G	G/G	Affx-110631046	1B	374913451
AX-109946575	T/T	T/C	C/C	Affx-110768124	1B	307136332
AX-109947536	G/G	A/G	G/G	Affx-109081320	1B	222837178
AX-109948353	G/G	A/G	G/G	Affx-88703322	1B	571073013
AX-109956862	G/G	G/G	A/G	Affx-109802243	1B	6474849
AX-109961006	G/G	G/G	G/G	Affx-111100056	1B	679299190
AX-109966524	G/G	A/G	A/A	Affx-110021127	1B	512107159
AX-109971635	C/C	T/C	C/C	Affx-111790665	1B	632634933
AX-109971835	A/A	A/A	G/G	Affx-109357626	1B	401333244
AX-109980796	T/T	T/C	T/T	Affx-109113643	1B	581197863
AX-109980951	T/T	T/T	T/T	Affx-111202864	1B	467925765
AX-109983759	A/A	A/C	C/C	Affx-109653470	1B	383176594
AX-109997234	G/G	A/G	A/A	Affx-108884489	1B	481322282
AX-109997417	C/C	T/C	C/C	Affx-109193692	1B	474100622
AX-110002545	T/T	T/G	T/T	Affx-111306761	1B	667499368
AX-110002681	T/T	T/C	C/C	Affx-110948526	1B	391917068
AX-110011386	T/T	T/C	T/T	Affx-110513175	1B	640341564
AX-110020631	C/C	T/C	T/T	Affx-110480973	1B	322947409
AX-110023834	C/C	T/C	C/C	Affx-110263339	1B	267572715
AX-110029221	T/T	T/C	C/C	Affx-110382777	1B	486535039
AX-110041028	C/C	T/C	C/C	Affx-111677717	1B	252258452
AX-110052300	T/C	C/C	C/C	Affx-111163965	1B	299266246
AX-110057926	G/G	A/G	A/A	Affx-111759055	1B	407869097
AX-110062945	G/G	A/G	A/A	Affx-110843989	1B	387783486
AX-110063809	C/C	T/C	T/T	Affx-109804546	1B	293640276
AX-110076399	G/G	A/G	A/A	Affx-110177705	1B	483286941
AX-110091358	T/T	T/C	C/C	Affx-88628338	1B	577924275
AX-110094943	G/G	C/G	G/G	Affx-88722781	1B	456252134
AX-110121329	C/C	T/C	T/T	Affx-111677238	1B	388895243
AX-110121671	G/G	T/G	G/G	Affx-111893136	1B	459400879
AX-110124276	A/A	A/G	A/A	Affx-111431392	1B	464840844
AX-110125845	G/G	A/G	A/A	Affx-111050557	1B	434542934
AX-110128022	G/G	T/T	G/G	Affx-111761250	1B	660564756
AX-110134125	A/A	A/G	G/G	Affx-110210586	1B	431894597
AX-110151702	C/C	T/C	T/C	Affx-109484203	1B	422482291
AX-110167781	C/C	C/G	G/G	Affx-110929900	1B	346408189
AX-110169030	G/G	A/G	A/A	Affx-111887857	1B	418617796
AX-110190018	C/C	T/C	T/T	Affx-111181925	1B	399230670
AX-110195432	T/T	T/C	C/C	Affx-111436324	1B	525222630
AX-110200329	G/G	A/G	G/G	Affx-111372389	1B	477589529
AX-110359707	T/T	T/C	C/C	Affx-111300514	1B	685225141
AX-110361204	C/C	C/C	G/G	Affx-111893751	1B	446519377
AX-110364298	G/G	C/G	G/G	Affx-109695206	1B	661603601
AX-110365668	C/C	A/C	A/A	Affx-111236074	1B	443330272
AX-110365753	G/G	A/G	A/A	Affx-110499413	1B	251531124
AX-110368320	T/T	T/C	C/C	Affx-109531164	1B	311736328
AX-110368439	C/C	A/C	A/A	Affx-110273502	1B	419950198
AX-110369292	T/T	A/T	A/A	Affx-109340856	1B	315384262

AX-110378403	A/A	A/G	A/A	Affx-111105457	1B	336272947
AX-110386580	G/G	A/G	A/A	Affx-110858242	1B	372157887
AX-110389721	G/G	C/G	C/C	Affx-111862657	1B	587026825
AX-110390375	A/G	G/G	G/G	Affx-110835332	1B	555794665
AX-110392069	G/G	C/G	G/G	Affx-110069848	1B	639638582
AX-110402488	T/T	T/G	T/T	Affx-109740770	1B	580075805
AX-110403620	G/G	A/G	G/G	Affx-109996822	1B	667911274
AX-110406362	A/G	G/G	A/G	Affx-109591647	1B	330233699
AX-110407313	C/G	C/G	C/C	Affx-111729353	1B	664773810
AX-110411292	T/T	T/C	C/C	Affx-111396743	1B	494329346
AX-110419096	T/T	T/C	C/C	Affx-109647180	1B	290870245
AX-110419189	T/T	T/T	T/C	Affx-110940771	1B	418160680
AX-110420436	G/G	C/G	C/C	Affx-109660509	1B	624082112
AX-110428546	T/T	T/C	T/T	Affx-108870998	1B	473485052
AX-110434923	T/T	T/C	C/C	Affx-88662365	1B	551496829
AX-110437942	C/C	T/C	T/T	Affx-109230498	1B	399329113
AX-110439262	G/G	A/G	A/A	Affx-88474786	1B	308011295
AX-110458682	G/G	A/G	A/A	Affx-111369094	1B	6389544
AX-110464799	C/C	C/G	G/G	Affx-109889282	1B	615373735
AX-110468810	G/G	A/G	A/A	Affx-110124017	1B	302543411
AX-110469350	G/G	A/G	G/G	Affx-110164136	1B	630513611
AX-110474956	A/A	A/G	G/G	Affx-111647615	1B	369345717
AX-110477792	C/C	A/C	A/A	Affx-109276609	1B	258918059
AX-110479520	G/G	A/G	G/G	Affx-109578636	1B	470419525
AX-110480919	C/G	G/G	G/G	Affx-109691303	1B	363458923
AX-110483186	T/T	T/C	T/T	Affx-110633414	1B	470006417
AX-110483510	A/A	A/G	A/A	Affx-88654237	1B	583685696
AX-110489420	C/C	T/C	C/C	Affx-108965698	1B	663230030
AX-110491218	A/A	A/A	G/G	Affx-109881483	1B	416171678
AX-110494096	T/T	T/C	T/T	Affx-109269353	1B	504516171
AX-110501384	A/A	G/G	G/G	Affx-110851557	1B	33947872
AX-110501414	C/C	T/C	T/T	Affx-109372171	1B	505111854
AX-110506641	G/G	A/G	G/G	Affx-110873808	1B	653263871
AX-110508398	C/C	T/C	T/T	Affx-111511499	1B	445388556
AX-110513953	T/T	C/C	C/C	Affx-111331187	1B	414157717
AX-110514435	T/G	G/G	G/G	Affx-111807907	1B	251039124
AX-110517311	C/C	T/C	T/T	Affx-109728240	1B	361778280
AX-110519906	C/G	C/G	C/C	Affx-111175020	1B	552477160
AX-110523194	A/A	A/C	A/A	Affx-108955171	1B	545213643
AX-110524254	T/T	T/C	C/C	Affx-111355961	1B	493356115
AX-110524472	A/A	A/G	A/A	Affx-108990525	1B	340381039
AX-110528563	T/C	T/C	T/T	Affx-109546349	1B	254688842
AX-110531641	G/G	A/G	A/A	Affx-109989509	1B	365441304
AX-110535000	A/G	A/G	A/A	Affx-110095022	1B	280184833
AX-110538382	A/A	A/G	G/G	Affx-108910468	1B	594654251
AX-110539535	C/C	T/C	T/T	Affx-110481087	1B	306229922
AX-110546334	A/A	A/G	A/A	Affx-110140464	1B	456746687
AX-110552523	G/G	A/G	A/A	Affx-111665641	1B	311443328
AX-110552541	C/C	C/G	C/G	Affx-111338801	1B	293996773
AX-110553429	C/C	A/C	C/C	Affx-110288758	1B	542929558
AX-110557228	G/G	A/G	G/G	Affx-110818157	1B	641857414
AX-110559599	T/G	T/G	G/G	Affx-109208502	1B	551763800
AX-110565341	T/T	T/C	T/T	Affx-111930680	1B	591956667
AX-110571242	T/C	T/T	T/C	Affx-110689077	1B	566763085

AX-110573119	C/C	T/C	C/C	Affx-110458676	1B	468316769
AX-110575668	A/A	A/G	A/A	Affx-110984026	1B	620458755
AX-110581042	A/A	A/G	G/G	Affx-88348720	1B	637371064
AX-110581950	A/A	G/G	A/A	Affx-112001206	1B	654866336
AX-110585482	A/A	A/G	A/A	Affx-109451970	1B	656500596
AX-110590407	C/C	T/C	T/T	Affx-109058618	1B	449051020
AX-110598522	C/C	T/C	T/T	Affx-111814403	1B	544144588
AX-110598536	T/T	T/G	G/G	Affx-111579344	1B	509300586
AX-110600824	T/T	T/G	G/G	Affx-109891783	1B	278927057
AX-110601772	C/C	T/C	T/T	Affx-108952636	1B	313527562
AX-110603923	C/C	T/C	C/C	Affx-109047748	1B	530597303
AX-110618463	G/G	A/G	A/A	Affx-110682367	1B	558086532
AX-110623229	C/C	C/G	G/G	Affx-108981454	1B	369515449
AX-110641985	C/C	T/C	C/C	Affx-109385689	1B	630923053
AX-110670988	T/T	T/C	C/C	Affx-110091425	1B	489973360
AX-110676933	C/C	A/C	A/A	Affx-109613100	1B	412236309
AX-110687238	C/C	T/C	T/T	Affx-110045392	1B	448131731
AX-110689665	G/G	A/G	A/A	Affx-109266107	1B	372748785
AX-110735575	A/A	A/G	A/A	Affx-111903830	1B	525076350
AX-110736518	G/G	C/G	G/G	Affx-111150815	1B	644991383
AX-110737060	C/C	T/T	T/T	Affx-111635670	1B	413387645
AX-110740030	C/C	T/C	T/T	Affx-111359916	1B	373856428
AX-110906363	T/T	T/T	C/C	Affx-88379848	1B	168622406
AX-110906777	G/G	T/G	G/G	Affx-109137850	1B	631154284
AX-110908967	C/C	C/G	C/C	Affx-111137520	1B	550499883
AX-110916356	A/A	A/G	G/G	Affx-109293409	1B	489129788
AX-110920101	G/G	G/G	C/C	Affx-109076724	1B	593918322
AX-110922932	G/G	T/G	G/G	Affx-110632045	1B	570569224
AX-110924487	A/A	A/C	A/A	Affx-110477414	1B	343245050
AX-110926323	C/C	T/C	C/C	Affx-109966667	1B	325256496
AX-110934854	C/C	C/G	G/G	Affx-110881156	1B	297978096
AX-110938801	T/T	C/C	C/C	Affx-109765624	1B	344591605
AX-110941446	T/T	T/C	T/T	Affx-110658900	1B	648035023
AX-110947408	A/A	G/G	A/A	Affx-111177277	1B	658924414
AX-110948037	G/G	C/G	G/G	Affx-110118321	1B	641491265
AX-110961208	A/A	A/G	G/G	Affx-109766841	1B	573261850
AX-110972752	C/C	C/G	G/G	Affx-88762083	1B	266270602
AX-110974122	C/C	T/C	T/T	Affx-88783294	1B	577610016
AX-110976797	G/G	C/G	C/C	Affx-109825064	1B	595632410
AX-110979003	G/G	A/G	G/G	Affx-109333541	1B	642429411
AX-110989601	A/A	G/G	G/G	Affx-110627176	1B	573759887
AX-110990078	A/A	A/G	A/A	Affx-111510441	1B	666089546
AX-110994035	A/A	A/G	A/A	Affx-109052606	1B	250986312
AX-110994863	T/T	T/C	C/C	Affx-109858317	1B	5510525
AX-111001918	C/C	C/G	C/C	Affx-109896115	1B	627355270
AX-111007632	G/G	C/G	C/C	Affx-109805780	1B	410299329
AX-111009896	C/C	T/C	C/C	Affx-109495874	1B	569506918
AX-111011493	C/C	T/C	C/C	Affx-110611716	1B	328989594
AX-111022842	G/G	A/A	A/A	Affx-88782495	1B	389321645
AX-111029725	C/G	C/G	G/G	Affx-111988558	1B	370369876
AX-111036581	T/T	T/C	T/T	Affx-109295341	1B	662192117
AX-111044282	T/T	T/C	T/T	Affx-109505320	1B	548888538
AX-111047964	C/G	C/C	C/C	Affx-111128428	1B	313050757
AX-111051682	G/G	A/G	G/G	Affx-110851466	1B	335879496

AX-111065451	C/G	G/G	G/G	Affx-111486179	1B	555941640
AX-111077672	C/C	T/C	T/T	Affx-110920751	1B	415335518
AX-111078071	G/G	A/G	G/G	Affx-110019881	1B	601735612
AX-111082456	T/T	T/C	T/T	Affx-111972897	1B	563932900
AX-111100937	A/G	G/G	A/A	Affx-109797629	1B	496471296
AX-111109396	G/G	A/G	A/A	Affx-111351050	1B	390276179
AX-111116890	T/T	T/C	T/T	Affx-110068417	1B	44503020
AX-111118968	G/G	A/G	A/A	Affx-109962894	1B	367588737
AX-111119209	T/T	C/C	C/C	Affx-109048863	1B	397999065
AX-111121168	T/T	C/C	T/T	Affx-111871975	1B	651496559
AX-111121879	G/G	A/G	A/A	Affx-109601238	1B	376791311
AX-111122222	G/G	A/G	G/G	Affx-111209800	1B	267335708
AX-111125943	C/G	C/C	C/C	Affx-109997990	1B	376367673
AX-111127325	C/C	A/A	C/C	Affx-110104718	1B	471818125
AX-111127329	C/C	T/C	T/T	Affx-111139389	1B	687069734
AX-111127879	C/C	C/C	C/C	Affx-111677870	1B	685250458
AX-111130381	G/G	A/G	G/G	Affx-111975239	1B	565517030
AX-111139084	G/G	A/G	G/G	Affx-111649194	1B	268698914
AX-111152864	G/G	A/G	G/G	Affx-111909406	1B	180560455
AX-111153034	T/T	T/C	C/C	Affx-108941398	1B	393998509
AX-111157060	T/T	A/T	T/T	Affx-109738629	1B	580520152
AX-111169510	T/T	T/T	G/G	Affx-110156787	1B	430155688
AX-111190944	A/A	A/C	A/A	Affx-108861152	1B	686755725
AX-111215445	G/G	A/G	G/G	Affx-110415555	1B	463745635
AX-111217038	T/T	A/T	A/A	Affx-109873535	1B	295451495
AX-111220018	C/C	T/C	T/T	Affx-110316660	1B	614315845
AX-111234844	T/T	T/C	T/T	Affx-111339183	1B	589948569
AX-111251872	A/A	A/G	A/A	Affx-108966953	1B	601372461
AX-111279813	T/T	T/C	T/T	Affx-110565928	1B	474760938
AX-111460132	A/A	A/G	A/A	Affx-110400915	1B	662206501
AX-111461819	C/C	T/C	C/C	Affx-109797470	1B	338428325
AX-111463788	C/C	T/C	T/T	Affx-111797364	1B	365926056
AX-111467003	G/G	T/G	G/G	Affx-111989796	1B	642958515
AX-111467657	C/C	T/C	C/C	Affx-110739884	1B	662188924
AX-111468437	G/G	T/G	T/T	Affx-111527550	1B	386240704
AX-111471029	C/C	T/C	C/C	Affx-109945449	1B	5488234
AX-111478905	G/G	G/G	G/G	Affx-111371582	1B	584167426
AX-111482877	A/A	A/G	G/G	Affx-88423682	1B	366492631
AX-111486408	G/G	C/G	C/C	Affx-88499705	1B	256922599
AX-111486820	T/T	T/C	C/C	Affx-109804576	1B	595094796
AX-111491365	G/G	C/C	C/C	Affx-110905408	1B	614939267
AX-111493581	C/C	A/C	A/A	Affx-109796271	1B	544715072
AX-111496509	C/C	C/G	G/G	Affx-109595049	1B	378971833
AX-111498627	C/C	T/C	T/T	Affx-111340704	1B	636323283
AX-111503092	C/C	T/C	T/T	Affx-109264359	1B	380647070
AX-111506075	C/C	A/C	A/A	Affx-109017477	1B	583407715
AX-111506135	C/C	T/C	T/T	Affx-109685232	1B	520372454
AX-111509539	G/G	A/G	G/G	Affx-111416288	1B	459930303
AX-111518016	C/C	C/G	G/G	Affx-111204563	1B	340807774
AX-111518766	C/C	C/C	C/C	Affx-88631048	1B	334938192
AX-111522827	C/C	T/C	T/T	Affx-109705753	1B	305525240
AX-111525116	T/T	T/C	T/T	Affx-110801774	1B	428802849
AX-111525505	G/G	A/G	G/G	Affx-88466045	1B	249238373
AX-111530524	C/C	T/C	T/T	Affx-110495237	1B	524128971

AX-111531877	C/C	T/C	T/T	Affx-111540881	1B	273050832
AX-111533735	G/G	G/G	G/G	Affx-111442513	1B	6412374
AX-111539937	C/C	T/C	T/T	Affx-109982072	1B	685231381
AX-111540229	A/A	A/C	C/C	Affx-111244976	1B	392457146
AX-111548801	G/G	A/G	G/G	Affx-111492401	1B	666062250
AX-111549107	C/C	T/C	T/T	Affx-111364897	1B	388341838
AX-111551816	A/A	A/T	A/T	Affx-109777109	1B	637621654
AX-111563015	G/G	T/G	G/G	Affx-110966428	1B	633975362
AX-111563253	G/G	G/G	G/G	Affx-111106797	1B	676182328
AX-111567165	T/T	T/G	G/G	Affx-112001091	1B	557459362
AX-111570432	T/T	T/C	T/T	Affx-88538438	1B	635127319
AX-111570613	T/C	T/C	T/T	Affx-110641451	1B	310394850
AX-111572620	T/G	T/G	T/G	Affx-111735992	1B	456434163
AX-111574871	A/A	A/G	G/G	Affx-110509883	1B	543607555
AX-111582734	C/C	A/A	A/A	Affx-111199777	1B	594035180
AX-111584259	C/C	A/C	C/C	Affx-109275902	1B	664283262
AX-111594013	C/C	G/G	G/G	Affx-88574389	1B	449703084
AX-111595814	G/G	G/G	G/G	Affx-109801859	1B	568521078
AX-111597240	G/G	A/G	G/G	Affx-109216151	1B	343953543
AX-111598691	G/G	A/G	A/A	Affx-88781124	1B	309378938
AX-111599494	T/T	T/C	C/C	Affx-109491892	1B	444807500
AX-111601677	C/C	T/C	C/C	Affx-109821266	1B	550939867
AX-111604635	A/A	A/G	G/G	Affx-109973961	1B	585684159
AX-111604965	C/C	C/G	C/C	Affx-109420723	1B	264762000
AX-111608329	T/T	T/T	T/T	Affx-110924943	1B	621986744
AX-111610602	T/T	C/C	C/C	Affx-111065796	1B	354492000
AX-111613078	T/T	T/C	C/C	Affx-111070195	1B	281068855
AX-111621728	T/T	C/C	T/T	Affx-109962057	1B	454888450
AX-111622338	C/C	T/C	C/C	Affx-111122691	1B	687580365
AX-111625326	C/C	C/G	G/G	Affx-88672410	1B	314120195
AX-111628099	A/A	A/G	A/A	Affx-88780505	1B	661131293
AX-111636804	G/G	T/G	T/T	Affx-111002496	1B	433952917
AX-111641463	T/T	T/G	G/G	Affx-108932370	1B	576512603
AX-111649750	C/C	T/C	T/T	Affx-109206193	1B	416825691
AX-111668114	T/C	C/C	C/C	Affx-108935303	1B	681261624
AX-111672727	G/G	A/G	A/A	Affx-111178386	1B	420711917
AX-111685499	G/G	A/G	G/G	Affx-110345950	1B	490765887
AX-111687828	G/G	A/G	G/G	Affx-108921390	1B	454359373
AX-111693350	T/T	T/C	T/T	Affx-111023019	1B	673569391
AX-111694700	A/A	A/G	G/G	Affx-109196455	1B	637978034
AX-111697462	A/G	G/G	A/G	Affx-110899739	1B	662205397
AX-111700606	G/G	A/G	G/G	Affx-109003974	1B	222386241
AX-111701712	G/G	A/G	A/A	Affx-111404788	1B	407510156
AX-111706137	G/G	G/G	A/A	Affx-109456009	1B	398146164
AX-111707424	T/T	T/C	C/C	Affx-88386137	1B	374316467
AX-111712993	A/A	A/T	T/T	Affx-109075247	1B	522358458
AX-111715015	C/C	C/C	A/A	Affx-88374395	1B	385300997
AX-111717169	G/G	A/G	A/A	Affx-109773272	1B	393512736
AX-111733823	A/A	A/G	G/G	Affx-110214130	1B	367838063
AX-111744000	C/C	C/G	G/G	Affx-110841735	1B	405524154
AX-111746919	G/G	A/G	A/A	Affx-110620955	1B	503663047
AX-111759802	T/T	T/C	C/C	Affx-111294895	1B	589390217
AX-111759816	G/G	T/G	T/T	Affx-109186989	1B	391416920
AX-111780371	C/C	T/C	T/T	Affx-110878600	1B	510356945

AX-111782039	G/G	T/T	T/T	Affx-110866216	1B	404883727
AX-111825076	G/G	T/G	T/T	Affx-88413963	1B	572683419
AX-112286185	A/A	A/G	A/A	Affx-88749242	1B	685288402
AX-112289703	G/G	A/G	G/G	Affx-92968106	1B	673176421
AX-182025562	A/T	A/T	A/T	Affx-110889017	1B	662189624
AX-182065029	T/T	T/C	T/T	Affx-472312124	1B	553631103
AX-182074338	C/C	T/C	C/C	Affx-88511752	1B	42348472
AX-182076796	C/C	T/C	C/C	Affx-109912128	1B	685185081
AX-182077586	C/C	A/C	C/C	Affx-109824168	1B	686780338
AX-182098040	A/A	A/G	A/A	Affx-108990307	1B	42313047
AX-182117945	A/C	A/C	A/C	Affx-111727651	1B	662189731
AX-182144578	A/G	A/G	G/G	Affx-472292295	1B	385157636
AX-182170463	G/G	A/G	G/G	Affx-472314797	1B	436929798
AX-182181442	T/C	C/C	C/C	Affx-88514154	1B	474118005
AX-86164127	C/C	C/G	G/G	Affx-92228849	1B	223144303
AX-86169309	T/T	T/C	T/T	Affx-109806757	1B	457320992
AX-86174914	A/A	A/G	G/G	Affx-109547505	1B	482713448
AX-86179242	G/G	G/G	G/G	Affx-92593510	1B	651544458
AX-86184871	C/C	T/C	T/T	Affx-92266067	1B	555766156
AX-86185005	G/G	C/G	G/G	Affx-88723993	1B	5510238
AX-89332288	A/A	A/G	A/A	Affx-88363637	1B	621268508
AX-89413394	C/C	T/C	T/T	Affx-88446000	1B	559960641
AX-89433121	C/C	T/C	C/C	Affx-88465823	1B	167111039
AX-89447384	T/C	T/C	T/T	Affx-88480140	1B	624734299
AX-89472833	C/C	T/C	T/T	Affx-88505651	1B	566016466
AX-89553276	T/C	T/T	T/T	Affx-88586310	1B	555772591
AX-89676030	G/G	A/G	G/G	Affx-88708805	1B	326461107
AX-89737974	A/A	A/G	G/G	Affx-88770661	1B	686756205
AX-89763895	G/G	A/G	A/A	Affx-88794713	1B	681685826
AX-94432535	C/C	C/C	C/C	Affx-92468991	1B	95665797
AX-94433968	G/G	C/G	G/G	Affx-88744859	1B	333323500
AX-94484295	A/G	G/G	G/G	Affx-92243739	1B	32776200
AX-94489390	A/A	A/G	A/A	Affx-92539280	1B	662154441
AX-94508976	C/C	T/C	T/T	Affx-92401023	1B	484887610
AX-94510790	T/T	T/G	T/T	Affx-92681065	1B	427526336
AX-94516827	C/C	T/C	C/C	Affx-92389643	1B	555775838
AX-94522381	T/T	C/C	T/T	Affx-92635666	1B	8181935
AX-94534923	C/C	C/G	C/C	Affx-92624137	1B	599339927
AX-94546432	G/G	C/G	C/C	Affx-92766102	1B	496563290
AX-94548073	A/C	C/C	A/C	Affx-92365826	1B	135938291
AX-94566803	C/C	C/C	C/C	Affx-92700590	1B	686753715
AX-94648555	A/A	G/G	A/A	Affx-92733922	1B	57379417
AX-94692514	C/C	C/G	C/C	Affx-92348574	1B	11633627
AX-94693513	G/G	A/G	G/G	Affx-92258802	1B	223144108
AX-94715187	T/T	T/C	C/C	Affx-92524620	1B	317013429
AX-94717933	C/C	C/C	T/C	Affx-92644273	1B	326015618
AX-94743546	C/C	C/C	C/C	Affx-92235037	1B	555765509
AX-94787647	A/C	A/A	A/A	Affx-92409478	1B	17790520
AX-94794099	C/G	C/G	C/C	Affx-92315101	1B	555765784
AX-94796731	G/G	G/G	G/G	Affx-92806407	1B	565672749
AX-94799615	A/A	A/C	A/A	Affx-92728381	1B	555935065
AX-94801528	T/T	T/T	T/T	Affx-92365945	1B	555933664
AX-94816512	C/G	C/C	C/G	Affx-92482741	1B	223144121
AX-94847341	T/C	T/C	T/C	Affx-92790003	1B	613001140

AX-94850271	C/C	T/T	C/C	Affx-92758244	1B	100309644
AX-94863489	T/C	T/T	T/C	Affx-92149432	1B	555935235
AX-94899004	C/C	T/T	C/C	Affx-92570950	1B	140296394
AX-94910851	T/T	T/G	G/G	Affx-92386655	1B	285339010
AX-94912344	C/G	C/C	C/G	Affx-92430641	1B	22074355
AX-94914633	T/T	T/G	T/T	Affx-92197029	1B	626595366
AX-94957845	T/T	T/G	G/G	Affx-92717279	1B	428069284
AX-94966346	G/G	C/C	C/G	Affx-92635528	1B	11937396
AX-94980178	A/A	G/G	A/A	Affx-92795891	1B	4346095
AX-95089648	C/C	C/C	T/C	Affx-92352126	1B	18570663
AX-95108394	T/C	T/C	T/C	Affx-92517203	1B	227792006
AX-95161958	C/C	T/T	C/C	Affx-92542801	1B	96593340
AX-95174732	T/T	T/C	T/T	Affx-92886177	1B	396361603
AX-95176576	G/G	G/G	A/A	Affx-92719524	1B	314569349
AX-95182909	A/C	A/A	A/A	Affx-92732651	1B	538865554
AX-95190562	A/A	A/G	A/A	Affx-92182936	1B	640849997
AX-95191511	A/A	A/C	A/A	Affx-92640109	1B	566993653
AX-95202607	A/G	A/A	A/G	Affx-92374342	1B	678469978
AX-95213134	C/C	C/C	A/C	Affx-92132397	1B	16464540
AX-95224065	A/C	C/C	A/C	Affx-92237984	1B	225786048
AX-95244854	G/G	C/G	G/G	Affx-92577636	1B	158534186
AX-95250058	A/A	G/G	G/G	Affx-92096501	1B	16672239
AX-95255966	A/G	A/G	A/G	Affx-92856665	1B	539930329
AX-95632148	C/C	C/C	C/C	Affx-88432751	1B	548624786
AX-95633968	G/G	G/G	G/G	Affx-92586457	1B	222312515
AX-95654644	A/G	A/A	A/G	Affx-88436043	1B	566603733
AX-95654748	G/G	T/G	T/T	Affx-88673796	1B	445180564
AX-95683975	C/C	T/C	C/C	Affx-109854548	1B	340372963
AX-108727343	T/T	T/C	T/T	Affx-111593023	1D	26410363
AX-108745560	A/A	A/G	A/A	Affx-111811702	1D	38071759
AX-108747591	T/T	T/C	T/T	Affx-111615622	1D	223345533
AX-108760374	C/C	T/C	T/T	Affx-110350142	1D	275939984
AX-108770853	G/G	T/G	G/G	Affx-88528367	1D	411733093
AX-108774920	A/A	A/G	A/A	Affx-109132053	1D	439129334
AX-108775918	T/T	T/C	T/T	Affx-108960752	1D	309265557
AX-108784335	G/G	C/G	G/G	Affx-110249683	1D	40798236
AX-108791856	A/A	A/T	A/A	Affx-109848255	1D	468358389
AX-108797722	T/T	T/C	T/T	Affx-110023696	1D	230009578
AX-108816282	C/C	A/C	C/C	Affx-109168968	1D	463490283
AX-108821534	G/G	A/G	G/G	Affx-110339219	1D	473261171
AX-108822777	A/A	A/G	G/G	Affx-110826382	1D	317386880
AX-108833424	A/A	A/G	G/G	Affx-109773804	1D	218408723
AX-108842099	A/G	A/G	G/G	Affx-111441368	1D	214185496
AX-108851268	G/G	A/G	A/A	Affx-109355069	1D	3761325
AX-108862399	T/T	T/C	T/T	Affx-111960283	1D	477291386
AX-108865120	C/C	T/C	C/C	Affx-88409273	1D	415393096
AX-108873908	C/C	T/C	C/C	Affx-111076126	1D	30235522
AX-108894070	G/G	A/G	G/G	Affx-109793539	1D	362856964
AX-108896406	C/C	T/C	C/C	Affx-109978549	1D	19884528
AX-108897098	G/G	A/A	G/G	Affx-111562014	1D	11549644
AX-108906001	C/C	C/G	C/C	Affx-111198097	1D	462607065
AX-108906291	T/T	T/C	T/T	Affx-111561165	1D	432642615
AX-108942729	T/T	T/C	T/T	Affx-111298941	1D	244050349
AX-108943530	G/G	A/G	A/A	Affx-109596858	1D	319235853

AX-108966701	T/T	T/C	T/T	Affx-111742003	1D	473675474
AX-108966759	G/G	A/G	G/G	Affx-111383091	1D	55702577
AX-109010441	A/A	A/G	G/G	Affx-109811410	1D	422055222
AX-109042683	G/G	A/G	G/G	Affx-108938950	1D	47014297
AX-109149937	A/A	A/G	A/A	Affx-109600155	1D	254982755
AX-109154181	T/T	T/C	T/T	Affx-110351046	1D	227472007
AX-109161472	G/G	A/G	G/G	Affx-109656411	1D	187861946
AX-109211660	T/T	T/C	T/T	Affx-109178833	1D	28231546
AX-109224120	T/T	T/C	T/T	Affx-109475987	1D	484425653
AX-109247862	C/C	T/C	C/C	Affx-110150707	1D	13606887
AX-109271243	A/A	A/G	A/A	Affx-109662653	1D	9424088
AX-109276502	C/C	T/C	T/T	Affx-110252138	1D	288318891
AX-109292966	T/T	T/G	T/T	Affx-109947922	1D	234337247
AX-109294770	A/G	A/G	A/G	Affx-111289895	1D	485744077
AX-109319979	G/G	A/G	G/G	Affx-111332835	1D	410596143
AX-109320713	G/G	A/G	G/G	Affx-109987398	1D	24055581
AX-109322370	T/T	T/C	T/T	Affx-109928849	1D	256231188
AX-109326727	G/G	T/G	G/G	Affx-111594219	1D	7381436
AX-109343644	G/G	T/G	G/G	Affx-111758973	1D	419604138
AX-109347906	G/G	T/G	G/G	Affx-111080098	1D	460646658
AX-109352933	G/G	A/G	G/G	Affx-111518642	1D	449289500
AX-109391090	C/C	T/C	C/C	Affx-88384929	1D	447513558
AX-109403688	G/G	A/G	G/G	Affx-109730169	1D	18839050
AX-109403791	A/A	A/G	G/G	Affx-110219690	1D	271839634
AX-109407881	A/A	A/A	A/A	Affx-110929552	1D	44374566
AX-109416076	C/C	T/C	T/T	Affx-88646447	1D	274036539
AX-109444408	T/T	T/C	T/T	Affx-110418944	1D	494944501
AX-109451746	A/A	A/A	A/A	Affx-110627830	1D	21916285
AX-109469829	A/A	A/T	A/A	Affx-109037015	1D	15156071
AX-109475166	G/G	A/G	A/A	Affx-88419760	1D	327936477
AX-109484217	C/C	C/G	G/G	Affx-111944948	1D	340410355
AX-109603790	G/G	C/G	G/G	Affx-109365086	1D	141642261
AX-109604527	G/G	A/G	G/G	Affx-111828376	1D	66929588
AX-109627222	A/A	A/G	A/A	Affx-109948758	1D	43809885
AX-109680404	A/A	A/G	A/A	Affx-110005327	1D	361098571
AX-109690371	C/C	T/C	C/C	Affx-111282743	1D	344692530
AX-109746270	G/G	A/G	G/G	Affx-110014210	1D	433528632
AX-109793947	C/C	T/C	T/T	Affx-111757473	1D	283341813
AX-109803173	G/G	A/G	G/G	Affx-111548833	1D	44934131
AX-109816719	G/G	A/G	G/G	Affx-110305950	1D	439872729
AX-109825988	A/A	A/C	A/A	Affx-110023511	1D	220916654
AX-109830556	T/T	T/C	T/T	Affx-88525803	1D	435727476
AX-109834055	C/C	T/C	C/C	Affx-110852281	1D	47448008
AX-109848337	G/G	T/G	G/G	Affx-109213679	1D	81376551
AX-109849585	A/A	A/C	A/A	Affx-110139990	1D	39169012
AX-109864616	C/C	C/G	G/G	Affx-110880749	1D	335593288
AX-109867992	C/G	C/G	G/G	Affx-111968259	1D	487810162
AX-109873298	A/A	A/G	G/G	Affx-109736306	1D	384735628
AX-109877474	T/T	T/G	T/T	Affx-109737228	1D	57724905
AX-109887577	T/T	T/C	T/T	Affx-88700331	1D	43303785
AX-109902058	A/A	A/C	A/A	Affx-111371818	1D	474299646
AX-109902923	C/C	A/C	C/C	Affx-110992380	1D	2510758
AX-109907213	T/T	T/C	T/T	Affx-110713219	1D	114133690
AX-109908110	G/G	A/G	G/G	Affx-109711150	1D	9818744

AX-109911808	A/A	A/G	A/A	Affx-109917847	1D	458831515
AX-109931525	A/A	A/C	A/A	Affx-109474301	1D	52722148
AX-109934150	C/C	T/C	C/C	Affx-110634002	1D	403803989
AX-109955671	G/G	A/G	G/G	Affx-109372813	1D	37559070
AX-109972357	C/C	T/C	C/C	Affx-110053498	1D	457773597
AX-109973213	A/A	A/G	A/A	Affx-111753045	1D	420653158
AX-109977118	G/G	A/A	G/G	Affx-109823383	1D	158107643
AX-110019493	C/C	C/C	C/C	Affx-110829398	1D	45394092
AX-110028964	G/G	A/G	A/A	Affx-111224670	1D	272212007
AX-110029901	T/T	T/C	T/T	Affx-109799799	1D	2691667
AX-110059262	T/T	C/C	C/C	Affx-88681530	1D	391110538
AX-110066480	G/G	A/G	G/G	Affx-88580119	1D	249601945
AX-110132466	T/T	C/C	T/T	Affx-109103372	1D	38652952
AX-110142397	G/G	A/G	G/G	Affx-109707824	1D	20414994
AX-110196952	C/C	A/A	C/C	Affx-110631859	1D	16764563
AX-110249715	G/G	A/G	G/G	Affx-110889256	1D	56409892
AX-110270891	T/T	T/C	T/T	Affx-110712301	1D	66543821
AX-110297825	A/A	A/G	A/A	Affx-109102206	1D	18382410
AX-110317303	C/C	T/C	C/C	Affx-109624267	1D	436147309
AX-110332164	G/G	A/G	G/G	Affx-88439684	1D	458941978
AX-110340507	G/G	A/G	A/A	Affx-109667628	1D	270167261
AX-110389611	A/A	A/G	A/A	Affx-111826972	1D	248170970
AX-110394042	T/C	C/C	C/C	Affx-111196681	1D	343768882
AX-110401799	G/G	A/G	G/G	Affx-110975119	1D	443102647
AX-110404427	T/C	T/C	C/C	Affx-111387387	1D	328297911
AX-110425252	A/A	A/A	A/A	Affx-109164933	1D	2143935
AX-110428172	C/C	T/C	C/C	Affx-111751577	1D	479876451
AX-110436665	A/A	A/G	A/A	Affx-110953331	1D	478296988
AX-110448375	T/T	T/C	T/T	Affx-88743127	1D	2082331
AX-110449657	A/A	A/G	A/A	Affx-108972761	1D	250330210
AX-110466737	A/C	A/C	A/C	Affx-110173660	1D	464312891
AX-110472474	A/A	G/G	G/G	Affx-111117724	1D	431767641
AX-110490771	C/C	C/G	G/G	Affx-110266174	1D	302906441
AX-110493828	C/C	T/C	C/C	Affx-109427790	1D	427323196
AX-110501128	A/A	A/C	A/A	Affx-110860901	1D	36360126
AX-110517637	C/C	C/C	T/T	Affx-111473367	1D	338824357
AX-110519356	C/G	C/G	G/G	Affx-110039218	1D	1149707
AX-110538416	C/C	C/G	C/C	Affx-111742103	1D	434613679
AX-110541688	T/T	T/C	T/T	Affx-109800904	1D	62768834
AX-110562177	T/T	T/C	T/T	Affx-110482586	1D	180817148
AX-110581400	T/T	T/C	T/T	Affx-109798320	1D	479582629
AX-110592232	A/A	G/G	A/A	Affx-111878967	1D	486365358
AX-110595695	T/T	T/G	G/G	Affx-109647125	1D	320173505
AX-110601979	A/G	A/G	A/A	Affx-88788261	1D	292704788
AX-110669416	A/A	A/T	T/T	Affx-88504303	1D	296894137
AX-110691406	A/A	A/C	C/C	Affx-110768395	1D	225510768
AX-110711076	C/C	C/G	G/G	Affx-88562668	1D	339324005
AX-110711126	C/C	T/C	C/C	Affx-109001600	1D	49111128
AX-110711194	C/C	T/C	C/C	Affx-110819079	1D	423301609
AX-110755357	C/C	T/C	C/C	Affx-108945708	1D	64978839
AX-110782380	C/C	T/C	C/C	Affx-110118231	1D	63426387
AX-110782818	A/A	A/G	A/A	Affx-111557963	1D	250870210
AX-110815139	G/G	A/G	A/A	Affx-88499997	1D	324706009
AX-110878500	A/A	A/G	G/G	Affx-110474792	1D	343288661

AX-110899756	G/G	A/G	G/G	Affx-111600648	1D	365283025
AX-110903292	A/A	A/G	A/A	Affx-109243714	1D	468143780
AX-110913331	C/C	T/C	C/C	Affx-111322300	1D	69260500
AX-110914177	G/G	T/G	T/T	Affx-88402876	1D	3195409
AX-110914190	C/C	T/C	C/C	Affx-109551609	1D	482835321
AX-110915500	T/T	T/C	T/T	Affx-110676283	1D	54944586
AX-110925830	G/G	C/G	G/G	Affx-108929940	1D	451143783
AX-110934628	A/A	A/G	A/A	Affx-109506848	1D	413307926
AX-110935812	G/G	A/G	A/A	Affx-111746763	1D	292122764
AX-110938812	A/A	A/G	A/A	Affx-108919511	1D	426174092
AX-110953480	A/A	A/A	A/A	Affx-108904487	1D	474933435
AX-110957338	C/C	T/T	C/C	Affx-111204760	1D	246673559
AX-110964678	G/G	C/G	G/G	Affx-111986667	1D	46456721
AX-110985056	A/A	A/A	A/A	Affx-110810156	1D	398740112
AX-111003889	T/T	T/C	C/C	Affx-110200646	1D	356525752
AX-111019122	A/A	A/G	A/A	Affx-88549345	1D	416455295
AX-111023958	T/T	T/C	T/T	Affx-110613456	1D	175415911
AX-111050579	A/A	C/C	A/A	Affx-111399008	1D	58543156
AX-111054188	C/C	A/C	C/C	Affx-109707413	1D	97916993
AX-111060518	C/C	T/C	C/C	Affx-111507517	1D	408591672
AX-111066559	A/A	A/G	G/G	Affx-111398419	1D	278099954
AX-111084369	T/T	T/T	T/T	Affx-111774443	1D	34946910
AX-111086781	A/A	A/G	G/G	Affx-110844727	1D	300949936
AX-111094817	T/T	T/C	T/T	Affx-111535266	1D	178101573
AX-111102983	C/C	T/T	C/C	Affx-110619565	1D	8387434
AX-111104769	A/A	A/G	G/G	Affx-88619398	1D	388980087
AX-111109010	T/T	T/C	C/C	Affx-109131094	1D	398573861
AX-111111736	C/C	C/C	T/T	Affx-88467549	1D	203867554
AX-111116895	G/G	A/G	A/A	Affx-111075825	1D	276211123
AX-111142978	G/G	T/T	G/G	Affx-88722439	1D	41766420
AX-111143912	T/C	T/C	C/C	Affx-109969457	1D	403068837
AX-111144748	T/T	T/G	T/T	Affx-109671898	1D	42288655
AX-111154755	A/A	A/T	A/A	Affx-88784416	1D	17259088
AX-111157081	A/A	A/G	A/A	Affx-109398292	1D	249659215
AX-111161814	C/C	T/C	C/C	Affx-110865062	1D	487288353
AX-111218609	G/G	A/G	A/A	Affx-108922569	1D	281976438
AX-111222830	G/G	A/G	G/G	Affx-111186558	1D	356251497
AX-111234221	G/G	G/G	A/A	Affx-111482485	1D	271159812
AX-111259344	C/C	T/C	C/C	Affx-109721951	1D	246019385
AX-111266392	A/A	A/C	A/A	Affx-110406886	1D	12564891
AX-111298055	C/C	T/C	C/C	Affx-111998117	1D	125713824
AX-111317494	C/C	C/C	A/A	Affx-111621738	1D	321641448
AX-111340592	T/T	T/C	T/T	Affx-110684812	1D	12217875
AX-111342069	G/G	T/G	G/G	Affx-109355237	1D	449648115
AX-111345949	T/T	T/C	T/T	Affx-110533825	1D	253194257
AX-111377235	A/A	A/C	A/A	Affx-110632840	1D	432341979
AX-111382637	A/G	A/G	A/A	Affx-110176033	1D	418529886
AX-111382744	C/C	T/C	T/T	Affx-110734120	1D	290416494
AX-111384150	G/G	A/A	G/G	Affx-111376128	1D	39647525
AX-111385220	T/T	T/G	T/T	Affx-110797956	1D	40204504
AX-111404590	T/T	T/C	T/T	Affx-109893874	1D	417570479
AX-111432746	C/C	A/C	C/C	Affx-110764630	1D	160871494
AX-111448639	C/C	T/C	C/C	Affx-88352015	1D	417097168
AX-111449828	G/G	T/G	T/T	Affx-111369128	1D	233341193

AX-111462038	A/A	A/C	A/A	Affx-111959820	1D	54376593
AX-111462673	C/C	C/C	C/C	Affx-108880281	1D	485559586
AX-111477094	A/A	A/C	A/A	Affx-111437874	1D	41310656
AX-111481948	C/C	T/C	T/T	Affx-111584001	1D	324620935
AX-111482680	A/A	A/G	A/A	Affx-109304206	1D	20899707
AX-111484181	C/C	T/C	C/C	Affx-111994552	1D	376870037
AX-111486847	A/A	A/G	A/A	Affx-109181044	1D	476897578
AX-111504007	T/T	T/C	T/T	Affx-109838019	1D	238590321
AX-111505034	G/G	A/G	G/G	Affx-108883098	1D	448643614
AX-111506698	G/G	T/G	G/G	Affx-109963063	1D	262037290
AX-111515122	C/C	C/C	C/C	Affx-111676701	1D	22627999
AX-111525866	T/T	T/C	T/T	Affx-111352454	1D	263206187
AX-111542671	T/T	T/C	C/C	Affx-109956358	1D	317076819
AX-111552331	C/C	T/C	C/C	Affx-110239907	1D	411236732
AX-111558345	G/G	A/G	A/A	Affx-88488774	1D	363102979
AX-111575769	G/G	A/G	A/A	Affx-88469980	1D	337993385
AX-111596833	C/C	T/C	T/T	Affx-111279386	1D	233804449
AX-111604536	G/G	G/G	A/A	Affx-110491596	1D	398868962
AX-111611089	A/A	G/G	A/A	Affx-111554338	1D	465441408
AX-111611926	G/G	T/G	G/G	Affx-109634736	1D	319277352
AX-111632760	T/T	T/C	T/T	Affx-111223895	1D	454809902
AX-111633021	G/G	A/G	G/G	Affx-110971895	1D	415982829
AX-111639777	T/T	T/C	T/T	Affx-108946149	1D	147725106
AX-111642834	T/T	T/T	T/T	Affx-109914453	1D	42822335
AX-111655785	C/C	T/C	C/C	Affx-109632810	1D	465447405
AX-111657285	C/C	C/G	C/C	Affx-110525288	1D	31431978
AX-111657506	C/C	T/C	C/C	Affx-111902535	1D	35593746
AX-111689845	A/A	G/G	A/A	Affx-109845067	1D	2510793
AX-111696550	G/G	G/G	A/A	Affx-110453594	1D	284675388
AX-111698420	C/C	C/C	T/T	Affx-110098135	1D	427312100
AX-111739692	C/C	T/C	C/C	Affx-111653210	1D	28570280
AX-111775358	A/A	A/G	A/A	Affx-111181578	1D	222329535
AX-111784010	A/A	A/G	A/A	Affx-109399743	1D	410253074
AX-111834269	G/G	A/G	G/G	Affx-109784651	1D	48483141
AX-111845708	C/C	T/C	T/T	Affx-109000362	1D	480522814
AX-111861594	C/C	T/C	T/T	Affx-111579670	1D	294945399
AX-111904596	A/A	A/G	A/A	Affx-109324679	1D	21462131
AX-111912186	C/C	T/T	C/C	Affx-110366685	1D	442695124
AX-111946510	T/T	T/C	T/T	Affx-111001615	1D	478865323
AX-111949480	T/T	T/C	T/T	Affx-111914273	1D	2512461
AX-111972540	C/C	T/C	C/C	Affx-110089774	1D	129965629
AX-111973810	T/T	T/C	T/T	Affx-111495372	1D	429988897
AX-111976657	C/C	T/C	C/C	Affx-111991237	1D	274558716
AX-111976732	G/G	A/G	A/A	Affx-110318329	1D	232481676
AX-111980689	C/C	T/C	T/T	Affx-110612805	1D	252477049
AX-111989456	A/A	A/A	A/A	Affx-109388756	1D	34385389
AX-112288941	G/G	G/G	G/G	Affx-88664771	1D	308269723
AX-176642000	A/G	G/G	A/A	Affx-111667063	1D	2506244
AX-179558649	T/T	T/G	T/T	Affx-88587097	1D	414301748
AX-182178301	T/T	T/T	T/T	Affx-472322165	1D	295970307
AX-86164321	T/T	T/C	T/T	Affx-88794902	1D	32544853
AX-86171903	T/T	T/C	T/T	Affx-88427783	1D	8606637
AX-86175013	C/C	C/C	C/C	Affx-88560772	1D	408244146
AX-86178400	C/C	T/C	C/C	Affx-92684204	1D	462203580

AX-86184274	G/G	C/G	G/G	Affx-92541051	1D	57476693
AX-86185223	A/A	A/G	A/A	Affx-88656176	1D	347209497
AX-89322703	G/G	G/G	G/G	Affx-88353725	1D	477761249
AX-89331569	G/G	A/A	G/G	Affx-88362896	1D	246474998
AX-89352046	T/T	T/C	T/T	Affx-88383899	1D	32482238
AX-89479023	A/A	A/C	C/C	Affx-88511863	1D	395555220
AX-89510455	G/G	A/G	G/G	Affx-88543340	1D	404139401
AX-89527061	T/T	T/T	T/T	Affx-88559997	1D	413061160
AX-89566231	T/C	T/T	T/C	Affx-92779659	1D	402833633
AX-89617229	G/G	A/G	G/G	Affx-110445086	1D	443743448
AX-89641535	C/C	T/T	T/T	Affx-88674327	1D	459215
AX-89677377	G/G	A/G	G/G	Affx-109317726	1D	464856673
AX-94390476	G/G	G/G	G/G	Affx-92791923	1D	52339516
AX-94439418	A/G	A/G	G/G	Affx-92898551	1D	360152385
AX-94452612	A/C	A/A	A/C	Affx-92933369	1D	197741402
AX-94483528	A/A	G/G	A/A	Affx-92241900	1D	433258903
AX-94491903	C/C	T/C	C/C	Affx-92621654	1D	73679336
AX-94520263	T/T	T/C	T/T	Affx-92212803	1D	476559967
AX-94536972	A/G	G/G	A/G	Affx-92530595	1D	470895867
AX-94567834	T/G	G/G	T/G	Affx-92190278	1D	79054647
AX-94568928	T/C	C/C	T/C	Affx-92390028	1D	142339386
AX-94592833	A/A	A/T	A/A	Affx-92925698	1D	55668825
AX-94595542	C/C	C/C	C/C	Affx-92886539	1D	2498101
AX-94613367	A/A	A/G	A/A	Affx-92414953	1D	414303471
AX-94621037	C/G	C/G	C/G	Affx-92535498	1D	474822782
AX-94726629	A/A	A/A	A/A	Affx-92542727	1D	355507949
AX-94745216	T/C	T/C	C/C	Affx-92796544	1D	206310777
AX-94820546	A/A	A/G	G/G	Affx-92334415	1D	318497325
AX-94826666	G/G	C/G	G/G	Affx-92632012	1D	33706214
AX-94857942	A/A	A/G	A/A	Affx-92459477	1D	86924197
AX-94865294	A/G	A/A	A/G	Affx-92671583	1D	427771961
AX-94893744	C/C	T/C	C/C	Affx-88796897	1D	366197995
AX-94897297	T/T	T/C	T/T	Affx-88563228	1D	50296821
AX-94920155	C/C	A/A	C/C	Affx-92450140	1D	2485638
AX-94921655	T/T	T/C	T/T	Affx-92941814	1D	2486095
AX-94935157	T/T	T/T	T/T	Affx-92962830	1D	470729143
AX-94988029	A/A	A/G	A/A	Affx-92126852	1D	56753155
AX-95003107	A/G	A/A	A/G	Affx-92319129	1D	491031010
AX-95012515	C/C	C/C	C/C	Affx-92404840	1D	412161094
AX-95096526	A/G	G/G	A/G	Affx-92126754	1D	70571588
AX-95126009	C/C	C/C	C/C	Affx-92095872	1D	419061436
AX-95166896	G/G	A/G	G/G	Affx-92183321	1D	307214665
AX-95191356	G/G	A/G	G/G	Affx-92712250	1D	240598374
AX-95224233	C/C	C/G	C/C	Affx-92387077	1D	2486098
AX-95255982	C/C	T/C	C/C	Affx-92471498	1D	49982410
AX-95684095	A/A	A/G	A/A	Affx-88752382	1D	408076260
AX-108725242	T/T	T/C	T/T	Affx-109561496	2A	707307102
AX-108726641	G/G	A/G	G/G	Affx-111705728	2A	191661613
AX-108727827	A/A	A/G	G/G	Affx-109202410	2A	488711207
AX-108729568	T/T	T/C	C/C	Affx-110101550	2A	36691094
AX-108730507	C/C	C/C	C/C	Affx-111523946	2A	775177316
AX-108730878	C/C	C/C	C/C	Affx-88592695	2A	450888757
AX-108731034	T/T	T/C	C/C	Affx-109518793	2A	619778658
AX-108733196	G/G	A/G	A/A	Affx-111851959	2A	703697674

AX-108736779	G/G	G/G	G/G	Affx-109580962	2A	723836613
AX-108736884	C/C	T/C	C/C	Affx-111039774	2A	70466202
AX-108737030	T/T	T/C	T/T	Affx-88620061	2A	90748859
AX-108740470	G/G	G/G	G/G	Affx-110030547	2A	773477239
AX-108741112	A/A	A/C	A/A	Affx-111603243	2A	63924247
AX-108741878	A/G	G/G	A/G	Affx-109868728	2A	24312723
AX-108746627	G/G	A/G	G/G	Affx-108874920	2A	166832823
AX-108748890	T/T	T/C	T/T	Affx-110337033	2A	99657139
AX-108749391	A/G	A/G	A/A	Affx-111536060	2A	5891463
AX-108751272	C/C	A/C	A/A	Affx-88560988	2A	704705895
AX-108752202	A/A	A/G	A/G	Affx-111678174	2A	523660145
AX-108752520	C/C	T/C	C/C	Affx-109526066	2A	637670079
AX-108754042	T/G	T/G	G/G	Affx-110873885	2A	712195205
AX-108754792	A/A	A/A	A/A	Affx-108858436	2A	181825469
AX-108756875	C/G	G/G	G/G	Affx-110032664	2A	773513807
AX-108757769	A/G	A/G	G/G	Affx-109365782	2A	691701805
AX-108758337	C/C	T/C	C/C	Affx-109902104	2A	705262523
AX-108760728	A/A	A/T	A/A	Affx-109162730	2A	416559461
AX-108762870	C/C	A/C	C/C	Affx-110271565	2A	337215204
AX-108763047	G/G	A/G	G/G	Affx-109264106	2A	555479878
AX-108764087	A/A	A/G	A/A	Affx-109080716	2A	458972183
AX-108771187	C/C	G/G	C/C	Affx-111461269	2A	566602667
AX-108772316	G/G	A/G	G/G	Affx-111443142	2A	618431097
AX-108776811	G/G	A/G	G/G	Affx-111687435	2A	741083161
AX-108777020	T/C	T/C	T/T	Affx-111557509	2A	723145304
AX-108778918	A/A	A/G	A/A	Affx-110779639	2A	362403151
AX-108780549	A/A	A/C	A/A	Affx-109688436	2A	625566277
AX-108782406	A/A	A/G	A/A	Affx-111873289	2A	417053197
AX-108788038	C/C	T/C	C/C	Affx-110526618	2A	91496408
AX-108788135	C/C	T/C	C/C	Affx-88524103	2A	533951446
AX-108793362	T/T	T/G	G/G	Affx-112003121	2A	654861962
AX-108794050	A/A	G/G	G/G	Affx-88522323	2A	698175312
AX-108796626	C/G	C/G	G/G	Affx-111094332	2A	711520792
AX-108797935	C/C	T/C	T/C	Affx-111906548	2A	515308729
AX-108797947	A/A	A/G	A/A	Affx-109229983	2A	744405533
AX-108798703	A/A	A/G	A/A	Affx-109423067	2A	374927247
AX-108798996	C/C	T/C	C/C	Affx-110605962	2A	378396172
AX-108807379	A/C	A/C	C/C	Affx-109414810	2A	674538688
AX-108812607	A/A	A/G	G/G	Affx-110931178	2A	507015642
AX-108813587	C/C	T/C	C/C	Affx-110680488	2A	84543649
AX-108814760	C/C	T/C	C/C	Affx-110280036	2A	264159488
AX-108816422	T/T	T/C	T/T	Affx-110701859	2A	351409607
AX-108817652	C/C	T/C	C/C	Affx-109275954	2A	241413012
AX-108820015	A/G	A/A	G/G	Affx-111892350	2A	33339365
AX-108822367	G/G	G/G	G/G	Affx-109408006	2A	595221601
AX-108822824	C/C	T/C	C/C	Affx-108851780	2A	387613337
AX-108826483	T/T	T/C	T/T	Affx-109281177	2A	398675637
AX-108829822	T/T	A/A	A/A	Affx-110725076	2A	578948247
AX-108830384	C/C	C/G	C/C	Affx-110403649	2A	386290386
AX-108831697	T/T	T/C	T/T	Affx-110952499	2A	407737169
AX-108837723	G/G	A/G	G/G	Affx-110852303	2A	261922713
AX-108847326	C/C	T/C	T/T	Affx-110569080	2A	718829717
AX-108847910	A/A	A/G	A/A	Affx-109493651	2A	323117801
AX-108850995	A/A	A/C	A/A	Affx-109622003	2A	312062101

AX-108853639	A/A	A/C	A/A	Affx-111741242	2A	374083673
AX-108855326	A/A	A/G	A/A	Affx-109348743	2A	137719741
AX-108856880	G/G	C/G	C/C	Affx-110529796	2A	636942552
AX-108858837	C/C	G/G	C/C	Affx-109180855	2A	165742936
AX-108859404	T/T	T/C	T/T	Affx-109226461	2A	532501451
AX-108860676	T/T	T/C	T/T	Affx-108888302	2A	272277206
AX-108862282	T/T	T/C	T/T	Affx-109585114	2A	281199449
AX-108866779	T/T	T/C	T/T	Affx-111406050	2A	369201690
AX-108870568	A/A	A/A	A/A	Affx-109229567	2A	423206208
AX-108871469	T/C	C/C	T/C	Affx-109688214	2A	694865571
AX-108872297	G/G	A/G	G/G	Affx-110518133	2A	755754906
AX-108873118	T/T	T/C	T/T	Affx-111204356	2A	121013178
AX-108877039	A/A	A/G	A/A	Affx-111107436	2A	310927739
AX-108877841	A/A	A/G	A/A	Affx-109236645	2A	66295099
AX-108878043	G/G	A/G	G/G	Affx-110073812	2A	709581813
AX-108878388	C/C	C/G	C/C	Affx-110431470	2A	284847912
AX-108880717	C/C	C/C	C/C	Affx-109819022	2A	94337951
AX-108885412	G/G	C/G	C/C	Affx-109730562	2A	42775274
AX-108887064	A/G	A/G	A/G	Affx-88700895	2A	461494375
AX-108887315	T/T	T/G	T/T	Affx-88431379	2A	461010115
AX-108888086	G/G	G/G	A/A	Affx-108856063	2A	508031457
AX-108888172	A/A	A/T	T/T	Affx-110065757	2A	676164880
AX-108888319	C/C	C/G	G/G	Affx-110959806	2A	719305063
AX-108892822	G/G	A/G	G/G	Affx-111841988	2A	233469608
AX-108900832	C/C	T/C	C/C	Affx-110293000	2A	341936923
AX-108903144	T/T	C/C	C/C	Affx-110945029	2A	624355649
AX-108903952	A/A	A/G	A/A	Affx-111885506	2A	254670735
AX-108909435	T/T	T/C	T/T	Affx-88720826	2A	365374675
AX-108909605	T/T	T/C	T/T	Affx-109269463	2A	464307045
AX-108910006	G/G	A/G	G/G	Affx-110170554	2A	103810925
AX-108910927	T/T	T/G	T/T	Affx-110033091	2A	204992189
AX-108913451	A/A	A/C	A/A	Affx-111557901	2A	475586589
AX-108915431	C/C	T/C	C/C	Affx-112000434	2A	258792053
AX-108917349	C/C	T/C	C/C	Affx-110695018	2A	285408079
AX-108917352	A/A	A/G	G/G	Affx-109365824	2A	476598342
AX-108918456	T/T	T/T	T/T	Affx-111628640	2A	750368127
AX-108924975	A/A	A/G	A/A	Affx-110097397	2A	224605008
AX-108927083	A/A	A/A	A/A	Affx-88790082	2A	223611657
AX-108927963	C/C	C/G	C/C	Affx-88363456	2A	419647959
AX-108930705	C/C	T/C	C/C	Affx-110935551	2A	77767585
AX-108931530	A/A	A/A	A/A	Affx-110428607	2A	294383380
AX-108933054	C/C	T/C	C/C	Affx-109629593	2A	184931543
AX-108933205	G/G	A/G	G/G	Affx-110072969	2A	744686507
AX-108934390	A/A	A/A	G/G	Affx-110386362	2A	563945960
AX-108934714	C/C	C/G	C/C	Affx-109511788	2A	301458365
AX-108937736	G/G	A/G	G/G	Affx-110278198	2A	742693322
AX-108938046	G/G	T/G	G/G	Affx-111092190	2A	310080347
AX-108939508	C/C	C/G	C/C	Affx-110258099	2A	726103014
AX-108941983	A/A	A/G	A/A	Affx-111942128	2A	413495472
AX-108942101	A/A	A/G	A/A	Affx-109195140	2A	131232866
AX-108943315	A/A	A/G	A/A	Affx-110747525	2A	353939970
AX-108945005	G/G	G/G	A/A	Affx-111777163	2A	619622294
AX-108949998	C/G	C/G	C/G	Affx-111978413	2A	740138704
AX-108954150	A/A	A/G	A/A	Affx-110897763	2A	247034362

AX-108954440	A/A	A/G	A/A	Affx-109420088	2A	210037139
AX-108960874	A/A	A/G	A/A	Affx-111220313	2A	133333815
AX-108961585	G/G	A/A	G/G	Affx-109330467	2A	43479980
AX-108968163	C/C	T/C	C/C	Affx-109957125	2A	236770729
AX-108970659	T/T	T/G	T/T	Affx-111625804	2A	472294151
AX-108971787	T/T	T/C	T/T	Affx-110378302	2A	237512130
AX-108983807	G/G	A/G	G/G	Affx-110097663	2A	535420929
AX-108987688	G/G	C/G	G/G	Affx-109814374	2A	385905150
AX-108989384	A/A	A/G	A/A	Affx-111451553	2A	728616286
AX-108996098	A/G	G/G	A/G	Affx-111589721	2A	37271835
AX-109000762	A/A	A/C	A/A	Affx-109246220	2A	777618495
AX-109031576	T/T	T/C	T/T	Affx-109254745	2A	47175271
AX-109034269	T/T	T/C	T/T	Affx-110373119	2A	408808050
AX-109035014	T/T	T/T	G/G	Affx-110546564	2A	499765864
AX-109043229	C/C	T/C	C/C	Affx-109414156	2A	172016242
AX-109043882	A/A	A/G	A/A	Affx-110912715	2A	460445544
AX-109053578	T/T	T/C	T/T	Affx-110184105	2A	324953502
AX-109053822	A/A	A/C	A/A	Affx-111516705	2A	198472675
AX-109058027	T/T	T/C	T/T	Affx-108935953	2A	291028203
AX-109062065	A/A	A/G	A/A	Affx-111148513	2A	382173530
AX-109067976	G/G	T/G	G/G	Affx-109808110	2A	758295272
AX-109075563	A/A	A/G	A/A	Affx-109143924	2A	432560923
AX-109079508	T/T	C/C	C/C	Affx-108851111	2A	62305002
AX-109081995	A/A	A/C	A/A	Affx-109896531	2A	472636282
AX-109083208	A/A	A/G	A/A	Affx-109780166	2A	286019963
AX-109084387	C/C	T/C	C/C	Affx-109607099	2A	312488660
AX-109085552	A/A	A/G	A/A	Affx-109701353	2A	237007335
AX-109086075	T/T	T/C	T/T	Affx-112003371	2A	297395295
AX-109086246	C/C	C/C	C/C	Affx-109548974	2A	196929547
AX-109099445	C/C	T/C	T/T	Affx-110453267	2A	481089738
AX-109104513	G/G	T/G	G/G	Affx-108954478	2A	465855094
AX-109270714	G/G	A/G	G/G	Affx-111106124	2A	118664684
AX-109272661	A/A	A/A	A/A	Affx-109136999	2A	500226380
AX-109273952	T/T	T/C	T/T	Affx-111855557	2A	21251869
AX-109274495	C/C	C/G	C/C	Affx-110518247	2A	735757681
AX-109275825	A/G	A/A	G/G	Affx-111617364	2A	676717419
AX-109277365	A/A	G/G	A/A	Affx-111005471	2A	221299291
AX-109279815	C/C	T/C	C/C	Affx-109953973	2A	456863991
AX-109280068	C/C	T/C	T/T	Affx-109913157	2A	522124547
AX-109280291	G/G	C/G	C/C	Affx-88419306	2A	604861056
AX-109280395	T/T	T/T	T/T	Affx-110806494	2A	570503553
AX-109286120	A/A	G/G	A/A	Affx-110584922	2A	469809657
AX-109286714	T/T	T/C	T/T	Affx-110138436	2A	562727585
AX-109287035	G/G	T/G	G/G	Affx-110117572	2A	134059822
AX-109290429	T/T	T/G	T/T	Affx-109396470	2A	608865127
AX-109294771	C/C	C/G	C/C	Affx-109248460	2A	79482551
AX-109298057	A/A	G/G	A/A	Affx-111547362	2A	502218856
AX-109298194	A/A	A/G	A/A	Affx-88640478	2A	178475267
AX-109299919	G/G	C/G	G/G	Affx-109239910	2A	377442281
AX-109302733	A/A	A/G	A/A	Affx-111455750	2A	430557237
AX-109302752	C/C	T/C	C/C	Affx-111943352	2A	369878910
AX-109303155	G/G	A/G	A/A	Affx-109212267	2A	605534683
AX-109304281	C/C	T/C	C/C	Affx-110734801	2A	40827600
AX-109304717	G/G	C/G	G/G	Affx-109691833	2A	254193062

AX-109304879	C/C	C/C	C/C	Affx-110920500	2A	542733297
AX-109306875	A/A	A/C	A/A	Affx-109779425	2A	728810807
AX-109312754	G/G	A/G	G/G	Affx-109968846	2A	120395466
AX-109314831	C/C	C/G	C/C	Affx-110099999	2A	92682662
AX-109319646	G/G	A/G	G/G	Affx-88446288	2A	740382718
AX-109319676	C/C	A/C	C/C	Affx-111116207	2A	305553289
AX-109320983	C/C	G/G	C/C	Affx-109489932	2A	365023487
AX-109324324	G/G	T/G	G/G	Affx-111931848	2A	294657516
AX-109325569	A/A	A/G	A/A	Affx-111422491	2A	121583709
AX-109325916	T/T	T/C	T/T	Affx-109406124	2A	382430088
AX-109328674	T/T	T/G	T/T	Affx-111531241	2A	367674408
AX-109330937	G/G	G/G	G/G	Affx-109215623	2A	755378747
AX-109336250	C/C	T/C	C/C	Affx-110995351	2A	82071397
AX-109339921	T/T	T/G	T/T	Affx-110221412	2A	617817164
AX-109342183	G/G	A/G	G/G	Affx-111741796	2A	444115720
AX-109344129	A/A	A/G	A/A	Affx-109707376	2A	717803976
AX-109346982	A/C	A/C	A/A	Affx-108876873	2A	702360003
AX-109353607	T/T	T/C	T/T	Affx-110621352	2A	249572887
AX-109356459	T/T	T/C	T/T	Affx-110213572	2A	475625022
AX-109356488	G/G	T/G	G/G	Affx-111845927	2A	203191300
AX-109357723	T/T	T/G	T/T	Affx-111487813	2A	154695499
AX-109360675	C/C	A/C	C/C	Affx-110481291	2A	728565588
AX-109361326	T/T	T/C	T/T	Affx-110744307	2A	103395469
AX-109361547	G/G	A/G	G/G	Affx-111120572	2A	300901931
AX-109368860	A/A	A/G	G/G	Affx-111301645	2A	671073632
AX-109371230	T/T	T/T	T/T	Affx-110418292	2A	198187756
AX-109371667	C/C	T/C	C/C	Affx-111150631	2A	489834523
AX-109374842	T/T	T/T	T/T	Affx-110014297	2A	557501278
AX-109375320	C/C	C/G	C/C	Affx-110345658	2A	396706485
AX-109376020	C/C	T/C	C/C	Affx-110058476	2A	560690433
AX-109376830	T/T	T/C	C/C	Affx-109214376	2A	484616747
AX-109384630	C/C	C/G	C/C	Affx-110404304	2A	758939697
AX-109384817	T/T	T/C	T/T	Affx-109990024	2A	235968232
AX-109394913	G/G	A/G	G/G	Affx-110266319	2A	142416204
AX-109395596	C/C	C/C	C/C	Affx-109173030	2A	157444912
AX-109408043	G/G	A/G	G/G	Affx-109104682	2A	230647633
AX-109408339	A/A	A/C	A/A	Affx-109655230	2A	228464851
AX-109414409	T/T	A/T	T/T	Affx-109143931	2A	76770764
AX-109415589	C/C	C/C	C/C	Affx-109143552	2A	727183765
AX-109419208	T/T	T/C	T/T	Affx-111608448	2A	555886644
AX-109422320	A/G	A/G	A/A	Affx-110139706	2A	719945932
AX-109425314	A/A	G/G	A/A	Affx-111379951	2A	693336312
AX-109429816	A/A	A/G	A/A	Affx-111384234	2A	538781298
AX-109435456	T/T	T/T	T/T	Affx-110496278	2A	706293432
AX-109437310	A/A	A/G	A/A	Affx-109071866	2A	86266686
AX-109448888	T/T	T/G	T/T	Affx-109659802	2A	381075962
AX-109449769	T/T	T/G	T/T	Affx-108990603	2A	73606037
AX-109452286	G/G	T/G	G/G	Affx-109514469	2A	525028734
AX-109453105	G/G	A/G	G/G	Affx-109563418	2A	505090339
AX-109456438	C/C	T/C	C/C	Affx-108898902	2A	554582895
AX-109459963	T/T	T/T	T/T	Affx-109880541	2A	270377095
AX-109460053	T/T	T/C	T/T	Affx-109575192	2A	367126408
AX-109463348	A/A	A/G	A/A	Affx-110107417	2A	567768912
AX-109463882	C/C	A/C	C/C	Affx-109043957	2A	345493685

AX-109467214	A/A	A/G	A/A	Affx-109592702	2A	512230192
AX-109467781	A/A	A/G	A/A	Affx-110467351	2A	222607461
AX-109468797	C/C	C/G	C/C	Affx-110013030	2A	250023670
AX-109469762	G/G	T/G	G/G	Affx-110699722	2A	565744636
AX-109470982	A/A	G/G	A/A	Affx-110765849	2A	342658773
AX-109475595	A/A	A/G	A/A	Affx-111447164	2A	45119859
AX-109475658	G/G	G/G	G/G	Affx-88620359	2A	214447864
AX-109484752	T/T	T/T	C/C	Affx-111160800	2A	510364374
AX-109491828	G/G	G/G	G/G	Affx-111414658	2A	140360386
AX-109492818	T/T	T/C	T/T	Affx-110508622	2A	566443098
AX-109492934	G/G	A/G	G/G	Affx-111908629	2A	438100843
AX-109495816	A/A	A/G	A/A	Affx-111187298	2A	429331510
AX-109496411	G/G	A/G	G/G	Affx-112004534	2A	239819167
AX-109501778	T/T	T/G	T/T	Affx-88722423	2A	779821335
AX-109502752	G/G	G/G	G/G	Affx-88788905	2A	99129390
AX-109509615	G/G	T/G	G/G	Affx-110269611	2A	100188441
AX-109516561	A/G	A/A	A/G	Affx-109517715	2A	113926361
AX-109517867	C/C	C/G	C/C	Affx-110229922	2A	313014943
AX-109520848	G/G	A/G	G/G	Affx-110698901	2A	102285615
AX-109521183	A/A	G/G	A/A	Affx-109692260	2A	402685194
AX-109531973	G/G	A/G	G/G	Affx-111910100	2A	350700340
AX-109548035	C/C	T/C	T/T	Affx-110664750	2A	516702974
AX-109559783	G/G	A/G	G/G	Affx-108996553	2A	218209331
AX-109568335	A/A	A/T	A/A	Affx-111617179	2A	46346287
AX-109576757	C/C	T/C	C/C	Affx-110808527	2A	751912827
AX-109579697	C/C	C/G	C/C	Affx-111078673	2A	267685478
AX-109585515	A/A	A/G	A/A	Affx-88377201	2A	350937796
AX-109585688	T/T	T/C	T/T	Affx-111841698	2A	538405337
AX-109586321	G/G	A/G	G/G	Affx-110254284	2A	183810440
AX-109586368	T/T	T/C	T/T	Affx-109187707	2A	426385502
AX-109588929	G/G	A/G	G/G	Affx-110216855	2A	444608820
AX-109599637	A/A	G/G	A/A	Affx-111054572	2A	315321995
AX-109616106	A/A	A/G	A/A	Affx-109705329	2A	247731603
AX-109619678	A/A	A/G	A/A	Affx-111722197	2A	360866802
AX-109622666	T/T	T/C	T/T	Affx-111629104	2A	499217931
AX-109624083	T/T	T/C	T/T	Affx-109410341	2A	571429016
AX-109624356	A/A	A/G	A/A	Affx-109804844	2A	334317011
AX-109627137	G/G	T/G	G/G	Affx-88418576	2A	463734951
AX-109627720	T/T	T/T	T/T	Affx-109449994	2A	406447770
AX-109630363	C/C	T/C	C/C	Affx-110445034	2A	473326088
AX-109640532	T/C	T/T	T/T	Affx-110058444	2A	470039936
AX-109815538	G/G	C/G	G/G	Affx-111886623	2A	328566925
AX-109822798	G/G	G/G	G/G	Affx-110004842	2A	709153364
AX-109826841	G/G	G/G	G/G	Affx-88629988	2A	132867433
AX-109830632	G/G	A/G	G/G	Affx-111618455	2A	202257479
AX-109832463	A/A	A/G	A/A	Affx-109808139	2A	358906450
AX-109834294	A/A	A/G	A/A	Affx-109291175	2A	135593270
AX-109837902	T/T	T/C	T/T	Affx-109981376	2A	235266213
AX-109838371	G/G	A/G	G/G	Affx-111541625	2A	347373658
AX-109844516	C/C	T/C	C/C	Affx-111599181	2A	84051959
AX-109847055	C/C	T/C	C/C	Affx-110266229	2A	320889791
AX-109850613	T/T	T/C	T/T	Affx-109755933	2A	273985053
AX-109852228	A/G	A/A	A/A	Affx-88668334	2A	709820814
AX-109855643	A/A	A/G	A/A	Affx-109694446	2A	379504214

AX-109857803	A/A	A/G	A/A	Affx-109103434	2A	541386617
AX-109858272	A/A	A/G	A/A	Affx-110294764	2A	279843475
AX-109861551	A/A	A/G	A/A	Affx-110693527	2A	387045348
AX-109866206	C/G	C/C	C/C	Affx-88396723	2A	677352227
AX-109867478	T/T	T/C	T/T	Affx-110268771	2A	81494051
AX-109869056	C/C	C/G	C/C	Affx-108878229	2A	268611216
AX-109869548	C/C	A/C	C/C	Affx-111503730	2A	426779407
AX-109869866	T/T	T/C	T/T	Affx-88507415	2A	178465988
AX-109871489	A/A	A/G	A/A	Affx-109017349	2A	309419476
AX-109872000	C/C	T/C	C/C	Affx-110391048	2A	380566504
AX-109873677	A/A	A/G	A/A	Affx-110675095	2A	307673861
AX-109873869	C/C	T/C	C/C	Affx-110685219	2A	341077669
AX-109877670	T/T	T/T	T/T	Affx-111928325	2A	220358977
AX-109879152	A/A	A/G	G/G	Affx-110583589	2A	531555997
AX-109880231	C/C	A/A	C/C	Affx-109138804	2A	3799547
AX-109884562	A/A	A/G	A/A	Affx-109122992	2A	250873331
AX-109885140	C/C	T/C	C/C	Affx-110148277	2A	725263317
AX-109887498	A/A	A/G	A/A	Affx-110050401	2A	621852583
AX-109888163	C/C	T/C	C/C	Affx-110491021	2A	115139933
AX-109892024	T/T	T/C	T/T	Affx-110320844	2A	289074697
AX-109894450	A/G	G/G	G/G	Affx-111205470	2A	722527045
AX-109895548	C/C	T/C	C/C	Affx-109481283	2A	125268294
AX-109900195	C/C	T/C	T/T	Affx-110743447	2A	477481645
AX-109905049	T/T	T/C	T/T	Affx-109591024	2A	305011108
AX-109905124	C/C	T/C	C/C	Affx-111960056	2A	54708777
AX-109905354	T/C	C/C	C/C	Affx-108928288	2A	745671770
AX-109909628	A/A	A/G	A/A	Affx-111865481	2A	212597210
AX-109913197	G/G	C/G	G/G	Affx-88605208	2A	585141659
AX-109914824	G/G	A/G	G/G	Affx-110986840	2A	194415959
AX-109920901	G/G	A/G	G/G	Affx-109181653	2A	304095417
AX-109927143	A/A	G/G	A/A	Affx-109769657	2A	390151013
AX-109927461	C/C	C/C	C/C	Affx-109009960	2A	556953839
AX-109930556	A/A	A/G	A/A	Affx-109543842	2A	393112120
AX-109931170	T/T	T/C	T/T	Affx-110998796	2A	261640389
AX-109932432	C/C	T/T	C/C	Affx-111449952	2A	280046999
AX-109934830	A/A	A/G	A/A	Affx-88620026	2A	227629371
AX-109940674	A/A	A/G	G/G	Affx-110466423	2A	36193231
AX-109941294	G/G	C/G	C/C	Affx-109974861	2A	478116638
AX-109946432	T/G	G/G	T/T	Affx-111139403	2A	729772311
AX-109952008	T/T	T/C	T/T	Affx-109992087	2A	427516883
AX-109956214	T/T	T/C	T/T	Affx-111688084	2A	279221118
AX-109958037	G/G	T/G	G/G	Affx-111708968	2A	454511057
AX-109963316	T/T	T/C	C/C	Affx-111445484	2A	605379330
AX-109964750	C/C	T/C	T/T	Affx-111473952	2A	28136415
AX-109965731	A/A	A/G	A/G	Affx-110470421	2A	49657167
AX-109969172	G/G	A/G	G/G	Affx-109929895	2A	150913149
AX-109973196	G/G	T/G	G/G	Affx-88797221	2A	430704269
AX-109973577	A/A	A/G	A/A	Affx-88695721	2A	209395020
AX-109979106	G/G	G/G	G/G	Affx-108862331	2A	383325806
AX-109980094	A/A	A/G	A/A	Affx-109944186	2A	230409121
AX-109982523	T/T	T/C	T/T	Affx-110838616	2A	464834490
AX-109987891	T/T	T/T	T/T	Affx-109990695	2A	273514923
AX-109988368	A/A	A/G	A/A	Affx-109865235	2A	539005515
AX-109989452	A/A	A/G	G/G	Affx-111821461	2A	501287611

AX-109996565	A/A	A/G	G/G	Affx-111685292	2A	675292214
AX-109999226	T/C	T/C	T/T	Affx-109159863	2A	698090161
AX-110002114	A/A	A/G	A/A	Affx-111060683	2A	245404879
AX-110017836	A/A	A/G	A/A	Affx-110378449	2A	277616177
AX-110019663	T/T	T/C	T/T	Affx-109588942	2A	303100699
AX-110021916	G/G	A/G	G/G	Affx-109148127	2A	201794988
AX-110026721	A/A	A/G	A/A	Affx-108872365	2A	34322083
AX-110036265	C/C	T/C	C/C	Affx-111353902	2A	39896043
AX-110042217	T/T	T/T	T/T	Affx-111227947	2A	111421060
AX-110043968	G/G	A/G	G/G	Affx-110673701	2A	500706298
AX-110048469	A/A	A/G	A/A	Affx-109142263	2A	268474156
AX-110049033	G/G	A/G	G/G	Affx-108934364	2A	234040055
AX-110049660	C/C	T/C	C/C	Affx-109222460	2A	770262196
AX-110054856	C/C	T/C	C/C	Affx-111793379	2A	153094292
AX-110055832	C/G	C/C	C/G	Affx-109417289	2A	371706997
AX-110059104	C/C	T/T	C/C	Affx-109820647	2A	300354063
AX-110059855	A/A	G/G	A/A	Affx-110741257	2A	216822516
AX-110060090	T/T	T/C	T/T	Affx-111551123	2A	376976024
AX-110067600	T/C	T/T	C/C	Affx-110793316	2A	66848383
AX-110068305	A/A	A/C	A/A	Affx-109498562	2A	445576073
AX-110071357	A/A	A/G	A/A	Affx-111376692	2A	50961104
AX-110084490	G/G	A/G	G/G	Affx-111871025	2A	93926994
AX-110088938	T/T	C/C	T/T	Affx-110749095	2A	73158478
AX-110094355	A/A	A/T	T/T	Affx-110520454	2A	532853140
AX-110105548	T/T	A/T	T/T	Affx-88347425	2A	146954804
AX-110107350	T/T	T/G	T/T	Affx-109122325	2A	322831689
AX-110109766	C/C	T/C	C/C	Affx-109303185	2A	733920848
AX-110120699	T/T	T/C	T/T	Affx-88492100	2A	257782439
AX-110133293	G/G	C/C	G/G	Affx-110446475	2A	769460727
AX-110139178	A/A	A/G	G/G	Affx-110469445	2A	675735185
AX-110147247	T/T	G/G	T/T	Affx-111976541	2A	234983329
AX-110147472	T/T	T/C	T/T	Affx-109805611	2A	208236100
AX-110151409	G/G	A/G	G/G	Affx-110001309	2A	76282095
AX-110153178	A/A	G/G	A/A	Affx-109935550	2A	274507199
AX-110167860	A/G	A/G	G/G	Affx-110306611	2A	720800649
AX-110168387	A/A	A/A	G/G	Affx-109525273	2A	604129562
AX-110169699	G/G	G/G	G/G	Affx-109145509	2A	37034485
AX-110173514	T/T	T/C	T/T	Affx-111009014	2A	221829832
AX-110173580	G/G	G/G	G/G	Affx-111605493	2A	697044628
AX-110174582	C/C	T/C	C/C	Affx-110404561	2A	284251431
AX-110181609	G/G	A/G	G/G	Affx-109015969	2A	95318478
AX-110185039	G/G	A/G	G/G	Affx-109425716	2A	260305542
AX-110191562	G/G	G/G	G/G	Affx-111469143	2A	728595656
AX-110194285	T/T	T/C	T/T	Affx-111974009	2A	271385488
AX-110196019	A/A	A/C	A/A	Affx-109654740	2A	252636564
AX-110201207	A/A	A/C	A/A	Affx-110609135	2A	536199735
AX-110362480	G/G	G/G	G/G	Affx-110658015	2A	579442018
AX-110363192	T/T	T/C	T/T	Affx-109839088	2A	269744080
AX-110364988	A/A	G/G	G/G	Affx-109301644	2A	41460965
AX-110366552	G/G	A/G	G/G	Affx-110166563	2A	753693013
AX-110366863	G/G	A/G	A/G	Affx-111657811	2A	699327822
AX-110368854	A/A	A/G	A/A	Affx-110051099	2A	733268129
AX-110370613	C/C	A/C	C/C	Affx-109779026	2A	558536561
AX-110371172	C/C	T/C	C/C	Affx-110033826	2A	309720902

AX-110374006	C/C	T/C	C/C	Affx-109265590	2A	330647830
AX-110378983	G/G	A/G	G/G	Affx-111901298	2A	155773444
AX-110380823	G/G	A/G	G/G	Affx-111565627	2A	434985498
AX-110382047	A/A	A/G	A/A	Affx-110700035	2A	378597899
AX-110386046	A/A	A/G	A/A	Affx-109119921	2A	389468397
AX-110394261	T/T	T/C	T/T	Affx-110304139	2A	123173960
AX-110400114	A/A	A/G	A/A	Affx-111897873	2A	242410318
AX-110401567	T/T	C/C	T/T	Affx-111627680	2A	765572135
AX-110401789	T/T	T/C	T/T	Affx-111145631	2A	536230998
AX-110405613	C/C	T/C	C/C	Affx-109082449	2A	77275669
AX-110406457	C/C	C/G	C/C	Affx-111691479	2A	184498897
AX-110407291	C/C	T/C	T/T	Affx-111644987	2A	603732041
AX-110408259	G/G	C/G	C/G	Affx-111291234	2A	282247129
AX-110410190	G/G	A/G	G/G	Affx-109341255	2A	754182070
AX-110410791	A/A	A/G	A/A	Affx-110485579	2A	322120520
AX-110410792	G/G	A/G	G/G	Affx-110471818	2A	503441606
AX-110411637	T/T	T/C	T/T	Affx-110381601	2A	68488793
AX-110412549	G/G	A/G	G/G	Affx-109356436	2A	204190724
AX-110412734	C/C	C/G	G/G	Affx-111467155	2A	199795124
AX-110414293	T/T	T/C	T/T	Affx-110708793	2A	407580375
AX-110416654	C/C	C/G	C/C	Affx-110308740	2A	756836682
AX-110417428	T/T	T/C	T/T	Affx-111368396	2A	324334935
AX-110418781	A/C	A/C	C/C	Affx-111901533	2A	714728390
AX-110420403	G/G	C/G	G/G	Affx-109074194	2A	70032576
AX-110422707	T/T	T/C	T/T	Affx-111115112	2A	157543621
AX-110424435	C/C	A/C	C/C	Affx-111810540	2A	44507449
AX-110425132	C/C	C/G	G/G	Affx-109147393	2A	705807076
AX-110428035	C/C	C/G	G/G	Affx-111111439	2A	690748100
AX-110429464	A/A	A/C	A/A	Affx-110712616	2A	715290882
AX-110430948	A/A	A/C	A/A	Affx-111439262	2A	278535731
AX-110434181	A/A	A/G	A/A	Affx-110881425	2A	388631671
AX-110435756	G/G	A/G	G/G	Affx-111277798	2A	25940996
AX-110438624	T/T	T/C	T/T	Affx-110454991	2A	630790510
AX-110439763	C/C	A/C	A/A	Affx-109192732	2A	474792453
AX-110448758	C/C	C/C	C/C	Affx-111200343	2A	741582244
AX-110450470	C/C	T/C	C/C	Affx-109830987	2A	95989678
AX-110451187	C/C	C/C	C/C	Affx-111483487	2A	607768697
AX-110457326	A/A	A/G	A/A	Affx-111691657	2A	410445224
AX-110459357	T/T	T/C	T/T	Affx-110386551	2A	379251758
AX-110461158	T/C	T/C	T/T	Affx-111966949	2A	712182754
AX-110463753	G/G	G/G	G/G	Affx-110768998	2A	708670097
AX-110471740	A/A	A/C	C/C	Affx-111422517	2A	202783752
AX-110471961	T/T	T/C	T/T	Affx-110371513	2A	143549655
AX-110472753	T/T	T/C	T/T	Affx-108870294	2A	227266911
AX-110477514	C/C	T/C	C/C	Affx-111214527	2A	292467640
AX-110478420	A/A	A/G	A/A	Affx-109727387	2A	207963097
AX-110484064	T/T	T/T	T/T	Affx-109278039	2A	148875653
AX-110484643	C/C	T/C	C/C	Affx-110007456	2A	27908336
AX-110485184	T/T	T/C	T/T	Affx-109704737	2A	211904429
AX-110489311	G/G	A/G	G/G	Affx-111827447	2A	360612136
AX-110490034	G/G	A/G	A/A	Affx-109809689	2A	39322557
AX-110490284	G/G	A/G	G/G	Affx-110992493	2A	298956856
AX-110494827	T/C	T/C	T/T	Affx-109434878	2A	697416051
AX-110503212	T/T	T/C	T/T	Affx-109424795	2A	368761413

AX-110503320	T/T	T/C	T/T	Affx-110877473	2A	405133994
AX-110507459	G/G	T/G	G/G	Affx-108892839	2A	417633392
AX-110510308	T/T	T/C	T/T	Affx-111651271	2A	611567265
AX-110515487	A/A	A/G	A/G	Affx-110580505	2A	486841595
AX-110516001	G/G	G/G	G/G	Affx-109692651	2A	104298168
AX-110522750	T/T	T/G	T/T	Affx-111447717	2A	89734060
AX-110525818	T/T	T/C	T/T	Affx-111492498	2A	466404784
AX-110526044	G/G	A/G	G/G	Affx-109389927	2A	105479971
AX-110530076	G/G	T/G	G/G	Affx-111659324	2A	225571820
AX-110530347	C/C	T/C	C/C	Affx-110529241	2A	491530184
AX-110530356	G/G	A/G	G/G	Affx-111029130	2A	695435145
AX-110536509	T/T	T/T	C/C	Affx-109267491	2A	506591684
AX-110538140	T/T	T/G	G/G	Affx-111056958	2A	622396527
AX-110541525	T/T	T/C	T/T	Affx-109817021	2A	96639805
AX-110543101	C/C	T/C	C/C	Affx-111658505	2A	226648684
AX-110545682	A/A	A/A	A/A	Affx-111366254	2A	107939992
AX-110547058	A/A	A/C	A/A	Affx-111956819	2A	400673727
AX-110548395	A/A	A/A	A/A	Affx-111633576	2A	563167478
AX-110548472	G/G	A/G	A/A	Affx-110973596	2A	474288952
AX-110552326	G/G	C/G	G/G	Affx-88635889	2A	245823157
AX-110552715	G/G	A/G	A/A	Affx-110136728	2A	509713231
AX-110555632	C/C	T/C	T/T	Affx-109410737	2A	59751671
AX-110555924	C/C	T/C	C/C	Affx-109925946	2A	352040466
AX-110555957	T/C	T/C	C/C	Affx-109198073	2A	679875877
AX-110556767	G/G	G/G	G/G	Affx-109092078	2A	7843115
AX-110559582	G/G	A/G	G/G	Affx-88460590	2A	366411621
AX-110563624	A/A	C/C	A/A	Affx-110036365	2A	349657251
AX-110564463	T/T	T/T	T/T	Affx-109063216	2A	71100411
AX-110566050	G/G	G/G	G/G	Affx-110925114	2A	56866848
AX-110566442	T/T	T/G	T/T	Affx-111579546	2A	442443938
AX-110572142	G/G	C/G	G/G	Affx-111859123	2A	316503774
AX-110578550	G/G	G/G	T/G	Affx-111210201	2A	518736789
AX-110582375	C/C	T/C	C/C	Affx-111264731	2A	244815308
AX-110583742	A/A	A/G	A/A	Affx-110736691	2A	216476485
AX-110583762	A/A	A/C	A/A	Affx-109143852	2A	260961798
AX-110585397	T/T	A/T	T/T	Affx-110298083	2A	234406258
AX-110591194	T/T	T/C	T/T	Affx-111467867	2A	393587786
AX-110591479	T/T	A/T	A/A	Affx-110561463	2A	502890353
AX-110599729	A/A	A/G	G/G	Affx-110178054	2A	521421510
AX-110600847	T/T	C/C	T/T	Affx-109965144	2A	751003420
AX-110602138	G/G	C/G	C/C	Affx-111246104	2A	699575539
AX-110602968	T/T	T/C	T/T	Affx-110023129	2A	44160170
AX-110604032	G/G	A/G	A/A	Affx-109844573	2A	491864078
AX-110609000	C/C	T/C	C/C	Affx-111517051	2A	335211309
AX-110624465	C/C	T/C	C/C	Affx-109353585	2A	751539653
AX-110626001	T/C	T/T	C/C	Affx-110199604	2A	135199508
AX-110632246	C/C	C/C	C/C	Affx-110107174	2A	207487603
AX-110634829	C/C	A/C	C/C	Affx-110426855	2A	98121230
AX-110643098	C/C	C/C	C/C	Affx-110417777	2A	75645645
AX-110644055	A/A	G/G	A/A	Affx-111900783	2A	252096017
AX-110651741	A/A	A/G	A/A	Affx-110102976	2A	115714343
AX-110671887	A/A	A/G	A/A	Affx-110448189	2A	424285375
AX-110672429	C/C	A/C	C/C	Affx-109608395	2A	641597655
AX-110673342	C/C	T/C	C/C	Affx-111580894	2A	239089251

AX-110675258	G/G	C/G	G/G	Affx-109035498	2A	447357578
AX-110686156	C/C	T/C	C/C	Affx-109252104	2A	779185155
AX-110698025	G/G	A/A	G/G	Affx-111450879	2A	186357528
AX-110698294	A/A	A/G	A/A	Affx-111333280	2A	251671558
AX-110714623	T/T	T/C	T/T	Affx-111356277	2A	379935646
AX-110717327	A/A	A/G	A/A	Affx-110351325	2A	123454504
AX-110742192	T/T	T/C	T/T	Affx-110512703	2A	219835497
AX-110744451	A/A	A/G	A/A	Affx-111043359	2A	283287222
AX-110904713	C/C	T/C	T/T	Affx-109414629	2A	32456864
AX-110906351	G/G	C/G	G/G	Affx-110852508	2A	151365203
AX-110909648	A/A	A/G	A/A	Affx-110444305	2A	383744810
AX-110921635	G/G	C/G	G/G	Affx-109733578	2A	125607419
AX-110925974	A/A	A/G	A/A	Affx-110015299	2A	246345023
AX-110927273	A/A	A/G	A/A	Affx-108912566	2A	229334651
AX-110927984	G/G	A/G	G/G	Affx-111565150	2A	275353029
AX-110931139	G/G	C/G	G/G	Affx-110550941	2A	533502163
AX-110936572	T/T	T/C	C/C	Affx-88341983	2A	526344402
AX-110939807	T/T	T/C	T/T	Affx-110647352	2A	314183801
AX-110943070	C/C	C/C	C/C	Affx-110570190	2A	195069118
AX-110945780	C/C	A/C	C/C	Affx-109544760	2A	375058192
AX-110948179	A/A	A/A	A/A	Affx-111998795	2A	738974772
AX-110951305	C/C	A/C	A/A	Affx-110428074	2A	473729432
AX-110953884	A/A	A/A	A/A	Affx-109555342	2A	541773562
AX-110957267	C/C	G/G	C/C	Affx-109380778	2A	756276818
AX-110957721	T/T	T/C	T/T	Affx-111480172	2A	52582317
AX-110960394	T/T	T/C	T/T	Affx-109621741	2A	116346354
AX-110962340	G/G	T/G	T/T	Affx-111021659	2A	616149567
AX-110970966	T/T	T/C	T/T	Affx-111613266	2A	412852918
AX-110972463	T/T	T/C	T/T	Affx-109686589	2A	83190989
AX-110975737	T/T	T/G	T/G	Affx-110045847	2A	531144581
AX-110976760	C/C	T/C	C/C	Affx-109200080	2A	391542360
AX-110983898	A/G	A/G	G/G	Affx-110774742	2A	641084037
AX-110986480	T/T	T/C	T/T	Affx-111098625	2A	27328559
AX-110988671	A/A	A/G	A/A	Affx-88359728	2A	746687013
AX-110990373	G/G	C/G	G/G	Affx-111821161	2A	480691588
AX-110996940	T/T	T/C	T/T	Affx-111184890	2A	541010682
AX-111002498	C/C	T/C	C/C	Affx-109534513	2A	621776014
AX-111004768	T/T	G/G	T/T	Affx-111904135	2A	452477259
AX-111007109	T/T	T/C	T/T	Affx-109710598	2A	384230185
AX-111007235	T/T	T/G	T/T	Affx-109691651	2A	53792742
AX-111012954	G/G	A/G	G/G	Affx-109769136	2A	290059635
AX-111013049	A/A	A/G	A/A	Affx-110360820	2A	100667546
AX-111013754	G/G	A/G	G/G	Affx-111551863	2A	776038161
AX-111016073	A/A	A/C	A/C	Affx-110507577	2A	777949563
AX-111017807	T/T	T/C	T/T	Affx-88417597	2A	65819322
AX-111018581	C/C	T/C	C/C	Affx-109497989	2A	424010865
AX-111019035	A/A	A/G	A/A	Affx-109256900	2A	357809535
AX-111021682	C/C	T/C	C/C	Affx-110254256	2A	295562443
AX-111021698	G/G	C/G	G/G	Affx-110848076	2A	153532042
AX-111023281	G/G	A/G	G/G	Affx-108957659	2A	411473841
AX-111026642	T/T	T/C	T/T	Affx-110291016	2A	253145258
AX-111027219	T/T	T/C	T/T	Affx-109662466	2A	343626409
AX-111032286	T/T	T/C	T/T	Affx-110959451	2A	144429850
AX-111033486	A/A	G/G	A/A	Affx-110196537	2A	358391597

AX-111035281	A/A	A/G	A/A	Affx-110325342	2A	370945663
AX-111037738	C/C	T/C	C/C	Affx-109287308	2A	436213047
AX-111038917	C/C	C/G	C/C	Affx-110721837	2A	348904233
AX-111039042	A/G	A/A	A/G	Affx-88796205	2A	754646198
AX-111040521	T/T	T/T	T/T	Affx-88781652	2A	97844535
AX-111051222	A/A	A/G	A/A	Affx-111641996	2A	333010799
AX-111051255	A/A	A/G	A/A	Affx-110440023	2A	211479871
AX-111052252	T/T	T/C	T/T	Affx-111532085	2A	317737126
AX-111054566	A/G	A/A	G/G	Affx-88604712	2A	144652972
AX-111056196	A/A	A/G	A/A	Affx-111791047	2A	101309153
AX-111057162	G/G	A/G	G/G	Affx-110523850	2A	218933005
AX-111057189	A/A	A/G	A/A	Affx-110622682	2A	59163482
AX-111063614	T/T	C/C	T/C	Affx-111411321	2A	20560281
AX-111064589	G/G	A/G	A/G	Affx-110670278	2A	31501225
AX-111065350	G/G	A/G	G/G	Affx-110881560	2A	712199617
AX-111065497	A/A	A/A	A/A	Affx-110844494	2A	318784905
AX-111066910	G/G	A/G	G/G	Affx-109183638	2A	141283511
AX-111068711	G/G	A/G	G/G	Affx-111373755	2A	381677023
AX-111072317	A/A	A/G	A/A	Affx-111130778	2A	429331078
AX-111074204	A/A	A/G	A/A	Affx-110844124	2A	136330264
AX-111079268	C/C	T/C	T/T	Affx-111557934	2A	62210787
AX-111083554	T/T	T/C	T/T	Affx-109222056	2A	347743656
AX-111086203	C/C	T/C	C/C	Affx-109690812	2A	231890154
AX-111090636	T/T	T/C	T/T	Affx-109302307	2A	221077674
AX-111091913	C/C	T/C	C/C	Affx-109165930	2A	406659995
AX-111097048	C/C	T/C	C/C	Affx-111538731	2A	410801735
AX-111102740	A/A	A/G	A/A	Affx-108933383	2A	638532972
AX-111103541	C/C	C/G	C/C	Affx-109588177	2A	411730746
AX-111108642	G/G	T/G	G/G	Affx-109534541	2A	297841617
AX-111115396	C/C	T/C	C/C	Affx-111135454	2A	148299057
AX-111122997	T/T	T/C	C/C	Affx-111916331	2A	508022370
AX-111130517	A/A	A/G	A/A	Affx-109403894	2A	414981751
AX-111132644	T/T	C/C	T/T	Affx-111928489	2A	224224002
AX-111132810	G/G	A/G	G/G	Affx-111145850	2A	42155602
AX-111135521	C/C	T/T	C/C	Affx-109687236	2A	599161484
AX-111136195	C/C	T/C	C/C	Affx-109261175	2A	293307020
AX-111139916	C/C	C/C	C/C	Affx-109729710	2A	407859024
AX-111140886	T/T	T/C	T/T	Affx-111935298	2A	183502393
AX-111141805	T/T	T/C	C/C	Affx-110594153	2A	718336567
AX-111146633	C/C	T/C	C/C	Affx-111467623	2A	721600701
AX-111147496	G/G	C/G	G/G	Affx-109570059	2A	199431055
AX-111148504	G/G	T/G	T/T	Affx-111379219	2A	485122009
AX-111155519	T/T	T/C	T/T	Affx-109315927	2A	435178900
AX-111156124	C/C	T/C	C/C	Affx-110435475	2A	524691482
AX-111156458	A/A	A/G	A/A	Affx-109428173	2A	291633988
AX-111167599	C/C	C/G	C/C	Affx-88630726	2A	83847793
AX-111183138	A/A	A/A	A/A	Affx-88725862	2A	36934685
AX-111184907	A/A	A/G	A/G	Affx-109756868	2A	60713662
AX-111191799	C/C	T/C	C/C	Affx-110063185	2A	33051366
AX-111193925	A/A	A/G	A/A	Affx-111565365	2A	130716187
AX-111217913	G/G	A/G	A/G	Affx-109658833	2A	248428999
AX-111219462	G/G	A/G	G/G	Affx-110146211	2A	367541178
AX-111220378	G/G	G/G	G/G	Affx-109961199	2A	225728270
AX-111233813	A/A	A/G	A/A	Affx-110208040	2A	700080368

AX-111235185	C/C	T/C	C/C	Affx-111212559	2A	69017760
AX-111241893	A/A	A/G	A/A	Affx-110846493	2A	452311371
AX-111242166	A/A	G/G	A/A	Affx-111895119	2A	240936661
AX-111242459	C/C	T/C	C/C	Affx-111652294	2A	232434748
AX-111243448	A/A	A/G	A/A	Affx-111041243	2A	346301375
AX-111250334	G/G	A/G	A/A	Affx-109573055	2A	516293673
AX-111252418	A/C	A/A	A/C	Affx-110809180	2A	366070573
AX-111262091	T/T	C/C	T/T	Affx-109231386	2A	425864793
AX-111262343	T/T	A/T	T/T	Affx-111292678	2A	295211296
AX-111262818	A/A	A/G	A/A	Affx-110032394	2A	344171302
AX-111264284	T/T	T/C	T/T	Affx-109205653	2A	348482235
AX-111275119	T/T	T/G	T/T	Affx-109360746	2A	370886133
AX-111282949	A/A	A/G	A/A	Affx-109716028	2A	317102250
AX-111284810	C/C	C/C	C/C	Affx-110650867	2A	308911448
AX-111286508	T/T	T/C	T/T	Affx-110058623	2A	324010732
AX-111288309	T/T	T/C	T/T	Affx-109780387	2A	284264404
AX-111452392	T/T	T/C	C/C	Affx-111709102	2A	677051371
AX-111453699	G/G	C/G	C/C	Affx-92133769	2A	693285987
AX-111456048	T/T	T/C	T/T	Affx-109187126	2A	206403326
AX-111459334	T/T	T/C	T/T	Affx-109474320	2A	240786893
AX-111461384	C/C	T/C	T/T	Affx-109049438	2A	523184732
AX-111462442	T/T	T/G	T/T	Affx-110480516	2A	428183518
AX-111463139	A/A	A/G	A/A	Affx-108861569	2A	192212882
AX-111464925	G/G	G/G	G/G	Affx-110652743	2A	256799575
AX-111467132	C/C	T/C	T/T	Affx-109523670	2A	713190960
AX-111470472	C/C	T/C	C/C	Affx-88402482	2A	182913782
AX-111470590	C/C	C/G	C/C	Affx-110127854	2A	213011797
AX-111473626	G/G	C/G	G/G	Affx-109407414	2A	560214941
AX-111477441	G/G	T/G	G/G	Affx-111163503	2A	420647543
AX-111480932	T/C	T/C	C/C	Affx-88618351	2A	685986891
AX-111484682	C/C	C/G	C/C	Affx-110846797	2A	275017870
AX-111490124	C/C	T/C	C/C	Affx-110916210	2A	359440463
AX-111502994	A/A	A/G	A/A	Affx-109513268	2A	468931814
AX-111505518	T/T	T/C	T/T	Affx-110195162	2A	565156466
AX-111508694	G/G	A/G	G/G	Affx-109826564	2A	349699837
AX-111508962	A/A	A/C	A/A	Affx-111407405	2A	288124225
AX-111512123	C/C	T/C	C/C	Affx-111513012	2A	372332314
AX-111522943	C/C	A/C	C/C	Affx-110707115	2A	334822241
AX-111523061	G/G	C/G	G/G	Affx-111028904	2A	128507219
AX-111530205	T/T	T/C	T/T	Affx-111169779	2A	238781909
AX-111534288	T/T	T/C	T/T	Affx-110752073	2A	205339018
AX-111536655	C/C	C/G	C/C	Affx-109998888	2A	395148235
AX-111543324	T/T	T/C	T/T	Affx-110765794	2A	562416065
AX-111543568	G/G	C/C	G/G	Affx-110641778	2A	315976655
AX-111545880	G/G	A/G	G/G	Affx-109927339	2A	287460537
AX-111545910	T/T	T/C	T/T	Affx-111923776	2A	50573778
AX-111546236	C/C	T/C	C/C	Affx-109810013	2A	561592294
AX-111551108	A/A	A/G	A/A	Affx-109058395	2A	387920022
AX-111551574	G/G	G/G	G/G	Affx-111999804	2A	152020327
AX-111553006	T/T	T/C	T/T	Affx-109090660	2A	471752184
AX-111557864	C/C	C/C	T/T	Affx-110690323	2A	7720330
AX-111558028	A/A	A/G	A/A	Affx-109197185	2A	238022903
AX-111561373	G/G	C/G	G/G	Affx-109913855	2A	242813032
AX-111566257	T/T	T/C	T/T	Affx-108927831	2A	126864122

AX-111567066	G/G	A/G	G/G	Affx-110821700	2A	193864024
AX-111567270	T/T	T/C	T/T	Affx-110160307	2A	257359568
AX-111568841	T/T	T/C	T/T	Affx-111625423	2A	465068029
AX-111570872	G/G	C/G	G/G	Affx-110308825	2A	124363723
AX-111572097	T/T	C/C	T/T	Affx-109905791	2A	266236659
AX-111576466	T/T	C/C	T/T	Affx-88582344	2A	606521847
AX-111578463	G/G	A/G	G/G	Affx-88805815	2A	280779370
AX-111581418	A/A	A/G	A/A	Affx-110963941	2A	249254331
AX-111587627	A/A	A/G	A/A	Affx-111655693	2A	551737758
AX-111601550	G/G	C/G	G/G	Affx-88392645	2A	69348846
AX-111602144	T/T	T/G	T/T	Affx-109358454	2A	409910791
AX-111605098	T/C	T/C	C/C	Affx-109668709	2A	712184069
AX-111605423	T/T	T/C	T/T	Affx-110079171	2A	325296511
AX-111611118	G/G	C/G	G/G	Affx-108861237	2A	722065099
AX-111614940	C/C	C/C	C/C	Affx-111892817	2A	342187163
AX-111621295	A/A	A/G	A/A	Affx-109223628	2A	282708692
AX-111625107	C/C	T/C	C/C	Affx-111019639	2A	728702106
AX-111628094	T/T	T/T	T/C	Affx-109904026	2A	487754918
AX-111630220	A/A	G/G	A/A	Affx-110427490	2A	392442570
AX-111635700	T/T	T/C	T/T	Affx-109759873	2A	122005612
AX-111635844	C/C	T/T	C/C	Affx-109406926	2A	289457436
AX-111637353	G/G	C/G	G/G	Affx-109264006	2A	640294640
AX-111640814	C/C	A/A	C/C	Affx-109349123	2A	507539588
AX-111640815	C/C	T/T	C/C	Affx-109760669	2A	441208448
AX-111641006	C/C	T/C	C/C	Affx-110545856	2A	281752126
AX-111643530	T/T	T/C	T/T	Affx-111942160	2A	388352119
AX-111644814	A/A	A/G	G/G	Affx-111565052	2A	520037431
AX-111645780	C/C	T/C	C/C	Affx-111056794	2A	513160239
AX-111650354	T/T	T/C	T/T	Affx-108881118	2A	408734620
AX-111652625	T/T	T/T	T/T	Affx-109939200	2A	272037603
AX-111654047	A/G	A/G	A/A	Affx-109021201	2A	684202228
AX-111654221	C/C	C/G	C/C	Affx-111801700	2A	757318954
AX-111656956	T/T	T/C	T/T	Affx-111509394	2A	253414355
AX-111657193	T/T	T/C	T/T	Affx-110722063	2A	343106937
AX-111667636	G/G	A/G	G/G	Affx-111308942	2A	319625303
AX-111674816	C/C	T/C	C/C	Affx-88435558	2A	299771231
AX-111684371	G/G	A/G	G/G	Affx-111256339	2A	215596770
AX-111689411	C/C	C/C	C/C	Affx-111852937	2A	777902836
AX-111690116	A/A	A/C	A/A	Affx-110919652	2A	270993211
AX-111711805	T/G	T/T	G/G	Affx-111035514	2A	711644532
AX-111715216	A/A	A/G	G/G	Affx-109815601	2A	570015234
AX-111715319	G/G	C/G	C/C	Affx-109893823	2A	508033045
AX-111722425	C/C	T/C	C/C	Affx-109437838	2A	131715390
AX-111734552	C/C	C/G	G/G	Affx-110306507	2A	509082466
AX-111736818	G/G	A/G	G/G	Affx-111555933	2A	203727350
AX-111747762	C/C	T/C	C/C	Affx-109159029	2A	57822616
AX-111761576	A/A	A/G	A/G	Affx-109491413	2A	505416217
AX-111765585	G/G	A/G	G/G	Affx-109064427	2A	441928727
AX-111775436	A/A	A/G	G/G	Affx-111981208	2A	487455485
AX-111777192	A/A	A/G	G/G	Affx-110249301	2A	489471196
AX-111783557	A/G	A/G	A/G	Affx-111406436	2A	276412594
AX-111786164	A/A	A/G	A/A	Affx-110148117	2A	223205751
AX-112288292	G/G	G/G	G/G	Affx-88651577	2A	92311801
AX-179476247	T/C	C/C	T/T	Affx-109202884	2A	694470714

AX-179559730	A/G	A/G	A/A	Affx-88392281	2A	770011695
AX-182028108	C/C	T/C	T/C	Affx-111279076	2A	777933307
AX-182064705	T/T	T/G	T/G	Affx-472311815	2A	502626466
AX-182075709	G/G	C/C	C/G	Affx-92901465	2A	36930249
AX-182077476	T/T	T/C	T/T	Affx-109970424	2A	728728732
AX-182097727	G/G	G/G	G/G	Affx-108946040	2A	712184394
AX-86166753	A/G	A/A	A/A	Affx-92662770	2A	508032607
AX-86179679	C/C	T/T	T/T	Affx-92547179	2A	612845080
AX-86179766	G/G	C/G	G/G	Affx-110831295	2A	611946860
AX-86184745	C/C	T/C	C/C	Affx-110176295	2A	564502809
AX-89362189	A/A	G/G	A/A	Affx-109353538	2A	712553377
AX-89509613	A/G	G/G	G/G	Affx-88542495	2A	681195112
AX-89560380	C/C	C/C	C/C	Affx-88593427	2A	52241277
AX-89606293	T/T	T/C	C/C	Affx-88639465	2A	37078970
AX-89641927	G/G	A/G	A/A	Affx-110129039	2A	55176566
AX-94400293	G/G	T/T	T/T	Affx-92222714	2A	200901187
AX-94404959	T/C	T/C	T/T	Affx-92737310	2A	715301272
AX-94410389	C/C	T/C	C/C	Affx-92543149	2A	80871520
AX-94423221	T/T	T/T	T/T	Affx-92637657	2A	88983810
AX-94432917	G/G	G/G	G/G	Affx-92883419	2A	507387284
AX-94440806	A/A	A/A	A/A	Affx-92917946	2A	147536397
AX-94478882	T/C	T/T	T/C	Affx-92461565	2A	102677096
AX-94483464	G/G	G/G	G/G	Affx-92861403	2A	755927666
AX-94495090	A/A	G/G	A/A	Affx-92247472	2A	747147132
AX-94497666	A/A	G/G	A/A	Affx-88573602	2A	748441942
AX-94521851	C/C	C/G	C/C	Affx-92881420	2A	58000340
AX-94547450	A/A	A/G	A/A	Affx-88649759	2A	694333888
AX-94553665	C/C	C/C	C/C	Affx-92160548	2A	579615957
AX-94677616	C/C	C/C	T/C	Affx-92700322	2A	728752590
AX-94751398	A/A	A/G	A/A	Affx-92829675	2A	573368522
AX-94769514	A/A	G/G	A/A	Affx-92703757	2A	256192732
AX-94798203	C/C	C/G	C/C	Affx-92612197	2A	759830783
AX-94809384	C/C	C/G	C/C	Affx-92557044	2A	628095790
AX-94840205	A/A	A/A	G/G	Affx-92803882	2A	657110378
AX-94891284	T/T	T/T	T/T	Affx-92948519	2A	770012720
AX-94897794	T/T	T/T	T/T	Affx-88406798	2A	146962617
AX-94928008	G/G	G/G	G/G	Affx-92199383	2A	202872713
AX-95085901	G/G	A/G	G/G	Affx-92708450	2A	5690181
AX-95115448	A/A	A/A	A/A	Affx-92194119	2A	10730932
AX-95209305	A/G	A/A	A/G	Affx-92535696	2A	92314368
AX-95209629	A/A	A/T	T/T	Affx-92957613	2A	674531982
AX-95211185	G/G	A/G	G/G	Affx-92790686	2A	742718678
AX-95225438	A/A	A/A	A/G	Affx-92923866	2A	728752366
AX-95247860	C/G	G/G	G/G	Affx-92387939	2A	710363412
AX-95258273	G/G	G/G	G/G	Affx-92660054	2A	778020672
AX-95630089	A/A	A/A	A/A	Affx-88360443	2A	47826702
AX-95631682	T/C	T/T	T/T	Affx-88568737	2A	48874233
AX-95659338	T/T	T/C	T/T	Affx-88493708	2A	747611742
AX-95660056	T/T	T/G	G/G	Affx-110177310	2A	603524503
AX-95660982	T/T	T/T	T/T	Affx-112313513	2A	696853810
AX-95681919	C/C	T/C	C/C	Affx-92586020	2A	34665317
AX-95684604	T/G	T/G	T/T	Affx-88726489	2A	692850266
AX-108728636	A/A	G/G	G/G	Affx-111735717	2B	30460624
AX-108731425	T/T	T/G	G/G	Affx-110297596	2B	148039783

AX-108733350	C/C	C/G	G/G	Affx-110974249	2B	445897529
AX-108736754	A/C	C/C	C/C	Affx-109263097	2B	562260123
AX-108739525	T/C	T/C	T/C	Affx-109633694	2B	65842048
AX-108743280	T/T	C/C	C/C	Affx-111464406	2B	151651287
AX-108743330	C/C	C/C	C/C	Affx-108968993	2B	9200344
AX-108748081	C/C	T/T	T/T	Affx-111626326	2B	310929339
AX-108757058	T/T	T/G	T/T	Affx-110404249	2B	640086397
AX-108758077	T/T	T/G	T/T	Affx-109975780	2B	354882209
AX-108758236	A/A	A/G	A/A	Affx-109118307	2B	315909140
AX-108773165	T/T	T/G	T/T	Affx-109618950	2B	646890531
AX-108775172	C/C	A/C	A/A	Affx-110900036	2B	200380539
AX-108779261	G/G	C/G	C/C	Affx-109066046	2B	546624638
AX-108782930	C/C	T/C	T/T	Affx-109698397	2B	31575527
AX-108784400	C/C	C/G	C/C	Affx-109432019	2B	95544433
AX-108785918	G/G	A/G	A/A	Affx-111805794	2B	619634652
AX-108790673	G/G	A/A	A/A	Affx-110465406	2B	482049712
AX-108798195	A/A	A/A	G/G	Affx-111601113	2B	448398325
AX-108799498	G/G	A/G	G/G	Affx-110356551	2B	117343285
AX-108802714	G/G	A/G	G/G	Affx-109565410	2B	656693961
AX-108822057	A/A	A/G	A/A	Affx-110667057	2B	95013420
AX-108827959	T/T	T/C	T/T	Affx-88473167	2B	131308826
AX-108828788	G/G	G/G	G/G	Affx-111583303	2B	101301846
AX-108831989	G/G	C/G	G/G	Affx-111111513	2B	759764225
AX-108833859	C/C	T/C	T/T	Affx-110112551	2B	563059164
AX-108834963	C/C	T/C	C/C	Affx-110390289	2B	326867309
AX-108842112	G/G	A/G	G/G	Affx-111670889	2B	51882060
AX-108843759	T/T	T/T	C/C	Affx-108937497	2B	564920289
AX-108844747	G/G	T/G	T/G	Affx-111054873	2B	166417747
AX-108861823	G/G	T/G	G/G	Affx-109854711	2B	245394163
AX-108864330	C/C	T/T	C/C	Affx-110546022	2B	596102789
AX-108884726	T/T	T/G	G/G	Affx-109182354	2B	406114925
AX-108885817	G/G	T/G	G/G	Affx-110870573	2B	161113018
AX-108889031	G/G	A/G	A/A	Affx-111753848	2B	247561903
AX-108895268	A/A	A/C	C/C	Affx-111333589	2B	173185859
AX-108897178	A/A	A/C	A/A	Affx-110909962	2B	569485690
AX-108898684	C/C	T/C	C/C	Affx-111588082	2B	595783496
AX-108899106	C/C	T/T	T/T	Affx-111369890	2B	441180994
AX-108899460	T/T	T/C	C/C	Affx-111572678	2B	201571271
AX-108906314	G/G	A/G	A/A	Affx-109672166	2B	550396678
AX-108910513	T/T	T/T	T/T	Affx-111611729	2B	614900284
AX-108913586	C/C	T/C	C/C	Affx-110181343	2B	439506472
AX-108924817	A/A	A/C	A/A	Affx-110893331	2B	19352401
AX-108925419	G/G	A/G	G/G	Affx-110202352	2B	733838369
AX-108926581	G/G	A/G	A/A	Affx-111353092	2B	609757868
AX-108930003	G/G	C/G	G/G	Affx-110713013	2B	708997932
AX-108947093	A/A	A/G	A/A	Affx-109112636	2B	753837266
AX-108947860	C/C	T/C	T/T	Affx-110184421	2B	363290246
AX-108948069	G/G	A/G	A/A	Affx-111776553	2B	718403638
AX-108950662	A/A	A/C	A/A	Affx-109623779	2B	243824253
AX-108950967	C/C	T/C	C/C	Affx-111765686	2B	529732388
AX-108959459	G/G	G/G	G/G	Affx-110911738	2B	44120129
AX-108962019	A/A	C/C	A/A	Affx-109877471	2B	134094087
AX-108963229	A/C	A/C	A/C	Affx-111249707	2B	130931913
AX-108966669	C/C	T/T	C/C	Affx-109492428	2B	216705806

AX-108974438	C/C	C/G	C/C	Affx-109300377	2B	749894254
AX-108980566	T/T	T/G	T/T	Affx-108910779	2B	714127900
AX-108982891	A/A	A/G	G/G	Affx-88653319	2B	560264243
AX-108991149	C/C	T/C	C/C	Affx-111231506	2B	548391485
AX-108992905	C/C	C/G	C/C	Affx-110472619	2B	640489307
AX-109015706	T/C	T/T	T/C	Affx-109082870	2B	664223432
AX-109018898	C/C	T/C	C/C	Affx-111177259	2B	46116725
AX-109022733	T/T	G/G	T/T	Affx-111309140	2B	109932031
AX-109048108	T/C	T/C	T/T	Affx-111387937	2B	424916275
AX-109051357	G/G	C/G	C/G	Affx-111347559	2B	100258157
AX-109058368	G/G	A/G	A/A	Affx-108923208	2B	246424638
AX-109110439	C/C	T/C	T/T	Affx-110037379	2B	564469966
AX-109271530	G/G	C/G	G/G	Affx-88597658	2B	760929469
AX-109272515	T/T	T/C	T/T	Affx-109797540	2B	100497156
AX-109279654	T/T	T/C	T/T	Affx-111787995	2B	240227545
AX-109279699	C/C	T/C	T/T	Affx-109346361	2B	512174586
AX-109280044	C/C	T/C	C/C	Affx-110411953	2B	145089844
AX-109287003	G/G	A/G	G/G	Affx-109599332	2B	652289944
AX-109290889	T/T	T/C	C/C	Affx-110402588	2B	427947473
AX-109291428	C/C	A/C	C/C	Affx-88607872	2B	101778320
AX-109295999	C/C	C/G	C/C	Affx-111713950	2B	589878582
AX-109297362	T/T	T/C	T/T	Affx-110391578	2B	154235208
AX-109300203	G/G	G/G	G/G	Affx-111452204	2B	725108994
AX-109301932	T/T	T/C	T/T	Affx-110720239	2B	159319395
AX-109302825	T/T	T/C	T/T	Affx-110430223	2B	238650299
AX-109302901	G/G	A/G	A/A	Affx-88589559	2B	182515019
AX-109303300	T/T	C/C	T/T	Affx-109330966	2B	122643252
AX-109308718	G/G	A/G	G/G	Affx-109013536	2B	215330637
AX-109313070	G/G	G/G	G/G	Affx-109600731	2B	743921927
AX-109317883	A/A	A/G	A/A	Affx-109821075	2B	239400946
AX-109317898	G/G	A/G	G/G	Affx-111724991	2B	91848480
AX-109318596	G/G	A/G	A/A	Affx-111453324	2B	476474766
AX-109325753	A/A	A/G	G/G	Affx-111951638	2B	459803014
AX-109326836	T/T	T/C	C/C	Affx-109016155	2B	574044010
AX-109327254	C/C	A/C	C/C	Affx-110299352	2B	712790204
AX-109329349	A/A	G/G	A/A	Affx-111603499	2B	663893121
AX-109335572	G/G	T/G	G/G	Affx-110366402	2B	249919102
AX-109336160	A/A	A/G	A/A	Affx-110643284	2B	239642629
AX-109337411	A/A	A/G	A/A	Affx-111228653	2B	10794822
AX-109343971	T/T	T/G	T/T	Affx-111584777	2B	754661631
AX-109344537	C/C	T/C	T/T	Affx-110237630	2B	428592268
AX-109349804	C/C	C/G	G/G	Affx-109575516	2B	777831275
AX-109358097	T/T	T/C	T/T	Affx-109803369	2B	788026493
AX-109359046	G/G	A/G	A/A	Affx-110719433	2B	715306856
AX-109361154	A/A	A/C	C/C	Affx-110696181	2B	90235514
AX-109365572	G/G	G/G	G/G	Affx-109323058	2B	595368442
AX-109371038	C/C	C/G	C/C	Affx-110703148	2B	111371100
AX-109379034	G/G	G/G	A/A	Affx-110866705	2B	31055018
AX-109380038	T/T	T/C	C/C	Affx-111875920	2B	456013459
AX-109380957	G/G	A/G	A/A	Affx-108939824	2B	233732179
AX-109390390	C/C	C/G	C/C	Affx-110057452	2B	242673179
AX-109390638	C/C	T/C	C/C	Affx-109247991	2B	736856811
AX-109392008	A/A	A/C	C/C	Affx-109212536	2B	427036307
AX-109393067	G/G	T/G	T/T	Affx-88371842	2B	560183014

AX-109393313	C/C	C/G	C/C	Affx-111602436	2B	231354841
AX-109403355	G/G	T/T	T/T	Affx-88663262	2B	455474435
AX-109406051	C/C	C/G	G/G	Affx-109816469	2B	457333642
AX-109408488	T/T	T/C	T/T	Affx-109668782	2B	635722071
AX-109411819	C/C	C/G	C/C	Affx-111415563	2B	581478789
AX-109417361	C/C	A/C	A/A	Affx-111692315	2B	140154748
AX-109418433	T/T	T/C	C/C	Affx-109672711	2B	779250528
AX-109426374	T/T	T/C	T/T	Affx-110892980	2B	130392915
AX-109430236	G/G	A/G	G/G	Affx-109052014	2B	653401634
AX-109431885	C/C	T/C	T/T	Affx-110673109	2B	316047898
AX-109432408	G/G	A/G	G/G	Affx-88541723	2B	47670429
AX-109439054	C/C	T/C	C/C	Affx-111769134	2B	634683534
AX-109439337	G/G	A/G	G/G	Affx-110417631	2B	53511339
AX-109444973	G/G	G/G	G/G	Affx-110044523	2B	9724391
AX-109450139	G/G	A/G	G/G	Affx-110730600	2B	51426839
AX-109455434	T/T	T/C	C/C	Affx-110892471	2B	6044105
AX-109467353	G/G	T/G	T/T	Affx-109422854	2B	171986689
AX-109467835	G/G	A/G	G/G	Affx-88421381	2B	179398337
AX-109470204	T/T	T/C	T/T	Affx-111253561	2B	13750920
AX-109478091	G/G	A/G	G/G	Affx-110307888	2B	20011029
AX-109482497	T/T	T/C	C/C	Affx-109022023	2B	622835608
AX-109491877	G/G	A/G	G/G	Affx-111224890	2B	320757412
AX-109496660	T/T	T/C	C/C	Affx-110936535	2B	415028275
AX-109505207	T/C	T/C	T/C	Affx-110328772	2B	666474502
AX-109506225	A/A	A/A	C/C	Affx-88544147	2B	419628941
AX-109507548	G/G	A/G	G/G	Affx-110698604	2B	358064392
AX-109513912	T/T	T/C	C/C	Affx-109576484	2B	454006048
AX-109516447	T/T	T/T	T/T	Affx-108887013	2B	612873108
AX-109524215	T/T	T/C	T/T	Affx-109573469	2B	98733810
AX-109526191	C/C	A/C	A/A	Affx-110929442	2B	195531681
AX-109529774	C/C	C/C	T/T	Affx-110461892	2B	575838521
AX-109535680	A/A	A/A	G/G	Affx-110194404	2B	422235785
AX-109536693	A/A	A/G	A/A	Affx-88452025	2B	209438343
AX-109544284	G/G	C/G	C/C	Affx-110561134	2B	560459846
AX-109547533	C/C	C/C	C/C	Affx-109507426	2B	376495312
AX-109578675	A/A	A/G	A/A	Affx-110284420	2B	740730962
AX-109582793	A/A	A/G	G/G	Affx-109812686	2B	555504458
AX-109582956	G/G	A/G	A/A	Affx-111007151	2B	425502702
AX-109584685	C/C	G/G	C/C	Affx-111659445	2B	190220157
AX-109596529	G/G	A/G	A/A	Affx-110799178	2B	620077866
AX-109600191	C/C	C/G	C/C	Affx-109914838	2B	67554684
AX-109617405	C/C	A/C	C/C	Affx-110153977	2B	141695923
AX-109618013	G/G	A/G	G/G	Affx-109823544	2B	2386090
AX-109639586	A/A	A/T	A/A	Affx-110031711	2B	128657508
AX-109645815	C/C	T/C	C/C	Affx-109387904	2B	539697028
AX-109651423	G/G	A/A	G/G	Affx-110068592	2B	38730876
AX-109815879	G/G	G/G	G/G	Affx-111506808	2B	760991576
AX-109822778	G/G	A/G	G/G	Affx-110770093	2B	619241066
AX-109823635	A/A	A/C	A/A	Affx-110626942	2B	238026549
AX-109826569	A/A	A/G	A/A	Affx-110661782	2B	242613952
AX-109829147	C/C	T/C	C/C	Affx-110531901	2B	653890805
AX-109829661	C/C	T/C	T/T	Affx-109518694	2B	152182300
AX-109837306	A/A	A/G	G/G	Affx-109366556	2B	177658640
AX-109844754	A/A	A/G	A/A	Affx-110925390	2B	650169193

AX-109851103	C/C	A/C	C/C	Affx-111139883	2B	676020901
AX-109852366	A/A	G/G	A/A	Affx-111542215	2B	19135357
AX-109853315	C/C	T/C	T/T	Affx-110591377	2B	561262081
AX-109856353	C/C	C/G	C/C	Affx-111058093	2B	615364194
AX-109859865	C/C	C/C	C/C	Affx-109786519	2B	193707656
AX-109862245	G/G	A/G	A/A	Affx-110554097	2B	544763013
AX-109865811	A/A	A/G	A/A	Affx-109473074	2B	311954125
AX-109883545	T/T	A/A	T/T	Affx-111566375	2B	190668686
AX-109890461	G/G	A/G	G/G	Affx-111999312	2B	48216300
AX-109893424	T/T	T/C	T/T	Affx-111549587	2B	598672838
AX-109893758	G/G	C/G	G/G	Affx-88675000	2B	663131898
AX-109893764	G/G	G/G	G/G	Affx-109419151	2B	761322218
AX-109900954	C/C	C/G	C/C	Affx-109378795	2B	75535809
AX-109901545	T/T	T/C	T/T	Affx-110586672	2B	232796225
AX-109902602	G/G	T/G	G/G	Affx-110590396	2B	732218082
AX-109904133	T/T	T/G	T/T	Affx-88450887	2B	229992624
AX-109904652	C/C	T/C	T/T	Affx-111788699	2B	434828710
AX-109906455	C/C	C/G	C/C	Affx-111787627	2B	697669319
AX-109917986	G/G	C/G	C/C	Affx-111290094	2B	182927577
AX-109918460	A/A	A/G	G/G	Affx-110891396	2B	10179688
AX-109920471	A/A	A/G	G/G	Affx-110619092	2B	183840020
AX-109921058	C/C	T/C	T/T	Affx-109596928	2B	421260553
AX-109923867	G/G	G/G	G/G	Affx-111246075	2B	438883860
AX-109924214	C/C	T/C	C/C	Affx-109276903	2B	96218192
AX-109932071	G/G	A/G	G/G	Affx-109873997	2B	67987589
AX-109932674	A/A	A/G	A/A	Affx-109284541	2B	522725584
AX-109933035	C/C	A/C	C/C	Affx-109791284	2B	130011251
AX-109943612	T/T	T/T	T/T	Affx-109147152	2B	140776148
AX-109958263	A/A	A/G	A/A	Affx-109744093	2B	105917391
AX-109959398	C/C	T/C	C/C	Affx-110962193	2B	592529748
AX-109965430	A/A	A/C	A/A	Affx-88783330	2B	99497283
AX-109966089	G/G	A/G	G/G	Affx-108890197	2B	224469436
AX-109967115	A/A	A/C	C/C	Affx-109589224	2B	79313063
AX-109971206	C/C	T/C	T/T	Affx-111164042	2B	278733812
AX-109973557	G/G	A/G	A/A	Affx-109247422	2B	474594315
AX-109973772	A/A	A/G	A/A	Affx-111047311	2B	373493468
AX-109974331	T/T	T/C	T/T	Affx-111854961	2B	74205027
AX-109980364	A/A	A/G	A/A	Affx-111008886	2B	755997957
AX-109983420	G/G	A/G	A/A	Affx-111413322	2B	459479288
AX-109989078	T/T	T/T	T/T	Affx-110196097	2B	55065222
AX-109996524	A/A	A/T	A/A	Affx-111716077	2B	673713506
AX-109996754	G/G	A/G	A/A	Affx-109204271	2B	481202213
AX-110000352	T/C	C/C	C/C	Affx-109715940	2B	18312328
AX-110001867	G/G	G/G	G/G	Affx-109766627	2B	632180853
AX-110003371	C/C	C/G	C/C	Affx-109184441	2B	448874637
AX-110012011	C/C	T/C	C/C	Affx-110194808	2B	533556140
AX-110012385	C/G	G/G	C/G	Affx-88703087	2B	244917192
AX-110017170	T/T	T/C	C/C	Affx-109678353	2B	464442409
AX-110028121	T/T	T/T	T/T	Affx-111717499	2B	440353159
AX-110030593	G/G	T/T	T/T	Affx-109908249	2B	395912472
AX-110064241	C/C	T/C	T/C	Affx-111753487	2B	539807448
AX-110064360	C/C	T/C	C/C	Affx-109093430	2B	569129018
AX-110066081	A/A	A/G	G/G	Affx-111806057	2B	423951704
AX-110066797	C/C	T/C	T/T	Affx-110895614	2B	74400676

AX-110075779	G/G	A/G	A/A	Affx-110551429	2B	617931451
AX-110078507	C/C	T/C	T/T	Affx-108987681	2B	273734672
AX-110078747	C/C	C/C	C/C	Affx-108870895	2B	707176237
AX-110093564	C/C	T/C	C/C	Affx-110691363	2B	602996264
AX-110115984	T/T	T/C	T/T	Affx-109151408	2B	388504010
AX-110119738	G/G	G/G	A/A	Affx-109340310	2B	572077967
AX-110126250	A/A	A/C	A/A	Affx-110676462	2B	41440270
AX-110157933	G/G	G/G	A/A	Affx-110109728	2B	138764698
AX-110199373	C/C	C/C	C/C	Affx-109854050	2B	568009990
AX-110363148	A/A	A/A	G/G	Affx-110584645	2B	142884723
AX-110366234	C/C	T/C	T/T	Affx-109782056	2B	607065001
AX-110366650	C/C	T/C	T/T	Affx-109957177	2B	167895042
AX-110370342	G/G	C/G	G/G	Affx-110869273	2B	208409966
AX-110375928	T/T	T/G	G/G	Affx-112001130	2B	572313511
AX-110376228	C/C	C/G	G/G	Affx-111687523	2B	180824564
AX-110384300	C/C	T/C	T/T	Affx-88641631	2B	26100288
AX-110389802	T/T	T/C	T/T	Affx-88423312	2B	132341294
AX-110391760	A/G	A/A	G/G	Affx-88786392	2B	165273090
AX-110393982	C/C	C/C	C/C	Affx-111569187	2B	646210582
AX-110409260	C/C	T/C	C/C	Affx-110470145	2B	5625360
AX-110409899	G/G	A/G	G/G	Affx-111035126	2B	110573187
AX-110409902	G/G	T/G	G/G	Affx-109220285	2B	73796750
AX-110411572	C/C	T/C	T/T	Affx-109045306	2B	174651978
AX-110412968	G/G	A/A	G/G	Affx-111877224	2B	384396003
AX-110413996	A/A	A/G	A/G	Affx-109081257	2B	600247014
AX-110415170	C/C	C/G	C/C	Affx-109960686	2B	57683608
AX-110420126	T/C	C/C	T/C	Affx-110612409	2B	662317267
AX-110420288	T/T	T/C	T/T	Affx-111549676	2B	737494926
AX-110423885	G/G	A/G	G/G	Affx-111390249	2B	766172990
AX-110425178	T/T	T/G	T/T	Affx-110664245	2B	568625945
AX-110426534	A/A	A/G	G/G	Affx-111088221	2B	549307092
AX-110426897	A/A	A/G	A/A	Affx-109458055	2B	33541973
AX-110429143	C/C	G/G	G/G	Affx-110750729	2B	415360166
AX-110430167	C/C	C/G	C/C	Affx-109508488	2B	642705852
AX-110430269	G/G	A/G	A/G	Affx-109767910	2B	487866767
AX-110431393	A/A	A/G	G/G	Affx-109353706	2B	462901651
AX-110431742	A/A	A/C	A/A	Affx-111683253	2B	598974226
AX-110434717	G/G	A/G	G/G	Affx-109480949	2B	652851113
AX-110439040	C/C	T/C	T/T	Affx-109520969	2B	611282001
AX-110441819	C/C	C/C	C/C	Affx-110052674	2B	780017142
AX-110443918	T/T	T/C	C/C	Affx-111579486	2B	409883668
AX-110445877	A/A	A/G	A/A	Affx-110145473	2B	676259042
AX-110447694	G/G	C/G	C/C	Affx-108975135	2B	461092490
AX-110450766	C/C	T/C	C/C	Affx-110470215	2B	648418905
AX-110456354	T/T	T/C	C/C	Affx-109474208	2B	575336662
AX-110457023	T/T	T/C	T/T	Affx-111848775	2B	158136698
AX-110457367	G/G	A/A	A/A	Affx-108873812	2B	475855702
AX-110465578	T/G	T/G	G/G	Affx-109554771	2B	236298408
AX-110467472	C/C	T/C	C/C	Affx-109455851	2B	45521096
AX-110473362	C/C	C/G	G/G	Affx-108961612	2B	206109286
AX-110476598	C/C	T/C	T/T	Affx-109443389	2B	174319593
AX-110480883	G/G	T/G	G/G	Affx-111921866	2B	86894359
AX-110481484	C/C	C/C	T/T	Affx-111389973	2B	464029181
AX-110485102	A/A	A/G	G/G	Affx-111332685	2B	704576252

AX-110486035	C/C	C/G	G/G	Affx-109906132	2B	573811849
AX-110488291	C/C	C/C	G/G	Affx-111062614	2B	788994770
AX-110488760	G/G	A/G	G/G	Affx-111311135	2B	651360266
AX-110494473	T/T	T/C	C/C	Affx-109551311	2B	181410560
AX-110494713	C/C	T/C	T/T	Affx-111845279	2B	183323298
AX-110497059	G/G	C/G	G/G	Affx-111022871	2B	649480210
AX-110503087	A/A	A/G	A/A	Affx-109106283	2B	119588815
AX-110505529	A/A	A/C	A/A	Affx-110629390	2B	680523508
AX-110506507	C/C	A/C	A/A	Affx-109692890	2B	169919175
AX-110511445	A/A	A/C	A/A	Affx-111674582	2B	522815621
AX-110518304	A/T	A/T	A/T	Affx-109144777	2B	42156077
AX-110518923	C/C	A/C	C/C	Affx-108883161	2B	633694762
AX-110519757	C/C	C/C	T/T	Affx-109694052	2B	433175613
AX-110520808	C/C	T/C	C/C	Affx-109547116	2B	269131592
AX-110530071	C/C	T/C	C/C	Affx-109969504	2B	601850608
AX-110530754	C/C	T/C	C/C	Affx-110467602	2B	581791893
AX-110541891	T/T	T/C	T/T	Affx-110872576	2B	236881264
AX-110542001	C/C	T/C	C/C	Affx-88531969	2B	209087017
AX-110542725	C/C	C/G	C/C	Affx-110681771	2B	103712610
AX-110549181	A/A	A/G	A/A	Affx-110693942	2B	657079783
AX-110550065	T/T	T/C	T/T	Affx-110681592	2B	38757362
AX-110550882	T/T	T/G	G/G	Affx-109742141	2B	235232003
AX-110558084	T/C	T/C	C/C	Affx-110949233	2B	618665539
AX-110598098	G/G	A/G	A/A	Affx-111266591	2B	26178491
AX-110602750	C/C	T/C	C/C	Affx-109327318	2B	598620287
AX-110607918	G/G	A/A	A/A	Affx-111444264	2B	613917550
AX-110611894	A/A	A/T	A/A	Affx-111090413	2B	546230426
AX-110622949	C/C	A/A	C/C	Affx-111400458	2B	99757256
AX-110634292	C/C	C/C	C/C	Affx-88694340	2B	594172193
AX-110644789	T/C	C/C	C/C	Affx-109694445	2B	159099574
AX-110645975	T/T	G/G	T/T	Affx-111141389	2B	679910221
AX-110649607	G/G	G/G	G/G	Affx-110990504	2B	92508072
AX-110653326	T/T	T/T	C/C	Affx-108940745	2B	164896979
AX-110659895	T/T	T/G	T/T	Affx-111257172	2B	680754280
AX-110661329	C/C	T/C	T/T	Affx-109681787	2B	150716138
AX-110663094	C/C	T/C	C/C	Affx-111023605	2B	793131716
AX-110674570	G/G	A/G	A/A	Affx-111313056	2B	460377207
AX-110689474	G/G	A/G	A/A	Affx-110442183	2B	474628864
AX-110906506	G/G	A/G	A/A	Affx-109177363	2B	456705951
AX-110911052	G/G	A/G	G/G	Affx-109864725	2B	355601056
AX-110913830	G/G	C/G	G/G	Affx-88429969	2B	115380003
AX-110916919	C/C	T/C	C/C	Affx-109322579	2B	397856216
AX-110921250	C/C	C/C	A/A	Affx-109762153	2B	603800305
AX-110921670	G/G	A/G	G/G	Affx-108887832	2B	243351094
AX-110924488	T/T	T/C	T/T	Affx-88449304	2B	597807793
AX-110925252	T/T	T/C	T/C	Affx-111212149	2B	167085403
AX-110927135	C/C	T/C	C/C	Affx-109544273	2B	690894650
AX-110930369	C/C	C/G	C/C	Affx-111860798	2B	533507731
AX-110932305	T/T	T/C	T/T	Affx-111738144	2B	40444714
AX-110933465	C/C	C/G	C/C	Affx-110038782	2B	632588442
AX-110934424	T/T	T/C	T/T	Affx-108963846	2B	745698699
AX-110935265	C/C	C/C	C/C	Affx-109534894	2B	643681584
AX-110939718	G/G	A/G	G/G	Affx-111860121	2B	67084062
AX-110941007	C/C	A/C	A/A	Affx-110767710	2B	797272162

AX-110942863	G/G	G/G	T/T	Affx-110631397	2B	717778130
AX-110945324	C/G	G/G	C/G	Affx-110904576	2B	678077347
AX-110950084	C/C	T/C	C/C	Affx-111021720	2B	677851371
AX-110953404	G/G	A/G	G/G	Affx-109480424	2B	39521120
AX-110957832	C/C	C/C	T/T	Affx-110703424	2B	608538522
AX-110959136	C/C	T/C	T/T	Affx-110672905	2B	431032642
AX-110965570	G/G	A/G	A/A	Affx-111011183	2B	198308454
AX-110969660	A/A	A/C	C/C	Affx-110181251	2B	576093016
AX-110978270	G/G	T/G	T/T	Affx-111532060	2B	173571322
AX-110983186	G/G	A/G	A/A	Affx-111760383	2B	486061007
AX-110987046	A/A	A/G	A/A	Affx-109115713	2B	104941104
AX-110992798	G/G	A/G	A/A	Affx-88421257	2B	691415158
AX-110994146	G/G	A/G	G/G	Affx-111271412	2B	48879041
AX-110996334	A/A	A/C	C/C	Affx-110812225	2B	449164364
AX-110997403	C/C	A/C	C/C	Affx-109940204	2B	773410300
AX-110998243	C/C	C/G	C/C	Affx-109385850	2B	103397808
AX-111002022	G/G	A/G	A/A	Affx-110137973	2B	149811219
AX-111003717	T/T	T/G	T/T	Affx-110380900	2B	712344206
AX-111007493	G/G	A/G	A/A	Affx-111631941	2B	436879717
AX-111008818	A/A	A/T	T/T	Affx-111756867	2B	169514864
AX-111009907	G/G	G/G	G/G	Affx-88661649	2B	732889613
AX-111014597	G/G	T/G	T/T	Affx-110407511	2B	616395582
AX-111015582	G/G	A/A	A/A	Affx-111657152	2B	783455781
AX-111017539	A/A	G/G	G/G	Affx-109327811	2B	171424483
AX-111017863	C/C	T/C	C/C	Affx-110901047	2B	615886592
AX-111026116	G/G	A/A	G/G	Affx-109508320	2B	579565661
AX-111026170	A/A	A/T	A/A	Affx-111571857	2B	107386493
AX-111026216	T/T	T/G	T/T	Affx-108995764	2B	538016047
AX-111029824	A/A	G/G	G/G	Affx-110803431	2B	458092952
AX-111030216	C/C	T/C	C/C	Affx-111195998	2B	122259442
AX-111030988	G/G	A/G	A/A	Affx-110672303	2B	621107807
AX-111038278	A/A	A/G	A/A	Affx-108890915	2B	583576595
AX-111038927	G/G	A/G	G/G	Affx-111373495	2B	438977511
AX-111044341	G/G	A/G	G/G	Affx-111191855	2B	178321939
AX-111045653	G/G	T/G	T/T	Affx-110805538	2B	436520872
AX-111054388	G/G	A/G	G/G	Affx-109559457	2B	191881369
AX-111055545	T/T	T/C	T/T	Affx-110709321	2B	662841162
AX-111058825	C/C	T/C	C/C	Affx-110712950	2B	648702418
AX-111063291	G/G	A/G	A/A	Affx-111353451	2B	549157401
AX-111068233	G/G	A/G	G/G	Affx-111576389	2B	303103533
AX-111073595	G/G	A/G	A/A	Affx-109682224	2B	158437492
AX-111081804	G/G	C/G	C/C	Affx-111859428	2B	785065309
AX-111086670	T/T	T/C	C/C	Affx-109659772	2B	447826269
AX-111104538	G/G	A/G	A/A	Affx-109621521	2B	451952291
AX-111105226	A/A	A/C	A/A	Affx-109623457	2B	5074308
AX-111106689	G/G	G/G	A/G	Affx-109095838	2B	153228147
AX-111109669	G/G	C/C	G/G	Affx-110095820	2B	117990136
AX-111111731	A/A	A/G	A/G	Affx-111943361	2B	177545070
AX-111114948	T/T	T/C	T/T	Affx-109158240	2B	655225005
AX-111115491	T/T	T/C	T/T	Affx-111108631	2B	592062105
AX-111123423	T/C	T/C	C/C	Affx-109058742	2B	202181594
AX-111125171	G/G	A/G	A/A	Affx-110535455	2B	787967304
AX-111140614	C/C	T/C	T/T	Affx-110279597	2B	423235978
AX-111154071	G/G	A/G	G/G	Affx-109492292	2B	93105895

AX-111156102	C/C	T/C	T/T	Affx-109276699	2B	427410746
AX-111166288	C/C	T/C	C/C	Affx-88700360	2B	675337447
AX-111166929	A/A	A/G	A/A	Affx-109194658	2B	612680821
AX-111167392	C/C	T/C	T/C	Affx-111490385	2B	236168397
AX-111167874	G/G	G/G	T/T	Affx-109180763	2B	547007986
AX-111169101	T/T	C/C	C/C	Affx-88346845	2B	620619423
AX-111183247	G/G	A/G	A/A	Affx-111053323	2B	563313895
AX-111190808	A/A	A/G	G/G	Affx-111720803	2B	414071323
AX-111193111	G/G	G/G	G/G	Affx-109075027	2B	59654758
AX-111208332	T/T	C/C	T/T	Affx-92356351	2B	91261147
AX-111215348	A/A	A/G	A/A	Affx-111153523	2B	240700422
AX-111228210	T/T	T/G	G/G	Affx-109234701	2B	433794909
AX-111230062	G/G	G/G	A/A	Affx-111241413	2B	17141279
AX-111233617	T/T	T/T	T/T	Affx-111932111	2B	636413723
AX-111251967	C/C	T/C	T/T	Affx-109611950	2B	149045888
AX-111288278	T/T	T/G	T/T	Affx-111491623	2B	590583512
AX-111458861	G/G	A/G	G/G	Affx-111987350	2B	116848708
AX-111467730	T/T	T/T	T/T	Affx-109115316	2B	774113399
AX-111472031	A/A	A/A	A/A	Affx-111610052	2B	589089676
AX-111472511	G/G	A/G	G/G	Affx-110798615	2B	732339052
AX-111478251	T/T	T/T	T/T	Affx-108858676	2B	332563
AX-111478321	G/G	G/G	G/G	Affx-109033700	2B	231864141
AX-111480841	C/C	C/C	C/C	Affx-111809611	2B	137864504
AX-111483767	C/C	T/C	T/T	Affx-88768559	2B	422965790
AX-111489510	A/A	A/G	A/A	Affx-110481152	2B	135515635
AX-111489579	G/G	A/G	A/A	Affx-110711502	2B	463455498
AX-111491698	A/A	A/G	A/A	Affx-111561893	2B	786409318
AX-111496247	A/A	A/G	A/A	Affx-110522182	2B	93932311
AX-111500033	G/G	G/G	C/C	Affx-110874354	2B	22617347
AX-111503087	G/G	A/A	G/G	Affx-109303286	2B	639060797
AX-111503986	C/C	T/C	T/T	Affx-110001087	2B	426597162
AX-111507200	C/C	T/C	T/T	Affx-110765535	2B	613255706
AX-111513823	G/G	A/G	G/G	Affx-110672912	2B	69017622
AX-111524104	G/G	C/G	G/G	Affx-109731430	2B	118474965
AX-111526877	G/G	G/G	G/G	Affx-111390079	2B	674024885
AX-111529691	A/A	G/G	A/A	Affx-110933501	2B	24133804
AX-111531325	A/A	A/G	A/A	Affx-109314706	2B	11342028
AX-111531916	T/T	T/T	T/T	Affx-108972380	2B	768379332
AX-111534641	A/A	A/G	A/A	Affx-111788461	2B	134238802
AX-111535545	T/T	T/C	T/T	Affx-109667318	2B	98149781
AX-111536250	G/G	C/G	G/G	Affx-110753046	2B	632616996
AX-111540604	G/G	C/G	G/G	Affx-109936106	2B	596890631
AX-111544566	A/A	A/G	A/A	Affx-110523807	2B	555836744
AX-111546188	C/C	T/C	C/C	Affx-111980925	2B	739729603
AX-111549027	T/T	G/G	T/T	Affx-111277846	2B	752404826
AX-111553009	T/C	T/C	T/C	Affx-109511236	2B	636560110
AX-111555027	T/T	T/C	T/T	Affx-111531918	2B	566424468
AX-111557818	A/A	A/G	A/A	Affx-110667052	2B	756597337
AX-111559149	A/A	A/A	G/G	Affx-109253105	2B	3295296
AX-111559203	T/T	T/T	T/T	Affx-111342005	2B	154588905
AX-111561046	C/C	C/C	T/T	Affx-110069201	2B	425876715
AX-111561311	G/G	A/G	A/A	Affx-88805254	2B	611276060
AX-111561925	A/A	A/G	A/A	Affx-108938787	2B	473216513
AX-111563920	G/G	A/G	A/A	Affx-111462548	2B	462944142

AX-111564969	A/A	A/G	A/A	Affx-111811659	2B	721895424
AX-111565799	T/T	T/C	T/T	Affx-88743748	2B	215197322
AX-111568766	C/C	C/C	C/C	Affx-88579579	2B	93138203
AX-111579036	C/C	C/G	G/G	Affx-111239508	2B	453587085
AX-111580003	T/T	T/C	T/T	Affx-110751378	2B	106479620
AX-111586036	A/A	A/G	A/A	Affx-111474389	2B	647450303
AX-111588208	C/C	C/C	C/C	Affx-110880615	2B	191359578
AX-111590094	C/C	T/C	C/C	Affx-110517399	2B	641942882
AX-111590492	G/G	G/G	A/A	Affx-109902732	2B	562211963
AX-111596419	C/C	T/C	C/C	Affx-109969538	2B	536994668
AX-111597567	A/A	A/G	A/A	Affx-111891926	2B	535380713
AX-111599386	T/T	T/G	G/G	Affx-111480102	2B	457596300
AX-111605121	C/C	C/G	C/C	Affx-110638582	2B	20370863
AX-111608510	G/G	A/G	G/G	Affx-109165051	2B	66027761
AX-111608888	C/C	T/C	C/C	Affx-109649405	2B	113165090
AX-111614429	A/A	C/C	C/C	Affx-109166291	2B	17890308
AX-111616168	G/G	T/G	G/G	Affx-109724719	2B	72590170
AX-111617443	A/G	A/G	A/G	Affx-110702264	2B	207024045
AX-111622432	C/C	C/G	C/C	Affx-88530723	2B	50624192
AX-111623225	C/C	T/C	C/C	Affx-109907328	2B	11903807
AX-111631370	G/G	T/G	G/G	Affx-109261824	2B	59245230
AX-111632213	G/G	C/G	C/C	Affx-109319501	2B	481643114
AX-111634141	C/C	T/C	C/C	Affx-109842890	2B	762369730
AX-111636316	G/G	A/G	A/A	Affx-110958722	2B	603198153
AX-111640532	G/G	A/A	G/G	Affx-109519392	2B	682801798
AX-111653012	C/C	C/C	C/C	Affx-110940345	2B	192931796
AX-111657169	A/A	A/G	G/G	Affx-108982747	2B	165503771
AX-111661669	C/C	T/C	C/C	Affx-110405065	2B	666653511
AX-111676781	G/G	A/G	G/G	Affx-111600271	2B	793056666
AX-111682299	C/C	T/T	T/T	Affx-110725132	2B	419659310
AX-111684176	G/G	A/G	A/A	Affx-109363771	2B	164094555
AX-111687869	A/G	A/G	G/G	Affx-109460716	2B	147915244
AX-111688164	C/C	T/C	T/T	Affx-110683360	2B	429116480
AX-111699215	C/C	C/C	C/C	Affx-111078127	2B	88932426
AX-111708657	A/A	A/T	A/A	Affx-109621834	2B	755589356
AX-111709620	A/A	G/G	G/G	Affx-88520406	2B	185811728
AX-111710157	G/G	A/G	G/G	Affx-88720090	2B	214285161
AX-111710268	G/G	A/G	A/A	Affx-109297689	2B	24269887
AX-111715350	A/A	A/A	A/A	Affx-111212513	2B	593655953
AX-111715747	A/A	A/G	A/A	Affx-110017166	2B	610691577
AX-111716247	A/A	G/G	G/G	Affx-110887047	2B	507508283
AX-111735245	C/C	T/C	T/T	Affx-109751152	2B	468662680
AX-111735530	C/C	T/C	T/T	Affx-110086211	2B	430242427
AX-111735910	C/C	T/C	T/T	Affx-110298762	2B	144434018
AX-111738233	T/T	T/T	T/T	Affx-109317785	2B	661781633
AX-111748754	C/C	T/C	T/C	Affx-110494632	2B	488992890
AX-111756131	C/C	T/C	C/C	Affx-88635325	2B	737227764
AX-111757791	T/T	T/C	C/C	Affx-109616323	2B	489262863
AX-111760099	C/C	C/C	C/C	Affx-109137653	2B	141734940
AX-111764426	G/G	A/G	G/G	Affx-109957574	2B	207445710
AX-111768556	T/T	T/C	T/T	Affx-111213342	2B	661556117
AX-111772934	T/T	C/C	C/C	Affx-110467324	2B	27899945
AX-111778463	C/C	T/C	T/T	Affx-109342483	2B	472356066
AX-111795121	G/G	A/G	A/A	Affx-111489997	2B	497513565

AX-111826050	G/G	G/G	C/G	Affx-110382855	2B	362539985
AX-111830247	T/T	T/C	C/C	Affx-109570583	2B	783166133
AX-112286171	C/C	T/C	C/C	Affx-92961796	2B	133704693
AX-112287417	C/C	T/C	C/C	Affx-88802226	2B	601334100
AX-112290186	A/G	A/G	A/A	Affx-112314638	2B	746101729
AX-179558346	T/T	T/T	T/G	Affx-88488002	2B	139342527
AX-179558727	T/C	C/C	T/C	Affx-88456670	2B	154250491
AX-86165272	A/G	G/G	G/G	Affx-92222852	2B	72610551
AX-86174797	C/C	C/C	C/C	Affx-109502077	2B	191752438
AX-89331684	T/T	T/C	T/T	Affx-88363014	2B	49491559
AX-89358007	C/C	C/C	A/C	Affx-88389980	2B	563860364
AX-89359264	G/G	G/G	G/G	Affx-88391275	2B	540707530
AX-89410672	A/A	A/G	A/A	Affx-88443265	2B	373760113
AX-89454891	G/G	G/G	G/G	Affx-110842819	2B	106829802
AX-89494371	G/G	A/G	G/G	Affx-88527240	2B	695518245
AX-89496645	C/C	T/C	C/C	Affx-88529518	2B	692463399
AX-89514984	C/C	T/C	C/C	Affx-88547878	2B	210207567
AX-89520190	C/C	C/G	C/C	Affx-88553103	2B	781579139
AX-89589127	G/G	G/G	G/G	Affx-88622254	2B	696375979
AX-89620072	G/G	G/G	G/G	Affx-88653268	2B	154251253
AX-89681468	C/C	T/T	C/C	Affx-88714230	2B	767687813
AX-89729906	A/A	A/G	G/G	Affx-88762595	2B	514340796
AX-89734740	C/C	A/C	A/A	Affx-88767430	2B	547910322
AX-89774932	C/C	C/C	T/T	Affx-88805762	2B	716815705
AX-94381307	C/C	C/C	C/C	Affx-92659580	2B	39308802
AX-94401169	A/G	A/A	G/G	Affx-92818589	2B	709830737
AX-94431531	C/C	C/C	C/C	Affx-92711215	2B	160282610
AX-94457175	C/C	C/C	C/C	Affx-92941887	2B	595126605
AX-94462172	T/T	T/C	C/C	Affx-92742008	2B	323078373
AX-94469044	G/G	A/A	G/G	Affx-92327580	2B	588709339
AX-94475621	G/G	G/G	G/G	Affx-88605401	2B	594849586
AX-94483531	C/G	C/C	C/C	Affx-92628250	2B	26986979
AX-94484528	A/A	A/A	A/A	Affx-92686143	2B	217908634
AX-94495566	T/C	T/T	T/C	Affx-92103552	2B	762502999
AX-94508024	T/T	T/G	G/G	Affx-92834185	2B	28452664
AX-94509892	C/C	T/C	T/T	Affx-92521913	2B	777301801
AX-94515318	T/T	T/T	C/C	Affx-88422889	2B	148589581
AX-94525439	T/C	T/T	C/C	Affx-92143959	2B	683754755
AX-94586951	T/T	T/T	C/C	Affx-92453875	2B	443159960
AX-94587803	T/T	G/G	T/T	Affx-92714519	2B	677347803
AX-94603887	G/G	A/A	G/G	Affx-92713848	2B	210465878
AX-94606504	T/T	T/G	T/T	Affx-92782878	2B	688431192
AX-94645156	C/G	C/C	C/C	Affx-92809930	2B	719473943
AX-94655468	A/A	A/A	A/G	Affx-92282769	2B	330448556
AX-94672440	G/G	A/G	G/G	Affx-92463394	2B	764074058
AX-94707612	G/G	A/G	G/G	Affx-92739623	2B	765295852
AX-94717967	C/C	A/A	A/C	Affx-92322914	2B	78871044
AX-94740068	A/G	A/G	A/A	Affx-92203409	2B	695891858
AX-94761657	C/C	C/C	T/C	Affx-472305348	2B	149841689
AX-94763687	A/G	A/A	A/G	Affx-92867473	2B	767021430
AX-94766218	T/T	C/C	T/T	Affx-88645032	2B	164107563
AX-94791055	A/A	A/C	A/A	Affx-92563494	2B	749138678
AX-94797912	G/G	G/G	A/A	Affx-88715262	2B	174931450
AX-94812811	C/C	C/C	C/C	Affx-88611238	2B	21950859

AX-94818828	T/T	T/G	T/T	Affx-92598266	2B	538236340
AX-94842524	G/G	G/G	G/G	Affx-92388842	2B	743746456
AX-94845937	T/T	T/C	T/T	Affx-92698619	2B	97414508
AX-94849048	G/G	C/G	G/G	Affx-92649474	2B	33944383
AX-94861184	C/G	C/C	C/G	Affx-92262947	2B	753091286
AX-94873920	G/G	T/G	G/G	Affx-92164556	2B	245888605
AX-94881558	A/A	A/A	A/C	Affx-92219164	2B	442924785
AX-94901457	A/A	A/G	A/G	Affx-92389050	2B	50412251
AX-94925003	C/C	C/C	C/C	Affx-92757714	2B	116472420
AX-94940925	A/A	G/G	G/G	Affx-92287891	2B	404109978
AX-94943179	T/C	C/C	T/C	Affx-92573093	2B	566627471
AX-94945766	G/G	G/G	C/G	Affx-92661612	2B	30497799
AX-94946490	T/T	T/T	T/T	Affx-88487536	2B	706269885
AX-94970241	T/G	T/G	T/G	Affx-92539327	2B	248098460
AX-94970352	C/C	T/C	T/T	Affx-92747453	2B	786105457
AX-94974280	T/C	T/T	C/C	Affx-92312631	2B	789950007
AX-95008467	C/C	C/G	C/C	Affx-92140969	2B	188719350
AX-95099952	T/G	T/G	T/T	Affx-92476592	2B	157693798
AX-95107926	G/G	A/G	G/G	Affx-92460390	2B	798135144
AX-95112282	C/C	C/C	A/C	Affx-92818704	2B	445251869
AX-95141306	C/C	C/C	C/C	Affx-92127895	2B	635153496
AX-95144982	G/G	G/G	G/G	Affx-92283149	2B	65370487
AX-95158415	A/A	A/G	G/G	Affx-92870256	2B	529257684
AX-95166376	G/G	A/G	A/A	Affx-92875769	2B	399353854
AX-95185074	G/G	A/G	G/G	Affx-92935637	2B	247390128
AX-95197196	T/T	C/C	T/T	Affx-92488639	2B	65100055
AX-95219673	C/C	T/C	C/C	Affx-92953104	2B	671741110
AX-95235935	T/T	C/C	T/T	Affx-92775486	2B	556847032
AX-95239034	A/A	A/C	A/A	Affx-92246600	2B	229443704
AX-95629355	G/G	A/A	G/G	Affx-109675800	2B	42953388
AX-95630098	C/C	A/C	C/C	Affx-92185860	2B	697510373
AX-95630445	C/C	C/C	C/C	Affx-92116375	2B	763633070
AX-95630572	G/G	G/G	G/G	Affx-92499748	2B	690218325
AX-95633225	A/G	G/G	A/G	Affx-92280180	2B	31422132
AX-95658804	T/T	T/G	G/G	Affx-88663321	2B	245790742
AX-95658926	C/C	C/C	T/T	Affx-88591210	2B	31622741
AX-95683850	G/G	A/G	G/G	Affx-88469114	2B	58978466
AX-108725521	C/C	C/G	C/C	Affx-111097491	2D	76975783
AX-108732016	C/C	T/C	C/C	Affx-110827493	2D	447375226
AX-108736633	C/G	G/G	C/G	Affx-110126671	2D	21251282
AX-108738641	G/G	T/G	G/G	Affx-110603403	2D	300433221
AX-108744106	A/A	A/G	G/G	Affx-88573861	2D	571074310
AX-108749673	T/T	T/G	T/T	Affx-109273884	2D	287587632
AX-108762451	C/C	A/C	C/C	Affx-110967038	2D	5908105
AX-108764406	G/G	C/G	G/G	Affx-110520215	2D	641122338
AX-108768445	G/G	C/G	C/C	Affx-110652297	2D	11779968
AX-108775703	T/T	T/C	T/T	Affx-111611116	2D	202606507
AX-108778899	G/G	A/G	G/G	Affx-109145870	2D	607495200
AX-108792404	C/C	C/C	A/A	Affx-110457031	2D	51856210
AX-108796917	C/C	T/C	C/C	Affx-110182374	2D	493713570
AX-108814021	G/G	A/G	G/G	Affx-109042003	2D	156140885
AX-108816400	G/G	A/A	G/G	Affx-110789790	2D	315814243
AX-108818140	G/G	T/G	G/G	Affx-109057281	2D	68350399
AX-108820900	T/T	T/T	T/T	Affx-111190128	2D	170473270

AX-108821201	C/C	C/G	C/C	Affx-110031330	2D	19626261
AX-108822265	C/C	T/C	T/T	Affx-111276586	2D	130258240
AX-108826933	C/C	T/C	C/C	Affx-88523966	2D	506202563
AX-108838533	G/G	A/G	G/G	Affx-109042782	2D	20349449
AX-108840527	A/A	A/C	A/A	Affx-111552481	2D	392616983
AX-108854624	G/G	A/G	A/G	Affx-111222986	2D	236087391
AX-108856762	A/A	C/C	A/A	Affx-109861191	2D	62404512
AX-108859528	T/T	T/C	T/T	Affx-111310391	2D	333835510
AX-108864872	A/A	A/A	G/G	Affx-110199264	2D	615716971
AX-108868236	A/A	A/G	A/A	Affx-110463198	2D	365573358
AX-108873907	G/G	A/G	G/G	Affx-111440364	2D	151129463
AX-108893965	A/A	G/G	G/G	Affx-109869458	2D	561439797
AX-108902405	C/C	T/T	T/T	Affx-109741895	2D	543044648
AX-108903243	A/G	A/G	A/G	Affx-111293739	2D	49238270
AX-108911375	T/T	T/C	T/T	Affx-110959796	2D	38546495
AX-108912943	C/C	T/C	T/T	Affx-108917545	2D	256536600
AX-108914573	G/G	C/G	G/G	Affx-109395574	2D	58547509
AX-108929084	C/C	T/C	C/C	Affx-108857801	2D	314419095
AX-108929642	C/C	A/C	C/C	Affx-111125438	2D	125076643
AX-108937321	G/G	A/G	A/A	Affx-109139615	2D	589280336
AX-108945987	T/T	T/C	T/T	Affx-111473819	2D	23045002
AX-108947865	A/A	A/G	A/A	Affx-111099095	2D	344766560
AX-108949551	A/A	A/T	T/T	Affx-88688105	2D	588798706
AX-108950311	A/G	G/G	G/G	Affx-93001820	2D	173007892
AX-108953769	T/T	T/C	T/T	Affx-111855142	2D	337851889
AX-108978599	G/G	A/G	G/G	Affx-110921146	2D	619954348
AX-108983103	A/A	A/C	A/A	Affx-111440940	2D	23047572
AX-108986429	A/A	A/G	A/A	Affx-109659921	2D	17235991
AX-108988107	C/C	T/C	C/C	Affx-108901764	2D	19624470
AX-108991883	A/A	A/G	A/A	Affx-110498813	2D	73856404
AX-109000366	C/C	T/C	C/C	Affx-111777313	2D	108772078
AX-109013500	T/T	T/G	T/T	Affx-108923878	2D	63568198
AX-109014614	G/G	A/G	G/G	Affx-110479146	2D	473586844
AX-109023662	A/A	A/G	A/G	Affx-111438948	2D	521528010
AX-109034933	C/C	T/C	C/C	Affx-110076134	2D	77111579
AX-109054659	A/G	A/G	A/G	Affx-111196266	2D	627848496
AX-109058883	G/G	T/G	G/G	Affx-111468753	2D	24555490
AX-109075737	T/T	T/C	T/T	Affx-111572900	2D	255384852
AX-109080377	G/G	A/G	A/A	Affx-108937778	2D	613624691
AX-109080970	G/G	G/G	G/G	Affx-111715608	2D	262963174
AX-109081621	G/G	T/G	G/G	Affx-110627566	2D	228066069
AX-109093094	G/G	A/G	A/A	Affx-111694297	2D	576583493
AX-109106856	G/G	A/G	G/G	Affx-110236075	2D	254590190
AX-109187701	G/G	A/G	G/G	Affx-88779741	2D	452749318
AX-109194011	A/A	A/G	A/A	Affx-88428663	2D	65111982
AX-109208408	G/G	C/G	G/G	Affx-110726058	2D	212372982
AX-109245428	C/C	C/C	C/C	Affx-111552394	2D	171434354
AX-109246010	G/G	A/G	G/G	Affx-110934526	2D	467689413
AX-109266674	C/C	T/C	C/C	Affx-109133967	2D	72895147
AX-109267554	C/C	A/C	C/C	Affx-111639912	2D	77110599
AX-109270146	G/G	T/G	G/G	Affx-109071935	2D	24564497
AX-109270631	T/T	A/T	T/T	Affx-109429317	2D	67652383
AX-109274813	T/T	T/C	C/C	Affx-109910780	2D	522505429
AX-109276654	C/C	T/C	T/T	Affx-111610663	2D	333096382

AX-109278936	C/C	C/C	C/C	Affx-88341176	2D	70282170
AX-109282323	G/G	A/G	G/G	Affx-109139361	2D	482280198
AX-109283238	T/T	T/C	T/T	Affx-109165489	2D	82189082
AX-109287188	T/T	T/C	T/T	Affx-111539868	2D	619405908
AX-109299975	G/G	A/G	A/A	Affx-88516181	2D	538795825
AX-109302441	G/G	G/G	A/A	Affx-109664402	2D	327758997
AX-109309192	G/G	C/C	C/C	Affx-111301913	2D	584855933
AX-109319204	C/C	T/C	T/T	Affx-88785357	2D	594580754
AX-109328071	A/A	A/G	A/A	Affx-110105620	2D	24448702
AX-109330666	G/G	A/G	G/G	Affx-109506672	2D	431959521
AX-109331314	T/T	T/C	T/T	Affx-109865915	2D	60011290
AX-109339465	C/C	C/G	C/C	Affx-111157522	2D	111001161
AX-109348456	C/C	A/A	C/C	Affx-110518542	2D	149932168
AX-109351097	A/A	A/G	G/G	Affx-110559478	2D	547162284
AX-109358637	C/C	C/C	C/C	Affx-110993320	2D	465115877
AX-109359261	A/A	A/C	A/A	Affx-109361252	2D	141113352
AX-109360320	C/C	T/C	C/C	Affx-88773934	2D	195723966
AX-109363258	C/C	C/G	C/C	Affx-111986851	2D	622821293
AX-109369979	G/G	A/G	G/G	Affx-88766901	2D	71386197
AX-109376985	C/C	T/C	T/T	Affx-109711555	2D	591179908
AX-109378474	C/C	T/C	T/T	Affx-111440573	2D	123990046
AX-109379330	G/G	A/G	G/G	Affx-109965218	2D	186622046
AX-109401533	T/C	C/C	T/T	Affx-88691548	2D	78571676
AX-109402447	A/A	G/G	A/A	Affx-109042822	2D	50846513
AX-109410927	C/C	C/C	C/C	Affx-109212711	2D	151726940
AX-109410970	G/G	A/G	G/G	Affx-111519574	2D	22460303
AX-109412860	T/T	T/G	G/G	Affx-111422059	2D	539234388
AX-109417243	G/G	A/G	A/A	Affx-110030941	2D	370121814
AX-109419238	G/G	A/G	A/A	Affx-88390686	2D	523632941
AX-109421761	C/C	C/C	T/T	Affx-111900003	2D	546175964
AX-109422294	T/T	T/C	T/T	Affx-109602631	2D	19676996
AX-109422396	C/C	T/C	T/T	Affx-88708192	2D	144288086
AX-109431101	A/A	A/A	A/A	Affx-110480907	2D	2598501
AX-109434675	G/G	A/G	G/G	Affx-88778156	2D	293928572
AX-109437900	C/C	T/C	C/C	Affx-110548276	2D	232228979
AX-109438856	A/A	A/A	G/G	Affx-111533566	2D	555166009
AX-109441212	C/C	C/C	C/C	Affx-110535852	2D	11035756
AX-109444255	C/C	A/C	C/C	Affx-110024180	2D	151645004
AX-109454081	A/G	A/G	A/G	Affx-110115280	2D	621610433
AX-109455709	G/G	A/G	G/G	Affx-108887090	2D	160826799
AX-109458829	A/A	A/G	G/G	Affx-109553670	2D	586282931
AX-109459272	A/A	A/C	C/C	Affx-111598077	2D	534493100
AX-109473421	T/T	T/C	C/C	Affx-111297789	2D	542135244
AX-109482558	T/T	C/C	T/T	Affx-109091670	2D	273001160
AX-109505933	C/C	T/C	C/C	Affx-109338630	2D	109036346
AX-109517255	G/G	A/G	G/G	Affx-109845386	2D	87783745
AX-109541787	T/T	T/C	T/T	Affx-88648758	2D	356727367
AX-109546505	C/C	T/C	C/C	Affx-111294267	2D	85825646
AX-109550867	G/G	G/G	A/A	Affx-111523150	2D	122036051
AX-109560578	C/C	T/C	C/C	Affx-111295174	2D	618154353
AX-109560725	A/A	A/G	A/A	Affx-111292628	2D	79427449
AX-109580345	G/G	G/G	G/G	Affx-110646555	2D	423551843
AX-109589399	G/G	A/G	G/G	Affx-111239738	2D	77006076
AX-109615884	G/G	T/T	T/T	Affx-110933641	2D	524338392

AX-109627624	C/C	T/C	T/T	Affx-110592393	2D	121209049
AX-109634352	T/T	T/G	T/T	Affx-111725229	2D	61450231
AX-109684297	A/A	A/G	A/A	Affx-109769478	2D	197289919
AX-109722609	T/T	T/C	T/T	Affx-109270483	2D	241341358
AX-109724216	G/G	A/G	A/A	Affx-109559747	2D	555510230
AX-109746831	A/A	A/G	A/G	Affx-109175212	2D	71776011
AX-109783146	C/C	C/G	G/G	Affx-109933968	2D	528197326
AX-109786315	G/G	A/G	A/A	Affx-109654209	2D	574823162
AX-109791414	C/C	C/G	C/C	Affx-111872546	2D	440626870
AX-109809848	G/G	T/G	G/G	Affx-109585924	2D	606926893
AX-109817248	T/T	T/G	T/T	Affx-111415821	2D	355172018
AX-109820081	G/G	A/G	A/A	Affx-109168926	2D	552953078
AX-109820955	A/G	A/G	A/G	Affx-110710079	2D	640025566
AX-109822169	A/C	A/C	C/C	Affx-111154426	2D	577544875
AX-109822660	T/T	T/C	T/T	Affx-111846932	2D	19621408
AX-109823171	C/C	T/C	T/T	Affx-109983469	2D	351982131
AX-109827976	G/G	A/G	A/A	Affx-109011695	2D	593409274
AX-109832985	A/A	A/G	G/G	Affx-111703797	2D	561139244
AX-109835020	C/C	T/C	T/T	Affx-88673348	2D	299080190
AX-109841610	A/A	A/G	A/A	Affx-110801286	2D	76993557
AX-109842248	C/C	T/C	C/C	Affx-111226438	2D	23102530
AX-109846478	G/G	A/G	A/A	Affx-108967331	2D	508137964
AX-109853546	C/C	T/C	C/C	Affx-111722504	2D	433687148
AX-109853665	G/G	C/G	G/G	Affx-110209498	2D	470072254
AX-109870453	T/T	T/C	C/C	Affx-111198697	2D	610628212
AX-109875224	C/C	T/C	C/C	Affx-109493703	2D	526252853
AX-109875511	G/G	A/G	G/G	Affx-111565938	2D	628905883
AX-109877450	A/C	A/C	C/C	Affx-110565408	2D	582013112
AX-109882185	G/G	A/G	G/G	Affx-88544103	2D	9366449
AX-109889129	A/A	A/T	A/A	Affx-110441648	2D	632974015
AX-109894807	C/C	T/C	C/C	Affx-111881645	2D	602513015
AX-109906393	C/C	T/T	C/C	Affx-110575483	2D	50127931
AX-109911091	T/T	C/C	C/C	Affx-109989853	2D	521530466
AX-109911369	G/G	A/G	G/G	Affx-110292877	2D	18201989
AX-109912977	G/G	A/G	G/G	Affx-109539124	2D	183101416
AX-109913646	A/A	A/A	G/G	Affx-109216856	2D	334261225
AX-109925088	G/G	A/G	G/G	Affx-111707410	2D	250770132
AX-109926918	G/G	A/G	G/G	Affx-109154705	2D	494312828
AX-109928351	T/T	T/C	T/C	Affx-111019533	2D	593129985
AX-109929648	C/C	T/T	C/C	Affx-110168899	2D	77565614
AX-109936423	G/G	G/G	G/G	Affx-110541769	2D	465647792
AX-109945455	G/G	A/G	A/A	Affx-110947196	2D	546174846
AX-109946720	C/C	A/C	C/C	Affx-110417933	2D	110666440
AX-109953195	C/C	T/C	C/C	Affx-88682812	2D	393560039
AX-109954296	A/G	A/G	A/G	Affx-109326527	2D	634850543
AX-109970191	A/A	A/G	G/G	Affx-110217537	2D	140759212
AX-109981832	C/C	T/C	C/C	Affx-108884901	2D	487626614
AX-109988452	C/C	T/C	C/C	Affx-111003373	2D	186630190
AX-109990903	G/G	A/G	G/G	Affx-109183243	2D	90366014
AX-110003319	C/C	T/C	C/C	Affx-111928831	2D	228813074
AX-110032854	G/G	A/G	G/G	Affx-110669595	2D	320499368
AX-110041776	T/T	T/C	T/T	Affx-111244785	2D	8334913
AX-110042451	C/C	T/C	C/C	Affx-110408787	2D	248962036
AX-110049677	T/T	T/C	T/T	Affx-111170165	2D	217011420

AX-110071222	T/T	T/C	T/T	Affx-109365354	2D	47428769
AX-110072786	C/C	T/C	C/C	Affx-109411687	2D	37041235
AX-110078020	T/C	C/C	T/T	Affx-110541345	2D	579898378
AX-110092834	C/C	T/C	T/C	Affx-110239439	2D	647673443
AX-110124878	A/A	A/G	A/A	Affx-109057815	2D	80202160
AX-110173236	C/C	T/C	T/T	Affx-111679441	2D	179865285
AX-110190135	A/A	A/G	A/A	Affx-109994660	2D	131360352
AX-110202788	G/G	G/G	G/G	Affx-110678852	2D	283507644
AX-110206836	T/T	T/C	T/T	Affx-110877810	2D	52382103
AX-110219513	G/G	T/G	G/G	Affx-110705271	2D	160838611
AX-110231229	T/T	T/C	T/T	Affx-88529355	2D	334996622
AX-110234653	T/T	T/C	T/T	Affx-110552610	2D	42880021
AX-110236383	C/C	T/C	C/C	Affx-109663876	2D	305333853
AX-110242942	A/A	A/C	C/C	Affx-110977213	2D	150731068
AX-110244413	A/A	A/G	G/G	Affx-111119171	2D	103670886
AX-110264747	C/C	C/C	C/C	Affx-109900949	2D	234003612
AX-110271360	G/G	A/G	G/G	Affx-111392949	2D	67176552
AX-110277684	C/C	C/C	C/C	Affx-111582982	2D	620599311
AX-110288603	G/G	A/A	A/A	Affx-110689718	2D	362905975
AX-110289516	G/G	A/G	G/G	Affx-110818034	2D	57255828
AX-110314714	T/T	T/C	T/T	Affx-110507534	2D	24070633
AX-110360323	A/A	A/G	A/A	Affx-110872243	2D	116589667
AX-110360420	T/T	T/C	T/T	Affx-110319718	2D	297174340
AX-110384776	C/C	T/C	C/C	Affx-111148973	2D	45523435
AX-110385388	C/G	C/G	C/G	Affx-110047648	2D	631951336
AX-110388126	A/A	A/G	G/G	Affx-109178475	2D	572405659
AX-110391227	A/G	A/G	G/G	Affx-108883361	2D	594542718
AX-110399827	C/C	T/C	C/C	Affx-111318592	2D	17800534
AX-110405805	A/A	A/G	A/A	Affx-110087485	2D	324888442
AX-110407943	T/T	T/G	T/T	Affx-111152738	2D	356481524
AX-110410377	C/C	T/C	C/C	Affx-109918786	2D	520533216
AX-110411457	A/A	A/G	G/G	Affx-109638707	2D	489715115
AX-110415488	G/G	C/G	G/G	Affx-109087353	2D	66559089
AX-110419159	C/G	C/G	C/G	Affx-110951941	2D	629204744
AX-110420393	G/G	C/G	C/C	Affx-88613794	2D	577344499
AX-110423675	T/T	T/C	C/C	Affx-110673330	2D	516638982
AX-110426079	C/C	A/C	A/A	Affx-108944229	2D	618146414
AX-110426295	G/G	C/G	G/G	Affx-110355673	2D	282918785
AX-110434103	A/A	A/G	G/G	Affx-110464778	2D	560932543
AX-110434251	G/G	T/G	G/G	Affx-111082119	2D	237684760
AX-110434845	A/C	A/A	A/A	Affx-109105531	2D	577631199
AX-110436671	T/C	T/C	T/C	Affx-109137097	2D	64609697
AX-110436925	C/C	T/C	C/C	Affx-110347728	2D	151920939
AX-110443165	C/C	T/T	C/C	Affx-88492459	2D	378056225
AX-110444796	G/G	T/G	G/G	Affx-110555984	2D	163496730
AX-110458487	G/G	A/G	G/G	Affx-109726074	2D	414386186
AX-110463874	T/T	T/C	T/T	Affx-88710478	2D	413688538
AX-110465217	A/C	A/C	A/A	Affx-88517635	2D	587305172
AX-110474977	C/C	T/C	C/C	Affx-111843851	2D	577533263
AX-110477999	C/C	A/C	C/C	Affx-111997229	2D	600764072
AX-110481984	G/G	G/G	A/A	Affx-109304750	2D	345093180
AX-110505028	C/C	T/T	T/T	Affx-111784172	2D	574113228
AX-110513479	A/A	A/C	A/A	Affx-111120341	2D	498104469
AX-110514436	T/T	T/C	T/T	Affx-110598892	2D	83857224

AX-110514662	C/C	T/C	C/C	Affx-110750198	2D	478702045
AX-110519154	G/G	A/G	A/A	Affx-111749631	2D	508189126
AX-110535834	C/C	A/C	C/C	Affx-110835979	2D	562734588
AX-110540000	G/G	A/G	G/G	Affx-109180191	2D	642125777
AX-110544009	C/C	T/C	C/C	Affx-110046593	2D	95895661
AX-110554181	G/G	G/G	G/G	Affx-109393456	2D	562366231
AX-110554523	G/G	C/G	C/C	Affx-111368922	2D	613105551
AX-110558245	C/C	T/C	C/C	Affx-109787329	2D	422881650
AX-110558888	G/G	A/G	G/G	Affx-88552032	2D	414990339
AX-110564336	C/G	C/G	G/G	Affx-111308109	2D	602648059
AX-110574726	A/A	G/G	A/A	Affx-111356972	2D	633299660
AX-110577922	G/G	T/G	G/G	Affx-88431863	2D	480659177
AX-110585234	G/G	T/G	G/G	Affx-88354762	2D	243666989
AX-110587475	G/G	A/G	G/G	Affx-110867948	2D	203503713
AX-110627926	A/A	A/G	G/G	Affx-88437428	2D	590331216
AX-110628312	G/G	A/G	A/A	Affx-109719985	2D	555919550
AX-110630968	T/G	G/G	T/G	Affx-111190009	2D	622489233
AX-110638095	A/A	A/G	G/G	Affx-111008258	2D	122701570
AX-110656579	G/G	T/G	G/G	Affx-110013646	2D	109830985
AX-110668262	T/T	T/C	C/C	Affx-109021137	2D	577343489
AX-110675161	T/T	T/C	C/C	Affx-111030409	2D	387562470
AX-110676927	G/G	A/G	G/G	Affx-111153568	2D	453442208
AX-110678877	G/G	G/G	G/G	Affx-110227528	2D	247677909
AX-110682929	T/T	T/C	T/T	Affx-111788341	2D	446928838
AX-110715550	G/G	C/G	G/G	Affx-109359540	2D	34067072
AX-110715898	T/T	T/C	T/T	Affx-111721704	2D	24476165
AX-110746935	T/T	T/C	T/T	Affx-111265798	2D	223207130
AX-110758731	T/T	T/C	T/T	Affx-109406112	2D	217041669
AX-110773416	T/T	T/C	C/C	Affx-109123898	2D	388580229
AX-110773527	T/T	T/C	T/T	Affx-111525982	2D	424667394
AX-110781772	G/G	A/G	A/A	Affx-110120515	2D	221937571
AX-110818422	G/G	A/G	A/A	Affx-111350779	2D	151467684
AX-110823770	C/C	C/G	C/C	Affx-111898728	2D	606602875
AX-110828891	C/C	C/C	A/C	Affx-108989712	2D	573464009
AX-110833961	C/C	C/C	T/T	Affx-110792913	2D	518184423
AX-110840332	C/C	C/C	C/C	Affx-109038793	2D	500949522
AX-110868681	C/C	C/C	C/C	Affx-110078869	2D	455977525
AX-110868690	A/A	A/A	A/A	Affx-110514529	2D	341265706
AX-110872666	C/C	T/C	C/C	Affx-109682219	2D	143234332
AX-110876453	C/C	T/C	C/C	Affx-110160293	2D	651674727
AX-110887735	C/C	T/C	C/C	Affx-109102440	2D	277558980
AX-110891632	C/C	C/C	C/C	Affx-111157962	2D	34094836
AX-110897560	A/A	A/T	A/A	Affx-110641753	2D	151444866
AX-110899429	C/C	T/C	C/C	Affx-110120876	2D	487313590
AX-110902523	G/G	G/G	G/G	Affx-109127492	2D	477296885
AX-110912704	G/G	A/G	G/G	Affx-111508429	2D	389286831
AX-110918206	T/T	T/C	T/C	Affx-109412711	2D	563942465
AX-110926874	T/T	T/G	T/T	Affx-110338680	2D	617337839
AX-110929430	G/G	A/G	A/A	Affx-110370623	2D	605802782
AX-110929471	G/G	C/G	G/G	Affx-111963080	2D	502198648
AX-110931635	T/T	T/G	G/G	Affx-109011017	2D	597322454
AX-110951587	T/T	T/G	G/G	Affx-110597840	2D	551877542
AX-110952458	C/C	T/C	T/T	Affx-109537542	2D	564387100
AX-110955068	T/T	C/C	C/C	Affx-110018913	2D	517058180

AX-110958528	A/A	A/G	A/A	Affx-111656321	2D	114502376
AX-110972681	C/C	C/G	C/C	Affx-110887784	2D	60642438
AX-110977542	T/T	T/C	T/T	Affx-111190155	2D	175283182
AX-110984672	G/G	G/G	G/G	Affx-109070693	2D	22819682
AX-110986870	C/C	T/C	C/C	Affx-110205394	2D	18752383
AX-110987979	A/A	A/G	A/A	Affx-111932420	2D	43369979
AX-110993528	T/T	T/C	T/T	Affx-111796937	2D	77111540
AX-110996701	G/G	A/G	A/G	Affx-111566152	2D	533065332
AX-111004947	C/C	C/C	T/T	Affx-110805404	2D	612450178
AX-111007074	C/G	C/G	C/C	Affx-111721544	2D	364875707
AX-111010133	A/A	A/G	A/A	Affx-111693727	2D	303387364
AX-111010483	G/G	T/G	G/G	Affx-111588299	2D	24564422
AX-111011450	C/C	T/C	C/C	Affx-110569670	2D	499834862
AX-111024861	G/G	A/G	G/G	Affx-111133882	2D	373807033
AX-111025542	A/A	A/C	C/C	Affx-111713754	2D	595120668
AX-111039921	C/C	C/G	G/G	Affx-111998281	2D	381880185
AX-111042061	G/G	A/G	G/G	Affx-111218257	2D	45800408
AX-111045226	T/T	T/T	T/T	Affx-111281762	2D	24546128
AX-111057518	C/C	T/C	C/C	Affx-109679673	2D	533744404
AX-111059742	A/A	A/G	G/G	Affx-110052128	2D	596001428
AX-111059876	G/G	A/G	G/G	Affx-110735768	2D	650882131
AX-111066402	A/A	A/G	A/A	Affx-109304764	2D	52106312
AX-111070763	G/G	C/G	G/G	Affx-108897893	2D	462955613
AX-111071533	C/C	G/G	C/C	Affx-111891243	2D	19083561
AX-111079418	C/C	T/T	C/C	Affx-110131165	2D	247055029
AX-111083601	G/G	A/G	A/A	Affx-111858744	2D	331914363
AX-111085838	C/C	T/C	C/C	Affx-111530658	2D	480308268
AX-111097388	C/C	T/C	C/C	Affx-88412145	2D	138711090
AX-111097743	C/C	T/C	C/C	Affx-109299303	2D	208904341
AX-111112336	T/T	T/C	C/C	Affx-108884420	2D	582143448
AX-111113779	G/G	G/G	G/G	Affx-109184941	2D	385341175
AX-111114755	G/G	C/G	G/G	Affx-111122430	2D	182101983
AX-111115549	T/T	A/T	A/A	Affx-110902427	2D	522543523
AX-111116269	G/G	A/G	A/A	Affx-109171496	2D	345970097
AX-111120304	A/A	A/C	C/C	Affx-111615240	2D	391281094
AX-111121096	G/G	A/G	G/G	Affx-111240684	2D	577441213
AX-111124756	A/A	A/G	G/G	Affx-111344430	2D	509222448
AX-111125241	C/C	T/C	C/C	Affx-112005028	2D	378768744
AX-111133298	A/T	A/A	A/A	Affx-110981667	2D	531493394
AX-111134429	C/C	C/G	G/G	Affx-111076165	2D	1848340
AX-111138700	C/C	T/C	C/C	Affx-111630784	2D	419435813
AX-111139679	G/G	C/G	G/G	Affx-109296862	2D	605433936
AX-111141477	A/A	A/G	G/G	Affx-109141390	2D	543590608
AX-111143909	C/C	T/C	T/T	Affx-110061461	2D	175760610
AX-111151139	C/C	A/C	C/C	Affx-111683142	2D	411476541
AX-111151907	G/G	T/G	G/G	Affx-110727906	2D	95521662
AX-111154051	C/C	C/G	C/C	Affx-110429018	2D	28424689
AX-111155965	T/T	T/C	T/T	Affx-109366118	2D	429503499
AX-111159673	A/G	G/G	A/A	Affx-111986946	2D	13221455
AX-111169393	A/A	A/G	A/A	Affx-110606225	2D	121062796
AX-111174437	C/C	T/C	C/C	Affx-110309907	2D	78089215
AX-111193092	G/G	T/T	T/T	Affx-111440763	2D	11400036
AX-111197188	C/C	T/C	C/C	Affx-108986854	2D	600035515
AX-111202731	A/A	A/G	G/G	Affx-110819283	2D	593798107

AX-111224483	G/G	A/G	G/G	Affx-111721055	2D	24267332
AX-111226837	A/A	G/G	A/A	Affx-108917301	2D	110850040
AX-111237877	G/G	A/G	G/G	Affx-110690697	2D	33946250
AX-111248112	T/T	T/T	T/T	Affx-110495298	2D	379695980
AX-111249650	G/G	A/G	G/G	Affx-110727955	2D	234099294
AX-111262216	T/T	T/G	G/G	Affx-109331409	2D	150731934
AX-111271035	C/C	C/C	C/C	Affx-110091145	2D	14114787
AX-111283000	T/T	T/C	T/T	Affx-110255754	2D	37961972
AX-111295182	T/C	C/C	C/C	Affx-109735392	2D	556434057
AX-111297034	C/C	T/C	C/C	Affx-109120922	2D	76011568
AX-111298592	T/T	T/C	T/T	Affx-110004320	2D	363522891
AX-111312852	A/A	A/G	A/A	Affx-111514404	2D	331961215
AX-111318850	T/T	T/C	C/C	Affx-110197147	2D	536937814
AX-111341034	G/G	T/G	T/T	Affx-111357584	2D	545532570
AX-111348648	G/G	A/G	G/G	Affx-111394684	2D	481354508
AX-111418357	A/A	A/G	A/A	Affx-111152282	2D	355905978
AX-111418408	T/T	T/C	T/T	Affx-109014252	2D	359378675
AX-111430851	T/T	T/C	T/T	Affx-111699510	2D	243794549
AX-111437866	G/G	A/G	G/G	Affx-110960662	2D	248323103
AX-111440986	G/G	A/G	G/G	Affx-110597328	2D	317248788
AX-111443697	C/C	T/C	C/C	Affx-110486168	2D	194982270
AX-111446644	C/C	T/C	C/C	Affx-111969575	2D	24569603
AX-111452151	A/A	A/G	A/A	Affx-109398220	2D	599789059
AX-111453536	C/C	T/C	T/T	Affx-109656119	2D	552968702
AX-111461589	C/G	C/G	C/G	Affx-109494558	2D	645368910
AX-111467211	G/G	C/G	C/C	Affx-88766136	2D	558333935
AX-111491026	G/G	C/G	C/C	Affx-111807646	2D	529540670
AX-111491672	C/C	C/G	C/C	Affx-92444590	2D	634476812
AX-111497459	G/G	C/G	C/C	Affx-109462298	2D	548602129
AX-111512423	A/A	A/G	A/A	Affx-111522306	2D	30581329
AX-111516380	T/T	T/C	T/T	Affx-88424382	2D	519126261
AX-111519867	G/G	C/G	C/C	Affx-110692051	2D	611893236
AX-111533760	C/C	C/G	C/C	Affx-110629706	2D	12549155
AX-111536272	T/T	T/C	T/T	Affx-110726677	2D	23047412
AX-111538812	A/A	A/G	G/G	Affx-109500359	2D	544737384
AX-111542571	C/C	C/G	C/C	Affx-110719962	2D	630384909
AX-111544559	A/C	A/C	A/C	Affx-110098257	2D	640356954
AX-111545463	T/T	T/C	T/T	Affx-110986098	2D	74975412
AX-111559873	C/C	T/C	C/C	Affx-110757115	2D	79095877
AX-111566947	A/A	A/G	G/G	Affx-110299878	2D	585677429
AX-111567609	T/T	T/C	C/C	Affx-109548527	2D	125107110
AX-111569013	A/A	A/G	A/A	Affx-109198379	2D	50749420
AX-111574425	T/T	T/G	T/T	Affx-88444841	2D	32366296
AX-111576421	C/C	A/C	C/C	Affx-108993979	2D	351967298
AX-111593778	C/C	T/C	C/C	Affx-110482175	2D	601216007
AX-111594338	G/G	A/G	G/G	Affx-109479898	2D	248490614
AX-111596411	T/T	T/C	T/T	Affx-111318507	2D	68729483
AX-111601893	T/T	A/T	A/A	Affx-110612635	2D	534761567
AX-111603747	C/C	T/C	C/C	Affx-88750805	2D	107898272
AX-111605724	A/A	A/G	A/A	Affx-110257354	2D	77128347
AX-111613674	A/A	A/C	C/C	Affx-111263151	2D	515550356
AX-111620087	G/G	T/G	T/T	Affx-109123649	2D	578874114
AX-111620976	T/T	T/T	C/C	Affx-109025895	2D	594545548
AX-111632123	T/T	T/C	C/C	Affx-111029587	2D	576580701

AX-111638682	A/A	A/G	A/A	Affx-111200465	2D	353395755
AX-111660097	A/C	A/C	A/A	Affx-110061961	2D	603492664
AX-111662504	G/G	A/G	G/G	Affx-109192993	2D	325051064
AX-111665890	C/C	T/C	T/T	Affx-88534519	2D	119385512
AX-111678972	G/G	C/G	G/G	Affx-110739284	2D	302779959
AX-111684683	G/G	A/G	G/G	Affx-108877981	2D	566768726
AX-111692399	A/G	G/G	A/G	Affx-110014002	2D	28960260
AX-111694202	G/G	A/G	G/G	Affx-110826435	2D	127299989
AX-111694875	C/C	A/C	A/A	Affx-109740651	2D	597123888
AX-111706752	A/A	A/G	A/A	Affx-109887115	2D	56204366
AX-111717371	G/G	A/G	G/G	Affx-110342186	2D	375366803
AX-111722527	C/C	C/C	C/C	Affx-111861062	2D	527923767
AX-111726891	C/C	C/C	C/C	Affx-111406797	2D	23025187
AX-111734356	T/T	T/T	T/T	Affx-109627683	2D	22965710
AX-111734656	C/C	T/C	C/C	Affx-109013026	2D	383553293
AX-111761440	G/G	A/A	A/A	Affx-110607060	2D	609508490
AX-111763561	T/T	T/C	T/T	Affx-88472404	2D	64050283
AX-111769456	T/T	T/C	T/T	Affx-110717050	2D	59334582
AX-111771147	G/G	A/G	G/G	Affx-110420848	2D	55275948
AX-111810220	C/C	C/C	C/C	Affx-110938169	2D	304302930
AX-111829004	G/G	A/G	G/G	Affx-109285896	2D	233711385
AX-111830625	G/G	T/G	G/G	Affx-109997862	2D	463337085
AX-111838598	C/C	T/T	T/T	Affx-88546702	2D	351120485
AX-111873000	T/T	T/G	T/T	Affx-110814806	2D	490800057
AX-111906843	G/G	T/G	G/G	Affx-111487988	2D	19630627
AX-111907126	C/C	T/C	C/C	Affx-108871391	2D	40518152
AX-111912426	G/G	A/A	G/G	Affx-111621212	2D	46570644
AX-111914694	C/C	T/C	C/C	Affx-88679985	2D	66117477
AX-111914966	G/G	T/G	G/G	Affx-110557464	2D	87846939
AX-111916695	C/C	T/C	C/C	Affx-110286670	2D	247089350
AX-111924290	A/A	A/G	A/A	Affx-110573948	2D	194282758
AX-111924697	G/G	A/G	G/G	Affx-109161258	2D	396421052
AX-111956072	T/T	T/C	T/T	Affx-111736294	2D	34428838
AX-111970711	G/G	A/G	G/G	Affx-110752880	2D	290959201
AX-111976176	G/G	A/G	A/A	Affx-110271751	2D	359033765
AX-111988840	G/G	A/G	G/G	Affx-111125639	2D	296092350
AX-112286202	C/C	C/C	C/C	Affx-112315030	2D	117112325
AX-112287601	T/T	T/C	T/T	Affx-112315227	2D	56805639
AX-112290193	G/G	A/G	G/G	Affx-112314630	2D	83161369
AX-182075166	G/G	A/G	G/G	Affx-111057960	2D	19621355
AX-182076870	G/G	G/G	G/G	Affx-109059972	2D	76990925
AX-182077400	A/G	A/G	A/A	Affx-111391262	2D	572794214
AX-182077589	G/G	G/G	G/G	Affx-92593614	2D	34229911
AX-182097730	G/G	T/G	G/G	Affx-108946491	2D	233753762
AX-182101001	G/G	G/G	G/G	Affx-109383401	2D	577434769
AX-182108364	A/A	A/G	A/A	Affx-110409490	2D	234198193
AX-182119126	C/C	T/C	C/C	Affx-111890012	2D	234144413
AX-182151054	T/C	T/T	T/C	Affx-472299355	2D	43374897
AX-182176330	A/A	A/G	A/G	Affx-472321732	2D	642871929
AX-86163393	C/C	T/T	C/C	Affx-109371264	2D	35683268
AX-86166630	A/A	A/G	A/A	Affx-92874397	2D	66759628
AX-86171316	A/G	A/A	G/G	Affx-88520727	2D	34231554
AX-86172904	C/C	T/C	C/C	Affx-112313644	2D	63570753
AX-86173747	T/T	C/C	T/T	Affx-92572356	2D	170449545

AX-86174700	A/G	G/G	A/G	Affx-88773542	2D	20768620
AX-86174781	A/A	A/G	A/A	Affx-88362949	2D	62023929
AX-86180694	G/G	G/G	G/G	Affx-111232269	2D	30681234
AX-86184903	G/G	A/G	G/G	Affx-110713265	2D	310301211
AX-89322641	A/A	A/G	A/A	Affx-88353662	2D	185668957
AX-89375500	A/A	A/G	A/A	Affx-88407770	2D	318423790
AX-89469352	G/G	C/G	G/G	Affx-92601214	2D	13542925
AX-89483789	G/G	A/G	A/A	Affx-110892864	2D	119801484
AX-89496787	C/G	C/C	C/G	Affx-88529659	2D	501058902
AX-89536198	C/C	T/C	T/T	Affx-88569170	2D	130844477
AX-89563243	G/G	A/G	G/G	Affx-110326784	2D	57963281
AX-89564230	C/C	C/G	C/C	Affx-88597285	2D	6726945
AX-89567255	T/T	T/C	T/T	Affx-88600318	2D	354962161
AX-89629279	G/G	C/G	C/C	Affx-88662456	2D	535219320
AX-89690184	C/C	T/C	C/C	Affx-88722941	2D	419865085
AX-89716596	A/A	A/G	A/A	Affx-111345000	2D	553894783
AX-89728114	T/T	T/C	T/T	Affx-88760808	2D	26344368
AX-89745236	C/C	C/C	C/C	Affx-88777924	2D	15730641
AX-94393403	T/T	T/T	T/T	Affx-92787583	2D	80820394
AX-94401246	C/C	T/T	T/T	Affx-92482862	2D	97600655
AX-94407958	T/C	T/T	T/C	Affx-92506034	2D	33952856
AX-94474729	G/G	A/G	A/A	Affx-92855624	2D	584545797
AX-94475323	C/C	T/C	C/C	Affx-92422357	2D	474889537
AX-94496990	C/C	C/C	C/C	Affx-88354888	2D	552141388
AX-94509548	C/C	C/G	C/C	Affx-92449416	2D	34226986
AX-94515955	C/C	T/T	T/C	Affx-92581270	2D	645833803
AX-94519027	C/C	T/C	C/C	Affx-92305371	2D	33952292
AX-94546639	C/C	T/C	C/C	Affx-92689838	2D	234350029
AX-94566110	T/T	G/G	T/T	Affx-92473706	2D	642201396
AX-94568362	T/C	T/C	T/T	Affx-92250141	2D	580240187
AX-94569767	C/C	G/G	C/C	Affx-92874964	2D	75506472
AX-94575160	C/C	C/C	C/C	Affx-92861585	2D	108922133
AX-94646652	T/T	T/G	T/T	Affx-88531141	2D	103466072
AX-94651272	A/A	A/A	A/A	Affx-92221041	2D	33952935
AX-94661042	A/G	A/A	G/G	Affx-88384271	2D	77131618
AX-94662189	C/C	C/C	C/C	Affx-88636855	2D	74929599
AX-94669260	G/G	G/G	C/G	Affx-92651766	2D	577455390
AX-94674489	C/G	G/G	C/C	Affx-92483441	2D	614811456
AX-94679692	C/C	C/G	C/C	Affx-92810299	2D	569949927
AX-94685617	G/G	G/G	G/G	Affx-92924741	2D	637650719
AX-94726031	A/A	A/G	A/A	Affx-92885618	2D	33905270
AX-94786386	A/G	A/G	A/G	Affx-92882715	2D	573969935
AX-94789982	A/A	A/G	A/G	Affx-92598237	2D	588226934
AX-94790883	T/T	T/C	C/C	Affx-92110498	2D	594551885
AX-94824663	A/G	A/G	G/G	Affx-92372946	2D	594549850
AX-94836887	T/T	T/T	T/T	Affx-92361652	2D	22963332
AX-94852677	G/G	G/G	G/G	Affx-92860363	2D	33952933
AX-94863655	A/A	G/G	A/G	Affx-92846165	2D	594547629
AX-94889252	G/G	A/A	G/G	Affx-92671916	2D	22963320
AX-94898545	C/C	T/C	C/C	Affx-92258076	2D	29844281
AX-94907276	T/C	C/C	T/C	Affx-92280000	2D	536150992
AX-94920651	G/G	A/G	G/G	Affx-92485462	2D	565182924
AX-94934527	A/A	G/G	A/G	Affx-92811160	2D	638367747
AX-94939121	C/C	C/G	C/G	Affx-92844802	2D	568410165

AX-94991064	G/G	T/G	G/G	Affx-92885392	2D	648470041
AX-95019559	A/A	G/G	G/G	Affx-92542596	2D	594551735
AX-95108787	T/C	T/T	T/T	Affx-88675533	2D	635599182
AX-95151743	C/C	C/C	C/C	Affx-110042284	2D	20769354
AX-95159933	C/C	T/T	T/C	Affx-92462228	2D	577454882
AX-95163727	C/C	C/C	C/C	Affx-88784433	2D	157138592
AX-95182465	T/C	T/T	C/C	Affx-92354241	2D	594552008
AX-95629299	A/A	A/G	G/G	Affx-88519911	2D	344297544
AX-95658742	T/T	T/T	T/T	Affx-110892515	2D	61979535
AX-108726616	G/G	T/G	G/G	Affx-111238762	3A	217573701
AX-108728793	T/T	C/C	C/C	Affx-108924255	3A	539295590
AX-108731494	T/T	T/G	G/G	Affx-111931532	3A	69058163
AX-108732808	A/A	A/G	G/G	Affx-109669004	3A	658754178
AX-108736878	T/T	T/G	T/T	Affx-111159370	3A	67157110
AX-108737998	C/C	C/G	C/C	Affx-109190349	3A	377243317
AX-108738634	C/C	T/C	C/C	Affx-88780750	3A	673864200
AX-108742089	A/A	A/G	A/A	Affx-109682419	3A	731718623
AX-108749058	A/A	A/C	A/A	Affx-110544354	3A	251999289
AX-108753757	C/C	C/G	C/C	Affx-109721373	3A	532936947
AX-108757118	C/C	T/C	T/T	Affx-109553718	3A	520604825
AX-108759149	T/T	T/T	T/T	Affx-109240587	3A	616598201
AX-108762191	G/G	T/G	T/T	Affx-88804821	3A	703907794
AX-108772543	T/T	C/C	T/T	Affx-110614236	3A	532667811
AX-108781282	A/A	A/G	A/A	Affx-110469241	3A	15823731
AX-108782307	G/G	A/G	G/G	Affx-111558830	3A	623396069
AX-108783249	T/T	T/C	T/T	Affx-110855968	3A	100023583
AX-108789206	C/C	T/C	C/C	Affx-111984157	3A	723320227
AX-108793374	C/C	C/C	C/C	Affx-110995095	3A	58042019
AX-108794102	C/C	T/C	C/C	Affx-111804410	3A	682975023
AX-108803311	C/C	T/C	C/C	Affx-111595665	3A	27578944
AX-108807007	T/T	T/G	T/T	Affx-111067319	3A	236371664
AX-108808975	A/A	A/A	A/A	Affx-111553387	3A	657199121
AX-108812379	C/C	C/C	C/C	Affx-110170865	3A	283121917
AX-108814864	G/G	A/G	G/G	Affx-111765301	3A	46459334
AX-108823960	C/C	A/C	C/C	Affx-109320679	3A	534207068
AX-108829349	T/T	T/C	C/C	Affx-109587920	3A	95900329
AX-108840355	A/C	A/C	A/C	Affx-88403101	3A	747610277
AX-108840867	T/T	T/C	T/T	Affx-108987099	3A	368011188
AX-108841461	T/T	T/C	C/C	Affx-111252279	3A	684584230
AX-108844656	A/A	C/C	A/A	Affx-110850373	3A	394646940
AX-108850617	C/C	T/C	C/C	Affx-109808671	3A	732759410
AX-108851173	T/T	T/C	T/T	Affx-111786418	3A	302101106
AX-108852387	T/T	T/C	T/T	Affx-109470208	3A	497649046
AX-108860141	C/C	T/C	C/C	Affx-111223221	3A	713551836
AX-108861464	C/C	T/C	C/C	Affx-88656577	3A	528526784
AX-108863292	C/C	T/C	C/C	Affx-88653100	3A	213601013
AX-108863620	C/C	A/C	C/C	Affx-109961998	3A	696288052
AX-108868845	T/T	T/C	T/T	Affx-109591552	3A	435048800
AX-108870115	G/G	A/G	A/A	Affx-109012886	3A	645147145
AX-108871682	C/C	C/G	C/C	Affx-109317723	3A	691471004
AX-108872401	T/T	T/C	C/C	Affx-109258224	3A	659354592
AX-108873891	G/G	C/G	G/G	Affx-111585841	3A	733865298
AX-108885694	C/C	T/C	C/C	Affx-111658099	3A	142038846
AX-108887003	A/A	A/A	A/A	Affx-110923986	3A	264670869

AX-108887339	C/C	T/C	C/C	Affx-88682966	3A	714020755
AX-108887673	C/C	C/G	C/C	Affx-109172127	3A	726511279
AX-108895821	A/A	A/G	A/A	Affx-109530018	3A	373216098
AX-108897269	G/G	G/G	G/G	Affx-109206216	3A	634086482
AX-108900741	C/C	C/C	C/C	Affx-111699985	3A	69414613
AX-108903233	A/A	A/G	A/A	Affx-110605381	3A	302994545
AX-108907898	C/C	T/C	C/C	Affx-108914625	3A	548263896
AX-108916293	G/G	C/G	C/C	Affx-109771471	3A	745778401
AX-108917324	T/T	T/C	T/T	Affx-111413004	3A	562235556
AX-108922928	T/T	T/C	T/T	Affx-88672153	3A	69664698
AX-108942438	A/A	A/G	A/A	Affx-111538450	3A	218754427
AX-108942559	T/T	G/G	T/T	Affx-110368078	3A	405682568
AX-108946027	C/C	C/G	C/C	Affx-111908121	3A	667504748
AX-108948880	G/G	A/G	G/G	Affx-110212825	3A	530252404
AX-108949997	G/G	A/G	G/G	Affx-111323031	3A	601548229
AX-108950927	T/T	C/C	T/T	Affx-111443896	3A	468209196
AX-108952371	C/C	T/C	C/C	Affx-88445614	3A	346956954
AX-108954219	G/G	A/G	G/G	Affx-110256766	3A	20488129
AX-108954613	T/T	T/C	C/C	Affx-110998113	3A	661927943
AX-108965832	T/T	T/C	T/T	Affx-111241684	3A	235275601
AX-108969393	A/A	A/G	A/A	Affx-88655490	3A	356997315
AX-108975984	A/A	A/G	A/A	Affx-110024835	3A	340430384
AX-108976589	G/G	A/G	G/G	Affx-110298336	3A	526868695
AX-108982447	T/T	T/C	T/T	Affx-110066578	3A	238675957
AX-108986053	G/G	A/G	G/G	Affx-111771409	3A	36486115
AX-109035969	G/G	A/G	G/G	Affx-88634722	3A	137905111
AX-109037934	A/A	A/G	A/A	Affx-111580363	3A	664457077
AX-109051215	G/G	T/G	G/G	Affx-109797409	3A	499692658
AX-109057491	G/G	G/G	G/G	Affx-109462304	3A	626001685
AX-109082264	C/C	T/T	C/C	Affx-111228363	3A	323956037
AX-109109227	A/A	A/G	A/A	Affx-111603005	3A	478652433
AX-109110953	C/C	T/C	C/C	Affx-110225045	3A	624911306
AX-109111090	C/C	C/G	C/C	Affx-109610009	3A	665183659
AX-109270909	G/G	T/G	G/G	Affx-88696200	3A	621663454
AX-109271198	C/C	T/C	T/T	Affx-111217236	3A	521302994
AX-109274942	A/A	A/G	A/A	Affx-92231114	3A	631281663
AX-109276794	C/C	T/C	C/C	Affx-88458590	3A	749866305
AX-109279741	A/A	A/G	A/A	Affx-111327250	3A	305828190
AX-109281734	C/C	T/C	T/T	Affx-109078384	3A	744244068
AX-109285480	A/A	A/G	A/A	Affx-111179330	3A	421126525
AX-109293721	G/G	A/G	G/G	Affx-109442853	3A	53747934
AX-109295136	T/T	T/C	T/T	Affx-88602971	3A	162216499
AX-109296490	G/G	A/G	A/A	Affx-110973153	3A	750473917
AX-109296862	G/G	C/G	G/G	Affx-109844013	3A	111239566
AX-109298595	C/C	A/C	C/C	Affx-111195520	3A	623906165
AX-109306101	A/A	A/A	A/A	Affx-110578825	3A	589312417
AX-109311301	A/A	A/G	A/A	Affx-88444030	3A	56964449
AX-109314183	A/A	A/G	A/A	Affx-111978744	3A	729702071
AX-109329110	T/T	G/G	T/T	Affx-110815991	3A	726117192
AX-109333342	C/C	C/C	C/C	Affx-109311989	3A	648031222
AX-109337059	A/A	A/G	A/A	Affx-109067213	3A	348122452
AX-109340230	C/C	A/C	A/A	Affx-110646840	3A	702537849
AX-109344896	C/C	A/C	A/A	Affx-109224016	3A	702989138
AX-109345729	C/C	C/G	C/C	Affx-111249868	3A	483161152

AX-109354334	C/C	C/C	C/C	Affx-110159978	3A	718049516
AX-109354736	C/C	G/G	C/C	Affx-88429419	3A	115029271
AX-109355304	T/T	T/C	T/T	Affx-110205641	3A	364796452
AX-109358980	C/C	C/G	G/G	Affx-111582964	3A	687645012
AX-109366295	A/A	A/G	G/G	Affx-110224819	3A	594636638
AX-109372178	T/T	T/C	T/T	Affx-110595383	3A	721662185
AX-109374994	T/T	T/C	T/T	Affx-111269992	3A	735901607
AX-109378241	T/T	C/C	T/T	Affx-109335978	3A	194475049
AX-109386536	A/G	A/G	A/A	Affx-110578877	3A	706854185
AX-109390259	T/T	T/C	C/C	Affx-111840810	3A	513097221
AX-109394676	G/G	A/G	G/G	Affx-111969911	3A	565813257
AX-109399799	G/G	A/G	G/G	Affx-109267343	3A	602855338
AX-109405558	T/T	T/C	T/T	Affx-111689688	3A	710847128
AX-109405592	C/C	T/C	C/C	Affx-111229344	3A	547380944
AX-109406380	C/C	A/A	C/C	Affx-111393943	3A	17491805
AX-109408044	C/C	C/C	C/C	Affx-109511020	3A	176024198
AX-109414456	T/T	T/C	T/T	Affx-88702099	3A	669563961
AX-109415257	G/G	A/G	A/A	Affx-110182799	3A	686306116
AX-109419068	T/C	T/T	T/T	Affx-110949059	3A	484638603
AX-109422028	C/C	T/C	C/C	Affx-111157706	3A	34898050
AX-109428087	C/C	T/T	C/C	Affx-111803209	3A	717006402
AX-109437970	A/A	A/C	A/A	Affx-109932331	3A	612522271
AX-109442780	G/G	A/A	G/G	Affx-88446273	3A	185289726
AX-109445299	C/C	C/G	C/C	Affx-110325179	3A	540997680
AX-109448260	A/A	A/A	A/A	Affx-110321005	3A	21553840
AX-109464579	A/C	A/C	A/A	Affx-110983436	3A	535329985
AX-109466225	G/G	A/A	A/A	Affx-110161766	3A	709207023
AX-109468077	A/A	A/G	A/A	Affx-109901995	3A	603209772
AX-109482628	G/G	A/G	G/G	Affx-108929322	3A	183076242
AX-109484907	C/C	T/C	C/C	Affx-111655288	3A	18751927
AX-109495465	A/A	A/G	A/A	Affx-111299626	3A	313850798
AX-109501195	T/T	T/C	T/T	Affx-111175498	3A	397470897
AX-109520633	G/G	A/G	G/G	Affx-110295016	3A	55802927
AX-109530078	T/T	T/C	T/T	Affx-111009626	3A	37188265
AX-109536494	G/G	G/G	A/A	Affx-109673966	3A	487858938
AX-109540612	C/C	C/C	C/C	Affx-110488640	3A	670859827
AX-109551920	C/C	T/T	C/C	Affx-109187883	3A	727992228
AX-109552940	T/C	T/T	C/C	Affx-109487671	3A	91029792
AX-109553201	T/T	C/C	T/T	Affx-88673920	3A	61478965
AX-109556103	T/T	T/T	C/C	Affx-111751617	3A	645097534
AX-109579517	A/A	G/G	A/A	Affx-111053511	3A	17441035
AX-109580486	C/C	A/C	C/C	Affx-109499972	3A	718753717
AX-109584650	C/C	T/C	C/C	Affx-109142418	3A	431092156
AX-109596960	T/T	T/C	T/T	Affx-109274440	3A	251648006
AX-109620716	G/G	C/G	G/G	Affx-110901395	3A	525355460
AX-109621355	A/A	G/G	A/A	Affx-111308022	3A	662934359
AX-109627023	C/C	T/C	C/C	Affx-109006042	3A	447619711
AX-109637665	A/A	A/A	A/A	Affx-109572809	3A	488599278
AX-109656347	A/A	A/G	A/A	Affx-109045161	3A	626745010
AX-109817797	T/T	T/G	T/T	Affx-88747010	3A	552024688
AX-109817800	C/C	C/C	C/C	Affx-111501565	3A	164853933
AX-109823045	T/T	T/C	T/T	Affx-88596418	3A	38303804
AX-109827120	T/T	T/C	C/C	Affx-110490328	3A	683524484
AX-109828373	T/C	C/C	T/T	Affx-111979619	3A	503699208

AX-109830962	C/C	T/C	T/T	Affx-111164166	3A	688581758
AX-109839765	T/T	T/C	T/T	Affx-111398853	3A	106619127
AX-109839982	A/A	A/T	A/A	Affx-111117338	3A	208353827
AX-109849154	G/G	A/G	G/G	Affx-110586093	3A	550727993
AX-109857950	T/T	T/C	T/T	Affx-110956280	3A	612950062
AX-109862505	C/C	T/C	C/C	Affx-111927431	3A	635730117
AX-109863037	T/T	T/C	T/T	Affx-109339148	3A	208653248
AX-109868656	C/C	G/G	C/C	Affx-110506027	3A	208799533
AX-109874039	C/C	T/C	C/C	Affx-110206907	3A	701453362
AX-109874982	T/T	T/C	C/C	Affx-110730948	3A	523307960
AX-109877370	C/C	C/G	C/C	Affx-109658897	3A	12284831
AX-109878195	G/G	G/G	G/G	Affx-109107517	3A	364441736
AX-109878197	C/G	C/G	C/G	Affx-111934721	3A	22080855
AX-109879188	C/C	T/C	C/C	Affx-110189560	3A	171225718
AX-109883243	C/G	C/G	G/G	Affx-109047562	3A	739167446
AX-109885319	A/A	A/G	A/A	Affx-109352496	3A	289660992
AX-109885501	A/A	G/G	A/A	Affx-109552473	3A	185221418
AX-109891069	A/A	A/T	T/T	Affx-110388124	3A	737971959
AX-109892060	A/A	C/C	A/A	Affx-111401626	3A	620205831
AX-109892978	G/G	A/G	A/A	Affx-111473880	3A	683258402
AX-109900697	T/T	T/G	T/T	Affx-88704251	3A	725732230
AX-109901092	G/G	C/G	G/G	Affx-109450722	3A	549622671
AX-109901267	G/G	T/G	G/G	Affx-111101633	3A	664098325
AX-109901713	C/C	T/C	C/C	Affx-108978481	3A	101904483
AX-109904053	T/T	T/C	T/T	Affx-111063732	3A	712448164
AX-109905422	A/A	G/G	A/A	Affx-88585993	3A	142898890
AX-109907412	T/T	T/C	T/T	Affx-111587440	3A	743704414
AX-109908411	T/T	T/C	T/T	Affx-88776895	3A	215346038
AX-109910042	A/A	A/G	A/A	Affx-109911653	3A	666010449
AX-109915534	A/A	C/C	A/A	Affx-110425849	3A	164936444
AX-109915920	T/T	T/C	T/T	Affx-110085910	3A	287684810
AX-109923607	A/A	A/G	G/G	Affx-110762674	3A	518605849
AX-109924523	T/T	C/C	T/T	Affx-109307736	3A	324676068
AX-109924761	T/T	T/G	T/T	Affx-109732273	3A	246568262
AX-109927445	G/G	G/G	G/G	Affx-110033235	3A	22574739
AX-109932282	C/C	C/G	C/C	Affx-109966002	3A	700442428
AX-109936929	G/G	A/G	G/G	Affx-109344667	3A	331023200
AX-109958475	G/G	G/G	G/G	Affx-110474247	3A	478168058
AX-109958833	T/T	T/C	T/T	Affx-110967495	3A	360871003
AX-109963078	A/A	G/G	A/A	Affx-110390240	3A	353962405
AX-109964112	T/T	T/C	C/C	Affx-109441029	3A	744740924
AX-109972641	C/C	C/C	C/C	Affx-109957174	3A	438639490
AX-109973125	G/G	G/G	G/G	Affx-109215341	3A	478680500
AX-109974155	T/T	T/C	C/C	Affx-109994404	3A	746092746
AX-109975400	T/T	T/C	T/T	Affx-109193574	3A	695775395
AX-109976177	A/A	A/A	A/A	Affx-110311905	3A	425906269
AX-109983808	T/T	T/G	T/T	Affx-109656909	3A	442755348
AX-109984137	A/A	A/G	A/A	Affx-111305428	3A	633772383
AX-109989137	T/T	T/C	T/T	Affx-111113859	3A	544035190
AX-109991064	A/A	A/G	A/A	Affx-109586737	3A	239681127
AX-109994397	C/C	C/G	C/C	Affx-111106609	3A	574226760
AX-109995109	T/C	C/C	T/C	Affx-109856284	3A	1291040
AX-109996983	G/G	A/G	G/G	Affx-111518224	3A	726992644
AX-110003002	C/C	T/C	C/C	Affx-88508743	3A	551689452

AX-110005443	G/G	C/G	G/G	Affx-109585208	3A	530379211
AX-110015690	C/C	T/C	C/C	Affx-110340257	3A	358908977
AX-110021843	C/C	A/C	C/C	Affx-109783319	3A	738856482
AX-110025042	A/A	A/G	A/A	Affx-109821377	3A	466572697
AX-110041975	T/T	T/C	T/T	Affx-111997061	3A	374969741
AX-110044435	C/C	C/C	C/C	Affx-109248321	3A	697378386
AX-110049334	G/G	A/G	G/G	Affx-111063276	3A	58495973
AX-110067819	T/T	T/C	T/T	Affx-109067420	3A	243560929
AX-110090221	A/A	A/T	T/T	Affx-88695733	3A	584280144
AX-110130005	T/T	T/C	T/T	Affx-88376023	3A	334758162
AX-110145801	A/A	A/G	G/G	Affx-111803842	3A	524646632
AX-110151010	G/G	A/G	G/G	Affx-111668001	3A	627680947
AX-110153926	A/A	A/G	A/A	Affx-109767678	3A	544508470
AX-110184263	G/G	A/G	A/A	Affx-109484529	3A	517488377
AX-110192525	C/C	T/C	C/C	Affx-109144185	3A	511277990
AX-110364070	T/T	T/C	T/T	Affx-109738622	3A	272860831
AX-110365258	C/C	T/C	T/T	Affx-109731116	3A	540048652
AX-110370953	C/C	T/C	C/C	Affx-109382511	3A	42382536
AX-110372846	A/A	A/C	A/A	Affx-109759341	3A	206274603
AX-110375431	A/A	A/G	A/A	Affx-111116693	3A	531624704
AX-110378026	A/A	A/G	A/A	Affx-110754803	3A	480967259
AX-110378663	G/G	A/G	G/G	Affx-109951016	3A	740890868
AX-110384514	A/A	A/G	G/G	Affx-109964883	3A	523903871
AX-110392318	A/G	A/A	A/A	Affx-109280182	3A	505028713
AX-110401758	C/C	T/C	T/T	Affx-111403335	3A	75417425
AX-110405435	G/G	G/G	A/A	Affx-110654974	3A	540415945
AX-110406616	T/T	T/C	C/C	Affx-111857541	3A	579373891
AX-110407102	T/T	C/C	T/T	Affx-111250833	3A	45586555
AX-110417260	A/A	G/G	A/A	Affx-109808514	3A	566328400
AX-110422872	A/A	A/G	A/A	Affx-88496809	3A	56395321
AX-110423015	A/A	A/C	A/A	Affx-111370582	3A	498347343
AX-110426891	T/T	T/C	T/T	Affx-109873316	3A	245799037
AX-110434299	C/C	C/G	G/G	Affx-109667047	3A	486753510
AX-110439700	G/G	A/G	A/A	Affx-110400790	3A	580188154
AX-110441365	T/T	C/C	T/T	Affx-110062835	3A	397153501
AX-110445755	C/C	T/C	C/C	Affx-111522975	3A	141388051
AX-110448936	T/T	T/C	C/C	Affx-109180418	3A	535992220
AX-110449221	A/G	G/G	A/A	Affx-88532211	3A	68290954
AX-110464726	G/G	A/G	A/A	Affx-109800885	3A	688589316
AX-110470853	T/C	T/T	T/C	Affx-110461286	3A	7448422
AX-110471403	A/A	A/G	A/A	Affx-109342412	3A	137407658
AX-110473707	A/A	A/C	C/C	Affx-110738792	3A	502449420
AX-110485390	T/T	T/C	T/T	Affx-109592187	3A	389557415
AX-110486710	T/T	T/C	T/T	Affx-109905015	3A	287570769
AX-110488116	A/A	A/G	A/A	Affx-109715723	3A	729157170
AX-110489629	C/C	T/C	C/C	Affx-111809383	3A	144293004
AX-110491100	A/A	A/A	G/G	Affx-110532243	3A	705251779
AX-110496623	G/G	C/G	G/G	Affx-109339467	3A	697019421
AX-110502824	T/T	T/C	T/T	Affx-110759552	3A	313026610
AX-110505846	G/G	A/G	G/G	Affx-88471088	3A	731225339
AX-110508416	G/G	G/G	G/G	Affx-109016615	3A	600748097
AX-110512340	T/T	T/C	T/T	Affx-111667688	3A	372883144
AX-110512746	T/T	T/C	T/T	Affx-110668588	3A	531915987
AX-110518818	T/T	T/C	T/T	Affx-109772143	3A	649438322

AX-110526674	A/A	A/G	A/A	Affx-88755071	3A	716098200
AX-110529370	T/T	T/C	C/C	Affx-111667228	3A	522897991
AX-110531751	G/G	T/G	G/G	Affx-110021698	3A	664881698
AX-110533572	C/C	T/C	T/T	Affx-111715476	3A	575067560
AX-110534147	G/G	A/G	A/A	Affx-110095238	3A	747608264
AX-110539312	A/A	A/G	A/A	Affx-109166445	3A	421736363
AX-110545382	C/C	T/C	C/C	Affx-111725228	3A	716670776
AX-110552246	A/A	A/C	A/A	Affx-109713705	3A	740058361
AX-110567176	T/T	T/C	T/T	Affx-109372844	3A	356700734
AX-110575119	G/G	A/G	G/G	Affx-109859066	3A	678504151
AX-110578705	C/C	T/C	T/T	Affx-110790000	3A	500678257
AX-110578732	C/C	T/C	C/C	Affx-109725190	3A	233195363
AX-110578860	A/A	A/G	A/A	Affx-110541170	3A	507297890
AX-110583106	A/G	G/G	A/G	Affx-109244462	3A	225088162
AX-110591324	G/G	A/G	G/G	Affx-110897051	3A	530933571
AX-110600168	G/G	T/G	G/G	Affx-111212924	3A	132769679
AX-110602564	T/T	T/C	C/C	Affx-110616161	3A	538596696
AX-110603951	G/G	T/G	T/T	Affx-110894997	3A	502968574
AX-110606671	A/A	A/G	A/A	Affx-109019371	3A	626895350
AX-110611074	C/C	T/C	C/C	Affx-110210926	3A	143439600
AX-110617585	G/G	A/G	A/A	Affx-109027906	3A	649032514
AX-110631819	G/G	A/G	G/G	Affx-88736960	3A	482062594
AX-110636649	C/C	T/C	C/C	Affx-88745191	3A	14848821
AX-110667231	T/T	T/T	T/T	Affx-108903979	3A	554331876
AX-110667599	C/C	C/C	C/C	Affx-110653364	3A	580598934
AX-110669775	A/A	A/G	A/A	Affx-111819388	3A	667086037
AX-110670767	T/C	T/C	T/T	Affx-109844453	3A	79296520
AX-110687537	T/T	T/C	T/T	Affx-111887922	3A	13735015
AX-110688259	A/A	A/A	A/A	Affx-110807793	3A	209823664
AX-110707258	C/C	T/T	C/C	Affx-110019024	3A	733432801
AX-110735133	C/C	T/C	C/C	Affx-109482666	3A	193972914
AX-110736133	C/C	T/T	C/C	Affx-109318836	3A	207246691
AX-110904521	G/G	A/G	G/G	Affx-111566920	3A	515286376
AX-110906848	A/A	A/G	A/A	Affx-111920276	3A	741672899
AX-110908981	G/G	C/G	G/G	Affx-111185944	3A	304464818
AX-110913950	C/C	A/C	C/C	Affx-111074085	3A	24063664
AX-110915520	T/T	G/G	T/T	Affx-111822344	3A	261929425
AX-110916132	C/C	A/C	C/C	Affx-109801360	3A	67388570
AX-110917484	T/T	T/C	C/C	Affx-111595619	3A	512328586
AX-110919428	C/C	T/C	C/C	Affx-110989714	3A	565792184
AX-110919865	C/C	A/C	C/C	Affx-111460756	3A	501652399
AX-110924505	A/A	A/G	A/A	Affx-111789209	3A	294937693
AX-110937777	C/C	T/C	C/C	Affx-111191670	3A	510767874
AX-110941450	A/A	A/C	A/A	Affx-109563871	3A	370670763
AX-110948185	A/A	A/T	A/A	Affx-111933471	3A	272541098
AX-110951668	G/G	G/G	A/A	Affx-108976054	3A	595736993
AX-110954847	A/A	A/G	A/A	Affx-110548250	3A	132146770
AX-110956298	C/C	C/C	C/C	Affx-110909183	3A	526512160
AX-110962843	G/G	A/G	G/G	Affx-110364947	3A	636807602
AX-110963001	T/T	C/C	T/C	Affx-88526177	3A	510597814
AX-110965999	A/A	G/G	A/A	Affx-109158434	3A	260495992
AX-110967458	A/A	A/G	A/A	Affx-88711835	3A	23642562
AX-110972761	C/C	C/G	C/C	Affx-88369957	3A	743186032
AX-110982768	C/C	T/C	C/C	Affx-110984942	3A	720706536

AX-111008632	G/G	T/G	G/G	Affx-109163101	3A	476985471
AX-111024665	T/T	T/C	T/T	Affx-109901916	3A	471289954
AX-111024746	A/A	A/C	A/A	Affx-88530805	3A	557414688
AX-111033605	T/T	T/C	C/C	Affx-110322309	3A	506442311
AX-111044516	A/A	A/A	G/G	Affx-109939689	3A	659803155
AX-111047293	T/T	T/C	T/T	Affx-111145148	3A	264265589
AX-111048385	G/G	G/G	G/G	Affx-111626172	3A	719168001
AX-111051495	T/T	T/C	T/T	Affx-108874600	3A	100192924
AX-111053963	A/A	A/G	A/A	Affx-109071927	3A	255064405
AX-111054546	G/G	A/G	G/G	Affx-109007826	3A	453007021
AX-111055491	G/G	A/G	G/G	Affx-111734675	3A	652020267
AX-111060245	A/A	A/C	A/A	Affx-110008799	3A	301115820
AX-111065136	A/A	A/G	A/A	Affx-110874004	3A	278367815
AX-111067313	G/G	A/G	G/G	Affx-88597986	3A	31949850
AX-111078409	G/G	A/G	A/A	Affx-109286934	3A	583347793
AX-111079049	C/C	T/C	C/C	Affx-111871460	3A	685708135
AX-111087059	A/A	A/G	A/A	Affx-110525709	3A	387767367
AX-111088663	C/C	T/C	C/C	Affx-110582587	3A	59560091
AX-111091518	T/T	T/C	T/T	Affx-109069431	3A	171778823
AX-111096752	T/T	T/G	T/T	Affx-109700001	3A	219758396
AX-111100488	T/T	T/G	G/G	Affx-110148485	3A	524896092
AX-111106126	A/A	A/G	A/A	Affx-108929389	3A	707835332
AX-111110553	G/G	A/G	G/G	Affx-109809921	3A	250912640
AX-111116170	A/A	A/G	A/A	Affx-88788969	3A	669624832
AX-111129267	C/C	T/C	T/T	Affx-110521770	3A	481678454
AX-111138337	A/G	A/G	A/G	Affx-110823699	3A	19999667
AX-111142459	G/G	A/G	G/G	Affx-109270084	3A	57730709
AX-111147819	T/T	T/C	T/T	Affx-110833270	3A	542635181
AX-111153043	G/G	A/G	G/G	Affx-111371155	3A	562360021
AX-111157535	A/A	A/A	A/A	Affx-108983646	3A	246411867
AX-111161056	C/C	T/C	T/T	Affx-88480537	3A	684869105
AX-111168955	T/T	C/C	T/T	Affx-109705103	3A	168834162
AX-111177475	T/T	T/C	T/T	Affx-88724836	3A	127978380
AX-111214616	C/C	T/C	C/C	Affx-88707178	3A	239360099
AX-111215306	T/T	T/G	T/T	Affx-110247808	3A	196496804
AX-111223386	A/A	G/G	A/A	Affx-111480522	3A	737114806
AX-111227798	T/T	T/C	T/T	Affx-110708949	3A	489656413
AX-111230990	A/A	A/C	A/A	Affx-111774219	3A	141970789
AX-111231934	A/A	A/T	A/A	Affx-111449054	3A	418013879
AX-111233264	T/T	T/G	T/T	Affx-109391760	3A	516556597
AX-111235003	C/C	T/C	C/C	Affx-109061588	3A	300271350
AX-111256427	T/T	T/T	T/T	Affx-110374666	3A	270958055
AX-111258786	T/T	T/C	T/C	Affx-109147870	3A	568669609
AX-111277545	T/T	T/G	T/T	Affx-109333723	3A	616717574
AX-111450442	C/G	C/G	C/C	Affx-110960309	3A	724018150
AX-111450751	C/C	T/C	C/C	Affx-88718429	3A	70703503
AX-111454706	A/A	A/G	A/A	Affx-110144029	3A	468111360
AX-111456639	T/T	T/C	T/T	Affx-109995631	3A	425822778
AX-111457875	A/A	A/G	A/A	Affx-88654774	3A	296113337
AX-111458518	A/A	A/G	A/A	Affx-109668541	3A	571922769
AX-111460264	G/G	A/G	A/A	Affx-88428955	3A	537515609
AX-111462081	A/A	A/C	A/A	Affx-110439264	3A	625409071
AX-111466140	A/A	A/C	C/C	Affx-110820398	3A	534852566
AX-111472865	G/G	A/G	G/G	Affx-111125679	3A	12785295

AX-111475163	G/G	A/G	G/G	Affx-88499896	3A	543020870
AX-111478032	G/G	G/G	G/G	Affx-109955409	3A	29049045
AX-111481495	G/G	A/G	G/G	Affx-111422866	3A	692969135
AX-111482593	C/C	T/C	C/C	Affx-111207102	3A	473455087
AX-111485003	T/T	T/G	T/T	Affx-110757491	3A	330279116
AX-111487970	C/C	T/C	T/T	Affx-109416968	3A	504553590
AX-111490400	T/T	T/C	T/T	Affx-111734273	3A	547993061
AX-111504992	G/G	C/G	G/G	Affx-92588693	3A	715144266
AX-111505762	C/C	T/C	C/C	Affx-88419933	3A	175848032
AX-111506211	C/C	T/C	T/T	Affx-88725497	3A	662510241
AX-111513334	T/T	A/T	T/T	Affx-108867251	3A	487368256
AX-111514932	T/T	T/C	T/T	Affx-109095878	3A	49469642
AX-111515732	G/G	G/G	G/G	Affx-110179934	3A	671651234
AX-111520556	A/A	A/G	A/A	Affx-110632616	3A	285931698
AX-111521043	G/G	A/A	G/G	Affx-111951095	3A	622904985
AX-111522081	C/C	T/C	T/T	Affx-111464784	3A	496600883
AX-111524586	G/G	A/G	A/A	Affx-110312469	3A	73631815
AX-111528638	G/G	T/G	G/G	Affx-88470602	3A	667950773
AX-111543244	A/A	A/A	A/A	Affx-110023718	3A	303256707
AX-111543258	A/A	A/G	A/A	Affx-111740231	3A	27884426
AX-111551307	C/C	T/C	C/C	Affx-109519177	3A	217838315
AX-111551830	A/A	A/G	G/G	Affx-88452177	3A	513431220
AX-111552673	G/G	A/G	G/G	Affx-110660187	3A	55525014
AX-111555163	T/T	T/C	T/T	Affx-110533723	3A	339665587
AX-111556286	A/A	A/C	A/A	Affx-111587911	3A	619103441
AX-111559124	A/A	A/G	A/A	Affx-109200205	3A	436276990
AX-111560904	G/G	C/G	C/C	Affx-110329201	3A	686861658
AX-111564924	T/T	T/C	T/T	Affx-111834117	3A	203295625
AX-111571396	T/T	T/C	T/T	Affx-111225937	3A	359441868
AX-111572609	T/T	T/C	T/T	Affx-110334948	3A	710297516
AX-111582765	G/G	A/G	G/G	Affx-109936559	3A	1888764
AX-111583763	A/A	A/C	C/C	Affx-109511648	3A	675963357
AX-111588609	C/C	T/C	C/C	Affx-110451261	3A	9061977
AX-111590533	C/C	T/C	C/C	Affx-111685992	3A	552470059
AX-111591393	A/A	G/G	A/A	Affx-109404562	3A	564453709
AX-111591696	G/G	A/G	A/A	Affx-108870250	3A	89970813
AX-111602433	G/G	A/G	G/G	Affx-111313034	3A	603932976
AX-111609470	T/C	C/C	T/C	Affx-110733699	3A	355772705
AX-111611367	G/G	A/G	G/G	Affx-88399558	3A	36657660
AX-111612053	C/C	T/C	C/C	Affx-110524472	3A	104135679
AX-111616392	A/A	A/C	A/A	Affx-109267102	3A	693181635
AX-111618763	T/T	T/C	C/C	Affx-109530742	3A	599422133
AX-111627621	G/G	C/G	C/C	Affx-111848978	3A	483182120
AX-111628960	T/T	T/C	C/C	Affx-111772921	3A	652909942
AX-111631905	G/G	C/G	G/G	Affx-110412256	3A	46005047
AX-111634399	G/G	G/G	G/G	Affx-109350410	3A	589373358
AX-111643946	G/G	C/G	C/C	Affx-109965857	3A	646585307
AX-111644842	G/G	A/G	G/G	Affx-109485396	3A	550045144
AX-111651061	C/C	T/C	C/C	Affx-110566248	3A	620625489
AX-111653361	T/T	T/C	T/T	Affx-109028974	3A	35648919
AX-111653822	A/G	A/G	G/G	Affx-88346664	3A	539167083
AX-111656976	A/A	A/A	A/A	Affx-110749654	3A	728515342
AX-111679785	C/C	T/C	C/C	Affx-111600148	3A	393491130
AX-111680640	A/A	A/G	A/A	Affx-110334694	3A	33704927

AX-111680877	G/G	A/G	G/G	Affx-111320020	3A	92946550
AX-111688182	C/C	C/C	C/C	Affx-111549869	3A	71126489
AX-111692048	G/G	A/G	G/G	Affx-111772899	3A	574475183
AX-111693127	C/C	C/C	C/C	Affx-109097045	3A	592932640
AX-111703137	T/T	T/C	T/T	Affx-111911942	3A	529139968
AX-111706579	C/C	C/C	C/C	Affx-110432495	3A	602858570
AX-111708993	A/G	G/G	A/G	Affx-109684401	3A	272796105
AX-111710665	C/C	T/C	C/C	Affx-88722273	3A	697591212
AX-111712891	G/G	A/G	G/G	Affx-88640703	3A	58931009
AX-111756816	T/T	T/C	T/T	Affx-111856671	3A	26230125
AX-111763200	T/T	T/C	T/T	Affx-109664828	3A	632744119
AX-111776997	G/G	T/G	G/G	Affx-109293871	3A	714533041
AX-111777043	A/A	A/G	A/A	Affx-109061423	3A	378356920
AX-111778608	C/C	T/C	C/C	Affx-111483649	3A	234721794
AX-111786035	C/C	C/G	C/C	Affx-110240505	3A	498624716
AX-111801077	T/T	T/C	T/T	Affx-109563666	3A	486243976
AX-111802923	A/A	A/A	A/A	Affx-110095030	3A	17066992
AX-111802937	A/A	A/G	A/A	Affx-109663753	3A	183001
AX-111811874	C/C	T/C	T/T	Affx-109894927	3A	568838274
AX-111826360	G/G	G/G	G/G	Affx-111991311	3A	618761167
AX-179559318	T/T	T/C	T/T	Affx-88649128	3A	53569343
AX-179561097	A/A	A/A	A/A	Affx-88347021	3A	9855414
AX-86165946	G/G	A/G	A/A	Affx-110782052	3A	660409864
AX-86165976	G/G	A/G	A/A	Affx-93016228	3A	685290137
AX-86179914	C/C	C/C	C/C	Affx-92453346	3A	14045695
AX-89381998	T/T	T/C	T/T	Affx-88414397	3A	637901078
AX-89400276	A/A	A/A	A/A	Affx-92462338	3A	704584881
AX-89436449	T/T	T/G	G/G	Affx-88469160	3A	586881536
AX-89471844	G/G	T/G	G/G	Affx-111350497	3A	608339919
AX-89497626	C/C	T/C	T/T	Affx-109271250	3A	95137255
AX-89532923	G/G	A/G	G/G	Affx-88565881	3A	717623709
AX-89537027	T/T	T/C	T/T	Affx-88570001	3A	655630478
AX-89567713	T/T	C/C	T/T	Affx-88600777	3A	724870059
AX-89583101	C/C	C/G	C/C	Affx-88616209	3A	29031077
AX-89596424	C/C	C/C	C/C	Affx-88629572	3A	513927888
AX-89644172	G/G	G/G	G/G	Affx-88676968	3A	722348715
AX-89650480	G/G	G/G	G/G	Affx-88683277	3A	254359818
AX-89681107	T/T	T/C	T/T	Affx-88713871	3A	114422824
AX-89718722	A/T	A/T	T/T	Affx-88751426	3A	739532725
AX-94417941	C/C	T/C	C/C	Affx-92671650	3A	741240716
AX-94434389	C/C	T/C	C/C	Affx-92293746	3A	41540618
AX-94457106	A/G	A/G	A/G	Affx-92159356	3A	7474715
AX-94470714	T/T	T/C	T/T	Affx-92676151	3A	53204808
AX-94522426	C/C	T/T	C/C	Affx-92147259	3A	13405119
AX-94541365	G/G	C/G	G/G	Affx-92689265	3A	369405010
AX-94558225	T/T	T/T	T/T	Affx-92740106	3A	32415515
AX-94564058	C/C	A/C	A/A	Affx-92751635	3A	598563592
AX-94571147	G/G	A/G	A/A	Affx-92206064	3A	687527098
AX-94607247	A/G	G/G	A/G	Affx-92822061	3A	49480504
AX-94684776	C/C	T/C	C/C	Affx-92279064	3A	674753256
AX-94707079	G/G	A/G	G/G	Affx-92326701	3A	466775222
AX-94732123	A/A	A/G	A/A	Affx-92957655	3A	32252220
AX-94793174	A/A	A/G	A/A	Affx-92712875	3A	749346577
AX-94920311	G/G	C/G	G/G	Affx-92818240	3A	719764197

AX-94996868	C/C	C/C	C/C	Affx-92442257	3A	1030541
AX-95072891	C/C	C/C	C/C	Affx-92290640	3A	690432617
AX-95081885	C/C	T/C	T/T	Affx-92507230	3A	646737497
AX-95168510	T/G	T/G	T/G	Affx-92847974	3A	661474496
AX-95207768	G/G	G/G	G/G	Affx-92446131	3A	656104843
AX-95210076	C/C	T/C	T/T	Affx-92829565	3A	699844037
AX-95245536	G/G	A/G	G/G	Affx-92132605	3A	680749657
AX-95631471	A/A	A/A	A/A	Affx-88677523	3A	44629466
AX-95632304	C/C	T/C	C/C	Affx-88601389	3A	41068049
AX-95651716	G/G	A/G	G/G	Affx-110753119	3A	611948316
AX-95653997	A/C	C/C	A/C	Affx-88423600	3A	714300447
AX-95659229	C/C	T/C	C/C	Affx-88620274	3A	724319545
AX-108725311	G/G	A/G	G/G	Affx-110325588	3B	448360950
AX-108726233	A/A	A/G	A/A	Affx-111639586	3B	147811615
AX-108726753	A/A	A/G	A/A	Affx-109267947	3B	471803070
AX-108726773	C/C	C/C	C/C	Affx-110979475	3B	54537197
AX-108729416	A/A	A/G	A/A	Affx-109532580	3B	572682335
AX-108730093	C/C	T/C	C/C	Affx-88493338	3B	415055375
AX-108733780	G/G	T/G	G/G	Affx-109981670	3B	556044152
AX-108734214	G/G	G/G	G/G	Affx-111057274	3B	237834184
AX-108734897	T/T	T/C	T/T	Affx-109219015	3B	598125119
AX-108738400	C/C	A/C	C/C	Affx-110526174	3B	714114257
AX-108738488	G/G	A/G	G/G	Affx-111582192	3B	701436510
AX-108745377	C/C	C/C	C/C	Affx-110266040	3B	742860094
AX-108746674	G/G	T/G	G/G	Affx-108981334	3B	700425037
AX-108746747	A/A	A/C	A/A	Affx-109920195	3B	133188397
AX-108748429	A/A	A/C	A/A	Affx-111419352	3B	257583571
AX-108750946	G/G	T/G	G/G	Affx-111304625	3B	70866497
AX-108758435	A/A	A/G	A/A	Affx-110883562	3B	752640736
AX-108758512	C/C	T/C	C/C	Affx-110560071	3B	565854417
AX-108759829	G/G	A/G	G/G	Affx-109777540	3B	780263451
AX-108760591	T/T	T/T	T/T	Affx-111107623	3B	750468624
AX-108763755	C/C	T/C	C/C	Affx-110512964	3B	187522668
AX-108765398	A/A	C/C	A/A	Affx-111226814	3B	342112154
AX-108769641	A/A	G/G	A/A	Affx-111566481	3B	604365827
AX-108772830	T/T	T/C	T/T	Affx-110641319	3B	554646783
AX-108773562	C/C	C/G	C/C	Affx-110218003	3B	33595676
AX-108774865	A/G	A/G	A/G	Affx-109986088	3B	391632477
AX-108777806	C/C	C/G	C/C	Affx-110108617	3B	549337723
AX-108780104	T/T	T/G	T/T	Affx-110621751	3B	312198054
AX-108783141	T/T	T/C	T/T	Affx-111696200	3B	452091179
AX-108784392	T/T	T/C	T/T	Affx-88806417	3B	507497340
AX-108785727	G/G	C/G	G/G	Affx-110017457	3B	274865116
AX-108789522	A/A	A/G	A/A	Affx-111503569	3B	205142563
AX-108790822	C/C	C/C	C/C	Affx-110318281	3B	141628197
AX-108791887	A/A	A/G	A/A	Affx-109040996	3B	430521720
AX-108793109	T/T	T/C	T/T	Affx-110353427	3B	336834198
AX-108796653	G/G	G/G	G/G	Affx-111540322	3B	206627475
AX-108796704	C/C	C/G	C/C	Affx-88664630	3B	715181513
AX-108802911	A/A	A/G	A/A	Affx-109457841	3B	590351349
AX-108807333	C/C	C/C	C/C	Affx-111962446	3B	68297981
AX-108808626	C/C	T/C	C/C	Affx-111178669	3B	42490761
AX-108827917	C/C	T/C	C/C	Affx-109176176	3B	802133
AX-108829779	A/A	A/G	A/A	Affx-110488538	3B	413226860

AX-108833535	A/A	A/G	A/A	Affx-110263530	3B	312483736
AX-108836843	G/G	T/G	T/T	Affx-111570212	3B	8688389
AX-108838049	A/A	A/G	A/A	Affx-109859427	3B	788107453
AX-108842651	T/T	T/C	T/T	Affx-109201685	3B	505615705
AX-108843975	A/A	A/G	A/A	Affx-110290141	3B	723025109
AX-108847061	G/G	A/G	G/G	Affx-111483544	3B	397555911
AX-108847395	G/G	C/G	C/C	Affx-109970392	3B	156722691
AX-108858773	A/A	A/G	A/A	Affx-109062747	3B	39147989
AX-108859880	C/C	C/C	C/C	Affx-109505633	3B	758066501
AX-108863110	A/A	A/A	A/A	Affx-109626893	3B	21589710
AX-108864275	C/C	T/C	C/C	Affx-110833596	3B	63663790
AX-108866480	A/A	A/A	A/A	Affx-110180378	3B	64597399
AX-108877144	C/C	T/T	C/C	Affx-109441270	3B	716047318
AX-108878935	G/G	C/G	G/G	Affx-111514139	3B	76427597
AX-108881120	C/C	T/C	C/C	Affx-109346123	3B	656576315
AX-108884085	G/G	A/G	G/G	Affx-108938371	3B	689177086
AX-108888746	A/A	A/A	A/A	Affx-111511955	3B	342872276
AX-108890155	T/T	T/C	T/T	Affx-110408103	3B	427596739
AX-108901344	C/C	C/C	C/C	Affx-88557493	3B	146828510
AX-108901896	T/T	T/C	T/T	Affx-109518382	3B	278773672
AX-108903792	A/A	A/G	G/G	Affx-110324584	3B	171796382
AX-108906727	A/A	A/G	A/A	Affx-110866307	3B	22666096
AX-108912148	T/T	T/C	T/T	Affx-109503910	3B	208366330
AX-108914590	A/A	A/A	A/A	Affx-111357520	3B	696355856
AX-108915318	A/A	A/A	A/A	Affx-110843931	3B	437483153
AX-108920444	G/G	A/G	G/G	Affx-110322534	3B	588992083
AX-108923034	T/T	T/C	T/T	Affx-109982931	3B	809831411
AX-108925120	G/G	C/G	G/G	Affx-111804255	3B	807126866
AX-108929188	A/G	A/G	A/G	Affx-110023588	3B	583729208
AX-108936122	A/A	A/G	A/A	Affx-88785116	3B	119465526
AX-108936649	C/C	T/C	C/C	Affx-108879446	3B	275948730
AX-108938781	A/A	A/G	A/A	Affx-109741070	3B	240957047
AX-108939054	C/C	T/T	C/C	Affx-111697092	3B	693333994
AX-108940705	G/G	A/G	G/G	Affx-111865346	3B	544554612
AX-108942819	C/C	C/C	C/C	Affx-110682551	3B	189919284
AX-108949111	C/C	A/A	C/C	Affx-112002655	3B	564780210
AX-108951729	A/A	A/G	A/A	Affx-109539089	3B	353279751
AX-108952050	T/T	T/C	T/T	Affx-109606125	3B	636115233
AX-108956706	C/C	A/C	C/C	Affx-111283045	3B	657951050
AX-108959409	C/C	T/C	C/C	Affx-110989501	3B	267311087
AX-108963257	G/G	T/G	G/G	Affx-109195270	3B	338747455
AX-108968612	C/C	T/C	C/C	Affx-110318519	3B	81299474
AX-108968961	T/T	T/C	T/C	Affx-109631627	3B	399747428
AX-108972858	G/G	G/G	G/G	Affx-111146723	3B	494132452
AX-108973826	C/C	C/C	C/C	Affx-111140563	3B	777354696
AX-108975699	A/A	A/G	A/A	Affx-111506108	3B	432339464
AX-108977600	T/T	T/C	T/T	Affx-108959068	3B	411983223
AX-108978268	A/A	A/A	A/A	Affx-111198706	3B	310826930
AX-108980315	G/G	C/G	G/G	Affx-88468070	3B	750138437
AX-108981804	G/G	T/T	G/G	Affx-109341921	3B	224321666
AX-108982782	C/C	C/C	C/C	Affx-109693630	3B	139831353
AX-108985787	C/C	T/C	C/C	Affx-109058761	3B	481607098
AX-108992105	T/T	T/C	T/T	Affx-110591482	3B	452755356
AX-108993437	C/C	T/C	C/C	Affx-109247727	3B	417711437

AX-108998031	A/A	A/G	A/A	Affx-111017562	3B	736420673
AX-109007866	A/A	A/A	A/A	Affx-111286162	3B	12103184
AX-109011985	T/C	T/T	T/C	Affx-110400313	3B	356866416
AX-109026323	C/C	T/C	C/C	Affx-110009940	3B	766641736
AX-109032259	G/G	A/G	G/G	Affx-111093356	3B	43318741
AX-109032419	C/C	T/C	C/C	Affx-110433627	3B	817963420
AX-109033816	A/A	A/C	A/A	Affx-109849942	3B	668891822
AX-109033951	C/C	T/C	C/C	Affx-111606099	3B	519135463
AX-109035695	T/T	T/C	T/T	Affx-111159941	3B	463010669
AX-109037794	G/G	C/G	G/G	Affx-109142925	3B	488315553
AX-109037819	C/G	C/G	C/G	Affx-109188135	3B	294513370
AX-109040399	T/T	T/C	T/T	Affx-110886835	3B	47474068
AX-109050064	T/T	T/G	T/T	Affx-111856109	3B	41260367
AX-109052060	A/G	G/G	G/G	Affx-109103612	3B	15126449
AX-109052320	A/A	A/G	A/A	Affx-110164543	3B	754617441
AX-109052327	C/C	C/G	C/C	Affx-109832005	3B	274039162
AX-109067519	G/G	T/G	G/G	Affx-110478518	3B	348164798
AX-109074160	C/C	T/C	C/C	Affx-111791994	3B	222179110
AX-109074171	G/G	T/G	G/G	Affx-109345563	3B	303945428
AX-109077284	G/G	A/G	G/G	Affx-111365724	3B	580888626
AX-109095345	T/T	T/T	T/T	Affx-110490010	3B	341868232
AX-109101572	T/T	C/C	T/C	Affx-88712393	3B	280826233
AX-109103577	G/G	A/G	G/G	Affx-110696622	3B	391262703
AX-109107259	G/G	A/G	G/G	Affx-111112832	3B	461653754
AX-109107783	C/C	T/C	T/T	Affx-108885125	3B	167636481
AX-109273424	G/G	A/G	G/G	Affx-110309676	3B	354449283
AX-109277293	G/G	A/G	A/A	Affx-109976487	3B	761958862
AX-109279813	T/T	T/G	T/T	Affx-110420773	3B	826222477
AX-109282231	G/G	C/G	G/G	Affx-110602837	3B	138504201
AX-109282650	C/C	C/C	C/C	Affx-110181086	3B	703120585
AX-109282755	T/T	T/C	T/T	Affx-88444455	3B	412226008
AX-109283006	T/T	T/C	T/T	Affx-111660370	3B	404628122
AX-109283973	C/C	C/G	C/C	Affx-109325415	3B	749556778
AX-109285798	C/C	C/G	C/C	Affx-109166510	3B	744425981
AX-109287073	T/T	T/C	C/C	Affx-110069630	3B	169132680
AX-109290113	A/A	A/G	A/A	Affx-109573629	3B	74314714
AX-109291094	C/C	C/C	C/C	Affx-110255325	3B	434952247
AX-109291541	C/C	T/C	C/C	Affx-111957110	3B	692258285
AX-109291778	A/A	A/G	A/A	Affx-111592437	3B	204280701
AX-109292076	C/C	C/C	C/C	Affx-110569929	3B	573305711
AX-109296588	G/G	A/G	A/G	Affx-109695785	3B	325008942
AX-109298417	A/A	A/C	C/C	Affx-109012064	3B	164950977
AX-109298987	A/G	G/G	A/G	Affx-111764871	3B	374530740
AX-109300864	T/T	C/C	T/T	Affx-111780071	3B	724520788
AX-109301883	A/A	A/G	A/A	Affx-109157908	3B	13926060
AX-109303193	C/C	T/C	C/C	Affx-110499695	3B	143573669
AX-109304063	A/A	A/G	A/A	Affx-111276766	3B	259661638
AX-109309332	A/A	A/G	A/A	Affx-109904776	3B	820059298
AX-109311204	C/C	C/C	C/C	Affx-109854851	3B	2281393
AX-109312574	C/C	A/C	C/C	Affx-110454565	3B	829561349
AX-109312590	C/C	T/C	C/C	Affx-111129672	3B	510650172
AX-109314845	A/A	A/G	A/A	Affx-110495730	3B	705239568
AX-109315356	C/C	T/C	C/C	Affx-111721748	3B	418126914
AX-109316106	G/G	C/G	G/G	Affx-111561093	3B	468158443

AX-109316602	T/T	T/T	T/T	Affx-88807087	3B	84882515
AX-109317301	C/C	T/C	C/C	Affx-111319024	3B	227411849
AX-109317912	A/A	A/C	A/A	Affx-109288689	3B	437762394
AX-109324755	A/A	A/G	A/A	Affx-111839124	3B	152277884
AX-109330597	T/T	T/C	T/T	Affx-111586721	3B	188615036
AX-109334330	C/C	T/C	C/C	Affx-110089397	3B	118193311
AX-109338627	C/C	A/C	C/C	Affx-108975196	3B	126845393
AX-109340844	C/C	T/C	C/C	Affx-110837931	3B	204101414
AX-109343990	C/C	T/C	C/C	Affx-111611180	3B	563042838
AX-109344528	T/T	T/C	T/T	Affx-108998470	3B	17996883
AX-109345409	A/A	A/A	A/A	Affx-111101972	3B	745287661
AX-109345791	G/G	A/G	G/G	Affx-109276727	3B	432839506
AX-109348047	G/G	A/G	G/G	Affx-110703138	3B	271817248
AX-109349136	T/T	C/C	T/T	Affx-111088198	3B	693344830
AX-109350957	G/G	A/G	G/G	Affx-108970358	3B	725539969
AX-109352323	C/C	C/G	C/C	Affx-109595180	3B	31815536
AX-109352341	C/C	C/G	C/C	Affx-111729239	3B	719084243
AX-109353400	A/A	A/G	A/A	Affx-111299537	3B	694655086
AX-109353708	C/C	T/C	C/C	Affx-111566696	3B	434048229
AX-109353769	T/C	T/C	T/C	Affx-109303392	3B	254428024
AX-109354553	A/A	A/G	A/A	Affx-110823028	3B	450057707
AX-109355238	C/C	A/C	C/C	Affx-109748135	3B	503528440
AX-109357190	C/C	T/C	C/C	Affx-109845023	3B	82825841
AX-109358405	C/C	C/C	C/C	Affx-109140140	3B	331158286
AX-109359639	C/C	T/C	C/C	Affx-109628995	3B	123714815
AX-109361035	G/G	A/G	G/G	Affx-111697571	3B	358756195
AX-109361439	C/C	T/C	C/C	Affx-109086495	3B	73309971
AX-109362087	G/G	A/G	G/G	Affx-109700679	3B	271637508
AX-109363475	T/T	T/G	G/G	Affx-88409762	3B	169257438
AX-109363741	C/C	T/C	C/C	Affx-111132338	3B	603859767
AX-109363759	C/C	A/C	C/C	Affx-88660599	3B	493120678
AX-109364846	G/G	A/G	G/G	Affx-111500774	3B	417151439
AX-109367315	A/A	A/G	A/A	Affx-109640200	3B	848047
AX-109368009	T/T	T/C	T/T	Affx-110833306	3B	707430572
AX-109368875	T/T	T/C	T/T	Affx-109100967	3B	800662875
AX-109370170	T/T	T/C	T/T	Affx-109497375	3B	449200697
AX-109373871	A/A	A/G	A/A	Affx-110025446	3B	702123161
AX-109382739	G/G	A/G	G/G	Affx-111707168	3B	712556064
AX-109385302	G/G	A/G	A/A	Affx-109132226	3B	159468894
AX-109388420	T/T	T/T	T/T	Affx-111034514	3B	100965565
AX-109390753	T/T	C/C	T/T	Affx-110793570	3B	336614552
AX-109393290	C/C	A/C	C/C	Affx-108911338	3B	737058774
AX-109394116	G/G	G/G	G/G	Affx-112002565	3B	201339460
AX-109394157	A/G	G/G	A/G	Affx-110222016	3B	133803932
AX-109395116	A/A	A/G	A/A	Affx-109844626	3B	128705070
AX-109397005	G/G	A/G	G/G	Affx-111340371	3B	294975151
AX-109399079	C/C	T/C	C/C	Affx-110251196	3B	62976210
AX-109401676	T/T	T/C	T/T	Affx-111795223	3B	796516602
AX-109401831	A/A	A/C	C/C	Affx-110312381	3B	8525559
AX-109403706	C/C	T/C	C/C	Affx-110938267	3B	304686941
AX-109406585	C/C	T/C	C/C	Affx-109853982	3B	519055599
AX-109407787	T/T	G/G	T/T	Affx-109061968	3B	572203246
AX-109417098	C/C	C/C	C/C	Affx-110987773	3B	83743770
AX-109418825	C/C	T/C	C/C	Affx-109675411	3B	423458416

AX-109419419	A/A	A/G	A/A	Affx-109282202	3B	207510339
AX-109419793	A/A	A/C	A/A	Affx-110757905	3B	458393821
AX-109420075	C/C	C/G	C/C	Affx-109306402	3B	142841868
AX-109422148	A/A	A/G	A/A	Affx-110485768	3B	130152525
AX-109425523	A/A	A/G	A/A	Affx-110696185	3B	794151
AX-109425901	C/C	T/C	C/C	Affx-109779729	3B	783892992
AX-109426126	A/A	A/G	A/A	Affx-110013942	3B	218068759
AX-109430773	A/A	G/G	A/A	Affx-109123904	3B	206231842
AX-109431626	G/G	A/G	G/G	Affx-108921023	3B	565428198
AX-109432046	C/C	A/C	C/C	Affx-110055850	3B	225452705
AX-109434649	A/A	A/G	A/A	Affx-109135670	3B	375306110
AX-109436590	G/G	T/G	G/G	Affx-88426311	3B	562896234
AX-109445547	A/A	A/G	A/A	Affx-88521351	3B	75586197
AX-109448454	T/T	T/C	T/T	Affx-109643486	3B	237244358
AX-109448604	T/T	T/C	T/T	Affx-109047651	3B	51160313
AX-109449661	C/C	T/C	C/C	Affx-110970497	3B	659731316
AX-109451814	G/G	A/G	G/G	Affx-109414838	3B	567335280
AX-109456714	C/C	T/C	C/C	Affx-111336775	3B	250243985
AX-109460486	A/A	A/G	A/A	Affx-109465265	3B	209345123
AX-109461374	T/T	T/C	T/T	Affx-109341353	3B	660763814
AX-109462503	T/T	T/C	T/T	Affx-110841538	3B	694262332
AX-109464610	T/T	T/G	T/T	Affx-109583673	3B	320038226
AX-109465849	G/G	A/G	G/G	Affx-111663250	3B	720167764
AX-109471815	C/C	C/C	C/C	Affx-109067728	3B	18859231
AX-109474140	G/G	A/G	G/G	Affx-110888659	3B	614569594
AX-109474531	T/T	T/G	T/T	Affx-109014039	3B	440856985
AX-109474707	A/A	A/C	A/A	Affx-109905898	3B	506745093
AX-109479342	C/C	C/C	C/C	Affx-111767157	3B	182594559
AX-109480581	G/G	A/G	G/G	Affx-88704638	3B	333423971
AX-109485929	A/A	A/C	A/A	Affx-110468948	3B	411504089
AX-109489209	T/T	A/T	T/T	Affx-108895751	3B	333099002
AX-109498003	G/G	A/G	G/G	Affx-88519652	3B	242644992
AX-109501026	C/C	A/C	C/C	Affx-109055035	3B	570630589
AX-109506024	T/T	T/C	T/T	Affx-109412049	3B	127815155
AX-109516466	A/A	A/G	A/A	Affx-108953660	3B	485233845
AX-109517771	T/T	C/C	T/T	Affx-110598964	3B	79757518
AX-109524188	T/T	T/T	T/C	Affx-109753188	3B	349292946
AX-109524255	A/A	A/A	A/A	Affx-88425817	3B	67061431
AX-109528490	A/A	A/G	A/A	Affx-110028396	3B	223347014
AX-109540016	C/C	T/C	C/C	Affx-110987380	3B	367846286
AX-109545021	C/C	C/C	C/C	Affx-110206023	3B	496613533
AX-109556014	G/G	T/T	T/G	Affx-88385121	3B	416690221
AX-109565402	T/T	T/T	T/T	Affx-109151644	3B	571797820
AX-109576658	C/C	T/C	T/C	Affx-111970233	3B	704760617
AX-109577406	C/C	T/C	T/T	Affx-109664924	3B	161452216
AX-109579163	C/C	T/C	C/C	Affx-110035344	3B	452577309
AX-109580558	G/G	A/G	G/G	Affx-109857049	3B	173040672
AX-109586689	G/G	A/G	G/G	Affx-109631128	3B	205017875
AX-109597708	C/C	T/C	C/C	Affx-111608874	3B	297628359
AX-109600737	T/T	T/C	T/T	Affx-110227933	3B	728080025
AX-109601926	A/A	A/C	A/A	Affx-110875858	3B	95407387
AX-109608571	T/T	T/C	T/T	Affx-111173048	3B	520966246
AX-109616863	T/T	T/T	T/T	Affx-110760930	3B	713121356
AX-109639680	A/G	A/A	A/G	Affx-109095271	3B	384307167

AX-109816383	C/C	C/G	C/C	Affx-111691871	3B	810305500
AX-109818115	C/C	T/C	C/C	Affx-111645223	3B	687070462
AX-109819224	G/G	C/G	G/G	Affx-111801625	3B	239651943
AX-109820425	G/G	A/G	G/G	Affx-88757602	3B	775477801
AX-109822302	G/G	A/G	G/G	Affx-110882804	3B	684410865
AX-109823525	T/T	T/C	T/T	Affx-109256856	3B	704620
AX-109823534	C/C	T/C	C/C	Affx-108923569	3B	246504870
AX-109823960	G/G	G/G	G/G	Affx-110942838	3B	522215560
AX-109824459	A/A	C/C	A/A	Affx-110584776	3B	476392908
AX-109830120	C/C	T/C	C/C	Affx-110207468	3B	733652421
AX-109838018	G/G	A/G	G/G	Affx-109254077	3B	771059006
AX-109843747	A/A	G/G	A/A	Affx-111628891	3B	618068302
AX-109843966	C/C	T/C	C/C	Affx-110047644	3B	431933549
AX-109846676	A/A	A/G	A/A	Affx-109164547	3B	762431703
AX-109847816	T/T	T/C	T/T	Affx-111070754	3B	134303979
AX-109851198	G/G	A/G	G/G	Affx-109990118	3B	499055944
AX-109852115	G/G	A/G	G/G	Affx-110449837	3B	130737993
AX-109856670	A/A	A/G	A/A	Affx-110113777	3B	583105331
AX-109857761	A/G	A/G	A/A	Affx-110748748	3B	8529719
AX-109857767	G/G	A/G	G/G	Affx-109503266	3B	354940656
AX-109859344	A/A	A/G	A/A	Affx-109327875	3B	311136321
AX-109860276	C/C	C/G	C/C	Affx-88421394	3B	305805032
AX-109865886	T/T	T/T	T/T	Affx-109433000	3B	617843270
AX-109868685	C/C	T/C	C/C	Affx-110072663	3B	291098014
AX-109868743	C/C	C/G	C/C	Affx-110264723	3B	48783951
AX-109869327	C/C	T/C	C/C	Affx-111788826	3B	735916548
AX-109870033	G/G	A/G	G/G	Affx-109899447	3B	173828879
AX-109875013	C/C	T/C	C/C	Affx-109145678	3B	523299540
AX-109881148	G/G	A/G	G/G	Affx-111277030	3B	793075912
AX-109884780	G/G	C/G	G/G	Affx-108988898	3B	373674365
AX-109885946	T/T	C/C	T/T	Affx-109100720	3B	256543783
AX-109888602	C/C	T/C	C/C	Affx-110578181	3B	62705179
AX-109888896	A/A	A/G	A/A	Affx-109098206	3B	626368819
AX-109895957	G/G	A/G	G/G	Affx-110216451	3B	852394
AX-109896119	A/A	A/G	A/A	Affx-109069546	3B	469761203
AX-109898057	C/C	T/T	C/C	Affx-111660223	3B	195646468
AX-109898213	G/G	T/G	G/G	Affx-111837024	3B	125550726
AX-109900531	T/T	T/C	T/T	Affx-110147578	3B	810347
AX-109908465	T/T	T/C	T/T	Affx-111001382	3B	803518201
AX-109908727	T/T	C/C	T/T	Affx-88422622	3B	148064322
AX-109908756	C/C	T/C	C/C	Affx-111960735	3B	567404389
AX-109909251	G/G	A/G	G/G	Affx-109063893	3B	384912818
AX-109910220	G/G	A/G	G/G	Affx-111517295	3B	383561659
AX-109910758	A/A	A/C	A/A	Affx-110262619	3B	59054885
AX-109913835	C/C	T/C	C/C	Affx-111096358	3B	84414549
AX-109914068	A/A	A/G	A/A	Affx-110919472	3B	710389123
AX-109914961	A/A	A/T	A/A	Affx-110333563	3B	77002784
AX-109916563	C/C	C/C	C/C	Affx-108900237	3B	40283206
AX-109917771	C/C	T/C	C/C	Affx-110763017	3B	821018272
AX-109918384	T/T	T/C	C/C	Affx-111807049	3B	157414447
AX-109919410	G/G	A/G	G/G	Affx-110934563	3B	352209956
AX-109919715	G/G	G/G	A/A	Affx-111956217	3B	161883858
AX-109921712	G/G	A/G	G/G	Affx-110552802	3B	813976090
AX-109922030	C/C	C/G	C/C	Affx-111312256	3B	666440590

AX-109923656	G/G	A/G	G/G	Affx-110847750	3B	749075585
AX-109925583	A/A	A/A	A/A	Affx-111095021	3B	819726035
AX-109926249	C/C	A/C	C/C	Affx-111803512	3B	219020051
AX-109928767	G/G	A/A	G/G	Affx-111792906	3B	465479015
AX-109930036	A/A	A/C	A/A	Affx-110572386	3B	6532260
AX-109932909	C/C	A/C	C/C	Affx-111306069	3B	328593499
AX-109936390	G/G	A/G	A/A	Affx-111736464	3B	165434771
AX-109936625	T/C	C/C	T/C	Affx-108890446	3B	503989114
AX-109946806	T/T	C/C	T/T	Affx-111726663	3B	560593489
AX-109947716	T/T	T/C	T/T	Affx-110573079	3B	70297703
AX-109948554	T/T	T/G	T/T	Affx-109393804	3B	48812520
AX-109949000	A/A	A/C	A/A	Affx-109149699	3B	740959951
AX-109949731	G/G	T/G	G/G	Affx-109827937	3B	448436709
AX-109949824	C/C	T/C	C/C	Affx-111085146	3B	779679244
AX-109950866	C/C	T/C	C/C	Affx-110228912	3B	592529930
AX-109951689	A/A	A/G	A/A	Affx-109248900	3B	351790841
AX-109952325	C/C	T/C	C/C	Affx-111749895	3B	190487924
AX-109952688	T/T	T/C	T/T	Affx-111016155	3B	625565078
AX-109966505	G/G	T/G	G/G	Affx-111176135	3B	740056716
AX-109967961	C/C	T/C	C/C	Affx-110928517	3B	314319717
AX-109973533	A/A	A/G	A/A	Affx-111248527	3B	774776246
AX-109973543	G/G	T/G	G/G	Affx-109297057	3B	493634836
AX-109974660	A/C	A/C	A/C	Affx-109088894	3B	598502162
AX-109977463	G/G	A/G	G/G	Affx-109631575	3B	470237410
AX-109982848	C/C	T/C	C/C	Affx-111312950	3B	252856693
AX-109983339	C/G	C/G	C/G	Affx-110242172	3B	702650072
AX-109989303	A/A	A/G	A/A	Affx-111038354	3B	53391818
AX-109995421	A/A	G/G	A/A	Affx-110068563	3B	144775288
AX-109997779	T/T	A/T	T/T	Affx-88678782	3B	759129141
AX-109997810	G/G	C/G	G/G	Affx-109788673	3B	761367399
AX-109998246	G/G	T/G	G/G	Affx-110588929	3B	265286196
AX-109999763	T/T	T/G	T/T	Affx-110817249	3B	139732938
AX-110001564	T/T	T/C	T/T	Affx-108940634	3B	17861649
AX-110003940	C/C	T/C	C/C	Affx-109071550	3B	78230553
AX-110006721	T/G	G/G	T/G	Affx-110275619	3B	13922164
AX-110007345	G/G	A/A	G/G	Affx-109743678	3B	129442308
AX-110010332	A/A	A/G	A/A	Affx-111373586	3B	308485819
AX-110012386	T/T	T/G	T/T	Affx-108889162	3B	602437857
AX-110015731	G/G	C/G	G/G	Affx-111353869	3B	225491211
AX-110020080	A/A	A/C	A/A	Affx-109169649	3B	717363041
AX-110020100	G/G	A/G	G/G	Affx-109160779	3B	433597031
AX-110023083	C/C	C/G	C/C	Affx-110382464	3B	722473040
AX-110023853	C/C	C/G	C/C	Affx-111590510	3B	103545894
AX-110027914	A/A	A/A	A/A	Affx-110421500	3B	276668715
AX-110029041	G/G	A/G	G/G	Affx-109368127	3B	36529142
AX-110037504	C/C	A/C	C/C	Affx-111077914	3B	111895480
AX-110038027	C/C	T/C	C/C	Affx-110544702	3B	561260728
AX-110043375	A/A	A/C	A/A	Affx-111897171	3B	663135418
AX-110046410	G/G	A/G	G/G	Affx-88356392	3B	96358483
AX-110046844	G/G	T/G	G/G	Affx-110380069	3B	456923752
AX-110054156	C/C	C/G	C/C	Affx-111240922	3B	490190391
AX-110054908	C/C	T/C	C/C	Affx-110881634	3B	188969255
AX-110055716	T/T	T/C	T/T	Affx-111939005	3B	319167808
AX-110057474	C/G	C/G	C/G	Affx-111070919	3B	468480672

AX-110057811	C/C	C/G	C/C	Affx-111175902	3B	192357090
AX-110059538	C/C	T/C	C/C	Affx-109927434	3B	247108576
AX-110064449	C/C	A/C	C/C	Affx-88726576	3B	698422386
AX-110064692	A/A	A/T	A/A	Affx-109814897	3B	422915980
AX-110065841	A/A	A/G	A/A	Affx-111955469	3B	663618446
AX-110066746	C/C	T/C	C/C	Affx-108926531	3B	215080075
AX-110078107	C/C	T/T	C/C	Affx-88365282	3B	605680984
AX-110078584	C/C	C/C	C/C	Affx-109503679	3B	132392796
AX-110087708	C/C	T/C	C/C	Affx-111306722	3B	183540084
AX-110089945	T/T	T/C	T/T	Affx-109381836	3B	489347129
AX-110092119	T/T	T/C	T/T	Affx-109404288	3B	513811949
AX-110098038	C/C	T/C	C/C	Affx-109344749	3B	482253775
AX-110121230	G/G	A/G	G/G	Affx-111407510	3B	420259844
AX-110121516	T/T	T/G	T/T	Affx-111540841	3B	463769637
AX-110122585	T/T	T/C	T/T	Affx-110215939	3B	787966608
AX-110127112	G/G	A/G	G/G	Affx-108951517	3B	535932133
AX-110127873	C/C	C/C	C/C	Affx-109185893	3B	734485466
AX-110129723	T/T	T/T	T/T	Affx-111157146	3B	732604431
AX-110145424	T/T	T/C	T/T	Affx-111026258	3B	62019866
AX-110147189	G/G	A/G	G/G	Affx-110861918	3B	201301378
AX-110161789	C/C	T/C	C/C	Affx-111518462	3B	692704332
AX-110176190	G/G	G/G	G/G	Affx-111952260	3B	710981540
AX-110186181	T/T	T/G	T/T	Affx-110330052	3B	83458933
AX-110186589	A/A	G/G	A/A	Affx-110468413	3B	376813920
AX-110201577	T/T	T/C	T/T	Affx-111548511	3B	466677284
AX-110365211	T/G	T/G	T/G	Affx-109063827	3B	244022033
AX-110369753	A/A	A/G	A/A	Affx-110400812	3B	752092733
AX-110369837	C/C	C/G	C/C	Affx-111180089	3B	718852343
AX-110371960	T/T	C/C	T/T	Affx-110403702	3B	2335309
AX-110374110	C/C	C/C	C/C	Affx-111572622	3B	239229887
AX-110375013	C/C	A/C	C/C	Affx-109503618	3B	675645832
AX-110385548	C/C	T/C	C/C	Affx-88568732	3B	560120140
AX-110388325	G/G	C/G	G/G	Affx-109780118	3B	458286638
AX-110388372	G/G	G/G	A/A	Affx-110152110	3B	162196290
AX-110388654	C/C	A/C	C/C	Affx-109004184	3B	596753225
AX-110391554	G/G	T/G	G/G	Affx-109957875	3B	747333806
AX-110393576	T/T	T/T	T/T	Affx-110050059	3B	688166078
AX-110396359	T/T	T/C	T/T	Affx-109352502	3B	368583270
AX-110397286	C/C	A/C	C/C	Affx-109956675	3B	296900027
AX-110397799	A/A	A/G	A/A	Affx-111551612	3B	144903259
AX-110399852	G/G	G/G	G/G	Affx-111058764	3B	255698913
AX-110401384	A/A	A/G	A/A	Affx-108988812	3B	127853486
AX-110402355	G/G	C/G	G/G	Affx-110564969	3B	404838851
AX-110402477	T/T	T/G	T/T	Affx-109832307	3B	146283576
AX-110403159	T/T	A/T	T/T	Affx-109208439	3B	40570476
AX-110404067	A/A	A/G	A/A	Affx-109289036	3B	492347539
AX-110406955	A/A	A/G	A/A	Affx-111651945	3B	708393013
AX-110407488	C/C	C/G	C/C	Affx-111194393	3B	693192098
AX-110409055	T/T	T/C	T/T	Affx-110773888	3B	571142331
AX-110410665	C/C	T/C	C/C	Affx-110731999	3B	479502603
AX-110412449	G/G	T/G	G/G	Affx-111630182	3B	828111067
AX-110413790	T/T	T/C	T/T	Affx-109906164	3B	502839623
AX-110417292	G/G	A/G	G/G	Affx-111133422	3B	291922715
AX-110418888	A/A	A/G	A/A	Affx-109032019	3B	785432286

AX-110420501	A/A	A/G	A/A	Affx-109453693	3B	82055926
AX-110424510	C/C	C/C	C/C	Affx-110911928	3B	495122102
AX-110428478	A/A	A/G	A/A	Affx-109659047	3B	826058509
AX-110434278	C/C	T/C	C/C	Affx-110327933	3B	121249574
AX-110434901	G/G	C/G	G/G	Affx-111065545	3B	509703787
AX-110442253	A/A	A/G	A/A	Affx-109101804	3B	498263547
AX-110446018	T/T	T/G	T/T	Affx-88430105	3B	512775287
AX-110448973	T/T	T/C	T/T	Affx-110093333	3B	633277598
AX-110449094	G/G	G/G	G/G	Affx-109070462	3B	546908768
AX-110453095	T/T	T/T	T/T	Affx-109239417	3B	682805336
AX-110453546	T/T	C/C	T/T	Affx-110578251	3B	308024936
AX-110454918	A/A	A/G	A/A	Affx-110600754	3B	77689598
AX-110458083	C/C	T/C	T/T	Affx-108939141	3B	148686209
AX-110459328	A/A	C/C	A/A	Affx-109781078	3B	568258798
AX-110463370	C/C	T/C	C/C	Affx-110841501	3B	75068641
AX-110463744	T/T	T/C	T/T	Affx-110381775	3B	751059749
AX-110464999	G/G	A/G	G/G	Affx-109149951	3B	187591224
AX-110465107	C/C	T/C	C/C	Affx-110003177	3B	820859582
AX-110466545	T/T	A/T	T/T	Affx-110033419	3B	472866095
AX-110466741	T/G	T/G	T/G	Affx-92634867	3B	545010427
AX-110467038	T/T	C/C	T/T	Affx-110335766	3B	451326529
AX-110467612	A/A	A/G	A/A	Affx-110978076	3B	38066589
AX-110468691	A/G	G/G	A/G	Affx-111751592	3B	737432678
AX-110469464	C/C	T/C	C/C	Affx-109678033	3B	724908584
AX-110475726	C/C	A/C	C/C	Affx-111437256	3B	699502711
AX-110477578	A/A	G/G	A/A	Affx-109960120	3B	61432791
AX-110481138	T/T	T/T	T/T	Affx-111146357	3B	80874937
AX-110486206	T/C	T/C	T/C	Affx-109694084	3B	50041464
AX-110487433	A/A	A/A	A/A	Affx-109754864	3B	502085125
AX-110490439	C/C	C/C	C/C	Affx-111833215	3B	575194074
AX-110492055	C/C	T/C	C/C	Affx-109000834	3B	152129909
AX-110492252	T/T	T/T	T/T	Affx-111464838	3B	605014517
AX-110493720	A/A	A/G	A/A	Affx-109352500	3B	798205726
AX-110495689	A/A	A/C	A/A	Affx-111347417	3B	72319717
AX-110498328	C/C	T/C	C/C	Affx-109731256	3B	380237460
AX-110501722	C/C	A/C	C/C	Affx-109375852	3B	553728315
AX-110502590	G/G	C/G	G/G	Affx-111130592	3B	627139543
AX-110505528	C/C	T/T	C/C	Affx-110943385	3B	280111710
AX-110506853	C/C	T/C	C/C	Affx-110947612	3B	119222104
AX-110507612	C/C	C/C	C/C	Affx-108921036	3B	721204128
AX-110512664	C/C	C/G	C/C	Affx-111793984	3B	408589368
AX-110512827	T/T	T/C	T/T	Affx-108853911	3B	211144526
AX-110514010	T/T	T/G	T/T	Affx-109315939	3B	269253129
AX-110518110	T/T	T/C	T/T	Affx-110843468	3B	512859298
AX-110520630	G/G	C/G	G/G	Affx-109255604	3B	233566221
AX-110521277	G/G	A/G	G/G	Affx-109470107	3B	815473690
AX-110522553	A/A	A/G	A/A	Affx-109647602	3B	817315199
AX-110525576	G/G	A/G	G/G	Affx-110980444	3B	360089288
AX-110530007	G/G	C/G	G/G	Affx-111565597	3B	680405
AX-110532165	G/G	A/G	G/G	Affx-109728318	3B	253299895
AX-110534176	C/C	C/G	C/C	Affx-110620134	3B	145696681
AX-110535477	C/C	A/C	C/C	Affx-88406409	3B	114705252
AX-110537855	T/T	C/C	T/T	Affx-111524572	3B	413785498
AX-110538297	C/C	T/C	C/C	Affx-111587381	3B	726079249

AX-110547011	C/C	A/C	C/C	Affx-111037232	3B	379113845
AX-110548993	G/G	A/G	G/G	Affx-110476486	3B	65160500
AX-110552499	G/G	A/G	G/G	Affx-109161610	3B	748599308
AX-110555494	T/T	T/C	T/T	Affx-110368192	3B	497909138
AX-110555657	C/C	C/G	C/C	Affx-110766358	3B	124820642
AX-110555684	C/C	T/C	C/C	Affx-110168367	3B	478663267
AX-110560303	C/C	A/C	A/C	Affx-110351448	3B	101991336
AX-110560395	C/C	T/C	C/C	Affx-109720959	3B	172162096
AX-110560967	G/G	A/G	G/G	Affx-109782301	3B	598860826
AX-110565050	G/G	A/G	G/G	Affx-110012024	3B	422135614
AX-110569582	C/C	T/C	C/C	Affx-110385205	3B	677184808
AX-110570027	A/A	C/C	A/A	Affx-111644269	3B	494686691
AX-110571583	T/T	T/C	T/T	Affx-111484648	3B	795998963
AX-110578998	C/C	T/T	C/C	Affx-111927774	3B	251278175
AX-110582386	G/G	A/G	G/G	Affx-109550321	3B	573575010
AX-110589424	A/A	G/G	A/A	Affx-109086444	3B	708910315
AX-110594860	A/A	A/G	A/A	Affx-110984597	3B	360891934
AX-110595452	G/G	T/G	G/G	Affx-111265872	3B	377706871
AX-110595716	T/T	T/C	T/T	Affx-109104353	3B	190952373
AX-110596585	A/A	A/G	A/A	Affx-110579086	3B	364785925
AX-110603776	C/C	T/C	C/C	Affx-110712657	3B	561689729
AX-110611050	G/G	A/G	G/G	Affx-109967769	3B	372615881
AX-110619125	G/G	A/G	G/G	Affx-111049451	3B	224321708
AX-110625472	A/A	A/A	A/A	Affx-110185556	3B	23683022
AX-110629944	C/C	C/G	C/C	Affx-109651058	3B	30535199
AX-110632321	A/A	A/G	A/A	Affx-109912513	3B	262733030
AX-110632424	C/C	T/C	C/C	Affx-109879602	3B	692044031
AX-110636902	G/G	A/G	G/G	Affx-110332569	3B	666932757
AX-110642930	A/A	G/G	A/A	Affx-109941612	3B	245504315
AX-110667739	T/T	T/C	T/T	Affx-111907977	3B	31359950
AX-110668896	G/G	A/G	G/G	Affx-108946769	3B	662966013
AX-110669263	C/C	T/C	C/C	Affx-109655929	3B	709485000
AX-110674067	A/G	G/G	A/G	Affx-111778667	3B	523797691
AX-110675086	C/C	T/C	C/C	Affx-110803860	3B	544107766
AX-110675833	C/C	T/C	C/C	Affx-110816982	3B	49362975
AX-110687689	A/A	A/G	A/A	Affx-110013563	3B	684499245
AX-110693349	C/C	T/C	C/C	Affx-110381402	3B	455134671
AX-110696526	A/A	A/G	A/A	Affx-111634052	3B	359154850
AX-110703410	T/T	T/G	T/T	Affx-110205379	3B	294336295
AX-110705689	A/A	C/C	C/C	Affx-110552717	3B	162766575
AX-110707418	G/G	G/G	G/G	Affx-109642191	3B	470790712
AX-110731274	T/T	T/C	T/T	Affx-111017056	3B	332011251
AX-110736452	G/G	G/G	G/G	Affx-109611640	3B	323129271
AX-110741718	A/A	A/G	A/A	Affx-111645655	3B	625190335
AX-110745662	A/A	A/G	A/A	Affx-110538970	3B	315276377
AX-110746369	T/T	C/C	T/T	Affx-111442012	3B	238594755
AX-110907148	C/C	C/C	C/C	Affx-111964088	3B	105721113
AX-110907745	T/T	T/C	T/T	Affx-111685342	3B	109506362
AX-110911256	A/A	G/G	A/A	Affx-111529757	3B	275111539
AX-110915949	A/A	A/G	G/G	Affx-109035355	3B	163705451
AX-110917789	A/A	A/G	A/A	Affx-110509728	3B	764683626
AX-110918326	C/C	A/C	C/C	Affx-108928762	3B	414004908
AX-110918414	A/A	A/G	A/A	Affx-109913152	3B	595285917
AX-110918569	C/C	C/C	C/C	Affx-110063649	3B	438549269

AX-110919058	C/C	C/C	C/C	Affx-110778587	3B	110440349
AX-110919590	G/G	C/G	G/G	Affx-88699759	3B	704324505
AX-110924608	C/C	A/C	C/C	Affx-110847065	3B	475027006
AX-110926626	C/C	C/G	C/C	Affx-110981485	3B	199055121
AX-110928735	G/G	T/G	G/G	Affx-110393578	3B	587726214
AX-110931611	T/T	C/C	T/T	Affx-110270161	3B	629541335
AX-110933729	T/C	T/C	T/C	Affx-111605644	3B	732794365
AX-110934652	T/T	T/G	T/T	Affx-110409460	3B	96833319
AX-110935708	C/C	T/C	C/C	Affx-109830206	3B	709920915
AX-110935747	A/A	A/C	A/A	Affx-109896240	3B	54865590
AX-110936863	T/T	T/G	T/T	Affx-110370463	3B	285478152
AX-110937931	A/A	A/G	A/A	Affx-109570260	3B	587638317
AX-110944855	C/C	A/C	C/C	Affx-110504666	3B	492681586
AX-110947001	A/A	A/G	A/A	Affx-109333828	3B	273155840
AX-110949684	C/C	T/C	C/C	Affx-111896455	3B	665266915
AX-110949869	G/G	A/G	G/G	Affx-111967490	3B	802706579
AX-110950334	A/A	A/G	A/A	Affx-108983617	3B	311311580
AX-110955883	T/T	T/G	T/T	Affx-111391567	3B	201856167
AX-110962754	A/A	A/T	A/A	Affx-110461391	3B	738471467
AX-110966342	G/G	A/G	G/G	Affx-108926422	3B	305521013
AX-110967082	C/C	T/T	C/C	Affx-110615834	3B	295402622
AX-110967267	C/C	T/C	C/C	Affx-109931499	3B	759269484
AX-110968381	G/G	G/G	G/G	Affx-111942314	3B	607839898
AX-110968918	T/T	T/C	T/T	Affx-111329677	3B	555108521
AX-110973312	A/A	A/G	A/A	Affx-110194105	3B	228215911
AX-110975611	G/G	C/C	G/G	Affx-111864363	3B	730778944
AX-110977565	A/A	A/A	A/A	Affx-111123720	3B	386826626
AX-110978195	T/C	T/C	T/C	Affx-111282806	3B	547605375
AX-110978224	C/C	C/G	C/C	Affx-110920909	3B	695351048
AX-110982148	C/C	A/C	A/A	Affx-110286173	3B	171601226
AX-110985096	G/G	C/G	G/G	Affx-109195696	3B	719824246
AX-110985949	G/G	A/G	G/G	Affx-109740341	3B	299744465
AX-110991513	A/A	A/G	A/A	Affx-111130358	3B	244974398
AX-110991543	G/G	A/G	G/G	Affx-111820789	3B	697066748
AX-110992117	G/G	A/G	G/G	Affx-110815995	3B	703296419
AX-110993591	C/C	T/C	C/C	Affx-109854106	3B	811774176
AX-110995995	A/A	A/G	A/A	Affx-111919307	3B	318155028
AX-110998126	A/A	A/G	A/A	Affx-109247505	3B	317166456
AX-111005155	T/T	T/T	T/T	Affx-111668104	3B	467087129
AX-111005286	T/T	T/C	T/T	Affx-108854730	3B	299916460
AX-111005371	G/G	T/G	G/G	Affx-110105327	3B	259086198
AX-111009485	T/T	T/G	T/T	Affx-111166864	3B	243551802
AX-111012654	A/A	A/G	A/A	Affx-109969009	3B	534967702
AX-111014939	A/A	G/G	A/A	Affx-109535381	3B	367479279
AX-111017737	T/C	T/T	T/C	Affx-108968930	3B	122898368
AX-111026930	T/T	T/G	T/T	Affx-111008927	3B	489599090
AX-111032146	A/T	T/T	A/T	Affx-88568152	3B	112496447
AX-111034136	T/T	T/T	T/T	Affx-111354834	3B	630113507
AX-111035325	G/G	A/G	G/G	Affx-108940784	3B	174969988
AX-111036738	T/T	T/C	T/T	Affx-110796664	3B	580515292
AX-111038183	A/A	A/C	A/A	Affx-110562888	3B	243122717
AX-111045227	T/T	T/C	T/T	Affx-111335847	3B	636606568
AX-111047357	A/A	G/G	A/A	Affx-111514698	3B	131328597
AX-111049794	C/C	T/C	C/C	Affx-110204575	3B	116062830

AX-111049873	T/T	T/G	G/G	Affx-111688436	3B	13961173
AX-111052161	G/G	C/G	G/G	Affx-111552522	3B	44847495
AX-111054315	A/A	A/G	A/A	Affx-110867849	3B	686562308
AX-111055895	T/T	T/C	T/T	Affx-110621548	3B	390462076
AX-111059473	T/T	T/C	T/T	Affx-110651232	3B	117601160
AX-111060112	C/C	T/C	C/C	Affx-109570529	3B	253946072
AX-111060272	T/T	T/T	T/T	Affx-111772132	3B	38507491
AX-111064745	A/A	A/C	A/A	Affx-88558779	3B	781670598
AX-111065820	C/C	T/C	C/C	Affx-111410440	3B	352738605
AX-111065940	G/G	A/G	G/G	Affx-109230514	3B	316302855
AX-111066145	T/T	T/C	T/T	Affx-111289694	3B	314344631
AX-111067310	C/C	T/C	C/C	Affx-88763990	3B	583828554
AX-111078172	A/A	A/G	A/A	Affx-110934845	3B	321589691
AX-111078613	G/G	A/G	G/G	Affx-111950315	3B	578872313
AX-111080504	A/A	A/G	A/A	Affx-109054355	3B	29378527
AX-111082444	G/G	A/A	G/G	Affx-111213422	3B	136337889
AX-111082743	G/G	A/G	A/A	Affx-111534173	3B	158255403
AX-111087147	T/T	T/C	T/T	Affx-110025754	3B	765322402
AX-111089329	C/C	C/C	C/C	Affx-111998463	3B	30961339
AX-111092558	G/G	A/G	G/G	Affx-111533272	3B	137690114
AX-111092736	T/T	T/C	T/T	Affx-109989147	3B	457755811
AX-111094165	A/G	A/G	A/A	Affx-108859813	3B	8526844
AX-111107270	C/C	A/C	C/C	Affx-109046469	3B	771735967
AX-111108902	T/T	T/C	T/T	Affx-110583288	3B	237215791
AX-111111941	G/G	A/G	G/G	Affx-109737809	3B	32759323
AX-111112626	C/C	T/C	C/C	Affx-109011890	3B	655419174
AX-111118939	C/C	T/T	C/C	Affx-111576806	3B	543024314
AX-111121613	A/A	G/G	A/A	Affx-109463200	3B	116973899
AX-111122394	G/G	A/G	G/G	Affx-109343514	3B	326100901
AX-111125878	T/T	T/C	T/T	Affx-110114781	3B	500321893
AX-111126455	G/G	A/G	G/G	Affx-109748277	3B	197533719
AX-111128552	A/A	A/G	A/A	Affx-110975404	3B	79273088
AX-111134381	C/C	C/G	C/C	Affx-111265448	3B	543625285
AX-111134770	A/A	G/G	A/A	Affx-110785651	3B	359068053
AX-111135493	G/G	T/G	G/G	Affx-109525895	3B	226959197
AX-111137087	G/G	G/G	G/G	Affx-108893448	3B	730289551
AX-111142543	T/T	T/C	T/T	Affx-109399993	3B	774055431
AX-111143672	C/C	C/G	C/C	Affx-109118182	3B	121851856
AX-111146964	T/C	C/C	T/C	Affx-111672767	3B	113120924
AX-111148430	C/C	T/C	C/C	Affx-111128458	3B	144573595
AX-111150812	A/A	A/G	A/A	Affx-111328968	3B	693338012
AX-111153177	A/A	A/G	A/A	Affx-109460471	3B	569411550
AX-111175175	C/C	T/C	C/C	Affx-88767625	3B	539563314
AX-111180833	G/G	G/G	G/G	Affx-111054630	3B	361753115
AX-111216623	G/G	A/G	G/G	Affx-111676892	3B	321439066
AX-111217864	G/G	A/A	G/G	Affx-109378815	3B	273694640
AX-111218149	C/C	T/C	C/C	Affx-111707875	3B	553184800
AX-111221059	C/C	T/C	C/C	Affx-110244737	3B	495812507
AX-111222212	T/T	T/C	T/T	Affx-111329659	3B	298327194
AX-111234034	G/G	T/G	G/G	Affx-111794951	3B	228876362
AX-111238935	G/G	A/G	G/G	Affx-109463115	3B	747333648
AX-111239152	T/T	T/C	T/T	Affx-109475071	3B	579620189
AX-111241221	A/A	A/C	A/A	Affx-111156957	3B	355977084
AX-111254437	G/G	A/A	G/G	Affx-110681226	3B	524769466

AX-111289488	C/C	T/C	C/C	Affx-109329260	3B	435300438
AX-111452616	G/G	T/G	G/G	Affx-111251727	3B	136944287
AX-111452618	C/C	A/C	C/C	Affx-111073109	3B	487482948
AX-111453796	A/A	A/G	A/A	Affx-109483419	3B	425392540
AX-111460004	T/T	C/C	T/T	Affx-110206629	3B	474362420
AX-111460388	T/T	T/C	T/T	Affx-111992885	3B	118758942
AX-111460582	G/G	A/G	G/G	Affx-109075008	3B	626895720
AX-111462429	C/C	A/C	C/C	Affx-111473241	3B	811081371
AX-111462497	C/C	T/C	C/C	Affx-111847864	3B	221175941
AX-111464292	G/G	A/G	G/G	Affx-111644831	3B	486363415
AX-111466357	G/G	C/G	G/G	Affx-109782254	3B	798946140
AX-111467893	G/G	C/G	G/G	Affx-111531859	3B	284359076
AX-111470695	A/A	A/G	A/A	Affx-111564892	3B	449050969
AX-111471417	T/T	T/C	T/T	Affx-110337167	3B	363216083
AX-111471993	G/G	A/G	G/G	Affx-111102982	3B	44295939
AX-111473833	A/A	A/G	A/A	Affx-109192288	3B	289490443
AX-111474329	C/C	T/C	C/C	Affx-110159483	3B	250202053
AX-111485400	T/T	T/C	T/T	Affx-111974395	3B	501103637
AX-111487253	A/A	A/G	A/A	Affx-109826799	3B	13947301
AX-111487323	A/G	G/G	A/G	Affx-109525657	3B	25660580
AX-111490441	G/G	A/G	G/G	Affx-109011327	3B	131508802
AX-111493700	A/A	A/G	G/G	Affx-110909578	3B	160824814
AX-111496230	A/A	A/G	A/A	Affx-109861413	3B	550732999
AX-111498247	A/A	G/G	A/A	Affx-109573811	3B	476995793
AX-111498514	T/T	T/G	T/T	Affx-110659078	3B	800076
AX-111499744	G/G	A/G	G/G	Affx-109592937	3B	223500579
AX-111499878	C/C	A/C	A/A	Affx-111512216	3B	164454009
AX-111500014	C/C	T/C	C/C	Affx-109340352	3B	106282187
AX-111500132	A/A	C/C	A/A	Affx-109706098	3B	582752000
AX-111500891	T/T	T/T	T/T	Affx-111552410	3B	588202868
AX-111502226	G/G	C/G	G/G	Affx-110188431	3B	808674178
AX-111505239	T/T	T/G	T/T	Affx-110113877	3B	556516090
AX-111507044	A/A	A/G	A/A	Affx-109472923	3B	562208657
AX-111516675	T/T	T/C	C/C	Affx-111601527	3B	172731792
AX-111520472	G/G	C/G	G/G	Affx-111822310	3B	265005744
AX-111522812	G/G	A/G	G/G	Affx-111005151	3B	19076222
AX-111523002	G/G	T/G	G/G	Affx-109640909	3B	55369721
AX-111527380	A/A	A/G	A/A	Affx-110753235	3B	728600649
AX-111527419	C/C	T/C	C/C	Affx-111071280	3B	120447221
AX-111528074	G/G	A/G	G/G	Affx-111365896	3B	711539558
AX-111528429	G/G	A/G	G/G	Affx-109840329	3B	299248350
AX-111529839	C/C	T/C	C/C	Affx-109533364	3B	605479686
AX-111534973	C/C	T/C	C/C	Affx-111400327	3B	41633676
AX-111535241	T/T	T/G	T/T	Affx-109168415	3B	700077821
AX-111538441	C/C	A/C	C/C	Affx-109749410	3B	739578525
AX-111538584	C/C	C/C	C/C	Affx-110530699	3B	508979462
AX-111543811	A/A	A/G	A/A	Affx-110860738	3B	779365674
AX-111547004	T/T	T/C	T/T	Affx-109693357	3B	774970334
AX-111549070	T/T	T/C	T/T	Affx-110955682	3B	21144644
AX-111550253	C/C	T/C	C/C	Affx-109035550	3B	484237988
AX-111554419	C/C	T/C	C/C	Affx-110351610	3B	852727
AX-111555405	G/G	A/G	G/G	Affx-109077491	3B	284666644
AX-111556075	G/G	A/G	G/G	Affx-109762825	3B	520180546
AX-111556751	T/C	T/C	C/C	Affx-88495130	3B	8525959

AX-111557528	A/A	A/G	A/A	Affx-111721625	3B	482971892
AX-111562340	C/C	C/C	C/C	Affx-109924298	3B	740571241
AX-111564047	T/T	T/T	T/G	Affx-110743734	3B	7065119
AX-111564611	G/G	A/G	G/G	Affx-110365224	3B	335094380
AX-111568941	T/C	T/C	T/T	Affx-110439568	3B	8529361
AX-111575503	G/G	C/G	G/G	Affx-111028021	3B	59921458
AX-111575709	G/G	A/G	G/G	Affx-109003339	3B	350255587
AX-111575846	A/A	A/G	A/A	Affx-109034874	3B	486865816
AX-111585816	G/G	T/G	G/G	Affx-111252679	3B	44414888
AX-111589131	T/T	T/C	T/T	Affx-109711768	3B	132651306
AX-111592969	T/T	T/C	T/T	Affx-109995629	3B	377105899
AX-111596195	G/G	T/T	T/T	Affx-109702419	3B	166506151
AX-111597310	G/G	G/G	A/A	Affx-109811717	3B	156109579
AX-111598479	C/C	C/C	C/C	Affx-88394271	3B	394098359
AX-111609440	C/C	T/C	C/C	Affx-111627401	3B	482777022
AX-111627751	C/C	C/C	C/C	Affx-110954696	3B	135708637
AX-111629154	C/C	G/G	C/C	Affx-110532698	3B	338527758
AX-111632768	A/A	A/C	A/A	Affx-110970068	3B	363599606
AX-111644158	C/C	A/C	C/C	Affx-111551922	3B	113971433
AX-111651783	T/T	T/C	T/T	Affx-110952889	3B	32415185
AX-111657644	A/A	A/C	A/A	Affx-111374053	3B	800251
AX-111659275	G/G	A/G	G/G	Affx-111310456	3B	818500773
AX-111665680	G/G	G/G	G/G	Affx-109712999	3B	231985003
AX-111676302	T/T	T/C	T/T	Affx-109905998	3B	590611413
AX-111679454	G/G	G/G	G/G	Affx-111653694	3B	103091700
AX-111688452	C/C	T/C	T/T	Affx-111695570	3B	167108564
AX-111689619	G/G	A/G	G/G	Affx-111085342	3B	310290191
AX-111709142	G/G	G/G	G/G	Affx-110044225	3B	450849184
AX-111718394	A/A	A/G	A/A	Affx-111341345	3B	222819213
AX-111742012	G/G	A/G	G/G	Affx-88745387	3B	487882549
AX-111761312	T/T	T/T	T/T	Affx-111554427	3B	665985296
AX-111765564	T/T	T/C	T/T	Affx-110183634	3B	102574451
AX-111806699	G/G	A/G	G/G	Affx-110516470	3B	287237222
AX-111819095	A/A	A/A	A/A	Affx-111861269	3B	306782490
AX-112287824	T/T	T/T	T/T	Affx-112312628	3B	368904550
AX-112287914	T/T	T/C	T/T	Affx-112315780	3B	700793486
AX-112289221	T/T	T/T	T/T	Affx-88524196	3B	626090095
AX-181995637	C/C	A/C	C/C	Affx-88707342	3B	13925695
AX-182017117	G/G	T/G	G/G	Affx-109663922	3B	803196
AX-182075826	C/C	C/G	C/C	Affx-109463018	3B	693344097
AX-86164425	G/G	G/G	G/G	Affx-109792367	3B	8528337
AX-86165721	T/C	T/C	C/C	Affx-92825371	3B	8528973
AX-86168146	G/G	A/G	G/G	Affx-88646040	3B	630003884
AX-86176142	G/G	A/G	G/G	Affx-92274183	3B	705817220
AX-89331046	C/C	C/G	C/C	Affx-88362356	3B	781210302
AX-89334206	T/T	T/C	T/T	Affx-88365628	3B	697473092
AX-94383985	T/C	T/T	T/C	Affx-92660727	3B	147683153
AX-94388240	A/A	A/G	A/A	Affx-92325889	3B	521699151
AX-94408616	G/G	A/G	G/G	Affx-88426424	3B	232636240
AX-94415578	C/C	C/C	C/C	Affx-92403198	3B	50535078
AX-94425087	C/C	T/C	C/C	Affx-111757562	3B	772399790
AX-94430973	G/G	T/G	G/G	Affx-92932689	3B	18873524
AX-94474208	T/T	T/T	T/T	Affx-92409621	3B	609306781
AX-94479654	C/C	C/C	C/C	Affx-88741840	3B	778277374

AX-94486206	T/T	T/T	T/T	Affx-111056338	3B	664896468
AX-94497295	A/A	A/G	A/A	Affx-92422523	3B	485388046
AX-94525566	G/G	A/G	G/G	Affx-92763287	3B	232568881
AX-94541525	C/C	C/C	C/C	Affx-92248361	3B	511072341
AX-94580358	T/T	T/T	T/T	Affx-92378364	3B	373133501
AX-94585575	A/A	A/G	A/A	Affx-92812696	3B	745733321
AX-94673418	T/C	C/C	C/C	Affx-92710367	3B	8529086
AX-94678554	G/G	C/G	G/G	Affx-92529090	3B	568572005
AX-94680435	A/G	A/A	A/G	Affx-92274386	3B	652967775
AX-94690361	C/C	A/C	C/C	Affx-292486533	3B	730309957
AX-94691217	A/G	A/G	G/G	Affx-92832137	3B	8030002
AX-94701598	C/C	T/T	C/C	Affx-88631221	3B	222192138
AX-94719696	C/C	C/C	C/C	Affx-92544083	3B	574699008
AX-94746929	T/C	C/C	C/C	Affx-92629785	3B	800933346
AX-94772565	A/A	G/G	A/A	Affx-88528225	3B	480343218
AX-94776650	C/C	T/T	C/C	Affx-92669386	3B	8529089
AX-94778032	T/C	C/C	T/C	Affx-92770860	3B	723443994
AX-94781108	T/T	T/C	T/T	Affx-92770939	3B	597048703
AX-94834964	T/C	T/T	C/C	Affx-92419451	3B	778483814
AX-94851468	C/C	C/C	C/C	Affx-92180182	3B	723032017
AX-94870174	C/C	T/C	C/C	Affx-92645543	3B	8529176
AX-94891019	C/C	A/C	C/C	Affx-92553197	3B	8529171
AX-94960477	A/A	A/A	A/A	Affx-92996317	3B	798549518
AX-94961252	C/C	C/C	C/C	Affx-92783377	3B	8529194
AX-94966844	C/C	A/C	C/C	Affx-92238142	3B	5951921
AX-94973648	A/G	G/G	A/G	Affx-88770148	3B	567245879
AX-95007989	A/A	A/G	G/G	Affx-92580135	3B	11087306
AX-95020695	C/C	C/G	C/C	Affx-92952785	3B	581968958
AX-95141357	T/T	T/C	T/T	Affx-88355253	3B	622082295
AX-95149573	G/G	C/G	C/C	Affx-92855196	3B	469339536
AX-95630127	A/G	A/G	A/G	Affx-88444322	3B	682905538
AX-95631919	G/G	A/G	G/G	Affx-88521784	3B	542918623
AX-95632500	G/G	G/G	G/G	Affx-92896163	3B	766642477
AX-95634266	T/T	T/C	T/T	Affx-112314754	3B	700793524
AX-95660711	C/C	C/C	C/C	Affx-92305584	3B	18248020
AX-95662394	G/G	G/G	G/G	Affx-92212306	3B	611176652
AX-95681447	T/G	T/T	T/G	Affx-88508805	3B	9778282
AX-95684555	G/G	A/A	G/G	Affx-88356514	3B	566278179
AX-108726768	G/G	T/G	G/G	Affx-111593999	3D	355298544
AX-108729781	A/A	A/C	A/A	Affx-109863305	3D	537288463
AX-108743433	G/G	A/A	G/G	Affx-110681063	3D	608199658
AX-108746383	T/T	T/T	T/T	Affx-111399374	3D	583225232
AX-108756011	C/C	C/G	C/C	Affx-88716161	3D	166966216
AX-108756717	C/C	C/G	C/C	Affx-111600401	3D	231987021
AX-108762281	A/A	A/G	A/A	Affx-109550027	3D	45536948
AX-108788717	G/G	G/G	G/G	Affx-110291390	3D	329440343
AX-108791525	C/C	T/C	C/C	Affx-110085247	3D	115948335
AX-108794764	T/T	T/T	T/T	Affx-109079118	3D	72674445
AX-108796887	A/A	A/G	A/A	Affx-88794818	3D	309005131
AX-108800061	C/C	T/C	C/C	Affx-111262985	3D	48813095
AX-108813316	T/T	T/C	T/T	Affx-111290878	3D	453813853
AX-108814752	C/C	C/G	C/C	Affx-111502530	3D	306499252
AX-108814935	C/C	C/C	C/C	Affx-110520321	3D	221688128
AX-108822255	T/T	T/C	T/T	Affx-111537407	3D	555788926

AX-108830038	C/C	T/C	C/C	Affx-110078763	3D	210397227
AX-108835458	G/G	G/G	G/G	Affx-110745779	3D	496096057
AX-108841875	C/C	T/C	C/C	Affx-109228314	3D	228457205
AX-108844351	G/G	A/G	G/G	Affx-111269396	3D	436163601
AX-108852641	C/C	C/C	C/C	Affx-109346970	3D	43382936
AX-108855898	T/T	T/C	T/T	Affx-110105569	3D	167558513
AX-108855919	G/G	T/G	G/G	Affx-111483811	3D	330657683
AX-108859112	T/T	T/C	T/T	Affx-109220178	3D	139250658
AX-108874896	C/C	T/C	C/C	Affx-110108347	3D	168363074
AX-108883881	C/C	T/C	C/C	Affx-111195933	3D	354035297
AX-108886572	T/T	T/C	T/T	Affx-109298580	3D	212145323
AX-108887549	T/T	T/C	T/T	Affx-109975272	3D	432548137
AX-108889732	T/T	A/T	T/T	Affx-111698274	3D	516523905
AX-108889888	C/C	T/C	C/C	Affx-111604592	3D	231591719
AX-108890092	A/A	A/G	A/A	Affx-88450439	3D	552579234
AX-108891293	G/G	T/G	G/G	Affx-111298325	3D	418788268
AX-108895854	A/A	A/G	A/A	Affx-111853474	3D	184816308
AX-108895977	C/C	C/C	C/C	Affx-111787126	3D	184119274
AX-108898785	T/T	T/C	T/T	Affx-111156238	3D	170935503
AX-108900736	A/A	A/G	A/A	Affx-111188860	3D	453091459
AX-108907834	T/T	T/C	T/T	Affx-111723068	3D	28139557
AX-108922865	G/G	A/G	G/G	Affx-108908963	3D	193535792
AX-108923200	G/G	A/G	G/G	Affx-108965658	3D	462246045
AX-108938799	T/T	T/C	C/C	Affx-110084855	3D	138761117
AX-108939123	C/C	T/C	C/C	Affx-111544010	3D	525476942
AX-108951591	C/G	C/G	C/G	Affx-111719339	3D	420455318
AX-108954855	T/T	T/C	T/T	Affx-109672127	3D	168170961
AX-108958991	T/T	T/T	T/T	Affx-110972701	3D	313586377
AX-108977327	C/C	T/C	C/C	Affx-111289808	3D	60201189
AX-108981598	T/T	T/C	T/T	Affx-109115901	3D	225850549
AX-108984905	T/C	T/C	T/C	Affx-110613958	3D	5232771
AX-109001880	T/T	T/T	T/T	Affx-88789354	3D	453985895
AX-109074021	G/G	T/G	G/G	Affx-109390705	3D	205654761
AX-109094869	G/G	A/G	G/G	Affx-110845318	3D	235597637
AX-109135722	C/C	T/C	C/C	Affx-111654944	3D	330872440
AX-109163107	C/C	T/C	C/C	Affx-110382011	3D	250604319
AX-109164596	G/G	A/G	G/G	Affx-109252711	3D	449334823
AX-109179417	T/T	T/C	T/T	Affx-110948679	3D	128789755
AX-109190189	G/G	A/G	G/G	Affx-111348623	3D	217247970
AX-109191215	C/C	T/C	C/C	Affx-109183109	3D	221754382
AX-109252875	T/T	T/C	T/T	Affx-110303186	3D	235244297
AX-109260274	C/C	T/C	C/C	Affx-109383429	3D	360114932
AX-109271722	A/A	A/G	A/A	Affx-108910488	3D	600506761
AX-109272613	T/T	T/G	T/T	Affx-109049417	3D	445027315
AX-109273377	C/C	T/C	C/C	Affx-108941958	3D	254037102
AX-109278916	C/C	C/G	C/C	Affx-110269382	3D	332810364
AX-109280131	C/C	T/C	T/T	Affx-109906334	3D	595707535
AX-109281427	C/C	C/G	C/C	Affx-110333613	3D	510291511
AX-109282311	C/C	T/C	C/C	Affx-111663289	3D	36356349
AX-109284864	A/A	G/G	A/A	Affx-109482244	3D	433805595
AX-109290092	G/G	A/G	G/G	Affx-108981931	3D	329493015
AX-109303898	A/A	A/G	A/A	Affx-109468853	3D	508627455
AX-109306840	C/C	A/C	C/C	Affx-111179058	3D	366150994
AX-109314274	T/G	T/G	T/G	Affx-109077105	3D	226613746

AX-109317471	C/C	C/G	G/G	Affx-110459583	3D	129523657
AX-109321880	G/G	T/G	G/G	Affx-109783933	3D	230298369
AX-109324006	G/G	A/G	G/G	Affx-110595164	3D	39427536
AX-109324739	C/C	T/C	C/C	Affx-108932071	3D	334648506
AX-109332041	T/T	T/T	T/T	Affx-109818100	3D	598357875
AX-109332060	G/G	C/G	C/C	Affx-111696098	3D	136261654
AX-109334326	C/C	T/C	C/C	Affx-111765925	3D	379028266
AX-109340084	A/A	A/G	A/A	Affx-109356524	3D	567495210
AX-109340678	G/G	A/G	G/G	Affx-109303547	3D	190610905
AX-109340955	G/G	C/G	G/G	Affx-111047656	3D	359109904
AX-109341556	C/C	T/C	C/C	Affx-88551129	3D	221046278
AX-109347149	T/T	T/G	T/T	Affx-108948851	3D	554065442
AX-109354595	T/T	T/C	T/T	Affx-111340899	3D	459785395
AX-109354756	A/A	A/G	A/A	Affx-110166534	3D	355810081
AX-109355391	G/G	T/G	G/G	Affx-109266334	3D	313055523
AX-109366390	C/C	T/C	T/T	Affx-109262611	3D	605325583
AX-109376383	C/C	A/C	C/C	Affx-111890604	3D	614961323
AX-109379099	T/T	T/C	T/T	Affx-110061443	3D	310104819
AX-109383254	T/T	T/C	T/T	Affx-111916005	3D	165740593
AX-109383325	A/A	A/G	A/A	Affx-111218854	3D	306280291
AX-109383426	C/C	T/C	C/C	Affx-110271455	3D	416194807
AX-109388904	G/G	A/G	G/G	Affx-109933698	3D	509214141
AX-109392999	T/T	T/G	T/T	Affx-109274559	3D	236169118
AX-109394070	G/G	C/G	G/G	Affx-109309483	3D	595279092
AX-109394931	T/T	T/C	T/T	Affx-110110050	3D	246015647
AX-109395143	G/G	G/G	G/G	Affx-109646080	3D	53232885
AX-109395601	C/C	C/G	C/C	Affx-111055486	3D	607502469
AX-109399048	A/A	G/G	A/A	Affx-111596994	3D	603491986
AX-109399234	T/T	T/C	T/T	Affx-109203937	3D	272303735
AX-109402106	C/C	T/C	C/C	Affx-110690216	3D	517510756
AX-109421237	T/T	T/T	T/T	Affx-110500235	3D	42412616
AX-109427323	A/G	A/A	A/G	Affx-110752984	3D	555143613
AX-109429611	A/A	A/G	A/A	Affx-109691301	3D	240786059
AX-109430243	T/T	T/C	T/T	Affx-108917743	3D	328546232
AX-109430769	C/C	A/C	C/C	Affx-109711815	3D	515115245
AX-109452423	G/G	A/G	G/G	Affx-111515665	3D	514503117
AX-109453451	C/C	A/C	C/C	Affx-109649884	3D	471993990
AX-109457357	C/C	T/C	C/C	Affx-109125589	3D	602382062
AX-109459945	C/C	T/C	C/C	Affx-111477215	3D	21983774
AX-109466386	G/G	G/G	G/G	Affx-88679005	3D	180398232
AX-109471652	G/G	G/G	G/G	Affx-110791958	3D	268152476
AX-109474689	G/G	T/G	G/G	Affx-110626948	3D	225705453
AX-109477772	T/T	T/C	T/T	Affx-88629567	3D	220375362
AX-109500294	G/G	G/G	G/G	Affx-111235281	3D	599688383
AX-109505110	A/A	A/G	A/A	Affx-111291531	3D	278876619
AX-109509234	C/C	T/C	C/C	Affx-109556154	3D	563598345
AX-109512464	G/G	A/G	G/G	Affx-111855590	3D	590323433
AX-109516184	T/T	T/C	T/T	Affx-111145055	3D	74463281
AX-109517575	T/T	T/C	T/T	Affx-109477231	3D	208559092
AX-109537884	C/C	C/C	C/C	Affx-109829018	3D	120632091
AX-109550406	A/A	A/G	A/A	Affx-110611373	3D	288095946
AX-109580071	G/G	C/G	G/G	Affx-110426957	3D	477927929
AX-109626991	C/C	T/C	C/C	Affx-109307068	3D	224648576
AX-109656887	G/G	G/G	G/G	Affx-109293121	3D	294031863

AX-109660230	G/G	A/G	G/G	Affx-109043548	3D	112755007
AX-109688927	G/G	A/G	G/G	Affx-110495479	3D	32465081
AX-109730385	C/C	T/C	C/C	Affx-111605555	3D	452409102
AX-109735361	G/G	A/G	G/G	Affx-109766645	3D	291979439
AX-109743167	T/T	T/C	T/T	Affx-109008601	3D	178403033
AX-109744083	T/T	T/C	T/T	Affx-109379587	3D	317228145
AX-109744476	G/G	A/G	G/G	Affx-108920421	3D	603006147
AX-109746093	A/A	A/T	A/A	Affx-111289727	3D	7109785
AX-109783478	T/T	T/C	T/T	Affx-111434440	3D	289604746
AX-109790903	G/G	A/G	G/G	Affx-110585672	3D	287421733
AX-109791399	A/A	A/G	A/A	Affx-111880575	3D	113418590
AX-109799345	G/G	A/G	G/G	Affx-109833465	3D	519527578
AX-109820373	T/T	T/C	T/T	Affx-111801941	3D	280991305
AX-109826344	G/G	T/G	G/G	Affx-109855901	3D	486048911
AX-109829856	C/C	T/C	C/C	Affx-111387831	3D	362729567
AX-109831081	G/G	T/G	G/G	Affx-88731357	3D	342698100
AX-109838356	A/G	A/A	A/G	Affx-111376819	3D	578616909
AX-109841931	C/C	C/G	C/C	Affx-88704661	3D	515233348
AX-109842327	A/A	A/G	A/A	Affx-108855415	3D	82908502
AX-109852628	A/A	C/C	A/A	Affx-109318149	3D	291976983
AX-109859243	A/A	A/A	A/A	Affx-88728743	3D	338876849
AX-109861780	G/G	A/G	G/G	Affx-88621830	3D	613915672
AX-109878029	G/G	C/G	G/G	Affx-109559358	3D	21194487
AX-109879761	A/A	A/G	A/A	Affx-111594816	3D	104565286
AX-109888800	T/T	T/C	T/T	Affx-109998297	3D	405752708
AX-109889579	A/A	A/G	A/A	Affx-110935779	3D	234508367
AX-109901692	G/G	C/G	G/G	Affx-109268205	3D	589012130
AX-109903567	T/T	T/C	T/T	Affx-88603903	3D	196832500
AX-109909862	A/A	G/G	A/A	Affx-110416859	3D	108204161
AX-109913440	C/C	A/C	C/C	Affx-111473303	3D	302956138
AX-109914330	C/C	A/C	C/C	Affx-109041722	3D	320537077
AX-109914938	C/C	G/G	C/C	Affx-109853820	3D	64172442
AX-109920728	A/G	A/A	A/G	Affx-111907690	3D	290681086
AX-109923833	C/C	T/C	C/C	Affx-111023302	3D	266549227
AX-109928860	G/G	G/G	G/G	Affx-109721911	3D	396371815
AX-109929931	A/A	A/G	G/G	Affx-111481960	3D	136732204
AX-109933515	T/T	A/T	T/T	Affx-88608636	3D	16993556
AX-109934763	C/C	T/C	C/C	Affx-109549398	3D	559161485
AX-109948496	C/C	T/C	C/C	Affx-109518021	3D	576258807
AX-109954183	A/A	A/C	A/A	Affx-110586335	3D	297247421
AX-109954993	C/C	C/C	C/C	Affx-88584980	3D	241384973
AX-109961746	G/G	G/G	G/G	Affx-111873438	3D	387824727
AX-109967350	C/C	T/C	C/C	Affx-109666437	3D	86468028
AX-109968883	T/C	T/C	T/C	Affx-88516147	3D	11983894
AX-109972844	C/C	C/G	C/C	Affx-109615128	3D	604006564
AX-109976094	G/G	A/G	G/G	Affx-111256793	3D	204178154
AX-109980905	G/G	A/G	G/G	Affx-110244795	3D	517896526
AX-109988401	G/G	A/G	G/G	Affx-111765274	3D	395857441
AX-109996937	G/G	A/G	G/G	Affx-111997630	3D	250020125
AX-109998069	C/C	C/G	C/C	Affx-110703516	3D	438515754
AX-110015572	C/C	A/C	A/C	Affx-109382763	3D	249305505
AX-110029250	T/T	T/C	T/T	Affx-111859613	3D	312753243
AX-110032043	G/G	A/G	G/G	Affx-109319932	3D	247584116
AX-110056468	G/G	A/G	G/G	Affx-111103588	3D	349681443

AX-110065383	C/C	T/C	C/C	Affx-109876895	3D	260889407
AX-110192563	G/G	G/G	G/G	Affx-110570605	3D	31708112
AX-110197158	G/G	A/G	G/G	Affx-111456780	3D	317908975
AX-110214968	A/A	A/G	A/A	Affx-110214863	3D	130247863
AX-110215504	G/G	G/G	G/G	Affx-110342705	3D	94554126
AX-110234451	G/G	G/G	G/G	Affx-109175958	3D	57095551
AX-110237963	A/A	A/G	A/A	Affx-110797322	3D	102119028
AX-110246370	C/C	T/C	C/C	Affx-111132699	3D	111622698
AX-110269943	C/C	A/C	C/C	Affx-111569787	3D	222364369
AX-110270468	G/G	A/G	G/G	Affx-109540411	3D	75351635
AX-110282866	G/G	A/G	G/G	Affx-111612908	3D	497955725
AX-110284733	G/G	T/G	G/G	Affx-88660012	3D	414838372
AX-110288100	T/T	T/C	T/T	Affx-111126667	3D	311850375
AX-110292863	A/A	A/C	A/A	Affx-110571004	3D	132643681
AX-110336377	A/A	G/G	A/A	Affx-88587391	3D	132645888
AX-110363017	T/T	T/G	T/T	Affx-110979403	3D	165411038
AX-110365670	C/C	T/C	C/C	Affx-109000195	3D	610791016
AX-110365829	C/C	T/C	C/C	Affx-88777389	3D	119573204
AX-110372611	G/G	A/G	G/G	Affx-109557959	3D	105100029
AX-110372649	C/C	T/C	C/C	Affx-110575906	3D	414216471
AX-110372784	G/G	C/G	G/G	Affx-88406695	3D	155952359
AX-110376389	G/G	T/G	G/G	Affx-111288660	3D	59260838
AX-110381382	G/G	T/G	G/G	Affx-111998114	3D	325968497
AX-110381932	A/A	A/G	A/A	Affx-88804926	3D	294604232
AX-110385725	C/G	C/G	C/G	Affx-109007519	3D	907807
AX-110387704	T/T	C/C	T/T	Affx-109008563	3D	375409843
AX-110390723	C/C	C/C	C/C	Affx-111063835	3D	523815326
AX-110391699	C/C	T/C	C/C	Affx-110779703	3D	13750613
AX-110394178	C/C	C/G	C/C	Affx-111653362	3D	85063959
AX-110398405	T/T	T/C	T/T	Affx-110525261	3D	369421813
AX-110398452	A/A	A/G	A/A	Affx-110555316	3D	575092674
AX-110398758	C/C	T/C	C/C	Affx-88783128	3D	17368043
AX-110408939	C/C	T/C	C/C	Affx-110973845	3D	612317068
AX-110409135	C/C	T/C	C/C	Affx-110667197	3D	459093703
AX-110423300	G/G	T/G	G/G	Affx-111474309	3D	9449947
AX-110429684	G/G	T/G	G/G	Affx-110949516	3D	9097502
AX-110430652	G/G	A/G	G/G	Affx-111039270	3D	24790901
AX-110432825	G/G	A/G	G/G	Affx-110244643	3D	206655789
AX-110434962	T/T	T/C	T/T	Affx-88480120	3D	199370252
AX-110441918	G/G	A/G	G/G	Affx-110713864	3D	269201395
AX-110449684	G/G	T/G	G/G	Affx-110947049	3D	304509428
AX-110450605	A/A	A/G	G/G	Affx-110649009	3D	137627794
AX-110464489	C/C	T/C	C/C	Affx-110311667	3D	82139636
AX-110474025	C/C	T/C	C/C	Affx-111747703	3D	303722186
AX-110489977	A/A	A/A	A/A	Affx-88784091	3D	381001215
AX-110493936	G/G	A/G	G/G	Affx-109442319	3D	286781572
AX-110495749	T/T	T/C	T/T	Affx-111506952	3D	520819564
AX-110511459	T/T	T/C	T/T	Affx-110852220	3D	308340125
AX-110515593	A/A	A/G	A/A	Affx-109060623	3D	572120371
AX-110520367	G/G	A/G	G/G	Affx-110410493	3D	223988713
AX-110520414	T/T	T/C	T/T	Affx-111949043	3D	358892681
AX-110529732	G/G	A/G	G/G	Affx-111385240	3D	147425228
AX-110533567	A/A	A/G	A/A	Affx-111540330	3D	267248313
AX-110536745	A/A	A/G	A/G	Affx-88359503	3D	592622390

AX-110538357	A/A	A/G	A/A	Affx-109797244	3D	107923806
AX-110542392	C/C	T/C	C/C	Affx-111204419	3D	250887501
AX-110549944	C/C	A/C	C/C	Affx-110681152	3D	355419765
AX-110555702	C/C	A/C	C/C	Affx-109297702	3D	500667578
AX-110564748	A/A	A/G	A/A	Affx-111105442	3D	564533236
AX-110564913	G/G	T/G	G/G	Affx-110412539	3D	314697452
AX-110568568	T/T	T/G	T/T	Affx-110915814	3D	573285369
AX-110577697	G/G	A/G	G/G	Affx-109407332	3D	201622398
AX-110586439	T/T	T/G	T/T	Affx-111956258	3D	572553115
AX-110596103	C/C	T/C	C/C	Affx-110618900	3D	265297022
AX-110599821	A/A	A/A	A/A	Affx-110836731	3D	472449022
AX-110611865	A/A	A/G	A/A	Affx-109762222	3D	607161921
AX-110612358	T/T	T/C	T/T	Affx-108923041	3D	140733879
AX-110682765	G/G	A/G	G/G	Affx-109785702	3D	602059776
AX-110752656	T/T	T/C	T/T	Affx-110960962	3D	333013774
AX-110769382	G/G	A/G	G/G	Affx-111948835	3D	110595449
AX-110772653	A/A	A/T	A/A	Affx-111191532	3D	570154362
AX-110815872	G/G	A/G	G/G	Affx-88358621	3D	508554627
AX-110824500	T/T	T/G	T/T	Affx-111567560	3D	95794159
AX-110826498	A/A	A/G	A/A	Affx-109443399	3D	358630080
AX-110826955	T/T	T/C	T/T	Affx-109792960	3D	173719518
AX-110827948	A/A	A/G	A/A	Affx-110638152	3D	328521770
AX-110830021	C/C	A/C	C/C	Affx-109832844	3D	260816674
AX-110857044	T/T	T/T	T/T	Affx-111634537	3D	141027488
AX-110857561	C/C	T/C	C/C	Affx-109951796	3D	335291006
AX-110877291	C/C	T/C	C/C	Affx-109706824	3D	174688273
AX-110890657	C/C	T/C	C/C	Affx-109873934	3D	257547008
AX-110894943	G/G	C/G	G/G	Affx-109601710	3D	320767636
AX-110913237	T/T	T/C	T/T	Affx-110634344	3D	273001033
AX-110915537	A/A	A/G	A/A	Affx-111770962	3D	281172931
AX-110923174	G/G	G/G	G/G	Affx-111910607	3D	502426653
AX-110928411	T/T	T/C	T/T	Affx-109135844	3D	37736961
AX-110928853	C/C	C/G	C/C	Affx-88744194	3D	292886123
AX-110929772	G/G	A/G	G/G	Affx-109720654	3D	197222874
AX-110931772	T/T	T/T	T/T	Affx-111533688	3D	380974058
AX-110933776	A/C	A/C	A/C	Affx-110213790	3D	441646439
AX-110934345	A/A	A/G	A/A	Affx-111934563	3D	371908988
AX-110944192	A/A	A/G	A/A	Affx-110269154	3D	244908012
AX-110945027	G/G	A/G	G/G	Affx-111154591	3D	246620451
AX-110947845	C/C	T/C	C/C	Affx-110526113	3D	591711544
AX-110958097	A/A	A/C	A/A	Affx-88705289	3D	310718976
AX-110958532	C/C	C/C	C/C	Affx-111900707	3D	393537308
AX-110959583	C/C	T/C	C/C	Affx-109611289	3D	278280746
AX-110960738	G/G	A/G	G/G	Affx-111910844	3D	302054569
AX-110963247	A/A	A/G	A/A	Affx-111614805	3D	567829932
AX-110965214	G/G	A/A	G/G	Affx-110683091	3D	239555296
AX-110974000	T/T	T/C	T/T	Affx-111389338	3D	20553876
AX-110977729	G/G	A/G	G/G	Affx-111051484	3D	6413892
AX-110986033	C/C	A/C	C/C	Affx-111229530	3D	324382922
AX-111006048	A/A	A/G	A/A	Affx-110436630	3D	270143417
AX-111009818	T/T	T/C	T/T	Affx-110292792	3D	427234678
AX-111017855	A/A	A/T	A/A	Affx-109624089	3D	514427265
AX-111018209	A/A	A/G	A/A	Affx-110034587	3D	553594460
AX-111027124	G/G	A/G	G/G	Affx-111732687	3D	400781396

AX-111032615	T/C	C/C	T/C	Affx-111131174	3D	360336447
AX-111045512	G/G	A/G	G/G	Affx-110200620	3D	504958825
AX-111054120	G/G	C/G	G/G	Affx-111204067	3D	6179080
AX-111054517	T/T	A/T	T/T	Affx-111861240	3D	180840097
AX-111058247	G/G	A/G	G/G	Affx-110792767	3D	25295435
AX-111064749	G/G	G/G	G/G	Affx-110735840	3D	248216457
AX-111064903	A/A	A/A	A/A	Affx-111887936	3D	124002154
AX-111068370	C/C	C/G	G/G	Affx-109420035	3D	606079108
AX-111069941	T/T	T/C	T/T	Affx-108915429	3D	557233365
AX-111072391	C/C	C/G	C/C	Affx-108964897	3D	600563327
AX-111073783	C/C	C/G	C/C	Affx-109693819	3D	1316117
AX-111080889	T/T	T/G	T/T	Affx-111036866	3D	47497260
AX-111087309	C/C	T/C	C/C	Affx-108977390	3D	56675266
AX-111087665	T/T	T/C	T/T	Affx-109623117	3D	454756313
AX-111096826	G/G	G/G	G/G	Affx-109246860	3D	311490328
AX-111099273	T/T	T/C	T/T	Affx-109383431	3D	182645267
AX-111109273	T/T	T/G	T/T	Affx-110561003	3D	537495536
AX-111113250	C/C	T/C	C/C	Affx-110875988	3D	233559884
AX-111126228	A/A	A/C	A/A	Affx-110344305	3D	586628845
AX-111131164	A/A	G/G	A/A	Affx-111344743	3D	195149719
AX-111131844	G/G	A/G	G/G	Affx-110652068	3D	306268290
AX-111134749	C/C	C/G	C/C	Affx-109699672	3D	273702089
AX-111147649	T/T	T/C	T/T	Affx-111076672	3D	274822017
AX-111149329	T/T	C/C	T/T	Affx-108958998	3D	2369603
AX-111156965	C/C	C/C	C/C	Affx-111311025	3D	569810636
AX-111162584	C/C	T/C	T/T	Affx-109078820	3D	605070947
AX-111212045	A/A	A/G	A/A	Affx-111010162	3D	552874646
AX-111247757	C/C	T/C	C/C	Affx-110696089	3D	355745657
AX-111258512	C/C	T/C	C/C	Affx-108965751	3D	608763229
AX-111314189	G/G	A/G	G/G	Affx-111402814	3D	411614162
AX-111334624	G/G	A/G	G/G	Affx-109206264	3D	451690279
AX-111337684	G/G	G/G	G/G	Affx-109129741	3D	42304664
AX-111344580	A/A	A/C	A/A	Affx-110195437	3D	327523526
AX-111362517	A/A	A/A	A/A	Affx-111118882	3D	179182860
AX-111379101	C/C	T/C	C/C	Affx-111171106	3D	123936212
AX-111379661	G/G	T/G	G/G	Affx-110036628	3D	193808840
AX-111384640	T/C	T/C	T/C	Affx-110130694	3D	180845126
AX-111408899	T/T	T/C	T/T	Affx-109786316	3D	206984620
AX-111410277	T/T	T/C	T/T	Affx-110996114	3D	370015638
AX-111437884	C/C	T/C	C/C	Affx-111068364	3D	211043283
AX-111456724	C/C	T/C	C/C	Affx-111573399	3D	574348173
AX-111473752	C/C	C/G	C/C	Affx-88617878	3D	611829994
AX-111479535	C/C	T/C	C/C	Affx-109253727	3D	578749369
AX-111481627	C/C	T/C	T/T	Affx-111716684	3D	606612639
AX-111489545	G/G	T/G	G/G	Affx-110067485	3D	361774219
AX-111501818	A/G	G/G	A/G	Affx-109008787	3D	35045070
AX-111504450	T/T	T/C	T/T	Affx-109893399	3D	212626573
AX-111507826	C/C	T/C	C/C	Affx-111600207	3D	298513548
AX-111510604	G/G	A/G	G/G	Affx-109505289	3D	198093300
AX-111526651	T/T	T/C	T/T	Affx-111703261	3D	568434417
AX-111528152	C/C	T/C	C/C	Affx-111328997	3D	155603467
AX-111532282	A/A	A/G	A/A	Affx-109337676	3D	519334124
AX-111543321	G/G	A/G	G/G	Affx-110478704	3D	326082748
AX-111556201	A/A	A/A	A/A	Affx-110446557	3D	96397684

AX-111560917	A/A	A/G	A/A	Affx-111777442	3D	382982278
AX-111564705	T/T	T/C	T/T	Affx-109509420	3D	241726550
AX-111567425	A/T	T/T	A/T	Affx-111911716	3D	352371395
AX-111589572	C/C	T/C	C/C	Affx-109926117	3D	367224093
AX-111591851	A/A	A/A	A/A	Affx-110137357	3D	153856651
AX-111593417	G/G	T/G	G/G	Affx-111028154	3D	501056082
AX-111600316	C/C	T/C	C/C	Affx-109555270	3D	391646817
AX-111617121	C/C	T/T	C/C	Affx-109808134	3D	587046321
AX-111624595	T/T	T/C	T/T	Affx-88603031	3D	572830191
AX-111629109	G/G	T/G	G/G	Affx-111527735	3D	355416408
AX-111644320	A/A	A/G	A/A	Affx-111166529	3D	30763681
AX-111644531	C/C	T/C	C/C	Affx-111754876	3D	97400667
AX-111654550	T/T	T/C	T/T	Affx-108849675	3D	257929333
AX-111666894	A/A	A/C	C/C	Affx-109805953	3D	130341908
AX-111675563	A/A	A/G	A/A	Affx-111040846	3D	184510481
AX-111686153	A/A	A/G	A/A	Affx-111255436	3D	548549709
AX-111689571	A/A	A/G	A/A	Affx-110330408	3D	274984684
AX-111699502	T/T	T/C	T/T	Affx-109122946	3D	335608888
AX-111703658	C/C	C/C	C/C	Affx-110863128	3D	32268623
AX-111713559	G/G	C/G	G/G	Affx-109460997	3D	609216655
AX-111760688	C/C	T/C	C/C	Affx-110014446	3D	566145912
AX-111762331	G/G	A/G	G/G	Affx-110833492	3D	185798059
AX-111778716	G/G	A/G	G/G	Affx-111257420	3D	202665077
AX-111789343	G/G	C/G	G/G	Affx-109601958	3D	7573610
AX-111801578	C/C	A/C	C/C	Affx-110336687	3D	564700009
AX-111802381	G/G	A/G	A/A	Affx-110198574	3D	596163294
AX-111838222	A/A	A/G	A/A	Affx-110351044	3D	383963360
AX-111856034	C/C	T/C	C/C	Affx-110888237	3D	304695607
AX-111891650	C/C	G/G	G/G	Affx-109809881	3D	134822499
AX-111902423	C/C	C/G	C/C	Affx-109580084	3D	474581581
AX-111916664	G/G	A/G	G/G	Affx-109970885	3D	494644813
AX-111921159	G/G	A/G	G/G	Affx-109172852	3D	300938385
AX-111922285	C/C	T/C	C/C	Affx-110967055	3D	386803983
AX-111934532	C/C	T/C	C/C	Affx-110620980	3D	315488357
AX-112288490	A/A	G/G	A/A	Affx-92491473	3D	474433545
AX-179558692	A/A	A/G	A/A	Affx-112313111	3D	456632768
AX-182024349	T/T	T/C	T/T	Affx-110709245	3D	1119618
AX-182061318	A/A	A/A	A/A	Affx-472306471	3D	1346661
AX-182062780	T/T	T/C	T/T	Affx-472308653	3D	3301484
AX-182069952	A/A	A/A	A/A	Affx-472319707	3D	593762055
AX-182084286	T/G	T/G	T/G	Affx-92363707	3D	355887538
AX-182114415	A/C	C/C	A/C	Affx-111244215	3D	1113885
AX-182145297	G/G	A/G	G/G	Affx-472293081	3D	388331190
AX-86171339	C/C	T/C	C/C	Affx-112314367	3D	320431186
AX-89337262	G/G	A/G	G/G	Affx-88368760	3D	495579864
AX-89402731	A/A	A/G	A/A	Affx-88435277	3D	390036286
AX-89444624	C/C	C/G	C/C	Affx-88477373	3D	122017758
AX-89529257	T/T	A/T	T/T	Affx-88562202	3D	405819459
AX-89547630	C/C	T/C	T/T	Affx-88580647	3D	594062466
AX-89562145	T/T	A/T	A/A	Affx-88595200	3D	14276999
AX-89574305	A/A	A/G	A/A	Affx-88607384	3D	27877072
AX-89576720	C/C	C/C	C/C	Affx-88609803	3D	481570980
AX-89577616	T/T	T/C	T/T	Affx-88610699	3D	355222731
AX-89616590	T/T	T/C	T/T	Affx-88649781	3D	584947318

AX-89683800	G/G	A/G	G/G	Affx-88716556	3D	474455590
AX-89699893	A/A	A/C	A/A	Affx-88732628	3D	515930886
AX-89725997	T/T	T/T	T/T	Affx-88758691	3D	535431090
AX-94396808	G/G	C/G	G/G	Affx-92374139	3D	7029149
AX-94410643	G/G	C/C	G/G	Affx-92732930	3D	39336014
AX-94427000	C/C	C/C	C/C	Affx-92761215	3D	355705864
AX-94434095	G/G	A/G	G/G	Affx-92566167	3D	391262590
AX-94436946	A/A	A/G	A/A	Affx-92634360	3D	599384795
AX-94469025	A/A	A/G	A/A	Affx-92314892	3D	22680085
AX-94474428	T/T	T/T	T/T	Affx-92747283	3D	430151785
AX-94528475	C/C	T/C	C/C	Affx-92444702	3D	536029042
AX-94568016	A/A	A/A	A/A	Affx-92312426	3D	561020396
AX-94584272	A/A	A/G	A/A	Affx-88515782	3D	3299022
AX-94596190	A/C	A/A	A/C	Affx-92092052	3D	355891129
AX-94604542	G/G	A/A	G/G	Affx-92835250	3D	75357976
AX-94612981	T/C	T/C	T/C	Affx-92135320	3D	567148746
AX-94615465	C/C	A/A	C/C	Affx-92276569	3D	7028308
AX-94620485	T/C	T/T	T/C	Affx-92637238	3D	3298609
AX-94773542	A/G	G/G	A/G	Affx-92851925	3D	7028396
AX-94813847	A/A	A/G	A/A	Affx-92771119	3D	456909217
AX-94814857	T/T	C/C	T/T	Affx-92888342	3D	7028462
AX-94831084	G/G	A/G	G/G	Affx-92303275	3D	469487113
AX-94843098	C/C	A/A	A/C	Affx-92547800	3D	64475691
AX-94881820	T/C	T/C	T/T	Affx-92382699	3D	554776932
AX-94900846	A/G	A/A	A/G	Affx-92566283	3D	66174780
AX-94929224	T/C	C/C	T/C	Affx-92834802	3D	68169516
AX-94970705	C/C	T/T	C/C	Affx-92276070	3D	7028769
AX-95008504	C/C	C/G	C/C	Affx-92471739	3D	151440459
AX-95015595	T/T	T/C	T/T	Affx-92117743	3D	7028669
AX-95018922	A/A	A/A	A/A	Affx-92177097	3D	7028555
AX-95019827	C/C	C/C	C/C	Affx-92100912	3D	355705747
AX-95093952	T/T	T/C	T/T	Affx-92690369	3D	168578218
AX-95216372	G/G	A/G	G/G	Affx-92693403	3D	351288448
AX-95237651	T/T	T/C	T/T	Affx-92236427	3D	7028940
AX-95245565	G/G	G/G	G/G	Affx-92167177	3D	16055389
AX-95259287	T/T	T/C	T/T	Affx-92387748	3D	29883056
AX-95659137	C/C	T/C	C/C	Affx-109837951	3D	547625537
AX-108726336	C/C	T/C	C/C	Affx-108933185	4A	628647529
AX-108728455	G/G	G/G	G/G	Affx-111325659	4A	723754064
AX-108730686	G/G	A/G	G/G	Affx-111578798	4A	461649762
AX-108732542	A/A	C/C	A/A	Affx-88789640	4A	75340640
AX-108733323	A/A	A/G	A/A	Affx-109831075	4A	297261385
AX-108734424	C/C	T/C	C/C	Affx-111555365	4A	375775511
AX-108751023	T/T	T/C	T/T	Affx-110428240	4A	60921930
AX-108753429	C/C	T/T	T/T	Affx-110524699	4A	656036868
AX-108756264	A/A	A/T	T/T	Affx-111334766	4A	721372477
AX-108756659	G/G	A/G	G/G	Affx-110553297	4A	1688925
AX-108759403	A/G	A/G	G/G	Affx-111280630	4A	601738568
AX-108761059	A/A	G/G	A/A	Affx-109318846	4A	117533026
AX-108762339	G/G	T/G	T/T	Affx-109644629	4A	698328871
AX-108763086	T/T	T/C	T/T	Affx-108870179	4A	570776310
AX-108765395	C/C	T/C	C/C	Affx-111202039	4A	6525884
AX-108765827	G/G	G/G	G/G	Affx-111038199	4A	463491357
AX-108769971	T/T	T/G	T/T	Affx-111457956	4A	347426354

AX-108775849	G/G	C/G	C/C	Affx-110923294	4A	660922081
AX-108777378	T/T	T/C	T/T	Affx-109051694	4A	224270372
AX-108777965	C/C	A/C	A/A	Affx-109088106	4A	605767873
AX-108778812	G/G	C/G	G/G	Affx-109361268	4A	434034619
AX-108784859	G/G	C/G	G/G	Affx-111433062	4A	468750775
AX-108786421	C/C	A/C	C/C	Affx-111826879	4A	466567543
AX-108787220	G/G	C/G	C/C	Affx-109604740	4A	651348161
AX-108791064	A/A	A/G	A/A	Affx-110302505	4A	121149583
AX-108791065	G/G	T/G	G/G	Affx-109614190	4A	439005977
AX-108800004	G/G	A/G	G/G	Affx-109044099	4A	172952173
AX-108800547	T/T	T/G	T/T	Affx-110373443	4A	230005172
AX-108801740	A/A	A/G	A/A	Affx-109002853	4A	207961798
AX-108807126	C/C	T/T	T/T	Affx-109101058	4A	662711385
AX-108810926	A/A	A/G	A/A	Affx-111342428	4A	349919216
AX-108818424	C/C	C/C	C/C	Affx-109548387	4A	739062775
AX-108819266	T/T	T/C	T/T	Affx-108914622	4A	88259542
AX-108822859	C/C	C/G	C/C	Affx-110825096	4A	459243902
AX-108824978	C/C	C/C	C/C	Affx-110701132	4A	191189140
AX-108826643	C/C	T/C	C/C	Affx-110244773	4A	504192371
AX-108827229	A/A	A/C	A/A	Affx-110024543	4A	219799342
AX-108831193	A/A	A/T	A/A	Affx-109394003	4A	6921676
AX-108835358	T/C	T/T	T/C	Affx-111236313	4A	20247616
AX-108843686	A/A	A/G	A/A	Affx-110847769	4A	198230666
AX-108844812	T/T	A/T	A/A	Affx-109504161	4A	697727582
AX-108844977	A/A	A/A	A/A	Affx-110567045	4A	388784621
AX-108845537	C/C	A/C	C/C	Affx-111045811	4A	257351928
AX-108852119	A/A	A/C	A/A	Affx-111691709	4A	390455289
AX-108858024	G/G	A/G	G/G	Affx-110540017	4A	237687734
AX-108861243	T/T	T/C	T/T	Affx-111329184	4A	255163375
AX-108864812	T/T	T/C	T/T	Affx-109147805	4A	366643698
AX-108866087	C/C	A/C	C/C	Affx-109442395	4A	690415394
AX-108870577	C/C	T/T	C/C	Affx-109190009	4A	500119543
AX-108870819	C/C	T/C	C/C	Affx-111708673	4A	466153131
AX-108875018	C/C	C/C	C/C	Affx-111422917	4A	87404384
AX-108887341	G/G	T/G	G/G	Affx-109843153	4A	567213012
AX-108889870	C/C	T/C	C/C	Affx-111431762	4A	41851073
AX-108891522	T/T	T/C	T/T	Affx-109033962	4A	347884168
AX-108892689	A/A	A/G	A/A	Affx-110435063	4A	236087580
AX-108893017	G/G	C/G	G/G	Affx-110671701	4A	209022700
AX-108893379	C/C	T/C	C/C	Affx-111767008	4A	228762273
AX-108896162	A/A	A/G	A/A	Affx-110893716	4A	195228852
AX-108897689	T/T	T/C	T/T	Affx-108881700	4A	306918700
AX-108899858	A/A	A/G	A/A	Affx-109005361	4A	582517876
AX-108901881	T/T	T/C	T/T	Affx-109568530	4A	254047787
AX-108902677	G/G	G/G	G/G	Affx-110855605	4A	367657564
AX-108904981	T/T	T/C	T/T	Affx-110323908	4A	393329272
AX-108905356	C/C	C/G	G/G	Affx-88451265	4A	706497864
AX-108907490	A/A	A/G	G/G	Affx-110482380	4A	616997960
AX-108908317	G/G	A/G	G/G	Affx-110321677	4A	681180902
AX-108915470	G/G	T/G	T/T	Affx-110750438	4A	650860925
AX-108917261	T/T	T/C	T/T	Affx-109931803	4A	600798863
AX-108928080	C/C	C/C	C/C	Affx-109819753	4A	535306463
AX-108929552	T/T	A/A	A/A	Affx-110454525	4A	658857490
AX-108937128	C/C	T/C	C/C	Affx-111555620	4A	227361546

AX-108941938	G/G	G/G	G/G	Affx-109898861	4A	622565277
AX-108942399	A/A	A/G	A/A	Affx-110819493	4A	618017608
AX-108945836	G/G	A/G	G/G	Affx-109772435	4A	193097707
AX-108946086	C/C	C/C	C/C	Affx-110479934	4A	43236449
AX-108948523	T/C	T/C	C/C	Affx-109508916	4A	666134124
AX-108949287	G/G	C/G	G/G	Affx-110348872	4A	686173880
AX-108950088	T/T	T/G	T/T	Affx-111260521	4A	200222793
AX-108954112	A/A	A/G	A/A	Affx-110324270	4A	29789708
AX-108958423	A/A	A/G	A/A	Affx-110520700	4A	193626324
AX-108959104	C/C	C/C	C/C	Affx-110501342	4A	617121150
AX-108962379	T/T	T/C	T/T	Affx-111113755	4A	222625075
AX-108962808	G/G	G/G	G/G	Affx-88526356	4A	541338660
AX-108966946	G/G	G/G	G/G	Affx-109902427	4A	679986545
AX-108969300	C/C	T/C	C/C	Affx-110762918	4A	125356184
AX-108970642	A/A	A/G	A/A	Affx-110432924	4A	427495528
AX-108973520	G/G	A/A	G/G	Affx-110801445	4A	591069873
AX-108978044	A/A	A/C	A/A	Affx-111056929	4A	366571410
AX-108995460	T/T	T/C	T/T	Affx-110036233	4A	138169822
AX-108995509	G/G	A/G	G/G	Affx-109104345	4A	108747433
AX-109012904	C/C	T/C	C/C	Affx-88771387	4A	120607938
AX-109031111	C/C	T/C	C/C	Affx-110026487	4A	592217074
AX-109033949	G/G	A/G	G/G	Affx-111385688	4A	360635039
AX-109034391	A/A	A/G	A/A	Affx-109361385	4A	245959001
AX-109035440	T/T	T/C	T/T	Affx-110501151	4A	560004466
AX-109046234	T/T	T/C	T/T	Affx-110769396	4A	323173734
AX-109046794	A/A	A/C	A/A	Affx-110713841	4A	407820648
AX-109061611	T/T	T/C	T/T	Affx-110737383	4A	351181284
AX-109063836	C/C	T/C	C/C	Affx-109073977	4A	314707804
AX-109064955	A/A	A/G	A/A	Affx-109615904	4A	324352032
AX-109066119	A/A	A/G	A/A	Affx-111190935	4A	200400738
AX-109066809	G/G	T/G	T/G	Affx-109441492	4A	179941606
AX-109068706	T/T	T/C	T/T	Affx-110020160	4A	394999396
AX-109078019	T/T	T/C	T/T	Affx-109142974	4A	258632382
AX-109078060	T/T	T/C	T/T	Affx-109401392	4A	214432066
AX-109078940	C/C	A/C	C/C	Affx-110051565	4A	321565249
AX-109090565	G/G	C/G	G/G	Affx-111873864	4A	298352750
AX-109096175	T/T	T/C	T/T	Affx-109921222	4A	355221059
AX-109097939	A/A	A/A	A/A	Affx-111754292	4A	184956911
AX-109270432	T/T	T/C	T/T	Affx-109235800	4A	546376158
AX-109281192	G/G	C/G	G/G	Affx-110904304	4A	82399805
AX-109284677	C/C	T/C	C/C	Affx-110506214	4A	598794578
AX-109288734	T/T	T/C	T/T	Affx-111366928	4A	241964245
AX-109296730	C/C	A/C	C/C	Affx-88562331	4A	68155791
AX-109301139	G/G	A/G	G/G	Affx-110167069	4A	537551725
AX-109302588	T/C	T/C	T/T	Affx-111615412	4A	689834479
AX-109303147	G/G	T/G	G/G	Affx-109965135	4A	556090153
AX-109303277	C/C	C/G	C/C	Affx-109989425	4A	83721635
AX-109305480	G/G	A/G	G/G	Affx-110551334	4A	667064769
AX-109307896	T/T	T/C	T/T	Affx-111261076	4A	484860570
AX-109316763	G/G	G/G	G/G	Affx-109439675	4A	626964421
AX-109320243	C/C	C/C	C/C	Affx-108861329	4A	731337851
AX-109321866	C/C	T/C	C/C	Affx-109922350	4A	377184186
AX-109329316	C/C	T/C	C/C	Affx-111834514	4A	21222835
AX-109330895	A/A	A/C	A/A	Affx-110247574	4A	112794824

AX-109335943	G/G	A/G	G/G	Affx-109858467	4A	164722729
AX-109343088	A/A	A/G	A/A	Affx-111303968	4A	223797711
AX-109353504	G/G	C/G	G/G	Affx-110755542	4A	647278920
AX-109354657	A/A	A/G	A/A	Affx-110376125	4A	607895298
AX-109354728	T/T	T/C	T/T	Affx-109328265	4A	111268266
AX-109355314	C/C	T/C	C/C	Affx-110865110	4A	202580524
AX-109357717	A/A	A/G	A/A	Affx-111402276	4A	274739802
AX-109357719	G/G	A/G	G/G	Affx-110983987	4A	238292732
AX-109375057	A/A	G/G	A/A	Affx-108996291	4A	633308367
AX-109375154	A/A	A/G	A/A	Affx-110767771	4A	36077013
AX-109378399	A/A	A/G	A/A	Affx-111841310	4A	129442800
AX-109378449	A/A	A/G	A/A	Affx-110206521	4A	565712501
AX-109379729	C/C	T/C	C/C	Affx-109289673	4A	415622605
AX-109381636	C/C	T/C	C/C	Affx-110005692	4A	428355624
AX-109385165	C/C	T/C	C/C	Affx-88752167	4A	375603552
AX-109385715	A/A	A/G	A/A	Affx-111635988	4A	365203816
AX-109387432	T/T	T/T	T/T	Affx-109627251	4A	456131339
AX-109391536	A/A	A/C	A/A	Affx-108999363	4A	584425606
AX-109398960	G/G	A/G	G/G	Affx-110545833	4A	508987930
AX-109410055	G/G	A/G	G/G	Affx-110532059	4A	238189813
AX-109411075	T/T	T/T	T/T	Affx-111315043	4A	201142650
AX-109411376	C/C	C/G	C/C	Affx-109033912	4A	75340592
AX-109416582	C/C	T/C	C/C	Affx-109562099	4A	668471682
AX-109417418	G/G	A/G	G/G	Affx-111557391	4A	134166568
AX-109417674	G/G	A/G	G/G	Affx-111885755	4A	21487929
AX-109418813	G/G	A/G	G/G	Affx-88634064	4A	122248852
AX-109420461	A/A	A/G	A/A	Affx-109855802	4A	130045834
AX-109424742	C/C	C/G	C/C	Affx-110939848	4A	578009366
AX-109431844	G/G	A/G	G/G	Affx-109011312	4A	688095701
AX-109441116	G/G	A/G	G/G	Affx-109326283	4A	448753319
AX-109443083	G/G	A/G	G/G	Affx-88737282	4A	627995201
AX-109444347	A/A	A/G	A/A	Affx-111734429	4A	256714635
AX-109445204	A/A	A/G	A/A	Affx-110797574	4A	449503304
AX-109445989	A/A	A/A	A/A	Affx-111049976	4A	249653742
AX-109453226	A/A	A/C	A/A	Affx-111903337	4A	189934998
AX-109459438	T/T	T/C	T/T	Affx-109596799	4A	384709102
AX-109460468	A/A	G/G	G/G	Affx-109315534	4A	735301568
AX-109462029	G/G	T/G	G/G	Affx-110089259	4A	437846087
AX-109462231	A/A	A/G	A/A	Affx-111976526	4A	244569162
AX-109465075	G/G	A/G	G/G	Affx-108937540	4A	206684372
AX-109466596	A/A	A/G	A/A	Affx-109772697	4A	199618717
AX-109469915	A/A	A/G	A/A	Affx-109884990	4A	607240777
AX-109474997	C/C	T/C	C/C	Affx-109114049	4A	572876165
AX-109478972	C/C	T/C	C/C	Affx-110604095	4A	238870410
AX-109485039	G/G	A/G	G/G	Affx-109728222	4A	595191246
AX-109491044	A/A	A/G	A/A	Affx-111704875	4A	28496969
AX-109498140	A/A	A/T	A/A	Affx-109777229	4A	195796612
AX-109500638	A/A	A/G	A/A	Affx-109996886	4A	483900334
AX-109501519	A/A	A/C	A/A	Affx-109603951	4A	229478509
AX-109501673	A/A	A/G	A/A	Affx-109413914	4A	297970326
AX-109506032	T/T	T/C	T/T	Affx-111164998	4A	108192759
AX-109509506	G/G	A/G	G/G	Affx-88502860	4A	488867412
AX-109516552	T/T	T/C	T/T	Affx-111754637	4A	378880979
AX-109519189	T/T	T/C	C/C	Affx-110062647	4A	668099094

AX-109519591	T/T	T/C	T/T	Affx-110116300	4A	402648216
AX-109522359	G/G	A/G	G/G	Affx-109367205	4A	171063659
AX-109522614	A/A	A/G	A/A	Affx-109886761	4A	198399014
AX-109523054	G/G	A/G	G/G	Affx-110473174	4A	190830097
AX-109527362	A/A	A/G	A/A	Affx-111173309	4A	467258017
AX-109531016	T/T	T/C	T/T	Affx-111384979	4A	575803839
AX-109533701	C/C	C/G	C/C	Affx-111644387	4A	544388730
AX-109547195	C/C	T/C	C/C	Affx-111813367	4A	471936748
AX-109551603	T/C	T/C	C/C	Affx-110551122	4A	710250345
AX-109557696	T/T	T/C	T/T	Affx-110598738	4A	363085623
AX-109560132	A/A	A/A	A/A	Affx-111400596	4A	617001134
AX-109576951	C/C	T/C	C/C	Affx-110420940	4A	607659749
AX-109581996	G/G	A/G	G/G	Affx-111252224	4A	107699007
AX-109582340	T/T	T/C	C/C	Affx-110301306	4A	624134756
AX-109586850	T/T	T/C	T/T	Affx-111900362	4A	215030134
AX-109589535	T/T	T/C	T/T	Affx-109726195	4A	325735172
AX-109591794	C/C	C/G	C/C	Affx-111777245	4A	379478873
AX-109592982	A/A	A/C	A/A	Affx-109127050	4A	608143197
AX-109598497	T/T	T/C	T/T	Affx-109629319	4A	462917533
AX-109605630	C/C	T/C	C/C	Affx-110414875	4A	393907277
AX-109606131	T/T	T/C	T/T	Affx-110899406	4A	322238693
AX-109608136	A/A	A/G	A/A	Affx-111708966	4A	236899213
AX-109608155	A/A	A/G	A/A	Affx-109127254	4A	192320047
AX-109611940	C/C	C/G	C/C	Affx-109335277	4A	233182829
AX-109612102	C/C	C/C	C/C	Affx-108916531	4A	363163179
AX-109621305	A/A	A/A	A/A	Affx-110172882	4A	301370958
AX-109621953	G/G	A/G	G/G	Affx-111021953	4A	4252653
AX-109623142	G/G	A/G	G/G	Affx-110041026	4A	299967005
AX-109626103	A/G	A/G	G/G	Affx-109907227	4A	605764861
AX-109626778	A/A	A/A	A/A	Affx-110226637	4A	728506942
AX-109635876	A/A	A/G	A/A	Affx-109452390	4A	310387847
AX-109636902	A/A	A/G	A/A	Affx-109675350	4A	177137981
AX-109641724	A/A	A/G	A/A	Affx-111684121	4A	326203583
AX-109653442	G/G	A/G	G/G	Affx-110207769	4A	705111908
AX-109816804	G/G	A/G	G/G	Affx-111105930	4A	457186764
AX-109821180	A/A	A/G	A/A	Affx-111738702	4A	381252914
AX-109821994	A/A	A/G	A/A	Affx-108999341	4A	30283352
AX-109829567	C/C	T/C	C/C	Affx-109302649	4A	358123548
AX-109831483	C/C	C/C	C/C	Affx-111935921	4A	253554130
AX-109831996	G/G	G/G	G/G	Affx-110891779	4A	723824292
AX-109832465	A/A	A/G	A/A	Affx-109546530	4A	370311709
AX-109832498	A/A	A/G	A/A	Affx-110531130	4A	182917881
AX-109837616	A/A	G/G	A/A	Affx-109349469	4A	688162850
AX-109837983	G/G	C/G	G/G	Affx-109719177	4A	452591821
AX-109838175	A/A	A/G	A/A	Affx-111771677	4A	235052820
AX-109844186	T/T	T/C	T/T	Affx-111117380	4A	260186477
AX-109846101	C/C	T/C	C/C	Affx-110402867	4A	234888406
AX-109847574	G/G	G/G	G/G	Affx-109516346	4A	454068934
AX-109849616	A/A	A/G	A/A	Affx-110417449	4A	113853276
AX-109850424	G/G	A/G	G/G	Affx-88626637	4A	83577736
AX-109865197	T/T	T/T	T/T	Affx-110653582	4A	305321313
AX-109865536	C/C	T/C	C/C	Affx-109218851	4A	59758336
AX-109871319	C/C	T/C	C/C	Affx-110382622	4A	636966545
AX-109872804	G/G	C/G	G/G	Affx-109239532	4A	174817775

AX-109873138	A/A	A/G	A/A	Affx-111727153	4A	18113634
AX-109874353	A/A	A/C	A/A	Affx-110744718	4A	354991981
AX-109877185	T/T	T/G	T/T	Affx-111956359	4A	353466969
AX-109887699	A/A	A/A	A/A	Affx-109982025	4A	360423850
AX-109897504	G/G	T/G	G/G	Affx-109040168	4A	124202338
AX-109899762	C/C	T/C	C/C	Affx-109711936	4A	307114322
AX-109903780	G/G	A/G	G/G	Affx-111754575	4A	457667921
AX-109906020	T/T	T/T	C/C	Affx-108864043	4A	724864050
AX-109906404	C/C	T/C	T/T	Affx-109314555	4A	602747129
AX-109906921	C/C	C/G	C/C	Affx-109301719	4A	175850359
AX-109907411	G/G	C/G	G/G	Affx-111402851	4A	460894784
AX-109909525	A/A	A/G	A/A	Affx-108988639	4A	397960287
AX-109910451	A/A	A/C	A/A	Affx-111192617	4A	89020303
AX-109912320	A/A	C/C	A/A	Affx-110694661	4A	133037135
AX-109915181	G/G	A/G	A/A	Affx-110612656	4A	707831859
AX-109915676	C/C	C/C	C/C	Affx-110488855	4A	103004266
AX-109915854	T/T	T/C	T/T	Affx-112003042	4A	455105192
AX-109918424	C/C	T/C	C/C	Affx-111404396	4A	417150345
AX-109924369	A/A	A/G	A/A	Affx-111207934	4A	692678872
AX-109926385	T/T	T/G	T/T	Affx-110062793	4A	717292028
AX-109930082	T/C	T/C	C/C	Affx-111137504	4A	615040986
AX-109933740	T/T	T/C	T/T	Affx-111765328	4A	232523882
AX-109935326	G/G	A/G	G/G	Affx-111526021	4A	576915354
AX-109937843	C/C	T/T	C/C	Affx-108987069	4A	544051718
AX-109940964	A/A	A/G	A/A	Affx-110971597	4A	307094791
AX-109941394	C/C	A/C	C/C	Affx-109892250	4A	69263904
AX-109942778	A/A	A/G	A/A	Affx-110952229	4A	204509238
AX-109943224	C/C	T/C	C/C	Affx-109929829	4A	344389722
AX-109944318	T/T	T/C	T/T	Affx-111891123	4A	670154221
AX-109947925	C/C	C/G	C/C	Affx-109089474	4A	625394661
AX-109950677	G/G	A/G	A/G	Affx-110627655	4A	102623557
AX-109952205	C/G	G/G	C/G	Affx-109289214	4A	113320208
AX-109955677	C/C	C/G	C/C	Affx-109778686	4A	343199577
AX-109958299	C/C	C/G	C/C	Affx-110392481	4A	450985342
AX-109966230	G/G	C/G	G/G	Affx-110681623	4A	131483037
AX-109969990	A/A	A/G	A/A	Affx-110977378	4A	450785581
AX-109975374	A/A	G/G	G/G	Affx-109975956	4A	605611795
AX-109981440	T/T	T/C	T/T	Affx-111844494	4A	89895885
AX-109987959	G/G	A/G	G/G	Affx-88672282	4A	176313163
AX-109989938	A/A	A/G	A/A	Affx-109029321	4A	197561715
AX-109997716	T/G	T/G	T/T	Affx-110522801	4A	109770546
AX-110000339	T/T	T/G	T/T	Affx-109217514	4A	381733790
AX-110003255	A/A	A/G	A/A	Affx-111817985	4A	608034508
AX-110004800	T/T	T/C	T/T	Affx-110779560	4A	448504214
AX-110008463	C/C	T/C	T/T	Affx-111971456	4A	624314019
AX-110013658	C/C	T/T	C/C	Affx-88666405	4A	719294633
AX-110016016	G/G	A/A	G/G	Affx-109211815	4A	488553399
AX-110025666	T/T	T/C	T/T	Affx-110524061	4A	210999660
AX-110028010	A/A	A/G	A/A	Affx-111997553	4A	694968010
AX-110029039	G/G	G/G	G/G	Affx-108850997	4A	616784428
AX-110030140	A/A	A/G	A/A	Affx-109327384	4A	60357887
AX-110031134	A/A	A/G	A/A	Affx-111261030	4A	446743459
AX-110033939	T/T	T/C	T/T	Affx-88733458	4A	86986458
AX-110038537	A/A	A/G	A/A	Affx-111396630	4A	376756974

AX-110051101	G/G	A/G	G/G	Affx-109122976	4A	614608229
AX-110063347	G/G	A/G	G/G	Affx-110694003	4A	300876463
AX-110065068	T/T	T/C	T/T	Affx-110387680	4A	226300072
AX-110070806	C/C	T/C	C/C	Affx-88630632	4A	545112318
AX-110079340	C/C	A/C	C/C	Affx-110888364	4A	446947823
AX-110090771	G/G	A/G	A/A	Affx-88413464	4A	597693615
AX-110105180	T/T	T/G	T/T	Affx-108955666	4A	549802369
AX-110136795	G/G	A/G	G/G	Affx-109940719	4A	217812207
AX-110137673	T/T	T/G	T/T	Affx-109734098	4A	299931728
AX-110152016	T/T	T/T	T/T	Affx-109299036	4A	342669201
AX-110152069	A/A	A/C	A/A	Affx-110019739	4A	304836280
AX-110153359	T/T	T/T	T/T	Affx-110148174	4A	621908244
AX-110156738	G/G	T/T	G/G	Affx-110081718	4A	227099916
AX-110159812	T/T	T/C	T/T	Affx-109905968	4A	239777402
AX-110160936	A/A	G/G	A/A	Affx-109616059	4A	132418119
AX-110167324	T/T	T/G	T/T	Affx-110913365	4A	240728588
AX-110170422	T/T	T/C	T/T	Affx-111767560	4A	370926619
AX-110170825	G/G	T/G	G/G	Affx-111125151	4A	383616359
AX-110171894	T/T	T/G	G/G	Affx-111921011	4A	605753144
AX-110173837	T/T	T/C	T/T	Affx-110094392	4A	594895428
AX-110188041	T/T	T/G	T/T	Affx-109067184	4A	231836914
AX-110189943	A/A	A/G	A/A	Affx-111541491	4A	131002609
AX-110375460	T/T	T/C	T/T	Affx-108981567	4A	243154560
AX-110378497	T/T	T/C	T/T	Affx-110864220	4A	567928784
AX-110381475	G/G	C/G	G/G	Affx-109983558	4A	611685687
AX-110383389	C/C	T/C	C/C	Affx-110892646	4A	418868157
AX-110389563	T/T	T/C	T/T	Affx-88790605	4A	593736783
AX-110400967	C/C	T/C	C/C	Affx-109852062	4A	17842239
AX-110407556	C/C	C/G	C/C	Affx-111076914	4A	352513820
AX-110411289	G/G	A/G	G/G	Affx-110988514	4A	34992384
AX-110413463	T/T	T/C	T/T	Affx-109825685	4A	252753933
AX-110418569	T/T	T/T	T/T	Affx-109070802	4A	246720295
AX-110421400	A/A	A/G	A/A	Affx-110533473	4A	324639269
AX-110422223	A/A	A/G	G/G	Affx-110624547	4A	37994192
AX-110424798	T/T	T/C	T/T	Affx-109309907	4A	405567987
AX-110428036	A/A	A/G	A/A	Affx-109157007	4A	404722805
AX-110432225	G/G	A/G	G/G	Affx-111701640	4A	239579535
AX-110434115	A/A	A/G	G/G	Affx-109366087	4A	660342651
AX-110440161	A/A	C/C	A/A	Affx-110851146	4A	635292292
AX-110441175	C/C	T/C	C/C	Affx-110349747	4A	673854310
AX-110441897	C/C	C/G	C/C	Affx-110849403	4A	31961071
AX-110444194	T/T	T/C	T/T	Affx-110112393	4A	24230776
AX-110448637	G/G	A/G	G/G	Affx-111370520	4A	626114839
AX-110453968	A/A	A/G	A/A	Affx-109935455	4A	252123154
AX-110461918	T/T	T/C	T/T	Affx-110810728	4A	234410553
AX-110463783	C/C	G/G	C/C	Affx-110201396	4A	619163932
AX-110464108	G/G	A/G	G/G	Affx-111158210	4A	566238363
AX-110468887	A/A	A/G	A/A	Affx-109662812	4A	491976508
AX-110468999	A/A	A/G	A/A	Affx-111690864	4A	634108610
AX-110475420	G/G	A/G	G/G	Affx-110993111	4A	128008334
AX-110477913	G/G	C/G	G/G	Affx-110531295	4A	65518451
AX-110482544	C/C	T/C	C/C	Affx-109126976	4A	191586585
AX-110492648	G/G	A/G	G/G	Affx-109592357	4A	663368503
AX-110495470	A/A	A/G	A/A	Affx-111589500	4A	295000074

AX-110495483	T/T	T/G	T/T	Affx-109517451	4A	196931088
AX-110496172	C/C	A/A	A/A	Affx-108874716	4A	39535627
AX-110497944	T/T	T/C	T/T	Affx-109527422	4A	623443281
AX-110498003	G/G	C/G	C/C	Affx-110678110	4A	11172578
AX-110499015	C/C	C/G	G/G	Affx-88572829	4A	665969616
AX-110502952	C/C	T/C	T/T	Affx-111688177	4A	13162802
AX-110503209	A/G	A/A	A/A	Affx-110042871	4A	50865546
AX-110506031	C/C	T/C	C/C	Affx-111021010	4A	262333845
AX-110507213	A/A	A/G	A/A	Affx-109560325	4A	543578640
AX-110513048	T/T	T/C	T/T	Affx-109139355	4A	243717076
AX-110517011	G/G	G/G	A/A	Affx-109118821	4A	616240584
AX-110517066	G/G	A/G	G/G	Affx-109602376	4A	607418782
AX-110517615	C/C	C/G	C/C	Affx-111900492	4A	477578034
AX-110519359	C/C	T/C	C/C	Affx-111521258	4A	388382392
AX-110521890	G/G	C/G	G/G	Affx-111730425	4A	248362051
AX-110525414	A/A	A/G	A/A	Affx-109514725	4A	101425969
AX-110544730	A/A	A/G	A/A	Affx-111595972	4A	105593476
AX-110546053	T/T	T/C	T/T	Affx-109682315	4A	380139725
AX-110546873	G/G	A/G	G/G	Affx-109687501	4A	243106027
AX-110551774	A/A	A/G	A/A	Affx-109201768	4A	84004390
AX-110552751	A/A	A/G	A/A	Affx-108975310	4A	110298500
AX-110553224	A/A	A/G	A/A	Affx-109051396	4A	194476085
AX-110557238	A/A	A/G	G/G	Affx-110859114	4A	603105510
AX-110557442	T/T	T/C	T/T	Affx-111384039	4A	127182154
AX-110562204	T/T	T/C	T/T	Affx-110298965	4A	309771447
AX-110564633	G/G	A/G	G/G	Affx-110153336	4A	171816949
AX-110567984	A/A	A/G	A/A	Affx-110767920	4A	308780434
AX-110568569	C/C	T/C	C/C	Affx-111333464	4A	401780030
AX-110570010	A/A	A/G	G/G	Affx-110924992	4A	629272194
AX-110572314	T/T	T/C	T/T	Affx-109859619	4A	323054685
AX-110572606	A/A	A/C	C/C	Affx-110369893	4A	11622245
AX-110574688	G/G	A/G	G/G	Affx-109875298	4A	583294977
AX-110589297	T/T	T/T	T/T	Affx-110280214	4A	209166286
AX-110594565	A/A	A/A	C/C	Affx-109430895	4A	16738393
AX-110595501	C/C	T/C	C/C	Affx-109676449	4A	125955942
AX-110604181	T/T	T/C	T/T	Affx-109861067	4A	27650871
AX-110608805	A/A	A/G	A/A	Affx-108850870	4A	348610204
AX-110609450	C/C	C/C	C/C	Affx-111980255	4A	185992956
AX-110619374	A/A	A/A	A/A	Affx-111074010	4A	189602409
AX-110623825	G/G	A/G	G/G	Affx-110518162	4A	2906761
AX-110624745	G/G	G/G	G/G	Affx-109691525	4A	726440339
AX-110632061	A/A	A/G	A/A	Affx-110729137	4A	310238453
AX-110637287	C/C	T/C	T/T	Affx-111730174	4A	630787052
AX-110645577	C/C	A/C	C/C	Affx-110603263	4A	447791554
AX-110650371	T/T	T/C	T/T	Affx-111821187	4A	465522756
AX-110654719	C/C	T/C	C/C	Affx-88703438	4A	250433381
AX-110660148	A/A	A/G	A/A	Affx-109486506	4A	225320730
AX-110667940	C/C	T/T	C/C	Affx-111433288	4A	114534224
AX-110669737	G/G	A/G	G/G	Affx-110471215	4A	647993252
AX-110679413	T/T	T/C	C/C	Affx-109363146	4A	604118467
AX-110682914	A/A	A/G	A/A	Affx-111904904	4A	410383505
AX-110683722	A/A	A/G	A/A	Affx-111529565	4A	206237731
AX-110685340	A/A	A/C	A/A	Affx-109335757	4A	433065093
AX-110695426	A/A	A/G	A/A	Affx-110496525	4A	414096110

AX-110695947	C/C	C/G	C/C	Affx-111512993	4A	314240348
AX-110699658	T/T	T/C	T/T	Affx-110776334	4A	283991721
AX-110699726	T/T	T/C	T/T	Affx-109842965	4A	419477132
AX-110711093	A/A	A/A	A/A	Affx-110813589	4A	204665120
AX-110713312	A/A	A/G	A/A	Affx-111975795	4A	429094129
AX-110713345	T/T	T/C	T/T	Affx-111996466	4A	347191097
AX-110714502	T/T	T/C	T/T	Affx-108969330	4A	567032796
AX-110715055	G/G	C/G	G/G	Affx-111893422	4A	320486427
AX-110715945	T/T	T/C	T/T	Affx-111134734	4A	216738481
AX-110721047	G/G	G/G	G/G	Affx-108900054	4A	263973818
AX-110724792	A/A	A/C	A/A	Affx-110535226	4A	435305580
AX-110732245	T/T	T/C	T/T	Affx-111042418	4A	422589208
AX-110736322	C/C	T/C	C/C	Affx-110216803	4A	540846999
AX-110912744	T/T	T/C	T/T	Affx-111318276	4A	107228194
AX-110913699	T/T	T/C	C/C	Affx-110872935	4A	658030337
AX-110915963	C/C	T/C	C/C	Affx-110333725	4A	85202379
AX-110918783	G/G	A/G	G/G	Affx-108866843	4A	302811098
AX-110920884	G/G	T/G	G/G	Affx-110741282	4A	686381407
AX-110925613	G/G	A/G	G/G	Affx-109171033	4A	209932966
AX-110928325	T/T	T/T	C/C	Affx-110054500	4A	704647631
AX-110931508	T/T	T/C	C/C	Affx-109407797	4A	632242065
AX-110939606	T/T	T/C	T/T	Affx-109484326	4A	327293743
AX-110941433	G/G	A/G	A/A	Affx-110583682	4A	40194450
AX-110943725	C/C	T/T	C/C	Affx-109509804	4A	454757425
AX-110943747	A/A	A/G	A/A	Affx-110433347	4A	312722683
AX-110947034	T/T	T/G	T/T	Affx-109064043	4A	397370118
AX-110947653	C/C	T/C	T/T	Affx-109216915	4A	620278125
AX-110948139	C/C	A/C	C/C	Affx-109343947	4A	462384495
AX-110954698	G/G	A/G	G/G	Affx-109771268	4A	178189915
AX-110955621	C/C	T/C	C/C	Affx-109313202	4A	136009700
AX-110957374	C/C	A/C	C/C	Affx-111207841	4A	562284355
AX-110958863	G/G	A/G	G/G	Affx-111563839	4A	403313269
AX-110961937	G/G	T/G	T/T	Affx-111357552	4A	605785021
AX-110962439	T/T	T/C	T/T	Affx-109078741	4A	303776128
AX-110966412	A/A	A/G	A/A	Affx-88619654	4A	733709860
AX-110967676	C/C	T/C	T/T	Affx-109703858	4A	604640113
AX-110971025	G/G	G/G	G/G	Affx-88390391	4A	641592792
AX-110971456	C/C	C/C	C/C	Affx-109643385	4A	241610599
AX-110971879	A/G	A/G	A/A	Affx-111674988	4A	47388390
AX-110972299	C/C	T/C	C/C	Affx-111828282	4A	139163959
AX-110973932	A/A	A/C	A/A	Affx-109139551	4A	344035688
AX-110978991	C/C	T/C	C/C	Affx-110180526	4A	550161506
AX-110980424	C/C	A/C	C/C	Affx-111617604	4A	581923370
AX-110985030	T/T	T/T	T/T	Affx-88422431	4A	593284738
AX-110989896	A/A	A/T	A/A	Affx-88446253	4A	725515273
AX-110990610	G/G	T/G	G/G	Affx-110015493	4A	538834340
AX-110990907	G/G	A/G	G/G	Affx-109555274	4A	175004875
AX-110990995	A/A	A/G	A/A	Affx-109577244	4A	87968750
AX-110996449	C/C	T/C	C/C	Affx-111298746	4A	367347533
AX-111000365	T/T	T/C	T/T	Affx-111387630	4A	451510985
AX-111003988	C/C	C/C	C/C	Affx-110631043	4A	59320646
AX-111004006	C/C	C/G	C/C	Affx-88742498	4A	440910199
AX-111012469	A/A	A/G	A/A	Affx-110657937	4A	251732649
AX-111014081	G/G	A/G	G/G	Affx-111888493	4A	111816817

AX-111017399	T/T	T/C	C/C	Affx-110437454	4A	655303475
AX-111019523	C/C	C/C	C/C	Affx-109291593	4A	27282972
AX-111025153	G/G	T/G	T/T	Affx-110111758	4A	685743313
AX-111028663	A/A	A/G	A/A	Affx-111438599	4A	210982112
AX-111033575	G/G	A/G	G/G	Affx-110673141	4A	438400938
AX-111036587	C/C	T/T	C/C	Affx-109884468	4A	717974550
AX-111040045	C/C	T/T	C/C	Affx-109705064	4A	632764370
AX-111041741	T/T	T/C	T/T	Affx-109073482	4A	244272896
AX-111043895	C/C	A/C	C/C	Affx-108963486	4A	119633876
AX-111045266	T/T	T/C	T/T	Affx-111729922	4A	381459131
AX-111047367	T/T	G/G	T/T	Affx-110038485	4A	402103713
AX-111050965	G/G	A/G	A/A	Affx-109698731	4A	703966317
AX-111052968	A/A	A/G	A/G	Affx-109423068	4A	3779204
AX-111054150	T/T	T/G	G/G	Affx-110309798	4A	617492598
AX-111056812	C/C	C/G	C/C	Affx-109355279	4A	379775825
AX-111056818	C/C	C/G	C/C	Affx-109456181	4A	463877070
AX-111060229	C/C	C/C	C/C	Affx-109896107	4A	695066644
AX-111061218	T/T	T/C	T/T	Affx-110156297	4A	626539165
AX-111072530	T/C	T/C	C/C	Affx-111798608	4A	669436779
AX-111073865	C/C	T/T	T/T	Affx-108957121	4A	649882935
AX-111075384	T/T	T/G	T/T	Affx-111630035	4A	425837222
AX-111077580	T/T	T/G	T/T	Affx-110153939	4A	25578658
AX-111077847	T/T	T/C	T/T	Affx-111749196	4A	69840804
AX-111079701	T/T	T/T	T/T	Affx-109067433	4A	611973106
AX-111080240	C/C	T/C	C/C	Affx-110660894	4A	449889292
AX-111080657	G/G	C/C	C/C	Affx-108935482	4A	618878648
AX-111082463	A/A	A/A	A/A	Affx-110242215	4A	164812973
AX-111088720	A/A	A/G	A/A	Affx-111499913	4A	565038133
AX-111091521	C/C	T/C	C/C	Affx-110916756	4A	391551250
AX-111093231	T/T	T/G	T/T	Affx-109466352	4A	412919825
AX-111097681	T/T	T/C	T/T	Affx-110409806	4A	429713439
AX-111097741	T/T	C/C	T/T	Affx-111233051	4A	130531560
AX-111099020	A/A	G/G	A/A	Affx-110764708	4A	119067913
AX-111102006	A/A	A/G	A/A	Affx-109183526	4A	432264282
AX-111103253	G/G	A/G	A/A	Affx-110813129	4A	650408536
AX-111108057	C/C	T/C	C/C	Affx-109645017	4A	349299114
AX-111115176	T/T	T/C	T/T	Affx-111018958	4A	185382050
AX-111116257	A/A	A/G	A/A	Affx-111835104	4A	461880041
AX-111116417	T/T	T/C	T/T	Affx-110355038	4A	313428495
AX-111122406	A/A	G/G	A/A	Affx-110746249	4A	112326134
AX-111122581	C/C	T/C	C/C	Affx-109796543	4A	41007610
AX-111123786	C/C	C/G	C/C	Affx-88779282	4A	720069028
AX-111124943	G/G	A/G	A/A	Affx-109890830	4A	38843724
AX-111127856	C/C	T/C	T/T	Affx-111626344	4A	690733305
AX-111133928	T/T	T/C	T/T	Affx-110812334	4A	221778743
AX-111134111	G/G	A/G	G/G	Affx-108990860	4A	138312670
AX-111140650	G/G	C/G	G/G	Affx-109235172	4A	634368256
AX-111143353	T/T	T/C	T/T	Affx-111367324	4A	135213716
AX-111144687	T/T	T/C	T/T	Affx-109965155	4A	549022738
AX-111150736	A/A	A/G	A/A	Affx-111442520	4A	250043729
AX-111155360	T/T	T/T	T/T	Affx-111391406	4A	605752401
AX-111157272	T/T	T/C	T/T	Affx-111101980	4A	585547742
AX-111164022	C/C	T/C	C/C	Affx-109516833	4A	149657857
AX-111189795	C/C	T/C	C/C	Affx-88621288	4A	616982210

AX-111190701	C/C	T/C	C/C	Affx-109396668	4A	100188255
AX-111196894	G/G	T/G	G/G	Affx-88463893	4A	85893318
AX-111207003	G/G	A/G	A/A	Affx-110169724	4A	684963302
AX-111215500	G/G	G/G	G/G	Affx-112002974	4A	223376866
AX-111218915	A/A	G/G	A/A	Affx-109871692	4A	169461200
AX-111221198	G/G	A/G	A/A	Affx-111656050	4A	709049773
AX-111224968	A/A	A/A	A/A	Affx-111497687	4A	257511116
AX-111229340	T/T	T/C	T/T	Affx-108943900	4A	178746629
AX-111229802	C/C	C/G	C/C	Affx-111622349	4A	571715193
AX-111233700	T/T	T/C	T/T	Affx-109600144	4A	556710774
AX-111240401	T/T	T/T	T/T	Affx-111705131	4A	404558508
AX-111241476	C/C	T/C	C/C	Affx-109158268	4A	45481056
AX-111246635	A/A	A/G	A/A	Affx-108889668	4A	218951670
AX-111255788	T/T	T/C	T/T	Affx-109601895	4A	302356720
AX-111265521	A/A	A/A	A/A	Affx-108995398	4A	436860711
AX-111268293	C/C	C/C	C/C	Affx-111816849	4A	394314570
AX-111269815	G/G	A/G	G/G	Affx-111985536	4A	411555312
AX-111275886	A/A	A/A	A/A	Affx-109083968	4A	439067727
AX-111282900	G/G	A/G	G/G	Affx-110724123	4A	99931771
AX-111451343	T/T	C/C	T/T	Affx-110203627	4A	499611798
AX-111453918	C/C	A/C	C/C	Affx-110587052	4A	634833572
AX-111455414	C/C	A/C	C/C	Affx-111593411	4A	413663730
AX-111456706	G/G	A/G	G/G	Affx-110815735	4A	369888696
AX-111462216	A/A	A/G	A/A	Affx-110814110	4A	26007667
AX-111466734	G/G	A/G	A/A	Affx-111596156	4A	45828504
AX-111466985	C/C	C/G	G/G	Affx-88627660	4A	596508514
AX-111467951	A/A	A/C	A/A	Affx-111402994	4A	398463372
AX-111475297	T/T	T/G	T/T	Affx-109947131	4A	19929740
AX-111486490	G/G	A/G	G/G	Affx-110036020	4A	428091895
AX-111486749	G/G	G/G	G/G	Affx-109674221	4A	679990778
AX-111489629	T/T	T/C	T/T	Affx-110752133	4A	608367255
AX-111491720	G/G	A/G	G/G	Affx-108878681	4A	568872627
AX-111491871	T/T	T/C	T/T	Affx-111931933	4A	350119101
AX-111492756	C/C	T/C	C/C	Affx-109513978	4A	104527477
AX-111494122	G/G	A/G	G/G	Affx-109855467	4A	165409007
AX-111494558	C/G	C/C	G/G	Affx-88773344	4A	13656042
AX-111496006	C/C	A/C	A/A	Affx-111204367	4A	702066069
AX-111502785	A/A	A/G	A/A	Affx-110445996	4A	695597362
AX-111508119	T/T	T/G	T/T	Affx-111562821	4A	398871722
AX-111512871	A/A	A/G	A/A	Affx-110186191	4A	246303434
AX-111514566	T/T	T/C	T/T	Affx-110982363	4A	248887725
AX-111516807	C/C	C/G	C/C	Affx-111697795	4A	15867998
AX-111517366	C/C	T/C	C/C	Affx-111290602	4A	386780991
AX-111517891	A/A	A/G	A/A	Affx-111753944	4A	225244831
AX-111518484	C/C	T/C	T/T	Affx-88375923	4A	649894759
AX-111524868	G/G	C/G	G/G	Affx-111515170	4A	38572576
AX-111526124	A/A	G/G	G/G	Affx-110127637	4A	631557405
AX-111527637	A/A	A/G	A/A	Affx-111636314	4A	426206439
AX-111528491	A/A	A/C	A/A	Affx-110283823	4A	496110069
AX-111529741	A/A	A/G	A/A	Affx-111588143	4A	327087786
AX-111530201	T/T	T/C	T/T	Affx-110814660	4A	207290085
AX-111534576	G/G	T/G	G/G	Affx-109674598	4A	66584231
AX-111535959	G/G	C/G	G/G	Affx-109641010	4A	254485741
AX-111537186	A/A	A/G	A/A	Affx-109168497	4A	613525981

AX-111537765	T/T	T/C	T/T	Affx-109379240	4A	410797513
AX-111539619	T/T	T/C	T/T	Affx-109906107	4A	302048701
AX-111550229	A/A	A/C	C/C	Affx-109568701	4A	661959307
AX-111556412	C/C	T/C	C/C	Affx-110045387	4A	572542614
AX-111559227	T/T	T/G	T/T	Affx-110084990	4A	576151848
AX-111559555	A/A	A/G	A/A	Affx-109216372	4A	346137584
AX-111559596	T/T	T/C	T/T	Affx-109908611	4A	415380598
AX-111563737	T/T	T/C	T/T	Affx-109831415	4A	350731597
AX-111566538	T/T	T/C	T/T	Affx-109922495	4A	89492828
AX-111567703	G/G	T/G	G/G	Affx-109134413	4A	66774207
AX-111568464	A/A	A/T	A/A	Affx-110281162	4A	458679865
AX-111577223	T/T	T/G	T/T	Affx-110761680	4A	427078459
AX-111592727	T/T	T/T	T/T	Affx-109360333	4A	639438855
AX-111595101	T/T	T/C	T/T	Affx-111694690	4A	368874375
AX-111600193	A/A	A/G	G/G	Affx-108862934	4A	642368254
AX-111606547	G/G	C/G	G/G	Affx-110994330	4A	202107240
AX-111608459	C/C	T/C	C/C	Affx-88535456	4A	577043564
AX-111612319	A/A	C/C	A/A	Affx-110940450	4A	544951380
AX-111612975	G/G	A/G	A/A	Affx-108912815	4A	605762329
AX-111620556	C/C	A/C	C/C	Affx-109967404	4A	21457087
AX-111624503	A/A	A/G	A/A	Affx-110101046	4A	532224647
AX-111627667	A/A	A/G	A/A	Affx-111460495	4A	378360717
AX-111628407	C/C	C/G	C/C	Affx-111154242	4A	314145680
AX-111629044	A/A	G/G	A/A	Affx-88788591	4A	29171671
AX-111631423	A/A	A/G	A/A	Affx-110727125	4A	133490130
AX-111631972	T/T	T/T	T/T	Affx-110640546	4A	619408262
AX-111632994	A/A	A/G	A/A	Affx-109778511	4A	368931761
AX-111634630	T/T	T/C	T/T	Affx-109076250	4A	444474180
AX-111635462	G/G	G/G	G/G	Affx-109434548	4A	106122676
AX-111635732	C/C	T/C	C/C	Affx-111157883	4A	537710083
AX-111648865	T/T	T/C	T/T	Affx-111583967	4A	513323192
AX-111652326	T/T	T/T	T/T	Affx-110740918	4A	346832721
AX-111660568	G/G	A/A	G/G	Affx-109639364	4A	555582681
AX-111662342	A/G	A/G	G/G	Affx-110305949	4A	640780733
AX-111664288	T/T	T/C	T/T	Affx-111008998	4A	247562357
AX-111664379	G/G	A/G	G/G	Affx-109679281	4A	424750154
AX-111666382	G/G	A/G	G/G	Affx-111263991	4A	497655031
AX-111668662	A/A	A/G	A/A	Affx-111722832	4A	383186144
AX-111679727	A/A	A/T	A/A	Affx-111115622	4A	97385587
AX-111704160	T/T	T/C	T/T	Affx-110549236	4A	621293968
AX-111711476	A/A	A/C	A/A	Affx-110250892	4A	669581507
AX-111720337	A/A	A/A	A/A	Affx-112312461	4A	596820651
AX-111724400	G/G	A/G	G/G	Affx-110555348	4A	165707527
AX-111725684	C/C	C/C	C/C	Affx-88743606	4A	673410578
AX-111736705	C/C	T/C	T/T	Affx-111817677	4A	594286762
AX-111757491	T/T	T/C	T/T	Affx-110210468	4A	23246102
AX-111759414	G/G	A/G	G/G	Affx-109721277	4A	607659964
AX-111759651	G/G	A/G	G/G	Affx-111595517	4A	605781362
AX-111802434	C/C	C/G	C/C	Affx-109490019	4A	607477416
AX-111820519	C/C	T/C	C/C	Affx-111746070	4A	639013716
AX-182045463	C/C	A/C	C/C	Affx-472282053	4A	435438329
AX-182047431	G/G	A/G	G/G	Affx-472285081	4A	674554685
AX-182101265	G/G	C/G	C/C	Affx-109423006	4A	688162455
AX-182101508	A/A	A/G	A/G	Affx-109457664	4A	607477716

AX-182102626	T/T	T/C	T/T	Affx-109612432	4A	688162575
AX-182109024	C/C	C/C	T/C	Affx-110504181	4A	616999189
AX-182130424	G/G	A/G	G/G	Affx-472274827	4A	511081600
AX-182175098	T/C	T/C	T/T	Affx-472318609	4A	660917654
AX-86184085	C/C	C/C	C/C	Affx-88411967	4A	594664440
AX-89333388	A/A	A/C	C/C	Affx-88364780	4A	684899213
AX-89389951	G/G	A/G	A/A	Affx-88422420	4A	606335407
AX-89428229	C/C	C/C	C/C	Affx-88460912	4A	595984457
AX-89475098	G/G	G/G	G/G	Affx-88507923	4A	577538225
AX-89518263	T/T	T/C	T/T	Affx-111104508	4A	720202333
AX-89552384	C/C	T/C	T/T	Affx-110789473	4A	616986956
AX-89567379	T/T	T/T	T/T	Affx-88600442	4A	612401584
AX-89590295	G/G	A/G	G/G	Affx-88623427	4A	590094228
AX-89597750	A/A	A/G	A/A	Affx-88630906	4A	489056536
AX-89608529	A/A	A/G	G/G	Affx-88641703	4A	605736295
AX-89704085	A/A	A/G	A/A	Affx-88736814	4A	700419229
AX-89739538	G/G	A/A	A/A	Affx-88772228	4A	40443801
AX-94394855	A/A	A/G	A/A	Affx-92825897	4A	323788515
AX-94400545	A/A	G/G	A/A	Affx-92626080	4A	584124176
AX-94542441	G/G	G/G	G/G	Affx-92535159	4A	585028316
AX-94544185	A/A	A/G	A/A	Affx-88743151	4A	684076698
AX-94545691	G/G	A/G	G/G	Affx-88773126	4A	608834862
AX-94610863	C/C	T/C	T/C	Affx-92622058	4A	590590535
AX-94705952	T/T	T/C	T/T	Affx-88632324	4A	615446891
AX-94729198	T/T	T/C	T/T	Affx-92453979	4A	548775724
AX-94747094	C/C	C/G	C/C	Affx-92331935	4A	688098707
AX-94796299	G/G	A/G	G/G	Affx-92825347	4A	546627198
AX-94807766	T/C	T/C	T/C	Affx-92511915	4A	705169743
AX-94923457	G/G	G/G	G/G	Affx-92686876	4A	688098661
AX-94971412	T/T	T/C	T/T	Affx-92644074	4A	240362011
AX-94981476	T/C	T/T	T/T	Affx-92512535	4A	688099392
AX-95104850	C/C	T/C	T/T	Affx-92695320	4A	688095748
AX-95109133	T/T	T/C	T/C	Affx-92282820	4A	617001800
AX-95133300	C/C	T/C	C/C	Affx-92155486	4A	68691437
AX-95142576	C/C	T/C	T/T	Affx-88599322	4A	43365322
AX-95237300	T/C	T/C	C/C	Affx-92665534	4A	712856709
AX-95629274	A/A	A/A	A/A	Affx-88611965	4A	38367204
AX-95630867	G/G	A/G	A/A	Affx-88670960	4A	665504435
AX-95630900	G/G	A/G	A/A	Affx-88543614	4A	597692833
AX-95654605	A/G	G/G	A/G	Affx-88433252	4A	125404137
AX-95661426	G/G	A/G	G/G	Affx-92982126	4A	590112266
AX-108730259	T/G	T/G	G/G	Affx-111384364	4B	3825576
AX-108737267	A/A	A/G	A/A	Affx-109162953	4B	546049947
AX-108739211	A/A	A/G	A/A	Affx-88733463	4B	4887172
AX-108743591	G/G	G/G	G/G	Affx-109963951	4B	662564529
AX-108744638	C/C	A/A	C/C	Affx-111678804	4B	547393888
AX-108750428	C/C	C/C	C/C	Affx-88518255	4B	557571
AX-108751245	C/C	T/C	C/C	Affx-109345524	4B	639126604
AX-108755302	A/A	A/T	A/A	Affx-88438186	4B	673473210
AX-108756572	T/T	T/G	T/T	Affx-111440378	4B	650927275
AX-108758643	C/C	T/C	T/T	Affx-110938253	4B	591576253
AX-108760637	A/A	A/G	A/A	Affx-110340588	4B	408884331
AX-108760910	G/G	A/G	G/G	Affx-110256153	4B	6813656
AX-108763423	C/C	T/C	C/C	Affx-109440970	4B	102522139

AX-108765412	G/G	G/G	G/G	Affx-109545983	4B	469050416
AX-108765521	G/G	A/G	A/A	Affx-111035290	4B	664643448
AX-108765576	T/T	T/G	T/T	Affx-110862836	4B	415707532
AX-108767762	A/A	C/C	C/C	Affx-109886302	4B	36395734
AX-108769486	C/C	T/C	C/C	Affx-88356145	4B	604699869
AX-108772683	C/C	T/C	C/C	Affx-109561887	4B	53861811
AX-108773581	C/C	T/C	C/C	Affx-110338177	4B	425121975
AX-108778637	G/G	A/G	G/G	Affx-109388545	4B	449553684
AX-108779354	T/T	T/G	T/T	Affx-111005865	4B	2331083
AX-108780242	C/C	T/C	T/T	Affx-110710374	4B	36998188
AX-108781802	C/C	T/C	C/C	Affx-109568195	4B	437181889
AX-108789625	G/G	G/G	G/G	Affx-110059565	4B	226901138
AX-108796389	A/A	A/G	A/A	Affx-111360005	4B	125177026
AX-108801401	C/C	T/C	C/C	Affx-109034862	4B	455664103
AX-108801712	G/G	C/G	G/G	Affx-110351562	4B	648780156
AX-108810685	A/A	A/G	G/G	Affx-109978913	4B	481242732
AX-108811389	C/C	T/C	C/C	Affx-109467227	4B	221203145
AX-108815183	C/C	T/C	C/C	Affx-110535635	4B	30963881
AX-108817437	G/G	G/G	G/G	Affx-88371036	4B	544445775
AX-108819885	C/C	C/C	C/C	Affx-110046127	4B	491081690
AX-108819906	T/T	T/C	T/T	Affx-88727619	4B	15616019
AX-108825702	C/C	C/G	C/C	Affx-110303535	4B	647849982
AX-108838330	A/G	A/G	A/A	Affx-110035463	4B	465981268
AX-108843065	C/C	T/C	C/C	Affx-111926236	4B	30422682
AX-108846181	T/T	T/T	T/T	Affx-109831869	4B	542803477
AX-108846403	T/T	T/C	T/T	Affx-110687111	4B	460415781
AX-108847266	T/T	T/C	T/T	Affx-109279242	4B	429339058
AX-108850496	G/G	A/G	G/G	Affx-109116641	4B	452094214
AX-108852650	T/T	T/T	T/T	Affx-109902817	4B	7019856
AX-108855305	A/A	A/A	G/G	Affx-110140346	4B	530910941
AX-108867943	C/C	T/C	C/C	Affx-110511416	4B	141423906
AX-108871853	G/G	A/G	G/G	Affx-110801271	4B	582494198
AX-108872710	A/A	A/T	A/A	Affx-109768711	4B	157906313
AX-108872915	A/A	A/C	A/A	Affx-109612354	4B	409392526
AX-108873214	C/C	A/A	C/C	Affx-110580845	4B	20228554
AX-108883190	A/A	A/G	G/G	Affx-88672537	4B	612026931
AX-108895386	T/G	T/G	T/G	Affx-111109543	4B	377022726
AX-108903411	G/G	T/G	T/T	Affx-111553180	4B	553629202
AX-108904078	A/A	A/G	A/A	Affx-111441264	4B	141682063
AX-108922248	G/G	A/G	G/G	Affx-111605176	4B	478848810
AX-108925984	C/C	T/C	C/C	Affx-109046738	4B	148208437
AX-108929144	A/A	A/C	C/C	Affx-111727156	4B	560047557
AX-108932466	G/G	G/G	G/G	Affx-110880097	4B	417231199
AX-108932515	G/G	A/A	G/G	Affx-110292872	4B	620870951
AX-108935256	G/G	G/G	G/G	Affx-110985349	4B	445689397
AX-108936384	G/G	T/G	T/T	Affx-88542206	4B	553252250
AX-108944018	G/G	A/A	G/G	Affx-88670881	4B	379296931
AX-108944519	C/C	T/T	C/C	Affx-111228740	4B	94801198
AX-108946167	T/T	T/G	T/T	Affx-110154750	4B	550393133
AX-108951002	C/C	T/C	C/C	Affx-109358036	4B	546380650
AX-108953195	T/C	T/T	T/T	Affx-111388226	4B	619080071
AX-108955555	C/C	C/G	G/G	Affx-108996622	4B	555938476
AX-108955591	G/G	A/G	G/G	Affx-108885809	4B	448988369
AX-108967914	T/T	T/C	T/T	Affx-111252772	4B	135957749

AX-108981554	T/T	T/C	T/T	Affx-109432829	4B	109050692
AX-108983733	G/G	A/G	A/A	Affx-111633249	4B	602657779
AX-108984536	A/A	A/G	A/A	Affx-88414106	4B	144684508
AX-108988739	C/C	A/C	C/C	Affx-109937366	4B	670057478
AX-108992830	A/G	G/G	G/G	Affx-111973085	4B	582225049
AX-108999430	A/A	A/G	A/A	Affx-109995482	4B	667683709
AX-108999867	A/A	A/G	A/A	Affx-110058761	4B	90717689
AX-109003749	C/C	C/C	C/C	Affx-109467876	4B	28736756
AX-109025374	C/C	T/C	C/C	Affx-109747527	4B	60423137
AX-109033164	C/C	T/C	C/C	Affx-109121058	4B	106934104
AX-109043204	A/A	A/G	A/A	Affx-111354154	4B	99894503
AX-109057982	C/G	C/G	C/G	Affx-110097224	4B	150564179
AX-109058133	C/C	T/C	C/C	Affx-111910215	4B	245967213
AX-109077061	A/A	A/T	A/A	Affx-88356901	4B	433967416
AX-109077371	A/G	G/G	A/G	Affx-109285545	4B	460873898
AX-109104755	G/G	A/G	G/G	Affx-111765617	4B	457275774
AX-109106609	C/C	T/C	C/C	Affx-111437170	4B	106257904
AX-109110130	C/C	T/C	C/C	Affx-110394023	4B	45419289
AX-109270209	T/T	T/C	T/T	Affx-110851398	4B	416688899
AX-109270926	C/C	T/C	C/C	Affx-111702459	4B	418761878
AX-109277201	C/C	A/C	C/C	Affx-109295446	4B	165638270
AX-109282409	T/G	T/G	G/G	Affx-109417823	4B	620058232
AX-109283664	C/C	A/C	A/A	Affx-88465379	4B	665404036
AX-109286577	T/T	A/T	T/T	Affx-111060328	4B	27537284
AX-109293700	A/A	A/A	A/A	Affx-110320210	4B	100578451
AX-109296842	A/A	A/C	A/A	Affx-109620140	4B	432891219
AX-109305650	G/G	A/G	G/G	Affx-109854794	4B	167334880
AX-109306007	T/T	T/C	T/T	Affx-110142464	4B	410558944
AX-109320255	G/G	A/G	G/G	Affx-109037619	4B	172526927
AX-109327739	C/G	G/G	C/G	Affx-110682178	4B	499534373
AX-109330683	G/G	G/G	G/G	Affx-109315519	4B	517563970
AX-109330857	G/G	A/G	A/A	Affx-109095872	4B	510914832
AX-109334541	C/C	T/T	C/C	Affx-110589475	4B	444835092
AX-109338618	T/T	T/C	T/T	Affx-109637988	4B	656241447
AX-109340409	A/A	A/G	G/G	Affx-109066984	4B	480827714
AX-109341809	A/A	A/G	A/A	Affx-111391494	4B	439322911
AX-109344427	C/C	T/C	C/C	Affx-110614533	4B	401165613
AX-109348684	A/A	A/T	T/T	Affx-88772809	4B	554816970
AX-109350990	T/T	T/C	T/C	Affx-110587100	4B	538607209
AX-109366326	A/A	A/G	A/A	Affx-111180410	4B	68295062
AX-109367178	T/T	T/C	T/T	Affx-111338315	4B	462134713
AX-109368728	G/G	C/G	G/G	Affx-110537942	4B	431418020
AX-109376424	G/G	G/G	G/G	Affx-111682035	4B	637734359
AX-109382842	T/T	T/T	C/C	Affx-109517562	4B	536427404
AX-109389257	A/A	A/G	A/A	Affx-110782353	4B	613092283
AX-109390649	G/G	G/G	A/A	Affx-111270923	4B	611483053
AX-109391034	G/G	A/G	G/G	Affx-109711415	4B	516359415
AX-109394806	A/A	A/G	A/A	Affx-111239703	4B	427715394
AX-109400188	T/T	T/C	T/T	Affx-111861248	4B	459007837
AX-109401270	G/G	A/G	G/G	Affx-109283675	4B	467624929
AX-109404611	C/C	C/C	C/C	Affx-111092765	4B	655770341
AX-109404699	G/G	A/G	G/G	Affx-111893419	4B	226540281
AX-109411152	C/C	C/C	C/C	Affx-88483666	4B	648499436
AX-109412222	C/C	T/C	C/C	Affx-111805058	4B	362135412

AX-109431795	G/G	A/G	G/G	Affx-109635417	4B	172430957
AX-109439249	C/C	C/C	T/T	Affx-111554515	4B	479415717
AX-109442600	C/C	A/C	C/C	Affx-109371601	4B	368653766
AX-109443970	C/C	T/C	C/C	Affx-110567160	4B	263259261
AX-109450442	C/C	T/C	C/C	Affx-111632246	4B	101034275
AX-109454068	G/G	A/G	G/G	Affx-111851251	4B	20732400
AX-109461383	A/A	A/C	C/C	Affx-109386313	4B	531804753
AX-109470797	C/C	A/C	C/C	Affx-88459258	4B	540328874
AX-109473487	T/T	G/G	T/T	Affx-109382564	4B	500833750
AX-109474466	C/C	T/C	C/C	Affx-111650103	4B	405737963
AX-109486127	T/T	T/C	T/T	Affx-111881971	4B	541138409
AX-109486719	C/C	T/C	C/C	Affx-109820494	4B	232412368
AX-109489399	C/C	T/C	C/C	Affx-111461797	4B	154684650
AX-109490418	G/G	A/G	G/G	Affx-88375610	4B	110443117
AX-109490801	C/C	C/C	C/C	Affx-110482655	4B	99743213
AX-109495166	G/G	G/G	G/G	Affx-111438561	4B	396263280
AX-109496762	T/T	T/C	T/T	Affx-110467297	4B	437542988
AX-109503404	A/A	A/G	A/A	Affx-110232091	4B	31117803
AX-109508177	A/A	A/G	A/A	Affx-110873273	4B	652231527
AX-109508708	G/G	A/G	G/G	Affx-110231811	4B	101967825
AX-109516755	A/A	A/G	A/A	Affx-111742978	4B	419816036
AX-109537396	T/T	T/G	G/G	Affx-109214462	4B	477239477
AX-109541334	A/A	A/G	A/G	Affx-88611684	4B	519338239
AX-109564075	T/T	T/C	C/C	Affx-111876245	4B	529675968
AX-109573634	T/T	A/T	T/T	Affx-109249581	4B	46609855
AX-109580651	G/G	A/G	G/G	Affx-110156381	4B	29195171
AX-109580890	C/C	T/C	C/C	Affx-109944388	4B	57481628
AX-109584489	G/G	C/G	G/G	Affx-110970805	4B	542367836
AX-109615458	G/G	T/G	G/G	Affx-110116237	4B	417911885
AX-109618065	C/C	C/G	C/C	Affx-110317075	4B	650256200
AX-109637078	T/T	T/C	C/C	Affx-111017781	4B	520152050
AX-109651759	C/C	T/C	C/C	Affx-111451024	4B	70319435
AX-109653070	G/G	G/G	G/G	Affx-88458563	4B	109316103
AX-109823421	A/A	A/C	A/A	Affx-111647189	4B	108344317
AX-109824765	C/C	T/C	C/C	Affx-109746548	4B	7038850
AX-109825313	C/C	T/C	C/C	Affx-111256501	4B	395315934
AX-109828730	C/C	C/G	C/C	Affx-110145821	4B	413524120
AX-109834673	A/A	A/G	G/G	Affx-110046208	4B	537800638
AX-109844491	G/G	G/G	T/T	Affx-110782517	4B	546913261
AX-109852597	T/T	T/C	T/T	Affx-108953929	4B	551524230
AX-109865884	G/G	A/G	G/G	Affx-109443081	4B	143284561
AX-109866370	G/G	A/G	G/G	Affx-110904243	4B	431265984
AX-109868698	A/A	G/G	G/G	Affx-109328074	4B	38456815
AX-109868843	G/G	C/G	G/G	Affx-109004819	4B	660541300
AX-109875431	C/C	T/C	C/C	Affx-110913693	4B	135499166
AX-109885115	C/C	G/G	C/C	Affx-110325901	4B	170853878
AX-109885529	C/C	T/C	T/T	Affx-109698439	4B	653685232
AX-109886612	C/C	T/C	C/C	Affx-110726039	4B	47448249
AX-109888571	A/A	A/G	A/A	Affx-110731814	4B	665203411
AX-109888584	C/C	T/C	T/T	Affx-110575188	4B	533747853
AX-109888756	T/T	T/G	T/T	Affx-110584322	4B	157404657
AX-109890177	G/G	T/G	T/T	Affx-88477980	4B	486275928
AX-109890179	T/T	T/T	T/C	Affx-108850558	4B	17379432
AX-109911996	C/C	T/C	C/C	Affx-88586504	4B	594356386

AX-109915480	T/T	T/C	T/T	Affx-109310021	4B	146681648
AX-109937343	T/T	T/C	T/T	Affx-109176969	4B	664060118
AX-109945364	T/T	T/C	T/T	Affx-111645809	4B	422933877
AX-109945538	C/C	T/C	C/C	Affx-111248429	4B	313985799
AX-109953958	C/C	T/C	T/T	Affx-111347282	4B	507357310
AX-109958106	A/A	A/G	G/G	Affx-111423987	4B	658347126
AX-109968140	C/C	C/C	C/C	Affx-109298129	4B	22255717
AX-109970053	C/C	T/C	C/C	Affx-110847982	4B	92387152
AX-109970298	C/C	C/C	C/C	Affx-111345839	4B	445196465
AX-109976508	C/C	T/C	C/C	Affx-110689201	4B	374912330
AX-109996117	A/A	A/G	A/A	Affx-88465452	4B	446289544
AX-110006837	T/G	T/G	T/G	Affx-111372331	4B	290162602
AX-110011440	A/A	A/G	A/A	Affx-111177906	4B	426592523
AX-110024661	G/G	A/G	G/G	Affx-109490273	4B	222573994
AX-110027725	G/G	A/G	G/G	Affx-110758402	4B	155279204
AX-110036233	C/C	T/C	C/C	Affx-111390462	4B	137550112
AX-110038932	A/G	A/G	A/G	Affx-88506647	4B	428198532
AX-110045185	G/G	A/A	G/G	Affx-110313305	4B	646379717
AX-110058582	T/T	T/C	T/T	Affx-110993849	4B	164537762
AX-110065819	G/G	C/C	G/G	Affx-110654942	4B	550525128
AX-110071052	A/A	A/G	A/A	Affx-110712539	4B	16385911
AX-110086840	A/G	G/G	A/G	Affx-109997438	4B	660666115
AX-110089362	C/C	A/C	C/C	Affx-111166548	4B	496421550
AX-110094666	A/A	A/G	A/A	Affx-111156322	4B	89501260
AX-110103911	G/G	A/G	G/G	Affx-109613254	4B	361732608
AX-110113053	A/A	A/G	A/A	Affx-108885641	4B	387870458
AX-110118973	G/G	A/G	G/G	Affx-111973231	4B	444115098
AX-110123920	A/A	A/G	A/A	Affx-110118254	4B	30865957
AX-110144677	C/C	T/C	C/C	Affx-110692508	4B	605156572
AX-110144838	G/G	A/G	G/G	Affx-110650592	4B	541414528
AX-110194716	G/G	G/G	G/G	Affx-110876459	4B	295427436
AX-110195987	T/T	T/C	T/T	Affx-109034670	4B	133917214
AX-110196145	A/A	A/G	A/A	Affx-110713147	4B	138699824
AX-110359713	G/G	T/G	G/G	Affx-111539209	4B	267026256
AX-110359993	C/C	A/C	C/C	Affx-109520818	4B	101682399
AX-110361956	C/C	C/C	C/C	Affx-88612113	4B	75741547
AX-110365713	G/G	C/G	G/G	Affx-111378583	4B	464813266
AX-110366613	G/G	C/G	G/G	Affx-88775240	4B	4947184
AX-110379435	C/C	T/C	C/C	Affx-109203157	4B	429292424
AX-110385750	C/C	A/C	C/C	Affx-111358614	4B	615171277
AX-110388342	T/T	T/T	T/T	Affx-110367989	4B	535030343
AX-110388876	G/G	G/G	G/G	Affx-111533393	4B	153692409
AX-110398879	A/A	G/G	A/A	Affx-111680948	4B	498949166
AX-110420496	T/T	T/C	T/T	Affx-110456696	4B	144884736
AX-110430111	T/T	T/C	T/T	Affx-110329555	4B	548887946
AX-110431208	A/A	C/C	A/A	Affx-109650457	4B	646910479
AX-110436318	G/G	A/G	G/G	Affx-110296246	4B	562867549
AX-110436979	G/G	G/G	G/G	Affx-110101023	4B	73152359
AX-110437226	C/C	T/C	C/C	Affx-109648531	4B	408272016
AX-110445927	C/C	C/C	C/C	Affx-110471348	4B	136424487
AX-110447227	G/G	A/G	G/G	Affx-110308722	4B	456666333
AX-110456258	T/T	A/T	T/T	Affx-110891684	4B	139907967
AX-110456764	G/G	A/G	G/G	Affx-111103154	4B	241782281
AX-110460111	T/T	T/T	T/T	Affx-109646184	4B	158313969

AX-110460498	C/C	T/C	C/C	Affx-88692452	4B	44924279
AX-110465483	C/C	T/C	C/C	Affx-111754861	4B	452708066
AX-110473522	C/C	T/C	C/C	Affx-109656379	4B	14583784
AX-110476859	C/C	T/C	C/C	Affx-111148140	4B	489433948
AX-110486518	A/A	A/G	A/A	Affx-108938305	4B	438870962
AX-110492111	C/C	T/C	C/C	Affx-110448949	4B	145378188
AX-110496718	C/C	T/C	C/C	Affx-109274439	4B	498455753
AX-110498186	G/G	A/G	G/G	Affx-110319790	4B	605731413
AX-110516900	T/T	T/C	T/T	Affx-111626777	4B	669712645
AX-110522681	C/C	T/C	T/T	Affx-111287853	4B	532268818
AX-110523915	C/C	T/C	C/C	Affx-109252867	4B	412109377
AX-110524514	G/G	A/G	G/G	Affx-88521691	4B	666613002
AX-110525046	G/G	G/G	G/G	Affx-111341710	4B	459579255
AX-110531267	T/T	T/C	T/T	Affx-88474031	4B	15414989
AX-110544397	T/T	T/C	C/C	Affx-111863679	4B	521379073
AX-110552647	A/A	A/G	G/G	Affx-110432414	4B	557660096
AX-110557012	A/G	A/A	A/G	Affx-109304332	4B	500088386
AX-110560008	T/T	T/C	T/T	Affx-110601684	4B	449829952
AX-110565938	G/G	G/G	G/G	Affx-111566273	4B	164115877
AX-110567615	A/A	A/G	A/A	Affx-109944398	4B	69867518
AX-110574019	C/C	T/C	T/T	Affx-110914534	4B	500712576
AX-110576785	A/A	G/G	A/A	Affx-109845699	4B	9943558
AX-110578000	C/C	T/C	C/C	Affx-109192679	4B	62000957
AX-110589591	T/T	T/T	C/C	Affx-88542744	4B	483764593
AX-110594414	T/C	T/C	T/C	Affx-109385302	4B	99045762
AX-110598304	C/C	T/C	C/C	Affx-108956412	4B	113629711
AX-110598973	G/G	C/G	C/C	Affx-110426615	4B	488579377
AX-110611878	T/C	T/C	T/T	Affx-108911238	4B	4245836
AX-110617743	C/C	A/A	A/A	Affx-109448064	4B	527116165
AX-110618009	T/C	T/T	T/C	Affx-111697940	4B	7044373
AX-110623354	C/C	C/C	C/C	Affx-109408851	4B	86064438
AX-110625039	T/T	T/C	T/T	Affx-110947724	4B	69386167
AX-110646091	G/G	G/G	G/G	Affx-109524723	4B	96933102
AX-110667705	G/G	A/G	A/A	Affx-109102444	4B	505919637
AX-110676376	C/C	T/C	T/T	Affx-110490224	4B	561517480
AX-110689694	T/T	T/C	T/T	Affx-111313200	4B	412528707
AX-110693775	G/G	G/G	A/A	Affx-109747097	4B	535364998
AX-110707631	G/G	A/G	G/G	Affx-110033981	4B	68839539
AX-110712645	A/A	A/G	G/G	Affx-111126662	4B	606850409
AX-110739442	T/C	C/C	T/C	Affx-111160620	4B	595795512
AX-110744838	T/T	A/T	T/T	Affx-109882547	4B	441164286
AX-110904766	C/C	T/C	C/C	Affx-88480093	4B	427070882
AX-110909842	C/C	T/C	C/C	Affx-110806880	4B	56124138
AX-110918826	C/C	C/G	C/C	Affx-109161437	4B	167777632
AX-110919561	C/C	C/C	C/C	Affx-108966249	4B	151093499
AX-110924350	T/T	T/C	T/T	Affx-109682493	4B	473286262
AX-110924859	T/T	C/C	T/T	Affx-472274074	4B	417138322
AX-110928817	T/T	T/C	C/C	Affx-109732983	4B	36010784
AX-110935762	G/G	A/G	G/G	Affx-109058387	4B	484780340
AX-110938398	C/C	C/C	T/T	Affx-109487096	4B	43794951
AX-110943296	T/T	T/C	T/T	Affx-110865055	4B	159347956
AX-110948898	A/G	G/G	A/G	Affx-110603029	4B	46197091
AX-110963107	T/T	T/C	C/C	Affx-110406450	4B	19726931
AX-110964980	C/G	C/G	C/G	Affx-111724782	4B	1367405

AX-110971353	C/C	T/C	C/C	Affx-109319198	4B	341638072
AX-110975012	G/G	A/G	G/G	Affx-110765792	4B	158893260
AX-110986344	G/G	A/G	G/G	Affx-110807849	4B	339115780
AX-111003696	G/G	A/G	G/G	Affx-109321811	4B	155810252
AX-111007966	A/G	G/G	A/G	Affx-110776325	4B	165135485
AX-111010159	C/C	C/C	T/C	Affx-111439451	4B	530235988
AX-111011650	G/G	T/G	T/T	Affx-110902645	4B	562049557
AX-111022564	A/A	A/A	A/A	Affx-110239128	4B	195165547
AX-111025706	G/G	A/G	G/G	Affx-109510507	4B	650547340
AX-111028236	T/T	T/C	T/T	Affx-109760512	4B	424010077
AX-111036309	A/A	A/A	A/A	Affx-109369517	4B	91185749
AX-111039156	T/T	C/C	T/T	Affx-109002028	4B	406309370
AX-111043077	T/T	T/C	T/T	Affx-109147646	4B	421994341
AX-111047109	A/A	A/G	A/A	Affx-110365632	4B	547701482
AX-111051657	G/G	T/G	G/G	Affx-110783599	4B	350585995
AX-111055753	G/G	A/G	G/G	Affx-111044350	4B	497878771
AX-111056525	C/C	T/C	C/C	Affx-111242895	4B	195248705
AX-111057050	T/T	T/G	T/T	Affx-111405023	4B	654587036
AX-111057902	A/A	C/C	A/A	Affx-88613506	4B	652373508
AX-111058090	C/C	T/C	C/C	Affx-111610840	4B	646115685
AX-111058939	C/C	A/C	C/C	Affx-111468601	4B	7046592
AX-111065695	C/C	C/C	C/C	Affx-110610421	4B	152038448
AX-111066123	C/C	T/T	T/T	Affx-109958507	4B	556889082
AX-111068079	G/G	G/G	G/G	Affx-111522722	4B	25349716
AX-111070464	G/G	A/G	G/G	Affx-110923307	4B	419229713
AX-111071365	T/T	C/C	T/T	Affx-111941834	4B	93282841
AX-111074167	G/G	T/G	G/G	Affx-111575490	4B	51188028
AX-111080767	A/A	A/G	A/G	Affx-110013533	4B	608675042
AX-111089153	A/A	A/G	A/A	Affx-111038072	4B	643788807
AX-111091451	G/G	A/G	G/G	Affx-111995455	4B	421542151
AX-111102215	T/T	T/C	T/T	Affx-109282611	4B	643278722
AX-111104382	T/T	T/T	T/T	Affx-111730753	4B	170344692
AX-111107314	G/G	A/G	A/A	Affx-111860669	4B	14110399
AX-111113300	C/C	C/G	G/G	Affx-109007308	4B	591171273
AX-111114716	C/C	T/C	C/C	Affx-110057222	4B	610226665
AX-111115843	C/C	C/C	T/T	Affx-88495978	4B	480592448
AX-111123332	C/C	C/G	C/G	Affx-109243835	4B	537537305
AX-111123475	T/C	T/T	C/C	Affx-109546482	4B	611015480
AX-111127678	C/C	A/C	C/C	Affx-111755932	4B	435978201
AX-111129840	A/A	A/G	A/A	Affx-109784130	4B	171826253
AX-111134841	T/T	T/C	T/T	Affx-111968012	4B	551079076
AX-111135027	G/G	A/G	G/G	Affx-111814798	4B	461336532
AX-111135044	T/T	T/G	T/T	Affx-110530601	4B	634706677
AX-111139813	C/C	C/G	C/C	Affx-110957156	4B	20230356
AX-111142832	G/G	A/G	G/G	Affx-111513757	4B	347866370
AX-111144316	T/T	A/T	T/T	Affx-110723311	4B	533876941
AX-111147540	C/C	T/C	C/C	Affx-111186510	4B	227345411
AX-111177101	C/C	T/C	C/C	Affx-111719193	4B	72904122
AX-111193334	T/T	T/G	T/T	Affx-111557940	4B	592671692
AX-111195432	T/C	T/T	T/C	Affx-111095138	4B	405050690
AX-111230804	C/C	T/C	C/C	Affx-109415136	4B	526159151
AX-111241403	T/T	T/C	T/T	Affx-110350399	4B	420802503
AX-111254019	C/C	C/C	C/C	Affx-110582479	4B	363712509
AX-111258815	C/C	T/C	C/C	Affx-111167710	4B	63008236

AX-111451315	G/G	T/G	G/G	Affx-111652949	4B	446731838
AX-111458984	G/G	A/G	G/G	Affx-88433034	4B	26826017
AX-111462813	A/A	A/A	A/A	Affx-88454874	4B	621436952
AX-111478022	C/C	T/C	T/T	Affx-110318717	4B	481803752
AX-111479600	A/A	A/A	A/A	Affx-88406759	4B	415125929
AX-111480041	C/C	T/C	T/T	Affx-88441559	4B	590138529
AX-111486674	G/G	G/G	A/G	Affx-111502448	4B	518872464
AX-111488369	A/A	A/G	A/G	Affx-88702181	4B	483336920
AX-111489554	T/T	T/G	T/T	Affx-110417960	4B	67344433
AX-111492474	T/T	T/G	T/T	Affx-109637381	4B	194422555
AX-111492749	T/T	T/C	C/C	Affx-111928040	4B	502718049
AX-111496693	G/G	A/G	G/G	Affx-110436835	4B	495324975
AX-111498788	G/G	T/G	G/G	Affx-110390116	4B	670846106
AX-111503872	G/G	C/G	C/C	Affx-111135467	4B	636757506
AX-111506685	T/T	T/T	T/T	Affx-111915246	4B	548768349
AX-111507276	C/C	C/C	C/C	Affx-109890742	4B	434712719
AX-111516589	T/T	T/C	T/T	Affx-108975613	4B	20304554
AX-111517823	T/C	T/C	T/T	Affx-108890268	4B	552203769
AX-111517964	G/G	A/G	G/G	Affx-88388066	4B	51191177
AX-111521785	T/T	T/C	T/T	Affx-110273574	4B	148376125
AX-111524568	C/C	C/G	C/C	Affx-110718997	4B	291366784
AX-111529675	G/G	A/A	G/G	Affx-109650806	4B	163050441
AX-111534302	G/G	A/G	G/G	Affx-110801250	4B	205108979
AX-111535446	A/A	A/G	G/G	Affx-111495159	4B	536101581
AX-111536609	T/C	C/C	T/C	Affx-111592051	4B	134831987
AX-111544338	G/G	A/A	G/G	Affx-88588103	4B	641174612
AX-111546987	C/G	C/G	G/G	Affx-111195303	4B	620263416
AX-111547150	A/C	C/C	C/C	Affx-111642309	4B	557813971
AX-111551708	G/G	C/G	G/G	Affx-88550771	4B	105713491
AX-111555225	T/T	T/C	T/T	Affx-109667496	4B	161970074
AX-111560371	T/T	T/C	T/T	Affx-109056603	4B	642749559
AX-111567546	C/C	T/C	C/C	Affx-111823345	4B	169419980
AX-111569876	A/A	A/G	A/A	Affx-109146612	4B	103771654
AX-111571727	G/G	G/G	G/G	Affx-109147582	4B	367782573
AX-111574640	T/C	C/C	T/C	Affx-109646361	4B	496876502
AX-111578160	T/T	T/C	T/T	Affx-108929218	4B	418209013
AX-111585045	A/A	A/G	A/A	Affx-110795673	4B	40755024
AX-111588547	C/C	T/C	T/T	Affx-109788778	4B	514866899
AX-111591811	G/G	T/G	G/G	Affx-88371636	4B	411491488
AX-111595278	G/G	C/G	G/G	Affx-110480759	4B	672958573
AX-111604877	C/C	T/C	C/C	Affx-110338000	4B	407386120
AX-111612159	A/A	A/G	A/A	Affx-109492491	4B	656717964
AX-111614876	C/C	A/C	C/C	Affx-109114009	4B	639857187
AX-111617938	A/A	A/G	G/G	Affx-109300772	4B	37688518
AX-111622536	A/G	G/G	A/G	Affx-111953871	4B	495892209
AX-111623537	A/A	A/G	A/A	Affx-111614072	4B	545011277
AX-111623649	G/G	A/G	G/G	Affx-109180993	4B	430923114
AX-111632283	C/C	T/C	C/C	Affx-111375818	4B	166698372
AX-111633224	G/G	A/A	G/G	Affx-88492111	4B	645296602
AX-111640796	G/G	G/G	G/G	Affx-110508361	4B	12536696
AX-111647053	A/A	A/C	A/A	Affx-110260053	4B	406811860
AX-111648532	G/G	G/G	A/A	Affx-88540529	4B	601516460
AX-111649698	T/T	T/C	C/C	Affx-110870226	4B	609415296
AX-111657163	G/G	A/G	A/A	Affx-110919939	4B	43230034

AX-111662284	T/T	T/G	T/T	Affx-109314213	4B	655177013
AX-111662410	A/A	A/T	A/A	Affx-88621002	4B	540413913
AX-111663104	A/A	A/G	A/A	Affx-109647032	4B	20304301
AX-111667739	A/A	A/G	A/A	Affx-111080698	4B	414139582
AX-111671536	A/A	A/T	T/T	Affx-109974135	4B	658966576
AX-111679434	A/A	A/G	G/G	Affx-111184594	4B	527815800
AX-111683241	C/C	C/C	C/C	Affx-109744224	4B	104308067
AX-111691756	A/A	A/G	A/A	Affx-110722984	4B	603576226
AX-111691908	C/C	T/C	C/C	Affx-110767816	4B	8560072
AX-111695911	T/T	T/C	T/T	Affx-111233507	4B	20282304
AX-111698919	A/A	A/C	A/A	Affx-110729901	4B	139629282
AX-111699737	G/G	A/G	G/G	Affx-88540431	4B	482647861
AX-111732484	A/A	A/G	A/A	Affx-111518673	4B	667887948
AX-111735154	A/A	A/T	A/A	Affx-110615807	4B	40436039
AX-111738184	C/C	T/C	C/C	Affx-110033042	4B	7047330
AX-111760190	T/T	T/C	C/C	Affx-88567688	4B	562340964
AX-111761210	G/G	A/G	G/G	Affx-88785061	4B	543746525
AX-111763540	G/G	A/G	G/G	Affx-110855101	4B	416105024
AX-111763569	C/C	T/C	C/C	Affx-109529276	4B	414696680
AX-111776570	T/T	C/C	T/T	Affx-109342989	4B	166096293
AX-111776628	G/G	A/A	A/A	Affx-108883096	4B	528821927
AX-111780380	A/A	A/G	A/A	Affx-111977757	4B	428712872
AX-112287873	A/A	A/A	A/A	Affx-88756747	4B	30839572
AX-112288720	G/G	G/G	A/G	Affx-112313622	4B	633763697
AX-112289353	A/A	A/A	A/A	Affx-88488956	4B	515161617
AX-182025148	C/C	T/C	C/C	Affx-110832049	4B	20282413
AX-182039057	G/G	A/A	G/G	Affx-472270655	4B	13975531
AX-182120078	C/C	T/C	C/C	Affx-112315190	4B	609498148
AX-182137578	T/T	T/T	T/T	Affx-472284649	4B	602235941
AX-86165280	T/C	T/C	T/C	Affx-92414287	4B	14118285
AX-86174001	T/T	T/C	C/C	Affx-92619364	4B	538997269
AX-86175614	A/A	A/A	A/A	Affx-88517414	4B	569630673
AX-86178412	A/A	A/G	A/A	Affx-92994681	4B	475806832
AX-86179821	T/T	T/T	T/T	Affx-88347995	4B	636739593
AX-89380210	C/C	C/C	C/C	Affx-88412594	4B	652854834
AX-89473451	C/C	T/C	T/T	Affx-88506272	4B	659168760
AX-89475307	T/G	G/G	T/T	Affx-88508133	4B	7038414
AX-89572432	C/C	C/C	C/C	Affx-88605507	4B	595709543
AX-89577308	C/C	T/C	T/T	Affx-88610391	4B	559647884
AX-89704189	A/A	A/G	G/G	Affx-88736918	4B	11426983
AX-89708030	G/G	A/G	A/A	Affx-88740744	4B	35634723
AX-89716448	C/C	T/C	C/C	Affx-88749151	4B	65940889
AX-94400582	T/C	C/C	T/C	Affx-92151811	4B	672235944
AX-94434500	C/C	C/C	C/C	Affx-92867229	4B	31881778
AX-94438527	T/T	T/C	T/T	Affx-88658390	4B	26491532
AX-94450937	G/G	A/G	G/G	Affx-88345766	4B	13977274
AX-94461604	C/C	T/C	C/C	Affx-92613344	4B	21384179
AX-94463730	G/G	A/A	G/G	Affx-92347405	4B	616274703
AX-94464472	G/G	C/G	C/C	Affx-92756196	4B	532719023
AX-94474747	A/A	A/G	A/A	Affx-92122006	4B	638589038
AX-94533084	C/C	T/C	C/C	Affx-92892512	4B	97924593
AX-94599381	G/G	G/G	G/G	Affx-92344279	4B	467712180
AX-94607619	G/G	G/G	G/G	Affx-92447579	4B	32252401
AX-94651309	G/G	G/G	G/G	Affx-92449642	4B	516503489

AX-94663391	G/G	A/G	G/G	Affx-92343293	4B	667413219
AX-94724471	G/G	C/G	C/G	Affx-88501484	4B	6084778
AX-94745154	C/C	T/C	C/C	Affx-92340198	4B	575042251
AX-94777230	A/A	A/C	C/C	Affx-92103982	4B	587471728
AX-94807699	A/A	A/G	A/A	Affx-92348715	4B	50404102
AX-94833233	A/A	A/G	A/A	Affx-92102375	4B	7037800
AX-94955456	G/G	A/G	A/A	Affx-92914304	4B	588431258
AX-95090958	C/C	T/T	C/C	Affx-92497224	4B	668987435
AX-95123781	C/C	C/G	C/C	Affx-92554673	4B	61306776
AX-95127068	T/T	T/C	T/T	Affx-92672567	4B	122310781
AX-95192653	C/C	T/T	C/C	Affx-92464227	4B	451495995
AX-95204903	C/G	G/G	G/G	Affx-88475090	4B	600367681
AX-95629588	G/G	A/G	G/G	Affx-92992885	4B	179738095
AX-95630628	A/A	C/C	A/A	Affx-88622428	4B	10437508
AX-95632191	A/G	G/G	A/G	Affx-88767510	4B	7043773
AX-95658062	C/C	T/T	T/T	Affx-88733624	4B	589178247
AX-95659409	A/A	A/G	G/G	Affx-92991737	4B	519794605
AX-95684402	C/C	T/C	C/C	Affx-88594969	4B	12892078
AX-95684545	T/T	T/T	T/T	Affx-88654040	4B	7037407
AX-108726608	T/T	T/C	T/T	Affx-111599206	4D	98066048
AX-108758749	G/G	C/G	G/G	Affx-111347325	4D	281303701
AX-108764247	A/A	A/T	A/A	Affx-109825063	4D	118435050
AX-108775441	A/G	A/G	A/G	Affx-88479468	4D	344368928
AX-108785930	C/C	T/C	C/C	Affx-111836132	4D	11217714
AX-108830474	C/C	C/C	C/C	Affx-109993758	4D	413032782
AX-108833523	G/G	A/G	G/G	Affx-111528097	4D	169868021
AX-108846076	G/G	C/G	G/G	Affx-88637408	4D	431518846
AX-108849036	C/C	T/C	C/C	Affx-109001228	4D	478832743
AX-108866224	G/G	C/G	G/G	Affx-111746205	4D	477019747
AX-108868821	G/G	T/G	G/G	Affx-108937536	4D	385630348
AX-108874010	G/G	T/G	G/G	Affx-111708616	4D	51305319
AX-108880252	C/C	T/C	C/C	Affx-110749619	4D	208384736
AX-108893617	G/G	A/G	G/G	Affx-111011789	4D	317510851
AX-108905056	T/T	T/G	T/T	Affx-88736764	4D	54131840
AX-108913628	G/G	C/G	G/G	Affx-88595688	4D	89535136
AX-108919604	G/G	A/G	G/G	Affx-111939994	4D	40039039
AX-108920376	G/G	A/G	G/G	Affx-88715839	4D	267200588
AX-108949163	C/C	T/C	C/C	Affx-110037522	4D	295112214
AX-108959486	G/G	A/G	G/G	Affx-110685121	4D	138804313
AX-108968169	A/A	A/G	A/A	Affx-111086519	4D	136468228
AX-109040303	C/C	C/C	C/C	Affx-110390184	4D	193303299
AX-109067446	C/C	C/C	C/C	Affx-111049695	4D	203168902
AX-109072188	T/T	T/C	T/T	Affx-111001543	4D	254073000
AX-109078146	C/C	T/T	C/C	Affx-110438543	4D	19007729
AX-109142060	T/T	T/C	T/T	Affx-110466644	4D	176907162
AX-109168318	G/G	A/G	G/G	Affx-111413951	4D	150778318
AX-109230716	G/G	A/G	G/G	Affx-109339827	4D	37812931
AX-109254069	G/G	G/G	G/G	Affx-110354543	4D	310222699
AX-109265317	C/C	C/C	C/C	Affx-110914206	4D	373587959
AX-109294476	C/C	T/T	T/C	Affx-109469849	4D	13058073
AX-109301283	C/C	T/C	C/C	Affx-109781271	4D	506938266
AX-109303311	G/G	A/G	G/G	Affx-109758235	4D	502834145
AX-109311273	G/G	A/G	G/G	Affx-109046355	4D	239346092
AX-109333522	G/G	T/G	G/G	Affx-109289396	4D	73418044

AX-109374357	A/A	A/G	A/A	Affx-111974500	4D	58887032
AX-109408679	C/C	T/T	C/C	Affx-88349764	4D	265722005
AX-109410812	A/A	A/G	A/A	Affx-110562424	4D	411592916
AX-109415509	A/A	A/G	A/A	Affx-110613149	4D	139566904
AX-109444707	T/T	A/T	T/T	Affx-111951809	4D	388932779
AX-109473050	C/C	T/C	C/C	Affx-109128525	4D	251964914
AX-109480349	A/A	A/C	A/A	Affx-111334540	4D	29022964
AX-109500517	A/A	A/G	A/A	Affx-109200355	4D	113319521
AX-109517658	G/G	A/G	G/G	Affx-109001614	4D	46714062
AX-109544451	C/C	T/T	C/C	Affx-110584757	4D	97387298
AX-109577418	A/A	G/G	A/A	Affx-110085477	4D	461846121
AX-109626693	G/G	A/G	G/G	Affx-88739277	4D	447882899
AX-109676199	T/T	T/C	T/T	Affx-111933192	4D	131761593
AX-109725873	A/A	A/T	A/A	Affx-88525475	4D	110236899
AX-109732646	T/T	T/C	T/T	Affx-111285768	4D	96147593
AX-109744296	C/C	T/C	C/C	Affx-109698705	4D	268664858
AX-109775486	C/C	T/C	C/C	Affx-111514070	4D	413584571
AX-109777243	A/A	A/G	A/A	Affx-111648843	4D	508932496
AX-109801979	G/G	A/G	G/G	Affx-111686082	4D	353641108
AX-109811892	T/T	T/C	T/T	Affx-111899321	4D	291641856
AX-109840748	G/G	A/G	G/G	Affx-109635290	4D	376363879
AX-109854739	G/G	A/G	G/G	Affx-111231246	4D	335373516
AX-109855610	T/T	T/C	T/T	Affx-109374223	4D	492037036
AX-109863735	C/C	T/C	C/C	Affx-111586014	4D	474915087
AX-109880021	T/T	T/C	T/T	Affx-110622563	4D	445106001
AX-109880580	C/C	A/C	C/C	Affx-88784920	4D	347480952
AX-109883229	G/G	T/G	T/T	Affx-109878766	4D	435197067
AX-109887289	G/G	C/G	G/G	Affx-109094166	4D	386481287
AX-109902086	A/A	A/A	A/A	Affx-109134736	4D	46048569
AX-109917825	G/G	G/G	G/G	Affx-110381645	4D	242952540
AX-109941006	C/C	T/C	C/C	Affx-88687432	4D	141083152
AX-109970514	G/G	T/G	G/G	Affx-109770456	4D	226613847
AX-109978559	A/A	A/G	A/A	Affx-109363318	4D	420919592
AX-110005081	A/A	A/T	A/A	Affx-109569544	4D	482515892
AX-110005953	G/G	G/G	G/G	Affx-109742413	4D	7202982
AX-110013165	A/A	A/G	A/A	Affx-110965626	4D	42143832
AX-110025501	T/T	T/T	T/T	Affx-110756760	4D	240967236
AX-110040955	G/G	A/G	G/G	Affx-109165732	4D	290335021
AX-110046962	A/A	A/G	A/A	Affx-88462488	4D	9555772
AX-110048819	G/G	C/G	G/G	Affx-88797200	4D	456500110
AX-110068845	T/T	T/G	T/T	Affx-109815365	4D	175585236
AX-110070510	G/G	G/G	G/G	Affx-109554346	4D	3259369
AX-110077470	T/T	T/C	T/T	Affx-111565577	4D	157425509
AX-110080795	C/C	A/C	C/C	Affx-110790576	4D	24260827
AX-110086640	C/C	T/C	C/C	Affx-110106857	4D	4286804
AX-110124262	C/C	C/C	C/C	Affx-111484903	4D	361268203
AX-110125921	C/C	G/G	C/C	Affx-109386967	4D	506334208
AX-110130415	C/C	C/G	C/C	Affx-110894338	4D	466435263
AX-110155453	A/A	A/G	A/A	Affx-111150561	4D	206233015
AX-110195764	G/G	A/G	G/G	Affx-108958938	4D	156562341
AX-110203330	A/A	A/G	A/A	Affx-88779021	4D	481959444
AX-110228302	T/T	T/C	T/T	Affx-109239474	4D	181975287
AX-110248393	T/T	T/C	T/T	Affx-109409612	4D	470636367
AX-110292338	G/G	A/G	G/G	Affx-111593990	4D	168841711

AX-110358910	G/G	C/G	G/G	Affx-109464149	4D	444873395
AX-110379757	T/T	T/G	T/T	Affx-110983980	4D	40456400
AX-110414620	G/G	A/G	G/G	Affx-110739495	4D	285773259
AX-110427760	C/C	T/C	C/C	Affx-88665548	4D	454354587
AX-110432863	G/G	G/G	G/G	Affx-88443828	4D	424857331
AX-110457483	T/T	T/C	T/T	Affx-110229509	4D	287609273
AX-110465046	C/C	T/C	C/C	Affx-110437046	4D	86844023
AX-110468740	C/C	T/C	C/C	Affx-111264214	4D	118605639
AX-110475524	C/C	T/C	C/C	Affx-110059959	4D	56710958
AX-110488905	C/C	T/C	C/C	Affx-111565752	4D	51307126
AX-110501305	C/C	T/C	C/C	Affx-109405353	4D	102600907
AX-110506790	G/G	C/G	G/G	Affx-109945646	4D	320826530
AX-110508642	C/C	T/C	C/C	Affx-109189924	4D	494147174
AX-110528269	T/T	T/C	T/T	Affx-110528161	4D	312590066
AX-110545576	C/C	T/C	C/C	Affx-88444695	4D	211038049
AX-110554000	G/G	A/G	G/G	Affx-88788585	4D	499095490
AX-110562226	T/T	T/C	T/T	Affx-111602110	4D	152123826
AX-110565602	A/A	A/G	A/A	Affx-111154277	4D	31398343
AX-110568577	C/C	C/G	C/C	Affx-111994785	4D	200403100
AX-110580938	T/T	T/C	T/T	Affx-111182520	4D	34721161
AX-110605497	T/T	T/C	T/T	Affx-88729096	4D	472162984
AX-110779068	T/T	T/C	T/T	Affx-88388592	4D	129387446
AX-110824009	A/G	A/G	A/G	Affx-111465593	4D	503271463
AX-110913622	G/G	T/G	G/G	Affx-109277604	4D	18463269
AX-110924545	G/G	A/G	G/G	Affx-111797027	4D	376307284
AX-110927163	G/G	T/G	G/G	Affx-111127204	4D	18471735
AX-110930425	A/A	A/A	A/A	Affx-88494554	4D	419300070
AX-110932954	G/G	G/G	G/G	Affx-88754676	4D	80221905
AX-110938872	A/A	A/C	A/A	Affx-108949853	4D	260743736
AX-110950982	A/A	A/C	A/A	Affx-109256171	4D	134261400
AX-110951951	C/C	C/G	C/C	Affx-111303410	4D	133615550
AX-110960877	G/G	A/G	G/G	Affx-111367521	4D	282304038
AX-110963720	A/A	A/C	A/A	Affx-111157803	4D	318329830
AX-110965371	T/T	T/C	T/T	Affx-110101096	4D	90693063
AX-110975379	G/G	A/G	G/G	Affx-110836282	4D	57491949
AX-110984199	G/G	G/G	G/G	Affx-109976380	4D	508262908
AX-110985744	T/T	T/C	T/T	Affx-109238428	4D	432949214
AX-110986986	A/A	A/G	A/A	Affx-109852513	4D	80609335
AX-111034497	C/C	T/C	C/C	Affx-110188096	4D	43460997
AX-111038704	G/G	A/G	G/G	Affx-110342547	4D	197605726
AX-111092299	G/G	A/G	G/G	Affx-111435270	4D	426603281
AX-111099416	T/T	T/C	T/T	Affx-109488891	4D	23904945
AX-111109088	T/T	T/C	T/T	Affx-109657184	4D	215403291
AX-111138121	A/A	A/A	A/A	Affx-88633837	4D	125227152
AX-111142287	G/G	A/G	G/G	Affx-111089964	4D	135660929
AX-111142906	T/T	T/C	T/T	Affx-109990530	4D	172954584
AX-111212212	G/G	A/G	G/G	Affx-110917600	4D	428014392
AX-111217552	A/A	A/G	A/A	Affx-109145563	4D	319086831
AX-111241745	T/T	T/T	T/T	Affx-108851292	4D	18785331
AX-111291738	C/C	C/G	C/C	Affx-111902938	4D	60163600
AX-111326720	T/T	T/T	T/T	Affx-110903673	4D	81637730
AX-111339779	T/T	T/C	T/T	Affx-110148936	4D	60740067
AX-111367897	T/T	C/C	T/T	Affx-110711329	4D	233161725
AX-111412197	C/C	T/C	C/C	Affx-88802146	4D	142538143

AX-111473671	T/C	T/C	T/C	Affx-109780192	4D	12537234
AX-111473765	C/C	T/C	C/C	Affx-109588685	4D	413648248
AX-111482406	C/C	A/C	C/C	Affx-88761504	4D	458264630
AX-111483158	A/A	A/G	A/A	Affx-109135004	4D	47752714
AX-111486136	A/A	A/A	A/A	Affx-110259415	4D	369262204
AX-111486871	C/C	C/G	C/C	Affx-111570314	4D	334120740
AX-111487973	C/G	G/G	C/G	Affx-109600227	4D	464854314
AX-111490698	A/A	A/G	A/A	Affx-88369819	4D	456052036
AX-111515970	G/G	A/G	G/G	Affx-109779483	4D	506466425
AX-111525938	T/C	T/T	C/C	Affx-111876632	4D	6798584
AX-111533335	G/G	A/G	G/G	Affx-110035348	4D	393846315
AX-111544209	C/C	C/G	C/C	Affx-110486596	4D	338444992
AX-111544932	T/T	T/C	T/T	Affx-88438902	4D	218795581
AX-111557122	T/G	T/T	T/G	Affx-108878392	4D	288430275
AX-111559361	G/G	A/G	G/G	Affx-109815695	4D	151601448
AX-111567243	T/T	T/G	T/T	Affx-109773251	4D	469891516
AX-111580635	C/C	T/C	C/C	Affx-88704025	4D	294021419
AX-111597798	C/C	C/C	C/C	Affx-88776304	4D	497362371
AX-111619291	G/G	A/G	G/G	Affx-111753949	4D	64626849
AX-111649369	A/A	A/G	A/A	Affx-111150993	4D	95800636
AX-111673530	G/G	A/G	G/G	Affx-109682904	4D	220005763
AX-111677925	G/G	A/G	G/G	Affx-108942674	4D	1184415
AX-111696733	G/G	T/G	G/G	Affx-109489546	4D	220208966
AX-111707375	G/G	A/G	G/G	Affx-109564689	4D	399728999
AX-111763265	G/G	G/G	G/G	Affx-111540695	4D	242387373
AX-111798748	C/C	C/G	C/C	Affx-111131358	4D	248693367
AX-111837027	T/T	T/T	T/T	Affx-88767535	4D	34458320
AX-111862796	C/C	T/C	C/C	Affx-88678729	4D	320423570
AX-111865096	C/C	T/C	C/C	Affx-109425386	4D	134736196
AX-111914504	A/A	G/G	A/A	Affx-88565469	4D	472356332
AX-111921300	T/T	T/C	T/T	Affx-111348244	4D	469764871
AX-111972968	C/C	A/C	C/C	Affx-111227930	4D	196203170
AX-112287112	T/T	T/T	T/T	Affx-112313905	4D	263055057
AX-179476902	A/C	A/C	A/C	Affx-292486199	4D	503271370
AX-89318480	A/A	A/G	A/A	Affx-88349337	4D	68421035
AX-89349130	G/G	A/G	G/G	Affx-88380923	4D	436911985
AX-89353317	A/A	A/C	A/A	Affx-88385206	4D	478670664
AX-89398182	G/G	A/G	G/G	Affx-88430705	4D	419137042
AX-89434670	T/T	T/T	T/T	Affx-88467377	4D	87946998
AX-89453387	C/C	C/G	C/C	Affx-88486153	4D	466337398
AX-89487309	G/G	T/G	G/G	Affx-88520167	4D	500821668
AX-89528361	G/G	G/G	G/G	Affx-88561303	4D	99275361
AX-89554948	C/C	A/C	C/C	Affx-88587984	4D	440289165
AX-89590551	C/C	A/A	C/C	Affx-88623683	4D	358475408
AX-89622172	G/G	G/G	G/G	Affx-88655368	4D	108216244
AX-89635542	A/A	A/C	A/A	Affx-88668328	4D	505369602
AX-89638215	G/G	T/G	G/G	Affx-88671004	4D	14429909
AX-89640744	A/A	A/G	A/A	Affx-88673535	4D	484938376
AX-89669974	C/C	T/C	C/C	Affx-92364772	4D	32347368
AX-94388553	C/C	C/C	C/C	Affx-92811934	4D	62463199
AX-94404950	A/A	A/A	G/G	Affx-92342248	4D	497036741
AX-94458172	G/G	G/G	G/G	Affx-92187424	4D	195021466
AX-94514321	A/T	A/A	A/T	Affx-92248470	4D	98303433
AX-94525959	G/G	A/G	G/G	Affx-92245307	4D	54411252

AX-94540236	C/C	C/C	C/C	Affx-92659022	4D	230606409
AX-94540844	T/C	T/T	T/C	Affx-92462334	4D	32349945
AX-94562005	A/A	G/G	A/A	Affx-92357091	4D	215219463
AX-94621737	T/T	T/T	T/T	Affx-92387223	4D	331637335
AX-94626067	G/G	G/G	G/G	Affx-92616418	4D	18783040
AX-94660634	A/A	A/G	A/A	Affx-92435599	4D	145504215
AX-94685783	G/G	C/G	G/G	Affx-92156777	4D	196235481
AX-94740564	A/A	A/G	A/A	Affx-88673044	4D	75228746
AX-94811965	T/T	T/C	T/T	Affx-92685356	4D	475029819
AX-94940465	G/G	A/G	G/G	Affx-92825280	4D	489187576
AX-94944176	T/C	T/C	T/C	Affx-92495977	4D	69850169
AX-95120271	T/C	T/C	T/C	Affx-92294897	4D	4132769
AX-95129154	T/G	T/T	T/G	Affx-92572285	4D	88153191
AX-95205240	C/C	C/C	C/C	Affx-92349884	4D	18783144
AX-95659405	G/G	A/A	G/G	Affx-88443737	4D	11567401
AX-95684199	C/C	T/C	C/C	Affx-88570948	4D	62469566
AX-108726870	C/C	T/T	T/T	Affx-88754621	5A	422053063
AX-108727324	C/C	G/G	C/C	Affx-88775334	5A	584055533
AX-108728294	T/T	A/T	T/T	Affx-111782460	5A	698212184
AX-108730624	C/C	A/C	A/A	Affx-109290458	5A	510161940
AX-108730664	C/C	C/C	C/C	Affx-108966784	5A	691531371
AX-108732431	T/C	T/C	C/C	Affx-109340504	5A	665987395
AX-108737654	G/G	A/G	G/G	Affx-111315242	5A	688070569
AX-108738342	G/G	A/G	G/G	Affx-109558534	5A	627985757
AX-108738809	G/G	C/G	G/G	Affx-109811512	5A	671572567
AX-108738893	T/T	T/C	C/C	Affx-110838778	5A	116567201
AX-108739527	A/A	A/C	C/C	Affx-88462284	5A	500625774
AX-108744704	G/G	T/G	T/T	Affx-111597081	5A	324074490
AX-108745515	C/C	C/G	G/G	Affx-88722399	5A	172050990
AX-108747780	T/T	T/C	C/C	Affx-109633575	5A	334002117
AX-108749190	A/G	A/G	G/G	Affx-88513286	5A	504909690
AX-108751490	G/G	A/G	G/G	Affx-111540366	5A	704535018
AX-108752051	A/A	A/G	A/A	Affx-110875549	5A	486728142
AX-108752495	C/C	C/G	C/C	Affx-111661588	5A	698733438
AX-108754749	G/G	A/G	A/A	Affx-110095561	5A	47183512
AX-108755627	A/A	A/C	A/A	Affx-111198689	5A	667094623
AX-108760060	A/A	A/G	G/G	Affx-109043589	5A	504451456
AX-108760305	C/C	T/C	C/C	Affx-110441638	5A	95467117
AX-108761227	C/C	C/G	C/C	Affx-108887552	5A	631400913
AX-108762454	C/C	T/C	T/T	Affx-109817320	5A	523610904
AX-108764636	G/G	A/G	A/A	Affx-111863025	5A	30649056
AX-108765823	G/G	A/G	G/G	Affx-109430482	5A	15109455
AX-108768128	C/C	T/C	C/C	Affx-111135256	5A	626973600
AX-108775851	C/C	C/G	G/G	Affx-110719723	5A	448615891
AX-108778546	A/A	G/G	G/G	Affx-110627753	5A	478321505
AX-108781805	C/C	T/C	T/T	Affx-88640363	5A	430238341
AX-108784117	G/G	G/G	G/G	Affx-88784372	5A	103425842
AX-108784886	A/A	A/G	G/G	Affx-88370949	5A	46574485
AX-108784978	T/T	T/C	T/T	Affx-111392292	5A	671312496
AX-108791192	T/T	T/C	C/C	Affx-110006099	5A	528370390
AX-108798179	G/G	C/G	G/G	Affx-88648059	5A	561698620
AX-108799536	C/C	C/C	C/C	Affx-110627751	5A	599209670
AX-108806718	A/A	A/C	A/A	Affx-111688844	5A	435245426
AX-108809007	A/G	A/G	A/G	Affx-110623163	5A	591948083

AX-108809340	C/C	T/T	C/C	Affx-110954911	5A	669733312
AX-108813905	G/G	A/G	A/A	Affx-111381453	5A	47440953
AX-108820536	C/C	C/C	C/C	Affx-110002131	5A	593394265
AX-108826245	C/C	C/C	T/C	Affx-110119740	5A	521297046
AX-108827165	A/A	A/G	A/A	Affx-111174288	5A	573653003
AX-108829675	G/G	C/G	G/G	Affx-110122902	5A	680593348
AX-108839873	C/C	C/G	C/G	Affx-88756790	5A	2083211
AX-108845213	A/A	A/C	A/A	Affx-109945642	5A	484627643
AX-108849435	A/A	A/G	A/A	Affx-109650931	5A	619685664
AX-108849763	C/C	T/C	C/C	Affx-109724721	5A	580471334
AX-108855890	G/G	C/G	G/G	Affx-109667645	5A	662510222
AX-108856072	G/G	C/G	C/C	Affx-109470809	5A	11704201
AX-108860059	A/A	A/C	A/A	Affx-110778906	5A	107747814
AX-108872310	G/G	T/G	T/T	Affx-110524967	5A	307125951
AX-108873291	G/G	A/G	G/G	Affx-108952018	5A	52349714
AX-108877013	C/C	T/C	C/C	Affx-111737968	5A	537922195
AX-108882806	C/C	C/C	C/C	Affx-111084110	5A	72843102
AX-108885457	G/G	A/G	G/G	Affx-109933747	5A	579957237
AX-108888015	C/C	C/C	C/C	Affx-110331741	5A	587542737
AX-108888026	G/G	A/G	G/G	Affx-88567240	5A	66847396
AX-108892277	A/A	A/G	A/A	Affx-111212343	5A	466858066
AX-108895342	T/T	T/C	T/T	Affx-110764348	5A	597294532
AX-108904079	G/G	C/G	G/G	Affx-88460628	5A	98883765
AX-108905823	G/G	A/G	A/A	Affx-109898799	5A	489785569
AX-108906603	G/G	T/G	G/G	Affx-109075128	5A	71693085
AX-108908151	C/C	C/C	C/C	Affx-110544929	5A	566613893
AX-108919814	T/T	T/C	T/T	Affx-109586958	5A	403282787
AX-108921355	C/C	T/C	C/C	Affx-109607265	5A	264849011
AX-108922021	G/G	G/G	A/A	Affx-111433695	5A	394533743
AX-108926220	G/G	G/G	G/G	Affx-110798001	5A	482823535
AX-108926681	C/C	T/C	T/T	Affx-109036681	5A	294268956
AX-108932459	G/G	A/G	G/G	Affx-110802632	5A	598025963
AX-108937232	G/G	T/G	G/G	Affx-109647333	5A	462154797
AX-108950164	C/C	T/C	C/C	Affx-109780614	5A	410798375
AX-108951190	G/G	C/G	C/C	Affx-109228882	5A	613535939
AX-108953048	T/T	T/G	T/T	Affx-111617021	5A	89812286
AX-108955237	T/T	T/C	T/T	Affx-109287812	5A	468811587
AX-108959608	A/A	G/G	G/G	Affx-110809135	5A	496637843
AX-108969270	C/C	T/C	T/T	Affx-110999027	5A	32770473
AX-108980399	G/G	A/G	G/G	Affx-111218534	5A	700207993
AX-108987114	C/C	T/C	C/C	Affx-111024792	5A	12558497
AX-108990192	G/G	A/G	A/A	Affx-110740809	5A	117493461
AX-108995932	G/G	G/G	G/G	Affx-112314603	5A	9845562
AX-108997623	T/C	T/C	T/C	Affx-88518743	5A	587413011
AX-108998564	A/A	A/G	A/A	Affx-111022422	5A	479907422
AX-109008196	G/G	C/G	C/C	Affx-109267737	5A	9654013
AX-109032399	A/A	A/G	G/G	Affx-111461393	5A	455331298
AX-109038152	T/T	T/C	C/C	Affx-108894976	5A	439778184
AX-109038371	G/G	A/G	A/A	Affx-110079937	5A	532031271
AX-109053128	C/C	T/C	T/T	Affx-88616146	5A	423030474
AX-109066543	A/A	A/G	A/A	Affx-110138846	5A	689412500
AX-109069771	G/G	T/T	G/G	Affx-88575150	5A	415676129
AX-109071599	A/A	A/G	G/G	Affx-109511329	5A	517377106
AX-109076608	C/C	A/C	A/A	Affx-111717600	5A	532479423

AX-109078400	T/T	T/G	T/T	Affx-109705665	5A	438324990
AX-109270241	A/A	A/C	A/A	Affx-110797684	5A	107454635
AX-109278656	T/T	T/C	T/T	Affx-111107321	5A	698139847
AX-109281292	T/T	T/C	T/T	Affx-88534723	5A	34526416
AX-109284888	C/C	T/T	C/C	Affx-88565523	5A	68545999
AX-109285497	G/G	A/G	G/G	Affx-111081958	5A	494549505
AX-109288145	A/G	G/G	G/G	Affx-109858664	5A	394919729
AX-109296973	C/C	T/C	T/T	Affx-109396714	5A	160365315
AX-109300036	T/C	C/C	C/C	Affx-111927388	5A	64362236
AX-109301126	A/A	A/G	G/G	Affx-109669624	5A	384463504
AX-109302500	A/A	A/G	G/G	Affx-109132496	5A	621854970
AX-109303338	A/C	A/C	A/A	Affx-111165868	5A	214312449
AX-109312058	A/A	A/G	A/A	Affx-109800025	5A	698140191
AX-109316828	C/C	T/C	C/C	Affx-88363911	5A	677839663
AX-109320027	C/C	T/C	C/C	Affx-111500494	5A	706151849
AX-109321224	C/C	T/C	T/T	Affx-109260211	5A	21124842
AX-109325762	G/G	G/G	A/A	Affx-108995232	5A	451013373
AX-109326479	C/C	C/C	C/C	Affx-110210170	5A	709180671
AX-109329647	C/C	T/C	C/C	Affx-110792140	5A	382764200
AX-109330975	T/T	T/C	T/T	Affx-110284575	5A	633151157
AX-109336809	A/A	A/G	G/G	Affx-109359828	5A	8223137
AX-109339144	C/C	T/C	C/C	Affx-111281332	5A	93067697
AX-109339207	A/A	A/G	G/G	Affx-111985601	5A	523140753
AX-109339422	G/G	A/G	G/G	Affx-110924325	5A	593076700
AX-109341178	G/G	C/C	C/C	Affx-111878252	5A	515876173
AX-109341558	C/C	T/C	T/T	Affx-110166890	5A	617019058
AX-109348871	A/A	A/C	A/A	Affx-108928121	5A	689910228
AX-109349084	C/C	T/C	T/T	Affx-110549100	5A	11256304
AX-109353062	A/A	A/G	A/A	Affx-110637930	5A	586202021
AX-109355412	T/T	T/C	C/C	Affx-111268760	5A	533018904
AX-109359134	G/G	A/G	G/G	Affx-109775883	5A	396552359
AX-109359437	C/C	T/C	C/C	Affx-110633724	5A	23816215
AX-109363529	G/G	G/G	G/G	Affx-109125088	5A	698186813
AX-109365992	C/C	T/C	C/C	Affx-111039809	5A	596061230
AX-109366083	C/C	C/C	C/C	Affx-111942319	5A	86888413
AX-109366963	T/T	T/C	C/C	Affx-110415775	5A	515146592
AX-109369427	C/C	T/C	C/C	Affx-110339734	5A	546521932
AX-109371064	G/G	C/G	C/C	Affx-109422026	5A	308721791
AX-109373006	C/C	T/C	C/C	Affx-109268883	5A	54520253
AX-109374431	A/A	A/G	A/A	Affx-109707456	5A	595601337
AX-109375147	T/T	T/C	T/C	Affx-111959815	5A	1900210
AX-109379355	C/C	T/C	T/T	Affx-110954164	5A	244437850
AX-109379388	G/G	A/G	G/G	Affx-108861129	5A	99601799
AX-109386650	T/T	T/C	T/C	Affx-110625650	5A	20638374
AX-109399826	C/C	T/C	C/C	Affx-109100158	5A	81147802
AX-109406297	T/T	T/C	T/T	Affx-109768297	5A	608040095
AX-109407552	T/T	T/C	T/T	Affx-111529082	5A	459915316
AX-109410102	A/A	G/G	A/A	Affx-110485460	5A	395834363
AX-109410756	T/T	T/C	T/T	Affx-111764028	5A	698135305
AX-109411504	G/G	A/G	A/A	Affx-110677022	5A	9480014
AX-109412773	A/A	A/G	A/A	Affx-111107125	5A	78041572
AX-109430578	A/A	A/T	A/A	Affx-111465087	5A	635564295
AX-109432156	G/G	A/G	G/G	Affx-110964949	5A	552164955
AX-109432509	C/C	A/C	C/C	Affx-109007254	5A	374847556

AX-109434336	G/G	G/G	G/G	Affx-109261900	5A	709761060
AX-109434576	G/G	A/A	G/G	Affx-111009124	5A	457945935
AX-109436609	C/C	T/C	C/C	Affx-108892403	5A	680063456
AX-109438159	T/T	T/T	T/T	Affx-109453299	5A	452991474
AX-109439402	G/G	A/G	A/A	Affx-110065314	5A	414892950
AX-109440005	C/C	T/C	T/T	Affx-110309163	5A	4277047
AX-109447403	T/T	T/G	T/T	Affx-109719306	5A	92016766
AX-109449510	C/C	A/C	A/A	Affx-111366210	5A	480825839
AX-109451388	G/G	A/G	A/A	Affx-110664090	5A	27506250
AX-109451992	C/C	T/C	C/C	Affx-110558688	5A	663368927
AX-109459768	G/G	G/G	G/G	Affx-109627557	5A	540357613
AX-109461522	G/G	A/G	G/G	Affx-108955585	5A	67193968
AX-109466244	C/C	C/G	C/C	Affx-111933591	5A	458663071
AX-109468708	T/T	T/G	T/T	Affx-111330687	5A	535242446
AX-109474493	A/A	A/G	A/A	Affx-109450169	5A	107746833
AX-109477775	T/T	C/C	C/C	Affx-111227896	5A	31570960
AX-109490579	C/C	C/C	C/C	Affx-110781410	5A	576289359
AX-109494596	G/G	A/A	G/G	Affx-108978979	5A	559064137
AX-109504449	C/C	C/C	C/C	Affx-110614709	5A	539539942
AX-109507034	A/A	A/A	A/A	Affx-110771741	5A	62981310
AX-109511993	T/T	T/C	C/C	Affx-111555685	5A	612720178
AX-109516719	T/T	T/C	T/T	Affx-109636157	5A	97770469
AX-109532937	A/G	G/G	G/G	Affx-109959225	5A	476223977
AX-109553748	C/C	C/C	C/C	Affx-111362623	5A	444938405
AX-109576537	G/G	A/G	G/G	Affx-108923408	5A	102213382
AX-109580026	T/T	T/C	C/C	Affx-111879458	5A	243479586
AX-109586376	G/G	A/G	G/G	Affx-110242269	5A	72485620
AX-109620306	T/T	C/C	T/T	Affx-110656379	5A	677128986
AX-109622137	G/G	G/G	T/T	Affx-109334792	5A	506645472
AX-109623110	A/A	A/G	G/G	Affx-111019618	5A	10623547
AX-109628516	T/T	T/C	T/T	Affx-111411665	5A	584520745
AX-109630394	T/T	A/T	T/T	Affx-111551565	5A	100538917
AX-109696789	T/C	T/C	T/C	Affx-88618449	5A	587437830
AX-109818442	G/G	G/G	G/G	Affx-88389869	5A	464475374
AX-109825110	A/A	A/G	A/A	Affx-110228141	5A	594211828
AX-109825580	G/G	A/A	A/A	Affx-110147844	5A	522112286
AX-109829013	A/A	A/G	A/A	Affx-109859447	5A	616545374
AX-109830349	G/G	A/G	G/G	Affx-111582460	5A	56318112
AX-109832565	G/G	A/G	G/G	Affx-109458400	5A	654382500
AX-109833049	G/G	A/G	G/G	Affx-109854318	5A	587869843
AX-109833294	A/A	C/C	C/C	Affx-110399847	5A	606967711
AX-109841387	T/T	T/C	T/T	Affx-110957546	5A	702987090
AX-109852151	A/A	A/G	G/G	Affx-111080311	5A	261204415
AX-109857378	A/A	A/G	A/A	Affx-111415398	5A	538327300
AX-109857944	G/G	T/G	G/G	Affx-110101681	5A	692461596
AX-109860167	A/A	G/G	G/G	Affx-109985655	5A	308341558
AX-109864923	A/G	G/G	A/A	Affx-110778823	5A	244703801
AX-109867475	T/T	T/G	T/T	Affx-111760138	5A	691026249
AX-109870540	T/T	T/C	C/C	Affx-110474252	5A	328919767
AX-109871542	A/A	A/G	G/G	Affx-88404303	5A	477785486
AX-109874877	T/T	T/T	T/T	Affx-109502266	5A	684629598
AX-109876198	C/C	C/C	C/C	Affx-109011688	5A	572814765
AX-109878757	C/C	T/C	C/C	Affx-111642655	5A	71232311
AX-109878883	C/C	T/C	C/C	Affx-110112164	5A	53473485

AX-109879648	G/G	G/G	G/G	Affx-110157913	5A	554188968
AX-109879982	C/C	C/C	C/C	Affx-111713694	5A	36827299
AX-109881260	T/T	T/C	C/C	Affx-110791953	5A	526259404
AX-109892357	G/G	G/G	A/A	Affx-110710443	5A	216407475
AX-109897379	G/G	A/A	A/A	Affx-109358419	5A	454825183
AX-109900224	G/G	A/G	G/G	Affx-109962980	5A	573095667
AX-109901231	C/C	T/C	C/C	Affx-110981764	5A	444920379
AX-109907593	T/T	T/C	C/C	Affx-110970446	5A	619099698
AX-109912402	G/G	T/G	G/G	Affx-110509049	5A	456429835
AX-109916379	T/T	T/C	C/C	Affx-111834058	5A	503865926
AX-109917367	T/T	T/C	T/T	Affx-112000563	5A	708725277
AX-109919064	T/T	T/C	T/T	Affx-111608318	5A	578959222
AX-109919444	C/C	C/C	C/C	Affx-109859272	5A	542151312
AX-109919516	C/C	C/C	C/C	Affx-88536207	5A	106148934
AX-109919761	C/C	C/G	C/C	Affx-111470781	5A	683124698
AX-109924811	G/G	G/G	G/G	Affx-110155014	5A	93736418
AX-109934620	G/G	A/G	G/G	Affx-110533348	5A	107749811
AX-109936458	G/G	T/G	T/T	Affx-110674419	5A	46425005
AX-109941244	C/C	T/C	C/C	Affx-108971181	5A	459485450
AX-109943089	T/T	T/C	T/T	Affx-110518087	5A	77358250
AX-109945330	A/A	A/G	G/G	Affx-110273745	5A	17520423
AX-109945932	G/G	A/G	A/A	Affx-110809886	5A	232080095
AX-109956967	T/T	T/C	T/T	Affx-111400488	5A	64986786
AX-109960078	A/A	A/G	A/A	Affx-110839656	5A	560459597
AX-109964203	C/C	T/C	C/C	Affx-88684604	5A	109287475
AX-109965739	C/C	C/G	C/C	Affx-109012573	5A	670565662
AX-109967839	C/C	T/C	C/C	Affx-109348605	5A	442836929
AX-109981086	A/A	A/G	A/A	Affx-109931403	5A	442512695
AX-109989907	G/G	A/G	G/G	Affx-109233690	5A	444895797
AX-109991177	G/G	A/G	G/G	Affx-111134104	5A	104866210
AX-109994159	G/G	C/G	C/C	Affx-110878878	5A	55168001
AX-109994203	G/G	C/G	G/G	Affx-109761363	5A	536681326
AX-109997529	T/T	T/C	C/C	Affx-109386192	5A	49435195
AX-110001618	G/G	A/G	G/G	Affx-111630119	5A	490365840
AX-110005779	T/T	C/C	T/T	Affx-110808350	5A	658799499
AX-110016633	C/C	T/C	C/C	Affx-109044741	5A	547333614
AX-110019103	T/T	T/C	C/C	Affx-110266213	5A	5434377
AX-110027672	T/T	T/C	T/T	Affx-109123123	5A	605679022
AX-110027939	T/C	T/T	T/T	Affx-111304889	5A	165737958
AX-110028038	G/G	A/G	A/A	Affx-109627005	5A	256670448
AX-110028503	G/G	A/G	G/G	Affx-111139658	5A	59172007
AX-110029324	A/A	A/G	A/A	Affx-111390101	5A	30054946
AX-110033406	C/C	T/C	C/C	Affx-111433854	5A	556443868
AX-110041070	A/C	A/C	A/C	Affx-109201005	5A	586728447
AX-110044116	G/G	C/G	G/G	Affx-111377954	5A	465644470
AX-110045446	A/A	A/C	A/A	Affx-110351301	5A	535369838
AX-110045648	A/G	A/G	A/G	Affx-111850157	5A	473327763
AX-110048492	C/C	T/C	C/C	Affx-111537965	5A	474482164
AX-110056403	T/T	T/C	T/T	Affx-88689822	5A	536679182
AX-110073980	T/G	T/T	T/T	Affx-108930260	5A	509655834
AX-110076201	G/G	A/G	G/G	Affx-88656258	5A	540609832
AX-110092724	T/C	T/T	T/T	Affx-88407835	5A	406836872
AX-110122130	C/C	T/C	C/C	Affx-111044052	5A	82467667
AX-110146194	G/G	C/G	C/C	Affx-111520863	5A	512195490

AX-110169414	G/G	G/G	G/G	Affx-109585867	5A	575262390
AX-110194823	A/A	A/T	A/A	Affx-109116035	5A	361900982
AX-110199675	T/T	C/C	C/C	Affx-110256718	5A	511102827
AX-110365198	C/C	T/C	C/C	Affx-109916691	5A	6084644
AX-110366508	C/C	C/C	C/C	Affx-109813330	5A	479403203
AX-110368031	A/A	A/G	A/A	Affx-109025585	5A	702466899
AX-110368939	C/C	T/C	C/C	Affx-111469493	5A	58440954
AX-110368941	G/G	G/G	C/C	Affx-88732112	5A	26960179
AX-110369666	G/G	A/G	A/A	Affx-109788010	5A	118254663
AX-110373741	T/T	T/C	T/C	Affx-110225543	5A	548680213
AX-110374482	G/G	A/G	G/G	Affx-88474183	5A	35654813
AX-110374864	C/C	T/C	C/C	Affx-109466637	5A	73825468
AX-110376060	G/G	A/G	A/A	Affx-111414676	5A	481458491
AX-110386126	T/T	T/C	C/C	Affx-111586703	5A	377927619
AX-110388895	G/G	A/G	A/A	Affx-110971098	5A	343446148
AX-110394129	A/A	A/G	A/A	Affx-109564761	5A	706648498
AX-110406334	G/G	G/G	G/G	Affx-109504204	5A	37824978
AX-110410060	A/A	A/G	A/A	Affx-110963471	5A	574403483
AX-110414471	C/C	T/T	T/T	Affx-111899236	5A	611824503
AX-110415476	G/G	A/G	G/G	Affx-111811408	5A	568548832
AX-110418098	C/C	C/G	C/C	Affx-110345320	5A	475750030
AX-110418848	T/T	T/C	T/T	Affx-111100100	5A	577886153
AX-110421211	T/T	T/C	T/T	Affx-110224306	5A	380821415
AX-110422901	G/G	A/G	A/A	Affx-111977185	5A	25874030
AX-110424170	C/C	C/C	C/C	Affx-109756598	5A	557077569
AX-110425408	A/A	A/A	A/A	Affx-88470017	5A	88566637
AX-110426047	C/C	T/C	T/T	Affx-88727152	5A	145553623
AX-110426159	C/C	T/C	C/C	Affx-108998872	5A	464167014
AX-110426820	T/T	A/T	A/A	Affx-109057230	5A	507253458
AX-110427839	C/C	T/C	C/C	Affx-109810211	5A	673536329
AX-110430275	G/G	A/A	A/A	Affx-110447138	5A	51031518
AX-110430512	C/C	T/C	C/C	Affx-109035727	5A	570021123
AX-110430693	C/C	A/C	C/C	Affx-109907690	5A	35765945
AX-110432795	A/G	A/G	A/G	Affx-88622552	5A	596664160
AX-110433682	C/C	T/C	C/C	Affx-111206278	5A	564757408
AX-110433931	A/A	C/C	A/A	Affx-111604628	5A	463094367
AX-110434245	T/T	T/C	T/T	Affx-111468861	5A	298528557
AX-110437938	C/C	T/C	T/T	Affx-109292290	5A	524726318
AX-110443555	T/T	T/C	T/T	Affx-110470754	5A	534823471
AX-110449012	A/A	A/G	A/A	Affx-111432515	5A	670140785
AX-110451753	C/C	A/C	C/C	Affx-111800069	5A	586727128
AX-110453518	A/A	A/G	A/A	Affx-109321255	5A	708314516
AX-110456271	G/G	G/G	G/G	Affx-110456541	5A	476688454
AX-110457073	A/A	A/C	C/C	Affx-110795154	5A	393395646
AX-110457864	A/A	A/G	A/A	Affx-109322559	5A	477288479
AX-110458003	G/G	G/G	A/A	Affx-111309411	5A	502169239
AX-110460509	G/G	A/G	G/G	Affx-110063758	5A	678977548
AX-110460868	G/G	A/G	G/G	Affx-110224608	5A	581363539
AX-110470253	C/C	A/C	C/C	Affx-108948242	5A	562203853
AX-110471491	A/A	A/G	G/G	Affx-109748242	5A	390232494
AX-110481423	C/C	T/C	T/T	Affx-111511107	5A	411083005
AX-110482398	G/G	A/G	G/G	Affx-110184367	5A	108268156
AX-110484973	T/T	T/C	C/C	Affx-111423092	5A	543911672
AX-110485698	G/G	C/G	G/G	Affx-110999667	5A	108858405

AX-110508884	T/T	T/T	T/T	Affx-88348001	5A	428416594
AX-110513721	A/A	A/G	A/A	Affx-110568670	5A	690418544
AX-110515826	C/C	C/C	C/C	Affx-110977966	5A	444926030
AX-110516969	G/G	A/A	G/G	Affx-111978282	5A	460678209
AX-110521294	C/C	C/C	C/C	Affx-109833752	5A	462577830
AX-110524682	A/A	A/G	G/G	Affx-109376878	5A	518429189
AX-110525389	T/T	T/C	T/T	Affx-111947173	5A	443776767
AX-110529159	A/A	A/G	A/A	Affx-110269710	5A	10203187
AX-110529756	C/C	C/C	T/T	Affx-109935691	5A	29377478
AX-110535857	A/A	A/T	A/A	Affx-109140129	5A	622780434
AX-110536448	A/G	A/G	A/G	Affx-109095924	5A	536677135
AX-110537939	A/A	A/G	A/A	Affx-109312308	5A	37389245
AX-110538331	T/T	T/C	T/T	Affx-111184326	5A	91132050
AX-110538697	A/A	A/C	A/A	Affx-111506046	5A	76738828
AX-110541739	C/C	T/T	C/C	Affx-109665469	5A	698209053
AX-110546974	C/C	G/G	C/C	Affx-110784310	5A	667927542
AX-110547149	A/A	A/C	A/A	Affx-109083463	5A	19113978
AX-110551710	A/A	A/C	A/A	Affx-108979655	5A	556001658
AX-110553919	T/T	G/G	G/G	Affx-111217864	5A	396492613
AX-110563308	T/T	T/T	T/T	Affx-110161942	5A	17300681
AX-110564755	G/G	A/G	G/G	Affx-111931974	5A	698183533
AX-110570771	G/G	G/G	G/G	Affx-109330240	5A	83292772
AX-110574166	A/G	A/A	A/A	Affx-110395545	5A	32661825
AX-110578543	A/A	G/G	G/G	Affx-110930037	5A	480403379
AX-110581880	C/C	T/C	T/T	Affx-110335903	5A	412321392
AX-110587222	T/T	T/G	T/T	Affx-109247486	5A	331392
AX-110590751	C/C	T/C	C/C	Affx-109250190	5A	353276171
AX-110598501	G/G	A/G	G/G	Affx-88626627	5A	380340238
AX-110598576	C/C	T/C	C/C	Affx-109352642	5A	576774973
AX-110607280	T/T	T/C	T/T	Affx-110102779	5A	34109310
AX-110628292	A/A	A/C	C/C	Affx-110598634	5A	383945012
AX-110632493	C/C	T/C	C/C	Affx-110491449	5A	444937582
AX-110650413	T/T	C/C	C/C	Affx-110461240	5A	3434997
AX-110651786	T/T	T/C	T/T	Affx-109781521	5A	685793781
AX-110656648	C/C	G/G	G/G	Affx-111698143	5A	450169210
AX-110657845	C/C	C/C	C/C	Affx-108922418	5A	550251710
AX-110679088	G/G	C/G	G/G	Affx-110687159	5A	474822591
AX-110683877	T/T	T/C	T/T	Affx-111022599	5A	457500291
AX-110688523	C/C	T/C	T/T	Affx-109801220	5A	24361344
AX-110716987	A/A	A/C	C/C	Affx-110044521	5A	551785323
AX-110905339	T/T	T/C	T/T	Affx-109433238	5A	51939813
AX-110906920	T/T	T/C	C/C	Affx-109778363	5A	293499679
AX-110910878	C/C	T/C	T/T	Affx-111018968	5A	8810419
AX-110914648	A/A	A/C	A/A	Affx-109340658	5A	541176314
AX-110916541	T/T	T/G	T/T	Affx-110085121	5A	109859447
AX-110917175	T/T	T/C	T/T	Affx-111208107	5A	696475570
AX-110920089	C/C	T/C	T/T	Affx-108860755	5A	57352367
AX-110925012	C/C	T/T	C/C	Affx-110806881	5A	657111831
AX-110932081	C/C	C/G	G/G	Affx-109139502	5A	25025225
AX-110938645	C/C	T/C	T/T	Affx-111701786	5A	496177952
AX-110946923	C/C	T/T	C/C	Affx-111054489	5A	590461191
AX-110948784	C/C	A/C	A/A	Affx-109807178	5A	455864945
AX-110950653	A/A	A/G	G/G	Affx-110601326	5A	550723509
AX-110950823	A/A	A/A	G/G	Affx-110582019	5A	290075298

AX-110954172	A/A	A/G	G/G	Affx-111939146	5A	385544630
AX-110964036	G/G	A/G	A/A	Affx-108983762	5A	18066270
AX-110964357	A/A	A/G	G/G	Affx-109962601	5A	451703153
AX-110964922	C/C	T/C	T/T	Affx-110149580	5A	500700301
AX-110965872	T/C	T/C	T/C	Affx-92739300	5A	559506284
AX-110976602	A/A	A/G	A/A	Affx-111572960	5A	585742226
AX-110980253	C/C	T/C	T/T	Affx-109942343	5A	28898294
AX-110980485	T/T	T/G	T/T	Affx-111011297	5A	700667998
AX-110981119	C/C	C/C	C/C	Affx-109324725	5A	704952069
AX-110982778	A/A	A/G	A/A	Affx-109279445	5A	443251970
AX-110982869	C/C	T/C	C/C	Affx-110713782	5A	19654675
AX-110983825	A/A	A/G	G/G	Affx-110706707	5A	502089387
AX-110984275	A/A	G/G	G/G	Affx-110866813	5A	517957811
AX-110987164	C/C	T/C	C/C	Affx-110431511	5A	617350592
AX-110988415	A/A	A/G	G/G	Affx-109690982	5A	620745312
AX-110990158	T/T	T/C	C/C	Affx-109310925	5A	3411983
AX-110990493	C/C	C/G	G/G	Affx-109306081	5A	536242120
AX-110994135	T/T	T/C	T/T	Affx-111975815	5A	698184633
AX-110996757	G/G	A/G	A/A	Affx-88556119	5A	51208781
AX-110997971	G/G	A/G	G/G	Affx-110021391	5A	510550289
AX-111000665	C/C	C/C	C/C	Affx-109265806	5A	444894278
AX-111001091	G/G	A/G	A/A	Affx-109244042	5A	530991655
AX-111012029	G/G	A/G	G/G	Affx-111481005	5A	413639279
AX-111013097	C/C	T/C	C/C	Affx-110979600	5A	96125310
AX-111013276	G/G	A/A	A/A	Affx-88396659	5A	506159283
AX-111015602	A/A	A/A	A/A	Affx-109828609	5A	44110354
AX-111015864	C/C	A/C	A/A	Affx-110172676	5A	528863448
AX-111018645	C/C	T/C	T/T	Affx-111156989	5A	337818963
AX-111024628	G/G	A/G	A/A	Affx-111870440	5A	228907545
AX-111029956	G/G	G/G	G/G	Affx-110868558	5A	470179348
AX-111034416	C/C	C/C	C/C	Affx-108869483	5A	108585283
AX-111040701	G/G	A/G	G/G	Affx-108998333	5A	85818937
AX-111048027	A/G	A/G	A/A	Affx-109514729	5A	651554409
AX-111054664	T/T	T/G	G/G	Affx-109198154	5A	525229881
AX-111055855	C/C	T/C	C/C	Affx-111270488	5A	667361118
AX-111058119	C/C	A/C	C/C	Affx-88744861	5A	74948600
AX-111079031	A/A	A/G	A/A	Affx-111558801	5A	559617604
AX-111082727	C/C	T/C	C/C	Affx-109601497	5A	161182908
AX-111091728	A/A	A/A	G/G	Affx-111988252	5A	622462067
AX-111093021	G/G	A/G	A/A	Affx-111749922	5A	32133664
AX-111093453	A/A	A/A	C/C	Affx-109506774	5A	520533782
AX-111100693	G/G	A/G	A/A	Affx-110611609	5A	505421598
AX-111103773	C/C	C/C	T/T	Affx-111679536	5A	527398278
AX-111111351	A/A	A/G	A/A	Affx-111220479	5A	427825736
AX-111118131	T/T	T/T	T/T	Affx-111290555	5A	420276818
AX-111122310	G/G	A/G	A/A	Affx-111251735	5A	511619430
AX-111127880	T/T	C/C	C/C	Affx-109773772	5A	385023826
AX-111130384	C/C	T/C	C/C	Affx-109131600	5A	607626250
AX-111133235	G/G	C/G	G/G	Affx-111150689	5A	52881872
AX-111136936	A/A	A/A	A/A	Affx-109017044	5A	63679557
AX-111145015	T/T	T/C	T/T	Affx-110836095	5A	437157171
AX-111160395	C/G	C/G	C/G	Affx-88411281	5A	587424336
AX-111168684	G/G	A/G	A/A	Affx-110621260	5A	527847987
AX-111172588	A/A	A/G	A/A	Affx-111449870	5A	430956942

AX-111215341	T/T	T/G	T/T	Affx-88612769	5A	697051126
AX-111218757	C/C	T/C	C/C	Affx-109090371	5A	204664927
AX-111219562	T/T	T/C	T/T	Affx-110597023	5A	66154347
AX-111223047	A/A	A/T	T/T	Affx-109012404	5A	631677274
AX-111233738	G/G	A/G	G/G	Affx-109455043	5A	86393054
AX-111236067	T/T	T/C	T/C	Affx-111536862	5A	27950908
AX-111282277	C/C	C/C	C/C	Affx-88650938	5A	701381964
AX-111449298	A/A	T/T	A/A	Affx-112002221	5A	108206151
AX-111449978	T/T	T/T	A/A	Affx-109381427	5A	522621692
AX-111451244	C/C	T/C	T/T	Affx-108986807	5A	402162143
AX-111461409	G/G	A/G	G/G	Affx-88521244	5A	92988011
AX-111470884	T/T	T/C	T/T	Affx-88786207	5A	109288979
AX-111476613	T/C	T/C	T/T	Affx-109007966	5A	665888551
AX-111477039	A/A	A/G	A/A	Affx-88470546	5A	416128813
AX-111478304	T/T	T/C	T/T	Affx-110649920	5A	78379748
AX-111480624	A/A	A/C	A/A	Affx-110265263	5A	679484960
AX-111481515	C/C	T/C	C/C	Affx-110009983	5A	319791164
AX-111485276	C/C	C/G	G/G	Affx-110144721	5A	531516700
AX-111485449	C/C	T/C	C/C	Affx-109014911	5A	80274728
AX-111492494	T/T	T/C	C/C	Affx-110283542	5A	394010749
AX-111495410	C/C	T/C	C/C	Affx-111931139	5A	82284691
AX-111498584	G/G	T/G	G/G	Affx-109510174	5A	103052316
AX-111507729	C/C	T/C	C/C	Affx-110134927	5A	436138873
AX-111509179	G/G	A/G	G/G	Affx-111692737	5A	31163746
AX-111513675	A/A	A/G	A/A	Affx-109724662	5A	586886517
AX-111516417	T/T	T/G	T/T	Affx-109129217	5A	624117710
AX-111520163	C/C	C/G	G/G	Affx-110840837	5A	18577515
AX-111520340	A/A	A/G	G/G	Affx-110329928	5A	526850602
AX-111521631	A/A	A/G	A/A	Affx-109443424	5A	459002338
AX-111523523	T/T	C/C	T/T	Affx-110080426	5A	62219169
AX-111524655	G/G	A/G	G/G	Affx-109768718	5A	686708702
AX-111541044	C/C	T/C	C/C	Affx-110302337	5A	67519552
AX-111545805	T/T	G/G	T/T	Affx-109010831	5A	555335583
AX-111546813	A/G	A/G	A/A	Affx-110845833	5A	392845494
AX-111551944	G/G	A/G	G/G	Affx-109095579	5A	381761150
AX-111556373	G/G	A/G	A/A	Affx-88432033	5A	610750842
AX-111556948	C/C	C/C	C/C	Affx-109024047	5A	107454528
AX-111570699	G/G	A/G	A/A	Affx-109504740	5A	533331233
AX-111579038	T/T	T/C	T/T	Affx-88639107	5A	445442064
AX-111581139	C/C	T/C	C/C	Affx-109684822	5A	107393647
AX-111585657	G/G	T/G	T/T	Affx-88770914	5A	608951130
AX-111597238	G/G	C/G	G/G	Affx-110631122	5A	454308608
AX-111600282	C/C	A/C	C/C	Affx-110563793	5A	552903000
AX-111621991	A/A	A/G	A/A	Affx-111991875	5A	594878944
AX-111623511	G/G	A/G	G/G	Affx-109591984	5A	101053846
AX-111623933	A/A	A/C	A/A	Affx-111086732	5A	65421416
AX-111624916	T/T	T/C	T/T	Affx-110191274	5A	535239365
AX-111629269	A/A	A/G	A/A	Affx-108912841	5A	587427902
AX-111630207	A/A	A/G	A/A	Affx-110754677	5A	673781092
AX-111649156	G/G	G/G	G/G	Affx-109253960	5A	701986622
AX-111659295	T/T	T/C	T/T	Affx-109661864	5A	599430066
AX-111673783	A/A	A/G	A/A	Affx-110135393	5A	650828497
AX-111709270	T/T	T/C	T/T	Affx-111763864	5A	570507143
AX-111712601	C/C	T/C	C/C	Affx-111412641	5A	563326364

AX-111745910	C/C	T/C	C/C	Affx-111586895	5A	682619279
AX-112286341	A/A	A/A	A/A	Affx-88483747	5A	574489275
AX-112287103	G/G	A/A	G/G	Affx-112312771	5A	692163603
AX-112291111	T/T	T/T	T/T	Affx-88788150	5A	442701260
AX-179476812	G/G	A/G	G/G	Affx-292486096	5A	480337525
AX-182022014	T/T	C/C	T/T	Affx-110364194	5A	536681055
AX-182032282	G/G	A/G	G/G	Affx-111880185	5A	108616630
AX-182059896	T/T	T/C	T/T	Affx-472304272	5A	698636823
AX-182097490	T/T	T/C	T/T	Affx-108917609	5A	444938439
AX-86160965	G/G	A/G	G/G	Affx-92532894	5A	536677319
AX-86162414	A/C	C/C	A/C	Affx-88714707	5A	588555578
AX-86166078	A/A	G/G	A/A	Affx-93016094	5A	487270581
AX-86169055	G/G	A/G	G/G	Affx-109678513	5A	108280585
AX-86172819	T/T	T/T	T/T	Affx-88391771	5A	631668459
AX-86175375	G/G	A/A	A/A	Affx-92098183	5A	8243625
AX-86177228	T/T	T/C	T/T	Affx-92187789	5A	702752999
AX-86179857	T/T	T/C	T/T	Affx-110656821	5A	445191816
AX-89579652	T/T	T/C	T/T	Affx-109764205	5A	653677804
AX-89694368	C/C	T/C	C/C	Affx-88727116	5A	452801856
AX-94420428	T/T	A/T	T/T	Affx-92887323	5A	570938053
AX-94456702	C/C	T/C	C/C	Affx-92524264	5A	706932704
AX-94466060	T/T	T/T	T/T	Affx-92103161	5A	445287948
AX-94483629	G/G	A/G	G/G	Affx-92348053	5A	659722172
AX-94487480	T/T	C/C	T/T	Affx-92394552	5A	492899485
AX-94492363	T/T	T/C	T/T	Affx-92915307	5A	478822209
AX-94527096	A/A	A/G	A/A	Affx-92885826	5A	467396884
AX-94566461	A/G	A/A	A/A	Affx-92561429	5A	269318869
AX-94567136	C/C	T/C	C/C	Affx-92705264	5A	68549924
AX-94578770	T/T	T/C	T/T	Affx-92564535	5A	536677475
AX-94589715	C/C	T/C	C/C	Affx-92488155	5A	546531060
AX-94625527	T/T	T/T	T/T	Affx-92162276	5A	536677389
AX-94712295	T/T	T/C	C/C	Affx-92474255	5A	14844490
AX-94763692	T/T	T/G	T/T	Affx-88600630	5A	619682348
AX-94793223	C/C	C/C	C/C	Affx-88744186	5A	649245238
AX-94796135	G/G	A/G	G/G	Affx-92262964	5A	624213725
AX-94797656	G/G	A/G	G/G	Affx-112312429	5A	479848393
AX-94863799	G/G	A/G	G/G	Affx-88806850	5A	438267359
AX-94866088	C/C	T/C	T/T	Affx-88475357	5A	7105956
AX-94879565	G/G	A/G	G/G	Affx-92987320	5A	574750020
AX-94956393	A/A	A/G	A/A	Affx-92890637	5A	409319968
AX-94962936	T/T	T/G	T/T	Affx-472291075	5A	4956563
AX-95024922	A/A	A/A	A/A	Affx-92895229	5A	693300884
AX-95133936	A/G	A/G	A/A	Affx-88436432	5A	445277514
AX-95148754	C/C	C/C	T/T	Affx-92747197	5A	476659584
AX-95177077	A/C	A/A	A/C	Affx-92818905	5A	465541364
AX-95213349	T/T	T/T	T/T	Affx-92203108	5A	685434007
AX-95220581	A/A	A/A	A/A	Affx-92655481	5A	635502449
AX-95628994	A/A	A/G	A/A	Affx-88646595	5A	41426391
AX-95631051	T/T	T/C	T/T	Affx-88429832	5A	555540548
AX-95634783	A/A	A/G	G/G	Affx-92997982	5A	29892424
AX-95657287	G/G	T/G	G/G	Affx-88617311	5A	698508164
AX-95658699	C/C	T/C	T/T	Affx-88700676	5A	481670337
AX-95659495	G/G	A/G	G/G	Affx-88410596	5A	36012373
AX-95660782	A/G	A/G	A/A	Affx-92746103	5A	536352784

AX-108725176	G/G	A/G	G/G	Affx-109663015	5B	432985093
AX-108729070	G/G	A/G	G/G	Affx-110727225	5B	406190132
AX-108730808	G/G	C/G	G/G	Affx-111798837	5B	607190622
AX-108733256	C/C	T/C	C/C	Affx-109148693	5B	702228057
AX-108733827	T/T	T/C	T/C	Affx-110037426	5B	697378921
AX-108734272	T/T	T/C	T/T	Affx-110805396	5B	484833933
AX-108735405	C/C	C/G	G/G	Affx-110803683	5B	317779854
AX-108739360	T/T	T/C	T/T	Affx-111605194	5B	619604268
AX-108739557	A/A	A/C	A/A	Affx-111557259	5B	214182236
AX-108740226	T/C	T/C	T/T	Affx-109795687	5B	573813518
AX-108742618	A/A	A/T	A/A	Affx-109202128	5B	601331137
AX-108745488	G/G	A/G	G/G	Affx-110306071	5B	516561605
AX-108747595	G/G	A/G	A/A	Affx-110654450	5B	42261339
AX-108748458	A/A	A/G	G/G	Affx-109628479	5B	596300111
AX-108748929	C/C	T/C	C/C	Affx-110335543	5B	391446098
AX-108749262	C/C	C/G	G/G	Affx-88431592	5B	15004677
AX-108753713	T/C	C/C	C/C	Affx-111666508	5B	16052441
AX-108757760	T/T	T/C	T/T	Affx-111667569	5B	701544672
AX-108766237	C/C	C/G	C/C	Affx-108882506	5B	100779983
AX-108769798	T/T	T/C	T/T	Affx-111730839	5B	11621790
AX-108771448	A/A	A/C	A/A	Affx-110134473	5B	389901454
AX-108772938	C/C	T/C	C/C	Affx-109303969	5B	696986301
AX-108775382	C/C	T/C	C/C	Affx-111000908	5B	245724943
AX-108775541	G/G	C/G	G/G	Affx-109658795	5B	394025627
AX-108779962	G/G	A/G	G/G	Affx-109087417	5B	140175380
AX-108780883	G/G	A/G	G/G	Affx-109989556	5B	411079760
AX-108781071	T/T	T/G	T/T	Affx-111075518	5B	671903746
AX-108781101	T/T	T/C	T/T	Affx-109051697	5B	537858631
AX-108790751	A/G	G/G	A/G	Affx-110495538	5B	508683189
AX-108792379	T/T	T/G	T/T	Affx-108955598	5B	556076407
AX-108796258	G/G	T/T	G/G	Affx-111418823	5B	429724591
AX-108797427	G/G	T/G	G/G	Affx-111213913	5B	591586440
AX-108800607	A/A	A/G	A/A	Affx-110081816	5B	479496812
AX-108803584	C/C	T/T	C/C	Affx-110827475	5B	707261636
AX-108803769	A/A	A/G	A/A	Affx-111802817	5B	399233027
AX-108806076	G/G	T/G	G/G	Affx-111091690	5B	622737777
AX-108807464	T/T	T/T	C/C	Affx-110379085	5B	567104833
AX-108811852	C/C	T/C	T/T	Affx-111184228	5B	74507442
AX-108815271	C/C	T/C	T/T	Affx-111358985	5B	72588767
AX-108816702	G/G	A/G	G/G	Affx-111499064	5B	308265587
AX-108823103	G/G	A/G	A/A	Affx-111102421	5B	460246458
AX-108827033	G/G	A/G	G/G	Affx-109295459	5B	422218746
AX-108832106	C/C	T/C	T/T	Affx-108981920	5B	308576144
AX-108835880	A/A	A/G	A/A	Affx-111530498	5B	558281698
AX-108837732	T/T	T/C	T/T	Affx-109919570	5B	37260278
AX-108845888	G/G	G/G	G/G	Affx-110953684	5B	145727504
AX-108851631	C/C	T/C	C/C	Affx-109892399	5B	34377722
AX-108851808	A/A	A/G	A/A	Affx-110481608	5B	238055872
AX-108854304	C/C	T/C	C/C	Affx-111675449	5B	54335855
AX-108855275	G/G	T/G	G/G	Affx-109163635	5B	573617887
AX-108856729	T/T	T/C	T/T	Affx-88789006	5B	224625276
AX-108857064	T/C	C/C	T/C	Affx-109015819	5B	577844719
AX-108863692	A/A	A/G	A/A	Affx-110668959	5B	410266474
AX-108866616	A/G	G/G	G/G	Affx-110010942	5B	304819539

AX-108867348	T/T	T/C	T/T	Affx-111408035	5B	12965893
AX-108868834	A/A	A/G	A/A	Affx-111981721	5B	466540302
AX-108872409	A/A	A/G	G/G	Affx-110393216	5B	582744226
AX-108875349	T/T	T/C	C/C	Affx-109242292	5B	561247273
AX-108881152	G/G	A/G	G/G	Affx-110285385	5B	38385087
AX-108885876	A/G	A/G	A/G	Affx-110147404	5B	414442926
AX-108890871	C/C	C/G	C/C	Affx-109752778	5B	227344532
AX-108892257	T/T	T/C	T/T	Affx-110758629	5B	463228760
AX-108895416	T/T	T/C	T/T	Affx-109643176	5B	701018831
AX-108897432	T/T	T/C	C/C	Affx-109825947	5B	567669260
AX-108898461	A/A	A/G	G/G	Affx-109129811	5B	568574170
AX-108899793	C/C	T/C	C/C	Affx-111310570	5B	29124054
AX-108903666	G/G	A/G	G/G	Affx-110646000	5B	605514264
AX-108917213	G/G	A/G	A/G	Affx-109563554	5B	683595554
AX-108918994	C/C	A/C	A/C	Affx-111930397	5B	574104474
AX-108926511	C/C	A/C	C/C	Affx-110387696	5B	482518147
AX-108930395	T/T	C/C	T/T	Affx-109871090	5B	642118454
AX-108933291	T/T	T/G	T/G	Affx-88369772	5B	60826494
AX-108937379	G/G	A/G	G/G	Affx-88433496	5B	216860872
AX-108937604	A/A	A/G	G/G	Affx-109390286	5B	659472188
AX-108937690	G/G	A/G	A/A	Affx-110250913	5B	324414358
AX-108938824	C/C	A/C	C/C	Affx-111586820	5B	230816937
AX-108943229	C/C	T/C	C/C	Affx-111517555	5B	650408373
AX-108943772	G/G	C/G	G/G	Affx-109085541	5B	516366829
AX-108948115	T/T	T/C	T/T	Affx-109073752	5B	585825985
AX-108956203	T/T	T/C	C/C	Affx-111168630	5B	300156970
AX-108956314	G/G	A/G	G/G	Affx-111242541	5B	544673479
AX-108959579	C/C	G/G	C/G	Affx-88760498	5B	73478328
AX-108959845	T/T	G/G	G/G	Affx-111903231	5B	69850481
AX-108960940	T/T	T/C	C/C	Affx-88730182	5B	18778888
AX-108961268	G/G	A/G	G/G	Affx-109811112	5B	496987522
AX-108963974	C/C	A/C	C/C	Affx-109816705	5B	121751365
AX-108976112	G/G	T/G	G/G	Affx-88734197	5B	536236535
AX-108982572	C/C	C/G	C/C	Affx-110627032	5B	297076467
AX-108983046	A/A	A/C	A/A	Affx-109939807	5B	111684696
AX-108988155	G/G	A/G	G/G	Affx-111439017	5B	469120752
AX-109007669	C/C	C/G	G/G	Affx-109468030	5B	20014173
AX-109008593	T/T	T/T	C/C	Affx-109369177	5B	382910290
AX-109034524	G/G	C/G	G/G	Affx-111744990	5B	620435895
AX-109038646	T/T	C/C	T/T	Affx-109221829	5B	56902675
AX-109055754	C/C	A/C	A/A	Affx-111212122	5B	566536547
AX-109057599	A/A	A/G	A/A	Affx-110443171	5B	404016938
AX-109057609	G/G	A/G	A/A	Affx-110714459	5B	573781537
AX-109095411	G/G	G/G	G/G	Affx-110949388	5B	397234517
AX-109101238	C/C	T/C	C/C	Affx-109248779	5B	229964241
AX-109104260	C/C	T/C	C/C	Affx-111709506	5B	564092537
AX-109109180	C/C	T/T	C/C	Affx-109867144	5B	672367638
AX-109271670	G/G	A/G	G/G	Affx-109657200	5B	613456433
AX-109276499	A/A	C/C	C/C	Affx-110298643	5B	475521001
AX-109277469	G/G	G/G	G/G	Affx-111855994	5B	573497826
AX-109278051	G/G	A/G	A/A	Affx-111147509	5B	69179984
AX-109283196	C/C	C/C	A/A	Affx-111616219	5B	65697652
AX-109284684	A/A	A/G	A/A	Affx-110755825	5B	418309355
AX-109289769	C/C	T/C	C/C	Affx-108857075	5B	241602012

AX-109290755	C/C	T/C	C/C	Affx-109364917	5B	256952409
AX-109297817	T/T	T/C	T/T	Affx-109175947	5B	17177
AX-109298064	C/C	T/C	T/T	Affx-109197158	5B	297873574
AX-109298854	T/T	C/C	T/T	Affx-109029827	5B	551492867
AX-109301146	C/C	A/C	A/A	Affx-108857433	5B	279299841
AX-109302342	A/G	A/G	A/G	Affx-109467173	5B	357312386
AX-109307624	G/G	C/G	G/G	Affx-109361820	5B	490992801
AX-109321092	G/G	G/G	G/G	Affx-110571936	5B	243404910
AX-109322756	T/C	C/C	T/C	Affx-111449752	5B	652933087
AX-109323554	C/C	C/G	C/C	Affx-110048721	5B	403547127
AX-109329070	C/C	T/C	C/C	Affx-109456700	5B	447561698
AX-109330727	C/C	T/C	C/C	Affx-111844011	5B	353436493
AX-109335246	C/C	T/C	C/C	Affx-109586315	5B	549778567
AX-109338184	G/G	C/C	G/G	Affx-109121727	5B	609138849
AX-109339278	C/C	T/C	C/C	Affx-88367941	5B	129335519
AX-109339994	C/C	C/G	G/G	Affx-109621532	5B	43844720
AX-109348429	G/G	A/A	G/G	Affx-110349783	5B	404478483
AX-109349444	T/T	T/C	T/T	Affx-109718772	5B	499162826
AX-109350844	T/T	T/C	T/T	Affx-110811155	5B	413371882
AX-109353092	C/C	G/G	G/G	Affx-111294334	5B	43156367
AX-109353285	G/G	A/A	G/G	Affx-88768586	5B	396152744
AX-109355082	C/C	T/C	C/C	Affx-109250468	5B	281575640
AX-109356727	C/C	T/C	C/C	Affx-109728537	5B	62017016
AX-109360435	T/T	T/C	T/T	Affx-111435491	5B	500531363
AX-109363015	C/C	C/C	A/C	Affx-108874036	5B	174925421
AX-109376938	A/A	A/G	G/G	Affx-111708229	5B	312752972
AX-109377789	G/G	T/G	G/G	Affx-108874804	5B	56265351
AX-109381907	G/G	A/G	G/G	Affx-109882484	5B	68897604
AX-109386760	T/T	T/C	T/T	Affx-110218005	5B	692423831
AX-109386764	G/G	C/G	G/G	Affx-110684992	5B	8008893
AX-109390686	C/C	T/C	C/C	Affx-110794384	5B	189196757
AX-109395179	T/T	T/G	T/T	Affx-111537119	5B	593144668
AX-109400878	C/C	T/C	C/C	Affx-109057531	5B	507867002
AX-109404412	C/C	T/C	C/C	Affx-111604491	5B	591134765
AX-109407188	A/A	A/G	A/A	Affx-110479162	5B	615005628
AX-109408540	G/G	A/G	G/G	Affx-111303115	5B	151097830
AX-109411601	A/A	C/C	A/A	Affx-110371746	5B	511852027
AX-109412196	T/T	T/C	T/T	Affx-111940595	5B	11042453
AX-109415750	G/G	T/G	T/T	Affx-111720740	5B	74994463
AX-109415761	A/A	G/G	A/A	Affx-110928918	5B	399772234
AX-109419514	A/A	A/A	A/T	Affx-111985611	5B	72081930
AX-109421571	C/C	T/C	C/C	Affx-109181134	5B	482840179
AX-109429662	C/C	T/C	T/T	Affx-109446337	5B	49549251
AX-109430842	G/G	A/G	A/A	Affx-110020867	5B	305440381
AX-109431199	T/T	T/C	C/C	Affx-88658894	5B	57872375
AX-109433781	T/T	T/C	C/C	Affx-110683437	5B	47568031
AX-109434754	T/T	T/C	C/C	Affx-111327870	5B	560843923
AX-109441188	C/C	T/C	C/C	Affx-111338011	5B	387765973
AX-109449956	A/A	A/T	A/A	Affx-111106441	5B	154195762
AX-109455413	G/G	C/G	C/C	Affx-110351912	5B	558703913
AX-109460332	T/T	T/C	C/C	Affx-111722179	5B	458140438
AX-109466200	T/T	T/C	T/C	Affx-110838407	5B	63084542
AX-109473769	C/C	C/G	G/G	Affx-109648952	5B	61061996
AX-109475855	A/A	A/G	A/A	Affx-109227774	5B	646264944

AX-109476383	A/A	A/G	A/A	Affx-88402126	5B	1436163
AX-109479506	A/A	A/G	A/A	Affx-108927927	5B	690894208
AX-109480198	T/T	A/T	T/T	Affx-109418793	5B	643669795
AX-109488094	T/T	T/G	T/T	Affx-111063759	5B	615552723
AX-109496576	C/C	C/C	C/C	Affx-110652169	5B	411742999
AX-109496894	A/A	A/G	A/A	Affx-111703756	5B	7736591
AX-109500107	G/G	A/G	G/G	Affx-109204945	5B	497375926
AX-109518102	C/C	T/C	C/C	Affx-111558774	5B	61519806
AX-109520052	T/T	T/C	T/T	Affx-109717334	5B	605168108
AX-109520682	A/A	A/A	A/A	Affx-110555217	5B	680525930
AX-109526372	C/C	T/C	T/C	Affx-109437365	5B	498264848
AX-109538613	C/C	T/C	C/C	Affx-109869443	5B	29430400
AX-109549994	G/G	C/G	G/G	Affx-111952800	5B	501954912
AX-109556435	C/C	T/C	C/C	Affx-110157106	5B	512246353
AX-109566896	G/G	A/G	G/G	Affx-111528231	5B	590530563
AX-109567220	G/G	T/G	G/G	Affx-111265789	5B	480164070
AX-109581281	A/A	A/A	A/A	Affx-111639186	5B	437250895
AX-109581384	C/C	G/G	C/C	Affx-111866476	5B	579078809
AX-109581524	G/G	A/G	A/A	Affx-110361804	5B	250568458
AX-109581594	G/G	A/G	G/G	Affx-110962092	5B	126474838
AX-109584999	G/G	A/G	G/G	Affx-109950742	5B	577511935
AX-109603392	G/G	A/G	G/G	Affx-111263156	5B	127494030
AX-109616109	A/A	A/G	A/A	Affx-111392592	5B	73969695
AX-109651650	G/G	G/G	G/G	Affx-109409544	5B	549635972
AX-109655279	A/A	A/A	A/C	Affx-110296747	5B	665369153
AX-109725037	G/G	G/G	G/G	Affx-88392849	5B	126443500
AX-109815720	A/A	A/G	G/G	Affx-109390282	5B	326795120
AX-109819063	C/C	T/C	C/C	Affx-110985065	5B	550923097
AX-109820610	G/G	C/C	G/G	Affx-111547913	5B	518570389
AX-109827342	T/T	T/C	C/C	Affx-110380060	5B	661206166
AX-109833911	C/C	T/C	C/C	Affx-111269670	5B	586273870
AX-109837402	A/A	A/G	A/A	Affx-110173798	5B	400770851
AX-109843200	T/T	T/C	T/T	Affx-110215512	5B	327816328
AX-109843548	A/A	A/G	A/A	Affx-109666914	5B	471400496
AX-109847779	G/G	A/G	G/G	Affx-110194519	5B	438162503
AX-109850301	G/G	T/G	T/T	Affx-110935426	5B	71802763
AX-109853609	A/A	A/C	A/A	Affx-111324608	5B	588503041
AX-109858007	G/G	A/A	G/G	Affx-110296903	5B	431734637
AX-109872176	C/C	C/G	G/G	Affx-110218656	5B	285579010
AX-109887486	G/G	C/G	C/C	Affx-111831826	5B	589029765
AX-109896842	A/A	A/G	G/G	Affx-109127268	5B	41339124
AX-109900605	G/G	T/G	G/G	Affx-88475387	5B	64717145
AX-109901256	G/G	A/G	G/G	Affx-88558427	5B	401396289
AX-109901647	C/C	C/C	C/C	Affx-110692488	5B	531809901
AX-109903615	T/T	T/C	T/T	Affx-108879092	5B	505474703
AX-109921910	G/G	A/G	G/G	Affx-110494974	5B	684787299
AX-109923012	G/G	T/G	G/G	Affx-111805008	5B	604140500
AX-109928742	C/C	C/G	C/C	Affx-109282260	5B	394970067
AX-109932243	T/T	T/C	T/T	Affx-111778356	5B	182802694
AX-109936301	C/C	T/C	C/C	Affx-109012041	5B	416436584
AX-109936387	T/T	T/C	T/T	Affx-109112188	5B	315169138
AX-109947820	G/G	A/G	A/A	Affx-111262918	5B	310066563
AX-109964143	A/G	A/G	A/G	Affx-110911363	5B	415526589
AX-109969080	T/C	T/T	T/C	Affx-88371129	5B	157026923

AX-109969417	C/C	T/C	C/C	Affx-111198560	5B	171215533
AX-109979591	A/A	A/G	A/A	Affx-109752659	5B	220565003
AX-109981797	C/C	A/C	C/C	Affx-111923781	5B	40558070
AX-109983932	T/T	C/C	T/T	Affx-110196689	5B	604605522
AX-109987949	G/G	T/G	G/G	Affx-109019807	5B	467040785
AX-110002541	C/C	C/C	C/C	Affx-111700591	5B	242705963
AX-110016103	G/G	A/G	G/G	Affx-109966242	5B	610005233
AX-110020239	C/C	A/C	C/C	Affx-109445883	5B	364582696
AX-110020378	A/A	A/G	G/G	Affx-110298362	5B	379205423
AX-110020680	C/C	T/C	T/T	Affx-109898000	5B	251008827
AX-110022194	G/G	C/G	C/C	Affx-109553660	5B	678319870
AX-110029474	G/G	C/C	G/G	Affx-110093730	5B	501434030
AX-110031309	T/T	T/C	C/C	Affx-88345809	5B	584820661
AX-110032721	A/A	A/G	G/G	Affx-109655050	5B	83536643
AX-110036687	T/T	T/T	T/T	Affx-111946493	5B	409616449
AX-110037893	C/C	T/C	C/C	Affx-110101179	5B	455331376
AX-110042137	T/T	T/C	T/T	Affx-111650130	5B	7351805
AX-110046102	T/T	T/G	T/T	Affx-110575809	5B	634454038
AX-110048627	G/G	A/G	G/G	Affx-110173411	5B	573497741
AX-110049382	A/A	A/G	A/A	Affx-111310576	5B	517558688
AX-110054717	G/G	A/G	G/G	Affx-88615227	5B	406534932
AX-110071375	C/C	T/C	C/C	Affx-111740709	5B	354982771
AX-110072054	C/C	C/G	G/G	Affx-111393779	5B	681814959
AX-110091845	G/G	A/G	G/G	Affx-110487066	5B	634992395
AX-110092496	A/A	A/G	G/G	Affx-111119446	5B	78716064
AX-110094230	T/T	T/C	T/T	Affx-109317343	5B	580117291
AX-110108283	G/G	T/G	G/G	Affx-110708082	5B	435767356
AX-110125113	C/C	A/C	A/A	Affx-110563376	5B	574637997
AX-110143600	C/C	T/C	C/C	Affx-109117957	5B	155429885
AX-110146214	A/A	A/G	A/A	Affx-110416713	5B	652456380
AX-110195720	T/T	T/C	T/T	Affx-111302264	5B	179792373
AX-110200916	A/A	A/G	G/G	Affx-110657176	5B	311780438
AX-110363233	C/C	T/C	C/C	Affx-110520667	5B	487272756
AX-110376465	T/T	T/C	T/T	Affx-111525607	5B	241913753
AX-110377728	T/T	T/C	T/T	Affx-109807333	5B	127204048
AX-110386773	C/C	A/C	C/C	Affx-110352759	5B	641060386
AX-110391473	C/C	A/C	C/C	Affx-111369412	5B	333251442
AX-110396006	A/G	G/G	A/G	Affx-110808551	5B	28213204
AX-110398218	C/C	C/C	G/G	Affx-111481852	5B	57493343
AX-110398818	G/G	A/G	G/G	Affx-88648582	5B	121674070
AX-110399526	C/C	T/C	C/C	Affx-110139923	5B	682701383
AX-110403012	A/A	A/G	A/A	Affx-109491806	5B	552631894
AX-110405101	G/G	T/G	G/G	Affx-111204513	5B	66770721
AX-110406892	G/G	C/G	G/G	Affx-110166485	5B	553926370
AX-110410093	C/C	G/G	G/G	Affx-109973823	5B	459188143
AX-110414818	T/T	T/C	C/C	Affx-111697723	5B	102825583
AX-110420308	C/C	A/C	C/C	Affx-110285738	5B	268238887
AX-110421918	G/G	C/G	G/G	Affx-109451103	5B	490852219
AX-110422257	C/C	C/G	C/C	Affx-110201188	5B	37595552
AX-110423832	T/C	T/C	T/T	Affx-109825773	5B	595223778
AX-110423840	C/C	T/T	C/C	Affx-109738628	5B	502474886
AX-110428884	C/C	C/C	C/C	Affx-110315357	5B	503225188
AX-110432249	T/T	T/G	T/T	Affx-111467224	5B	482170109
AX-110433306	A/A	A/G	A/A	Affx-109034873	5B	600364575

AX-110438459	A/A	A/G	A/A	Affx-109367469	5B	669152047
AX-110438555	G/G	A/G	G/G	Affx-110967519	5B	359132611
AX-110439426	T/T	T/C	T/T	Affx-111843923	5B	521014241
AX-110456893	C/C	C/C	T/C	Affx-109305201	5B	65024995
AX-110458449	G/G	A/G	G/G	Affx-108974071	5B	34928309
AX-110459842	T/T	T/C	T/T	Affx-109662804	5B	689929829
AX-110460557	C/C	C/G	C/C	Affx-111938071	5B	431071423
AX-110464694	C/C	T/C	T/T	Affx-111730432	5B	296558740
AX-110467281	T/T	T/C	T/T	Affx-111079290	5B	279597853
AX-110469875	C/C	T/C	C/C	Affx-109668139	5B	579714349
AX-110476873	T/T	C/C	T/T	Affx-111502872	5B	463805727
AX-110482128	G/G	T/G	G/G	Affx-110678074	5B	485874021
AX-110485194	G/G	A/G	A/A	Affx-110551280	5B	571221629
AX-110486751	T/T	T/C	C/C	Affx-110455092	5B	301511779
AX-110490429	T/T	T/C	T/T	Affx-110439922	5B	575244362
AX-110494074	C/C	C/C	C/C	Affx-111934018	5B	231945840
AX-110500869	T/T	T/G	G/G	Affx-110501469	5B	63612215
AX-110503446	A/A	A/A	A/A	Affx-109373838	5B	196209958
AX-110506915	G/G	A/G	G/G	Affx-109287980	5B	473349466
AX-110506922	T/T	T/G	T/T	Affx-111503052	5B	392898707
AX-110516200	G/G	T/G	T/T	Affx-111158002	5B	277898057
AX-110519421	C/C	T/C	T/T	Affx-109973635	5B	41671830
AX-110529376	C/C	C/C	T/C	Affx-109984024	5B	264839983
AX-110532317	A/A	A/G	A/A	Affx-111180376	5B	486895975
AX-110534757	G/G	A/G	G/G	Affx-109892841	5B	573781939
AX-110534995	T/T	T/C	T/T	Affx-111772612	5B	466045610
AX-110538245	C/C	T/C	C/C	Affx-111038377	5B	169746684
AX-110542749	G/G	A/G	G/G	Affx-110207581	5B	416971022
AX-110543532	G/G	A/G	G/G	Affx-110103906	5B	427002693
AX-110547223	C/C	A/C	A/C	Affx-109326150	5B	583723964
AX-110554992	C/C	T/C	T/T	Affx-111830229	5B	659747810
AX-110557910	C/C	C/G	C/C	Affx-110020464	5B	671186480
AX-110558483	G/G	A/G	A/A	Affx-109651200	5B	262756054
AX-110560278	T/T	T/T	T/T	Affx-110217997	5B	423765353
AX-110561585	C/C	T/C	T/T	Affx-109302869	5B	35291342
AX-110562707	C/C	C/C	C/C	Affx-110077461	5B	323708005
AX-110563039	C/C	T/C	C/C	Affx-111280064	5B	269294177
AX-110565581	A/A	A/G	G/G	Affx-110083335	5B	456584616
AX-110567266	C/C	C/G	G/G	Affx-110780308	5B	58422632
AX-110568989	T/T	T/C	C/C	Affx-109300996	5B	259728350
AX-110572579	C/C	T/C	T/T	Affx-111890281	5B	442858727
AX-110574387	C/C	T/T	C/C	Affx-111860904	5B	173399995
AX-110576628	C/C	T/C	C/C	Affx-111202556	5B	420011554
AX-110586527	G/G	T/T	T/T	Affx-111066593	5B	317993039
AX-110600267	T/T	T/C	C/C	Affx-111867268	5B	571806177
AX-110608136	G/G	A/G	G/G	Affx-109687411	5B	454650013
AX-110609591	G/G	G/G	G/G	Affx-111545164	5B	206888469
AX-110614697	C/C	A/C	C/C	Affx-110187890	5B	710482711
AX-110614986	C/C	T/C	C/C	Affx-109210710	5B	145930173
AX-110621432	C/C	T/C	C/C	Affx-109214248	5B	462317161
AX-110624064	G/G	A/G	A/G	Affx-109097089	5B	263932139
AX-110624606	T/T	T/C	T/T	Affx-111537470	5B	508683139
AX-110639732	G/G	A/G	A/A	Affx-109846986	5B	228815862
AX-110671838	A/A	A/G	A/A	Affx-108926407	5B	649923509

AX-110674058	C/C	A/A	C/C	Affx-111786749	5B	155847180
AX-110678580	G/G	A/G	A/A	Affx-111505206	5B	46281677
AX-110688632	G/G	A/G	G/G	Affx-109287456	5B	235264733
AX-110696432	T/T	T/C	C/C	Affx-111954760	5B	84980810
AX-110703046	A/C	A/A	A/C	Affx-109718839	5B	415738957
AX-110705974	G/G	G/G	A/A	Affx-108876789	5B	64071548
AX-110730995	G/G	A/G	A/A	Affx-109991952	5B	249843782
AX-110735496	T/T	T/C	T/T	Affx-109063329	5B	226530925
AX-110742930	A/A	G/G	A/A	Affx-111413812	5B	472138311
AX-110746264	C/C	T/C	C/C	Affx-108917982	5B	450946550
AX-110908996	G/G	A/G	A/A	Affx-109863660	5B	70606547
AX-110913121	C/C	T/C	C/C	Affx-109357154	5B	598056192
AX-110914955	A/A	A/G	A/A	Affx-110475813	5B	599617983
AX-110923135	G/G	G/G	G/G	Affx-110833683	5B	30025200
AX-110924699	C/C	T/C	C/C	Affx-111415389	5B	702477560
AX-110933976	G/G	A/G	G/G	Affx-109298743	5B	133005470
AX-110935192	C/C	T/C	T/T	Affx-109965576	5B	271106945
AX-110937447	A/A	A/G	A/A	Affx-109588697	5B	611936084
AX-110939639	T/T	T/C	C/C	Affx-109859590	5B	673038030
AX-110942404	G/G	A/G	G/G	Affx-111305350	5B	643270382
AX-110942939	A/A	A/G	G/G	Affx-111167502	5B	451142150
AX-110946044	A/A	A/G	A/A	Affx-108975737	5B	426067392
AX-110947245	A/C	A/A	A/A	Affx-109473228	5B	570371405
AX-110948117	A/A	A/C	A/A	Affx-111527474	5B	538599096
AX-110950304	A/G	G/G	A/G	Affx-110549155	5B	425558106
AX-110953444	G/G	G/G	T/T	Affx-88703908	5B	666582500
AX-110959860	T/T	T/C	C/C	Affx-110994124	5B	560382358
AX-110968967	C/C	T/C	C/C	Affx-111932311	5B	394537477
AX-110970244	C/C	T/C	C/C	Affx-108892322	5B	36737758
AX-110974449	A/A	A/G	A/A	Affx-109405226	5B	582396625
AX-110974598	A/A	A/C	A/A	Affx-111469948	5B	461287290
AX-110978478	A/G	G/G	A/G	Affx-110100134	5B	223092073
AX-110979631	G/G	A/G	G/G	Affx-111046385	5B	402955204
AX-110980136	C/C	T/C	T/T	Affx-111780835	5B	318991202
AX-110990032	T/C	C/C	C/C	Affx-110590604	5B	660538357
AX-110995712	T/T	T/C	T/T	Affx-111991536	5B	23550308
AX-110997674	A/A	A/T	A/A	Affx-111241111	5B	594725651
AX-110999470	A/A	A/G	G/G	Affx-88423242	5B	87978405
AX-110999858	G/G	C/G	G/G	Affx-109365212	5B	668249812
AX-111008799	G/G	A/G	G/G	Affx-110647408	5B	398654546
AX-111010202	A/A	G/G	A/A	Affx-109069939	5B	639097242
AX-111011401	T/T	T/C	C/C	Affx-111781242	5B	447452862
AX-111012251	A/A	A/G	G/G	Affx-111413847	5B	679678760
AX-111013279	C/C	T/C	C/C	Affx-111454930	5B	219279347
AX-111013696	G/G	A/G	G/G	Affx-111729610	5B	410725022
AX-111017571	A/A	A/G	G/G	Affx-109860124	5B	79388047
AX-111020018	C/C	A/C	C/C	Affx-111206191	5B	687993424
AX-111022645	G/G	C/G	C/C	Affx-109570800	5B	75707984
AX-111030246	T/T	T/C	T/T	Affx-110682828	5B	429778218
AX-111030750	G/G	A/G	A/A	Affx-111867419	5B	311202620
AX-111031162	A/A	A/G	A/A	Affx-110562855	5B	557492
AX-111031411	G/G	G/G	G/G	Affx-109483586	5B	663739754
AX-111031509	C/C	T/C	T/T	Affx-109594409	5B	260577074
AX-111034587	C/C	C/G	C/C	Affx-109290707	5B	424677753

AX-111039513	G/G	A/G	G/G	Affx-110433076	5B	402128748
AX-111039767	A/A	A/G	A/A	Affx-111367397	5B	398006525
AX-111044647	G/G	A/A	G/G	Affx-111160470	5B	696192226
AX-111045033	C/G	C/C	C/C	Affx-109066243	5B	562749021
AX-111045042	T/T	T/G	T/T	Affx-110953900	5B	332327953
AX-111047334	C/C	T/C	C/C	Affx-111879051	5B	338953401
AX-111051645	T/T	T/G	T/T	Affx-110758094	5B	462851053
AX-111054061	C/C	C/C	C/C	Affx-111221194	5B	620824847
AX-111055724	C/C	C/G	C/C	Affx-109608165	5B	918659
AX-111060117	T/T	T/G	G/G	Affx-109179939	5B	96367597
AX-111062734	T/T	T/C	T/T	Affx-109608314	5B	149022438
AX-111065683	A/A	A/C	A/A	Affx-111039810	5B	503787828
AX-111069134	A/A	A/G	A/A	Affx-109580794	5B	421692330
AX-111073870	G/G	A/G	G/G	Affx-109759447	5B	222111171
AX-111074180	A/G	A/G	G/G	Affx-110184295	5B	706151181
AX-111078255	G/G	A/G	A/A	Affx-108859912	5B	86045179
AX-111090273	T/T	T/C	T/T	Affx-109250620	5B	600055829
AX-111095318	A/A	A/G	G/G	Affx-111887067	5B	287378000
AX-111097453	G/G	A/G	A/A	Affx-109764895	5B	557751191
AX-111107210	C/C	T/C	C/C	Affx-110916394	5B	355233859
AX-111107749	A/C	C/C	C/C	Affx-110390233	5B	81799202
AX-111108344	T/T	T/C	T/T	Affx-109642902	5B	481138494
AX-111109533	C/C	A/C	C/C	Affx-111203488	5B	691983845
AX-111112569	T/T	T/C	T/C	Affx-109216283	5B	91108574
AX-111116821	T/T	T/C	T/T	Affx-109972401	5B	655006775
AX-111124661	G/G	A/G	G/G	Affx-88477908	5B	437564786
AX-111125347	G/G	G/G	A/A	Affx-109911550	5B	313692393
AX-111130924	G/G	A/G	G/G	Affx-109521706	5B	616028158
AX-111135061	G/G	A/G	A/A	Affx-109068586	5B	314482454
AX-111138800	C/C	T/C	T/T	Affx-109064849	5B	149798202
AX-111143503	C/C	T/C	T/T	Affx-111397392	5B	86542400
AX-111152483	C/C	T/C	C/C	Affx-109718898	5B	635738462
AX-111157380	T/T	T/C	C/C	Affx-111815303	5B	59518216
AX-111160435	T/T	T/T	C/C	Affx-111098724	5B	247055611
AX-111170049	T/T	T/C	T/T	Affx-110095657	5B	8933428
AX-111183263	T/T	G/G	T/T	Affx-109087283	5B	435156158
AX-111194981	C/C	T/C	T/T	Affx-88391986	5B	79763489
AX-111212757	T/T	A/T	T/T	Affx-110234549	5B	477979665
AX-111213373	A/A	A/T	T/T	Affx-110369298	5B	67967665
AX-111213640	A/A	A/G	A/A	Affx-109831903	5B	602858320
AX-111214314	C/C	T/T	T/T	Affx-111851302	5B	298609095
AX-111216522	C/C	T/C	C/C	Affx-110006537	5B	644584894
AX-111226351	C/C	T/C	C/C	Affx-111584304	5B	619037329
AX-111260909	G/G	A/G	G/G	Affx-109292639	5B	355960368
AX-111274399	G/G	A/G	G/G	Affx-109371904	5B	388846440
AX-111279951	C/C	A/C	C/C	Affx-109779615	5B	681329446
AX-111451165	G/G	A/G	A/A	Affx-111499516	5B	244757341
AX-111452189	T/T	T/G	G/G	Affx-111337524	5B	299611232
AX-111453141	C/C	C/C	T/T	Affx-110609703	5B	72900403
AX-111454697	C/C	C/C	C/C	Affx-109689042	5B	234774340
AX-111458311	A/A	G/G	G/G	Affx-109664308	5B	82943977
AX-111458448	T/T	C/C	T/T	Affx-88422116	5B	508681557
AX-111458533	G/G	A/G	A/A	Affx-88694918	5B	573817452
AX-111464585	T/T	T/C	C/C	Affx-109140047	5B	460676602

AX-111468686	T/T	T/C	C/C	Affx-109242867	5B	474526386
AX-111469464	C/C	T/C	T/T	Affx-109034172	5B	254974932
AX-111470008	A/A	A/A	A/A	Affx-109305377	5B	603760636
AX-111470663	T/T	C/C	C/C	Affx-110513855	5B	455533462
AX-111472432	A/A	A/G	G/G	Affx-110011651	5B	443039779
AX-111478371	C/C	A/C	C/C	Affx-109804453	5B	652563024
AX-111478844	G/G	A/G	G/G	Affx-108991610	5B	662193081
AX-111481610	A/A	G/G	A/A	Affx-111281860	5B	589990299
AX-111482700	C/C	C/C	C/C	Affx-108855970	5B	11480487
AX-111487952	A/A	A/G	A/A	Affx-108977140	5B	546266494
AX-111489106	C/C	A/C	A/A	Affx-111722116	5B	596684011
AX-111489840	G/G	A/G	G/G	Affx-110737699	5B	21733792
AX-111495704	A/A	A/G	A/A	Affx-111916947	5B	645995712
AX-111501562	G/G	A/G	G/G	Affx-110920179	5B	242356046
AX-111504233	C/C	C/C	A/C	Affx-109688225	5B	682168775
AX-111504551	A/G	A/G	G/G	Affx-110109963	5B	82153967
AX-111505214	C/C	T/T	C/C	Affx-109553681	5B	287797121
AX-111505621	A/A	A/G	G/G	Affx-88675703	5B	475999991
AX-111507926	G/G	C/G	C/C	Affx-109375195	5B	553530166
AX-111508809	C/C	G/G	C/C	Affx-110195990	5B	548737615
AX-111512401	A/A	A/G	A/A	Affx-111639642	5B	585393018
AX-111516865	C/C	C/C	C/C	Affx-109758416	5B	693823642
AX-111517432	G/G	A/G	G/G	Affx-109723184	5B	220997285
AX-111519225	G/G	A/G	G/G	Affx-111349891	5B	422925740
AX-111521753	C/C	C/C	T/T	Affx-109660581	5B	304693656
AX-111528735	G/G	A/G	A/A	Affx-111545693	5B	574063657
AX-111529524	T/T	G/G	G/G	Affx-111210421	5B	561974130
AX-111532854	T/T	T/C	C/C	Affx-110674077	5B	9073281
AX-111537848	G/G	A/G	G/G	Affx-111561917	5B	641558504
AX-111538681	A/A	A/G	G/G	Affx-109305495	5B	576313268
AX-111540807	G/G	A/G	G/G	Affx-109620716	5B	684377974
AX-111543104	A/G	G/G	A/G	Affx-110062961	5B	638030617
AX-111548112	A/G	A/G	A/G	Affx-88554611	5B	508796063
AX-111550930	C/C	T/T	C/C	Affx-111020422	5B	634084607
AX-111552615	G/G	A/G	G/G	Affx-109259179	5B	369065238
AX-111556505	G/G	C/C	G/G	Affx-109121237	5B	656256752
AX-111556737	A/A	A/G	A/A	Affx-88419503	5B	6367150
AX-111560416	A/A	A/C	C/C	Affx-109387969	5B	309954977
AX-111560952	G/G	C/G	G/G	Affx-109889526	5B	630138401
AX-111562557	G/G	A/G	G/G	Affx-111019310	5B	529533766
AX-111563063	T/T	T/T	C/C	Affx-111792741	5B	239959069
AX-111569451	C/C	T/C	T/T	Affx-88602636	5B	233491515
AX-111574616	A/A	A/C	A/A	Affx-111518472	5B	499375416
AX-111575871	C/C	T/C	C/C	Affx-111927901	5B	223627702
AX-111576625	C/C	C/C	C/C	Affx-111616784	5B	543955291
AX-111579934	G/G	A/G	A/A	Affx-111024468	5B	666674324
AX-111581062	G/G	T/G	G/G	Affx-108925636	5B	131363116
AX-111586069	C/C	A/C	C/C	Affx-110756518	5B	669218069
AX-111587221	G/G	C/G	C/C	Affx-110123570	5B	457624474
AX-111597396	A/A	A/A	A/A	Affx-111820588	5B	237711410
AX-111598230	A/A	A/T	A/A	Affx-111638028	5B	508062768
AX-111598900	G/G	C/G	G/G	Affx-111160285	5B	473743985
AX-111600319	A/A	A/G	G/G	Affx-110024385	5B	80960680
AX-111600619	G/G	A/G	A/A	Affx-110474240	5B	351304221

AX-111602446	C/C	C/C	C/C	Affx-109766310	5B	508779742
AX-111605612	C/C	T/C	C/C	Affx-110268673	5B	445786738
AX-111618006	T/T	G/G	T/T	Affx-110797820	5B	640396807
AX-111619112	T/T	T/T	T/T	Affx-109375320	5B	226052844
AX-111619412	G/G	G/G	C/C	Affx-111451108	5B	302344994
AX-111622763	A/A	A/G	A/A	Affx-108984962	5B	231352724
AX-111627941	T/T	T/C	T/T	Affx-110840544	5B	480726544
AX-111628117	C/C	T/C	C/C	Affx-108973531	5B	442028293
AX-111637148	C/C	T/C	T/T	Affx-109917443	5B	278244236
AX-111638173	A/T	A/T	T/T	Affx-109672441	5B	325068931
AX-111646654	C/C	T/C	C/C	Affx-111068159	5B	658092585
AX-111649791	G/G	G/G	A/G	Affx-110448063	5B	363822599
AX-111655832	C/C	T/C	T/T	Affx-110880838	5B	322335291
AX-111659492	C/C	T/C	C/C	Affx-108910278	5B	98558779
AX-111661819	T/T	T/C	T/T	Affx-110401395	5B	618265886
AX-111661959	A/A	G/G	A/A	Affx-110747565	5B	631588782
AX-111661980	G/G	T/G	G/G	Affx-111686802	5B	30846525
AX-111667993	C/C	T/C	C/C	Affx-88737307	5B	240977879
AX-111668512	T/T	T/T	T/T	Affx-110698331	5B	348575672
AX-111672485	A/A	A/G	A/A	Affx-111940117	5B	422501097
AX-111680963	G/G	A/G	G/G	Affx-110857432	5B	222694338
AX-111686529	T/T	T/C	C/C	Affx-110086393	5B	570645838
AX-111686850	T/T	T/T	T/T	Affx-88726905	5B	130912143
AX-111688868	A/A	A/C	C/C	Affx-111229958	5B	48998916
AX-111689113	C/C	T/C	C/C	Affx-110844581	5B	67208695
AX-111689338	T/T	T/C	C/C	Affx-109681876	5B	84424851
AX-111697598	T/T	T/C	T/T	Affx-110453871	5B	405520813
AX-111700503	A/G	A/G	G/G	Affx-109984206	5B	457104001
AX-111701657	A/A	A/G	A/A	Affx-110318000	5B	580647873
AX-111702062	A/A	A/G	A/A	Affx-109704422	5B	489977810
AX-111704008	G/G	A/G	G/G	Affx-110455951	5B	662460076
AX-111709936	C/C	C/G	C/C	Affx-110989184	5B	515183465
AX-111710417	T/T	A/T	T/T	Affx-111153500	5B	393515648
AX-111715324	G/G	A/G	A/A	Affx-109508096	5B	597852055
AX-111726481	C/C	A/A	C/C	Affx-110841326	5B	476810524
AX-111728752	G/G	A/G	G/G	Affx-110267227	5B	508679088
AX-111731367	G/G	G/G	G/G	Affx-109908610	5B	445193423
AX-111760394	T/T	T/C	C/C	Affx-110138703	5B	303850189
AX-111762264	G/G	A/G	G/G	Affx-110366414	5B	33888435
AX-111762700	T/T	T/C	T/T	Affx-111363226	5B	535387665
AX-111764350	T/T	T/G	T/T	Affx-111603876	5B	414869465
AX-111794344	G/G	C/G	C/C	Affx-88606242	5B	45876471
AX-111806801	C/C	T/T	T/C	Affx-109311961	5B	62544341
AX-112286223	C/C	C/C	C/C	Affx-88406617	5B	669896712
AX-112286466	G/G	G/G	G/G	Affx-92982087	5B	617137928
AX-112286535	A/G	A/G	G/G	Affx-112315368	5B	708222957
AX-112287303	T/T	T/T	T/T	Affx-88375971	5B	631029058
AX-112291025	G/G	A/G	G/G	Affx-112315115	5B	207578864
AX-182073925	T/C	T/T	C/C	Affx-88438627	5B	655149053
AX-182075201	A/G	G/G	G/G	Affx-109530784	5B	573814114
AX-182120036	T/C	T/C	T/C	Affx-112313562	5B	701155513
AX-182124541	G/G	G/G	A/A	Affx-157768906	5B	65076743
AX-182133443	G/G	A/A	G/G	Affx-472281030	5B	430465990
AX-182182112	T/G	T/G	T/G	Affx-88615539	5B	635358654

AX-86169602	T/T	T/C	C/C	Affx-88677775	5B	60898128
AX-86170549	C/C	C/C	C/C	Affx-112315087	5B	669896568
AX-86172467	G/G	A/G	G/G	Affx-92967781	5B	656905209
AX-86173610	T/T	T/C	T/T	Affx-92743352	5B	484735572
AX-86174909	A/G	A/G	G/G	Affx-88534443	5B	70096902
AX-86179657	C/C	A/A	C/C	Affx-92763467	5B	670081444
AX-86183841	G/G	G/G	G/G	Affx-88516586	5B	577305479
AX-86184149	C/C	A/C	C/C	Affx-292486734	5B	709159885
AX-86184167	T/T	T/T	T/T	Affx-88695197	5B	65243608
AX-86184886	C/C	T/T	C/C	Affx-88418355	5B	411930852
AX-86185288	A/G	A/G	G/G	Affx-88467068	5B	455738909
AX-89338050	G/G	A/A	G/G	Affx-88369568	5B	439640248
AX-89347054	C/G	C/G	C/G	Affx-109207701	5B	610293195
AX-89350144	G/G	A/G	G/G	Affx-88381954	5B	578535924
AX-89369152	T/G	G/G	G/G	Affx-92263603	5B	461829096
AX-89376056	A/A	A/C	C/C	Affx-88408331	5B	572362593
AX-89390293	T/T	T/C	T/T	Affx-88422764	5B	26626241
AX-89545038	C/C	C/C	C/C	Affx-88578042	5B	548339223
AX-89559329	C/C	T/C	T/T	Affx-88592373	5B	577087101
AX-89559762	A/C	A/C	C/C	Affx-88592807	5B	713016195
AX-89619828	T/G	T/G	G/G	Affx-111378984	5B	33550874
AX-89634491	C/C	T/C	T/T	Affx-88667277	5B	15543544
AX-89643192	T/T	T/C	T/T	Affx-88675988	5B	427938995
AX-94395599	C/C	A/C	A/C	Affx-92375842	5B	461695824
AX-94402018	T/T	T/G	T/T	Affx-88660266	5B	671301137
AX-94416401	T/T	T/G	T/T	Affx-92377610	5B	541286983
AX-94424823	G/G	G/G	G/G	Affx-92247014	5B	580701912
AX-94427981	T/C	C/C	T/C	Affx-92093886	5B	79803840
AX-94466402	C/C	C/C	C/C	Affx-92407150	5B	640545010
AX-94468466	T/T	T/T	T/T	Affx-92711341	5B	558346522
AX-94473314	A/A	A/C	A/A	Affx-88461216	5B	357552957
AX-94496563	C/C	A/C	A/A	Affx-88371049	5B	279097462
AX-94501518	T/T	T/C	T/T	Affx-88725691	5B	678659619
AX-94524014	G/G	C/G	G/G	Affx-92760462	5B	440179428
AX-94551734	A/C	A/C	C/C	Affx-92275719	5B	595661843
AX-94557246	T/T	T/T	T/T	Affx-92812168	5B	701275379
AX-94574188	C/C	C/C	C/C	Affx-92352705	5B	671713474
AX-94598757	C/C	T/C	T/T	Affx-92191159	5B	316562253
AX-94705282	A/G	G/G	A/G	Affx-92635011	5B	420470949
AX-94790856	C/C	C/C	C/C	Affx-92463798	5B	646667048
AX-94791452	A/G	A/A	A/G	Affx-88552230	5B	12577524
AX-94924089	T/C	C/C	T/C	Affx-92246538	5B	601410495
AX-94961602	T/T	T/T	T/T	Affx-92182376	5B	656232891
AX-95086176	A/G	G/G	A/G	Affx-88386239	5B	656232736
AX-95158231	A/A	A/C	A/A	Affx-92590303	5B	327080423
AX-95224602	C/C	C/C	C/C	Affx-92172333	5B	323883060
AX-95244330	C/C	C/C	C/C	Affx-92507827	5B	153896290
AX-95629427	G/G	A/G	A/A	Affx-88619867	5B	476455882
AX-95630565	A/C	A/C	A/C	Affx-92488396	5B	638508778
AX-95631404	G/G	G/G	G/G	Affx-88446663	5B	587126799
AX-95631754	T/T	C/C	T/T	Affx-88430670	5B	432155448
AX-95632003	T/T	T/C	T/T	Affx-88587556	5B	587127305
AX-95632225	T/T	C/C	T/T	Affx-88785501	5B	617141997
AX-95632227	A/A	A/C	A/A	Affx-88570444	5B	562049149

AX-95632934	T/T	T/C	T/T	Affx-112315322	5B	631026825
AX-95652169	G/G	G/G	G/G	Affx-92986731	5B	702888997
AX-95658317	G/G	G/G	G/G	Affx-88415358	5B	13190710
AX-108731705	C/C	C/C	C/C	Affx-108893922	5D	225270546
AX-108736982	A/A	A/C	A/A	Affx-110220566	5D	477317408
AX-108744042	G/G	A/G	G/G	Affx-109469258	5D	229271887
AX-108746184	C/C	T/C	C/C	Affx-109194372	5D	485120007
AX-108749884	A/A	A/G	A/A	Affx-109530741	5D	122633245
AX-108752370	C/C	T/C	C/C	Affx-110467539	5D	450856089
AX-108754285	G/G	A/G	G/G	Affx-109000647	5D	452850949
AX-108760983	T/T	T/C	T/T	Affx-109352604	5D	446482624
AX-108761993	G/G	A/G	G/G	Affx-109915042	5D	222521882
AX-108762744	G/G	T/G	G/G	Affx-111872405	5D	120395509
AX-108765683	T/T	T/C	T/T	Affx-110691987	5D	166073814
AX-108767468	C/C	C/G	C/C	Affx-111261358	5D	337573644
AX-108771023	C/C	T/C	C/C	Affx-110212610	5D	246430312
AX-108774821	T/T	T/C	C/C	Affx-111594548	5D	316136440
AX-108775550	A/A	A/G	A/A	Affx-110285969	5D	557652144
AX-108776047	C/C	T/C	C/C	Affx-110887415	5D	215918674
AX-108782785	T/T	T/C	T/T	Affx-111195391	5D	298445559
AX-108785792	T/T	T/C	T/T	Affx-109037845	5D	143832746
AX-108800523	A/A	A/C	A/A	Affx-110391647	5D	525591988
AX-108803615	C/C	C/G	G/G	Affx-111036283	5D	486092519
AX-108815793	C/C	T/C	C/C	Affx-109774819	5D	341247615
AX-108816919	G/G	A/G	G/G	Affx-110783473	5D	219651429
AX-108822269	A/A	G/G	A/A	Affx-88375497	5D	499933111
AX-108827139	G/G	A/G	G/G	Affx-109740563	5D	177136362
AX-108827297	T/T	T/C	T/T	Affx-109303136	5D	370064912
AX-108840042	T/T	T/C	T/T	Affx-88792219	5D	42961528
AX-108843804	C/C	T/C	C/C	Affx-111265525	5D	453228696
AX-108848074	G/G	A/G	G/G	Affx-109465918	5D	338646619
AX-108854022	C/C	T/C	C/C	Affx-109205547	5D	110061615
AX-108861262	G/G	C/G	G/G	Affx-111285728	5D	468164833
AX-108871909	C/C	T/C	C/C	Affx-109617169	5D	256308438
AX-108879112	C/C	A/C	A/A	Affx-111126722	5D	552740737
AX-108886493	T/T	T/C	T/T	Affx-109841538	5D	24579492
AX-108889119	C/C	T/C	C/C	Affx-109940486	5D	467183234
AX-108893231	A/G	G/G	A/G	Affx-111055923	5D	467261908
AX-108907798	C/C	C/G	C/C	Affx-110141653	5D	419809473
AX-108910713	T/T	T/C	C/C	Affx-109928772	5D	486935936
AX-108913102	T/C	T/C	C/C	Affx-111186416	5D	65860986
AX-108919131	C/C	T/C	C/C	Affx-111086037	5D	330036559
AX-108930462	G/G	A/A	G/G	Affx-88743228	5D	40914557
AX-108947746	G/G	A/G	A/A	Affx-88502815	5D	362596703
AX-108948832	C/G	C/G	C/G	Affx-109630358	5D	429812333
AX-108955959	A/A	A/G	A/A	Affx-88709686	5D	359471713
AX-108960709	T/T	T/C	T/T	Affx-111708166	5D	162761169
AX-108980479	G/G	A/G	A/A	Affx-109319888	5D	305713314
AX-108986002	G/G	A/G	G/G	Affx-111527395	5D	213131952
AX-108986735	G/G	T/G	G/G	Affx-109073899	5D	51926474
AX-109005371	G/G	A/G	G/G	Affx-109466571	5D	466233355
AX-109033848	G/G	A/G	G/G	Affx-111930243	5D	486813483
AX-109037061	A/A	A/G	A/A	Affx-110400435	5D	326801822
AX-109040568	C/C	T/C	C/C	Affx-109063571	5D	357209295

AX-109042166	T/T	T/C	T/T	Affx-111465105	5D	456308062
AX-109047408	T/T	T/T	T/T	Affx-111765821	5D	467056864
AX-109078393	G/G	A/G	A/A	Affx-88766108	5D	405967649
AX-109082110	C/C	C/C	C/C	Affx-110771997	5D	154789958
AX-109110256	A/A	A/A	A/A	Affx-88553670	5D	49307298
AX-109124553	G/G	C/G	G/G	Affx-110564726	5D	40348441
AX-109126728	A/A	A/G	A/A	Affx-111600381	5D	493459522
AX-109129846	T/T	T/C	T/T	Affx-109696749	5D	294272278
AX-109138412	A/A	A/A	A/A	Affx-109690737	5D	245990805
AX-109147506	T/T	T/T	T/T	Affx-111446022	5D	76706021
AX-109172519	G/G	A/G	G/G	Affx-88526546	5D	119932161
AX-109174882	A/A	A/A	A/A	Affx-88607453	5D	520844905
AX-109184534	G/G	C/G	G/G	Affx-108917923	5D	494925798
AX-109195695	C/C	T/C	C/C	Affx-88802156	5D	447900718
AX-109199754	G/G	A/G	A/A	Affx-108988059	5D	318197754
AX-109207441	C/C	T/C	C/C	Affx-110543726	5D	534992827
AX-109243867	G/G	A/G	G/G	Affx-110073774	5D	226506245
AX-109255589	G/G	A/G	G/G	Affx-111721964	5D	366060708
AX-109270001	C/C	A/A	A/A	Affx-111566911	5D	513904055
AX-109272147	G/G	A/G	G/G	Affx-111241246	5D	433477782
AX-109274943	A/A	A/T	A/A	Affx-111796577	5D	30118258
AX-109280141	A/A	A/G	A/A	Affx-109603014	5D	507853822
AX-109292346	C/C	T/C	C/C	Affx-88574029	5D	501774813
AX-109292384	T/T	T/C	T/T	Affx-110502005	5D	274140861
AX-109311235	A/A	A/G	A/A	Affx-111512600	5D	497861569
AX-109314976	C/C	A/C	C/C	Affx-109722969	5D	34045139
AX-109323735	C/C	T/C	C/C	Affx-108924503	5D	191908598
AX-109324087	C/C	T/C	C/C	Affx-109724401	5D	348270969
AX-109341161	A/A	A/G	A/A	Affx-88785513	5D	344404704
AX-109341618	C/G	C/G	C/G	Affx-111509140	5D	416627832
AX-109342529	G/G	G/G	G/G	Affx-109940739	5D	156735104
AX-109352242	G/G	A/G	G/G	Affx-111385191	5D	132122496
AX-109353871	G/G	A/G	G/G	Affx-111013203	5D	311308086
AX-109355673	G/G	T/G	G/G	Affx-109935587	5D	35532314
AX-109360538	G/G	C/G	G/G	Affx-88623464	5D	439209094
AX-109369334	C/G	C/G	C/C	Affx-111694665	5D	458928629
AX-109373924	G/G	A/G	G/G	Affx-111851172	5D	131490051
AX-109377377	A/A	A/G	A/A	Affx-111270590	5D	556430004
AX-109384482	G/G	G/G	G/G	Affx-88377150	5D	47517811
AX-109394742	C/C	T/C	C/C	Affx-109331491	5D	12584488
AX-109407131	A/A	A/G	G/G	Affx-111527033	5D	512550680
AX-109411245	G/G	A/G	G/G	Affx-109817644	5D	58260415
AX-109418073	C/C	T/C	C/C	Affx-110751831	5D	254411856
AX-109418822	T/T	T/C	C/C	Affx-88793974	5D	549581524
AX-109418837	G/G	G/G	G/G	Affx-110654571	5D	27437991
AX-109425776	C/C	T/C	C/C	Affx-111523961	5D	150565578
AX-109435975	T/T	C/C	T/T	Affx-111820028	5D	561575252
AX-109437994	G/G	A/G	G/G	Affx-109788167	5D	322645648
AX-109460966	T/T	T/G	T/T	Affx-111111316	5D	8612500
AX-109464956	A/A	A/G	G/G	Affx-110835502	5D	495427294
AX-109477622	T/T	T/C	T/T	Affx-108901143	5D	235839061
AX-109500485	C/C	T/C	C/C	Affx-111002891	5D	167263932
AX-109510279	G/G	A/A	G/G	Affx-88634620	5D	45894336
AX-109527199	T/T	T/C	T/T	Affx-109129589	5D	426296784

AX-109541444	G/G	A/G	G/G	Affx-111718488	5D	221482992
AX-109558694	C/C	C/C	C/C	Affx-109043752	5D	562067185
AX-109582768	C/C	C/C	C/C	Affx-109026318	5D	444464972
AX-109586392	C/C	T/C	C/C	Affx-110079682	5D	285111348
AX-109629542	G/G	A/G	G/G	Affx-111283062	5D	266384393
AX-109636050	G/G	A/G	G/G	Affx-111186212	5D	3615837
AX-109684926	T/T	T/G	T/T	Affx-110379383	5D	100939685
AX-109714016	T/T	T/C	T/T	Affx-110597488	5D	166788561
AX-109725811	A/A	A/G	A/A	Affx-110415550	5D	440347835
AX-109726135	A/A	A/G	A/A	Affx-109898165	5D	381728268
AX-109748261	A/A	A/C	A/A	Affx-110634682	5D	217918643
AX-109749882	T/T	T/G	T/T	Affx-111268106	5D	457269886
AX-109750213	A/G	A/G	A/G	Affx-109914514	5D	466694319
AX-109772237	T/T	A/T	T/T	Affx-110905379	5D	46848662
AX-109809706	A/A	A/G	A/A	Affx-109227528	5D	476819446
AX-109814956	A/A	A/G	A/A	Affx-110796799	5D	96014369
AX-109815389	G/G	G/G	G/G	Affx-110304081	5D	66764672
AX-109815603	C/C	T/C	C/C	Affx-111071811	5D	449685905
AX-109816934	A/A	A/G	A/A	Affx-109688335	5D	526907207
AX-109822039	A/A	A/C	A/A	Affx-110523810	5D	154563964
AX-109825897	C/C	T/C	C/C	Affx-108867362	5D	221145774
AX-109839904	G/G	A/G	A/A	Affx-88610971	5D	553781207
AX-109850798	T/T	T/C	T/T	Affx-110903641	5D	351801215
AX-109855976	T/T	T/C	T/T	Affx-88422825	5D	449292471
AX-109856411	T/T	T/C	T/T	Affx-111561446	5D	232517194
AX-109857030	G/G	A/G	G/G	Affx-111957362	5D	327984356
AX-109859166	A/A	G/G	A/A	Affx-109302015	5D	423242733
AX-109861425	T/T	T/C	C/C	Affx-111339700	5D	488693044
AX-109889619	C/C	T/C	C/C	Affx-88705234	5D	504809929
AX-109891323	T/C	T/C	T/T	Affx-111249401	5D	327266591
AX-109897023	T/T	T/G	T/T	Affx-111666051	5D	408975320
AX-109900028	G/G	A/G	G/G	Affx-88659662	5D	304169514
AX-109904168	G/G	C/G	G/G	Affx-110452843	5D	161477671
AX-109907784	G/G	A/G	A/A	Affx-108954032	5D	551746348
AX-109909998	T/T	T/C	T/T	Affx-109068958	5D	421611849
AX-109915792	C/C	T/C	C/C	Affx-111838852	5D	251308792
AX-109923560	C/C	T/C	C/C	Affx-108913569	5D	479564938
AX-109924593	G/G	A/G	G/G	Affx-111464000	5D	169241521
AX-109927141	G/G	C/G	C/G	Affx-110585507	5D	542354416
AX-109929775	C/C	T/T	T/T	Affx-88685411	5D	369033454
AX-109940890	T/T	T/C	C/C	Affx-109887992	5D	545525704
AX-109943729	G/G	T/G	G/G	Affx-88489028	5D	129479551
AX-109944385	G/G	G/G	A/A	Affx-110885708	5D	541558903
AX-109947280	C/C	T/C	C/C	Affx-109267769	5D	409567524
AX-109968486	T/T	T/G	T/T	Affx-109770546	5D	414844279
AX-109970310	G/G	A/G	G/G	Affx-109118989	5D	397950472
AX-109986939	C/C	C/G	C/C	Affx-110799910	5D	2679503
AX-110023827	G/G	A/G	G/G	Affx-109684626	5D	467056904
AX-110024940	C/C	C/G	G/G	Affx-109017884	5D	554853129
AX-110035093	T/T	T/C	T/T	Affx-110431302	5D	464129291
AX-110035446	G/G	A/G	G/G	Affx-109907376	5D	286364529
AX-110036898	G/G	A/G	G/G	Affx-110870858	5D	341844881
AX-110038121	G/G	T/G	G/G	Affx-110339074	5D	276671772
AX-110039178	A/A	A/G	A/A	Affx-88705945	5D	303758601

AX-110041006	T/T	T/C	T/T	Affx-110325302	5D	175921241
AX-110041896	G/G	G/G	G/G	Affx-111261419	5D	323983507
AX-110043730	G/G	C/G	G/G	Affx-109447342	5D	537640605
AX-110044591	A/G	G/G	A/G	Affx-110780770	5D	367993384
AX-110050695	C/C	T/T	C/C	Affx-109836274	5D	63773557
AX-110058454	C/C	T/T	C/C	Affx-110789518	5D	506915637
AX-110063466	T/T	T/G	G/G	Affx-111602859	5D	551129528
AX-110079370	T/T	T/C	T/T	Affx-88395192	5D	309003912
AX-110088350	T/C	C/C	T/C	Affx-110273811	5D	101421590
AX-110089409	G/G	A/G	G/G	Affx-111844209	5D	548022662
AX-110102144	T/T	T/C	T/T	Affx-111245188	5D	181624609
AX-110129540	C/C	T/T	C/C	Affx-110638968	5D	36635637
AX-110132720	G/G	A/G	G/G	Affx-111302661	5D	227319415
AX-110172785	A/G	A/A	G/G	Affx-88362047	5D	197652565
AX-110186027	A/A	A/G	A/A	Affx-110309926	5D	279115969
AX-110186607	T/T	T/C	C/C	Affx-111053186	5D	401359202
AX-110213253	G/G	A/G	G/G	Affx-88803687	5D	157202548
AX-110238102	C/C	C/C	C/C	Affx-109597838	5D	16435705
AX-110253278	G/G	A/G	G/G	Affx-111902585	5D	469486357
AX-110271557	A/A	A/G	A/A	Affx-109467454	5D	246966623
AX-110323751	A/A	A/G	G/G	Affx-109616327	5D	485750527
AX-110328751	C/C	T/C	C/C	Affx-109196707	5D	232557806
AX-110369521	A/A	A/A	A/A	Affx-110756779	5D	347276902
AX-110379152	C/C	T/C	C/C	Affx-109703491	5D	503662714
AX-110385590	C/C	T/C	C/C	Affx-110604714	5D	447632536
AX-110387496	T/T	T/T	T/T	Affx-111551702	5D	434921458
AX-110399580	C/C	A/C	C/C	Affx-109698009	5D	290051251
AX-110405892	T/T	T/C	T/T	Affx-109535466	5D	410779455
AX-110409079	C/C	A/C	C/C	Affx-88592452	5D	562834171
AX-110410822	G/G	T/G	G/G	Affx-110036424	5D	507417391
AX-110411368	G/G	C/G	G/G	Affx-109983356	5D	282174276
AX-110415653	G/G	G/G	G/G	Affx-110644053	5D	276314212
AX-110415968	C/C	T/C	C/C	Affx-109308918	5D	303426242
AX-110416339	C/C	A/C	C/C	Affx-109592562	5D	93620719
AX-110421779	T/T	T/C	T/T	Affx-108940463	5D	434549183
AX-110445781	T/G	T/G	G/G	Affx-88614888	5D	541798353
AX-110460040	C/C	T/C	C/C	Affx-110612828	5D	247618289
AX-110461319	A/A	A/G	A/A	Affx-88577500	5D	86730527
AX-110476332	T/T	T/C	T/T	Affx-111006424	5D	6393316
AX-110476682	C/C	A/C	C/C	Affx-111040842	5D	506330821
AX-110478685	C/C	T/C	T/T	Affx-109876751	5D	530828601
AX-110491854	A/G	A/A	A/A	Affx-110062389	5D	275915579
AX-110499907	C/C	T/C	C/C	Affx-88660865	5D	342424964
AX-110503408	A/A	A/A	A/A	Affx-108878763	5D	379836128
AX-110503912	C/C	T/C	C/C	Affx-110709377	5D	175008367
AX-110506896	G/G	A/G	G/G	Affx-111137577	5D	357941654
AX-110510530	T/T	T/T	C/C	Affx-109274218	5D	544894510
AX-110521341	C/C	T/C	C/C	Affx-109730146	5D	30854436
AX-110528585	G/G	C/G	G/G	Affx-108949700	5D	472433603
AX-110534485	G/G	C/G	G/G	Affx-110877563	5D	317386724
AX-110541781	T/T	T/G	T/T	Affx-109662585	5D	378910396
AX-110542913	A/T	A/T	A/T	Affx-110305905	5D	157687538
AX-110549526	A/A	A/G	A/A	Affx-109345427	5D	481808025
AX-110558491	G/G	A/G	G/G	Affx-111443253	5D	385893910

AX-110563756	C/C	C/G	C/C	Affx-111602420	5D	366044543
AX-110570148	G/G	A/G	G/G	Affx-88395921	5D	407762345
AX-110570350	A/A	A/G	G/G	Affx-88615052	5D	404104771
AX-110572300	G/G	A/G	G/G	Affx-111055595	5D	262680048
AX-110575891	A/A	A/A	A/A	Affx-109386976	5D	237629967
AX-110578278	G/G	A/G	G/G	Affx-110903558	5D	448603707
AX-110581813	A/A	A/G	A/A	Affx-109320839	5D	302000445
AX-110586161	A/A	G/G	A/A	Affx-111241744	5D	465851494
AX-110594709	G/G	A/G	G/G	Affx-88733074	5D	124543281
AX-110616233	C/C	T/T	C/C	Affx-110738978	5D	434403364
AX-110617116	T/G	G/G	T/G	Affx-109265363	5D	200638598
AX-110618111	C/C	C/G	C/C	Affx-109626307	5D	115977500
AX-110619828	A/G	G/G	G/G	Affx-108961426	5D	371472298
AX-110641742	C/C	C/G	C/C	Affx-110429356	5D	416424385
AX-110643173	C/C	T/C	T/T	Affx-111913977	5D	555046055
AX-110670807	C/C	T/C	C/C	Affx-111334589	5D	134249892
AX-110676362	C/C	T/C	C/C	Affx-109867555	5D	353010493
AX-110703534	C/C	T/C	C/C	Affx-111999139	5D	288313381
AX-110717039	G/G	A/A	G/G	Affx-111380061	5D	190240423
AX-110717870	C/C	T/C	C/C	Affx-110499354	5D	465106394
AX-110717927	G/G	A/G	G/G	Affx-111582989	5D	192553846
AX-110753841	G/G	T/G	T/T	Affx-110475045	5D	333962239
AX-110758473	A/A	A/G	A/A	Affx-111260632	5D	292945585
AX-110767974	C/C	T/T	C/C	Affx-110594922	5D	282911507
AX-110793386	A/A	A/A	A/A	Affx-110299775	5D	306975268
AX-110808071	G/G	A/G	G/G	Affx-109149782	5D	387143750
AX-110825921	C/C	T/C	T/T	Affx-111665202	5D	244363694
AX-110834664	A/G	A/G	A/G	Affx-111152060	5D	177018620
AX-110840614	C/C	T/C	C/C	Affx-109374825	5D	216295270
AX-110853246	C/C	T/C	C/C	Affx-110325490	5D	467054086
AX-110878801	T/T	T/C	T/T	Affx-111653554	5D	4427092
AX-110899362	G/G	A/G	G/G	Affx-110560650	5D	218636547
AX-110912566	C/C	C/G	C/C	Affx-111186603	5D	104115457
AX-110930051	G/G	T/G	G/G	Affx-109594344	5D	480341552
AX-110931647	A/A	A/C	A/A	Affx-88573723	5D	46089612
AX-110935119	A/A	A/C	C/C	Affx-88681275	5D	543835634
AX-110950194	T/T	T/C	T/T	Affx-111321865	5D	467575526
AX-110954712	G/G	A/A	G/G	Affx-111653958	5D	200905940
AX-110955411	C/C	C/C	C/C	Affx-109812183	5D	224948340
AX-110966545	A/A	A/G	A/A	Affx-110916393	5D	155411381
AX-110967183	A/A	A/G	G/G	Affx-109538455	5D	546923023
AX-110978247	G/G	A/G	G/G	Affx-108888503	5D	34790697
AX-110985437	T/T	T/C	C/C	Affx-110558944	5D	404832130
AX-110996226	T/C	T/C	T/C	Affx-88784527	5D	500850181
AX-111011274	G/G	C/G	G/G	Affx-109852119	5D	280777473
AX-111022479	T/T	T/C	C/C	Affx-110361949	5D	489388881
AX-111028270	C/C	T/C	C/C	Affx-110183961	5D	528750424
AX-111030610	C/C	C/C	C/C	Affx-88744526	5D	106818950
AX-111035187	G/G	G/G	G/G	Affx-110000814	5D	429360696
AX-111053238	T/T	T/C	T/T	Affx-110187995	5D	513522362
AX-111055722	A/A	A/G	A/A	Affx-111877252	5D	446113191
AX-111062349	T/T	T/G	T/T	Affx-110932011	5D	556724545
AX-111069708	G/G	A/G	G/G	Affx-110859992	5D	139465833
AX-111072231	A/A	A/G	A/A	Affx-110125340	5D	503149709

AX-111085797	C/C	T/C	T/T	Affx-111780544	5D	406597866
AX-111100875	G/G	T/G	G/G	Affx-88455507	5D	560509338
AX-111106656	T/T	T/C	T/T	Affx-111563313	5D	101894979
AX-111109050	G/G	A/G	G/G	Affx-111900652	5D	472196928
AX-111117089	G/G	C/G	C/C	Affx-110363219	5D	290675234
AX-111118954	G/G	A/G	G/G	Affx-111127830	5D	419371806
AX-111119791	G/G	T/G	G/G	Affx-110610562	5D	562641263
AX-111132295	A/A	A/G	A/A	Affx-109322860	5D	469751094
AX-111137392	C/C	C/G	G/G	Affx-111065279	5D	266328580
AX-111157940	A/A	A/G	A/A	Affx-111723999	5D	478686148
AX-111165832	G/G	A/G	G/G	Affx-110947032	5D	367422540
AX-111175690	C/C	T/T	C/C	Affx-111881639	5D	464415388
AX-111232240	T/T	A/T	A/A	Affx-111841404	5D	550144075
AX-111261471	C/C	T/C	C/C	Affx-110296407	5D	139458502
AX-111279901	G/G	T/G	G/G	Affx-88659498	5D	501159808
AX-111288181	G/G	A/G	G/G	Affx-110161996	5D	321796245
AX-111289304	C/C	T/C	C/C	Affx-109390287	5D	135470314
AX-111290761	G/G	A/G	G/G	Affx-109646622	5D	230497326
AX-111299554	A/A	A/G	A/A	Affx-108993070	5D	178456848
AX-111300947	T/T	T/C	T/T	Affx-109371297	5D	311303363
AX-111313972	C/C	T/C	C/C	Affx-109989896	5D	214621998
AX-111316333	G/G	C/G	G/G	Affx-111580943	5D	178155340
AX-111317347	C/C	C/G	C/C	Affx-88707747	5D	540175608
AX-111328081	C/C	T/C	C/C	Affx-111496911	5D	388627630
AX-111337114	A/A	A/A	A/A	Affx-108997261	5D	126664967
AX-111353139	C/C	T/C	T/T	Affx-110126227	5D	349358367
AX-111355048	A/A	A/G	A/A	Affx-111041700	5D	505374811
AX-111364298	G/G	A/G	G/G	Affx-109424255	5D	533870402
AX-111370669	G/G	C/G	C/C	Affx-109140614	5D	546304611
AX-111388087	G/G	G/G	A/A	Affx-109079706	5D	525826255
AX-111390298	G/G	A/A	G/G	Affx-111037387	5D	196421898
AX-111437361	G/G	T/G	G/G	Affx-109825644	5D	491942565
AX-111448085	G/G	A/G	G/G	Affx-110758567	5D	467261465
AX-111459824	G/G	T/G	G/G	Affx-109952745	5D	545954778
AX-111464164	T/T	T/C	T/T	Affx-111724853	5D	5564218
AX-111469538	A/A	A/G	A/A	Affx-109049205	5D	502082794
AX-111476253	T/T	T/G	T/T	Affx-88640147	5D	504263518
AX-111481250	A/A	A/G	A/A	Affx-110480241	5D	530406361
AX-111481432	C/C	T/C	C/C	Affx-110571931	5D	236656109
AX-111481618	C/C	T/C	C/C	Affx-111260615	5D	468835604
AX-111488807	C/C	T/C	C/C	Affx-109358504	5D	159287810
AX-111489338	A/A	A/G	A/A	Affx-109364313	5D	414054015
AX-111489682	T/T	T/C	T/T	Affx-109973359	5D	21321942
AX-111497172	A/A	A/G	A/A	Affx-109908817	5D	454427605
AX-111499218	T/T	T/C	T/T	Affx-109772646	5D	558437887
AX-111512411	C/C	C/G	C/C	Affx-111261319	5D	432132545
AX-111517418	G/G	A/G	G/G	Affx-110305703	5D	365091678
AX-111520160	T/T	A/T	T/T	Affx-110551200	5D	237622586
AX-111522106	G/G	A/G	G/G	Affx-88440584	5D	228727295
AX-111537618	C/C	T/C	C/C	Affx-111403305	5D	285248649
AX-111539925	T/T	T/C	T/T	Affx-109341641	5D	91340939
AX-111540030	G/G	A/G	G/G	Affx-111610001	5D	335082923
AX-111543112	A/A	A/A	A/A	Affx-109240813	5D	38070258
AX-111544605	A/A	A/G	A/A	Affx-111864526	5D	499648723

AX-111546368	C/C	T/C	C/C	Affx-88435772	5D	119674210
AX-111548559	C/C	C/G	C/C	Affx-109103234	5D	10609796
AX-111549766	T/T	T/G	T/T	Affx-109289802	5D	400363592
AX-111555981	C/C	T/C	C/C	Affx-88609676	5D	458840577
AX-111557248	C/C	C/C	C/C	Affx-111772900	5D	539585015
AX-111558888	C/C	A/C	C/C	Affx-111805536	5D	340973683
AX-111577847	C/C	C/C	C/C	Affx-88350555	5D	240500805
AX-111580436	G/G	A/G	G/G	Affx-111418141	5D	93046756
AX-111584361	T/G	G/G	G/G	Affx-110054309	5D	493680450
AX-111593182	A/A	A/G	A/A	Affx-88472735	5D	467345178
AX-111593479	A/A	A/G	A/A	Affx-109007147	5D	272356697
AX-111597176	G/G	A/G	G/G	Affx-109704816	5D	70896874
AX-111597800	G/G	A/G	G/G	Affx-110784975	5D	166343001
AX-111602283	G/G	A/A	G/G	Affx-111602884	5D	438577821
AX-111605876	G/G	T/G	G/G	Affx-88760044	5D	361886565
AX-111611604	G/G	C/G	G/G	Affx-111137412	5D	558950981
AX-111616351	A/A	A/C	A/A	Affx-109258080	5D	440808320
AX-111616614	G/G	A/G	G/G	Affx-109612774	5D	393799703
AX-111628196	C/C	C/G	C/C	Affx-88373470	5D	559484099
AX-111631716	G/G	T/G	G/G	Affx-108944231	5D	137017224
AX-111632340	C/C	C/G	C/C	Affx-109726646	5D	214353499
AX-111641446	C/C	T/C	C/C	Affx-111019490	5D	234511209
AX-111641548	C/C	T/C	C/C	Affx-108896743	5D	432985451
AX-111643347	G/G	A/G	G/G	Affx-88411070	5D	240497119
AX-111652649	A/A	A/G	A/A	Affx-110680062	5D	478571830
AX-111670747	T/T	T/C	C/C	Affx-110798076	5D	509953266
AX-111676465	T/T	T/C	C/C	Affx-110918935	5D	364425715
AX-111677889	G/G	A/G	A/A	Affx-109650820	5D	313367535
AX-111681285	A/A	A/G	G/G	Affx-111332404	5D	314438299
AX-111681832	A/A	A/C	A/A	Affx-109306869	5D	408745460
AX-111686469	G/G	A/G	A/A	Affx-109967170	5D	307757618
AX-111689691	C/C	T/T	C/C	Affx-110600133	5D	129067973
AX-111691501	C/C	T/C	T/T	Affx-111699106	5D	510906580
AX-111717376	A/A	A/C	A/A	Affx-109225159	5D	345084248
AX-111723433	A/A	A/T	A/A	Affx-110499445	5D	65767361
AX-111734044	T/T	T/T	T/T	Affx-111797230	5D	3609930
AX-111746541	C/C	T/T	C/C	Affx-109315573	5D	387095607
AX-111752399	C/C	T/C	C/C	Affx-111792689	5D	277565500
AX-111756142	T/T	T/T	G/G	Affx-111336951	5D	398101846
AX-111768480	C/C	T/C	C/C	Affx-111356163	5D	26253090
AX-111770967	G/G	G/G	G/G	Affx-110493154	5D	466860796
AX-111780450	A/A	A/A	A/A	Affx-110213136	5D	467403585
AX-111785526	G/G	A/G	G/G	Affx-111462801	5D	232007032
AX-111802962	A/G	A/A	A/G	Affx-110248324	5D	187966401
AX-111806106	G/G	T/G	G/G	Affx-88670865	5D	131404225
AX-111820017	G/G	C/G	G/G	Affx-110337897	5D	218945113
AX-111833356	C/C	C/C	C/C	Affx-111008365	5D	210218814
AX-111841659	A/A	A/G	A/A	Affx-88644603	5D	502712815
AX-111860462	G/G	A/G	G/G	Affx-88478363	5D	337573520
AX-111869250	G/G	A/G	G/G	Affx-110465285	5D	445125196
AX-111881147	G/G	A/G	G/G	Affx-109894076	5D	482435875
AX-111881207	C/C	T/C	C/C	Affx-88624223	5D	125350806
AX-111903917	G/G	G/G	A/A	Affx-109964102	5D	386126820
AX-111904694	A/A	A/G	A/A	Affx-110215498	5D	249198846

AX-111905082	G/G	T/T	G/G	Affx-88643267	5D	394099902
AX-111906637	T/T	T/C	T/T	Affx-111836242	5D	202250871
AX-111917011	A/A	A/A	A/A	Affx-111470467	5D	462565152
AX-111988475	C/C	T/C	C/C	Affx-110557977	5D	79566961
AX-179559343	A/G	A/A	A/A	Affx-88607356	5D	244644291
AX-181995361	A/A	A/G	A/A	Affx-88652539	5D	486241669
AX-182064510	A/G	A/G	A/G	Affx-472311369	5D	60247934
AX-182066044	G/G	A/G	G/G	Affx-472313674	5D	501029446
AX-86171850	T/C	T/C	T/C	Affx-88543461	5D	437807470
AX-86185855	T/T	C/C	T/T	Affx-111439098	5D	135645455
AX-89336793	T/T	T/C	T/T	Affx-92718712	5D	489775906
AX-89349755	T/T	T/C	T/T	Affx-88381560	5D	250056746
AX-89369050	A/A	A/G	A/A	Affx-88401239	5D	426673650
AX-89369759	G/G	A/G	G/G	Affx-88401952	5D	360894184
AX-89390905	G/G	A/G	G/G	Affx-109659228	5D	8929907
AX-89420236	G/G	A/G	G/G	Affx-88452882	5D	162373028
AX-89431101	G/G	T/G	G/G	Affx-88463796	5D	15853242
AX-89451602	T/T	T/C	T/T	Affx-88484363	5D	496978212
AX-89484555	T/T	C/C	T/T	Affx-109099809	5D	412353356
AX-89485573	G/G	A/G	G/G	Affx-111714561	5D	61460997
AX-89489968	A/A	A/G	A/A	Affx-88522832	5D	27803137
AX-89500015	C/C	T/C	T/T	Affx-92566284	5D	552040110
AX-89564454	T/T	T/G	T/T	Affx-88597504	5D	35848644
AX-89591395	G/G	A/G	G/G	Affx-88624529	5D	290069056
AX-89603436	G/G	A/G	G/G	Affx-88636602	5D	37133842
AX-89633041	G/G	C/G	G/G	Affx-88666201	5D	7422654
AX-89640152	G/G	A/G	G/G	Affx-88672941	5D	58248663
AX-89642798	C/C	T/C	C/C	Affx-88675593	5D	339375197
AX-89651374	C/C	T/C	C/C	Affx-88684170	5D	43096805
AX-89655686	C/C	T/C	C/C	Affx-88688476	5D	370827067
AX-89700472	C/C	T/C	C/C	Affx-88733207	5D	11852387
AX-89744839	T/T	T/C	C/C	Affx-88777527	5D	391338720
AX-89753391	A/A	A/G	A/A	Affx-88786086	5D	499456685
AX-89765755	T/T	T/C	T/T	Affx-88796572	5D	423334940
AX-94409094	T/C	T/C	T/C	Affx-92198246	5D	399148417
AX-94415907	A/G	A/A	A/G	Affx-92916718	5D	356844248
AX-94418907	T/T	C/C	T/T	Affx-92843920	5D	3609842
AX-94421222	C/G	C/G	C/G	Affx-92145670	5D	6446076
AX-94439197	A/G	A/G	A/G	Affx-92765654	5D	384071457
AX-94504234	T/G	T/T	T/G	Affx-92230416	5D	428496395
AX-94524902	A/A	A/C	A/A	Affx-92452824	5D	547502682
AX-94557117	C/C	T/T	C/C	Affx-92472825	5D	375193851
AX-94645340	C/C	G/G	G/G	Affx-92223010	5D	489899802
AX-94663230	C/C	C/C	C/C	Affx-92902415	5D	28447906
AX-94667558	G/G	G/G	G/G	Affx-92208752	5D	3591858
AX-94679604	A/G	G/G	A/G	Affx-92880699	5D	503663988
AX-94730362	G/G	T/G	G/G	Affx-92281759	5D	3637226
AX-94732674	A/G	A/G	A/G	Affx-92927876	5D	9731646
AX-94771499	T/T	T/T	T/T	Affx-88697079	5D	445319209
AX-94823785	A/A	A/G	G/G	Affx-92819364	5D	480477333
AX-94840001	G/G	A/G	G/G	Affx-92629108	5D	362885623
AX-94846184	T/T	T/T	T/T	Affx-92203064	5D	3609719
AX-94848564	C/C	C/C	C/C	Affx-92172189	5D	450218182
AX-94866715	T/T	T/C	T/T	Affx-92726531	5D	284183683

AX-94914596	C/C	C/C	A/A	Affx-92190679	5D	549810893
AX-94936563	C/C	T/T	T/C	Affx-92666327	5D	381026442
AX-94943802	G/G	G/G	G/G	Affx-92943863	5D	3637886
AX-94969919	C/C	C/G	C/C	Affx-92391259	5D	475306639
AX-94972062	T/T	T/C	T/T	Affx-92778701	5D	3592032
AX-94984296	T/T	T/C	T/T	Affx-92707434	5D	91553414
AX-95008871	T/C	C/C	T/C	Affx-88741249	5D	192270194
AX-95017057	C/C	C/C	C/C	Affx-92487283	5D	3591847
AX-95117653	T/C	T/T	T/C	Affx-92913563	5D	409878960
AX-95151231	G/G	A/G	G/G	Affx-92528389	5D	3609767
AX-95200870	T/C	T/T	C/C	Affx-92916220	5D	479879441
AX-95229346	A/G	G/G	A/G	Affx-92879568	5D	362055466
AX-95246226	C/C	T/C	C/C	Affx-92306660	5D	3591782
AX-95658211	T/G	T/T	T/G	Affx-88446571	5D	520779781
AX-95659657	T/C	T/C	T/C	Affx-88768686	5D	506074431
AX-108729874	A/A	A/G	A/A	Affx-88359626	6A	605339700
AX-108736426	A/C	A/C	A/A	Affx-88634675	6A	615739593
AX-108755308	G/G	G/G	G/G	Affx-88686604	6A	511386261
AX-108758959	A/A	A/A	C/C	Affx-108997331	6A	75570466
AX-108767470	G/G	A/G	G/G	Affx-111995219	6A	455991636
AX-108790191	C/C	T/C	C/C	Affx-109557980	6A	435829949
AX-108794894	G/G	A/A	G/G	Affx-88715986	6A	529287926
AX-108799082	T/T	T/C	C/C	Affx-110509414	6A	568161303
AX-108800001	T/T	T/C	T/T	Affx-108970784	6A	427032287
AX-108800286	G/G	G/G	G/G	Affx-111840824	6A	335807066
AX-108813367	G/G	T/G	G/G	Affx-109998209	6A	515677079
AX-108815103	A/A	G/G	G/G	Affx-110573165	6A	73652625
AX-108817301	A/A	A/G	A/A	Affx-109575543	6A	341957483
AX-108820938	A/A	A/G	A/A	Affx-109691204	6A	404451680
AX-108831829	T/T	T/C	T/T	Affx-109000300	6A	555967861
AX-108836992	T/C	T/T	T/C	Affx-109867560	6A	84968548
AX-108842571	T/T	T/C	T/T	Affx-110794351	6A	249747986
AX-108847140	T/T	T/C	T/T	Affx-110571007	6A	418544355
AX-108869501	A/A	A/G	A/A	Affx-110595676	6A	338237015
AX-108876381	G/G	A/G	G/G	Affx-109385837	6A	48627792
AX-108876702	C/C	A/C	C/C	Affx-109811186	6A	495690446
AX-108884190	A/A	A/G	A/A	Affx-109605541	6A	560103470
AX-108891954	A/A	A/G	A/A	Affx-109001217	6A	587549709
AX-108895618	C/C	T/C	C/C	Affx-111617160	6A	517256874
AX-108911940	G/G	A/G	G/G	Affx-109857688	6A	332602380
AX-108914894	G/G	A/G	G/G	Affx-110933543	6A	412848027
AX-108917469	T/T	A/T	T/T	Affx-111505176	6A	597992358
AX-108919789	G/G	A/G	G/G	Affx-110618331	6A	430516320
AX-108930113	G/G	G/G	G/G	Affx-88510782	6A	330057165
AX-108932983	A/A	A/G	A/A	Affx-110425812	6A	594832326
AX-108933121	T/T	T/T	C/C	Affx-111262762	6A	16593556
AX-108936268	C/C	T/C	C/C	Affx-109291636	6A	502146344
AX-108937778	G/G	A/G	G/G	Affx-110733180	6A	327484309
AX-108939771	C/C	T/C	T/T	Affx-110615202	6A	8165603
AX-108944873	T/T	A/T	T/T	Affx-110105366	6A	601258225
AX-108949929	A/A	A/G	A/A	Affx-88360822	6A	237729748
AX-108952456	G/G	A/A	G/G	Affx-111913803	6A	516223874
AX-108958326	C/C	T/T	C/C	Affx-111316849	6A	456330273
AX-108964970	C/C	T/C	C/C	Affx-108916415	6A	320217958

AX-108965821	C/C	A/C	C/C	Affx-111045917	6A	600070296
AX-108969345	T/T	T/C	T/T	Affx-111805577	6A	190583889
AX-108975012	T/T	T/C	T/T	Affx-110734081	6A	303534001
AX-108975281	A/A	A/G	A/A	Affx-109807892	6A	292794082
AX-108982634	C/C	A/A	C/C	Affx-109831686	6A	573480204
AX-108988699	A/A	A/C	C/C	Affx-110374689	6A	68626300
AX-108991354	A/A	A/G	A/A	Affx-110213418	6A	306048895
AX-109008942	G/G	A/G	G/G	Affx-93002948	6A	544151382
AX-109022946	A/A	G/G	A/A	Affx-109163827	6A	550720077
AX-109037695	G/G	G/G	G/G	Affx-110189119	6A	238165490
AX-109040647	G/G	A/G	G/G	Affx-109512120	6A	315119937
AX-109043962	A/A	A/C	A/A	Affx-111753872	6A	352377413
AX-109061200	A/A	A/G	A/A	Affx-111632281	6A	237728800
AX-109063087	T/T	T/C	T/T	Affx-109293770	6A	419425586
AX-109063873	C/C	A/C	C/C	Affx-111206459	6A	433569417
AX-109070240	T/T	T/G	T/T	Affx-110309746	6A	310060628
AX-109071668	G/G	A/G	G/G	Affx-109615816	6A	544611830
AX-109077724	T/T	T/C	T/T	Affx-110508996	6A	385791611
AX-109084122	A/A	A/A	A/A	Affx-109273909	6A	374826615
AX-109086661	G/G	C/G	G/G	Affx-109260800	6A	366670047
AX-109093066	T/T	T/C	T/T	Affx-111221308	6A	237647623
AX-109094008	T/T	T/C	T/T	Affx-111770401	6A	440810433
AX-109272810	C/C	A/C	C/C	Affx-111730912	6A	520671232
AX-109280410	A/A	A/A	A/A	Affx-88735303	6A	531001811
AX-109288606	C/C	C/C	C/C	Affx-109118924	6A	20880738
AX-109291122	G/G	A/G	A/A	Affx-110689669	6A	570394656
AX-109295804	G/G	T/G	T/T	Affx-111842353	6A	585013842
AX-109302448	T/T	C/C	T/T	Affx-109345847	6A	547482441
AX-109304197	T/T	T/C	T/T	Affx-109067570	6A	558984034
AX-109304510	G/G	A/G	G/G	Affx-111193169	6A	452638948
AX-109334618	T/T	T/G	T/T	Affx-111384722	6A	328130345
AX-109345228	G/G	A/G	G/G	Affx-109366650	6A	523552857
AX-109348554	T/T	T/C	T/T	Affx-111866906	6A	318863829
AX-109351164	C/C	C/C	G/G	Affx-88374446	6A	83437991
AX-109353146	G/G	A/G	G/G	Affx-88718454	6A	442400023
AX-109365575	T/T	T/C	T/T	Affx-88699795	6A	441497134
AX-109367127	G/G	A/G	G/G	Affx-109410779	6A	20208936
AX-109368197	T/T	T/C	T/T	Affx-88548029	6A	530018130
AX-109369214	T/T	T/C	T/T	Affx-108941474	6A	362703230
AX-109371563	T/T	T/C	T/T	Affx-111404539	6A	388795386
AX-109376346	A/A	A/A	A/A	Affx-110975286	6A	61748025
AX-109380909	A/A	A/G	A/A	Affx-110704380	6A	381087756
AX-109402855	C/C	C/C	T/T	Affx-109095361	6A	581377569
AX-109409731	C/C	T/C	C/C	Affx-88800263	6A	546600983
AX-109413677	T/T	T/C	T/T	Affx-111149840	6A	341457029
AX-109424753	A/A	A/G	A/A	Affx-109561267	6A	76708283
AX-109440144	G/G	G/G	G/G	Affx-88415827	6A	383079556
AX-109448914	T/T	T/C	T/T	Affx-110623405	6A	349053789
AX-109452842	G/G	A/G	G/G	Affx-109251613	6A	593458385
AX-109478914	T/T	T/G	G/G	Affx-111450071	6A	72308875
AX-109511537	C/C	C/C	C/C	Affx-111464478	6A	396453721
AX-109520642	C/C	T/C	T/T	Affx-111090298	6A	67347005
AX-109527686	T/C	T/C	C/C	Affx-109158420	6A	582640221
AX-109528569	C/C	T/T	C/C	Affx-111661195	6A	549642310

AX-109534275	C/C	T/C	C/C	Affx-109509577	6A	604573611
AX-109552826	A/A	A/G	A/A	Affx-109234635	6A	13434303
AX-109579242	T/T	T/T	T/T	Affx-110270588	6A	430481582
AX-109587414	T/T	T/C	T/T	Affx-111143759	6A	557435943
AX-109591350	C/C	C/G	C/C	Affx-109440949	6A	299132338
AX-109592433	A/A	A/C	A/A	Affx-111356356	6A	409675308
AX-109605929	C/C	T/C	T/T	Affx-109243971	6A	74974399
AX-109623135	A/A	A/G	A/A	Affx-109017755	6A	364328298
AX-109815694	A/A	A/G	A/A	Affx-88422844	6A	452773710
AX-109820371	T/T	C/C	C/C	Affx-109676137	6A	606608343
AX-109822525	C/C	C/C	C/C	Affx-88637852	6A	27308682
AX-109826393	T/T	T/C	T/T	Affx-111272148	6A	402531319
AX-109830450	C/C	C/G	C/C	Affx-111761268	6A	109699408
AX-109836543	T/T	T/G	T/T	Affx-88536386	6A	453910278
AX-109837588	T/T	C/C	C/C	Affx-110900930	6A	583093375
AX-109840282	A/A	A/A	A/A	Affx-110052752	6A	20746677
AX-109840421	T/C	T/C	T/C	Affx-88572155	6A	536023376
AX-109841642	G/G	C/C	G/G	Affx-110032518	6A	93148851
AX-109846824	T/T	T/T	T/T	Affx-110521136	6A	397959733
AX-109848999	A/A	A/G	A/A	Affx-109879117	6A	354778024
AX-109850660	C/C	C/G	G/G	Affx-110818062	6A	24331268
AX-109859002	A/A	A/G	A/A	Affx-109281767	6A	537250156
AX-109861503	C/C	C/G	C/C	Affx-110890730	6A	437023433
AX-109863766	A/A	A/G	A/A	Affx-111493448	6A	316056716
AX-109869385	A/A	A/C	A/A	Affx-88676855	6A	522043305
AX-109884501	T/T	T/C	T/T	Affx-111065331	6A	436413100
AX-109894066	C/C	A/C	C/C	Affx-110970369	6A	525763348
AX-109894900	G/G	G/G	G/G	Affx-109358796	6A	84912436
AX-109896657	C/C	A/C	C/C	Affx-111850679	6A	607393131
AX-109902225	C/C	C/C	C/C	Affx-110993144	6A	599614662
AX-109903000	T/T	T/C	T/T	Affx-111422052	6A	302251872
AX-109903525	G/G	G/G	G/G	Affx-88452191	6A	18359552
AX-109912963	G/G	A/G	G/G	Affx-110743857	6A	370603369
AX-109918284	A/A	A/G	A/A	Affx-109156866	6A	300755858
AX-109921374	T/T	T/C	T/T	Affx-109559104	6A	596705016
AX-109926846	A/A	A/G	A/A	Affx-109589137	6A	414990865
AX-109933415	C/C	T/C	T/T	Affx-110139062	6A	66564615
AX-109934896	C/C	C/C	C/C	Affx-111943021	6A	614482543
AX-109935411	A/G	A/G	G/G	Affx-110182809	6A	613483176
AX-109939150	A/A	A/G	A/A	Affx-110964775	6A	359452409
AX-109945193	C/C	A/C	C/C	Affx-109101128	6A	533278906
AX-109947015	A/A	A/G	A/A	Affx-111953026	6A	556516922
AX-109961864	G/G	G/G	G/G	Affx-111338254	6A	348525936
AX-109967817	A/A	A/G	A/A	Affx-109397398	6A	316147992
AX-109985468	A/A	A/G	A/A	Affx-111808698	6A	356426724
AX-109994317	G/G	A/G	G/G	Affx-108888407	6A	532029964
AX-109999937	T/T	T/C	C/C	Affx-109843580	6A	445407910
AX-110005441	C/C	C/G	C/C	Affx-108864896	6A	364301782
AX-110007634	C/C	C/G	C/C	Affx-109433483	6A	380096716
AX-110024798	G/G	C/G	G/G	Affx-111431520	6A	324812584
AX-110025367	G/G	A/G	G/G	Affx-109736980	6A	237740291
AX-110067643	T/T	C/C	T/T	Affx-110282562	6A	299981580
AX-110077933	T/T	T/T	T/T	Affx-111936316	6A	496623764
AX-110087250	G/G	C/C	G/G	Affx-109705758	6A	525761014

AX-110111679	C/C	A/C	C/C	Affx-111463928	6A	100088198
AX-110129241	A/A	A/C	A/A	Affx-109616132	6A	306943674
AX-110135035	T/T	T/C	T/T	Affx-111318705	6A	421631671
AX-110139825	G/G	A/G	A/A	Affx-111696851	6A	617246581
AX-110150297	T/T	C/C	T/T	Affx-109511786	6A	364861727
AX-110151642	T/T	T/C	T/T	Affx-109165984	6A	296195705
AX-110152115	T/C	T/C	T/T	Affx-111792990	6A	615038714
AX-110158467	A/A	A/G	A/A	Affx-109623986	6A	552227712
AX-110165332	A/A	A/C	C/C	Affx-88467966	6A	570384523
AX-110167976	A/A	A/G	A/A	Affx-111611962	6A	395085734
AX-110174295	T/T	T/C	T/T	Affx-109169154	6A	521025786
AX-110196810	A/A	A/G	A/A	Affx-111754839	6A	535655702
AX-110362080	T/T	T/T	T/T	Affx-109290246	6A	361640602
AX-110385344	T/T	T/G	T/T	Affx-88677145	6A	107691933
AX-110398932	C/C	C/G	C/C	Affx-111607838	6A	420820706
AX-110400957	G/G	C/G	G/G	Affx-110882572	6A	552706707
AX-110419959	G/G	T/T	G/G	Affx-110046442	6A	499641383
AX-110428139	G/G	G/G	G/G	Affx-111844350	6A	344829509
AX-110431275	C/C	T/C	T/T	Affx-110172459	6A	571054819
AX-110436436	T/C	T/C	T/T	Affx-88646656	6A	942870
AX-110437185	C/C	C/G	C/C	Affx-109000997	6A	401607947
AX-110445351	A/A	A/C	C/C	Affx-108996432	6A	68423592
AX-110451033	A/A	A/G	A/A	Affx-110141901	6A	502889163
AX-110451544	C/C	T/C	C/C	Affx-108877941	6A	52954703
AX-110452751	C/C	T/C	T/T	Affx-111022634	6A	609340856
AX-110467915	G/G	G/G	G/G	Affx-110310701	6A	423307219
AX-110469098	T/C	T/T	T/C	Affx-109312217	6A	233809045
AX-110474238	A/A	A/G	A/A	Affx-111190282	6A	410272068
AX-110482082	T/T	T/C	T/T	Affx-111854574	6A	237479423
AX-110492067	A/A	A/C	A/A	Affx-111239736	6A	500363969
AX-110492544	G/G	G/G	G/G	Affx-111927520	6A	61137866
AX-110495466	G/G	G/G	G/G	Affx-109439007	6A	573142816
AX-110496796	T/T	T/C	C/C	Affx-109826641	6A	616860002
AX-110509224	T/T	T/C	T/T	Affx-109876886	6A	371722098
AX-110510229	C/C	C/G	C/C	Affx-109466835	6A	447632581
AX-110513379	G/G	A/G	G/G	Affx-110395340	6A	398180998
AX-110515380	C/C	T/T	C/C	Affx-111277523	6A	536570469
AX-110516770	T/T	T/C	T/T	Affx-110502613	6A	479317002
AX-110542807	A/A	A/A	A/A	Affx-111750214	6A	54977694
AX-110574258	G/G	C/G	C/C	Affx-88744080	6A	564725748
AX-110589298	A/A	G/G	A/A	Affx-110100993	6A	302666510
AX-110593017	T/T	T/C	T/T	Affx-110757314	6A	245781410
AX-110600929	A/A	C/C	A/A	Affx-110969974	6A	592498314
AX-110601472	C/C	A/C	C/C	Affx-109255193	6A	481582261
AX-110608637	C/C	T/C	C/C	Affx-110118362	6A	577836465
AX-110619257	A/G	G/G	G/G	Affx-88703758	6A	105181270
AX-110625337	A/A	A/A	A/A	Affx-109246543	6A	11961812
AX-110627175	C/C	T/C	T/T	Affx-88682461	6A	26932503
AX-110640576	G/G	C/C	C/C	Affx-111027854	6A	124268452
AX-110675630	T/T	T/C	C/C	Affx-110477141	6A	1629012
AX-110679439	T/T	T/C	T/T	Affx-111819011	6A	411231357
AX-110683634	C/C	T/C	C/C	Affx-108964660	6A	330790939
AX-110699549	A/A	A/G	A/A	Affx-110769284	6A	336667190
AX-110704425	A/A	A/G	A/A	Affx-110923180	6A	372026753

AX-110714910	T/T	T/C	T/T	Affx-111298124	6A	399621955
AX-110717166	C/C	T/C	C/C	Affx-109315353	6A	479994550
AX-110725457	C/C	T/C	T/T	Affx-110789991	6A	616415453
AX-110729210	C/C	T/C	C/C	Affx-111189302	6A	417449780
AX-110732929	A/A	A/G	A/A	Affx-111491369	6A	438691909
AX-110733526	T/T	T/C	T/T	Affx-110701819	6A	442024552
AX-110740737	C/C	T/C	C/C	Affx-111119921	6A	544863112
AX-110746199	G/G	T/G	G/G	Affx-88754999	6A	307053361
AX-110905863	T/T	T/C	T/T	Affx-109050858	6A	522639222
AX-110916005	G/G	C/G	G/G	Affx-110553218	6A	330873938
AX-110918702	G/G	C/G	G/G	Affx-88348686	6A	360530384
AX-110922589	T/T	T/C	T/T	Affx-109214218	6A	439651652
AX-110925472	G/G	C/C	G/G	Affx-110116563	6A	53500065
AX-110926805	T/T	T/G	T/T	Affx-109668441	6A	551296559
AX-110933588	T/T	G/G	T/T	Affx-108923926	6A	386403294
AX-110937386	G/G	T/G	G/G	Affx-111506970	6A	454649374
AX-110941753	C/C	C/G	C/C	Affx-110635356	6A	416958959
AX-110942819	G/G	G/G	T/T	Affx-109410463	6A	31874326
AX-110942969	G/G	C/G	C/C	Affx-109825601	6A	428454560
AX-110953968	C/C	C/G	G/G	Affx-110697909	6A	7725011
AX-110955246	G/G	A/G	G/G	Affx-109215491	6A	540625176
AX-110960515	C/C	T/C	C/C	Affx-109687736	6A	538493949
AX-110985951	C/C	C/G	C/C	Affx-111972549	6A	447416422
AX-110988853	A/A	A/A	A/A	Affx-111695208	6A	427606644
AX-110989256	G/G	A/G	G/G	Affx-111757010	6A	426052861
AX-110995858	T/T	T/C	T/T	Affx-110013361	6A	71705701
AX-110996452	T/T	T/T	T/T	Affx-111111203	6A	437766897
AX-111000070	G/G	A/G	G/G	Affx-109224838	6A	438270778
AX-111001517	A/A	A/G	A/A	Affx-108963839	6A	415103145
AX-111012430	C/C	T/C	C/C	Affx-108915708	6A	542327730
AX-111016902	C/C	T/C	C/C	Affx-109553391	6A	421369842
AX-111023212	A/A	A/C	A/A	Affx-111907830	6A	425052070
AX-111023474	T/T	T/C	T/T	Affx-110780640	6A	382524972
AX-111024457	C/C	C/G	C/C	Affx-109129823	6A	549172320
AX-111040003	C/C	T/C	C/C	Affx-110788084	6A	7369285
AX-111042006	C/C	T/C	T/T	Affx-109039981	6A	591897896
AX-111067830	T/C	T/C	T/C	Affx-110175073	6A	266971953
AX-111069725	C/C	T/C	C/C	Affx-109416706	6A	335560361
AX-111070931	G/G	A/G	G/G	Affx-109703674	6A	413877524
AX-111072559	A/A	A/G	A/A	Affx-111666705	6A	398796577
AX-111077590	T/T	T/C	T/T	Affx-109037329	6A	582941584
AX-111080243	T/T	T/C	T/T	Affx-111340558	6A	423523213
AX-111080943	C/C	C/G	C/C	Affx-109637257	6A	455497558
AX-111084180	T/T	T/C	T/T	Affx-111854957	6A	543400258
AX-111087643	G/G	A/G	G/G	Affx-110282595	6A	555709730
AX-111113291	T/T	T/T	T/T	Affx-109606270	6A	11162507
AX-111119782	C/C	T/C	C/C	Affx-111778306	6A	553142535
AX-111119786	T/T	T/C	T/T	Affx-110293182	6A	382655277
AX-111120402	C/C	C/C	C/C	Affx-111057449	6A	600456941
AX-111124455	T/T	A/T	T/T	Affx-110479972	6A	433964450
AX-111131762	G/G	G/G	G/G	Affx-111388284	6A	562466975
AX-111137643	T/T	T/C	T/T	Affx-109610314	6A	539195629
AX-111140750	C/C	C/G	C/C	Affx-109884904	6A	360237676
AX-111146080	T/T	T/C	T/T	Affx-111253274	6A	259315472

AX-111171333	C/C	A/C	C/C	Affx-111261664	6A	545837569
AX-111214686	T/T	T/T	T/T	Affx-111187146	6A	310507515
AX-111225267	G/G	A/G	G/G	Affx-109176745	6A	326068779
AX-111226349	G/G	G/G	G/G	Affx-109796173	6A	367494684
AX-111228628	T/T	T/C	T/T	Affx-110233630	6A	329070240
AX-111254008	G/G	T/G	G/G	Affx-110966705	6A	331577764
AX-111256020	T/T	T/G	T/T	Affx-111472989	6A	395846869
AX-111264704	T/T	T/C	T/T	Affx-111443072	6A	305038728
AX-111267479	A/A	A/A	A/A	Affx-109156181	6A	326823896
AX-111273273	T/T	T/C	T/T	Affx-109229208	6A	340704113
AX-111277473	T/T	C/C	C/C	Affx-108899570	6A	17594640
AX-111278973	A/A	A/G	A/A	Affx-111233290	6A	336253032
AX-111460761	G/G	T/G	G/G	Affx-109181359	6A	556910892
AX-111475401	A/A	A/T	A/A	Affx-111458600	6A	531410262
AX-111475953	C/C	C/C	C/C	Affx-111752958	6A	39050466
AX-111478362	A/A	A/G	A/A	Affx-110659934	6A	318287443
AX-111479914	G/G	A/G	A/A	Affx-109785737	6A	608863690
AX-111482414	G/G	A/G	G/G	Affx-111875886	6A	416377937
AX-111486809	T/T	T/C	T/T	Affx-111860729	6A	314551456
AX-111490517	G/G	A/G	A/A	Affx-110073428	6A	67464465
AX-111493438	C/C	T/C	C/C	Affx-88746547	6A	551654022
AX-111496708	T/T	T/G	G/G	Affx-109163629	6A	31755825
AX-111498896	T/T	T/C	T/T	Affx-108940807	6A	394065505
AX-111515803	A/A	A/G	A/A	Affx-109256626	6A	499402575
AX-111516383	C/C	T/C	T/T	Affx-88508526	6A	572137290
AX-111534811	G/G	A/G	G/G	Affx-111341364	6A	495840678
AX-111541600	G/G	A/G	G/G	Affx-111523476	6A	340919186
AX-111542381	G/G	A/G	G/G	Affx-88562826	6A	532432256
AX-111545289	C/C	C/C	C/C	Affx-108938263	6A	354716389
AX-111557233	A/A	A/G	G/G	Affx-88342126	6A	610066391
AX-111564273	A/A	A/G	A/A	Affx-109238288	6A	17947396
AX-111576549	G/G	A/G	G/G	Affx-110582791	6A	524067851
AX-111579006	G/G	A/G	G/G	Affx-111491074	6A	541425398
AX-111588952	G/G	A/G	G/G	Affx-109738111	6A	560241667
AX-111592631	C/C	T/C	C/C	Affx-111891129	6A	474585000
AX-111594972	T/T	T/C	T/T	Affx-109833619	6A	436975681
AX-111606694	G/G	A/G	G/G	Affx-88462888	6A	541401338
AX-111607630	A/A	A/G	A/A	Affx-88773421	6A	12842946
AX-111607947	T/T	T/C	T/T	Affx-110331879	6A	396606755
AX-111621532	C/C	T/C	T/T	Affx-110075127	6A	585498798
AX-111629868	C/C	T/C	C/C	Affx-109124927	6A	540041960
AX-111639623	T/C	C/C	T/C	Affx-109810721	6A	596350334
AX-111674966	A/A	A/A	A/A	Affx-109764203	6A	450462178
AX-111680204	T/T	C/C	C/C	Affx-110172496	6A	584508604
AX-111683356	T/T	T/C	T/T	Affx-110171801	6A	605874619
AX-111685841	T/T	T/C	T/T	Affx-109096397	6A	358591651
AX-111687133	A/A	A/G	A/A	Affx-110797344	6A	521460948
AX-111698356	T/T	T/T	T/T	Affx-110425272	6A	407940762
AX-111708346	G/G	A/G	G/G	Affx-88507641	6A	363522403
AX-111722073	T/G	G/G	T/G	Affx-111256462	6A	237734914
AX-111727298	A/A	A/A	A/A	Affx-109677966	6A	411086966
AX-111751043	G/G	A/G	G/G	Affx-88702231	6A	451989117
AX-111756143	G/G	G/G	G/G	Affx-109989736	6A	238166114
AX-111760775	T/T	T/C	T/T	Affx-108870654	6A	600369472

AX-111764165	A/A	A/G	G/G	Affx-110841027	6A	567807910
AX-111780081	C/C	T/C	T/T	Affx-111738371	6A	579819505
AX-111786353	T/T	T/C	T/T	Affx-110589055	6A	369061267
AX-111787297	T/T	T/C	T/T	Affx-109042973	6A	425330309
AX-111801320	A/A	A/G	A/A	Affx-110681531	6A	378218123
AX-111803864	G/G	A/A	G/G	Affx-111138904	6A	256754923
AX-111806667	A/A	A/G	A/A	Affx-110945724	6A	309291590
AX-111807548	A/A	A/C	A/A	Affx-109459548	6A	390557495
AX-111818250	A/A	A/C	A/A	Affx-111864069	6A	316545919
AX-111835367	T/T	T/C	T/T	Affx-109431470	6A	349835322
AX-179477168	C/C	T/C	C/C	Affx-292486499	6A	309814047
AX-179558759	T/T	T/G	T/T	Affx-88766095	6A	484276131
AX-179562467	T/T	T/C	T/T	Affx-292486558	6A	230699220
AX-182166197	G/G	G/G	G/G	Affx-472312406	6A	8997394
AX-86164404	C/C	C/C	T/T	Affx-111197989	6A	595916734
AX-86167031	A/A	A/G	A/A	Affx-92715865	6A	420092601
AX-86173631	T/T	C/C	T/T	Affx-88524919	6A	601473116
AX-89439930	A/A	G/G	A/A	Affx-88472656	6A	574385861
AX-89529042	C/C	C/C	C/C	Affx-88561984	6A	57726863
AX-89610547	A/C	A/C	A/A	Affx-88643726	6A	564585251
AX-89648484	G/G	T/G	G/G	Affx-88681282	6A	457660545
AX-89688808	A/A	A/A	A/A	Affx-88721570	6A	6725011
AX-94424031	C/C	C/C	C/C	Affx-92867468	6A	370466577
AX-94443076	T/T	T/C	T/T	Affx-92255945	6A	424093750
AX-94510892	T/T	T/T	T/T	Affx-92244675	6A	112585030
AX-94513257	T/C	T/C	T/C	Affx-92841122	6A	47106025
AX-94531491	C/C	T/C	C/C	Affx-92686504	6A	121668141
AX-94570509	T/T	T/T	T/T	Affx-92143574	6A	13072586
AX-94580424	A/C	C/C	A/C	Affx-92289591	6A	77538552
AX-94581785	G/G	G/G	G/G	Affx-92251190	6A	601873178
AX-94630995	T/G	T/G	T/T	Affx-88418300	6A	7595547
AX-94653398	T/T	G/G	G/G	Affx-92699422	6A	4678856
AX-94788907	T/G	T/T	T/G	Affx-92129559	6A	604107719
AX-94818186	T/T	T/T	T/T	Affx-92124319	6A	501801411
AX-94920262	A/G	G/G	A/G	Affx-92770722	6A	58149816
AX-94952400	A/A	A/G	A/A	Affx-88687125	6A	299335643
AX-94968471	G/G	G/G	G/G	Affx-92246648	6A	3295208
AX-94971944	C/C	C/G	C/C	Affx-92574799	6A	448230743
AX-95012251	G/G	A/G	A/A	Affx-92385227	6A	65845842
AX-95080022	T/C	T/C	T/C	Affx-92708204	6A	201091096
AX-95090235	C/G	G/G	C/C	Affx-92692098	6A	610898015
AX-95124889	A/C	C/C	A/C	Affx-92302008	6A	27748586
AX-95180452	A/A	A/A	A/A	Affx-92516014	6A	483899492
AX-95659661	A/A	A/G	G/G	Affx-88567695	6A	563558658
AX-95684870	G/G	A/G	G/G	Affx-88449917	6A	381025610
AX-108728117	C/C	T/C	C/C	Affx-111684449	6B	521292429
AX-108731300	G/G	C/G	G/G	Affx-111102213	6B	517104958
AX-108732920	T/T	T/C	T/T	Affx-110546585	6B	576825870
AX-108733957	T/T	T/C	T/T	Affx-109752686	6B	120671867
AX-108736410	G/G	A/G	G/G	Affx-108884223	6B	106054976
AX-108739547	G/G	C/G	G/G	Affx-109520424	6B	670210649
AX-108744211	T/T	T/C	T/T	Affx-111617749	6B	66670169
AX-108747764	C/C	C/G	C/C	Affx-111995042	6B	389112882
AX-108750365	C/C	T/C	C/C	Affx-110704114	6B	383523509

AX-108750902	C/C	C/G	C/C	Affx-111159934	6B	330222521
AX-108753446	A/A	A/G	A/A	Affx-110119661	6B	172462787
AX-108759355	C/G	C/G	C/G	Affx-111951013	6B	31781414
AX-108761692	G/G	A/G	G/G	Affx-109382605	6B	198912500
AX-108764548	G/G	A/G	G/G	Affx-110342279	6B	94172404
AX-108764912	C/C	G/G	C/C	Affx-109111970	6B	91721511
AX-108766147	G/G	A/G	G/G	Affx-109406095	6B	466546569
AX-108768271	G/G	A/G	G/G	Affx-110087730	6B	651887533
AX-108770725	G/G	A/G	G/G	Affx-109120713	6B	68046247
AX-108775111	T/T	T/C	T/T	Affx-109447755	6B	78289784
AX-108776690	G/G	A/G	G/G	Affx-109614163	6B	41200255
AX-108777810	T/T	T/C	T/T	Affx-109143008	6B	163652142
AX-108782817	G/G	G/G	G/G	Affx-110932877	6B	393687084
AX-108784464	T/T	T/T	T/T	Affx-109038001	6B	482876116
AX-108784743	G/G	A/G	G/G	Affx-110515791	6B	562533287
AX-108792754	A/A	A/G	A/A	Affx-109584096	6B	346131789
AX-108793024	C/C	T/C	C/C	Affx-110104769	6B	534906143
AX-108793108	T/T	T/C	T/T	Affx-109077236	6B	643788449
AX-108793358	T/T	A/T	T/T	Affx-109754646	6B	676812935
AX-108795710	C/C	T/C	C/C	Affx-110896695	6B	208894886
AX-108797662	C/C	T/C	C/C	Affx-109627815	6B	302600192
AX-108806747	A/A	A/G	A/A	Affx-108919108	6B	691612891
AX-108811307	G/G	A/G	G/G	Affx-109936430	6B	167360426
AX-108814212	G/G	A/G	G/G	Affx-110186565	6B	46691956
AX-108816054	A/A	A/A	A/A	Affx-109446544	6B	469779289
AX-108819892	A/A	G/G	A/G	Affx-109408919	6B	15081521
AX-108823000	C/C	A/C	C/C	Affx-111021781	6B	386423715
AX-108823280	C/C	T/C	C/C	Affx-111249348	6B	648193691
AX-108826673	A/A	A/C	A/A	Affx-88484182	6B	491959586
AX-108828297	A/A	A/G	A/A	Affx-110641837	6B	679548098
AX-108837662	A/A	A/G	A/A	Affx-110547323	6B	572770512
AX-108838340	G/G	T/T	G/G	Affx-109338273	6B	83339316
AX-108842014	T/T	T/C	T/T	Affx-111187132	6B	384600899
AX-108846022	G/G	G/G	G/G	Affx-109208605	6B	611632815
AX-108855599	C/C	C/C	C/C	Affx-110578997	6B	19450945
AX-108855762	A/A	A/G	A/A	Affx-111910703	6B	196911436
AX-108857026	G/G	G/G	G/G	Affx-88350756	6B	686799381
AX-108861803	T/T	T/C	T/T	Affx-109678218	6B	338674224
AX-108872777	A/A	A/G	A/A	Affx-110154473	6B	637046164
AX-108876772	A/A	A/G	A/A	Affx-109383012	6B	617042090
AX-108881023	G/G	G/G	G/G	Affx-110752025	6B	332734177
AX-108882237	A/A	A/G	A/A	Affx-110244131	6B	156400037
AX-108885091	T/T	T/C	T/T	Affx-108907661	6B	184366833
AX-108890743	A/A	A/T	A/A	Affx-109185858	6B	82861792
AX-108894698	A/A	A/A	A/A	Affx-109837226	6B	517780877
AX-108907951	T/T	T/T	T/T	Affx-111190074	6B	242440769
AX-108910452	T/T	T/G	T/T	Affx-88501621	6B	408200939
AX-108920483	G/G	A/G	G/G	Affx-111976946	6B	197238576
AX-108921085	A/A	A/G	A/A	Affx-109400828	6B	610981799
AX-108921495	A/A	G/G	G/G	Affx-109300354	6B	698606601
AX-108931309	A/A	A/G	A/A	Affx-88541895	6B	637478691
AX-108934604	C/C	C/C	C/C	Affx-109838265	6B	171051322
AX-108936780	A/A	A/C	A/A	Affx-88479792	6B	75509734
AX-108948208	G/G	A/G	G/G	Affx-108936361	6B	659920570

AX-108952417	G/G	T/G	G/G	Affx-110562331	6B	304454086
AX-108952758	T/T	T/C	T/T	Affx-109912214	6B	447655779
AX-108955912	C/C	C/C	C/C	Affx-111182803	6B	387564025
AX-108963553	G/G	C/G	G/G	Affx-109556582	6B	464918595
AX-108964461	A/A	A/G	A/A	Affx-110011534	6B	76999002
AX-108965440	T/T	T/T	T/T	Affx-111154466	6B	571046876
AX-108968375	T/T	T/C	T/T	Affx-110033111	6B	151648846
AX-108973596	A/A	A/G	A/A	Affx-109071127	6B	450133833
AX-108988347	C/C	T/C	C/C	Affx-108901524	6B	212564909
AX-109014766	T/T	T/C	T/T	Affx-108880159	6B	607008954
AX-109033363	T/T	T/G	T/T	Affx-110499004	6B	85339506
AX-109035172	C/C	C/C	C/C	Affx-111428706	6B	544463832
AX-109050147	T/T	T/C	T/T	Affx-109308575	6B	570707566
AX-109051503	T/T	C/C	T/T	Affx-109891492	6B	62149640
AX-109052894	A/A	A/G	A/A	Affx-110485285	6B	390068107
AX-109072157	T/T	T/C	T/T	Affx-110374956	6B	331677632
AX-109073134	C/C	T/C	C/C	Affx-109253552	6B	187910855
AX-109075608	C/C	T/C	C/C	Affx-111518367	6B	575814348
AX-109084018	A/A	G/G	A/A	Affx-109050182	6B	111668427
AX-109101466	A/A	A/G	A/A	Affx-111170821	6B	691872423
AX-109103343	C/C	T/C	C/C	Affx-109284101	6B	332056332
AX-109107466	G/G	A/G	G/G	Affx-110647763	6B	103155873
AX-109270335	G/G	A/G	G/G	Affx-111063277	6B	27162320
AX-109273058	T/T	T/C	T/T	Affx-111812524	6B	441590666
AX-109280095	G/G	A/G	G/G	Affx-111135227	6B	151133561
AX-109285445	C/C	A/C	C/C	Affx-110862592	6B	226349693
AX-109287224	A/A	A/G	A/A	Affx-110024907	6B	663645059
AX-109293652	T/T	T/G	T/T	Affx-109779047	6B	310353961
AX-109294614	T/T	C/C	T/T	Affx-110163586	6B	153635489
AX-109300656	G/G	G/G	G/G	Affx-109908039	6B	368961695
AX-109303852	G/G	A/G	G/G	Affx-109521407	6B	18265665
AX-109304547	G/G	A/G	G/G	Affx-109718723	6B	545588920
AX-109306312	T/T	T/C	T/T	Affx-110033935	6B	150475808
AX-109307431	C/C	T/C	C/C	Affx-111481609	6B	614699875
AX-109314339	G/G	A/G	G/G	Affx-111204774	6B	121532728
AX-109317829	G/G	A/G	G/G	Affx-111260713	6B	236329128
AX-109325937	C/C	A/C	C/C	Affx-111738319	6B	57366180
AX-109327918	C/C	A/C	C/C	Affx-109310187	6B	502090933
AX-109332231	C/C	C/C	C/C	Affx-110234528	6B	56012944
AX-109340413	T/T	T/C	T/T	Affx-109994000	6B	342274568
AX-109340938	A/A	A/G	A/A	Affx-111215951	6B	234420881
AX-109348334	T/T	T/C	T/T	Affx-109024931	6B	448463986
AX-109353281	G/G	A/G	G/G	Affx-111798635	6B	575039758
AX-109353543	C/C	T/C	C/C	Affx-110739812	6B	626608920
AX-109354374	G/G	A/G	G/G	Affx-109870272	6B	347659501
AX-109363245	T/T	T/G	T/T	Affx-111425446	6B	305336840
AX-109376175	G/G	A/G	G/G	Affx-109455266	6B	53626758
AX-109379124	C/C	C/C	C/C	Affx-109544577	6B	438360480
AX-109379950	C/C	T/C	C/C	Affx-109311478	6B	96644834
AX-109382255	T/T	T/C	T/T	Affx-111011828	6B	497267595
AX-109384410	C/C	T/C	C/C	Affx-110389243	6B	381333809
AX-109390366	T/T	T/C	T/T	Affx-111218363	6B	84753686
AX-109390939	C/C	T/C	C/C	Affx-110624350	6B	625007429
AX-109391825	G/G	A/A	G/G	Affx-110954700	6B	225147365

AX-109399904	G/G	C/C	G/G	Affx-110614249	6B	351685403
AX-109404434	C/C	T/C	C/C	Affx-108887217	6B	630032518
AX-109407231	T/T	C/C	T/T	Affx-110045056	6B	366689308
AX-109408478	C/C	T/C	C/C	Affx-110525002	6B	118028395
AX-109409036	G/G	A/G	A/G	Affx-111181194	6B	225569689
AX-109410029	A/A	A/G	A/A	Affx-111534502	6B	662881685
AX-109417607	C/C	T/C	C/C	Affx-110728408	6B	365511852
AX-109420976	A/A	A/C	A/A	Affx-111065463	6B	352578533
AX-109421662	A/A	A/G	A/A	Affx-109396587	6B	608715528
AX-109421706	C/C	C/G	C/C	Affx-109831511	6B	114056320
AX-109422502	G/G	G/G	G/G	Affx-111731904	6B	292756488
AX-109428896	T/T	T/G	T/T	Affx-108902957	6B	200387641
AX-109430792	G/G	T/G	G/G	Affx-111532436	6B	401158884
AX-109433229	G/G	G/G	G/G	Affx-110008507	6B	330980444
AX-109433248	T/T	T/C	T/T	Affx-111058832	6B	633545099
AX-109434218	T/T	T/C	T/T	Affx-110472713	6B	194581091
AX-109437939	A/A	C/C	A/A	Affx-108854651	6B	108197361
AX-109443696	A/A	G/G	A/A	Affx-110357023	6B	28982514
AX-109448085	G/G	A/G	G/G	Affx-109400143	6B	286990470
AX-109449869	C/C	C/C	C/C	Affx-108895359	6B	463376756
AX-109455846	G/G	A/G	G/G	Affx-110518165	6B	615972545
AX-109457311	T/T	T/C	T/T	Affx-109226081	6B	480405996
AX-109463287	C/C	T/C	C/C	Affx-111525818	6B	375994480
AX-109464405	A/A	A/G	A/A	Affx-111709579	6B	42489315
AX-109464666	G/G	A/G	G/G	Affx-109950174	6B	169168754
AX-109478686	T/T	T/C	T/T	Affx-111869928	6B	435965553
AX-109487902	C/C	T/C	C/C	Affx-110520706	6B	650042822
AX-109490176	C/C	C/C	C/C	Affx-110480746	6B	593364150
AX-109491130	G/G	A/G	G/G	Affx-110613412	6B	379902151
AX-109491912	C/C	A/C	C/C	Affx-110626030	6B	405874841
AX-109493566	G/G	C/G	G/G	Affx-111754365	6B	81595205
AX-109494940	C/C	T/C	C/C	Affx-110209175	6B	256012610
AX-109500087	C/C	C/C	C/C	Affx-109688688	6B	86094813
AX-109511525	C/C	T/C	C/C	Affx-110251538	6B	185660094
AX-109516374	T/T	T/C	T/T	Affx-109067276	6B	582472494
AX-109516966	C/C	C/G	C/C	Affx-110857911	6B	370445361
AX-109539002	C/C	C/C	C/C	Affx-88399919	6B	703287943
AX-109539352	G/G	T/G	G/G	Affx-111388067	6B	198399152
AX-109546154	A/A	A/G	A/A	Affx-108966630	6B	227069381
AX-109552230	T/T	T/G	T/G	Affx-111804253	6B	714063457
AX-109580213	C/C	T/C	C/C	Affx-88786405	6B	162617855
AX-109597558	A/A	A/G	A/A	Affx-110299650	6B	296361053
AX-109599193	A/A	A/G	A/A	Affx-110887204	6B	480954683
AX-109601574	A/A	A/C	A/A	Affx-111973668	6B	141174195
AX-109602187	C/C	A/A	C/C	Affx-109387241	6B	141925565
AX-109622459	A/A	A/T	A/A	Affx-110910945	6B	34867836
AX-109655924	A/A	A/T	A/A	Affx-88368139	6B	532268087
AX-109818261	C/C	C/C	C/C	Affx-111516781	6B	525996630
AX-109821421	G/G	A/A	G/G	Affx-110964050	6B	293899933
AX-109821745	C/C	C/C	C/C	Affx-109719797	6B	696852835
AX-109823731	C/C	C/G	C/C	Affx-109201127	6B	139891583
AX-109830719	A/A	A/A	A/A	Affx-109961283	6B	696291862
AX-109833239	T/T	T/T	T/T	Affx-111253655	6B	644381391
AX-109834904	G/G	G/G	G/G	Affx-111740554	6B	165499976

AX-109838811	T/T	T/G	T/T	Affx-110700186	6B	372574952
AX-109839736	C/C	A/C	C/C	Affx-111074990	6B	296413207
AX-109843562	C/C	T/C	C/C	Affx-110767478	6B	378248292
AX-109847053	C/C	T/C	C/C	Affx-109827235	6B	568046559
AX-109847998	G/G	T/G	G/G	Affx-110331204	6B	371000124
AX-109853078	A/A	A/G	G/G	Affx-111607993	6B	17498424
AX-109853121	G/G	T/G	G/G	Affx-88779139	6B	689169373
AX-109855319	A/A	A/G	A/A	Affx-109900965	6B	259491584
AX-109855720	T/T	T/C	T/T	Affx-109070598	6B	563996501
AX-109857103	G/G	G/G	G/G	Affx-111318122	6B	231347217
AX-109864053	C/C	A/C	C/C	Affx-111682100	6B	462683689
AX-109865736	C/C	T/C	C/C	Affx-109599313	6B	400311437
AX-109869500	A/A	A/C	A/A	Affx-109661885	6B	446915471
AX-109871902	T/C	C/C	T/C	Affx-109691046	6B	262466793
AX-109881322	T/T	T/C	T/T	Affx-109516935	6B	366261973
AX-109882390	A/A	A/G	A/A	Affx-109283448	6B	119578868
AX-109885280	T/T	T/G	T/T	Affx-110043893	6B	306570804
AX-109891244	C/C	T/C	C/C	Affx-110216831	6B	692260319
AX-109892644	C/C	T/C	C/C	Affx-110130041	6B	625877155
AX-109895054	A/A	A/G	A/A	Affx-110146743	6B	455074189
AX-109895765	G/G	G/G	G/G	Affx-108886793	6B	79169318
AX-109896360	G/G	A/G	G/G	Affx-111512947	6B	373496518
AX-109900320	A/A	A/G	G/G	Affx-111459459	6B	16385951
AX-109904101	T/T	T/C	T/T	Affx-109970539	6B	288129961
AX-109911195	C/C	T/C	C/C	Affx-112004733	6B	354970302
AX-109912504	C/C	T/C	C/C	Affx-111378619	6B	478605789
AX-109924805	C/C	T/C	C/C	Affx-110750038	6B	100162910
AX-109928620	A/A	A/G	A/A	Affx-109890674	6B	455985471
AX-109929885	T/T	T/G	T/T	Affx-111355675	6B	622021029
AX-109931505	T/T	T/T	T/T	Affx-111893041	6B	236221844
AX-109934270	C/C	A/A	C/C	Affx-110820407	6B	113304232
AX-109934300	A/A	A/C	A/A	Affx-111057826	6B	350613854
AX-109935603	A/A	A/G	A/A	Affx-88632281	6B	153407026
AX-109938252	A/A	A/C	A/A	Affx-111156272	6B	116097363
AX-109938641	A/A	A/G	A/A	Affx-109403209	6B	440416518
AX-109942891	G/G	T/G	G/G	Affx-110641230	6B	631358358
AX-109947841	G/G	C/G	G/G	Affx-109230932	6B	93435503
AX-109948619	T/T	A/T	T/T	Affx-109250795	6B	121173700
AX-109948773	A/A	A/G	A/A	Affx-109000143	6B	571664657
AX-109957595	A/A	A/G	A/A	Affx-111262741	6B	539613760
AX-109958534	T/T	T/C	T/T	Affx-110499178	6B	530155586
AX-109959106	T/T	T/C	T/T	Affx-110492862	6B	602182275
AX-109964278	A/A	A/G	A/A	Affx-111466411	6B	217680379
AX-109967135	C/C	C/G	C/C	Affx-110138556	6B	228026011
AX-109983347	T/T	T/C	T/T	Affx-110667865	6B	461592327
AX-109985395	C/C	A/C	C/C	Affx-110013452	6B	14747152
AX-109986783	A/A	A/G	A/A	Affx-111725936	6B	163153220
AX-109987486	C/C	T/C	C/C	Affx-111028663	6B	632966169
AX-109988972	A/A	A/G	A/A	Affx-108949664	6B	405658175
AX-109993782	G/G	A/A	G/G	Affx-110375292	6B	195268193
AX-109994687	C/C	T/C	C/C	Affx-111299324	6B	661463447
AX-109997680	G/G	G/G	G/G	Affx-109061176	6B	286406860
AX-110005847	A/A	A/G	A/A	Affx-88496383	6B	162075503
AX-110010345	G/G	A/G	G/G	Affx-110452712	6B	416666364

AX-110013167	A/A	C/C	A/A	Affx-109978787	6B	609105561
AX-110019687	G/G	G/G	G/G	Affx-111552947	6B	291185561
AX-110024963	C/C	C/G	C/C	Affx-109528599	6B	656594620
AX-110025065	G/G	T/G	G/G	Affx-111956688	6B	457673370
AX-110026281	T/T	T/C	T/T	Affx-109523659	6B	159814397
AX-110027969	C/C	C/C	C/C	Affx-109683060	6B	283255332
AX-110033377	A/A	A/G	A/A	Affx-110921901	6B	333109855
AX-110036577	G/G	C/G	G/G	Affx-110845490	6B	621057667
AX-110038697	G/G	A/G	G/G	Affx-111302982	6B	643567995
AX-110040469	C/C	T/C	C/C	Affx-111563468	6B	683209825
AX-110049114	C/C	T/C	C/C	Affx-88342341	6B	261569015
AX-110049553	A/A	A/A	A/A	Affx-111522680	6B	338191516
AX-110059009	A/A	G/G	G/G	Affx-111260930	6B	17060977
AX-110106058	T/T	T/C	T/T	Affx-88746239	6B	204113701
AX-110131390	C/C	T/C	C/C	Affx-111714714	6B	356387209
AX-110142003	C/C	A/C	C/C	Affx-111739008	6B	314148674
AX-110163999	A/A	A/C	A/A	Affx-111124389	6B	387742671
AX-110192475	T/T	T/C	T/T	Affx-109589466	6B	208147887
AX-110200242	G/G	G/G	G/G	Affx-111346092	6B	117519254
AX-110362828	C/C	C/C	C/C	Affx-110632485	6B	137091867
AX-110365879	C/C	T/T	C/C	Affx-88410670	6B	377931026
AX-110366131	C/C	A/A	C/C	Affx-109033173	6B	703846803
AX-110376502	G/G	G/G	G/G	Affx-110863141	6B	432556815
AX-110376515	T/T	C/C	T/T	Affx-108909327	6B	63999156
AX-110376868	G/G	A/G	G/G	Affx-109298529	6B	176270539
AX-110382845	G/G	G/G	G/G	Affx-109541937	6B	431786367
AX-110383997	G/G	A/G	G/G	Affx-111804385	6B	700774292
AX-110384791	A/A	A/G	A/A	Affx-111889570	6B	102066396
AX-110384871	T/T	T/C	T/T	Affx-109420874	6B	675434394
AX-110392804	G/G	C/G	G/G	Affx-111600041	6B	488802036
AX-110392914	T/T	T/C	T/T	Affx-110956219	6B	298364916
AX-110400198	G/G	A/G	G/G	Affx-109357795	6B	285484036
AX-110402961	T/T	T/C	T/T	Affx-111642378	6B	176544296
AX-110403904	G/G	G/G	G/G	Affx-111794805	6B	440767817
AX-110408267	A/A	A/G	A/A	Affx-109632616	6B	275673228
AX-110408298	G/G	A/G	G/G	Affx-109776137	6B	350443651
AX-110411792	C/C	T/C	C/C	Affx-110696504	6B	158975980
AX-110416865	C/C	T/C	C/C	Affx-111237286	6B	231573850
AX-110417382	T/T	T/T	T/T	Affx-109814810	6B	207666362
AX-110422139	G/G	G/G	G/G	Affx-110793772	6B	687186077
AX-110436320	C/C	T/C	C/C	Affx-110503701	6B	454042671
AX-110437331	C/C	T/C	C/C	Affx-108982202	6B	403906310
AX-110437699	G/G	C/G	G/G	Affx-88522833	6B	519332841
AX-110438609	T/T	T/C	T/T	Affx-109674925	6B	313147444
AX-110442012	C/C	T/C	C/C	Affx-110730774	6B	168778329
AX-110442023	C/C	C/C	C/C	Affx-110955176	6B	148004139
AX-110442184	C/C	C/C	C/C	Affx-111308729	6B	624678098
AX-110442365	T/T	T/C	T/T	Affx-109600725	6B	78568060
AX-110446004	G/G	A/G	G/G	Affx-109349521	6B	466904644
AX-110448396	A/A	A/A	A/A	Affx-88472554	6B	50120743
AX-110458821	G/G	A/G	G/G	Affx-110232469	6B	308671738
AX-110459448	T/T	T/C	T/T	Affx-109377864	6B	601310523
AX-110461069	A/A	A/G	A/A	Affx-109784368	6B	97257521
AX-110461373	C/C	T/C	C/C	Affx-110994745	6B	468691796

AX-110464411	C/C	C/C	C/C	Affx-109515956	6B	529217159
AX-110464915	G/G	C/G	G/G	Affx-109167830	6B	516246694
AX-110468217	A/A	A/G	A/A	Affx-109545040	6B	471304686
AX-110471588	G/G	A/G	G/G	Affx-109860004	6B	487554306
AX-110475129	A/A	A/G	A/A	Affx-88606948	6B	647390093
AX-110479269	A/A	A/C	A/A	Affx-111953090	6B	458015483
AX-110483199	T/T	T/G	T/T	Affx-111661433	6B	33627257
AX-110485498	A/A	A/G	A/A	Affx-108969877	6B	84049419
AX-110487450	C/C	C/C	C/C	Affx-111181912	6B	211565300
AX-110489742	G/G	A/G	G/G	Affx-111236595	6B	650637938
AX-110495169	G/G	A/G	A/A	Affx-109598001	6B	694241181
AX-110496727	G/G	T/G	G/G	Affx-111406416	6B	646424702
AX-110498623	C/C	C/G	G/G	Affx-109235959	6B	696641879
AX-110499186	G/G	A/G	G/G	Affx-110968436	6B	460184426
AX-110500742	T/T	T/C	T/T	Affx-88390250	6B	630949717
AX-110503247	A/A	A/C	A/A	Affx-110724663	6B	201934714
AX-110507188	G/G	G/G	G/G	Affx-110487591	6B	616244120
AX-110510046	G/G	G/G	G/G	Affx-88799682	6B	65093945
AX-110510060	C/C	T/T	C/C	Affx-88479949	6B	555855451
AX-110511632	C/C	C/C	C/C	Affx-110359478	6B	157908920
AX-110511805	A/A	A/G	A/A	Affx-111832833	6B	201454779
AX-110512211	G/G	A/G	G/G	Affx-109666449	6B	439678341
AX-110515622	G/G	A/G	A/A	Affx-111286738	6B	701485823
AX-110516180	A/A	G/G	A/A	Affx-109321463	6B	540107869
AX-110516589	C/C	T/T	C/C	Affx-109879403	6B	256904095
AX-110517181	G/G	A/A	G/G	Affx-109436233	6B	552805127
AX-110518632	A/A	A/G	A/A	Affx-110920827	6B	173020644
AX-110528523	A/A	A/G	A/A	Affx-111486955	6B	210575766
AX-110530219	T/T	T/T	T/T	Affx-109532872	6B	483529467
AX-110530579	G/G	T/G	T/T	Affx-110054172	6B	29770998
AX-110536146	C/C	T/C	C/C	Affx-111388797	6B	184757682
AX-110537016	G/G	A/G	G/G	Affx-110082419	6B	375417126
AX-110538777	C/C	T/C	C/C	Affx-111291877	6B	200952474
AX-110541293	C/C	T/C	C/C	Affx-111758289	6B	587476329
AX-110550919	A/A	A/A	A/A	Affx-109056963	6B	20033634
AX-110561681	A/A	A/A	A/A	Affx-88386784	6B	297767515
AX-110565080	T/T	T/G	T/T	Affx-111486670	6B	449423393
AX-110565871	A/A	A/G	A/A	Affx-110435374	6B	690006676
AX-110566220	C/C	T/T	C/C	Affx-111163555	6B	569374210
AX-110566343	C/C	T/C	C/C	Affx-110836447	6B	486650351
AX-110569296	T/T	T/C	T/T	Affx-110045713	6B	333750365
AX-110569331	G/G	A/G	G/G	Affx-109854696	6B	572188603
AX-110569959	C/C	T/C	C/C	Affx-111531724	6B	276062743
AX-110570192	G/G	A/G	G/G	Affx-110261751	6B	515433897
AX-110570335	T/T	T/C	T/T	Affx-111636341	6B	294913981
AX-110570616	A/A	A/G	A/A	Affx-110487062	6B	158129211
AX-110576978	T/T	T/C	T/T	Affx-109535125	6B	635019801
AX-110577201	C/C	T/C	C/C	Affx-109249986	6B	122094497
AX-110578548	A/A	A/G	A/A	Affx-110345808	6B	232588759
AX-110578609	A/C	A/C	A/C	Affx-110370033	6B	522345443
AX-110587319	G/G	A/A	G/G	Affx-109528117	6B	528138648
AX-110594331	G/G	T/G	G/G	Affx-111570012	6B	193125681
AX-110598414	C/C	T/C	C/C	Affx-111666751	6B	651404340
AX-110602604	C/C	A/C	C/C	Affx-109674811	6B	623436232

AX-110603734	C/C	C/C	C/C	Affx-110691582	6B	700499345
AX-110607060	A/A	G/G	A/A	Affx-108953065	6B	402195528
AX-110607926	A/A	A/T	A/A	Affx-110441375	6B	29357151
AX-110612330	T/T	T/C	T/T	Affx-109668890	6B	437873915
AX-110613536	G/G	A/G	G/G	Affx-109376008	6B	697764055
AX-110624037	C/C	C/C	C/C	Affx-88512664	6B	160660577
AX-110649297	T/T	T/G	T/T	Affx-109378484	6B	618933679
AX-110671748	A/A	A/G	A/A	Affx-109539007	6B	159947324
AX-110671936	T/T	T/C	T/T	Affx-110718097	6B	122675472
AX-110672225	G/G	A/G	G/G	Affx-109065691	6B	40594389
AX-110672473	T/T	T/C	T/T	Affx-109048163	6B	123229315
AX-110673368	T/T	T/C	T/T	Affx-111803841	6B	488284971
AX-110673698	G/G	T/G	G/G	Affx-109817683	6B	688778491
AX-110690284	G/G	A/A	G/G	Affx-110196971	6B	518103056
AX-110690650	C/C	T/C	C/C	Affx-111578283	6B	254889983
AX-110708362	C/C	C/C	C/C	Affx-109887312	6B	403608401
AX-110711667	T/T	T/T	T/T	Affx-109407895	6B	255388879
AX-110712511	T/T	T/T	T/T	Affx-111538309	6B	168476731
AX-110738236	T/T	T/C	T/T	Affx-110359676	6B	344679077
AX-110740055	C/C	A/C	C/C	Affx-110673676	6B	557676051
AX-110913213	G/G	A/G	G/G	Affx-110981942	6B	258013524
AX-110913630	G/G	T/G	G/G	Affx-111686313	6B	599879698
AX-110914628	G/G	C/G	G/G	Affx-110388229	6B	485111321
AX-110919020	T/T	T/C	T/T	Affx-110050636	6B	206845228
AX-110920236	C/C	T/C	C/C	Affx-111438682	6B	248178824
AX-110925419	C/G	G/G	C/G	Affx-111720058	6B	473642101
AX-110937917	C/C	T/C	C/C	Affx-110742032	6B	258574762
AX-110939504	A/A	A/G	G/G	Affx-110446750	6B	14646380
AX-110942372	A/T	A/T	A/T	Affx-109004273	6B	146135281
AX-110944295	T/T	T/C	T/T	Affx-110732859	6B	235922116
AX-110947229	C/C	C/C	C/C	Affx-109285459	6B	125175812
AX-110947926	G/G	A/G	G/G	Affx-111643579	6B	431323263
AX-110952865	A/A	A/G	A/A	Affx-110752685	6B	339999726
AX-110956033	A/A	A/C	A/A	Affx-111438490	6B	142886717
AX-110956189	G/G	A/G	G/G	Affx-109443555	6B	403038121
AX-110959547	G/G	G/G	G/G	Affx-109193793	6B	142223095
AX-110959852	G/G	A/A	G/G	Affx-110416491	6B	435312596
AX-110961066	A/A	A/G	A/A	Affx-110135451	6B	521991659
AX-110970474	A/A	A/G	A/A	Affx-109862184	6B	459662616
AX-110975578	A/A	A/G	A/A	Affx-110010935	6B	203015756
AX-110977373	G/G	C/G	G/G	Affx-109522521	6B	527032383
AX-110979311	T/T	T/C	T/T	Affx-111352905	6B	438494873
AX-110982448	G/G	C/G	G/G	Affx-109467791	6B	557763981
AX-110984386	C/C	T/C	C/C	Affx-110341775	6B	653383521
AX-110986739	G/G	A/A	G/G	Affx-109578802	6B	292125994
AX-110987417	A/A	A/G	A/A	Affx-109670402	6B	144979597
AX-110988400	T/T	T/C	T/T	Affx-111786890	6B	477614174
AX-110989990	T/T	T/C	T/T	Affx-111508346	6B	92056208
AX-110992012	T/T	T/C	T/T	Affx-111762569	6B	21400698
AX-110994491	T/T	T/C	T/T	Affx-109041823	6B	232044932
AX-110998250	G/G	A/G	G/G	Affx-109730505	6B	245984742
AX-111001230	G/G	G/G	G/G	Affx-109730829	6B	136782483
AX-111008709	C/C	T/C	C/C	Affx-111294426	6B	345149761
AX-111009092	T/T	C/C	T/T	Affx-109552115	6B	302154423

AX-111009098	A/A	A/G	A/A	Affx-111892520	6B	339429719
AX-111012187	C/C	T/C	C/C	Affx-110315715	6B	635068388
AX-111017309	G/G	A/G	G/G	Affx-111091883	6B	146899972
AX-111017462	G/G	C/G	G/G	Affx-111184895	6B	544900568
AX-111018214	C/C	T/C	C/C	Affx-110510828	6B	280032238
AX-111019388	C/C	C/C	C/C	Affx-110519213	6B	694675796
AX-111020201	C/C	T/C	C/C	Affx-88568297	6B	613351172
AX-111022908	A/A	A/G	A/A	Affx-111041194	6B	140329704
AX-111027042	C/C	T/C	C/C	Affx-109945186	6B	309588070
AX-111029681	G/G	G/G	G/G	Affx-111553323	6B	530743557
AX-111038641	G/G	A/G	G/G	Affx-109891454	6B	345995044
AX-111038900	G/G	A/G	A/A	Affx-110613465	6B	4569802
AX-111044027	C/C	G/G	C/C	Affx-111190004	6B	662058089
AX-111045757	G/G	A/G	G/G	Affx-110168337	6B	152009602
AX-111047314	T/T	A/T	T/T	Affx-110543625	6B	193686177
AX-111047526	T/T	C/C	T/T	Affx-111635343	6B	573257318
AX-111050096	C/C	C/C	C/C	Affx-111926054	6B	34195446
AX-111054488	T/G	G/G	G/G	Affx-109631224	6B	699592786
AX-111065292	C/C	C/G	C/C	Affx-110981742	6B	190282139
AX-111065717	A/A	A/C	A/A	Affx-109839564	6B	479192243
AX-111065959	T/T	T/C	T/T	Affx-110814428	6B	413610554
AX-111066914	C/C	T/C	C/C	Affx-110598455	6B	531785057
AX-111074264	A/A	A/C	A/A	Affx-109124844	6B	261085138
AX-111076083	G/G	T/G	G/G	Affx-110698117	6B	250340828
AX-111078839	A/A	A/G	A/A	Affx-88740395	6B	496330739
AX-111082654	G/G	T/G	G/G	Affx-110881284	6B	343970317
AX-111087964	A/A	A/G	A/A	Affx-111913599	6B	283968837
AX-111096337	C/C	T/C	C/C	Affx-109207862	6B	454326517
AX-111099951	C/C	C/G	C/C	Affx-111190602	6B	566477687
AX-111100795	A/A	A/G	A/A	Affx-111896607	6B	537893493
AX-111104087	G/G	G/G	G/G	Affx-111182481	6B	191015266
AX-111109046	C/C	T/C	C/C	Affx-108917405	6B	417129941
AX-111109562	C/C	C/C	T/T	Affx-88616497	6B	22244496
AX-111117265	T/T	T/C	T/T	Affx-109142449	6B	430943197
AX-111128068	A/A	A/C	A/A	Affx-110729624	6B	204572280
AX-111132903	A/A	A/G	A/A	Affx-88764544	6B	704945001
AX-111136170	T/T	T/C	T/T	Affx-110284528	6B	178548819
AX-111142565	C/C	G/G	C/C	Affx-111671189	6B	674839814
AX-111142975	G/G	C/G	G/G	Affx-111629244	6B	7304480
AX-111143764	A/A	A/G	A/A	Affx-111717823	6B	565081309
AX-111148313	C/C	T/C	C/C	Affx-109765049	6B	630285889
AX-111157211	T/T	T/T	T/T	Affx-110773320	6B	642465834
AX-111182870	G/G	C/G	G/G	Affx-111110212	6B	549416667
AX-111189818	A/A	A/G	A/A	Affx-110744255	6B	570159601
AX-111192864	A/A	A/T	A/A	Affx-109969856	6B	205263586
AX-111223108	G/G	A/G	G/G	Affx-110381963	6B	632441017
AX-111230620	C/C	C/C	C/C	Affx-109297705	6B	149006455
AX-111235563	A/A	A/G	A/A	Affx-111470629	6B	475251023
AX-111238897	T/T	T/C	T/T	Affx-111440094	6B	442521948
AX-111239605	A/A	A/G	A/A	Affx-111383560	6B	471040183
AX-111453972	C/C	T/C	C/C	Affx-110322449	6B	529697367
AX-111455590	G/G	A/G	A/G	Affx-88495795	6B	720502981
AX-111457109	A/C	A/C	A/C	Affx-108920751	6B	649150521
AX-111462583	T/T	T/C	T/T	Affx-111015060	6B	114708154

AX-111465031	C/C	T/C	C/C	Affx-110719185	6B	654602749
AX-111468538	G/G	A/G	G/G	Affx-109676996	6B	567613174
AX-111473381	A/A	G/G	A/A	Affx-111682122	6B	124635375
AX-111473384	A/A	A/G	A/A	Affx-109725492	6B	568573782
AX-111474462	C/C	C/G	C/C	Affx-108863415	6B	192116173
AX-111476049	C/C	C/C	C/C	Affx-110568559	6B	595293654
AX-111477437	C/C	T/C	C/C	Affx-110607093	6B	470736041
AX-111478169	G/G	A/G	G/G	Affx-111750901	6B	300849582
AX-111480682	G/G	G/G	G/G	Affx-109436065	6B	140751220
AX-111480952	G/G	A/G	G/G	Affx-109560741	6B	126786192
AX-111484692	A/A	A/G	A/A	Affx-88725761	6B	130342427
AX-111486170	A/A	A/C	A/A	Affx-109702939	6B	606426314
AX-111489823	G/G	G/G	G/G	Affx-111886413	6B	303628989
AX-111496655	C/C	C/C	C/C	Affx-110085973	6B	591241100
AX-111497763	A/C	A/C	A/C	Affx-109054337	6B	467455562
AX-111501601	A/A	A/G	A/A	Affx-111782123	6B	358659822
AX-111503079	C/C	C/C	C/C	Affx-88746477	6B	278087618
AX-111503809	A/A	A/G	A/A	Affx-110718007	6B	393411768
AX-111507665	G/G	A/G	G/G	Affx-110696814	6B	133867483
AX-111510231	C/C	T/C	C/C	Affx-109944354	6B	498668110
AX-111513240	T/T	T/G	T/T	Affx-109715143	6B	284470403
AX-111517688	G/G	A/G	G/G	Affx-110422878	6B	608989735
AX-111525440	C/G	C/C	C/G	Affx-111212152	6B	13822769
AX-111528309	A/A	A/G	A/A	Affx-110516889	6B	197705391
AX-111535121	T/T	T/C	T/T	Affx-109835702	6B	385195178
AX-111537240	A/A	A/G	A/A	Affx-88416951	6B	234151643
AX-111540360	G/G	A/G	G/G	Affx-110112956	6B	391459627
AX-111542357	G/G	A/G	G/G	Affx-111547610	6B	131909326
AX-111543278	T/T	T/C	T/T	Affx-110755253	6B	123933013
AX-111546255	T/T	C/C	T/T	Affx-111621211	6B	307835626
AX-111548422	A/A	A/G	A/A	Affx-108933796	6B	495546297
AX-111549094	A/A	A/G	A/A	Affx-109667087	6B	496413643
AX-111551106	C/C	T/C	C/C	Affx-109123078	6B	365290138
AX-111552086	G/G	A/G	G/G	Affx-110940457	6B	81184059
AX-111552346	G/G	G/G	G/G	Affx-109870737	6B	519464257
AX-111555213	A/A	A/G	A/A	Affx-111598966	6B	497525956
AX-111556078	G/G	A/G	G/G	Affx-111446889	6B	184176020
AX-111561208	C/C	T/C	C/C	Affx-109045093	6B	681840987
AX-111564385	T/T	T/C	T/T	Affx-109732439	6B	95442605
AX-111579957	C/C	C/C	C/C	Affx-108897556	6B	620534506
AX-111583351	A/A	A/G	A/A	Affx-108960919	6B	467912645
AX-111584479	C/C	T/C	C/C	Affx-110157133	6B	240390268
AX-111585519	T/T	T/C	T/T	Affx-109482992	6B	613572805
AX-111587247	C/C	C/C	C/C	Affx-110498512	6B	681676003
AX-111592923	G/G	C/C	G/G	Affx-111128026	6B	96325280
AX-111596067	A/A	A/G	A/A	Affx-109044185	6B	498575795
AX-111601308	C/C	C/G	C/C	Affx-111237030	6B	694795643
AX-111604322	G/G	A/G	G/G	Affx-109372458	6B	114521291
AX-111604779	C/C	G/G	C/C	Affx-111159214	6B	340446358
AX-111605648	C/C	C/G	C/C	Affx-110198981	6B	612849771
AX-111609846	A/A	A/T	A/A	Affx-88619989	6B	625775802
AX-111610327	C/C	A/C	C/C	Affx-109920897	6B	654177520
AX-111615114	G/G	A/G	G/G	Affx-109356078	6B	409385657
AX-111618122	C/C	T/C	C/C	Affx-110352986	6B	199447205

AX-111618289	C/C	T/C	C/C	Affx-109332767	6B	458619701
AX-111619260	T/T	T/C	T/C	Affx-111145069	6B	682661324
AX-111623534	A/A	A/T	A/A	Affx-110894987	6B	433157309
AX-111631929	T/T	C/C	T/T	Affx-110303888	6B	298479879
AX-111632479	T/T	T/C	T/T	Affx-109972507	6B	414367057
AX-111637054	A/A	A/C	A/A	Affx-110673522	6B	476774650
AX-111652073	G/G	A/G	G/G	Affx-109047818	6B	610363648
AX-111658296	G/G	C/G	G/G	Affx-109146335	6B	445522455
AX-111663688	A/A	A/G	A/A	Affx-109485682	6B	105447651
AX-111667747	C/C	C/C	C/C	Affx-109362032	6B	415041306
AX-111674729	G/G	T/G	G/G	Affx-110933177	6B	239909074
AX-111676705	A/A	A/G	A/A	Affx-110897117	6B	313429132
AX-111679125	A/A	A/G	A/A	Affx-111876823	6B	260142330
AX-111683398	T/T	T/C	T/T	Affx-110401574	6B	228521957
AX-111683764	C/C	T/C	C/C	Affx-110927945	6B	411361261
AX-111684549	C/C	A/C	C/C	Affx-111711281	6B	390801238
AX-111693296	A/A	A/G	A/A	Affx-110376693	6B	476207853
AX-111705051	T/T	T/C	T/T	Affx-88615433	6B	229519864
AX-111756578	C/C	C/C	C/C	Affx-111328919	6B	277645008
AX-111757493	T/T	T/C	T/T	Affx-88489387	6B	658833342
AX-111760343	C/C	A/C	C/C	Affx-109797380	6B	157322191
AX-111762061	C/C	T/C	C/C	Affx-109535260	6B	192866992
AX-111762280	C/C	C/G	C/C	Affx-108975437	6B	615169515
AX-111762719	A/A	C/C	A/A	Affx-109172210	6B	100926902
AX-111764640	A/A	A/G	A/A	Affx-109921591	6B	164566487
AX-111786324	C/C	T/C	C/C	Affx-110337701	6B	342575703
AX-111796482	G/G	T/G	G/G	Affx-111408663	6B	552133685
AX-111830355	C/C	C/G	C/C	Affx-110541442	6B	446700528
AX-112285928	T/T	T/C	C/C	Affx-88765850	6B	705159044
AX-112287354	T/T	T/T	T/T	Affx-112312530	6B	631951388
AX-112287590	C/C	C/C	C/C	Affx-92820383	6B	702306314
AX-112288144	A/A	A/G	A/A	Affx-92259449	6B	84133464
AX-178060675	T/T	C/C	T/T	Affx-88455644	6B	577479921
AX-179558763	T/T	C/C	T/T	Affx-88767610	6B	614437173
AX-182021105	G/G	T/G	T/G	Affx-110235263	6B	720506650
AX-182033130	G/G	G/G	G/G	Affx-112312332	6B	623729095
AX-182105094	T/C	T/C	T/C	Affx-109948868	6B	720495751
AX-86163175	C/C	T/C	T/C	Affx-93016552	6B	712345618
AX-86165735	T/C	T/T	T/T	Affx-92116085	6B	694168405
AX-86173055	G/G	A/G	G/G	Affx-92994373	6B	577478915
AX-86177832	A/A	A/A	A/A	Affx-92271676	6B	191527596
AX-86178856	G/G	G/G	G/G	Affx-88437883	6B	121489611
AX-86179978	C/C	C/C	C/C	Affx-92613943	6B	646261585
AX-89322751	G/G	A/G	G/G	Affx-88353776	6B	578302251
AX-89337531	G/G	G/G	G/G	Affx-88369033	6B	671927725
AX-89357538	G/G	A/G	G/G	Affx-88389499	6B	645659540
AX-89434290	T/T	T/C	T/T	Affx-110862902	6B	667896487
AX-89526116	C/C	T/C	C/C	Affx-88559046	6B	445007990
AX-89679503	G/G	A/G	G/G	Affx-109480801	6B	659565617
AX-89682312	G/G	C/G	C/C	Affx-88715071	6B	12632699
AX-89696866	G/G	C/G	G/G	Affx-88729611	6B	658582583
AX-94384454	G/G	T/T	G/G	Affx-92646628	6B	115945803
AX-94421041	G/G	G/G	G/G	Affx-92867048	6B	636879322
AX-94428703	T/C	C/C	C/C	Affx-92747060	6B	712674257

AX-94444496	G/G	A/G	G/G	Affx-92720544	6B	676152344
AX-94451943	G/G	A/G	G/G	Affx-92757087	6B	703156580
AX-94473519	T/C	T/T	T/C	Affx-88557244	6B	635172419
AX-94490517	A/A	A/A	A/A	Affx-92654815	6B	540591280
AX-94515056	A/A	A/A	A/A	Affx-88482879	6B	525560062
AX-94534246	G/G	T/G	G/G	Affx-92822268	6B	501522938
AX-94535598	C/C	C/C	C/C	Affx-88501782	6B	591250611
AX-94565231	T/C	T/C	C/C	Affx-92268991	6B	683647294
AX-94569967	C/C	T/T	T/C	Affx-92433516	6B	18855675
AX-94687055	C/C	T/T	T/T	Affx-92807668	6B	41703860
AX-94693808	C/C	C/C	C/C	Affx-92458524	6B	45759915
AX-94697016	T/C	T/T	T/C	Affx-88737161	6B	603358155
AX-94758158	T/C	C/C	T/C	Affx-92716031	6B	492492877
AX-94768091	G/G	A/G	G/G	Affx-92183582	6B	6833977
AX-94795504	G/G	A/G	G/G	Affx-92350200	6B	518391255
AX-94861181	A/A	A/A	A/A	Affx-88533887	6B	639148291
AX-94923203	A/A	A/A	A/A	Affx-92473526	6B	233027277
AX-94925274	T/C	T/C	T/C	Affx-92139242	6B	711758005
AX-95001516	C/C	T/C	C/C	Affx-92318170	6B	710324640
AX-95022626	G/G	C/G	C/G	Affx-92310391	6B	716258228
AX-95091214	C/C	C/C	C/C	Affx-92485126	6B	673765863
AX-95110233	G/G	C/G	G/G	Affx-92473279	6B	563055841
AX-95121775	A/A	A/A	A/A	Affx-88616980	6B	648088927
AX-95137200	C/C	T/C	C/C	Affx-92149237	6B	47860821
AX-95142104	T/C	T/T	T/C	Affx-92293046	6B	720497995
AX-95148867	T/T	T/T	T/T	Affx-92899209	6B	574662638
AX-95168433	T/T	T/T	T/T	Affx-92784482	6B	30277755
AX-95172400	C/C	C/C	C/C	Affx-92700457	6B	720497040
AX-95173501	T/T	T/C	C/C	Affx-92423842	6B	696468301
AX-95629678	A/G	G/G	G/G	Affx-88345922	6B	701387320
AX-95632153	G/G	A/G	G/G	Affx-88372530	6B	705285919
AX-95633821	C/C	T/C	C/C	Affx-112313102	6B	481862135
AX-95634492	A/A	G/G	A/A	Affx-92998265	6B	645353741
AX-95652825	G/G	G/G	G/G	Affx-88466468	6B	150665070
AX-95658009	G/G	A/G	G/G	Affx-88475885	6B	353872242
AX-95658903	A/C	C/C	A/C	Affx-88570649	6B	562598032
AX-95659492	T/T	C/C	T/T	Affx-88675854	6B	699006494
AX-95679065	G/G	A/G	G/G	Affx-92987083	6B	582824969
AX-108730358	A/G	A/G	G/G	Affx-109204291	6D	133466400
AX-108742476	A/C	A/C	A/C	Affx-110725215	6D	171134758
AX-108751452	T/T	T/T	T/C	Affx-111717809	6D	391892191
AX-108762606	G/G	G/G	G/G	Affx-111996260	6D	115052506
AX-108777047	A/C	A/C	C/C	Affx-109301191	6D	154397950
AX-108782933	T/C	T/C	T/T	Affx-88582531	6D	241408183
AX-108792360	A/A	A/C	A/A	Affx-109564251	6D	456398407
AX-108793636	G/G	G/G	G/G	Affx-110027852	6D	377831510
AX-108796868	A/A	A/C	A/A	Affx-110780763	6D	451938160
AX-108803341	A/G	A/G	G/G	Affx-110335922	6D	177345635
AX-108808425	A/A	A/G	A/A	Affx-109021277	6D	297401504
AX-108832017	A/A	A/G	A/A	Affx-109032932	6D	414466187
AX-108832623	T/C	T/C	T/C	Affx-109110791	6D	457352148
AX-108835095	T/C	T/C	T/T	Affx-88807616	6D	201518092
AX-108838575	A/G	A/G	A/G	Affx-111813724	6D	11662117
AX-108846105	A/G	A/G	G/G	Affx-111358316	6D	260154574

AX-108849732	G/G	A/G	G/G	Affx-111604901	6D	403652851
AX-108855910	T/C	T/C	C/C	Affx-109068687	6D	198139176
AX-108856383	T/G	T/G	T/T	Affx-111369192	6D	162550184
AX-108857636	C/C	C/G	G/G	Affx-88393462	6D	421550783
AX-108865480	T/T	T/C	T/T	Affx-109709099	6D	266778002
AX-108871977	C/C	T/C	T/T	Affx-110859864	6D	294634977
AX-108882824	G/G	A/G	A/A	Affx-88795187	6D	315742417
AX-108883119	G/G	T/T	G/G	Affx-111473676	6D	9872635
AX-108896531	C/C	C/C	C/C	Affx-111418418	6D	450698816
AX-108897553	T/C	T/C	C/C	Affx-111334057	6D	470390265
AX-108908222	A/C	A/C	A/A	Affx-110588689	6D	191467414
AX-108931445	C/C	A/A	C/C	Affx-111366737	6D	7624866
AX-108934077	A/G	A/G	G/G	Affx-111355445	6D	256936717
AX-108942504	A/G	A/G	A/A	Affx-109993136	6D	191421328
AX-108947983	A/C	A/A	C/C	Affx-111155063	6D	234290771
AX-108957529	G/G	C/G	G/G	Affx-111680547	6D	420742218
AX-108963827	G/G	A/G	G/G	Affx-88730122	6D	63463118
AX-108966812	A/A	A/G	A/A	Affx-110559819	6D	449994620
AX-108971890	T/T	T/C	T/T	Affx-110556371	6D	17363669
AX-109007839	C/C	C/C	C/C	Affx-110922772	6D	381343653
AX-109040618	T/C	T/C	C/C	Affx-109861102	6D	44300645
AX-109058428	C/C	T/C	C/C	Affx-109731684	6D	454100974
AX-109075708	T/C	T/T	T/T	Affx-109675312	6D	175242915
AX-109077353	G/G	A/A	G/G	Affx-109471531	6D	454019010
AX-109081975	A/G	A/G	A/A	Affx-111243816	6D	238535146
AX-109125695	T/C	T/C	C/C	Affx-110850129	6D	196579975
AX-109169405	A/G	A/G	A/A	Affx-109690645	6D	259610755
AX-109189090	C/C	T/C	C/C	Affx-111420641	6D	270078537
AX-109193807	G/G	T/G	T/G	Affx-109779643	6D	14044286
AX-109200636	A/A	A/T	A/A	Affx-110861549	6D	436921286
AX-109202589	G/G	T/G	G/G	Affx-109207138	6D	392028666
AX-109206187	C/C	T/C	C/C	Affx-110090824	6D	264185632
AX-109206549	T/C	T/C	T/T	Affx-88417917	6D	157811389
AX-109234601	A/C	A/C	C/C	Affx-109519499	6D	218805589
AX-109278213	C/C	T/C	C/C	Affx-88558462	6D	271306246
AX-109280501	A/A	A/C	C/C	Affx-109310805	6D	4721055
AX-109288163	T/T	T/C	T/T	Affx-111146953	6D	465173231
AX-109295258	A/G	A/A	A/A	Affx-108928401	6D	184507155
AX-109317417	A/C	A/C	A/C	Affx-110061562	6D	44498347
AX-109329400	G/G	A/G	G/G	Affx-111774600	6D	9978681
AX-109331772	T/C	T/C	C/C	Affx-88550889	6D	156759738
AX-109332621	G/G	A/G	A/A	Affx-110399537	6D	51494
AX-109341373	T/C	T/C	T/T	Affx-111045032	6D	223186793
AX-109343984	T/C	T/C	T/T	Affx-110455125	6D	91808956
AX-109355362	G/G	G/G	A/A	Affx-110061553	6D	351057618
AX-109361680	A/G	A/G	A/A	Affx-109013114	6D	231587590
AX-109391798	G/G	A/G	G/G	Affx-88352700	6D	445716104
AX-109392860	T/T	G/G	T/T	Affx-88791303	6D	361173041
AX-109397314	A/G	A/G	G/G	Affx-88437915	6D	90039802
AX-109397401	C/G	C/G	G/G	Affx-111757987	6D	803360
AX-109398984	T/C	T/C	C/C	Affx-110503480	6D	236285977
AX-109400723	G/G	A/G	A/A	Affx-111581162	6D	82718391
AX-109412721	A/G	A/G	G/G	Affx-109865825	6D	60114333
AX-109416883	A/A	A/C	A/A	Affx-111839226	6D	19648311

AX-109420819	C/G	C/G	C/C	Affx-88643286	6D	110760142
AX-109429350	C/C	C/G	G/G	Affx-88808431	6D	473518997
AX-109432523	C/G	C/C	C/C	Affx-88381239	6D	202973760
AX-109435951	A/A	A/G	G/G	Affx-88652505	6D	469993277
AX-109436607	T/C	T/C	C/C	Affx-110717494	6D	232767029
AX-109437972	T/G	T/G	G/G	Affx-109188099	6D	247196064
AX-109440539	T/T	T/C	T/T	Affx-111354487	6D	307087147
AX-109441126	C/C	C/G	C/C	Affx-111947520	6D	2172318
AX-109444036	C/G	C/G	G/G	Affx-109880926	6D	257149736
AX-109447460	A/G	A/A	G/G	Affx-111036700	6D	89262091
AX-109465489	G/G	T/T	G/G	Affx-111206270	6D	418197326
AX-109476856	C/C	T/C	T/T	Affx-111981121	6D	5318929
AX-109491420	T/T	T/C	T/T	Affx-109123858	6D	289517069
AX-109509128	G/G	C/G	G/G	Affx-110616872	6D	402608122
AX-109521374	G/G	A/G	G/G	Affx-111491244	6D	276415630
AX-109532919	C/C	T/C	C/C	Affx-108868617	6D	429039352
AX-109534413	G/G	A/G	G/G	Affx-110006428	6D	291244822
AX-109569276	T/T	T/G	T/T	Affx-110982584	6D	7567488
AX-109579274	G/G	A/G	G/G	Affx-111557508	6D	3184515
AX-109600542	A/G	A/G	A/G	Affx-111610775	6D	210118483
AX-109634053	A/A	A/A	A/A	Affx-109339606	6D	454828920
AX-109637291	C/C	C/G	C/C	Affx-111988986	6D	455805543
AX-109679460	C/G	C/G	G/G	Affx-111501239	6D	129043100
AX-109699919	G/G	T/G	G/G	Affx-109099578	6D	363410800
AX-109702819	T/C	C/C	C/C	Affx-88400653	6D	144065215
AX-109705668	T/T	T/T	T/T	Affx-111731944	6D	284074318
AX-109716798	G/G	A/G	G/G	Affx-110602770	6D	143583296
AX-109724223	A/G	A/G	A/A	Affx-109962259	6D	255095185
AX-109733276	A/G	G/G	G/G	Affx-111533582	6D	135116195
AX-109734121	T/C	C/C	C/C	Affx-109132078	6D	212997502
AX-109742573	C/C	T/C	C/C	Affx-110758146	6D	312330704
AX-109747732	T/C	T/C	T/T	Affx-109782464	6D	194853453
AX-109752826	G/G	A/G	G/G	Affx-110811035	6D	333490747
AX-109755494	T/T	T/C	T/T	Affx-110670664	6D	339286118
AX-109778600	A/G	A/G	A/G	Affx-110504867	6D	200381162
AX-109817610	A/G	A/G	G/G	Affx-111563577	6D	122691995
AX-109820077	T/T	T/C	T/T	Affx-109430126	6D	367904575
AX-109826795	G/G	A/G	G/G	Affx-111695402	6D	340461520
AX-109836360	G/G	A/G	G/G	Affx-109114586	6D	432925403
AX-109840078	G/G	C/G	G/G	Affx-109090012	6D	435946745
AX-109843427	G/G	A/G	A/A	Affx-111913343	6D	313877363
AX-109844231	T/T	T/G	T/T	Affx-110343092	6D	387110980
AX-109848275	C/C	C/C	C/C	Affx-111436944	6D	47255641
AX-109850108	A/C	A/C	C/C	Affx-111742056	6D	192624602
AX-109852543	G/G	A/A	G/G	Affx-111649052	6D	46498487
AX-109857388	T/C	T/C	C/C	Affx-110167788	6D	153205651
AX-109875433	T/T	T/G	T/T	Affx-111520920	6D	460859368
AX-109896910	C/C	C/C	C/C	Affx-110537510	6D	273914342
AX-109897923	C/C	T/C	C/C	Affx-109581648	6D	7291943
AX-109898786	G/G	A/G	G/G	Affx-109913733	6D	438653940
AX-109902223	C/G	C/G	G/G	Affx-88781889	6D	230525358
AX-109902270	T/C	T/C	T/T	Affx-111178345	6D	93853873
AX-109915068	C/C	T/C	C/C	Affx-109567499	6D	46871301
AX-109921915	T/C	T/C	C/C	Affx-88389221	6D	139823814

AX-109940039	A/C	A/C	A/C	Affx-109769265	6D	20554990
AX-109963640	T/C	T/C	T/T	Affx-111136756	6D	240772554
AX-109967269	T/C	T/C	T/C	Affx-109472887	6D	22183383
AX-109972620	A/G	A/G	A/A	Affx-111282549	6D	116347613
AX-109975102	A/A	A/G	A/A	Affx-88523518	6D	462601646
AX-109987618	A/A	A/G	A/G	Affx-111559193	6D	465691496
AX-110012620	C/C	T/C	C/C	Affx-110594437	6D	56909317
AX-110016680	A/A	A/C	C/C	Affx-88565391	6D	2669735
AX-110023402	C/C	A/C	C/C	Affx-111075085	6D	350066067
AX-110023483	G/G	T/G	G/G	Affx-111751114	6D	417554868
AX-110040257	G/G	C/G	G/G	Affx-109710482	6D	359358649
AX-110048049	T/T	G/G	T/G	Affx-111419590	6D	464663757
AX-110059925	A/A	A/G	A/A	Affx-109776991	6D	12326997
AX-110068547	C/C	T/C	T/T	Affx-111394443	6D	6315087
AX-110076571	A/A	A/C	A/A	Affx-111531157	6D	15698282
AX-110079598	T/T	T/C	T/T	Affx-109869168	6D	267521115
AX-110110950	T/T	C/C	C/C	Affx-110939739	6D	413272957
AX-110122439	A/A	A/G	G/G	Affx-88384434	6D	52043044
AX-110129788	A/G	A/G	G/G	Affx-88604245	6D	141531581
AX-110163413	G/G	A/G	G/G	Affx-109999576	6D	330821153
AX-110166827	T/T	T/C	T/T	Affx-110210145	6D	456645974
AX-110191700	A/G	A/G	A/A	Affx-109967295	6D	178529472
AX-110208453	C/C	G/G	C/C	Affx-111860126	6D	442226684
AX-110223323	A/A	A/G	A/A	Affx-110020773	6D	270763123
AX-110223575	T/C	T/T	T/C	Affx-109729785	6D	202316296
AX-110234676	T/C	T/C	C/C	Affx-111534545	6D	247259246
AX-110276770	G/G	A/G	G/G	Affx-110990217	6D	70212935
AX-110278595	A/G	A/G	A/G	Affx-88646188	6D	261614542
AX-110283645	T/C	T/C	T/T	Affx-88738782	6D	95440094
AX-110291378	A/A	A/G	G/G	Affx-111965320	6D	6833518
AX-110334567	C/G	C/G	C/C	Affx-110387648	6D	108731707
AX-110351486	T/T	T/C	T/T	Affx-111446807	6D	318231175
AX-110351501	A/A	A/G	A/A	Affx-108963383	6D	326593639
AX-110364740	T/C	T/C	T/T	Affx-88613455	6D	61004778
AX-110382511	T/T	T/C	T/T	Affx-109155369	6D	24026993
AX-110392457	A/G	A/G	A/A	Affx-111826859	6D	54005362
AX-110392637	G/G	A/G	G/G	Affx-110095398	6D	439800128
AX-110399963	A/G	A/A	A/G	Affx-110108590	6D	11807767
AX-110409139	A/A	A/G	A/A	Affx-109542649	6D	421889524
AX-110420143	T/T	T/G	G/G	Affx-111121629	6D	918288
AX-110423754	C/C	C/G	G/G	Affx-111225074	6D	322413086
AX-110425031	G/G	T/G	G/G	Affx-111472746	6D	381556904
AX-110431664	T/T	T/C	T/T	Affx-88696026	6D	357008385
AX-110435301	C/C	T/C	C/C	Affx-110226393	6D	449245754
AX-110452797	C/C	T/C	T/T	Affx-109822625	6D	430458554
AX-110483627	C/C	C/C	C/C	Affx-109478420	6D	42540858
AX-110493066	T/C	T/C	T/T	Affx-111640985	6D	162205401
AX-110507974	T/C	T/C	C/C	Affx-88492357	6D	185184180
AX-110509864	T/C	T/C	C/C	Affx-88715921	6D	109400790
AX-110521176	T/C	T/C	T/T	Affx-109709389	6D	196131574
AX-110525772	T/C	T/C	T/T	Affx-111361654	6D	212477708
AX-110532674	T/C	T/C	T/C	Affx-111827607	6D	232781080
AX-110561318	G/G	C/G	C/C	Affx-109632767	6D	430749349
AX-110561381	A/G	A/G	A/A	Affx-109561166	6D	183756308

AX-110570990	A/G	A/G	A/A	Affx-88363560	6D	94277759
AX-110571231	A/A	A/C	A/A	Affx-110783950	6D	419269593
AX-110575536	C/C	T/C	C/C	Affx-88699041	6D	380176310
AX-110582974	A/G	A/G	G/G	Affx-110944900	6D	59765241
AX-110590440	T/T	T/C	T/T	Affx-109353718	6D	326975395
AX-110591266	C/C	T/C	C/C	Affx-108972025	6D	360382104
AX-110599765	T/T	T/C	T/T	Affx-110583822	6D	294022095
AX-110612307	T/T	T/C	T/T	Affx-109221360	6D	402872625
AX-110617407	C/C	T/C	C/C	Affx-111098661	6D	291151697
AX-110642302	C/C	T/C	C/C	Affx-110456295	6D	346824446
AX-110667557	G/G	A/G	G/G	Affx-109458508	6D	24552431
AX-110667811	T/C	T/T	C/C	Affx-109466649	6D	182335550
AX-110670670	T/C	T/C	C/C	Affx-88615027	6D	123973259
AX-110685828	T/C	T/C	T/T	Affx-108888708	6D	255183924
AX-110687766	G/G	A/G	G/G	Affx-109812120	6D	410143937
AX-110689162	T/C	T/C	C/C	Affx-111312659	6D	147602946
AX-110711001	T/T	A/T	T/T	Affx-111066795	6D	345784593
AX-110716651	T/G	T/G	T/T	Affx-88743197	6D	141316324
AX-110763870	G/G	C/G	G/G	Affx-109747315	6D	239279634
AX-110785030	T/C	T/C	C/C	Affx-88474146	6D	126000097
AX-110787660	A/G	A/G	G/G	Affx-109595940	6D	127010065
AX-110830886	T/C	T/C	T/T	Affx-111942085	6D	106289433
AX-110831860	G/G	A/G	G/G	Affx-109293473	6D	405487312
AX-110837475	C/C	C/C	C/C	Affx-110089938	6D	144566597
AX-110871088	C/C	T/C	C/C	Affx-111911038	6D	375214100
AX-110917876	A/T	A/T	A/A	Affx-88788149	6D	206652606
AX-110924734	G/G	A/G	G/G	Affx-109333218	6D	373410046
AX-110936223	T/T	T/C	C/C	Affx-109939787	6D	3724650
AX-110943840	C/C	C/C	C/C	Affx-111755936	6D	437620622
AX-110953725	T/T	T/T	T/C	Affx-88361499	6D	87112369
AX-110956800	G/G	G/G	G/G	Affx-109541925	6D	435352936
AX-110973137	A/C	A/C	A/A	Affx-111194371	6D	158986892
AX-110976102	C/C	T/C	C/C	Affx-110513197	6D	446902564
AX-110981380	A/A	A/C	A/A	Affx-111532406	6D	460474953
AX-110984956	A/G	A/G	A/A	Affx-88796808	6D	110213131
AX-110999784	C/C	T/C	T/C	Affx-111525209	6D	16852681
AX-111019082	T/C	T/C	T/T	Affx-88372413	6D	155604027
AX-111028479	C/C	T/C	C/C	Affx-109401516	6D	50748612
AX-111028967	C/C	T/T	C/C	Affx-111918032	6D	29467167
AX-111037236	A/A	A/T	A/A	Affx-111232562	6D	371726368
AX-111052761	A/G	A/G	A/A	Affx-88720998	6D	93680935
AX-111058463	T/C	T/C	C/C	Affx-88391799	6D	159597462
AX-111078258	C/C	T/C	C/C	Affx-110124041	6D	448642466
AX-111089565	T/G	T/G	T/T	Affx-110870992	6D	217712796
AX-111097759	T/C	T/C	T/T	Affx-109372842	6D	176121225
AX-111104259	C/C	T/C	C/C	Affx-111654443	6D	303974853
AX-111121905	C/C	C/G	C/C	Affx-110969904	6D	441395809
AX-111131299	C/C	A/A	C/C	Affx-109181806	6D	7721640
AX-111135144	T/T	A/T	T/T	Affx-88801037	6D	463124552
AX-111149667	C/C	C/C	T/T	Affx-88351283	6D	59103513
AX-111150279	A/A	A/G	A/A	Affx-111551527	6D	451097768
AX-111153316	A/G	A/G	G/G	Affx-110636015	6D	225438207
AX-111157636	T/T	T/C	T/T	Affx-88411984	6D	370683123
AX-111178241	C/C	A/C	C/C	Affx-111351951	6D	7645075

AX-111285003	C/C	A/C	C/C	Affx-111906690	6D	335923113
AX-111287721	C/C	T/C	C/C	Affx-110860122	6D	101660116
AX-111295961	A/G	A/G	A/A	Affx-111090581	6D	103428404
AX-111330680	A/A	A/A	A/G	Affx-111808116	6D	156716180
AX-111359056	T/G	T/G	T/T	Affx-88431451	6D	201536241
AX-111360208	T/C	T/C	C/C	Affx-111568842	6D	218812119
AX-111361676	T/C	T/C	C/C	Affx-111787281	6D	104445297
AX-111368177	C/C	C/G	C/C	Affx-109567055	6D	296673517
AX-111369176	A/G	A/G	G/G	Affx-110519793	6D	207122143
AX-111369206	G/G	C/G	G/G	Affx-111079937	6D	458140740
AX-111370974	A/G	A/G	G/G	Affx-108968943	6D	137893383
AX-111378941	T/C	T/C	C/C	Affx-109980623	6D	200380054
AX-111385013	T/C	T/C	T/T	Affx-110727936	6D	182576587
AX-111385363	A/G	A/A	A/A	Affx-111793754	6D	261072260
AX-111385408	A/C	A/C	C/C	Affx-109995045	6D	225307677
AX-111386864	T/C	T/C	T/T	Affx-110922486	6D	237910770
AX-111387909	A/A	A/G	A/A	Affx-108906302	6D	373553718
AX-111406534	T/C	T/C	T/T	Affx-108899762	6D	166393322
AX-111419172	C/C	A/C	C/C	Affx-110259348	6D	444542241
AX-111455327	A/A	A/G	A/A	Affx-110893035	6D	442297503
AX-111455933	C/C	T/C	C/C	Affx-109740779	6D	277098313
AX-111463721	G/G	A/G	G/G	Affx-109623078	6D	433401548
AX-111465387	C/C	T/C	C/C	Affx-111209967	6D	29740810
AX-111468871	A/G	A/G	A/A	Affx-111638277	6D	223684320
AX-111470318	T/C	T/C	T/T	Affx-111103348	6D	222777175
AX-111480830	A/T	A/T	T/T	Affx-109111498	6D	78861580
AX-111484876	A/G	A/G	G/G	Affx-109733197	6D	163143345
AX-111492246	C/C	T/C	C/C	Affx-109026578	6D	413016928
AX-111498097	C/C	C/G	C/C	Affx-109579594	6D	45835710
AX-111501437	G/G	A/G	G/G	Affx-110880835	6D	434707559
AX-111505537	A/A	A/G	A/A	Affx-111669450	6D	23109492
AX-111511037	G/G	G/G	C/G	Affx-88696408	6D	468825726
AX-111517298	A/G	A/G	G/G	Affx-111915370	6D	432472118
AX-111519227	A/C	A/C	C/C	Affx-88780311	6D	117249452
AX-111521512	A/G	A/G	A/A	Affx-88364546	6D	179911424
AX-111527596	G/G	G/G	G/G	Affx-88554776	6D	472487225
AX-111530024	A/A	A/G	A/A	Affx-111728951	6D	306891367
AX-111532602	C/C	C/G	G/G	Affx-111998620	6D	1628930
AX-111538738	T/T	T/G	G/G	Affx-109387557	6D	396007971
AX-111551908	C/C	T/C	C/C	Affx-110843482	6D	305271752
AX-111567408	T/G	T/G	T/T	Affx-111132832	6D	207326362
AX-111571048	G/G	A/G	G/G	Affx-109859873	6D	48473093
AX-111579048	A/C	A/C	C/C	Affx-111862033	6D	209402624
AX-111581587	G/G	A/A	G/G	Affx-109736929	6D	445764997
AX-111583179	A/G	A/G	G/G	Affx-88513996	6D	242674808
AX-111609000	T/C	T/T	C/C	Affx-88386501	6D	124638265
AX-111615506	G/G	G/G	G/G	Affx-111713098	6D	429674465
AX-111615617	G/G	A/G	G/G	Affx-109517057	6D	379560423
AX-111645948	T/C	T/C	T/T	Affx-110079005	6D	5824441
AX-111657836	A/G	A/G	A/A	Affx-88395758	6D	194357819
AX-111671047	C/G	C/G	G/G	Affx-109328305	6D	205361872
AX-111675578	A/A	A/A	A/A	Affx-109785084	6D	464182885
AX-111691409	G/G	A/G	G/G	Affx-111983693	6D	361531417
AX-111692691	A/G	A/G	A/A	Affx-108952824	6D	210375321

AX-111696257	T/T	T/T	T/C	Affx-108971714	6D	431111581
AX-111696856	A/G	A/A	A/A	Affx-110337019	6D	230048043
AX-111700296	T/C	T/C	C/C	Affx-109771775	6D	107043704
AX-111716941	A/A	A/G	A/A	Affx-111746295	6D	377828091
AX-111768962	A/A	A/G	G/G	Affx-108992195	6D	470939128
AX-111780375	C/C	T/C	C/C	Affx-111456981	6D	264716195
AX-111803003	A/G	A/G	G/G	Affx-88343276	6D	204788554
AX-111803933	T/C	T/C	T/T	Affx-111099779	6D	189474435
AX-111844909	A/G	A/G	G/G	Affx-111148213	6D	234136544
AX-111861046	C/C	A/A	C/C	Affx-88659237	6D	444497482
AX-111878449	A/G	G/G	G/G	Affx-110873460	6D	236957054
AX-111885402	A/G	A/G	G/G	Affx-109803950	6D	231399388
AX-111913432	A/G	A/G	A/A	Affx-109062690	6D	221481148
AX-111917346	G/G	A/G	A/A	Affx-110545411	6D	301654754
AX-111919223	G/G	A/G	G/G	Affx-111959701	6D	278608385
AX-111923535	A/G	A/G	G/G	Affx-110652823	6D	117984429
AX-111926352	T/T	T/C	T/T	Affx-111255815	6D	408892368
AX-111931231	A/A	A/A	A/A	Affx-110719447	6D	329902322
AX-182016608	G/G	T/G	G/G	Affx-109591953	6D	7721585
AX-182069980	A/A	A/A	A/A	Affx-472319748	6D	107802746
AX-86174438	G/G	G/G	G/G	Affx-112314793	6D	311584676
AX-89372296	C/C	T/C	C/C	Affx-88404524	6D	3821292
AX-89508120	A/A	G/G	A/A	Affx-88540999	6D	329851607
AX-89578032	C/G	G/G	C/G	Affx-88611116	6D	149023768
AX-89640677	T/C	T/C	C/C	Affx-88673468	6D	151832215
AX-89643022	T/T	T/C	T/T	Affx-88675818	6D	67602805
AX-94504048	T/T	T/C	T/T	Affx-92807386	6D	33143556
AX-94618881	G/G	A/G	G/G	Affx-92393591	6D	45905035
AX-94790334	T/C	C/C	C/C	Affx-92794325	6D	462014080
AX-94826656	G/G	T/T	G/G	Affx-92678028	6D	459240264
AX-94875830	T/C	C/C	T/T	Affx-92964297	6D	452632659
AX-94912958	T/C	C/C	T/C	Affx-92282017	6D	18008318
AX-94978974	C/G	C/C	C/G	Affx-92695100	6D	29876083
AX-94994097	G/G	A/G	G/G	Affx-92682808	6D	448960177
AX-95120751	G/G	A/G	A/A	Affx-92224525	6D	466268739
AX-95127885	A/A	G/G	A/A	Affx-92903191	6D	157450680
AX-95154560	T/C	C/C	T/C	Affx-92912864	6D	55633875
AX-95163029	T/G	T/T	T/G	Affx-92830272	6D	390479790
AX-95175830	G/G	A/G	G/G	Affx-92909851	6D	8969404
AX-95230097	A/A	A/A	A/A	Affx-92516282	6D	21981934
AX-95633708	A/A	A/A	A/A	Affx-92580927	6D	462198638
AX-95657989	T/T	T/T	T/T	Affx-88687982	6D	2269185
AX-95658492	G/G	A/G	G/G	Affx-88365777	6D	389607042
AX-108727788	T/T	T/C	T/T	Affx-109667965	7A	263736512
AX-108733862	T/T	G/G	T/T	Affx-111700442	7A	508609040
AX-108734538	A/A	A/G	A/A	Affx-111212539	7A	272572340
AX-108736273	G/G	C/G	C/C	Affx-109886804	7A	627227763
AX-108736604	G/G	G/G	A/A	Affx-110013039	7A	655403231
AX-108737260	G/G	A/G	G/G	Affx-88350471	7A	48527112
AX-108737754	A/A	A/G	A/A	Affx-111354278	7A	535320141
AX-108737856	G/G	T/G	G/G	Affx-108857328	7A	721372763
AX-108742004	T/C	T/C	T/T	Affx-109181098	7A	20181252
AX-108745280	C/C	C/G	C/C	Affx-109499221	7A	83302634
AX-108747809	G/G	C/C	G/G	Affx-110586492	7A	18520295

AX-108748484	A/A	A/G	A/A	Affx-110879035	7A	730508297
AX-108750395	T/T	T/C	T/T	Affx-109119158	7A	514763496
AX-108750483	A/A	A/C	A/A	Affx-110013662	7A	498888425
AX-108751637	C/C	T/C	C/C	Affx-111483010	7A	661276347
AX-108758009	T/T	T/C	T/T	Affx-110455167	7A	165835321
AX-108759584	A/A	A/C	C/C	Affx-110287738	7A	642323037
AX-108760065	T/T	T/C	T/T	Affx-109515593	7A	708433195
AX-108762304	C/C	T/C	C/C	Affx-111238806	7A	170688269
AX-108763717	C/C	T/C	T/T	Affx-111022951	7A	643299390
AX-108764552	C/C	T/C	C/C	Affx-110564232	7A	91804065
AX-108769200	A/A	A/C	A/A	Affx-88412590	7A	736496356
AX-108771714	T/T	T/C	T/T	Affx-110581765	7A	89848789
AX-108773612	C/C	T/C	T/T	Affx-109226934	7A	694717237
AX-108774942	G/G	A/G	G/G	Affx-109661428	7A	706169060
AX-108775613	G/G	A/G	A/A	Affx-109181445	7A	709964480
AX-108783946	G/G	C/G	G/G	Affx-111521218	7A	172239797
AX-108786148	C/C	T/C	C/C	Affx-110386342	7A	202991984
AX-108786268	C/C	A/C	C/C	Affx-109256260	7A	685709184
AX-108790823	A/A	A/G	A/A	Affx-109231654	7A	6852308
AX-108792245	G/G	A/A	G/G	Affx-88604089	7A	30888727
AX-108792924	C/C	C/G	C/C	Affx-110104174	7A	729407468
AX-108793437	A/A	A/G	A/A	Affx-109890342	7A	143748531
AX-108794218	T/T	T/C	T/T	Affx-110814985	7A	17334236
AX-108794609	G/G	A/G	G/G	Affx-111444857	7A	691124730
AX-108796729	A/A	A/A	A/A	Affx-109562387	7A	720996426
AX-108797320	G/G	C/C	C/C	Affx-111959052	7A	683102521
AX-108797862	C/C	T/C	C/C	Affx-109465723	7A	121900736
AX-108798436	A/G	G/G	A/G	Affx-111853781	7A	486151778
AX-108807321	C/C	A/C	C/C	Affx-111152754	7A	691640086
AX-108808481	C/C	T/C	C/C	Affx-88558504	7A	218244507
AX-108809822	G/G	G/G	G/G	Affx-109176341	7A	200908974
AX-108815521	C/C	C/G	C/C	Affx-108965296	7A	50264659
AX-108815544	G/G	G/G	G/G	Affx-111788115	7A	577769168
AX-108820243	A/A	A/G	A/A	Affx-110145060	7A	554397580
AX-108820762	G/G	G/G	G/G	Affx-111532749	7A	68319324
AX-108822116	G/G	A/G	G/G	Affx-110026064	7A	92222040
AX-108823291	C/G	C/G	C/C	Affx-109442956	7A	608989092
AX-108824792	G/G	C/C	G/G	Affx-109266615	7A	83907674
AX-108825377	A/A	G/G	A/A	Affx-88670450	7A	64612571
AX-108839052	T/T	T/C	T/T	Affx-110275029	7A	165189216
AX-108842149	T/T	T/C	T/T	Affx-110545847	7A	593455658
AX-108851516	G/G	A/G	G/G	Affx-88502194	7A	172915273
AX-108851720	C/C	T/C	C/C	Affx-111197625	7A	198264793
AX-108856132	A/G	G/G	A/G	Affx-110749849	7A	163376302
AX-108856877	A/A	A/G	A/A	Affx-111835471	7A	594424921
AX-108860527	A/A	A/G	A/A	Affx-110807386	7A	729562890
AX-108863658	A/A	A/C	A/A	Affx-111700310	7A	260314152
AX-108866878	T/T	T/C	C/C	Affx-108944848	7A	619879562
AX-108873906	T/T	C/C	T/T	Affx-111728541	7A	91493943
AX-108874556	A/A	A/C	A/A	Affx-109188993	7A	53732551
AX-108875867	G/G	A/G	G/G	Affx-110716166	7A	524698892
AX-108881025	C/C	A/C	C/C	Affx-111817318	7A	237381233
AX-108881635	C/C	T/C	C/C	Affx-110274570	7A	533967237
AX-108882336	C/C	T/C	C/C	Affx-110697646	7A	494993433

AX-108883385	A/A	A/G	A/A	Affx-110459044	7A	274389607
AX-108890211	C/C	T/C	C/C	Affx-110262722	7A	158598016
AX-108890749	C/C	T/C	C/C	Affx-111504188	7A	671218701
AX-108895584	G/G	A/G	G/G	Affx-108887626	7A	576226346
AX-108896295	A/A	A/C	A/A	Affx-109423030	7A	732800049
AX-108903353	T/T	T/G	T/T	Affx-109528110	7A	515492874
AX-108905688	G/G	G/G	G/G	Affx-109337502	7A	167827577
AX-108907186	G/G	A/A	G/G	Affx-109607684	7A	35893073
AX-108910307	G/G	A/G	G/G	Affx-88767770	7A	518711979
AX-108910750	T/T	T/C	T/T	Affx-110213863	7A	544332789
AX-108918469	A/A	A/G	A/A	Affx-110773078	7A	593651189
AX-108921650	G/G	A/G	G/G	Affx-111855712	7A	410295448
AX-108926816	C/C	T/T	C/C	Affx-109732051	7A	162726566
AX-108928821	A/G	A/G	A/A	Affx-111665454	7A	1507323
AX-108930220	C/C	T/C	C/C	Affx-109288583	7A	500690931
AX-108934178	A/A	A/G	A/A	Affx-111485363	7A	369005541
AX-108936497	T/T	T/C	T/T	Affx-111650431	7A	503453184
AX-108938437	A/A	A/G	A/A	Affx-110497526	7A	73532452
AX-108945597	G/G	A/G	G/G	Affx-88430531	7A	640444902
AX-108947190	C/C	C/G	G/G	Affx-109995722	7A	712155088
AX-108951117	A/G	A/G	G/G	Affx-111873035	7A	26581427
AX-108951218	C/C	T/C	C/C	Affx-111980571	7A	278469228
AX-108951250	A/A	A/A	A/A	Affx-111938762	7A	349581681
AX-108952280	T/T	T/C	T/T	Affx-110380579	7A	704616544
AX-108958957	C/C	T/C	C/C	Affx-111658045	7A	247203613
AX-108958961	C/C	A/C	A/A	Affx-109635989	7A	697130213
AX-108964908	G/G	C/G	G/G	Affx-108861884	7A	702619923
AX-108967797	C/C	T/C	C/C	Affx-111539911	7A	267561876
AX-108973008	C/C	T/C	C/C	Affx-111832452	7A	8335405
AX-108975568	T/T	T/C	C/C	Affx-109214443	7A	612230640
AX-108990455	C/C	T/C	T/T	Affx-111604869	7A	637917558
AX-109002083	C/C	T/C	C/C	Affx-111968321	7A	604824034
AX-109013825	T/T	T/C	T/T	Affx-110026078	7A	649428801
AX-109017651	G/G	G/G	G/G	Affx-111309487	7A	29666380
AX-109030424	G/G	A/G	G/G	Affx-109673369	7A	705224535
AX-109032854	A/A	A/A	A/A	Affx-110612416	7A	442593043
AX-109036201	C/C	T/C	C/C	Affx-110085611	7A	112955297
AX-109038137	G/G	A/G	G/G	Affx-111793673	7A	437683051
AX-109038621	T/T	T/C	T/T	Affx-110409407	7A	450430448
AX-109043473	T/T	T/C	T/T	Affx-109770511	7A	508174856
AX-109057260	A/A	G/G	A/A	Affx-109454441	7A	241285357
AX-109065463	A/A	A/T	A/A	Affx-110705331	7A	518756020
AX-109074928	T/T	T/C	T/T	Affx-111211720	7A	454415603
AX-109077541	C/C	T/C	C/C	Affx-110367130	7A	621996849
AX-109079129	A/G	G/G	A/A	Affx-111730938	7A	276924374
AX-109079710	G/G	A/G	G/G	Affx-109282387	7A	545936235
AX-109270297	C/C	C/C	C/C	Affx-110780260	7A	201985415
AX-109270644	A/G	A/G	A/G	Affx-109854171	7A	562611891
AX-109272527	C/C	C/G	C/C	Affx-111832417	7A	731240127
AX-109276577	G/G	A/G	G/G	Affx-111721080	7A	276416695
AX-109277664	A/A	A/G	A/A	Affx-111241575	7A	561154016
AX-109280824	T/T	T/C	T/T	Affx-109334335	7A	609573971
AX-109281496	T/T	T/G	T/T	Affx-111845017	7A	10908600
AX-109285669	C/C	T/C	C/C	Affx-111177658	7A	502381893

AX-109293629	A/A	A/G	A/A	Affx-110605522	7A	52682466
AX-109297084	A/A	G/G	A/A	Affx-109216363	7A	514780756
AX-109302004	A/A	A/G	A/A	Affx-110393057	7A	243963035
AX-109304043	G/G	G/G	G/G	Affx-109219257	7A	723078587
AX-109306246	G/G	G/G	G/G	Affx-108984187	7A	32760827
AX-109306954	A/A	A/A	A/A	Affx-110456828	7A	35770555
AX-109308592	C/C	C/C	C/C	Affx-108886124	7A	115027322
AX-109311058	T/T	T/C	T/T	Affx-111038974	7A	273721095
AX-109313511	A/A	A/G	A/A	Affx-111363848	7A	27685009
AX-109314571	C/C	T/C	C/C	Affx-109207907	7A	70269402
AX-109320449	T/T	T/C	T/T	Affx-111994234	7A	702206148
AX-109320934	A/A	G/G	G/G	Affx-110105840	7A	700601301
AX-109325531	T/T	T/C	T/T	Affx-109152368	7A	669711285
AX-109327329	G/G	A/G	G/G	Affx-111979091	7A	37325145
AX-109328756	T/T	T/C	T/T	Affx-111262444	7A	732140958
AX-109331849	T/T	T/C	T/T	Affx-110602145	7A	21356352
AX-109334924	T/T	T/C	T/T	Affx-110822874	7A	200600947
AX-109336244	T/T	T/C	T/T	Affx-109933796	7A	390912610
AX-109337723	C/C	T/C	C/C	Affx-111871803	7A	570763890
AX-109343005	T/T	T/C	T/T	Affx-111461967	7A	155642772
AX-109346798	C/C	C/C	C/C	Affx-108926726	7A	729568062
AX-109348950	G/G	A/A	G/G	Affx-110875543	7A	716240732
AX-109349188	T/T	T/G	T/T	Affx-111761373	7A	596887507
AX-109352833	C/C	T/C	C/C	Affx-111370693	7A	521500742
AX-109356822	A/A	A/T	A/A	Affx-111934693	7A	403940524
AX-109360436	A/A	A/C	A/A	Affx-88667696	7A	573673654
AX-109361015	C/C	C/C	C/C	Affx-110252125	7A	479701092
AX-109362221	A/A	A/G	A/A	Affx-109976286	7A	368275326
AX-109366562	A/A	A/C	A/C	Affx-111815109	7A	647909350
AX-109370830	G/G	C/G	G/G	Affx-111794097	7A	677885666
AX-109379483	T/T	T/C	T/T	Affx-111347868	7A	448472921
AX-109383366	G/G	T/G	G/G	Affx-109336535	7A	147014252
AX-109383675	G/G	C/G	G/G	Affx-109463416	7A	532803491
AX-109387763	T/T	T/C	T/T	Affx-111789962	7A	458928689
AX-109389579	G/G	A/G	G/G	Affx-110548423	7A	617251534
AX-109392866	A/A	G/G	A/A	Affx-111620071	7A	491332297
AX-109395843	C/C	T/C	C/C	Affx-109439605	7A	211252984
AX-109401514	T/T	G/G	T/T	Affx-109317098	7A	15694617
AX-109401922	G/G	C/G	C/C	Affx-109202193	7A	10862047
AX-109401981	C/C	T/C	C/C	Affx-88771964	7A	127601935
AX-109402351	T/T	T/C	T/T	Affx-88619581	7A	203527622
AX-109407614	G/G	A/G	A/A	Affx-110560750	7A	682131388
AX-109412011	T/T	T/C	T/T	Affx-110455684	7A	313450726
AX-109412755	G/G	A/G	G/G	Affx-88460904	7A	516916105
AX-109414808	G/G	A/G	G/G	Affx-88798573	7A	154687132
AX-109418049	G/G	A/G	G/G	Affx-108978607	7A	124409064
AX-109418641	G/G	A/G	G/G	Affx-110908946	7A	221674914
AX-109420173	G/G	A/G	G/G	Affx-110871607	7A	71956475
AX-109423906	G/G	A/G	A/A	Affx-109132745	7A	680838030
AX-109443835	T/T	T/C	T/T	Affx-109434907	7A	436739090
AX-109443884	G/G	A/A	G/G	Affx-111345835	7A	229104499
AX-109449565	A/A	A/G	A/A	Affx-110980112	7A	117272565
AX-109454434	G/G	T/G	G/G	Affx-109736160	7A	617852995
AX-109455516	T/T	T/C	C/C	Affx-111570719	7A	681438512

AX-109455860	T/T	T/T	T/T	Affx-109508187	7A	313622796
AX-109457663	T/T	T/C	T/T	Affx-109660015	7A	51791594
AX-109467590	G/G	A/A	G/G	Affx-88489840	7A	540282515
AX-109468342	C/C	T/T	C/C	Affx-110580372	7A	503859770
AX-109470371	G/G	A/G	G/G	Affx-109306495	7A	76989606
AX-109475478	C/C	T/C	C/C	Affx-109842757	7A	9994886
AX-109482315	A/A	A/G	A/A	Affx-109832333	7A	703436711
AX-109487784	C/C	T/C	C/C	Affx-110390655	7A	152974268
AX-109494273	G/G	A/G	A/A	Affx-111473805	7A	672353266
AX-109496438	C/C	T/C	C/C	Affx-109586141	7A	486452668
AX-109508288	A/A	A/G	A/A	Affx-111624603	7A	511696928
AX-109515000	C/C	C/G	C/C	Affx-110813029	7A	159334829
AX-109518531	A/A	A/C	A/A	Affx-110265689	7A	121631419
AX-109539316	G/G	A/G	G/G	Affx-110366320	7A	550305969
AX-109542558	A/G	A/G	G/G	Affx-110506388	7A	610190891
AX-109549387	G/G	A/G	G/G	Affx-110736570	7A	175241287
AX-109561279	T/T	T/T	T/T	Affx-110542118	7A	544897932
AX-109564837	A/A	A/G	A/A	Affx-109212182	7A	35767258
AX-109576602	C/C	A/A	C/C	Affx-111509478	7A	527976835
AX-109581304	G/G	C/G	G/G	Affx-110652073	7A	285270089
AX-109585556	T/T	A/T	T/T	Affx-109790675	7A	48054483
AX-109588412	C/C	C/G	C/C	Affx-109678245	7A	588722219
AX-109598170	C/C	T/T	C/C	Affx-110213465	7A	528357522
AX-109599927	G/G	T/G	G/G	Affx-110830790	7A	261374316
AX-109654998	C/G	C/C	C/G	Affx-110732101	7A	525830958
AX-109816247	C/C	T/C	C/C	Affx-111984565	7A	172333317
AX-109817027	A/A	A/G	A/A	Affx-111653539	7A	573089065
AX-109824049	G/G	C/G	C/C	Affx-110420785	7A	717036233
AX-109825918	C/C	T/C	C/C	Affx-109480153	7A	233645211
AX-109826698	C/C	T/C	C/C	Affx-109114270	7A	604978689
AX-109834712	C/C	A/C	C/C	Affx-109430662	7A	501173531
AX-109836149	C/C	A/C	A/C	Affx-110439385	7A	25010081
AX-109839610	T/T	T/C	T/T	Affx-110611938	7A	603720481
AX-109848140	T/T	T/C	T/T	Affx-109356147	7A	463222829
AX-109848988	G/G	C/G	G/G	Affx-109526230	7A	86820252
AX-109849684	T/T	T/C	T/T	Affx-110022374	7A	553387349
AX-109853784	G/G	A/G	G/G	Affx-110720019	7A	719569010
AX-109855326	T/T	T/C	T/T	Affx-110926663	7A	70828911
AX-109855508	T/T	T/C	T/T	Affx-111506434	7A	507305525
AX-109855997	G/G	A/G	G/G	Affx-111284753	7A	616228298
AX-109860609	C/C	T/C	C/C	Affx-111366842	7A	287181966
AX-109861451	C/C	C/G	C/C	Affx-109801388	7A	448189278
AX-109864896	G/G	C/G	G/G	Affx-109828645	7A	241354455
AX-109866369	A/A	A/G	A/A	Affx-110106728	7A	39258056
AX-109868635	G/G	G/G	G/G	Affx-111709561	7A	36485440
AX-109870887	C/C	C/G	C/C	Affx-111635336	7A	143342968
AX-109871720	T/T	T/C	T/T	Affx-110064104	7A	339132775
AX-109874881	C/C	T/C	C/C	Affx-111622511	7A	662659668
AX-109881245	G/G	G/G	G/G	Affx-88604876	7A	173063197
AX-109881798	G/G	A/G	G/G	Affx-110357953	7A	150978807
AX-109883167	G/G	A/G	G/G	Affx-110468920	7A	639546921
AX-109883919	A/A	A/C	A/A	Affx-109312497	7A	505703489
AX-109885855	G/G	A/G	G/G	Affx-111694209	7A	151448400
AX-109889341	A/A	A/G	G/G	Affx-111091644	7A	689045357

AX-109892640	C/C	T/C	C/C	Affx-111169623	7A	686243498
AX-109908073	C/C	T/C	C/C	Affx-110161395	7A	528657923
AX-109916402	C/C	A/C	C/C	Affx-108937721	7A	33903713
AX-109916530	A/A	A/G	A/A	Affx-109261380	7A	137014930
AX-109916695	T/T	T/C	C/C	Affx-110477255	7A	694358656
AX-109917494	G/G	A/G	G/G	Affx-109514706	7A	736494885
AX-109919252	T/T	T/C	T/T	Affx-108924121	7A	264936834
AX-109920251	C/C	A/C	C/C	Affx-110484095	7A	115511014
AX-109921979	C/C	C/G	C/C	Affx-109341961	7A	589773792
AX-109923764	G/G	G/G	G/G	Affx-109359399	7A	159719667
AX-109930452	G/G	T/T	G/G	Affx-109826022	7A	82305435
AX-109937464	C/C	C/C	C/C	Affx-110217275	7A	122210437
AX-109938309	T/C	C/C	C/C	Affx-88455944	7A	657719711
AX-109940210	C/C	T/T	C/C	Affx-111200449	7A	644858583
AX-109940955	T/T	C/C	C/C	Affx-109395319	7A	643967438
AX-109942861	T/C	T/T	C/C	Affx-111318158	7A	19544997
AX-109944167	A/A	A/G	A/A	Affx-110623168	7A	586351756
AX-109949618	G/G	T/G	G/G	Affx-111732656	7A	286244638
AX-109950970	G/G	T/G	G/G	Affx-109815914	7A	531743775
AX-109952999	A/A	A/G	A/A	Affx-110164057	7A	81303838
AX-109953034	G/G	A/G	G/G	Affx-111180151	7A	537764187
AX-109954104	T/T	T/C	T/T	Affx-88697978	7A	517514878
AX-109957910	A/A	A/G	A/A	Affx-109769411	7A	449491276
AX-109961652	G/G	G/G	T/T	Affx-108889753	7A	683471763
AX-109962818	T/T	T/T	T/T	Affx-110892279	7A	438430857
AX-109963107	C/C	T/C	C/C	Affx-109817911	7A	424906611
AX-109963196	G/G	C/G	G/G	Affx-109967943	7A	221280786
AX-109982878	G/G	G/G	G/G	Affx-111679263	7A	729337002
AX-109983514	C/C	T/C	C/C	Affx-110266582	7A	561888353
AX-109984821	G/G	A/G	G/G	Affx-110799094	7A	34993598
AX-109985106	C/C	C/C	C/C	Affx-109525319	7A	281131367
AX-109985385	C/C	A/C	A/A	Affx-109027364	7A	660168492
AX-109987097	C/C	C/C	T/T	Affx-110858887	7A	715708027
AX-109993004	T/T	T/C	T/T	Affx-110477984	7A	501651941
AX-109993124	G/G	A/G	G/G	Affx-109869788	7A	564258148
AX-109993614	A/A	A/G	A/A	Affx-111106517	7A	347258820
AX-110002935	C/C	C/C	C/C	Affx-111997173	7A	158408329
AX-110007752	A/A	A/G	A/A	Affx-111276166	7A	730855963
AX-110009447	T/T	A/T	T/T	Affx-110797009	7A	711173258
AX-110011564	A/A	A/C	A/A	Affx-110275939	7A	350755960
AX-110012925	G/G	A/G	G/G	Affx-111660133	7A	163787723
AX-110019363	C/C	C/G	C/C	Affx-111289493	7A	153833847
AX-110020391	A/A	A/G	A/A	Affx-110279980	7A	410886142
AX-110023493	A/A	A/G	A/A	Affx-111667702	7A	266543671
AX-110024904	T/T	T/C	T/T	Affx-111358138	7A	224492523
AX-110027154	A/A	A/A	A/A	Affx-111409633	7A	502755015
AX-110037474	T/T	T/G	T/T	Affx-111837343	7A	247986261
AX-110039820	A/A	G/G	A/A	Affx-111125122	7A	596017037
AX-110041691	G/G	C/G	G/G	Affx-109504211	7A	498451011
AX-110046491	C/C	T/C	C/C	Affx-108926485	7A	145706907
AX-110049225	A/A	A/G	A/A	Affx-111227611	7A	75060424
AX-110054925	T/T	T/C	T/T	Affx-109399959	7A	135903747
AX-110062089	G/G	A/G	G/G	Affx-109101113	7A	57703226
AX-110095884	T/T	T/G	T/T	Affx-111238684	7A	243646787

AX-110106523	C/C	T/C	C/C	Affx-109126471	7A	145469493
AX-110109492	G/G	A/G	G/G	Affx-109337729	7A	58844570
AX-110109669	A/A	A/G	A/A	Affx-110106862	7A	277448223
AX-110123647	C/C	A/C	C/C	Affx-109471347	7A	13888196
AX-110131184	T/T	T/C	T/T	Affx-111395339	7A	35736231
AX-110134684	T/T	T/G	T/T	Affx-109624328	7A	598391038
AX-110164642	G/G	A/A	G/G	Affx-110175208	7A	510142058
AX-110167145	T/T	T/C	T/T	Affx-108938958	7A	117764161
AX-110169498	C/C	C/G	C/C	Affx-109839916	7A	604634616
AX-110173198	G/G	A/G	G/G	Affx-112001117	7A	262882078
AX-110362176	T/T	A/T	T/T	Affx-109874480	7A	595306645
AX-110367116	T/T	T/G	T/T	Affx-111670045	7A	565673079
AX-110369401	A/A	A/G	A/A	Affx-109873672	7A	375868838
AX-110371615	C/C	A/C	C/C	Affx-111225458	7A	227537031
AX-110373458	T/T	C/C	T/T	Affx-110035657	7A	538867721
AX-110373955	T/T	T/C	T/T	Affx-109376523	7A	600705008
AX-110375032	A/A	A/G	A/A	Affx-109804811	7A	178684081
AX-110376315	C/C	A/C	C/C	Affx-109664453	7A	613641473
AX-110381088	G/G	A/G	G/G	Affx-111087317	7A	135002090
AX-110382617	G/G	A/G	G/G	Affx-109336602	7A	51422162
AX-110385412	C/C	T/C	C/C	Affx-112003031	7A	484108065
AX-110386466	A/A	A/C	A/A	Affx-109301479	7A	374830103
AX-110387817	G/G	A/G	G/G	Affx-110897637	7A	485153032
AX-110391898	C/C	A/A	C/C	Affx-110488255	7A	552682454
AX-110394120	T/T	T/C	T/T	Affx-110478963	7A	25855136
AX-110398543	A/T	A/T	A/A	Affx-111385949	7A	642864251
AX-110400113	T/T	T/C	T/T	Affx-109727061	7A	110969688
AX-110408234	C/C	T/C	C/C	Affx-109128405	7A	170681931
AX-110411830	A/C	A/C	C/C	Affx-111653438	7A	155162999
AX-110415860	G/G	A/G	G/G	Affx-111967106	7A	129807095
AX-110423393	T/T	T/C	T/T	Affx-110802512	7A	112008227
AX-110426981	T/T	T/C	T/T	Affx-110692368	7A	69956773
AX-110427241	G/G	A/G	G/G	Affx-109992248	7A	571058110
AX-110427622	C/C	T/C	C/C	Affx-108994352	7A	569883408
AX-110430243	C/C	T/C	C/C	Affx-109768159	7A	670500650
AX-110430246	A/A	A/G	A/A	Affx-109844807	7A	347838437
AX-110433324	T/T	T/C	T/T	Affx-110764056	7A	271723318
AX-110441274	G/G	A/G	G/G	Affx-110739569	7A	174837011
AX-110442627	C/C	C/G	C/C	Affx-111015009	7A	473808119
AX-110444154	A/A	A/G	A/A	Affx-88549445	7A	448097564
AX-110445761	C/C	T/C	T/T	Affx-110891250	7A	610650515
AX-110450237	A/A	A/G	A/A	Affx-109460768	7A	536520597
AX-110454282	G/G	A/G	G/G	Affx-88449141	7A	512753658
AX-110463440	C/C	T/C	C/C	Affx-88706684	7A	490165825
AX-110465643	T/T	T/C	T/T	Affx-111475182	7A	280073160
AX-110466533	G/G	A/G	A/A	Affx-111648136	7A	612031314
AX-110467153	C/C	T/C	C/C	Affx-110988048	7A	6804366
AX-110471584	G/G	A/G	G/G	Affx-110628561	7A	96571668
AX-110474565	C/C	T/C	C/C	Affx-109184032	7A	149293288
AX-110476160	C/C	T/C	C/C	Affx-111377789	7A	702995726
AX-110477239	G/G	A/G	G/G	Affx-110119336	7A	119130369
AX-110478067	G/G	A/G	G/G	Affx-110385696	7A	696725961
AX-110481831	C/C	T/C	T/T	Affx-109666067	7A	695626012
AX-110482447	C/C	C/G	C/C	Affx-111805778	7A	16522790

AX-110483276	A/A	A/G	A/A	Affx-111640917	7A	268860063
AX-110483438	T/T	T/C	T/C	Affx-109100485	7A	634828503
AX-110487843	T/T	T/C	T/T	Affx-110232562	7A	251257641
AX-110493635	C/C	T/C	C/C	Affx-111251878	7A	127127284
AX-110494040	C/C	A/C	C/C	Affx-109131710	7A	348409497
AX-110496630	C/C	C/C	C/C	Affx-111623395	7A	439804475
AX-110498109	G/G	G/G	G/G	Affx-111748158	7A	283787330
AX-110499844	G/G	A/G	G/G	Affx-110293786	7A	1128317
AX-110501928	G/G	C/G	G/G	Affx-110217422	7A	620936613
AX-110507924	T/T	T/C	T/T	Affx-110576897	7A	270758817
AX-110512897	T/T	T/C	T/T	Affx-109592606	7A	16119089
AX-110513921	C/C	A/C	C/C	Affx-111600391	7A	28154801
AX-110514333	A/A	A/G	A/A	Affx-110938729	7A	102557054
AX-110515818	T/T	T/C	T/T	Affx-110593178	7A	31417676
AX-110516759	G/G	G/G	G/G	Affx-110493676	7A	543043362
AX-110531849	A/A	A/G	A/A	Affx-110909969	7A	499534906
AX-110534898	T/T	A/T	A/A	Affx-109704112	7A	644439973
AX-110538185	A/G	A/G	A/G	Affx-110709764	7A	69310008
AX-110539491	G/G	A/G	G/G	Affx-111604729	7A	453597490
AX-110541056	A/A	A/A	C/C	Affx-110781534	7A	682065285
AX-110547295	T/T	T/T	T/T	Affx-111356581	7A	284432259
AX-110565790	C/C	T/C	T/T	Affx-111955301	7A	684232267
AX-110565937	G/G	A/G	G/G	Affx-109871825	7A	510646953
AX-110571496	A/A	A/C	A/A	Affx-110516297	7A	282734987
AX-110575187	A/A	A/A	A/A	Affx-111615205	7A	62732718
AX-110575956	G/G	A/G	G/G	Affx-110764133	7A	85429456
AX-110576624	T/T	T/T	T/T	Affx-110345888	7A	712424146
AX-110577867	G/G	A/G	G/G	Affx-111446071	7A	441967473
AX-110581206	G/G	G/G	G/G	Affx-109047353	7A	272269873
AX-110582327	G/G	G/G	G/G	Affx-111640811	7A	284778512
AX-110587452	C/C	T/C	C/C	Affx-108853874	7A	229370997
AX-110595051	A/A	A/G	A/A	Affx-109868043	7A	455092740
AX-110599789	G/G	A/G	G/G	Affx-110901442	7A	262456531
AX-110602143	G/G	A/G	G/G	Affx-111732451	7A	574968434
AX-110602623	A/A	A/G	A/A	Affx-108902675	7A	441837287
AX-110602962	A/A	A/G	A/A	Affx-111213837	7A	90369575
AX-110611360	G/G	A/G	G/G	Affx-109576569	7A	446973155
AX-110612704	C/C	A/C	C/C	Affx-110801828	7A	405736928
AX-110623050	G/G	A/G	G/G	Affx-110058079	7A	574113860
AX-110627601	C/C	C/G	C/C	Affx-109150762	7A	584389646
AX-110640053	T/T	C/C	C/C	Affx-110108570	7A	652912511
AX-110660898	C/C	T/C	C/C	Affx-110587835	7A	364002481
AX-110667702	A/A	A/A	A/A	Affx-111776715	7A	108357136
AX-110667982	C/C	C/C	C/C	Affx-108875090	7A	166725288
AX-110671654	T/T	T/C	T/T	Affx-109782074	7A	506977908
AX-110677280	G/G	A/G	A/A	Affx-109939443	7A	673511134
AX-110680063	G/G	A/G	G/G	Affx-109367780	7A	92742178
AX-110690592	A/A	A/C	A/A	Affx-111650292	7A	565696022
AX-110690944	T/T	T/C	T/T	Affx-111650833	7A	571928348
AX-110694117	T/T	T/C	T/T	Affx-111630689	7A	110763207
AX-110697799	G/G	A/G	G/G	Affx-109313499	7A	49575505
AX-110712533	T/T	T/C	T/T	Affx-110474634	7A	661600000
AX-110714027	G/G	A/A	G/G	Affx-111168082	7A	512125695
AX-110908706	G/G	A/G	G/G	Affx-110575814	7A	706363667

AX-110911418	G/G	T/G	G/G	Affx-111711709	7A	199756835
AX-110913554	G/G	A/G	G/G	Affx-109557258	7A	531018966
AX-110913966	T/T	T/C	T/T	Affx-111355010	7A	176418706
AX-110920769	C/C	A/C	C/C	Affx-110679633	7A	201534350
AX-110922774	T/T	T/C	T/T	Affx-110954035	7A	592084413
AX-110923081	C/C	T/T	C/C	Affx-111080047	7A	122919517
AX-110923765	T/T	T/C	T/T	Affx-111909264	7A	435725519
AX-110925530	C/C	T/T	C/C	Affx-111569746	7A	725129384
AX-110928349	A/A	A/A	C/C	Affx-109238840	7A	674103324
AX-110937720	T/T	T/C	C/C	Affx-110537652	7A	693333071
AX-110947353	C/C	T/C	C/C	Affx-110160734	7A	651633553
AX-110950610	A/C	A/C	A/C	Affx-111025574	7A	200027774
AX-110951250	T/T	T/C	T/T	Affx-109072093	7A	244495255
AX-110952157	C/C	C/G	C/C	Affx-109669895	7A	66176328
AX-110968267	A/G	A/G	A/G	Affx-111243691	7A	402580859
AX-110970196	G/G	A/G	G/G	Affx-108921962	7A	30227813
AX-110972216	T/T	T/C	T/T	Affx-110696971	7A	170832686
AX-110973098	T/C	T/C	T/T	Affx-110085129	7A	675095331
AX-110973987	G/G	G/G	G/G	Affx-111054841	7A	77528921
AX-110974209	A/A	A/G	A/A	Affx-110937889	7A	504792925
AX-110976573	G/G	T/G	G/G	Affx-111944304	7A	270391280
AX-110978409	T/T	T/C	T/T	Affx-108909806	7A	292388498
AX-110981836	G/G	A/G	G/G	Affx-110523203	7A	589244709
AX-110988406	G/G	T/G	G/G	Affx-109441614	7A	509036511
AX-110998156	A/A	A/G	A/A	Affx-109843700	7A	571479842
AX-111001693	T/T	T/C	T/T	Affx-109800565	7A	271160712
AX-111002562	G/G	C/G	G/G	Affx-108925497	7A	13177990
AX-111011558	C/C	T/C	C/C	Affx-111542344	7A	35703756
AX-111013206	C/C	T/C	C/C	Affx-109447799	7A	729334584
AX-111018143	G/G	C/G	G/G	Affx-110309260	7A	278980999
AX-111027087	G/G	G/G	G/G	Affx-111878182	7A	440359149
AX-111027195	C/C	T/C	C/C	Affx-109229013	7A	529807391
AX-111028422	A/G	G/G	A/G	Affx-111087360	7A	576384680
AX-111028721	G/G	A/G	G/G	Affx-110579698	7A	266156267
AX-111030306	C/G	C/G	C/C	Affx-111287862	7A	24459650
AX-111031978	C/C	C/C	C/C	Affx-109222580	7A	555934528
AX-111033081	T/G	T/G	T/G	Affx-109710849	7A	234312220
AX-111034263	C/C	T/C	T/T	Affx-108863066	7A	646984252
AX-111034531	C/C	T/C	C/C	Affx-109084182	7A	349601232
AX-111035251	T/T	T/C	T/T	Affx-110657211	7A	142172252
AX-111036083	G/G	A/G	G/G	Affx-111774782	7A	285809848
AX-111038237	A/G	A/G	A/A	Affx-110890874	7A	637058250
AX-111038846	A/G	A/G	A/G	Affx-88413693	7A	527192621
AX-111039707	T/T	T/C	T/T	Affx-109246756	7A	412707408
AX-111040747	C/C	A/C	C/C	Affx-111544393	7A	484604073
AX-111044081	C/C	T/C	C/C	Affx-109227354	7A	168162721
AX-111045618	C/C	T/C	C/C	Affx-110818322	7A	649050388
AX-111047035	C/C	C/C	C/C	Affx-111308900	7A	533566242
AX-111047991	T/T	T/C	T/T	Affx-110697515	7A	468552494
AX-111048148	T/T	T/C	T/T	Affx-108906337	7A	531404450
AX-111049614	A/A	A/C	A/A	Affx-109236611	7A	729361052
AX-111050940	T/T	G/G	T/T	Affx-111112233	7A	669776436
AX-111055093	G/G	G/G	G/G	Affx-111835311	7A	437431952
AX-111055662	T/T	T/C	T/T	Affx-109217535	7A	354491775

AX-111056118	G/G	T/T	T/G	Affx-109046095	7A	206690963
AX-111064027	A/A	G/G	G/G	Affx-109888201	7A	1880264
AX-111064790	G/G	G/G	G/G	Affx-109187904	7A	281664956
AX-111070378	T/T	T/C	T/T	Affx-110618001	7A	340410229
AX-111073226	A/T	A/A	A/T	Affx-111681860	7A	383199792
AX-111073918	A/A	A/C	A/A	Affx-111703605	7A	470125685
AX-111076418	G/G	A/G	G/G	Affx-110973911	7A	8907477
AX-111095229	T/T	C/C	T/T	Affx-110281037	7A	506125843
AX-111096798	T/T	T/G	T/T	Affx-111098177	7A	625682847
AX-111096994	A/A	A/G	A/A	Affx-110569144	7A	456056180
AX-111107307	T/T	T/C	T/T	Affx-111676434	7A	651204227
AX-111110609	T/T	T/C	T/T	Affx-109098735	7A	456771911
AX-111113109	T/T	T/C	T/T	Affx-109690102	7A	78500883
AX-111113709	A/A	A/G	A/A	Affx-111529551	7A	419349967
AX-111114292	C/C	T/C	C/C	Affx-109055059	7A	321542868
AX-111115218	A/A	G/G	G/G	Affx-111894417	7A	720358804
AX-111122299	G/G	T/G	G/G	Affx-108975464	7A	282653427
AX-111123066	C/C	T/C	C/C	Affx-108994163	7A	55431597
AX-111133278	C/C	T/C	C/C	Affx-109387580	7A	169950681
AX-111134025	A/A	A/G	A/A	Affx-109383894	7A	600342409
AX-111139279	C/C	C/C	C/C	Affx-111414057	7A	231338553
AX-111145463	C/C	T/C	T/C	Affx-109095350	7A	149419196
AX-111149648	T/C	T/T	T/C	Affx-109967836	7A	21105596
AX-111151663	C/C	C/C	C/C	Affx-110498520	7A	84853031
AX-111152094	G/G	A/G	G/G	Affx-110381226	7A	169116644
AX-111153338	C/C	A/C	A/A	Affx-111328962	7A	701150627
AX-111155649	T/T	C/C	T/T	Affx-110093698	7A	494700469
AX-111157266	A/A	G/G	G/G	Affx-109577211	7A	719720445
AX-111157953	G/G	A/G	G/G	Affx-110725936	7A	646673200
AX-111193397	T/C	C/C	T/C	Affx-111989042	7A	374531964
AX-111215580	A/A	A/C	A/A	Affx-88380833	7A	611232522
AX-111218466	C/C	C/G	C/C	Affx-109287589	7A	232339492
AX-111218946	A/A	A/G	A/A	Affx-108977700	7A	260927024
AX-111235463	A/A	A/G	A/A	Affx-111404796	7A	569748156
AX-111247753	T/T	C/C	T/T	Affx-109550837	7A	345925930
AX-111251419	G/G	A/G	G/G	Affx-110872212	7A	455762099
AX-111254187	G/G	A/G	A/A	Affx-111709343	7A	711580181
AX-111278531	A/A	A/A	A/A	Affx-109869091	7A	280690671
AX-111278810	A/A	A/G	A/A	Affx-109331497	7A	252473892
AX-111450137	C/C	C/C	C/C	Affx-88608337	7A	53289120
AX-111450326	C/C	T/C	C/C	Affx-111014448	7A	215177875
AX-111450329	A/A	A/G	A/A	Affx-109949688	7A	389194409
AX-111450960	C/C	T/C	C/C	Affx-109430327	7A	205143046
AX-111452135	T/T	T/C	T/T	Affx-111397519	7A	148473862
AX-111453830	T/T	T/C	T/T	Affx-110868276	7A	491978301
AX-111454416	G/G	A/G	G/G	Affx-110541593	7A	496922269
AX-111460093	T/T	T/C	C/C	Affx-109938772	7A	9162784
AX-111460219	A/A	G/G	A/A	Affx-109889635	7A	619291317
AX-111460714	C/C	T/C	C/C	Affx-110896758	7A	274911468
AX-111463511	G/G	A/G	G/G	Affx-109994454	7A	107148210
AX-111464820	G/G	G/G	G/G	Affx-110980003	7A	12126517
AX-111472858	T/T	T/C	T/T	Affx-111818145	7A	287185901
AX-111474126	C/C	T/C	C/C	Affx-109927866	7A	22947083
AX-111475110	A/A	A/G	A/A	Affx-111893628	7A	331893825

AX-111478706	A/A	A/A	A/A	Affx-111364057	7A	135523364
AX-111486899	G/G	C/G	G/G	Affx-109322746	7A	191861132
AX-111488311	C/C	A/C	C/C	Affx-109859663	7A	33379848
AX-111491072	C/C	T/C	C/C	Affx-88746654	7A	52078448
AX-111491167	T/T	C/C	T/T	Affx-88807827	7A	230865361
AX-111491830	C/C	T/C	C/C	Affx-111717407	7A	590116129
AX-111491951	C/C	A/C	C/C	Affx-109052836	7A	46257001
AX-111496081	A/A	A/C	A/A	Affx-109556272	7A	29316810
AX-111497147	G/G	T/G	G/G	Affx-110253285	7A	487254583
AX-111497520	A/A	A/G	A/A	Affx-111283803	7A	538781672
AX-111498708	C/C	T/C	C/C	Affx-109768062	7A	15111488
AX-111500963	C/C	T/C	C/C	Affx-109887507	7A	537057222
AX-111504746	T/T	T/C	T/T	Affx-109739512	7A	189330912
AX-111505383	T/T	T/C	T/T	Affx-111644739	7A	641954519
AX-111506594	G/G	G/G	G/G	Affx-109980295	7A	35767381
AX-111510914	C/C	C/G	C/C	Affx-110212055	7A	729645625
AX-111511322	C/C	T/C	C/C	Affx-108884266	7A	657737118
AX-111514585	C/C	T/C	C/C	Affx-110026347	7A	279847024
AX-111526733	T/T	T/C	T/T	Affx-109416284	7A	273454346
AX-111526854	T/T	T/C	T/T	Affx-110933124	7A	545540546
AX-111527171	T/T	T/G	T/T	Affx-111280405	7A	267805287
AX-111527884	A/A	A/G	A/A	Affx-109598695	7A	591935136
AX-111530810	A/A	A/G	A/A	Affx-109445061	7A	198197403
AX-111530956	G/G	A/G	A/A	Affx-110164661	7A	645098407
AX-111532754	C/C	C/C	C/C	Affx-112313944	7A	108760202
AX-111536100	C/C	T/C	C/C	Affx-109089947	7A	68858419
AX-111537301	A/A	A/G	A/A	Affx-111743601	7A	76369386
AX-111539265	C/C	T/C	C/C	Affx-109299810	7A	526792383
AX-111544318	C/C	T/C	T/T	Affx-109064922	7A	638519956
AX-111551571	A/A	A/G	A/A	Affx-108986774	7A	65629832
AX-111551602	C/C	T/C	C/C	Affx-108991121	7A	473466418
AX-111558895	G/G	A/G	G/G	Affx-110326654	7A	563154675
AX-111569442	G/G	C/G	G/G	Affx-108932567	7A	116496069
AX-111572368	G/G	A/A	G/G	Affx-109592534	7A	278454240
AX-111574362	C/C	A/C	C/C	Affx-109852205	7A	625062717
AX-111584092	C/C	C/G	C/C	Affx-110028159	7A	275939077
AX-111585581	C/C	A/C	C/C	Affx-110870135	7A	665973294
AX-111586310	T/T	T/C	T/T	Affx-108851327	7A	402838791
AX-111589791	G/G	A/A	G/G	Affx-110419600	7A	202689901
AX-111589956	C/C	T/C	C/C	Affx-111468536	7A	32349397
AX-111591978	T/T	T/C	T/T	Affx-110160676	7A	436176653
AX-111598459	A/A	A/G	A/A	Affx-109540351	7A	572328981
AX-111602863	G/G	A/G	A/A	Affx-111367920	7A	659376423
AX-111605774	A/A	A/G	A/A	Affx-111892479	7A	583567038
AX-111610270	T/T	T/C	T/T	Affx-110263733	7A	31862949
AX-111610354	T/T	A/T	T/T	Affx-110547774	7A	238329852
AX-111614486	A/A	A/G	A/A	Affx-111064762	7A	26101067
AX-111619425	T/T	A/T	T/T	Affx-109040257	7A	79654213
AX-111627884	C/C	T/C	C/C	Affx-110269460	7A	43489030
AX-111628095	C/C	T/C	C/C	Affx-110030168	7A	580056957
AX-111635435	A/A	A/G	A/A	Affx-109943406	7A	234529481
AX-111636150	A/A	A/G	A/A	Affx-109116551	7A	367003512
AX-111638232	T/T	T/T	T/T	Affx-111082644	7A	80313737
AX-111638724	A/A	A/G	A/A	Affx-109797294	7A	170683740

AX-111640994	G/G	A/G	G/G	Affx-111654025	7A	543421370
AX-111644847	C/C	C/G	C/C	Affx-111353460	7A	22223571
AX-111645595	C/C	T/C	C/C	Affx-110404781	7A	41629778
AX-111648929	G/G	A/G	G/G	Affx-109606808	7A	85906187
AX-111651685	C/C	C/C	C/C	Affx-110447004	7A	580657787
AX-111653538	G/G	C/G	C/C	Affx-111434270	7A	714346808
AX-111662651	G/G	A/G	G/G	Affx-111552170	7A	66638331
AX-111663504	A/A	A/G	A/A	Affx-111556063	7A	114412492
AX-111664463	A/A	A/A	A/A	Affx-110047085	7A	123203710
AX-111672660	T/T	T/C	T/T	Affx-110933496	7A	164659140
AX-111674440	G/G	A/G	G/G	Affx-88777421	7A	195067554
AX-111676021	C/C	C/G	C/C	Affx-109316585	7A	701622837
AX-111678051	C/C	C/G	C/C	Affx-111056254	7A	735399772
AX-111685929	T/G	T/T	T/T	Affx-111413736	7A	673301623
AX-111691613	A/A	A/G	A/A	Affx-108989942	7A	14452077
AX-111691966	T/T	T/C	T/T	Affx-110603673	7A	262288280
AX-111721077	A/A	A/A	A/A	Affx-109115287	7A	506485751
AX-111726746	T/T	T/C	T/T	Affx-88772446	7A	53906949
AX-111738947	T/T	T/T	T/C	Affx-109226911	7A	671928476
AX-111761347	C/C	T/C	C/C	Affx-110369923	7A	442629694
AX-111776567	G/G	G/G	G/G	Affx-109892999	7A	169790292
AX-111796909	T/C	T/C	C/C	Affx-109181717	7A	608455615
AX-182019225	A/A	A/A	A/A	Affx-109962852	7A	170681735
AX-182063263	G/G	A/G	G/G	Affx-472309437	7A	50019980
AX-182077315	C/C	C/G	C/C	Affx-88602758	7A	35735644
AX-182077633	G/G	G/G	G/G	Affx-88429105	7A	35771174
AX-86171165	A/G	A/G	A/G	Affx-88783719	7A	660464836
AX-86176537	T/T	T/T	T/T	Affx-92557559	7A	63111864
AX-86178179	G/G	G/G	G/G	Affx-92580440	7A	54997958
AX-86178313	T/T	T/C	T/T	Affx-88447178	7A	729397498
AX-86179336	A/A	A/G	A/A	Affx-92347270	7A	8895372
AX-86179508	G/G	A/G	G/G	Affx-92664933	7A	603294449
AX-89336066	C/G	C/G	C/G	Affx-88367536	7A	35693731
AX-89542612	G/G	C/C	G/G	Affx-88575607	7A	42500174
AX-94384726	A/A	A/A	A/A	Affx-92544375	7A	592907405
AX-94395845	G/G	A/G	G/G	Affx-92354654	7A	28444158
AX-94431610	C/C	T/C	C/C	Affx-88423092	7A	458763873
AX-94479300	A/A	A/G	A/A	Affx-92847654	7A	134543249
AX-94505773	A/A	A/G	A/A	Affx-88521936	7A	83631494
AX-94531050	C/C	T/C	C/C	Affx-92840520	7A	696783447
AX-94586454	A/G	A/G	A/G	Affx-88416965	7A	13100987
AX-94596044	C/C	C/G	C/C	Affx-111821648	7A	490866365
AX-94598447	A/A	A/G	A/A	Affx-92948498	7A	635499219
AX-94603286	A/A	A/A	A/A	Affx-92387749	7A	725225002
AX-94613467	G/G	A/G	G/G	Affx-92507838	7A	198941503
AX-94616006	C/C	A/A	C/C	Affx-92615038	7A	205484970
AX-94638909	A/A	A/G	A/A	Affx-92241990	7A	542708854
AX-94650444	T/T	T/T	T/C	Affx-92868445	7A	724084881
AX-94681274	A/A	A/A	A/A	Affx-92229419	7A	475804567
AX-94698211	G/G	G/G	G/G	Affx-92573569	7A	129259313
AX-94713504	C/C	T/T	C/C	Affx-92274982	7A	87326031
AX-94713925	C/C	T/C	T/C	Affx-92324291	7A	18891446
AX-94793232	G/G	G/G	G/G	Affx-92911428	7A	428818886
AX-94849533	C/C	T/C	C/C	Affx-92163796	7A	709639520

AX-94926193	T/C	T/C	T/C	Affx-92559657	7A	4627065
AX-95075432	T/T	T/T	T/T	Affx-92870035	7A	35770406
AX-95080418	A/G	A/A	A/G	Affx-92728576	7A	108811168
AX-95111713	C/C	C/C	C/C	Affx-92308503	7A	729398037
AX-95197108	C/C	C/C	C/C	Affx-92332447	7A	90622871
AX-95227739	G/G	T/G	G/G	Affx-92736893	7A	102055764
AX-95239734	C/C	A/C	C/C	Affx-92367878	7A	521951985
AX-95248501	C/C	A/C	C/C	Affx-92395152	7A	443387442
AX-95654492	T/T	T/G	T/T	Affx-88420991	7A	481587624
AX-95658680	T/T	T/C	T/T	Affx-88619462	7A	11100954
AX-108727514	G/G	A/G	G/G	Affx-111736118	7B	702467752
AX-108733468	T/T	T/C	T/T	Affx-109883538	7B	517401359
AX-108739539	A/A	A/G	A/A	Affx-88804332	7B	595681330
AX-108740088	T/C	T/C	C/C	Affx-111036672	7B	739451473
AX-108743244	A/A	A/C	A/A	Affx-111871259	7B	636934739
AX-108745285	G/G	A/G	G/G	Affx-109537289	7B	559151132
AX-108745314	T/T	T/C	C/C	Affx-110980234	7B	86622987
AX-108746141	A/A	A/G	G/G	Affx-110779514	7B	718332606
AX-108748684	T/T	T/C	T/T	Affx-110662181	7B	158197801
AX-108750441	G/G	T/G	G/G	Affx-110345001	7B	129285264
AX-108751233	C/C	C/C	C/C	Affx-88482702	7B	114501292
AX-108755073	G/G	A/G	A/A	Affx-109438657	7B	480203425
AX-108759562	T/T	T/C	T/T	Affx-111277153	7B	687899458
AX-108761212	G/G	G/G	G/G	Affx-110657845	7B	165273484
AX-108762687	T/T	T/G	T/T	Affx-88704556	7B	87137825
AX-108765289	C/C	C/C	C/C	Affx-110661668	7B	121757919
AX-108765522	T/T	A/T	T/T	Affx-111294033	7B	608433419
AX-108766027	G/G	A/A	G/G	Affx-110796731	7B	237517496
AX-108768717	T/T	T/C	T/T	Affx-110836520	7B	566473938
AX-108770640	C/G	C/G	G/G	Affx-109769402	7B	333128603
AX-108772900	C/C	T/C	C/C	Affx-109299584	7B	112019148
AX-108774819	G/G	T/G	G/G	Affx-109113297	7B	611805938
AX-108776488	A/A	A/G	A/A	Affx-109595333	7B	439786873
AX-108779257	T/T	T/G	G/G	Affx-110936005	7B	510729119
AX-108779521	C/C	T/C	C/C	Affx-109279116	7B	15337790
AX-108783496	A/A	A/G	A/A	Affx-110651466	7B	52121148
AX-108783702	T/T	T/C	T/T	Affx-111712997	7B	49533903
AX-108786832	A/A	A/G	G/G	Affx-108900842	7B	742146990
AX-108795893	C/C	T/C	T/T	Affx-110746366	7B	71153685
AX-108797853	T/T	T/C	T/T	Affx-109025458	7B	573256302
AX-108801665	C/C	C/C	C/C	Affx-110864059	7B	556152709
AX-108802323	T/T	T/C	C/C	Affx-111613095	7B	57459062
AX-108808412	G/G	A/G	A/A	Affx-88648656	7B	115384259
AX-108809713	T/T	T/C	T/T	Affx-110076018	7B	122536613
AX-108813958	A/A	C/C	A/A	Affx-111379954	7B	586177191
AX-108814625	A/A	A/G	A/A	Affx-109658974	7B	588895091
AX-108818470	T/T	T/G	T/T	Affx-109612185	7B	203137274
AX-108818797	G/G	T/G	G/G	Affx-111990508	7B	143856849
AX-108819940	C/C	T/C	C/C	Affx-109690802	7B	103917626
AX-108824103	T/T	T/C	T/T	Affx-111597573	7B	612842476
AX-108826160	A/G	A/A	A/G	Affx-109982981	7B	121112897
AX-108828475	C/C	C/G	C/C	Affx-109184889	7B	678283884
AX-108829733	G/G	A/G	G/G	Affx-109030236	7B	96809478
AX-108840818	T/T	T/C	T/T	Affx-110255875	7B	577113920

AX-108849258	T/T	T/C	T/T	Affx-110669901	7B	539198681
AX-108851705	C/C	C/G	C/C	Affx-109606345	7B	693309046
AX-108852057	A/A	A/G	G/G	Affx-110765182	7B	170214761
AX-108853116	G/G	A/G	G/G	Affx-108958597	7B	705464806
AX-108854166	G/G	A/G	G/G	Affx-110527985	7B	648162197
AX-108861535	C/C	T/C	C/C	Affx-109622811	7B	643114040
AX-108870046	C/C	T/T	C/C	Affx-111194772	7B	494198748
AX-108870214	G/G	A/G	G/G	Affx-110995087	7B	644505307
AX-108886094	G/G	T/G	G/G	Affx-109571347	7B	578878380
AX-108898638	T/T	T/C	T/T	Affx-111871472	7B	538306955
AX-108904578	C/C	T/C	C/C	Affx-110656985	7B	638912901
AX-108904686	A/A	A/A	A/A	Affx-110442369	7B	165568207
AX-108912337	C/C	C/C	C/C	Affx-108949215	7B	83964304
AX-108916024	T/T	T/C	T/T	Affx-110928573	7B	657902648
AX-108917642	G/G	C/G	C/C	Affx-111484331	7B	739426374
AX-108920835	C/C	T/C	C/C	Affx-110234321	7B	614290894
AX-108929986	G/G	A/G	A/A	Affx-110020187	7B	508818410
AX-108933521	A/A	A/C	A/A	Affx-108954425	7B	569118908
AX-108938830	G/G	T/G	G/G	Affx-111076120	7B	673677947
AX-108946650	C/C	C/G	C/C	Affx-110576650	7B	330917466
AX-108952166	G/G	T/G	G/G	Affx-110662064	7B	565571347
AX-108952314	C/C	C/C	C/C	Affx-88430008	7B	194785410
AX-108965135	C/C	C/C	C/C	Affx-110782317	7B	187487473
AX-108972133	A/A	A/G	A/A	Affx-108920854	7B	520120517
AX-108972148	T/T	A/T	T/T	Affx-111587066	7B	656427481
AX-108996379	G/G	A/G	G/G	Affx-110430268	7B	570455186
AX-109036990	T/T	T/C	T/T	Affx-111785127	7B	567024913
AX-109051509	A/A	A/G	A/A	Affx-110944020	7B	616163888
AX-109052982	T/T	T/C	T/T	Affx-111413337	7B	491975243
AX-109073710	G/G	A/G	G/G	Affx-109149322	7B	571011455
AX-109076683	A/A	A/G	A/A	Affx-109428256	7B	488709339
AX-109104198	A/G	A/G	A/A	Affx-109158106	7B	750414253
AX-109275926	T/T	T/G	T/T	Affx-109535113	7B	619409195
AX-109276038	C/C	T/C	C/C	Affx-111059549	7B	639846021
AX-109278740	G/G	A/G	A/A	Affx-111597477	7B	11982545
AX-109279191	T/T	C/C	T/T	Affx-109917844	7B	547678466
AX-109286487	A/A	A/C	A/A	Affx-110445951	7B	641885147
AX-109288214	G/G	A/G	G/G	Affx-110304763	7B	45629211
AX-109294610	A/A	A/G	A/A	Affx-111081299	7B	519786031
AX-109297899	A/A	G/G	A/A	Affx-111730460	7B	633674931
AX-109300995	G/G	G/G	A/A	Affx-109540847	7B	679766501
AX-109301621	T/T	T/G	T/T	Affx-110285137	7B	506031058
AX-109302618	A/A	A/G	G/G	Affx-111474461	7B	185067482
AX-109306298	A/A	A/G	A/A	Affx-111605482	7B	142881442
AX-109311774	G/G	A/G	A/A	Affx-109303109	7B	731187649
AX-109312499	C/C	T/C	C/C	Affx-109982337	7B	140710979
AX-109315125	A/A	A/G	A/A	Affx-109049103	7B	583162024
AX-109318746	T/T	T/T	T/T	Affx-109516200	7B	167173369
AX-109324729	C/C	T/C	C/C	Affx-109970614	7B	537871709
AX-109325310	A/A	A/G	A/A	Affx-88654783	7B	153327466
AX-109325469	T/T	T/C	C/C	Affx-88804721	7B	653015750
AX-109330015	G/G	A/G	G/G	Affx-111004550	7B	231013192
AX-109330348	G/G	T/G	G/G	Affx-109082922	7B	596100475
AX-109343029	A/A	A/G	A/A	Affx-110561224	7B	494581316

AX-109352613	T/T	T/C	T/T	Affx-88370562	7B	327274463
AX-109356871	C/C	T/C	C/C	Affx-111648808	7B	123482032
AX-109360286	C/C	T/C	C/C	Affx-110464334	7B	495175978
AX-109361944	C/C	T/C	C/C	Affx-109398640	7B	17665895
AX-109362242	T/T	T/C	C/C	Affx-110585564	7B	458112975
AX-109363340	C/C	A/C	C/C	Affx-109185979	7B	355403232
AX-109365550	C/C	T/C	C/C	Affx-109125914	7B	546023659
AX-109369106	T/C	C/C	C/C	Affx-109635784	7B	718355064
AX-109371862	T/T	T/C	T/T	Affx-111158775	7B	704111622
AX-109372990	C/C	T/C	C/C	Affx-88784275	7B	19324835
AX-109375143	C/C	T/C	T/T	Affx-110224339	7B	54779941
AX-109380410	A/A	G/G	A/A	Affx-109069103	7B	549873695
AX-109388787	C/G	C/G	G/G	Affx-110659447	7B	384029864
AX-109389033	G/G	A/G	G/G	Affx-111966647	7B	641449375
AX-109390460	G/G	G/G	G/G	Affx-111017200	7B	75898525
AX-109396027	C/C	T/C	C/C	Affx-111128884	7B	322968652
AX-109396993	G/G	A/G	G/G	Affx-111680572	7B	128442529
AX-109404604	G/G	A/G	A/A	Affx-109557578	7B	654017213
AX-109410681	A/A	A/G	A/A	Affx-110632725	7B	505475180
AX-109412014	G/G	A/A	G/G	Affx-109059810	7B	323459176
AX-109412137	C/C	T/C	T/T	Affx-111913969	7B	6860330
AX-109412409	G/G	A/G	G/G	Affx-109022477	7B	595112905
AX-109414221	C/C	T/C	T/T	Affx-110162053	7B	525828498
AX-109422427	C/C	T/C	C/C	Affx-111104480	7B	605646070
AX-109431444	A/A	A/G	A/A	Affx-111502270	7B	24400402
AX-109431681	G/G	T/G	G/G	Affx-110681901	7B	182101208
AX-109433683	T/C	C/C	T/C	Affx-108929076	7B	625886692
AX-109435147	C/C	T/C	C/C	Affx-109289285	7B	182578877
AX-109435200	G/G	T/G	T/T	Affx-111261512	7B	83880584
AX-109439550	T/T	T/C	T/T	Affx-109559194	7B	331951363
AX-109440423	T/T	T/C	T/T	Affx-110359432	7B	709253035
AX-109448856	G/G	A/G	G/G	Affx-110701775	7B	490423102
AX-109450144	C/C	T/T	C/C	Affx-112002869	7B	394018200
AX-109450735	C/C	T/C	C/C	Affx-88712031	7B	605955822
AX-109452158	G/G	C/C	G/G	Affx-109703526	7B	625360910
AX-109460262	C/C	T/C	T/T	Affx-88664763	7B	131953517
AX-109477613	G/G	A/G	G/G	Affx-111535483	7B	190967728
AX-109478426	G/G	A/G	G/G	Affx-109861329	7B	100167828
AX-109480491	G/G	A/G	G/G	Affx-111142856	7B	129923891
AX-109486645	A/A	G/G	A/A	Affx-109833729	7B	119882839
AX-109486966	C/C	C/C	C/C	Affx-111316798	7B	50048677
AX-109495359	G/G	C/G	G/G	Affx-109762105	7B	640792753
AX-109510323	G/G	A/G	G/G	Affx-109029966	7B	734074055
AX-109512646	C/C	C/C	C/C	Affx-109296814	7B	148264571
AX-109513110	G/G	C/G	G/G	Affx-111299605	7B	631137606
AX-109523065	T/T	T/C	T/T	Affx-111744144	7B	34676493
AX-109553193	A/A	A/G	A/A	Affx-109191108	7B	506731675
AX-109554347	A/A	A/G	A/A	Affx-109697511	7B	495722476
AX-109577279	A/T	A/T	A/A	Affx-88342689	7B	726429311
AX-109586232	C/C	C/G	C/C	Affx-109629765	7B	550391006
AX-109610572	C/C	T/C	C/C	Affx-110381706	7B	610400865
AX-109632423	C/C	T/C	C/C	Affx-109128000	7B	171395211
AX-109649724	T/T	T/C	T/T	Affx-111662212	7B	597679203
AX-109653663	A/A	A/G	A/A	Affx-88437963	7B	133488955

AX-109817033	C/C	T/C	C/C	Affx-109308543	7B	711066385
AX-109826320	A/A	A/G	A/A	Affx-111926382	7B	577109756
AX-109826731	A/G	G/G	G/G	Affx-109943495	7B	69356017
AX-109844760	C/C	T/C	C/C	Affx-109722461	7B	644499830
AX-109849135	C/C	C/G	C/C	Affx-109284478	7B	621318393
AX-109850181	A/A	A/G	A/A	Affx-88642695	7B	457788083
AX-109854550	G/G	C/G	G/G	Affx-111499467	7B	560530528
AX-109857770	C/C	T/C	T/T	Affx-111001042	7B	336345556
AX-109861771	C/C	T/C	C/C	Affx-109979758	7B	539878315
AX-109862374	A/A	A/G	A/A	Affx-111909988	7B	113540956
AX-109862866	G/G	G/G	G/G	Affx-110518205	7B	347530723
AX-109865023	T/T	T/C	C/C	Affx-111228425	7B	84563486
AX-109868890	C/C	A/C	C/C	Affx-110834538	7B	48245051
AX-109872568	C/C	A/C	C/C	Affx-111252645	7B	124078283
AX-109877318	C/C	T/C	T/T	Affx-110500142	7B	9132666
AX-109879793	G/G	C/G	G/G	Affx-109945880	7B	496656626
AX-109881255	T/T	T/C	C/C	Affx-88554592	7B	40441870
AX-109882260	A/A	A/C	A/A	Affx-109931439	7B	639282693
AX-109882451	A/A	A/G	A/A	Affx-111704445	7B	47572017
AX-109885156	G/G	C/G	G/G	Affx-110257978	7B	555577131
AX-109886745	G/G	A/G	G/G	Affx-110419259	7B	596726391
AX-109890394	C/C	A/C	C/C	Affx-109976362	7B	446976626
AX-109895532	T/T	T/C	T/T	Affx-109752104	7B	597241491
AX-109896558	A/A	A/A	A/G	Affx-110151956	7B	237763475
AX-109897139	C/C	C/C	T/T	Affx-88547396	7B	7540964
AX-109897967	C/C	T/C	T/T	Affx-109009082	7B	458106210
AX-109908750	G/G	A/G	G/G	Affx-110567610	7B	135036998
AX-109909871	T/T	C/C	T/T	Affx-110444219	7B	155335873
AX-109910406	T/T	T/G	G/G	Affx-109898173	7B	153749501
AX-109922088	A/A	G/G	A/A	Affx-110454428	7B	686887157
AX-109925774	C/C	T/C	T/T	Affx-110892076	7B	41201260
AX-109927712	G/G	G/G	C/G	Affx-111261924	7B	452817308
AX-109930103	A/A	A/G	A/A	Affx-109372332	7B	242723609
AX-109932159	C/C	T/C	C/C	Affx-109163093	7B	502612173
AX-109933841	A/A	A/G	A/A	Affx-109422953	7B	553055012
AX-109939313	C/C	T/C	T/T	Affx-88428697	7B	699914993
AX-109940858	A/A	A/G	G/G	Affx-108854941	7B	60085702
AX-109941031	T/T	T/G	T/T	Affx-110571954	7B	160613849
AX-109942609	C/C	C/C	C/C	Affx-109150862	7B	607634644
AX-109943324	C/C	A/C	C/C	Affx-110991118	7B	156966639
AX-109948514	A/A	A/G	A/A	Affx-111041791	7B	570244567
AX-109949434	T/T	T/T	T/T	Affx-108892991	7B	139794130
AX-109951530	G/G	A/G	A/A	Affx-109601005	7B	57878528
AX-109954437	C/C	T/C	C/C	Affx-111095741	7B	463865971
AX-109955630	T/T	T/C	T/T	Affx-109489570	7B	99627616
AX-109964007	G/G	A/G	G/G	Affx-110210870	7B	630563984
AX-109975845	G/G	T/G	T/T	Affx-109656285	7B	1900089
AX-109977913	G/G	G/G	G/G	Affx-111114694	7B	488409390
AX-109978126	T/T	T/C	T/T	Affx-111459643	7B	491195957
AX-109985354	C/C	C/C	C/C	Affx-110090835	7B	158481702
AX-109986595	A/A	A/C	A/A	Affx-111700802	7B	600841004
AX-109988129	C/C	C/G	C/C	Affx-110307247	7B	537132954
AX-109990639	C/C	T/C	C/C	Affx-109387462	7B	475609768
AX-109997103	C/C	T/C	C/C	Affx-111553342	7B	601386005

AX-110004319	C/C	A/A	C/C	Affx-111736992	7B	616512792
AX-110006349	T/T	T/G	T/T	Affx-110239403	7B	631570631
AX-110006913	T/T	C/C	T/T	Affx-111169599	7B	716378192
AX-110009621	A/A	A/G	A/A	Affx-110469258	7B	716373353
AX-110009756	A/G	A/G	G/G	Affx-109376272	7B	522568743
AX-110027999	C/C	C/G	C/C	Affx-109266135	7B	723732430
AX-110028265	C/C	C/C	G/G	Affx-109269841	7B	41848257
AX-110028937	C/C	C/C	C/C	Affx-111137869	7B	707701717
AX-110029318	C/C	T/C	C/C	Affx-109570468	7B	331420649
AX-110031797	A/C	C/C	A/C	Affx-110961755	7B	60772068
AX-110032392	C/C	T/C	T/T	Affx-111294123	7B	124740578
AX-110040667	G/G	A/G	G/G	Affx-111412177	7B	551374169
AX-110057611	G/G	G/G	G/G	Affx-109256539	7B	739450212
AX-110059100	T/T	T/C	T/T	Affx-88758763	7B	637777510
AX-110061645	G/G	G/G	G/G	Affx-111724042	7B	489410587
AX-110063659	C/C	G/G	C/C	Affx-110502490	7B	35317645
AX-110066957	G/G	A/G	A/A	Affx-111723198	7B	329943564
AX-110080558	G/G	T/G	G/G	Affx-108957668	7B	535258496
AX-110080984	A/A	G/G	A/A	Affx-88408559	7B	602940428
AX-110087763	C/C	T/C	C/C	Affx-109031872	7B	12966274
AX-110093104	A/A	A/G	A/A	Affx-110413757	7B	44294484
AX-110098439	A/A	A/G	A/A	Affx-109607735	7B	550664262
AX-110128273	G/G	A/G	G/G	Affx-111018363	7B	568510759
AX-110128330	C/C	T/T	C/C	Affx-109884198	7B	162072959
AX-110130372	G/G	T/G	G/G	Affx-110683214	7B	616551732
AX-110130928	G/G	A/G	A/A	Affx-109834939	7B	376757699
AX-110132422	G/G	T/G	G/G	Affx-111781443	7B	9590277
AX-110140731	A/A	A/G	A/A	Affx-110201097	7B	593566048
AX-110200345	C/C	C/G	C/C	Affx-111963542	7B	497951385
AX-110360149	G/G	G/G	G/G	Affx-88568409	7B	640512560
AX-110362282	A/G	G/G	A/G	Affx-110807294	7B	524719555
AX-110363414	T/T	T/G	T/T	Affx-108928826	7B	518020822
AX-110364074	G/G	A/G	G/G	Affx-110103361	7B	686201853
AX-110368981	T/T	T/C	T/T	Affx-110093328	7B	211110746
AX-110371786	A/A	G/G	A/A	Affx-110292507	7B	581794310
AX-110372297	C/C	C/G	C/C	Affx-111212085	7B	539231114
AX-110373727	T/T	T/C	T/T	Affx-109377363	7B	571850020
AX-110374711	A/G	A/G	A/G	Affx-110261746	7B	111882074
AX-110376932	C/C	T/C	T/T	Affx-109605706	7B	504962219
AX-110380870	A/A	A/G	G/G	Affx-110855827	7B	278006801
AX-110383476	G/G	T/G	G/G	Affx-109056945	7B	438990784
AX-110389280	G/G	G/G	G/G	Affx-111024566	7B	321327804
AX-110395066	C/C	T/C	T/T	Affx-110301080	7B	53760546
AX-110403634	A/A	A/C	A/A	Affx-111813380	7B	632651354
AX-110405382	T/T	T/C	T/T	Affx-110979474	7B	544662112
AX-110405460	G/G	G/G	G/G	Affx-111017475	7B	557048165
AX-110405886	G/G	A/G	G/G	Affx-110344307	7B	45434802
AX-110410095	A/T	T/T	A/T	Affx-110464501	7B	655582688
AX-110412930	A/A	A/G	A/A	Affx-111457627	7B	643476079
AX-110414737	A/A	A/G	A/A	Affx-88519967	7B	119734760
AX-110416968	A/A	A/G	A/A	Affx-111914357	7B	334798001
AX-110421197	T/C	T/C	C/C	Affx-109838775	7B	177642999
AX-110421209	G/G	A/G	G/G	Affx-111274349	7B	113194012
AX-110423975	G/G	C/G	G/G	Affx-88795732	7B	602942895

AX-110429478	C/C	A/C	C/C	Affx-109252376	7B	638459542
AX-110441038	C/C	A/C	C/C	Affx-111413085	7B	649288844
AX-110444620	C/C	T/C	C/C	Affx-111571876	7B	9634844
AX-110444871	G/G	G/G	G/G	Affx-111387796	7B	150239322
AX-110446175	G/G	G/G	C/C	Affx-109213581	7B	154278614
AX-110448414	G/G	G/G	G/G	Affx-88792410	7B	544110137
AX-110448553	A/A	A/C	A/A	Affx-111358805	7B	553431449
AX-110453383	G/G	A/A	A/A	Affx-109864177	7B	80634430
AX-110453581	T/T	T/C	T/T	Affx-111357120	7B	336663268
AX-110456513	G/G	A/G	A/A	Affx-110824576	7B	64724052
AX-110465628	C/C	C/G	C/C	Affx-109365820	7B	543580337
AX-110476719	T/T	T/C	T/T	Affx-110666769	7B	712889277
AX-110483168	G/G	T/T	G/G	Affx-111850194	7B	498655099
AX-110484893	G/G	A/G	A/A	Affx-109783706	7B	504658052
AX-110485000	T/T	T/T	C/C	Affx-111723869	7B	63625853
AX-110485151	G/G	A/G	G/G	Affx-111889177	7B	654947377
AX-110490694	G/G	T/G	G/G	Affx-110067654	7B	612331547
AX-110492506	T/T	T/C	T/T	Affx-109951728	7B	594044623
AX-110493963	A/A	A/G	G/G	Affx-109748418	7B	739426317
AX-110502367	G/G	A/G	G/G	Affx-88745898	7B	100334504
AX-110502532	C/C	C/G	G/G	Affx-110832924	7B	739453420
AX-110507674	A/A	A/G	A/A	Affx-109164324	7B	562362432
AX-110511335	G/G	G/G	G/G	Affx-111354150	7B	234814445
AX-110530584	C/C	T/T	T/T	Affx-110653333	7B	144269602
AX-110531494	C/C	C/G	C/C	Affx-111347885	7B	99486267
AX-110534723	A/A	A/G	G/G	Affx-111591780	7B	521592642
AX-110537865	C/C	C/C	C/C	Affx-109622367	7B	615720206
AX-110552192	A/A	A/C	A/A	Affx-109122550	7B	708121130
AX-110558393	G/G	C/G	G/G	Affx-108891831	7B	503154163
AX-110560654	C/C	A/C	C/C	Affx-111232533	7B	629481235
AX-110563962	A/A	C/C	A/A	Affx-111353317	7B	650126448
AX-110574606	T/T	T/C	T/T	Affx-111963589	7B	192874937
AX-110585187	T/T	T/C	T/T	Affx-88559222	7B	34496693
AX-110585364	C/C	T/C	T/T	Affx-109976995	7B	529671933
AX-110587080	T/T	G/G	T/T	Affx-111564402	7B	652330352
AX-110599607	A/A	A/G	A/A	Affx-109378273	7B	602435973
AX-110602819	A/G	G/G	A/G	Affx-109297386	7B	563781905
AX-110602843	C/C	T/C	T/T	Affx-111804497	7B	451488111
AX-110608003	T/T	T/C	T/T	Affx-111347662	7B	519192491
AX-110611183	G/G	T/G	G/G	Affx-110954266	7B	355426093
AX-110632956	G/G	G/G	G/G	Affx-109490435	7B	594956479
AX-110632991	T/T	T/T	C/C	Affx-110867342	7B	483533955
AX-110634096	C/C	T/C	C/C	Affx-110146726	7B	3699874
AX-110642110	G/G	G/G	G/G	Affx-109136735	7B	118891334
AX-110648188	A/A	G/G	A/A	Affx-111655279	7B	637227520
AX-110670642	A/A	A/C	C/C	Affx-111811986	7B	8429297
AX-110687704	C/C	T/T	C/C	Affx-110192712	7B	38485827
AX-110743743	C/C	C/C	C/C	Affx-110105548	7B	562659792
AX-110905186	T/C	T/C	C/C	Affx-108947466	7B	677124216
AX-110912865	C/C	T/C	C/C	Affx-110220949	7B	166784193
AX-110912924	G/G	A/G	G/G	Affx-110938448	7B	18744325
AX-110919533	T/T	T/C	T/T	Affx-111144930	7B	329356598
AX-110920095	G/G	T/G	G/G	Affx-88608445	7B	341826996
AX-110922928	G/G	C/G	G/G	Affx-110144508	7B	167792166

AX-110923737	C/C	T/C	C/C	Affx-110503904	7B	492266353
AX-110939373	C/C	T/C	C/C	Affx-111882957	7B	525884266
AX-110945508	G/G	A/A	A/A	Affx-111847686	7B	93872079
AX-110947282	C/C	C/C	C/C	Affx-109381716	7B	496057552
AX-110950600	G/G	C/G	G/G	Affx-109161498	7B	534699179
AX-110950756	G/G	A/G	G/G	Affx-109814703	7B	15528284
AX-110953411	G/G	A/G	G/G	Affx-110450680	7B	5933087
AX-110954191	G/G	A/G	G/G	Affx-109000691	7B	587217
AX-110954560	T/T	T/C	C/C	Affx-109626634	7B	726567854
AX-110955130	C/C	A/C	C/C	Affx-110086196	7B	743303699
AX-110957024	T/T	T/C	T/T	Affx-109804480	7B	272111901
AX-110959488	T/T	T/C	T/T	Affx-110772552	7B	634229031
AX-110961784	G/G	G/G	G/G	Affx-109189330	7B	617851981
AX-110968189	T/T	T/C	T/T	Affx-109422267	7B	51586043
AX-110972260	T/T	T/G	T/T	Affx-111833962	7B	593070004
AX-110973356	G/G	A/G	A/A	Affx-110416936	7B	64180427
AX-110974083	G/G	A/G	G/G	Affx-110859615	7B	628463741
AX-110985656	G/G	C/G	G/G	Affx-111195766	7B	237762780
AX-110997141	G/G	A/G	G/G	Affx-108886320	7B	70713
AX-110997335	C/C	A/C	C/C	Affx-109430031	7B	147643495
AX-111000584	A/A	A/C	A/C	Affx-88636546	7B	715289272
AX-111001043	C/C	C/C	A/A	Affx-110445375	7B	687335236
AX-111005194	T/T	T/C	T/T	Affx-109848968	7B	636386093
AX-111012930	C/C	G/G	C/C	Affx-111560204	7B	144703486
AX-111013927	T/T	T/C	T/T	Affx-110297232	7B	682199428
AX-111015153	T/T	T/G	T/T	Affx-109065098	7B	689924794
AX-111015378	G/G	G/G	G/G	Affx-109033388	7B	44890180
AX-111026281	C/C	T/T	C/C	Affx-110092769	7B	498289076
AX-111026973	C/C	T/C	C/C	Affx-111797998	7B	135576110
AX-111030618	A/A	A/G	A/A	Affx-109427145	7B	706706666
AX-111032340	G/G	C/G	G/G	Affx-111054404	7B	53244819
AX-111032744	C/C	C/G	C/C	Affx-111253937	7B	591469018
AX-111036549	T/T	T/C	T/T	Affx-109426366	7B	572298519
AX-111045778	A/A	A/C	A/A	Affx-110013320	7B	484627247
AX-111048042	A/A	A/G	A/A	Affx-110737715	7B	630090482
AX-111050739	T/T	T/T	T/T	Affx-111705525	7B	513245811
AX-111058255	C/C	C/C	C/C	Affx-109181945	7B	599852172
AX-111063191	G/G	A/A	A/A	Affx-111143360	7B	85018815
AX-111067788	A/A	A/A	A/A	Affx-110289499	7B	484399370
AX-111068627	A/A	A/T	T/T	Affx-111392639	7B	521181164
AX-111069410	C/C	T/C	C/C	Affx-109884886	7B	68332958
AX-111069856	A/A	A/A	A/A	Affx-110241795	7B	549350406
AX-111072177	G/G	A/G	G/G	Affx-111269893	7B	156416844
AX-111073795	C/C	T/C	C/C	Affx-111249806	7B	46342044
AX-111074708	T/T	T/C	T/T	Affx-109022870	7B	134525510
AX-111082928	C/C	T/C	C/C	Affx-110879844	7B	490952763
AX-111083490	C/C	T/C	T/T	Affx-109718859	7B	42246044
AX-111085159	A/A	A/G	A/A	Affx-111948249	7B	481050974
AX-111096214	C/C	T/C	T/T	Affx-109756196	7B	74386639
AX-111096670	A/A	A/T	A/A	Affx-111843341	7B	89909867
AX-111098900	C/C	T/C	T/T	Affx-109709652	7B	187445806
AX-111102830	G/G	A/G	G/G	Affx-110137446	7B	691544860
AX-111108626	G/G	T/G	T/T	Affx-109196093	7B	164139326
AX-111121579	C/C	A/A	C/C	Affx-109734492	7B	554046058

AX-111126981	T/T	T/C	T/T	Affx-111506720	7B	520525930
AX-111127063	C/C	C/G	C/C	Affx-88564327	7B	493007896
AX-111134232	C/C	C/C	T/C	Affx-110989222	7B	109035230
AX-111134462	G/G	T/G	G/G	Affx-109392046	7B	512581824
AX-111136292	C/C	C/C	C/C	Affx-109252608	7B	557482908
AX-111137553	G/G	G/G	G/G	Affx-110075986	7B	605450530
AX-111139558	T/T	T/C	T/T	Affx-111862429	7B	614281773
AX-111141232	C/C	T/C	C/C	Affx-109839554	7B	205190157
AX-111143877	G/G	A/G	G/G	Affx-109226339	7B	706126603
AX-111147449	T/T	T/C	C/C	Affx-109502459	7B	714535142
AX-111153290	T/T	T/T	T/T	Affx-110353397	7B	52873290
AX-111190466	A/A	A/G	G/G	Affx-110504914	7B	14874652
AX-111198864	T/T	A/A	A/T	Affx-111919011	7B	739933197
AX-111218079	A/A	A/A	A/A	Affx-110284081	7B	51145115
AX-111219440	C/C	C/C	C/C	Affx-108881771	7B	453145778
AX-111232747	T/T	T/G	T/T	Affx-110351551	7B	334486014
AX-111247212	C/C	T/C	C/C	Affx-109282751	7B	649861001
AX-111265166	T/T	T/C	T/T	Affx-110097883	7B	621909831
AX-111280522	G/G	A/G	A/A	Affx-110081506	7B	130477199
AX-111285174	T/T	C/C	C/C	Affx-88346403	7B	522961079
AX-111286654	A/A	A/G	A/A	Affx-109721542	7B	559687793
AX-111290098	A/A	A/C	A/A	Affx-110519634	7B	689331124
AX-111450149	C/C	C/G	G/G	Affx-111772542	7B	69657558
AX-111450645	G/G	A/G	G/G	Affx-110357630	7B	2481009
AX-111451820	C/C	T/C	T/C	Affx-110678385	7B	223179894
AX-111453332	G/G	A/G	G/G	Affx-109267088	7B	136614993
AX-111458552	G/G	C/G	C/C	Affx-110839174	7B	40191951
AX-111461366	C/C	C/G	C/C	Affx-111549795	7B	115244932
AX-111461745	T/T	T/C	T/T	Affx-111136012	7B	700905584
AX-111463389	A/A	A/C	A/A	Affx-110051339	7B	645426085
AX-111473499	G/G	A/G	G/G	Affx-111440716	7B	580674169
AX-111487127	T/T	T/C	T/T	Affx-111266804	7B	747317254
AX-111491702	T/T	T/G	T/T	Affx-109248115	7B	489812908
AX-111493436	C/C	C/G	C/C	Affx-109980906	7B	717336091
AX-111507939	G/G	T/G	T/T	Affx-110644609	7B	55772582
AX-111509122	C/C	T/C	T/T	Affx-111958007	7B	693158185
AX-111513470	T/T	T/C	T/T	Affx-88462354	7B	547182940
AX-111521662	G/G	G/G	G/G	Affx-109953515	7B	137788944
AX-111528965	G/G	A/G	G/G	Affx-109583040	7B	9630896
AX-111531611	C/C	T/C	C/C	Affx-111275119	7B	561277280
AX-111535289	G/G	A/G	G/G	Affx-111756835	7B	6257146
AX-111537015	A/C	A/C	C/C	Affx-109160347	7B	667874340
AX-111537305	G/G	A/G	G/G	Affx-109458486	7B	533615334
AX-111538295	T/C	T/T	T/C	Affx-111645433	7B	656189292
AX-111547060	G/G	C/G	G/G	Affx-88612561	7B	541977545
AX-111553643	G/G	A/G	G/G	Affx-88603002	7B	611555477
AX-111554561	G/G	A/G	A/A	Affx-110996024	7B	740039841
AX-111555191	A/A	G/G	G/G	Affx-110942559	7B	8929550
AX-111555575	T/T	T/C	C/C	Affx-110473911	7B	231429954
AX-111564267	G/G	T/G	G/G	Affx-111075244	7B	543503541
AX-111565156	C/C	C/G	C/C	Affx-109930915	7B	128770131
AX-111571387	G/G	C/G	G/G	Affx-110747355	7B	552546165
AX-111571415	T/T	T/C	T/T	Affx-111418114	7B	66880709
AX-111573648	C/C	C/C	C/C	Affx-88737771	7B	188396445

AX-111574714	C/C	T/C	C/C	Affx-111337233	7B	124544385
AX-111576402	A/A	A/G	A/A	Affx-88753987	7B	541167701
AX-111582495	G/G	G/G	G/G	Affx-111404795	7B	747200211
AX-111588859	A/A	A/G	A/A	Affx-88436849	7B	190066222
AX-111590325	C/C	T/C	C/C	Affx-110129855	7B	162671570
AX-111601655	G/G	A/G	G/G	Affx-111764458	7B	351449986
AX-111601980	T/T	T/T	T/T	Affx-109749896	7B	582276177
AX-111608660	A/A	A/G	A/A	Affx-110124831	7B	142291056
AX-111615106	T/T	T/G	T/T	Affx-109533776	7B	152780660
AX-111630680	C/C	T/C	C/C	Affx-110685179	7B	719069692
AX-111635368	G/G	C/G	G/G	Affx-111569527	7B	125866490
AX-111640633	T/T	T/C	C/C	Affx-110622494	7B	55382261
AX-111642939	T/T	T/C	T/C	Affx-111830211	7B	42752823
AX-111643610	C/C	C/C	T/C	Affx-109660432	7B	449938218
AX-111644539	G/G	A/A	G/G	Affx-109769643	7B	610583816
AX-111650139	G/G	A/G	G/G	Affx-110869517	7B	12587548
AX-111654188	G/G	A/G	G/G	Affx-111538678	7B	669011083
AX-111658328	G/G	G/G	G/G	Affx-110765860	7B	39693818
AX-111659576	C/C	C/C	C/C	Affx-111417725	7B	133251429
AX-111661843	C/C	T/C	T/T	Affx-111979802	7B	731014652
AX-111661866	C/C	A/C	C/C	Affx-111404628	7B	237510511
AX-111662774	C/C	T/C	T/T	Affx-109553143	7B	168366606
AX-111666202	G/G	T/G	G/G	Affx-110141444	7B	230243183
AX-111671210	C/C	T/C	C/C	Affx-111941762	7B	592907066
AX-111671754	T/T	T/C	C/C	Affx-109353375	7B	364339203
AX-111681392	C/C	C/G	G/G	Affx-110870868	7B	79385471
AX-111687988	G/G	A/A	G/G	Affx-111781658	7B	330383143
AX-111690206	G/G	G/G	G/G	Affx-110654986	7B	454231498
AX-111690659	G/G	A/A	G/G	Affx-109039949	7B	136049502
AX-111696319	C/C	C/C	C/C	Affx-111520976	7B	485455658
AX-111701721	G/G	A/G	G/G	Affx-110180274	7B	280326894
AX-111712510	G/G	A/G	G/G	Affx-109459511	7B	705737251
AX-111720877	G/G	T/G	G/G	Affx-88576675	7B	703302360
AX-111758108	C/C	T/C	C/C	Affx-111909139	7B	722089981
AX-111761158	G/G	C/G	G/G	Affx-110368007	7B	657087464
AX-111779220	G/G	G/G	G/G	Affx-109393451	7B	518562852
AX-111780551	G/G	T/G	T/T	Affx-111523859	7B	478187518
AX-111797401	T/T	T/C	T/T	Affx-111963213	7B	580986785
AX-182014906	G/G	T/G	G/G	Affx-109349239	7B	237472039
AX-182047716	A/G	A/A	A/G	Affx-472285523	7B	488704566
AX-182061388	A/A	A/A	A/A	Affx-472306568	7B	395064125
AX-182067947	T/T	C/C	C/C	Affx-472316631	7B	289510857
AX-182073863	C/C	T/C	C/C	Affx-88430220	7B	739442084
AX-182104407	A/C	A/C	A/C	Affx-109847870	7B	128774784
AX-182149717	T/C	C/C	T/C	Affx-472297875	7B	744110463
AX-182152230	A/C	A/C	A/C	Affx-472300650	7B	541700515
AX-182169403	T/C	T/C	C/C	Affx-472314211	7B	389110565
AX-182181911	G/G	G/G	G/G	Affx-88586206	7B	24060528
AX-86165715	T/T	T/C	C/C	Affx-88627654	7B	109041902
AX-86169708	T/T	T/C	C/C	Affx-88404198	7B	739441934
AX-89344928	A/A	A/G	A/A	Affx-88376616	7B	601929203
AX-89364680	C/C	T/C	T/T	Affx-88396795	7B	692933721
AX-89382356	A/A	A/G	A/A	Affx-88414760	7B	708558138
AX-89432708	A/A	A/G	A/A	Affx-88465409	7B	701338884

AX-89434461	A/C	C/C	A/C	Affx-88467166	7B	653889472
AX-89451415	T/C	C/C	T/C	Affx-88484176	7B	709198270
AX-89528054	C/C	T/C	C/C	Affx-88560994	7B	712513981
AX-89576039	T/C	T/T	T/T	Affx-88609120	7B	1261396
AX-89658728	C/C	T/C	C/C	Affx-88691519	7B	721217470
AX-89740247	G/G	A/G	A/A	Affx-88772937	7B	3146812
AX-89749267	G/G	T/G	G/G	Affx-110805445	7B	85782674
AX-94397806	A/A	A/C	A/A	Affx-92253684	7B	657662537
AX-94416903	G/G	G/G	G/G	Affx-92502200	7B	651482078
AX-94418646	T/T	T/T	T/T	Affx-88743270	7B	426881224
AX-94426964	A/A	A/G	A/A	Affx-92728442	7B	27346800
AX-94431141	T/C	T/C	T/T	Affx-92814004	7B	474409091
AX-94438488	T/C	T/T	T/C	Affx-92096975	7B	647761792
AX-94498565	T/T	T/C	T/T	Affx-92963388	7B	440004871
AX-94498693	G/G	G/G	G/G	Affx-92890519	7B	210334471
AX-94502191	G/G	T/T	T/T	Affx-92231262	7B	678635351
AX-94515244	C/C	C/C	C/C	Affx-92892429	7B	210332300
AX-94516502	A/A	A/G	A/A	Affx-92566765	7B	21186227
AX-94525975	A/T	A/T	A/A	Affx-92537370	7B	660236021
AX-94527140	T/T	T/T	T/C	Affx-92404568	7B	452747475
AX-94532247	C/C	C/G	G/G	Affx-92871497	7B	524619772
AX-94545252	C/C	C/G	C/C	Affx-92406470	7B	133792799
AX-94620215	C/C	C/C	C/C	Affx-88526219	7B	645124763
AX-94653737	A/A	A/G	A/A	Affx-92607468	7B	337637165
AX-94657855	A/G	A/G	A/A	Affx-92264880	7B	674062954
AX-94670534	G/G	T/G	T/T	Affx-92368363	7B	234905885
AX-94714203	C/C	C/G	C/C	Affx-92865437	7B	483233601
AX-94733234	T/T	T/C	T/T	Affx-92961279	7B	678029900
AX-94778315	G/G	G/G	A/G	Affx-92761863	7B	587914953
AX-94782174	T/T	T/T	T/T	Affx-92619638	7B	684458037
AX-94787782	T/T	T/C	T/C	Affx-92351540	7B	1258395
AX-94829027	T/T	T/G	T/T	Affx-92117233	7B	744258498
AX-94977792	T/T	T/C	T/T	Affx-92755947	7B	711357268
AX-95015148	C/C	C/C	C/C	Affx-92817639	7B	237490792
AX-95020278	G/G	A/A	G/G	Affx-112314612	7B	3689322
AX-95021317	A/A	A/G	A/A	Affx-92788396	7B	647758189
AX-95152493	G/G	G/G	G/G	Affx-92225698	7B	608364832
AX-95164074	G/G	A/G	G/G	Affx-88451286	7B	9754433
AX-95194281	T/C	C/C	C/C	Affx-88461643	7B	83030077
AX-95197649	C/G	G/G	C/G	Affx-92561541	7B	4910078
AX-95214723	C/C	T/C	C/C	Affx-92765422	7B	436211110
AX-95215234	C/C	C/C	T/T	Affx-92831673	7B	662674605
AX-95222495	T/T	T/T	T/T	Affx-92700094	7B	709515058
AX-95226841	A/A	A/G	A/A	Affx-92485587	7B	744404878
AX-95241763	G/G	G/G	G/G	Affx-92675166	7B	237490093
AX-95248046	T/T	C/C	C/C	Affx-92301332	7B	186815399
AX-95632174	T/T	T/C	C/C	Affx-88417566	7B	680171148
AX-95651698	A/A	A/G	A/A	Affx-92982813	7B	705734663
AX-95653151	T/T	T/C	T/T	Affx-88649025	7B	21668998
AX-95658405	T/T	T/T	T/T	Affx-111364722	7B	699380633
AX-95659455	T/T	C/C	T/T	Affx-88345461	7B	744404849
AX-108727432	A/A	A/A	A/A	Affx-111724206	7D	633538199
AX-108734645	T/T	T/C	T/T	Affx-109090580	7D	113754782
AX-108738816	C/C	T/C	C/C	Affx-88524456	7D	368026614

AX-108739145	C/C	T/C	C/C	Affx-109334012	7D	351629965
AX-108753131	G/G	A/G	G/G	Affx-110816333	7D	534438488
AX-108753528	C/C	C/G	C/C	Affx-111676406	7D	32022244
AX-108758043	G/G	A/G	G/G	Affx-110492632	7D	547277450
AX-108759691	A/A	A/C	C/C	Affx-88804075	7D	28828614
AX-108763519	G/G	C/G	G/G	Affx-108868802	7D	572307546
AX-108770812	C/C	T/C	C/C	Affx-110780663	7D	561926335
AX-108771397	C/C	A/C	C/C	Affx-109460205	7D	483641598
AX-108775641	C/C	T/C	C/C	Affx-110906500	7D	276619956
AX-108789692	T/T	T/C	T/T	Affx-111993918	7D	523816912
AX-108797441	A/A	A/G	A/A	Affx-108928574	7D	274385247
AX-108814055	G/G	C/G	C/C	Affx-110715543	7D	212325041
AX-108815963	C/C	T/C	C/C	Affx-110110840	7D	577348898
AX-108818991	G/G	A/G	G/G	Affx-110980389	7D	161193787
AX-108826091	C/C	T/C	T/T	Affx-88601309	7D	40205746
AX-108834234	A/A	A/G	A/A	Affx-109721067	7D	228067937
AX-108834910	C/C	A/C	C/C	Affx-109608761	7D	617295905
AX-108838800	A/A	A/C	A/A	Affx-111133361	7D	524655107
AX-108844280	G/G	A/G	G/G	Affx-109038703	7D	120765843
AX-108848503	T/T	T/C	T/T	Affx-88471288	7D	529773089
AX-108859395	C/C	T/C	C/C	Affx-111773793	7D	469523704
AX-108859931	A/A	A/C	C/C	Affx-111752723	7D	19283721
AX-108864773	T/T	G/G	T/T	Affx-109179354	7D	610227213
AX-108872250	G/G	A/G	G/G	Affx-111347764	7D	344519276
AX-108872289	T/T	T/C	T/T	Affx-88466679	7D	72784828
AX-108875138	T/T	C/C	T/T	Affx-111781401	7D	112949752
AX-108879159	G/G	A/A	G/G	Affx-109802847	7D	397573909
AX-108890548	C/C	A/C	C/C	Affx-109028418	7D	51101289
AX-108894371	C/C	T/C	C/C	Affx-109562563	7D	184207924
AX-108895252	A/A	A/G	A/A	Affx-110577885	7D	122838225
AX-108902254	A/A	A/G	A/A	Affx-110405337	7D	465342584
AX-108902552	A/A	A/G	G/G	Affx-110794261	7D	241175921
AX-108904493	G/G	T/G	G/G	Affx-109044722	7D	486305641
AX-108908338	C/C	T/C	C/C	Affx-88568385	7D	8279359
AX-108917680	C/C	T/C	C/C	Affx-111731889	7D	106856353
AX-108920250	C/C	C/C	C/C	Affx-109471786	7D	54995342
AX-108920521	C/C	T/C	C/C	Affx-111499199	7D	565192679
AX-108922234	G/G	C/G	G/G	Affx-110689233	7D	526918058
AX-108931208	G/G	A/G	G/G	Affx-110003561	7D	293519910
AX-108933675	A/A	A/A	A/A	Affx-108990639	7D	309758149
AX-108934048	C/C	A/C	C/C	Affx-88467637	7D	317663434
AX-108934118	C/C	T/C	C/C	Affx-111638518	7D	269718810
AX-108934161	C/C	T/C	T/T	Affx-111595655	7D	242638493
AX-108938180	A/A	A/G	A/A	Affx-109785099	7D	276383475
AX-108938253	T/T	T/C	T/T	Affx-111499009	7D	149081034
AX-108942474	A/A	A/G	A/A	Affx-110222690	7D	529344748
AX-108947602	C/C	A/C	C/C	Affx-109793493	7D	51108343
AX-108948218	A/A	A/G	A/A	Affx-88806261	7D	15102252
AX-108948630	T/T	C/C	C/C	Affx-109931410	7D	27349728
AX-108955303	C/C	T/C	C/C	Affx-110592981	7D	499836798
AX-108975916	G/G	A/G	G/G	Affx-110053595	7D	513348634
AX-108978664	C/C	A/C	C/C	Affx-88426561	7D	303311869
AX-108978807	A/A	A/G	G/G	Affx-111951194	7D	24022589
AX-108991830	T/T	C/C	T/T	Affx-109309557	7D	556721941

AX-109005416	G/G	C/G	G/G	Affx-111292079	7D	51433803
AX-109010857	A/A	A/G	A/A	Affx-111689428	7D	189914442
AX-109030892	C/C	T/C	C/C	Affx-110974917	7D	424134599
AX-109035303	C/C	T/C	C/C	Affx-110613850	7D	188187300
AX-109075673	C/C	C/G	C/C	Affx-109498981	7D	522006428
AX-109112266	C/C	T/C	C/C	Affx-111330170	7D	459019918
AX-109113600	G/G	T/G	G/G	Affx-110499018	7D	555188205
AX-109122450	A/A	A/C	A/A	Affx-109322323	7D	419809323
AX-109123458	T/T	T/T	T/T	Affx-109042058	7D	523044460
AX-109130875	C/C	T/C	T/T	Affx-111740229	7D	98368435
AX-109157025	G/G	A/G	G/G	Affx-108961698	7D	344540496
AX-109160430	C/C	A/C	C/C	Affx-110152331	7D	455060262
AX-109171361	G/G	C/G	G/G	Affx-110311780	7D	139826159
AX-109178084	C/C	T/C	C/C	Affx-88642596	7D	431964240
AX-109178337	C/C	C/C	C/C	Affx-109741210	7D	363802864
AX-109180375	A/A	A/G	G/G	Affx-110160508	7D	249486123
AX-109189009	C/C	A/C	A/A	Affx-109341310	7D	593701314
AX-109190061	T/T	T/C	T/T	Affx-109288089	7D	340435710
AX-109192014	T/T	T/C	T/T	Affx-108890292	7D	60977272
AX-109192025	C/C	C/C	C/C	Affx-110161139	7D	330665730
AX-109194960	G/G	A/G	G/G	Affx-88707289	7D	419762439
AX-109195214	C/C	T/C	C/C	Affx-108979230	7D	437913303
AX-109199910	G/G	A/G	G/G	Affx-108879265	7D	500965637
AX-109207027	A/A	A/G	A/A	Affx-110223125	7D	586437729
AX-109247018	C/C	T/C	C/C	Affx-109445078	7D	495564808
AX-109260205	G/G	A/G	G/G	Affx-110863061	7D	273940971
AX-109264828	C/C	A/C	C/C	Affx-88793362	7D	554244756
AX-109270431	T/T	A/T	T/T	Affx-109018563	7D	104730268
AX-109270581	G/G	G/G	G/G	Affx-109618662	7D	476968368
AX-109272506	G/G	A/A	G/G	Affx-88574066	7D	386727016
AX-109278070	A/A	A/G	A/A	Affx-109435376	7D	181058073
AX-109279751	G/G	A/G	G/G	Affx-111536158	7D	349365912
AX-109284403	A/A	A/G	A/A	Affx-111939857	7D	400865662
AX-109284685	G/G	A/G	G/G	Affx-109288904	7D	412652160
AX-109287932	G/G	C/G	G/G	Affx-111659227	7D	570304273
AX-109302964	G/G	A/G	G/G	Affx-109623554	7D	585871745
AX-109307591	T/T	T/C	T/T	Affx-111621526	7D	179774353
AX-109318566	G/G	T/G	G/G	Affx-88489455	7D	394236920
AX-109321084	G/G	A/G	G/G	Affx-111412644	7D	327108368
AX-109326192	C/C	C/C	C/C	Affx-110264392	7D	100759755
AX-109328587	G/G	A/G	G/G	Affx-111290221	7D	6483856
AX-109331077	G/G	A/G	G/G	Affx-109938518	7D	442169256
AX-109332089	T/T	T/C	T/T	Affx-88741168	7D	187434448
AX-109333696	C/C	T/C	C/C	Affx-109849341	7D	294492674
AX-109345237	G/G	T/G	G/G	Affx-110139899	7D	203360272
AX-109345846	T/T	T/C	T/T	Affx-88614603	7D	178290046
AX-109358887	A/A	A/G	A/A	Affx-110667648	7D	552498818
AX-109359848	C/C	T/C	T/T	Affx-109472133	7D	23233202
AX-109368801	G/G	G/G	G/G	Affx-109321337	7D	378499959
AX-109374178	G/G	A/G	G/G	Affx-111872919	7D	148320932
AX-109379003	C/C	T/C	C/C	Affx-109111886	7D	358191875
AX-109384830	C/C	T/C	C/C	Affx-109201751	7D	161140809
AX-109390002	C/C	A/C	C/C	Affx-110972125	7D	99955788
AX-109390503	A/A	A/G	A/A	Affx-109799567	7D	564941775

AX-109396493	T/C	T/C	T/C	Affx-110902397	7D	225832408
AX-109396647	G/G	A/G	G/G	Affx-109639875	7D	371828157
AX-109396836	C/C	T/T	C/C	Affx-110286600	7D	530922075
AX-109398922	G/G	A/A	G/G	Affx-109223634	7D	138794578
AX-109402197	C/C	A/A	C/C	Affx-110647758	7D	35599515
AX-109411651	A/A	A/G	A/A	Affx-88683640	7D	536307583
AX-109416371	A/A	A/A	A/A	Affx-111952120	7D	219851388
AX-109418277	C/C	T/C	C/C	Affx-88374022	7D	312550282
AX-109420090	T/T	T/C	C/C	Affx-88520078	7D	89733983
AX-109426476	G/G	A/G	G/G	Affx-111337547	7D	441355535
AX-109430335	T/T	C/C	T/T	Affx-109063852	7D	315476303
AX-109432597	C/C	C/G	G/G	Affx-110326166	7D	102297853
AX-109434652	A/A	A/G	A/A	Affx-108953538	7D	592220423
AX-109464525	C/C	T/C	C/C	Affx-109108196	7D	322719746
AX-109469634	C/C	C/G	G/G	Affx-109453904	7D	247038359
AX-109477852	G/G	A/G	G/G	Affx-108887683	7D	7534268
AX-109482720	T/T	T/C	T/T	Affx-109227168	7D	27554508
AX-109483394	C/C	T/C	C/C	Affx-88695736	7D	337720040
AX-109485278	G/G	A/G	G/G	Affx-111103443	7D	605863195
AX-109488029	G/G	C/C	G/G	Affx-110446343	7D	513704834
AX-109490907	T/C	T/C	T/C	Affx-110890258	7D	566342804
AX-109496930	G/G	A/G	G/G	Affx-109369473	7D	386632215
AX-109497554	G/G	T/T	G/G	Affx-108872617	7D	14584380
AX-109504466	G/G	A/G	G/G	Affx-110716283	7D	38737717
AX-109514518	C/C	T/C	C/C	Affx-111480101	7D	303985064
AX-109517138	G/G	A/A	G/G	Affx-109915322	7D	297023142
AX-109518874	G/G	G/G	G/G	Affx-88530386	7D	517219684
AX-109526605	T/T	T/G	T/T	Affx-110325888	7D	518093101
AX-109531205	C/C	A/C	C/C	Affx-109832792	7D	572015293
AX-109541707	C/C	T/C	C/C	Affx-109794084	7D	538214362
AX-109543629	A/A	A/G	A/A	Affx-111659403	7D	388024474
AX-109551456	A/A	A/G	A/A	Affx-109359874	7D	505328180
AX-109569014	A/A	A/G	A/A	Affx-110052824	7D	611766722
AX-109577769	C/C	T/T	C/C	Affx-109609478	7D	10024324
AX-109580042	G/G	T/G	G/G	Affx-109523067	7D	316201825
AX-109580494	A/A	A/A	G/G	Affx-109141978	7D	251312653
AX-109583841	G/G	A/G	G/G	Affx-109671814	7D	283597173
AX-109584028	G/G	A/G	G/G	Affx-109758799	7D	379919367
AX-109590698	T/T	T/C	T/T	Affx-109750633	7D	573919490
AX-109592291	A/A	A/G	A/A	Affx-110157104	7D	613212737
AX-109609327	C/C	T/C	C/C	Affx-88780010	7D	510058158
AX-109627540	G/G	A/A	G/G	Affx-111950190	7D	123835396
AX-109640712	G/G	T/G	G/G	Affx-109946587	7D	359579308
AX-109656174	A/A	A/G	A/A	Affx-110729644	7D	202543585
AX-109657199	A/A	A/G	A/A	Affx-110586134	7D	422098230
AX-109660972	C/C	T/C	C/C	Affx-111094732	7D	405375400
AX-109668145	T/T	T/C	T/T	Affx-109651460	7D	51403695
AX-109670054	G/G	A/G	G/G	Affx-88745491	7D	101133815
AX-109686710	T/G	T/T	T/G	Affx-109187545	7D	359361395
AX-109695722	C/C	A/C	C/C	Affx-109238739	7D	461393928
AX-109707214	G/G	G/G	G/G	Affx-109714818	7D	354317191
AX-109723697	C/C	C/C	G/G	Affx-108886083	7D	208555487
AX-109724197	T/C	T/C	T/C	Affx-110377704	7D	58061642
AX-109728672	C/C	T/C	C/C	Affx-110324839	7D	109469429

AX-109731572	C/C	T/C	C/C	Affx-109333982	7D	53669596
AX-109739937	G/G	G/G	G/G	Affx-110310415	7D	292088420
AX-109777428	C/C	T/C	C/C	Affx-109955648	7D	331990742
AX-109780353	T/T	T/G	G/G	Affx-109055926	7D	93701787
AX-109790043	C/C	C/G	C/C	Affx-110370511	7D	528681357
AX-109791875	T/T	T/C	T/T	Affx-109841962	7D	458204666
AX-109808353	A/A	A/G	A/A	Affx-111928959	7D	559396464
AX-109835402	G/G	C/G	G/G	Affx-111986009	7D	631852152
AX-109843691	T/T	A/T	T/T	Affx-109901696	7D	50764553
AX-109847743	G/G	A/G	G/G	Affx-110429144	7D	355734382
AX-109847755	T/T	T/G	T/T	Affx-111582350	7D	145671548
AX-109851562	G/G	A/G	G/G	Affx-110166222	7D	360437986
AX-109853847	T/T	T/T	T/T	Affx-109387263	7D	543584208
AX-109855636	T/T	T/C	T/T	Affx-109671783	7D	99187403
AX-109856840	G/G	G/G	G/G	Affx-111510988	7D	5185888
AX-109857040	G/G	G/G	A/A	Affx-110207666	7D	47711832
AX-109860838	C/C	T/C	C/C	Affx-109512474	7D	40251201
AX-109861779	G/G	A/G	G/G	Affx-110157080	7D	519933125
AX-109865223	T/T	C/C	T/T	Affx-111844482	7D	6101832
AX-109866327	T/T	T/C	T/T	Affx-108983081	7D	61798325
AX-109867095	T/T	T/C	T/T	Affx-88695519	7D	577937328
AX-109868587	C/C	A/C	C/C	Affx-109936315	7D	260064496
AX-109869323	G/G	C/G	G/G	Affx-109063671	7D	364751427
AX-109870865	A/A	A/G	A/A	Affx-108880119	7D	191250576
AX-109883207	G/G	A/G	G/G	Affx-109122509	7D	474416298
AX-109891380	G/G	C/G	G/G	Affx-111780112	7D	109018739
AX-109895089	T/T	T/C	T/T	Affx-111426902	7D	104208874
AX-109903353	A/A	A/G	A/A	Affx-111956259	7D	467716039
AX-109904893	C/C	T/C	T/T	Affx-109123565	7D	80118308
AX-109905450	T/T	T/C	T/T	Affx-109231540	7D	305318970
AX-109905941	G/G	A/G	G/G	Affx-108960272	7D	182493965
AX-109917900	T/T	T/C	T/T	Affx-111289284	7D	192054373
AX-109920594	C/C	T/C	C/C	Affx-111297447	7D	253471003
AX-109922209	C/C	C/C	C/C	Affx-109956983	7D	391230651
AX-109927861	G/G	A/G	G/G	Affx-109018564	7D	36418870
AX-109931701	G/G	A/G	G/G	Affx-108964994	7D	464300742
AX-109932021	C/C	T/C	C/C	Affx-111413404	7D	494928829
AX-109936012	G/G	A/G	G/G	Affx-110110589	7D	433331776
AX-109937582	C/C	C/G	C/C	Affx-111994190	7D	553061932
AX-109942885	C/C	C/C	C/C	Affx-109303864	7D	123453469
AX-109945703	G/G	A/G	G/G	Affx-110752665	7D	241663683
AX-109949077	C/C	C/G	C/C	Affx-110733703	7D	164892887
AX-109951714	G/G	A/G	G/G	Affx-88367534	7D	446292326
AX-109953003	A/G	G/G	A/G	Affx-111707826	7D	603734338
AX-109953422	T/T	T/C	T/T	Affx-109259077	7D	511601534
AX-109953934	T/T	T/C	T/T	Affx-110235006	7D	525718333
AX-109955333	C/C	C/G	C/C	Affx-110034175	7D	73545031
AX-109965108	G/G	T/G	G/G	Affx-109502448	7D	604898892
AX-109972362	G/G	A/G	G/G	Affx-111731220	7D	311380137
AX-109988004	C/C	T/T	C/C	Affx-110193039	7D	350875022
AX-109998344	G/G	A/G	G/G	Affx-111925888	7D	516581179
AX-110001129	G/G	A/G	G/G	Affx-109075201	7D	254208315
AX-110013071	C/C	G/G	C/C	Affx-109187467	7D	610662850
AX-110019420	C/C	A/C	C/C	Affx-110037957	7D	564367347

AX-110019847	C/C	T/C	C/C	Affx-109636078	7D	260734546
AX-110021999	A/A	A/G	G/G	Affx-111634568	7D	228670666
AX-110029139	G/G	A/G	G/G	Affx-88731595	7D	464026081
AX-110033734	G/G	A/G	G/G	Affx-111279676	7D	470222624
AX-110044604	C/C	T/C	C/C	Affx-109014115	7D	636764352
AX-110045927	C/C	T/T	C/C	Affx-109803684	7D	393955572
AX-110047773	A/A	G/G	A/A	Affx-110186115	7D	5449877
AX-110047998	C/C	T/T	C/C	Affx-110283045	7D	154194269
AX-110052150	A/A	A/T	T/T	Affx-111470995	7D	71625991
AX-110052884	A/A	A/C	C/C	Affx-109910828	7D	84989874
AX-110059656	G/G	A/A	G/G	Affx-110481201	7D	377457704
AX-110066398	C/C	T/C	C/C	Affx-109649344	7D	311656884
AX-110078843	C/C	T/C	C/C	Affx-110400645	7D	378178093
AX-110081435	C/C	T/C	C/C	Affx-110595559	7D	20599369
AX-110082013	G/G	G/G	G/G	Affx-109251204	7D	401542892
AX-110084479	C/C	T/T	C/C	Affx-88644045	7D	549536522
AX-110097757	T/T	T/G	T/T	Affx-109532318	7D	299191143
AX-110121781	G/G	C/G	G/G	Affx-88476281	7D	526776624
AX-110136153	A/A	A/G	A/A	Affx-88669010	7D	559153552
AX-110146429	G/G	A/G	G/G	Affx-111735845	7D	366490711
AX-110146812	C/C	T/C	T/T	Affx-109329546	7D	244760778
AX-110174970	G/G	C/G	C/C	Affx-111981888	7D	24831080
AX-110183108	T/T	T/G	T/T	Affx-108914620	7D	392828782
AX-110200543	C/C	C/C	C/C	Affx-109435206	7D	404374290
AX-110201879	C/C	T/C	C/C	Affx-110668448	7D	175859355
AX-110231089	G/G	A/G	G/G	Affx-109996176	7D	109822213
AX-110240365	G/G	A/G	G/G	Affx-109294467	7D	348963768
AX-110245410	G/G	A/G	G/G	Affx-110297857	7D	320175344
AX-110251783	T/T	C/C	T/T	Affx-111037076	7D	213751994
AX-110254132	C/C	C/C	C/C	Affx-88553873	7D	227074119
AX-110258996	G/G	C/G	G/G	Affx-109150254	7D	370923350
AX-110271371	T/T	T/C	T/T	Affx-110176619	7D	93090818
AX-110275173	T/T	T/G	T/T	Affx-88539297	7D	15587559
AX-110281782	T/T	T/C	T/T	Affx-111098129	7D	136945404
AX-110287505	G/G	A/G	G/G	Affx-109971382	7D	248801166
AX-110289870	T/T	T/C	T/T	Affx-110877103	7D	405227637
AX-110292613	T/T	T/C	T/T	Affx-109990075	7D	608526855
AX-110296096	C/C	C/C	T/T	Affx-111911789	7D	35497309
AX-110334658	C/C	T/C	C/C	Affx-109956057	7D	336470780
AX-110338241	C/C	A/C	C/C	Affx-111900303	7D	567930035
AX-110354439	G/G	A/G	G/G	Affx-108912548	7D	300854195
AX-110363805	G/G	A/G	G/G	Affx-111306821	7D	56997324
AX-110376756	C/C	T/C	C/C	Affx-111044191	7D	305590880
AX-110385360	A/A	A/A	A/A	Affx-109569574	7D	267933033
AX-110386339	C/C	A/C	C/C	Affx-111188672	7D	464949314
AX-110398736	C/C	C/G	C/C	Affx-110096054	7D	523554117
AX-110403163	G/G	A/G	G/G	Affx-111220350	7D	264218947
AX-110411683	C/C	C/G	C/C	Affx-88357204	7D	520377192
AX-110415528	G/G	A/G	G/G	Affx-109775924	7D	318412761
AX-110427772	C/C	T/C	C/C	Affx-109089359	7D	502409867
AX-110442381	A/A	A/G	A/A	Affx-109789784	7D	138513896
AX-110446588	T/T	T/C	T/T	Affx-110681643	7D	615791419
AX-110456494	G/G	A/G	G/G	Affx-109382904	7D	572798505
AX-110457672	C/C	T/C	C/C	Affx-110617815	7D	342557267

AX-110460471	G/G	A/G	G/G	Affx-88361268	7D	405429164
AX-110467729	A/A	A/C	A/C	Affx-110282563	7D	43386933
AX-110474669	C/C	A/C	C/C	Affx-110438687	7D	392634823
AX-110475145	C/C	C/G	C/C	Affx-111408703	7D	415750021
AX-110480124	C/C	A/C	C/C	Affx-109774972	7D	636793186
AX-110481368	C/C	T/C	C/C	Affx-88532596	7D	291488731
AX-110483615	C/C	A/C	C/C	Affx-109954955	7D	353560181
AX-110489367	G/G	A/G	G/G	Affx-110550986	7D	283462339
AX-110497122	T/T	C/C	T/T	Affx-109465871	7D	554614049
AX-110498045	T/T	T/C	T/T	Affx-109874511	7D	570455731
AX-110499894	C/C	T/C	C/C	Affx-109993736	7D	403665346
AX-110501271	C/C	T/C	C/C	Affx-111383998	7D	366835241
AX-110502471	C/C	C/G	C/C	Affx-109112820	7D	54592977
AX-110503493	C/C	C/G	G/G	Affx-109461080	7D	40499497
AX-110503495	C/C	T/C	C/C	Affx-108885973	7D	367572887
AX-110508053	C/C	C/C	C/C	Affx-111957753	7D	455585611
AX-110509892	A/A	A/C	C/C	Affx-111970679	7D	239363239
AX-110510127	G/G	A/G	G/G	Affx-109064779	7D	491926954
AX-110510182	G/G	C/G	G/G	Affx-111632338	7D	389790631
AX-110510600	C/C	T/C	C/C	Affx-88643341	7D	314966929
AX-110514358	A/A	A/G	G/G	Affx-109599355	7D	250617324
AX-110516809	C/C	T/C	C/C	Affx-110305180	7D	526069740
AX-110516957	T/T	T/C	T/T	Affx-109133238	7D	430993309
AX-110525188	C/C	T/C	C/C	Affx-88583783	7D	400365776
AX-110529080	G/G	A/A	G/G	Affx-109917637	7D	32861344
AX-110532878	T/T	T/T	T/T	Affx-110873934	7D	301324476
AX-110541194	C/C	T/C	C/C	Affx-110693208	7D	337568928
AX-110555853	C/C	T/C	C/C	Affx-110211424	7D	525090029
AX-110585124	A/A	A/G	A/A	Affx-111098980	7D	173895553
AX-110588269	A/A	A/G	A/A	Affx-111398579	7D	611715311
AX-110605376	A/A	A/G	A/A	Affx-111374182	7D	561758308
AX-110611417	G/G	A/G	G/G	Affx-109930690	7D	102965992
AX-110613047	T/T	A/T	T/T	Affx-88365111	7D	552070147
AX-110648008	A/A	A/G	A/A	Affx-88609271	7D	527725404
AX-110666756	C/C	A/C	A/A	Affx-110504014	7D	245372112
AX-110667060	G/G	A/G	G/G	Affx-109281520	7D	395191226
AX-110667549	A/A	A/G	G/G	Affx-111003798	7D	91671945
AX-110676696	C/C	C/C	C/C	Affx-111126760	7D	556206935
AX-110702236	C/C	T/C	C/C	Affx-109646377	7D	372586989
AX-110712708	G/G	G/G	G/G	Affx-110559661	7D	491244594
AX-110717444	G/G	T/G	G/G	Affx-111547408	7D	246372934
AX-110718097	C/C	T/C	C/C	Affx-110914048	7D	276144417
AX-110748563	G/G	A/G	G/G	Affx-111439234	7D	8816762
AX-110750491	G/G	A/G	G/G	Affx-111022622	7D	189001615
AX-110767107	A/A	A/G	A/A	Affx-111262536	7D	201028390
AX-110773195	G/G	A/G	G/G	Affx-111193001	7D	129283409
AX-110777349	G/G	A/G	G/G	Affx-111868134	7D	425277770
AX-110788687	A/A	G/G	A/A	Affx-111409032	7D	469236625
AX-110804188	G/G	G/G	G/G	Affx-111471612	7D	373567295
AX-110809710	C/C	T/C	C/C	Affx-109082614	7D	342976821
AX-110811078	G/G	A/G	G/G	Affx-88657158	7D	551743816
AX-110826342	C/C	T/C	C/C	Affx-88435062	7D	480123390
AX-110830906	C/C	T/C	C/C	Affx-109027372	7D	280309034
AX-110841952	A/A	A/G	A/A	Affx-109703755	7D	286066895

AX-110888456	C/C	T/C	T/T	Affx-110166878	7D	41407631
AX-110893224	T/T	A/T	A/T	Affx-88804122	7D	31410924
AX-110907703	G/G	A/G	G/G	Affx-110790665	7D	447749769
AX-110908746	A/A	A/G	A/A	Affx-110416784	7D	139090813
AX-110910179	C/C	T/C	C/C	Affx-111760403	7D	103178967
AX-110913782	G/G	C/G	C/C	Affx-109490385	7D	25286841
AX-110913995	C/C	T/C	C/C	Affx-88485294	7D	428054768
AX-110915444	C/C	T/C	C/C	Affx-110733215	7D	267619865
AX-110920872	T/T	T/C	T/T	Affx-88432098	7D	72216966
AX-110923511	G/G	A/G	G/G	Affx-88805067	7D	403438211
AX-110924894	C/C	C/G	C/C	Affx-108969646	7D	84177937
AX-110928302	A/A	G/G	G/G	Affx-111244969	7D	256818338
AX-110928739	G/G	A/G	G/G	Affx-110144182	7D	322643182
AX-110929028	A/A	A/G	A/A	Affx-110796206	7D	368929884
AX-110929925	C/C	C/C	C/C	Affx-109663941	7D	74408039
AX-110934330	C/C	T/C	C/C	Affx-109580906	7D	168444849
AX-110938820	C/C	A/C	C/C	Affx-110067764	7D	318596344
AX-110949705	T/T	T/C	T/T	Affx-109561539	7D	548055156
AX-110955355	G/G	T/G	G/G	Affx-109423815	7D	609792380
AX-110962711	G/G	A/G	G/G	Affx-111634818	7D	365492954
AX-110967309	G/G	A/G	G/G	Affx-110771933	7D	433273817
AX-110967909	C/C	T/C	C/C	Affx-88416609	7D	449077562
AX-110972295	C/G	C/G	C/G	Affx-111065424	7D	479662000
AX-110974002	C/C	A/C	C/C	Affx-109897661	7D	591563891
AX-110974935	T/T	T/G	T/T	Affx-109002601	7D	10828241
AX-110981319	C/C	T/T	C/C	Affx-109912041	7D	60148964
AX-110982107	A/A	A/G	A/A	Affx-108892138	7D	623449316
AX-110983154	T/T	C/C	C/C	Affx-88569563	7D	590121412
AX-110990504	C/C	T/C	T/T	Affx-88682016	7D	92825155
AX-110991712	A/A	A/C	C/C	Affx-111184965	7D	40800789
AX-110993609	C/C	A/C	C/C	Affx-109118348	7D	277187622
AX-111000118	G/G	A/G	G/G	Affx-88377969	7D	498414557
AX-111003388	C/C	T/C	C/C	Affx-110311051	7D	223844284
AX-111003482	T/T	T/C	T/T	Affx-108988035	7D	618490790
AX-111011212	T/T	T/C	T/T	Affx-111207692	7D	103989082
AX-111014383	G/G	G/G	G/G	Affx-111760178	7D	203634696
AX-111022995	T/T	T/G	T/T	Affx-110307461	7D	167739780
AX-111023857	G/G	A/G	G/G	Affx-111531098	7D	349573875
AX-111024414	G/G	G/G	T/G	Affx-109332289	7D	498497704
AX-111026089	A/A	A/G	G/G	Affx-110218255	7D	246753196
AX-111030576	G/G	T/G	G/G	Affx-110426449	7D	299506827
AX-111032450	C/C	T/C	C/C	Affx-109525147	7D	412008592
AX-111034243	T/T	T/C	C/C	Affx-111607583	7D	242266485
AX-111034738	G/G	C/G	G/G	Affx-109869535	7D	216282674
AX-111038335	C/C	T/C	C/C	Affx-111039296	7D	227614380
AX-111038459	A/A	G/G	A/A	Affx-109448269	7D	510445868
AX-111044062	C/C	T/C	C/C	Affx-88624774	7D	189128925
AX-111044186	C/C	T/C	C/C	Affx-109552392	7D	389445296
AX-111048525	C/C	T/C	C/C	Affx-110874970	7D	380291909
AX-111049975	C/C	C/C	C/C	Affx-109761886	7D	399148625
AX-111050077	C/C	T/C	C/C	Affx-109371369	7D	184640574
AX-111053673	G/G	A/G	G/G	Affx-111805091	7D	509551901
AX-111065693	C/C	T/C	C/C	Affx-110013862	7D	330731534
AX-111066894	C/C	T/C	C/C	Affx-110891338	7D	40455264

AX-111077878	G/G	A/G	G/G	Affx-108862169	7D	164793391
AX-111083443	A/A	A/G	A/A	Affx-109112902	7D	326808368
AX-111094029	A/C	A/C	A/A	Affx-88385508	7D	92299226
AX-111094204	G/G	A/G	G/G	Affx-88564690	7D	328962924
AX-111095796	G/G	T/G	G/G	Affx-109195871	7D	172792129
AX-111096386	C/C	A/C	C/C	Affx-110745989	7D	153312097
AX-111100799	G/G	A/G	G/G	Affx-109856304	7D	355247644
AX-111101483	C/C	T/T	C/C	Affx-110357812	7D	136879180
AX-111101678	C/C	A/C	C/C	Affx-110055331	7D	496374172
AX-111106049	G/G	A/G	G/G	Affx-110945151	7D	135928566
AX-111114314	C/C	T/C	C/C	Affx-88517330	7D	411596748
AX-111116449	C/C	A/C	C/C	Affx-110966048	7D	550072905
AX-111117711	C/C	C/G	C/C	Affx-110017852	7D	56464183
AX-111118847	G/G	A/G	G/G	Affx-110805624	7D	264233488
AX-111124428	C/C	T/C	C/C	Affx-110380559	7D	40592370
AX-111126737	T/T	T/C	T/T	Affx-110594501	7D	300248169
AX-111131440	G/G	A/G	A/A	Affx-109016791	7D	25879752
AX-111132781	C/C	A/C	C/C	Affx-110619939	7D	129974864
AX-111133723	G/G	C/G	G/G	Affx-110906578	7D	101586460
AX-111140486	G/G	A/G	G/G	Affx-110145537	7D	306293287
AX-111140758	A/A	A/C	A/A	Affx-109253591	7D	334235968
AX-111144869	T/T	T/C	T/T	Affx-110844987	7D	463792796
AX-111153140	C/C	T/C	C/C	Affx-109512194	7D	184526122
AX-111154799	G/G	A/G	G/G	Affx-109131124	7D	358989499
AX-111158082	T/T	C/C	T/T	Affx-111654329	7D	448341105
AX-111159616	C/C	T/C	C/C	Affx-110911152	7D	608121185
AX-111186183	C/C	C/C	C/C	Affx-88588509	7D	228533251
AX-111187784	A/A	A/G	A/A	Affx-111292925	7D	541494182
AX-111203765	G/G	A/G	A/A	Affx-109131054	7D	237416590
AX-111215243	T/T	T/C	T/T	Affx-111371041	7D	545205343
AX-111217133	G/G	A/G	G/G	Affx-110262160	7D	105463498
AX-111254427	G/G	G/G	G/G	Affx-88654100	7D	553603020
AX-111260293	G/G	A/G	G/G	Affx-110566115	7D	636714974
AX-111261157	T/T	T/C	T/T	Affx-88561901	7D	334953598
AX-111261489	G/G	A/G	G/G	Affx-109272492	7D	183618646
AX-111296245	A/A	A/C	A/A	Affx-110474299	7D	346234109
AX-111301432	G/G	A/G	G/G	Affx-88754003	7D	423338216
AX-111303323	G/G	G/G	G/G	Affx-109945415	7D	51238939
AX-111307694	C/C	A/C	C/C	Affx-109971994	7D	56028745
AX-111310272	C/C	C/C	C/C	Affx-109703423	7D	308919493
AX-111315498	A/A	A/T	A/A	Affx-111242159	7D	427731244
AX-111322109	C/C	T/C	C/C	Affx-110538416	7D	319666082
AX-111327877	C/C	T/C	C/C	Affx-110926365	7D	487724409
AX-111337208	C/C	T/T	C/C	Affx-110159077	7D	334981889
AX-111337913	A/A	A/G	A/A	Affx-110801439	7D	117583720
AX-111353936	T/T	T/C	T/T	Affx-111540771	7D	566214823
AX-111361774	T/T	T/C	T/T	Affx-111931715	7D	113395195
AX-111361834	T/T	T/C	T/T	Affx-110802781	7D	33194684
AX-111372399	T/C	C/C	T/C	Affx-110264509	7D	481188919
AX-111374113	A/A	A/G	A/A	Affx-111132365	7D	287077858
AX-111375003	A/A	A/G	A/A	Affx-110201140	7D	19931270
AX-111379517	G/G	G/G	G/G	Affx-109051218	7D	3790491
AX-111385187	C/C	T/C	C/C	Affx-88578384	7D	217080664
AX-111408945	A/A	A/G	A/A	Affx-111329633	7D	332575308

AX-111422641	C/C	T/C	C/C	Affx-110340686	7D	508655797
AX-111435770	C/C	T/C	C/C	Affx-109167397	7D	413981709
AX-111441433	C/C	C/G	C/C	Affx-109408468	7D	40491800
AX-111454684	G/G	A/G	G/G	Affx-110945158	7D	59173779
AX-111460143	T/T	T/C	T/T	Affx-109441929	7D	162456920
AX-111462186	G/G	G/G	G/G	Affx-111705248	7D	35494300
AX-111468131	G/G	A/G	G/G	Affx-109192158	7D	51960914
AX-111469799	G/G	G/G	G/G	Affx-109674804	7D	72534087
AX-111480696	A/A	A/A	A/A	Affx-109705706	7D	614283454
AX-111481834	G/G	A/G	G/G	Affx-109250960	7D	611240587
AX-111482485	G/G	C/G	G/G	Affx-109682688	7D	11508889
AX-111485210	T/T	T/C	T/T	Affx-109833370	7D	382888416
AX-111485506	C/C	C/G	G/G	Affx-108895041	7D	38489607
AX-111490489	C/C	A/C	A/A	Affx-110717038	7D	76943557
AX-111493609	A/A	A/G	G/G	Affx-111171709	7D	198709865
AX-111501298	A/A	A/G	A/A	Affx-111756362	7D	612777316
AX-111502612	G/G	C/G	G/G	Affx-88365854	7D	592634289
AX-111507520	C/C	T/C	C/C	Affx-110115099	7D	164846275
AX-111510128	G/G	A/G	G/G	Affx-88507442	7D	307661459
AX-111527599	G/G	A/G	G/G	Affx-111833550	7D	500262865
AX-111527690	T/T	T/C	C/C	Affx-109191836	7D	89291499
AX-111529448	C/C	C/C	C/C	Affx-109928588	7D	135365248
AX-111534656	C/C	T/C	C/C	Affx-109186518	7D	212112835
AX-111537181	T/T	T/G	T/T	Affx-109858884	7D	295600632
AX-111538655	T/T	T/C	T/T	Affx-110833353	7D	446095351
AX-111542234	G/G	A/G	G/G	Affx-110833232	7D	191869153
AX-111542944	A/A	G/G	A/A	Affx-111238093	7D	609237748
AX-111544235	C/C	T/C	C/C	Affx-110626779	7D	426628434
AX-111546955	A/A	A/G	A/A	Affx-111391991	7D	384222864
AX-111550027	G/G	T/G	G/G	Affx-110180842	7D	325726738
AX-111553697	A/A	A/G	G/G	Affx-111161445	7D	596379527
AX-111554046	T/T	T/C	T/T	Affx-109839414	7D	194699994
AX-111554730	C/C	T/C	C/C	Affx-111332412	7D	289844956
AX-111558795	C/C	T/C	C/C	Affx-110125959	7D	295430566
AX-111559194	G/G	T/G	G/G	Affx-111623526	7D	501343925
AX-111562975	C/C	A/C	C/C	Affx-109807960	7D	442419654
AX-111586377	C/C	T/C	C/C	Affx-109971952	7D	155679205
AX-111588690	C/C	T/T	C/C	Affx-109426602	7D	328140242
AX-111591751	A/C	C/C	C/C	Affx-111119585	7D	597124845
AX-111593722	C/C	T/T	C/C	Affx-109345972	7D	124292422
AX-111594797	T/T	T/C	T/T	Affx-110701808	7D	636705608
AX-111597983	T/T	T/T	T/T	Affx-111960247	7D	13477031
AX-111600439	G/G	G/G	G/G	Affx-109211181	7D	83807813
AX-111601211	C/C	A/C	A/A	Affx-110300224	7D	243754656
AX-111607406	T/T	A/T	T/T	Affx-111557937	7D	16879459
AX-111612693	G/G	A/G	G/G	Affx-110610221	7D	203945849
AX-111612947	G/G	A/G	G/G	Affx-88667602	7D	499538484
AX-111618969	A/A	G/G	G/G	Affx-110000921	7D	75231024
AX-111619124	G/G	A/A	G/G	Affx-111399993	7D	37141347
AX-111623278	T/T	T/G	T/T	Affx-110004365	7D	4651904
AX-111627051	C/C	T/C	C/C	Affx-110907158	7D	190572959
AX-111629625	G/G	A/G	G/G	Affx-88377566	7D	574672141
AX-111629648	T/C	T/C	C/C	Affx-109838768	7D	24616190
AX-111645035	G/G	A/G	G/G	Affx-88580589	7D	421165352

AX-111647520	C/C	T/C	C/C	Affx-109585437	7D	312160357
AX-111651239	G/G	G/G	G/G	Affx-88468000	7D	234071487
AX-111654081	G/G	A/G	G/G	Affx-110686381	7D	186003054
AX-111658904	G/G	A/G	G/G	Affx-109449671	7D	454057711
AX-111664349	C/C	T/C	C/C	Affx-111227201	7D	7307720
AX-111666498	C/C	T/C	C/C	Affx-110708986	7D	150733742
AX-111680330	G/G	A/G	G/G	Affx-111353913	7D	120305444
AX-111684409	A/A	A/G	G/G	Affx-111473930	7D	94928642
AX-111694366	G/G	A/A	G/G	Affx-108850911	7D	178013279
AX-111723510	C/C	C/C	C/C	Affx-109510439	7D	515224099
AX-111726890	T/T	T/C	T/T	Affx-111037280	7D	3435339
AX-111728699	T/G	T/G	T/G	Affx-110046121	7D	607560711
AX-111729400	G/G	A/G	G/G	Affx-111755997	7D	420359516
AX-111761327	G/G	A/G	G/G	Affx-109210008	7D	422539630
AX-111763481	A/G	A/A	A/G	Affx-109705756	7D	385446310
AX-111764172	C/C	T/C	C/C	Affx-109781881	7D	362382364
AX-111807186	C/C	C/C	C/C	Affx-111883859	7D	40538801
AX-111839503	A/A	A/A	A/A	Affx-109214040	7D	215425762
AX-111839726	A/A	G/G	A/A	Affx-108852385	7D	74660741
AX-111839987	G/G	A/G	G/G	Affx-110298196	7D	374006817
AX-111842678	T/C	T/T	T/T	Affx-111862113	7D	18685405
AX-111858146	G/G	A/G	G/G	Affx-88340733	7D	370726892
AX-111876137	T/T	T/C	T/T	Affx-111093487	7D	193176799
AX-111876609	G/G	A/G	G/G	Affx-109561716	7D	346809166
AX-111879582	C/C	T/C	C/C	Affx-109000876	7D	470963878
AX-111887665	C/C	T/C	C/C	Affx-111704132	7D	270739365
AX-111888940	T/T	T/C	C/C	Affx-110543573	7D	232635868
AX-111913853	C/C	T/C	C/C	Affx-111131999	7D	474681405
AX-111914752	G/G	A/G	A/A	Affx-108890846	7D	86980257
AX-111914903	T/T	T/C	T/T	Affx-110670085	7D	576912596
AX-111917524	C/C	T/T	C/C	Affx-111217691	7D	519402236
AX-111928374	C/C	C/C	C/C	Affx-111095241	7D	636672802
AX-111961125	A/A	A/G	G/G	Affx-109657137	7D	248399010
AX-111971080	C/C	C/G	G/G	Affx-111542304	7D	90218786
AX-111973087	G/G	A/G	G/G	Affx-110006834	7D	145696067
AX-111980205	C/C	T/C	C/C	Affx-110122372	7D	12544962
AX-111988581	C/C	T/C	C/C	Affx-110951905	7D	316262749
AX-158555102	A/G	G/G	A/G	Affx-92990822	7D	5448289
AX-179388241	C/C	C/C	C/C	Affx-292406711	7D	55073787
AX-179475741	A/G	G/G	A/G	Affx-88406383	7D	518781335
AX-182023442	A/A	A/G	A/A	Affx-110580289	7D	40592680
AX-182076818	T/T	T/T	T/T	Affx-92670692	7D	35601907
AX-86171376	A/A	A/A	A/A	Affx-88473889	7D	7446590
AX-86177645	T/T	T/G	T/T	Affx-92232301	7D	391576285
AX-86178429	T/C	T/T	C/C	Affx-92905870	7D	58868443
AX-89378255	G/G	T/G	T/T	Affx-88410620	7D	47379368
AX-89381705	T/T	T/C	T/T	Affx-88414101	7D	568597256
AX-89393548	C/C	T/C	C/C	Affx-88426043	7D	185764663
AX-89434097	T/C	C/C	T/C	Affx-88466802	7D	4293394
AX-89474682	C/C	T/C	C/C	Affx-88507505	7D	120095772
AX-89516827	T/T	C/C	T/T	Affx-88549724	7D	13896220
AX-89544184	T/T	T/C	C/C	Affx-109602356	7D	208677935
AX-89661459	G/G	A/G	G/G	Affx-88694253	7D	595740453
AX-89695640	T/T	T/G	G/G	Affx-88728388	7D	193595264

AX-89742838	T/T	T/C	T/T	Affx-88775525	7D	199413838
AX-89753436	G/G	A/G	G/G	Affx-110198562	7D	158971054
AX-94391955	T/C	T/C	T/T	Affx-92682786	7D	92570636
AX-94393365	C/C	T/C	C/C	Affx-92188110	7D	636770831
AX-94396369	C/C	C/C	C/C	Affx-92234938	7D	53711630
AX-94409926	T/G	T/G	T/G	Affx-92637084	7D	472287868
AX-94459226	G/G	A/G	G/G	Affx-92193776	7D	51446384
AX-94470019	A/A	A/T	A/A	Affx-92546520	7D	35600378
AX-94519408	G/G	G/G	G/G	Affx-88524790	7D	579535413
AX-94567102	C/C	C/C	C/C	Affx-92333235	7D	575864262
AX-94567789	C/C	T/T	T/C	Affx-92661027	7D	623541660
AX-94583324	C/C	T/T	C/C	Affx-92119265	7D	20040742
AX-94593186	C/G	C/C	C/G	Affx-92313361	7D	2090033
AX-94618563	C/C	T/T	C/C	Affx-92137891	7D	397567649
AX-94625159	T/C	T/T	T/C	Affx-92914064	7D	51444768
AX-94634495	T/C	T/C	T/C	Affx-88652193	7D	73745695
AX-94688475	A/A	A/A	A/A	Affx-92899069	7D	156812705
AX-94726728	T/T	T/C	T/T	Affx-92775999	7D	35598453
AX-94789220	G/G	G/G	G/G	Affx-92259499	7D	8462708
AX-94896209	C/C	C/G	C/C	Affx-92702590	7D	51443468
AX-94898470	C/C	A/C	C/C	Affx-92164260	7D	520862010
AX-94923899	C/C	T/T	C/C	Affx-92454054	7D	35602093
AX-94930280	A/A	A/C	A/A	Affx-92328216	7D	16119691
AX-94950579	G/G	G/G	G/G	Affx-92900200	7D	571259108
AX-94950712	A/G	G/G	A/G	Affx-88664733	7D	565198540
AX-94976822	G/G	G/G	G/G	Affx-92135632	7D	326727292
AX-94978407	G/G	G/G	G/G	Affx-92881794	7D	588623995
AX-94983189	A/A	A/G	A/A	Affx-92901133	7D	35602045
AX-95079229	G/G	A/G	G/G	Affx-92848138	7D	53995989
AX-95105904	T/T	C/C	T/T	Affx-92792007	7D	247748048
AX-95109583	T/C	T/T	T/C	Affx-92830937	7D	575864292
AX-95149671	T/C	T/T	C/C	Affx-92762730	7D	607042805
AX-95158932	C/C	G/G	C/C	Affx-92493116	7D	635581280
AX-95198242	G/G	G/G	G/G	Affx-92924843	7D	591204168
AX-95202508	C/C	A/C	C/C	Affx-92779093	7D	634641526
AX-95220206	C/C	A/A	A/C	Affx-92503930	7D	2242792
AX-95630733	G/G	G/G	A/A	Affx-92979759	7D	235982986
AX-95631759	G/G	G/G	G/G	Affx-88399217	7D	4517551
AX-95682130	A/G	A/A	A/G	Affx-88703258	7D	13001473
