# Supplementary material for: The addition of Psathyrostachys Huashanica Keng 6Ns large segment chromosomes has positive impact on stripe rust resistance and plant spikelet number of common wheat
Source: BMC Plant Biol. 2024 Jul 18;24:685. doi: 10.1186/s12870-024-05395-9 (PMC11256485; doi:10.1186/s12870-024-05395-9)

1. Original image of gel electrophoresis in SCAR marker analysis. Lanes M-4 shown in the figure are DL2000 marker, wheat parent 7182, Trs-372, D88-2a addition line, and alien donor *P. huashanica*.

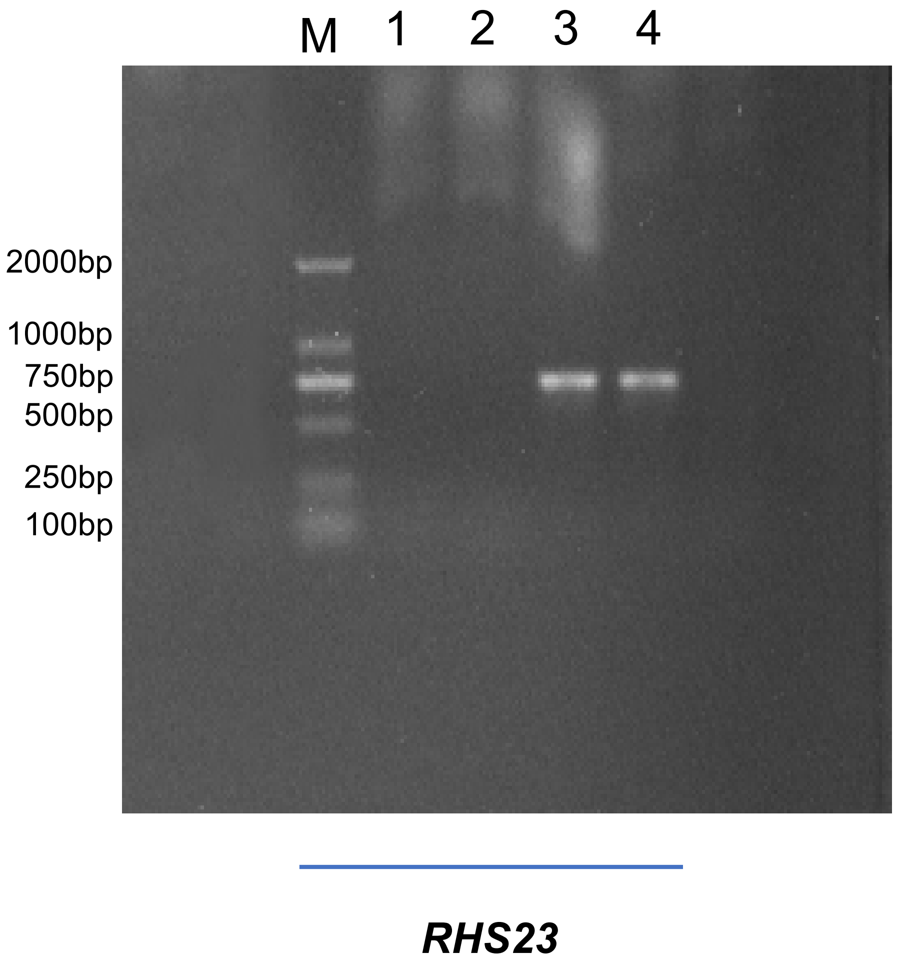

2. Original image of gel electrophoresis in STS marker analysis. Lanes M-4 shown in the figure are DL2000 marker, wheat parent 7182, Trs-372, D88-2a addition line, and alien donor *P. huashanica*. DL2000 marker: 2000bp-1000bp-750bp-500bp-250bp-100bp.

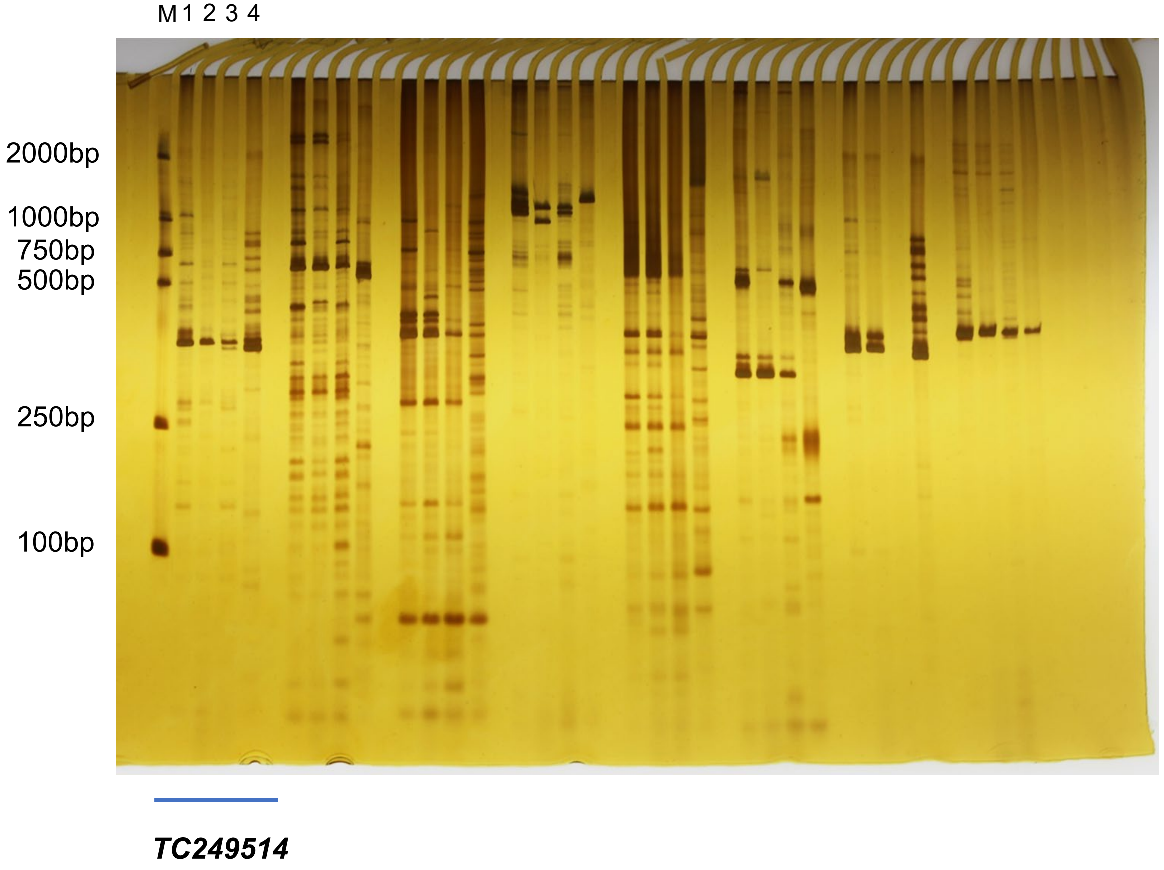

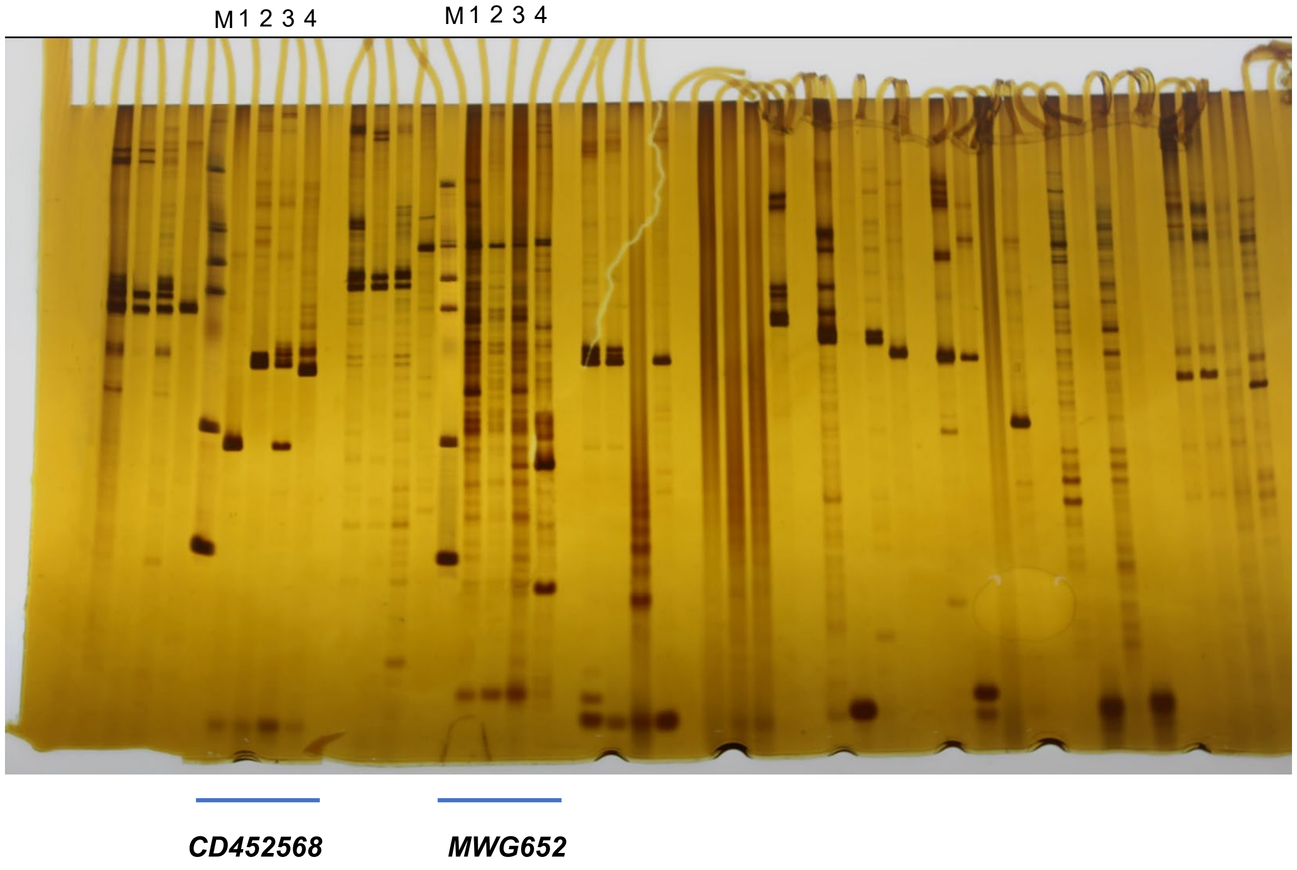

3. Original image of gel electrophoresis in gliadins analysis through A-PAGE experiment. *P.hua* indicated *P. huashanica.*

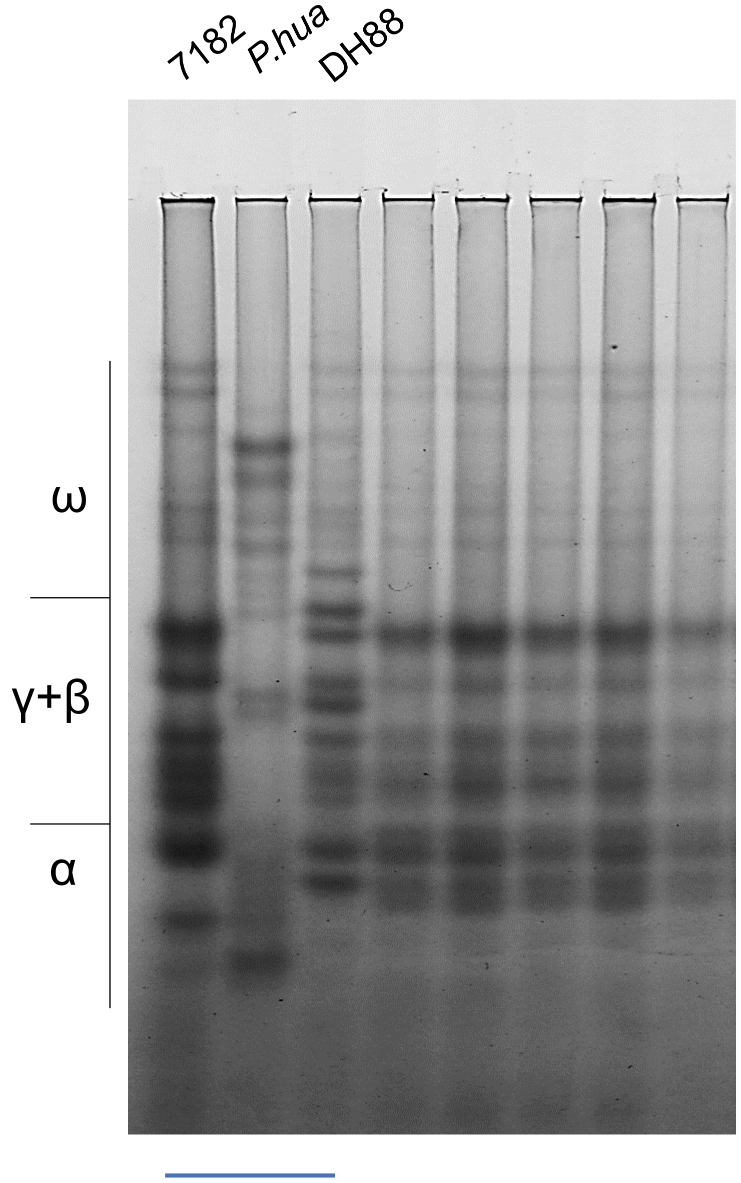

Supplement: Supplementary file 3 — Supplementary Material 3 [file 12870_2024_5395_MOESM3_ESM.pdf]
